# Analysis of deep sequencing exosome‐microRNA expression profile derived from CP‐II reveals potential role of gga‐miRNA‐451 in inflammation

**DOI:** 10.1111/jcmm.15244

**Published:** 2020-04-19

**Authors:** Yabo Zhao, Yali Fu, Mengyun Zou, Yingfei Sun, Xun Yin, Lumeng Niu, Yanzhang Gong, Xiuli Peng

**Affiliations:** ^1^ Key Laboratory of Agricultural Animal Genetics, Breeding and Reproduction Ministry of Education Huazhong Agricultural University Wuhan China; ^2^ College of Animal Science and Technology and College of Veterinary Medicine Huazhong Agricultural University Wuhan China

**Keywords:** exosomes, gga‐miR‐451, inflammatory cytokines, *Mycoplasma gallisepticum* (HS strain), *YWHAZ*

## Abstract

*Mycoplasma gallisepticum* (MG) can cause chronic respiratory disease (CRD) in chickens. While several studies have reported the inflammatory functions of microRNAs during MG infection, the mechanism by which exosomal miRNAs regulate MG‐induced inflammation remains to be elucidated. The expression of exosome‐microRNA derived from MG‐infected chicken type II pneumocytes (CP‐II) was screened, and the target genes and function of differentially expressed miRNAs (DEGs) were predicted. To verify the role of exosomal gga‐miR‐451, Western blot, ELISA and RT‐qPCR were used in this study. The results showed that a total of 722 miRNAs were identified from the two exosomal small RNA (sRNA) libraries, and 30 miRNAs (9 up‐regulated and 21 down‐regulated) were significantly differentially expressed. The target miRNAs were significantly enriched in the treatment group, such as cell cycle, Toll‐like receptor signalling pathway and MAPK signalling pathway. The results have also confirmed that gga‐miR‐451‐absent exosomes derived from MG‐infected CP‐II cells increased inflammatory cytokine production in chicken fibroblast cells (DF‐1), and wild‐type CP‐II cell–derived exosomes displayed protective effects. Collectively, our work suggests that exosomes from MG‐infected CP‐II cells alter the dynamics of the DF‐1 cells, and may contribute to pathology of the MG infection via exosomal gga‐miR‐451 targeting YWHAZ involving in inflammation.

## 1 INTRODUCTION

*Mycoplasma gallisepticum* (MG) is one of the most important avian pathogens. It is established that MG infection causes the chronic respiratory disease (CRD) leading to respiratory symptoms including rales, coughs, ocular discharge and nasal discharge in chickens and turkeys.^1^, ^2^ Increased embryonic mortality, reduced weight gain and egg production have been found in infected chickens, typically causing considerable economic losses to the poultry industry.^3^, ^4^

microRNAs (miRNAs) are one of the short endogenous single non‐coding RNAs of 18‐26 nucleotides (nt) in length.^5^, ^6^ Recent studies have shown that miRNAs are involved in the occurrence and development of inflammatory diseases.^7^ Our previous studies have also revealed that miRNAs are associated with MG infection and that they were differentially expressed in chicken lungs at 3 days after infection with 36 down‐regulated and 9 up‐regulated miRNAs belonging to 31 miRNA families.^8^ gga‐miR‐146c represses the expression of MMP16, activating TLR6/MyD88/NF‐κB pathway for promoting cell proliferation to defend against MG infection.^9^ The mature sequences of miR‐451 contain 22 nt in length and are highly conserved pending evolvement.^10^ Recent data clarified the role of miR‐451 in the cell apoptosis, migration and invasion. miR‐451 has also been reported to play a critical role in regulating a variety of human and animal diseases, such as influenza, cancer, cerebral ischaemia/reperfusion injury and vascular endothelial dysfunction.^11^, ^12^, ^13^ It is worth mentioning that gga‐miR‐451 modulates the MG‐induced production of inflammatory cytokines by targeting YWHAZ.^14^ Moreover, our previous study also confirmed that AhR:Arnt binds to the gga‐miR‐451 promoter and facilitates gga‐miR‐451 transcription in MG‐infected DF‐1 cells.^15^

Exosomes, approximately 30‐150 nm, are the small membranous vesicles, released by many kinds of cells (including B lymphocytes, endothelial cells and epithelial cells) and body fluids. They are known to represent the characteristics of the cells which secrete them.^16^, ^17^ The mode of intercellular communication which had achieved positive acknowledgement is mediated by exosomes. In juxtacrine or ectodomain cleavage‐based signalling, exosomes can regulate the non‐selective transfer of exosomal proteins and/or RNA to the target cell via fusing with the target cell.^17^ In addition to cellular proteins, lipids and functional RNA molecules and other non‐coding RNAs (miRNAs and lncRNAs) were also found in exosomes. Growing evidence indicates that exosome‐transferred miRNAs are also biologically active via regulating the mRNA level in target cells after entering the target cells.^18^, ^19^ Exosomes‐miRNAs secreted from the donor cells to the extracellular environment can modulate gene expression and cell function, playing a crucial role in several processes such as inflammation and immune response.^20^, ^21^ However, much of our previous understanding regarding miRNA‐mediated effects on mechanisms of MG infection is based on the use of ‘unpackaged’ miRNAs, which regulate the original cells and tissues. The function of exosomal miRNAs derived from MG‐infected chicken lung epithelial cells on both neighbouring and distal cells is still unknown. To expand on our knowledge, it is now critical to assess the effect of miRNAs in a biologically relevant setting in which they are present as exosomal cargo. The purpose of this study is to screen and annotate the differentially expressed exosomal miRNAs in MG‐infected CP‐II cells and to evaluate the function and potential mechanism of exosomal gga‐miR‐451 in DF‐1 cells, which provides a valuable miR‐451 as diagnostic markers and therapeutic targets in MG‐infected chickens.

## 2 MATERIALS AND METHODS

### 2.1 Reagents

The primary antibody anti‐YWHAZ was obtained from Sangon Biotech (Cat. D155211‐0025), the primary antibodies anti‐CD9 and anti‐CD63 were purchased from Abcam (Cat. ab92726 and ab193349), and the primary antibody against glyceraldehyde 3‐phosphate dehydrogenase (GAPDH) was acquired from Proteintech (Cat. 60004‐1‐1g). The secondary antibody horseradish peroxidase‐conjugated antimouse/anti‐rabbit was purchased from Sangon Biotech (Shanghai, China). The oligonucleotides of gga‐miR‐451 mimics and negative control (double‐stranded chemically modified oligonucleotides) were synthesized by GenePharma (Shanghai, China). The sequences of all the primers and the sequences of RNA oligonucleotides used in the study are shown in Tables S1 and S2 **,** respectively.

### 2.2 CP‐II isolation and DF‐1 cell culture

CP‐II cells were isolated as described previously,^22^ with modifications. Briefly, lungs were removed from 14‐day‐old White Leghorn specific‐pathogen‐free (SPF) chicken embryos and were cut into tiny tissue blocks. Then, 0.25% trypsin and 0.1% IV collagenase (Invitrogen‐Gibco) were used for digestion at 37°C for 15 and 20 minutes, respectively. Cell suspensions were filtrated by 200‐mesh sieves, resuspended with 10% foetal bovine serum (FBS; Invitrogen) in a 150‐mm sterile culture plate and incubated for 1 hour. Supernatants with the unattached cells were then collected three times. The unattached cells were centrifuged at 1200 r/min for 5 minutes, resuspended in fresh Dulbecco's modified Eagle's medium (DMEM) for three times and filtrated by 400‐mesh sieves. After the cell count was completed, cells were with 20% FBS and concentration was adjusted to 2 × 10^6^/mL and then inoculated into 150‐mm sterile culture plate. Cells were incubated for 18 hours at 39°C. The attaching cells on culture dish were CP‐II cells. Invert microscope and transmission electron microscope (TEM) were used to identify the cells.

The chicken embryonic fibroblast cell line (DF‐1), obtained from ATCC (Manassas, VA, USA), was maintained at 39°C in a 5% CO_2_ atmosphere in DMEM (Invitrogen) mixed with 1% penicillin‐streptavidin‐glutamine (PSG, Invitrogen) and 10% foetal bovine serum (FBS, Invitrogen).

### 2.3 *Mycoplasma* strains

MG‐HS, a virulent strain, was isolated from a chicken farm in Hubei, China, and conserved in the State Key Laboratory of Agricultural Microbiology, Huazhong Agricultural University (Wuhan, China).^23^, ^24^ The MG‐HS strain was cultured, and the concentration was determined as previously described. The viable number of MG‐HS in suspension was measured using a colour‐changing unit (CCU) assay.^25^

### 2.4 Infection experiments

When CP‐II cells in the experimental group reached 80‐90% confluence, twelve 150‐mm plates of the cells were incubated in medium without antibiotics. Six plates of the cells were infected with 2 mL/plate of MG‐HS at the mid‐exponential phase (1 × 10^12^ CCU/mL), while the other six plates of the cells were uninfected with MG‐HS used as a control. At 12 hours after infection, the medium of the cells in both groups was changed to exosome‐depleted media prepared by ultracentrifugation of FBS at 100 000 × g for 70 min.^26^ The culture supernatant and the cells were collected at 12‐60 hpi for further experiments.

### 2.5 Exosome isolation and characterization

Sixty mL of CP‐II cell culture supernatants in both groups was collected and immediately filtered through a 0.22‐mm filter (Millipore, USA) and centrifuged immediately at 2000 × g for 10 minutes at 4°C. Cell culture exosomes were isolated and characterized as described previously.^27^, ^28^, ^29^ Briefly, the supernatant was collected and then centrifuged at 10 000g for 40 minutes at 4°C. The resulting supernatant was transferred to a new ultracentrifuge tube and centrifuged at 100,000 × g for 2 hours at 4°C (Beckman Optima XE‐90, SW32 Ti rotor). The supernatant was aspirated, and the pellet was suspended in pure PBS (HyClone, USA) and centrifuged at 100 000 × g for another 2 hours at 4°C. The purified exosomes were resuspended in 50 μL PBS and used for experimental procedures or stored at −80°C. For nanoparticle tracking analysis (NTA), exosomes were diluted with PBS over a range of concentrations to obtain between 10 and 100 particles per image before analysis. The ZetaView Nanoparticle Tracking Analyzer (Particle Metrix, Germany) was used to automatically measure the average diameter and concentration. For transmission electron microscopy, a 10 μL aliquot of the suspended exosomes was applied to a carbon‐coated copper grid. Then, the sample was negatively stained with 2% uranyl acetate after drying. Micrographs were obtained under a HITACHI H‐7650 transmission electron microscope (HITACHI, Japan).

### 2.6 Exosome labelling

Exosomes secreted by CP‐II cells were labelled with a PKH67 green fluorescent labelling kit (Sigma‐Aldrich, MINI67, MO, USA) to detect the uptake of exosomes by DF‐1 cells in vitro. Labelled exosomes were incubated with DF‐1 cells at 39°C for 6 hours and then fixed. Fluorescent images were taken with a confocal laser scanning microscope (Zeiss LSM 800 META, Carl Zeiss Imaging, Germany).

### 2.7 Exosome sRNA sequencing and data processing

Exosomes were isolated from samples and characterized as described above. Total RNA from exosomes in both groups (n = 3) was used for sRNA sequencing. Library preparation and sRNA sequencing were performed by RiboBio (Guangzhou, China). In brief, total RNA samples were fractionated and only small RNAs ranging from 18 to 40 nts were used for library preparation. After amplification by 15‐cycle PCR, the libraries were sequenced using the Illumina HiSeq^TM^ 2500 platform. Clean reads were collected from raw reads by removing the adapter dimers, glow quality and contaminated reads. Then, clean reads of sRNA were mapped to the chicken (*G gallus*) genome by BWA 0.7.12.^30^ Mapped sRNA tags were used to look for known sRNA, and miRBase version 21.0 (www.mirbase.org), Rfam 12.1 (rfam.xfam.org) and piRNABank (pirnabank.ibab.ac.in) were used for reference. The sequencing data were deposited at NCBI SRA under accession number PRJNA606684.

### 2.8 Differentially expressed miRNA analysis

Two groups of miRNA expression levels [MG‐infected (MG) and non‐infected (NC)] were analysed using the edgeR software and showed by log_2_ (fold change) and scatter plot. miRNAs were considered differentially expressed if the log_2_ (expression in MG/ expression in NC) was >1 or <−1 (|log_2_^(Fold Change)^| ≥ 1) and adjusted *P* ＜ .05).miRNA target prediction and function annotation

2.9

Prediction of target genes of differentially expressed miRNAs (|log_2_^(Fold Change)^| ≥ 1, *P* ＜ .05) was performed by RNAhybrid, miRanda and PITA. GOseq‐based Wallenius non‐central hyper‐geometric distribution was performed for Gene Ontology (GO) enrichment analysis. KOBAS (v2.0) software was performed for Kyoto Encyclopedia of Genes and Genomes (KEGG) pathway analysis, and the corrected *P*‐value (FDR) cut‐off was set at .05.^31^, ^32^

### 2.10 Real‐time quantitative PCR 

The extraction of total RNA from exosomes and reverse transcription into cDNA were performed with a themiRcute Plus miRNA First‐Strand cDNA Synthesis Kit (#KR211‐02, Tiangen Biotech Co., Ltd), according to the manufacturer's recommendation. The miRNA RT‐qPCR analysis was performed with a miRcute Plus miRNA qPCR Detection Kit (SYBR Green) (#FP411‐02, Tiangen Biotech Co., Ltd) and a Bio‐Rad CFX384 TouchTM instrument (Bio‐Rad). Total RNA from cells was isolated using the TRIzol reagent according to the manufacturer's instructions (Invitrogen), and cDNA was synthesized using the PrimeScript^TM^ RT Reagent Kit with gDNA Eraser (TaKaRa). The RT‐PCR was subsequently determined using a TransStart Top Green qPCR SuperMix (Transgen, China). RT‐qPCR was performed to measure the expression levels of exosomal miRNAs and YWHAZ with chicken 5S RNA and GAPDH mRNA used as internal controls. Data were analysed using the 2^−ΔΔCt^ method. The primers of miRNA, mRNA and 5s RNA are presented in Table S1.

### 2.11 Western blotting

The total proteins were extracted from exosomes or DF‐1 cells at 48 hours after transfection or exosome‐treated, and their concentrations were then determined using a BCA Protein Assay Kit (Transgen, China). Equal amounts of protein (5 μg of exosomal proteins and 50 μg of cell proteins) were transferred to nitrocellulose membranes after separation using 12% SDS‐polyacrylamide gel electrophoresis (SDS‐PAGE) (Beyotime, China) and blocked in 5% (w/v) skimmed milk at 25 ± 1°C for 1 hours, followed by incubation overnight at 4°C with the previous antibodies (anti‐CD9, CD63, 14‐3‐3ζ or GAPDH). After washing with TBST, the membranes were incubated with the secondary antibodies at room temperature for 1.5 hours. After another wash with TBST, the protein expressions on the membranes were detected using an ECL^TM^ detection system (Bio‐Rad).

### 2.12 ELISA

After exosome treatment or transfection with siRNA in 6‐well plates, the DF‐1 cells were incubated for 36 hours; then, the culture supernatants were collected from the treated cells for determination of the inflammatory factor levels, including TNF‐α and IL‐1β. All experiments were performed according to the manufacturers’ protocols of the enzyme‐linked immunosorbent assay (ELISA) kits (Bio‐Swamp).

### 2.13 Statistical analysis

Data were presented as the mean ± SEM and analysed by SPSS 20.0 software for statistical analysis. ANOVA or Student's *t* test was used to determine significant differences between groups. A value of *P* < .05 was considered statistically significant and *P* < .01 considered extremely significant. (*P ＜ .05 and **P ＜ .01; different lowercase letters represent *P* < .01).

## 3 RESULTS

### 3.1 CP‐II **cell–derived exosomes were isolated and identified in morphology and phenotype**

**FIGURE 1:**
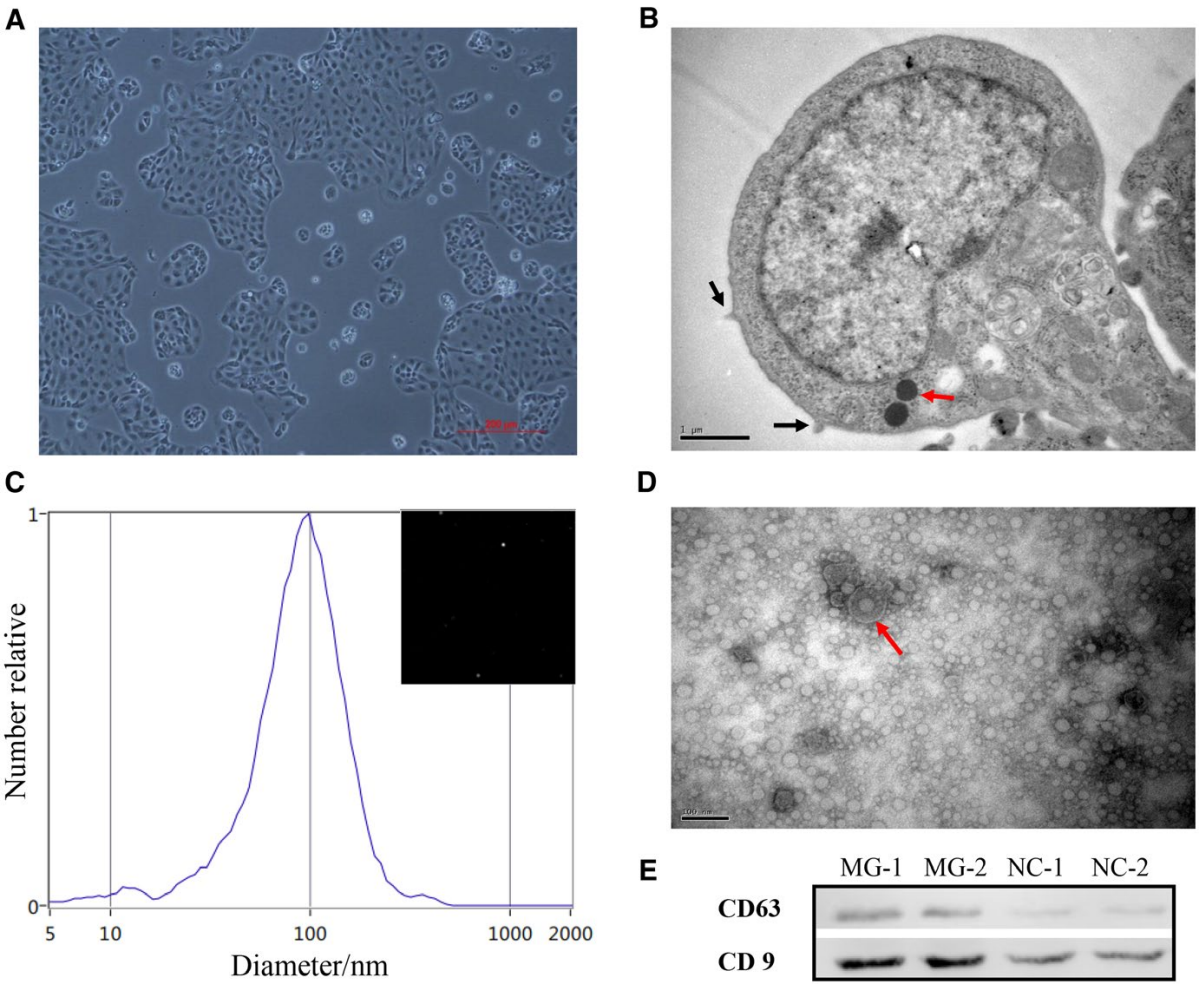
Identification of chicken type II pneumocytes (CP‐II) cells and exosomes. A, CP‐II cells shaped monolayers (scale bar, 200 μm). B, CP‐II cell structure (*Red arrow* indicates the osmiophilic lamellar body, and black *arrow* indicates microvilli; scale bar, 1 μm). C, Particle size distribution in exosome‐enriched fractions. D, Electron micrograph showing whole‐mount exosomes isolated from the supernatant fluid of cultured CP‐II cells (*Red arrow* indicates exosomes; scale bar, 100 nm). E, Western blot analysis showing exosome marker CD63 and CD9

The CP‐II cells shaped monolayers when adherent cultured for 18 hours, were small and cycloid and grouped together to form islands (Figure 1A). The morphology of CP‐II cells was observed by optical microscope and transmission electron microscopy (TEM). The cells have one or more osmophilic lamellar bodies and the surface microvilli (Figure 1B). The exosomes were isolated from the media and collected from MG‐infected (MG) and non‐infected (NC) CP‐II cells by high‐speed centrifugation and were characterized by nanoparticle tracking analysis (NTA) and transmission electron microscope (TEM). These results showed an average particle diameter of 30‐150 nm (Figure 1C **)**, double‐layer membrane structure of round‐shaped vesicles and diameters about 100 nm (Figure 1D). The exosomal surface markers [cluster of differentiation 9 (CD9) and CD63] could be detected using Western blotting (Figure 1E). There was difference in the MG‐infected and non‐infected CP‐II cells, and the group of MG infection is more consistent with previous reports on exosomes.^33^ These results demonstrate that CP‐II cell**–**derived particles collected in this experiment were identified as exosomes.

### 3.2 Sequence analysis

To identify exosomal miRNAs and their expression levels from MG‐infected and non‐infected CP‐II cells, two small RNA libraries were constructed for deep sequencing. The total reads reached more than thirteen million, and approximately 90.16% clean reads are listed in Table S3. These clean reads were matched to authoritative miRNA/rRNA/tRNA/snRNA/snoRNA databases, such as miRBase version 21, Rfam 12.1 and piRNABank. The length distribution range of these libraries is 19‐24, and 22 nt is the main length in the miRNA sequence distribution.

### 3.3 Differentially expressed miRNAs

**FIGURE 2:**
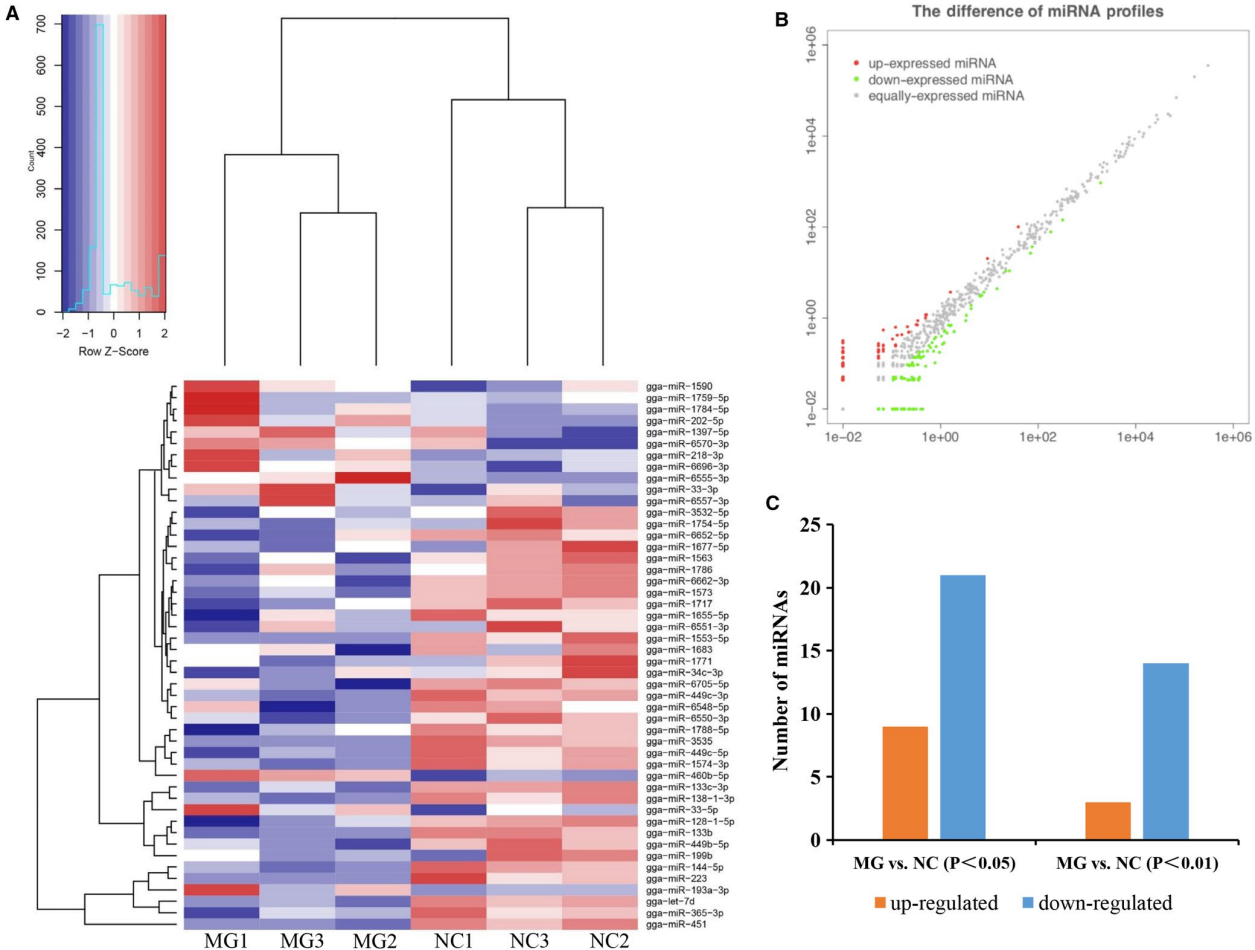
Cluster analysis of differentially expressed miRNAs. A, Heatmap of differentially expressed miRNAs between two groups (each row indicates a microRNA, and each column represents one sample; the colour scale illustrates the microRNA expression level). B, The difference of miRNAs profiles (each red point indicates up‐expressed miRNA, each green indicates down‐expressed miRNA, and grey indicates equally expressed miRNA). C, Differentially expressed miRNAs

A total of 722 mature chicken miRNAs were identified from the two exosomal sRNA libraries, and 279 novel miRNAs were discovered using miRDeep2 software (Table S4 and Figure S1 **)**. As shown in Figure 2A, the differential enriched miRNA profiles of all samples of MG‐infected (MG) and non‐infected (NC) were evaluated by edgeR. The transcriptional level of co‐expressing miRNAs was compared between MG‐infected and non‐infected libraries (Figure 2B). 469 of 722 miRNAs (65%) were equally expressed in the two libraries, while 253 miRNAs (35%) were differential expressed with a fold change > 1 (|log_2_^(Fold Change)^| ≥ 1) (Table S5). Among these differential expressed miRNAs, only 30 miRNAs (9 up‐regulated and 21 down‐regulated) were significantly differentially expressed (*P* ＜ .05) and 17 miRNAs (3 up‐regulated and 14 down‐regulated) were highly significantly different (*P* ＜ .01) in the MG‐infected vs. non‐infected groups (Figure 2C). The details of the differentially expressed chicken miRNAs and their log2 ^(fold change)^ and *P*‐value were shown in Table 1.

**TABLE 1:** List of significantly different exosomal miRNAs presented in the exosomes (fold Change), and these miRNAs are all known miRNAs

sRNA	Up/down	Log2 fold change	*P*‐value
gga‐let‐7d	Down	−1.1638	1.12E‐05
gga‐miR‐193a‐3p	Up	1.3701	1.53E‐05
gga‐miR‐451	Down	−1.0392	4.19E‐05
gga‐miR‐133c‐3p	Down	−1.2484	5.16E‐05
gga‐miR‐33‐5p	Up	1.1421	6.86E‐05
gga‐miR‐223	Down	−1.4015	9.18E‐05
gga‐miR‐460b‐5p	Up	1.199	0.00011881
gga‐miR‐144‐5p	Down	−1.0368	0.00021744
gga‐miR‐1574‐3p	Down	−1.9138	0.0006816
gga‐miR‐365‐3p	Down	−1.2153	0.00120446
gga‐miR‐449c‐5p	Down	−1.5737	0.00228921
gga‐miR‐138‐1‐3p	Down	−1.0125	0.00245147
gga‐miR‐3535	Down	−1.409	0.00301547
gga‐miR‐133b	Down	−1.156	0.0037047
gga‐miR‐449c‐3p	Down	−1.9064	0.00477357
gga‐miR‐128‐1‐5p	Down	−1.0753	0.00834198
gga‐miR‐1573	Down	−2.9042	0.00844519
gga‐miR‐202‐5p	Up	3.0172	0.01188627
gga‐miR‐1553‐5p	Down	−2.0543	0.01246738
gga‐miR‐449b‐5p	Down	−1.0932	0.01649992
gga‐miR‐1784‐5p	Up	2.3769	0.02353568
gga‐miR‐6550‐3p	Down	−2.0485	0.02378457
gga‐miR‐6555‐3p	Up	1.1886	0.02466438
gga‐miR‐1648‐5p	Down	−5.4263	0.02542181
gga‐miR‐1783	Up	4.985	0.02648076
gga‐miR‐199b	Down	−1.7126	0.0286867
gga‐miR‐6696‐3p	Up	1.3609	0.03085024
gga‐miR‐218‐3p	Up	1.2401	0.0338403
gga‐miR‐1788‐5p	Down	−1.1512	0.04110186
gga‐miR‐1771	Down	−2.4316	0.04355199

### 3.4 Functional annotation and enrichment analysis of predicted target genes of the differentially expressed miRNAs

**FIGURE 3:**
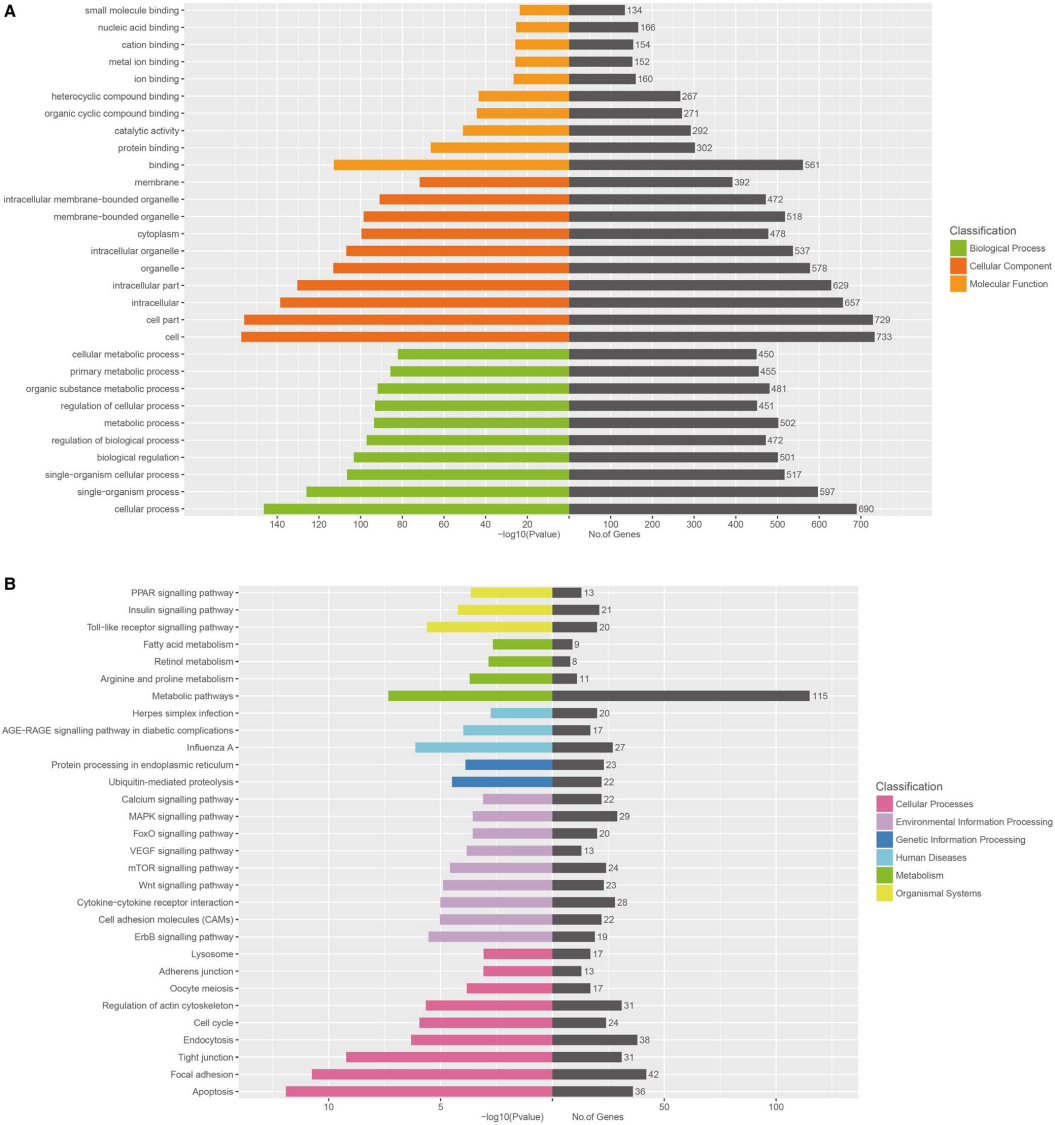
Functional annotation and enrichment analysis of differentially expressed miRNAs. A, Enriched GO terms of different expression exosomal miRNAs (blue represents the biological process; red represents cellular component; and orange indicates molecular function). The y‐axis shows gene functions, and the x‐axis corresponds to the per cent of miRNAs and number of samples. B, Kyoto encyclopedia of genes and genomes enrichment analysis of differentially expressed miRNAs in chicken type II pneumocyte‐derived exosomes (the most enriched top 30 pathway terms: pink, cellular processes; purple, environmental information processing; blue, genetic information processing; light blue, human diseases; green, metabolism; and yellow, organismal systems). The horizontal axis denoted the −log10 (*P*‐value) and the number of miRNAs, while the vertical axis indicated the pathway name. The right grey histogram indicates the numbers of samples in each cluster

To further understand the biological function of miRNAs in CP‐II cell**–**derived exosomes, a target prediction of miRNAs with log_2_^(fold change)^ ≥ 1 and *P* ＜ 0.05 was performed with miRanda, Pita and RNAhybrid (Table S6 **)**. Gene Ontology (GO) functional annotation of target genes was conducted with assignments to biological process (BP), cellular component (CC) and molecular function (MF) categories. The results showed that some items of BP were involved in regulating cellular process, single‐organism process and metabolic process; some items of CC were involved in regulating cell part, intracellular membrane‐bounded organelle; and some items of MF were involved in regulating protein binding, organic cyclic compound binding and catalytic activity (Figure 3A; corrected *P* < .05). Analysis of Kyoto Encyclopedia of Genes and Genomes (KEGG) pathway for these target genes was performed (*P* < .05). These target genes were mainly enriched in the following pathways: apoptosis, cell cycle, focal adhesion, tight junction, influenza A, Toll‐like receptor signalling pathway, Wnt signalling pathway, cytokine‐cytokine receptor interaction and MAPK signalling pathway (Figure 3B). These pathways reflect the physiological processes of MG infection and the potential regulatory mechanisms underlying them.

### 3.5 Exosomal miRNA interaction network for elements involved in cell cycle, apoptosis and Toll‐like receptor signals

**FIGURE 4:**
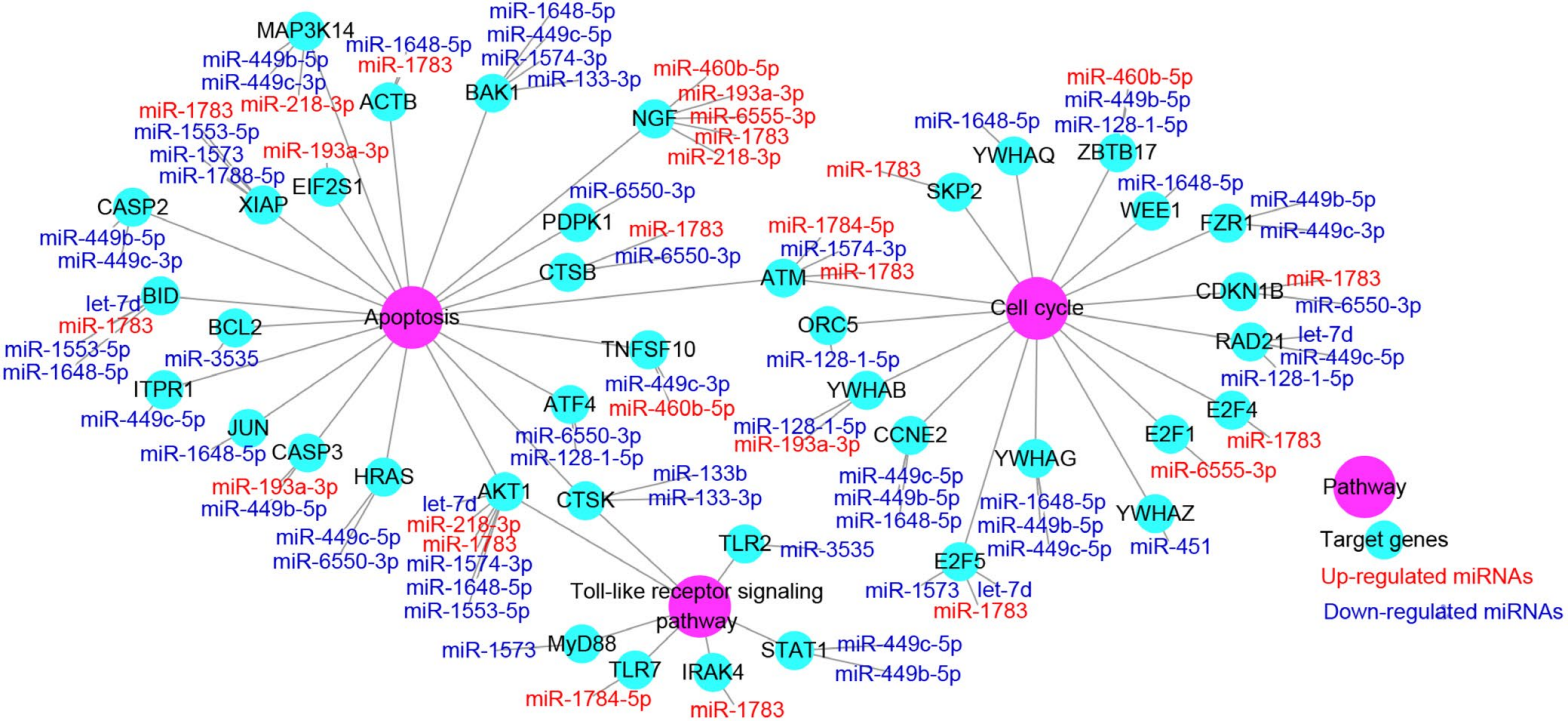
Network of miRNA target pathways involved in cell cycle, apoptosis and Toll‐like receptor signalling pathway for differential expressed miRNAs. The pink represents the pathways, green represents the genes, the red represents the up‐regulated miRNAs, and the blue represents the down‐regulated miRNAs

Our previous studies have shown that cell cycle, apoptosis and Toll‐like receptor signalling pathways are activated and play an important role in the inflammatory response to MG infection.^14^, ^34^ We found that 20 predicted targets of DEGs are involved in regulating apoptosis, 16 predicted targets of DEGs are involved in cell cycle, and 16 predicted targets of DEGs are involved in Toll‐like receptor signalling pathway (Figure 4). Interestingly, most of miRNAs (21 of 30) negatively regulate these three pathways by targeting the positive genes. Moreover, some other pathways are also modulated by DEGs, such as mitogen‐activated protein kinase (MAPK) and cytokine‐cytokine receptor interaction signals.

### 3.6 RT‐qPCR verification

**FIGURE 5:**
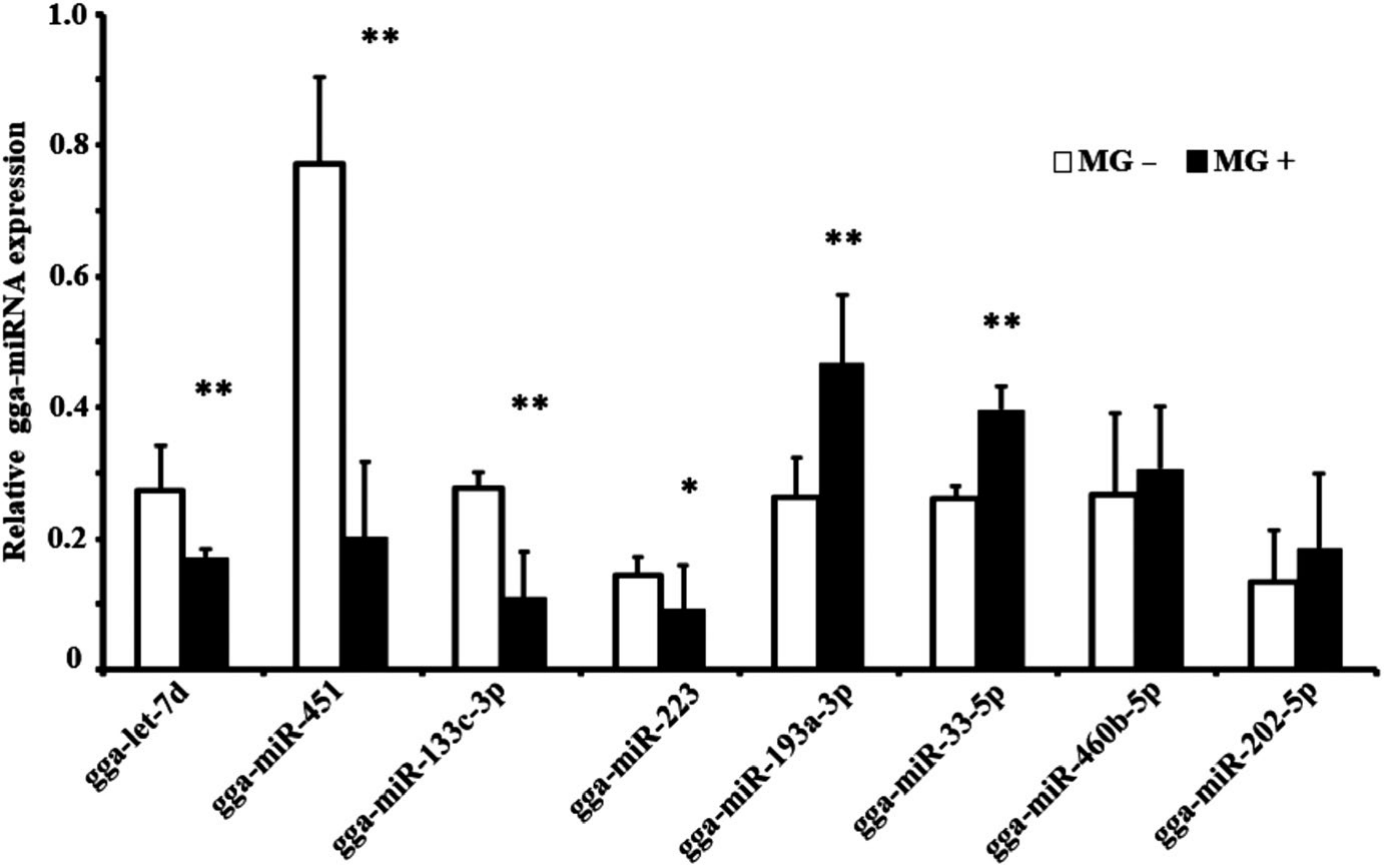
The validation of the selected exosome‐microRNA expression profile approach by using RT‐qPCR. 5s RNA was used as the internal control. Data represent means ± SDs of three independent experiments (two‐tailed Student's *t* test, **P < .01, *P < .05)

To validate the reliability of the sequencing results, a total of 8 differentially expressed exosomal miRNAs were selected for the poly (A) Plus real‐time PCR. RT‐qPCR results showed that the expression of gga‐let‐7d, gga‐miR‐451, gga‐miR‐133‐3p and gga‐miR‐223 was significantly down‐regulated in the MG‐infected group compared to the control groups. gga‐miR‐193a‐3p and gga‐miR‐33‐5p were up‐regulated in the MG‐infected group as compared with the control groups. In contrast, gga‐miR‐460b‐5p and gga‐miR‐202‐5p had no significant difference (Figure 5). These results indicated that exosomes derived from MG‐infected CP‐II cells in early infection may play a crucial role in the immune‐inflammatory process. Consistent with this hypothesis, gga‐miR‐451 is reportedly involved in the immune‐inflammatory process,^14^ suggesting that gga‐miR‐451 may play a key role in immune regulation during infection.

### 3.7 **gga‐miR‐451‐absent exosomes derived from MG‐infected** CP‐II** cells increase inflammatory cytokine production in DF‐1 cells**

**FIGURE 6:**
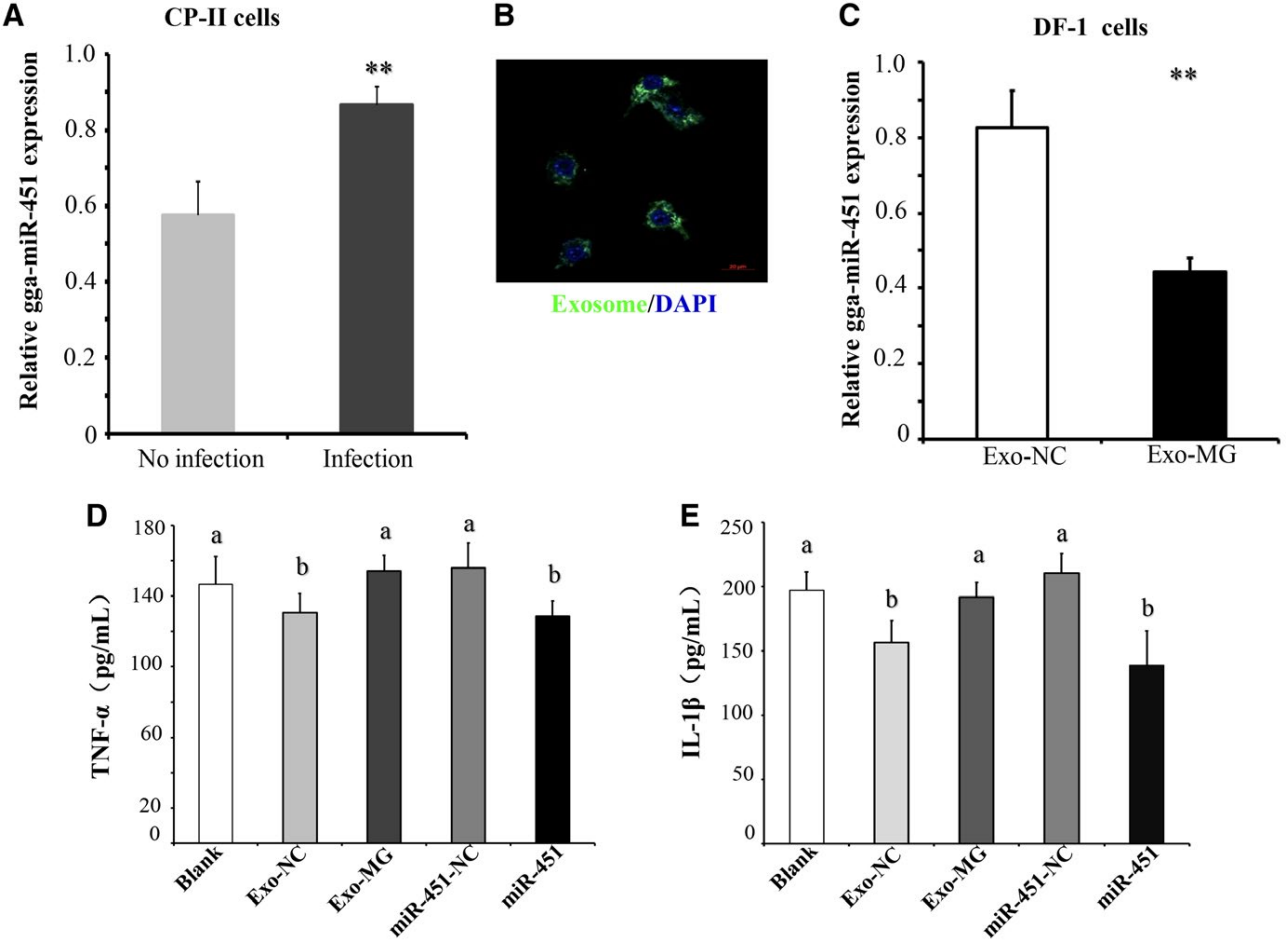
Chicken type II pneumocytes (CP‐II) cellular exosome‐derived gga‐miR‐451 increases inflammatory cytokine production in DF‐1 cells. A, Relative gga‐miR‐451 expression in MG‐HS‐infected CP‐II cells. B, CP‐II cell–derived exosomes were labelled with PKH67 (green), incubated with DF‐1 cells and then observed by confocal microscopy, cell nuclei (blue) and exosomes (green). C, Relative gga‐miR‐451 expression in DF‐1 cells cultured with CP‐II cell–derived exosomes (Exo‐MG or Exo‐NC). 5s RNA was used as the internal control. D‐E, The inflammatory cytokine level of TNF‐α and IL‐1β in DF‐1 cells at 48 h after treatment. Exo‐MG and Exo‐NC represent exosomes isolated from MG‐infected and non‐infected CP‐II cells, separately. A mock transfection was used as the Blank. Data represent three independent experiments and are presented as the means ± SDs (two‐tailed Student's *t* test, **P < .01, different lowercase letters represent *P* < .01)

Previous data showed that gga‐miR‐451 expression is significantly elevated during MG infection in DF‐1 cells. To verify this result in chicken lung epithelial cells, CP‐II cells were infected with MG‐HS. At 48 hours after infection, RT‐qPCR result showed that gga‐miR‐451 level was significantly increased in the CP‐II cells (Figure 6A). Interestingly, gga‐miR‐451 level was significantly decreased in the exosomes derived from MG‐infected CP‐II cells (Figure 5). To reveal whether gga‐miR‐451‐absent exosomes derived from MG‐infected CP‐II cells are involved in the regulation of inflammation to commute between lung epithelial cells and systemic fibroblast, CP‐II cell**–**derived exosomes were labelled with PKH67. They were then incubated with DF‐1 cells for 6 hours at 37°C and observed by confocal microscopy, which revealed the incorporation of exosomes into DF‐1 cells (Figure 6B). Next, DF‐1 cells were incubated with exosome derived from MG‐infected CP‐II cells (Exo‐MG) and exosome derived from non‐infected CP‐II cells (Exo‐NC). At 36h after treatment, RT‐qPCR result showed that gga‐miR‐451 was significantly decreased in Exo‐MG as compared with the control group (Figure 6C). Recently, we reported that gga‐miR‐451 decreases the inflammatory cytokine production, including TNF‐α and IL‐1β.^14^ We hypothesized that CP‐II cellular exosome‐derived gga‐miR‐451 can affect inflammatory cytokine levels in DF‐1 cells. To test this hypothesis, DF‐1 cells were separately incubated with Exo‐MG and Exo‐NC (10 μg/mL), transfected with 5 pmol of miR‐451 mimics (miR‐451) or a non‐specific control siRNA (miR‐451‐NC). Transfection efficiencies of exosomes and siRNA were above 80% (Figure 6B). In addition, a mock transfection was used as a blank control (Blank). TNF‐α and IL‐1β protein levels were detected by ELISA at 36 hours after treatment. As shown in Figure 6D,E, the Exo‐MG could notably increase TNF‐α and IL‐1β protein levels in DF‐1 cells compared with Exo‐NC**.** These results indicate that gga‐miR‐451 is selectively unloaded in MG‐infected exosomes and may play a crucial role in the immune‐inflammatory process during MG infection.

### 3.8 CP‐II **cellular exosome‐derived gga‐miR‐451 regulates YWHAZ expression in DF‐1 cells**

**FIGURE 7:**
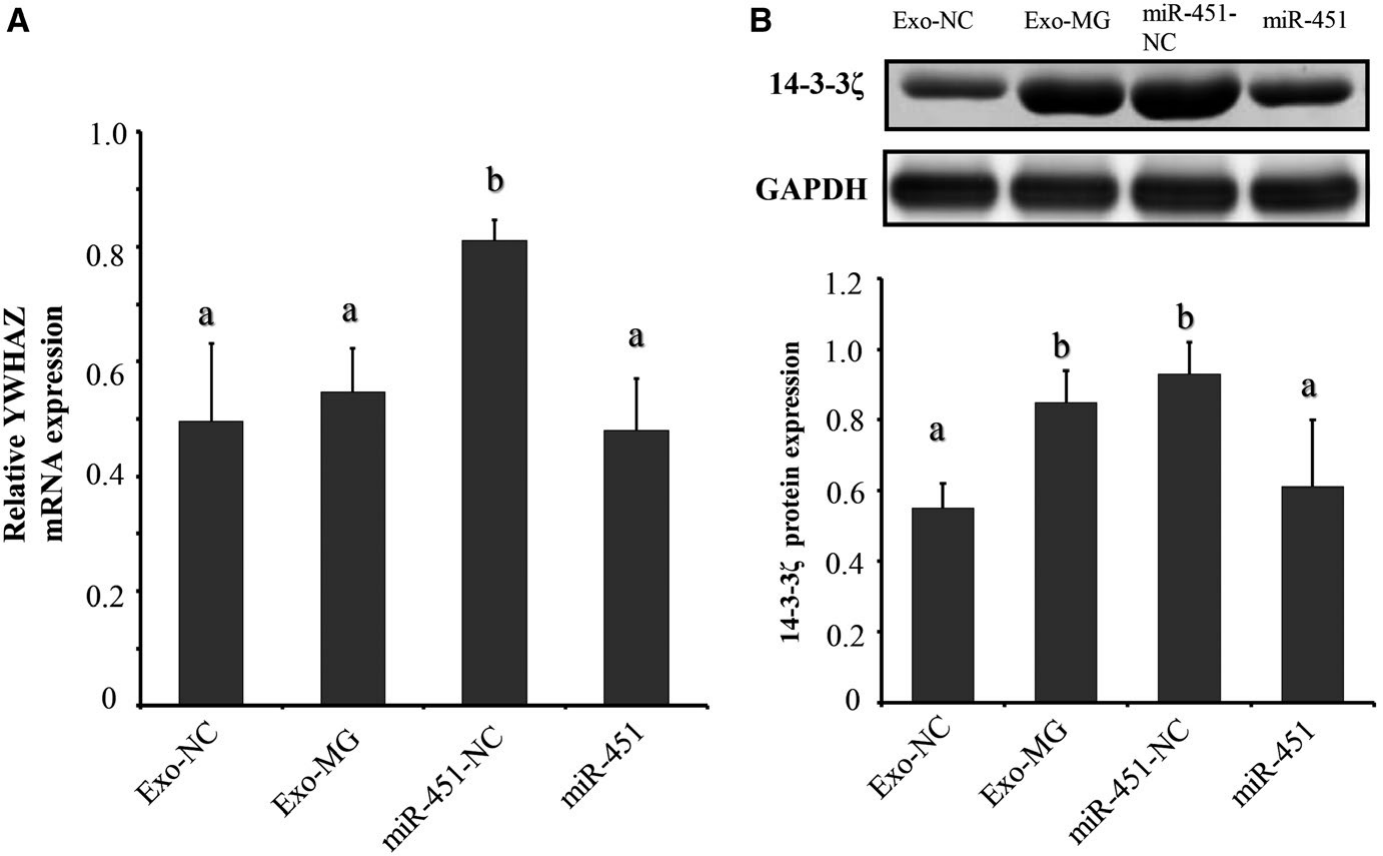
Chicken type II pneumocytes (CP‐II) cellular exosome‐derived gga‐miR‐451 regulates YWHAZ expression. A, *YWHAZ* mRNA expression was detected by RT‐qPCR. B, Western blot analyses of 14‐3‐3ζ expression level in different treatment groups. GAPDH was used as the internal control. Exo‐MG and Exo‐NC represent exosomes isolated from MG‐infected and non‐infected CP‐II cells, respectively. Data represent three independent experiments and are presented as the mean ± SDs (two‐tailed Student's *t* test, different lowercase letters represent *P* < .01)

YWHAZ is an established target gene of gga‐miR‐451. To confirm that exosomal gga‐miR‐451 can affect TNF‐α and IL‐1β expression through regulating YWHAZ, we separately cultured DF‐1 cells with Exo‐MG or Exo‐NC derived from CP‐II cells (10 μg/mL), transfected with 5 pmol of miR‐451 mimics (miR‐451) or negative control (miR‐451‐NC). Transfection efficiencies of exosomes and siRNA were above 80% (Figure 6B). As expected, gga‐miR‐451 significantly decreased the mRNA and protein levels of YWHAZ as compared with NC, and Exo‐MG significantly increased the protein levels of YWHAZ as compared with Exo‐NC, using RT‐qPCR and Western blot (Figure 7A,B). These data suggest that exosomes derived from CP‐II cells may deliver less gga‐miR‐451 to DF‐1 cells to induce inflammatory cytokines by targeting YWHAZ.

## 4 DISCUSSION

*Mycoplasma* provokes rock‐ribbed inflammation in organisms, resulting in the production of various pro‐inflammatory cytokines in many different cell types.^35^ MG is well known as a common pathogen that can cause chronic respiratory inflammation in poultry worldwide. After MG‐HS enters the chicken's body, it adheres to the respiratory tract mucous membrane and the epithelial cells through adhesin proteins (pMGA1.2), causing damage to the respiratory tract tissue. MG‐HS then multiplies locally to induce lung cell degeneration, causing the cilia to stop moving and falling off, thereby infecting the host respiratory tract and establishing a chronic infection.^36^ After passing through the respiratory mucosal barrier, MG enters the blood circulation, spreading to tissues and organs throughout the body. Therefore, the damage to the cells of the respiratory tract and the resulting lesions are the leading link in the pathogenesis of this disease.^37^ In this study, CP‐II cells as donor cells were isolated and infected with MG‐HS. Although CP‐II cells are not immune and inflammatory cells, they play an important role in regulating lung immune defence.^22^, ^38^

Exosome shuttling is a process with a wide range of important regulatory functions. They harbour a communicative function that involves an exchange of membranes or membrane proteins between cells and exosomes.^39^ Exosomes have been reported to play a significant role in inflammation processes due to their capability to carry inflammatory modulators such as miRNA and proteins which can act on both neighbouring and distal cells and affect cellular function.^40^ The special membrane structure of exosomes reinforces the stability of the miRNA and enhances its potential as a novel predictive marker in disease diagnosis. In addition, many functional experiments have demonstrated that exosomes affect lung injury and inflammation via exosomal miRNAs.^41^, ^42^ We sought to investigate the miRNA expression profiles of exosomes derived from MG‐infected CP‐II cells using Illumina HiSeq 2500 high‐throughput sequencing (miRNA‐seq) and the role of specific exosomal miRNA. The findings provide new insight into immunological variations during MG infection.

In this study, we identified 30 (*P* < .05, fold change > 1) significant DEGs between the MG‐infected and control groups, including 9 up‐regulated and 21 down‐regulated, which can be chosen as the target miRNA to understand the possible molecular mechanism of exosomal miRNAs derived from MG‐infected CP‐II cells. The levels of urinary exosomal miR‐193a were significantly higher in children with primary focal segmental glomerulosclerosis than those in children with minimal change disease.^43^ In polymicrobial sepsis, exosomal miR‐223 contributes to mesenchymal stem cell–elicited cardioprotection.^44^ In our study, the expression of exosomal miR‐193a and miR‐223 was significantly differentially expressed in the exosomes derived from MG‐infected CP‐II cells compared to the control group. miRNAs play a key role in cell signalling pathways by regulating transcription factors and gene expression. Meanwhile, Gene Ontology (GO) analysis showed that the target genes of differentially expressed miRNAs (DEGs) mainly originates from a class of membrane‐associated or organelle proteins (‘membrane’, ‘intracellular membrane‐bounded organelle’, ‘intracellular part and ‘cytoplasm’) with small molecule binding and enzyme catalysis functions (‘organic cyclic compound binding’, ‘nucleic acid binding’, ‘protein binding’ and ‘catalytic activity’). They are involved in the regulation of cellular process, metabolism and protein modification (‘cellular process’, ‘metabolic process regulation of response to stimulus’ and ‘biological regulation’). The KEGG pathways are widely used to predict the gene function and genome information, which is helpful for us to study the metabolism function, genetic information processing, cellular processes and diseases.^45^ The target genes of DEGs in this study were plotted to the reference pathways in KEGG, the most significant KEGG pathways were apoptosis, cell cycle, focal adhesion, tight junction, influenza A, Toll‐like receptor signalling pathway, mTOR signalling pathway, Wnt signalling pathway, cytokine‐cytokine receptor interaction and MAPK signalling pathway. These pathways reflect the physiological processes of MG infection and the potential regulatory mechanisms underlying them. Our previous data demonstrated that Toll‐like receptor signalling pathway, Wnt signalling pathway and MAPK signalling pathway play a crucial role in MG‐HS infection and implicated in chronic inflammatory reaction,^8^, ^34^ and DF‐1 cell cycle and apoptosis are also modulated during MG‐HS infection.^14^ Up‐regulation of gga‐miR‐451 significantly induced the MG‐infected DF‐1 cell cycle arrest and promoted apoptosis.^14^ It has been well documented in the literature that miR‐100‐5p‐abundant exosomes derived from infrapatellar fat pad mesenchymal stem cells (MSCs) protect articular cartilage and ameliorate gait abnormalities via inhibition of mTOR in osteoarthritis.^46^ Together, the results indicate that exosomal miRNAs derived from MG‐infected CP‐II cells play a complex role in signalling pathways and regulating the inflammatory reactions, which provides the theoretical basis, direction for the prevention and treatment of *Mycoplasma* infection in poultry.

Our results showed that the concentration of exosomes derived from MG‐infected CP‐II cells was increased as non‐infected cells (Figure 1E; Table S3), which was similar to the results of previous reports in other diseases.^33^, ^47^ Moreover, respiratory secretions of patients with cystic fibrosis are often found with protease‐rich exosomes that are produced by gram‐negative bacteria that colonize the airways. LPS stimulation of respiratory epithelial cells also could lead to release of exosomes in human.^48^ Among the 8 validated exosomal miRNAs, 6 miRNAs were confirmed by RT‐qPCR and deep sequencing to share similar trends in the MG‐infected and control groups. Although the validated miRNAs did not cover all the DEGs in the two libraries, the normalized profiling data and the RT‐qPCR results indicated the reliability of our analysis results regarding the exosomal miRNA distribution in the two groups. Interestingly, there are only 5 differentially expressed miRNAs (gga‐miR‐365‐3p, gga‐miR‐449b‐5p, gga‐miR‐133c‐3p, gga‐miR‐451 and gga‐miR‐223) which are consistent between the results of this study and our previous results of miRNA sequencing of MG‐infected chicken lungs at 3 and 10 days after infection.^8^ This study demonstrated that gga‐miR‐451 level was significantly increased in MG‐infected CP‐II cells, which was shared by the results of our previous report in DF‐1 cells (Figure 6A),^14^ while the exosomal gga‐miR‐451 was significantly decreased in the exosomes derived from MG‐infected CP‐II cells compared to the control group (Figure 5). These results suggest that gga‐miR‐451 was selected unloaded in exosomes derived from MG‐infected CP‐II cells. Under certain stimulating conditions, the epithelial cell–secreted exosomes are capable of causing or aggravating pulmonary inflammation. In this study, the Exo‐MG could notably increase TNF‐α and IL‐1β protein levels in DF‐1 cells compared to Exo‐NC (Figure 6D,E). It is reported that exosomes, like vesicles released by adipose tissue of obese mice, are taken up by mononuclear cells inducing their differentiation into active macrophages with an increased release of TNF‐α and IL‐6.^49^ The blood microparticle treatment led to remarkable increases in myeloperoxidase (MPO), TNF‐α, IL‐1β and IL‐10 productions in bronchoalveolar lavage fluid and plasma.^50^ Interestingly, the Exo‐NC decreased TNF‐α and IL‐1β expressions compared to Blank (Figure 6D,E); it may be that gga‐miR‐451 in exosomes had negative effect on TNF‐α and IL‐1β protein levels. We confirmed previously that gga‐miR‐451 expression significantly decreased TNF‐α and IL‐1β secretions in DF‐1 cells.^14^ These were consistent with results in this study. It is reasonable to believe that gga‐miR‐451‐absent exosomes derived from MG‐infected CP‐II cells increase inflammatory cytokine production, including TNF‐α and IL‐1β.

Studies on YWHAZ protein (14‐3‐3ζ) in multiple systems have found that it is a target gene and is negatively regulated by miR‐451 in mammals and chickens.^14^, ^51^ We also found that gga‐miR‐451‐absent exosomes can regulate the expression of YWHAZ protein in DF‐1 cells (Figure 7 **)**. Our previous data showed that YWHAZ protein in the miR‐451‐NC group is no significantly different from the mock transfection group (Blank).^14^ These results suggest that the exosomes derived from CP‐II cells may deliver less gga‐miR‐451 to DF‐1 cells to induce inflammatory cytokines by targeting YWHAZ and increasing its expression. In addition to gga‐miR‐451, other exosomal gga‐miRNAs may also regulate the immunity processes in MG infection. For example, exosomal miR‐223 targets the transcription factor STAT5 and is implicated in inflammatory reactions in multiple sclerosis.^52^ The serum exosome‐derived miR‐193 was reported to be a potential Alzheimer's disease biomarker.^53^ However, the molecular mechanism of these exosomal miRNAs that shuttle and regulate chronic respiratory inflammation needs to be further studied.

The present study provides evidence to prove that the mode of intercellular communication between chicken cells is mediated by exosomes. About 30 exosome‐microRNAs derived from CP‐II are significantly differentially expressed following MG infection. The changed exosome‐microRNAs target genes involve in cell cycle, Toll‐like receptor signalling pathway, MAPK signalling pathway, etc Additionally, exosomes from MG‐infected CP‐II cells alter the dynamics of the DF‐1 cells and may contribute to pathology of the MG infection via exosomal gga‐miR‐451 targeting YWHAZ involving in inflammation. These findings provide a new mechanism for the pathophysiology of MG infection and suggest exosomal gga‐miR‐451 as a new therapeutic target for the treatment of CRD.

## 5 ETHICS APPROVAL AND CONSENT TO PARTICIPATE

Our experimental protocols for chicken embryo treatment were approved by the Institutional Animal Care and Use Committee of Huazhong Agricultural University. The procedures were carried out in accordance with the approved guidelines.

## CONFLICT OF INTEREST

All authors listed declare that they have no competing interests.

## AUTHORS' CONTRIBUTIONS

Yabo Zhao and Yali Fu performed the experiments; Mengyun Zou and Yingfei Sun analysed the data; Xun Yin and Lumeng Niu performed the total RNA isolation and cDNA preparation; Yanzhang Gong and Xiuli Peng conceived and designed the study and helped write the manuscript. All authors have read and approved the final manuscript for publication.

## Supporting information

FigS1

TableS1

TableS2

TableS3

TableS4

TableS5

TableS6

## Data Availability

All data generated or analysed during this study are included in this published article [and its supplementary information files].
