# Supplementary material for: Analysis of deep sequencing exosome‐microRNA expression profile derived from CP‐II reveals potential role of gga‐miRNA‐451 in inflammation
Source: J Cell Mol Med. 2020 Apr 19;24(11):6178–90. doi: 10.1111/jcmm.15244 (PMC7294135; doi:10.1111/jcmm.15244)

**Supplementary Figure 1: The read counts of each novel mature miRNAs (G: MG infection; Z-non-infection).**


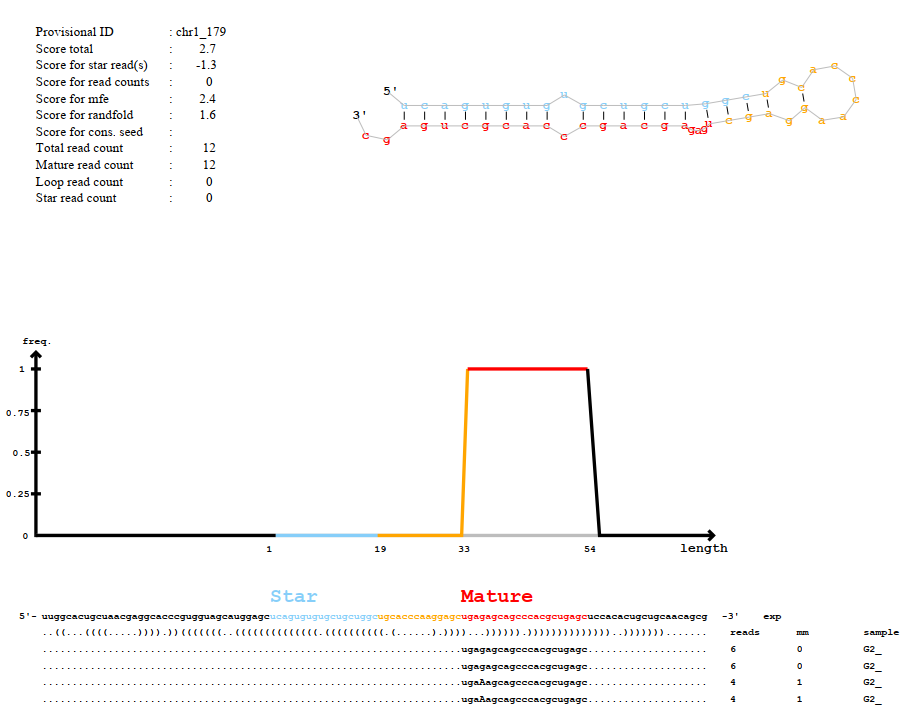


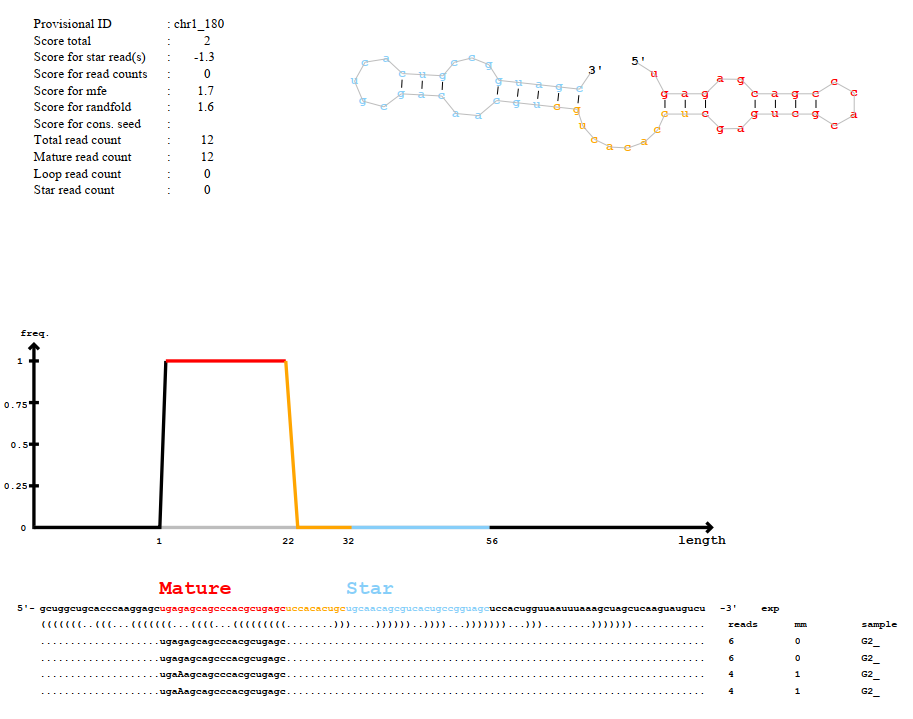


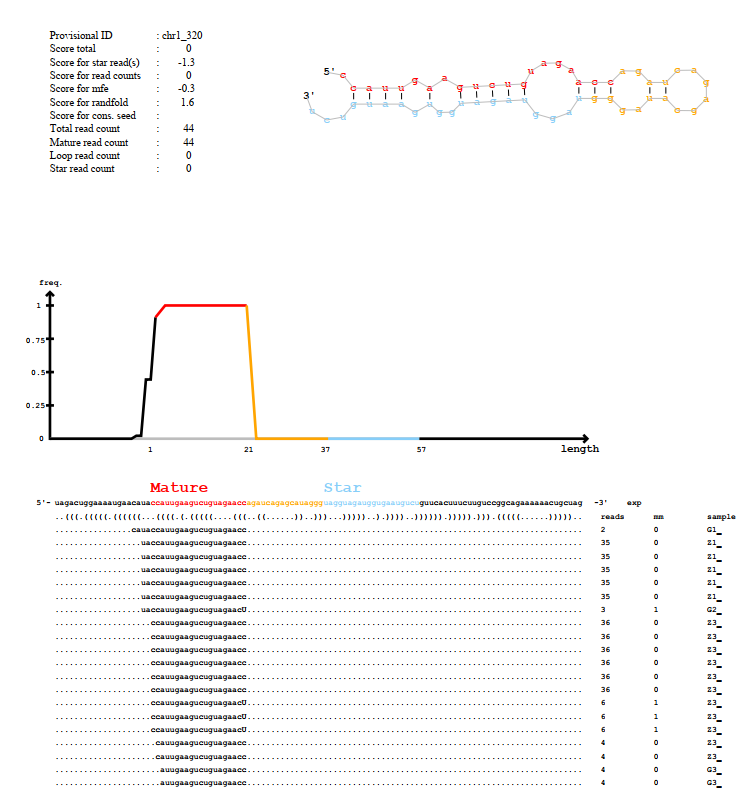


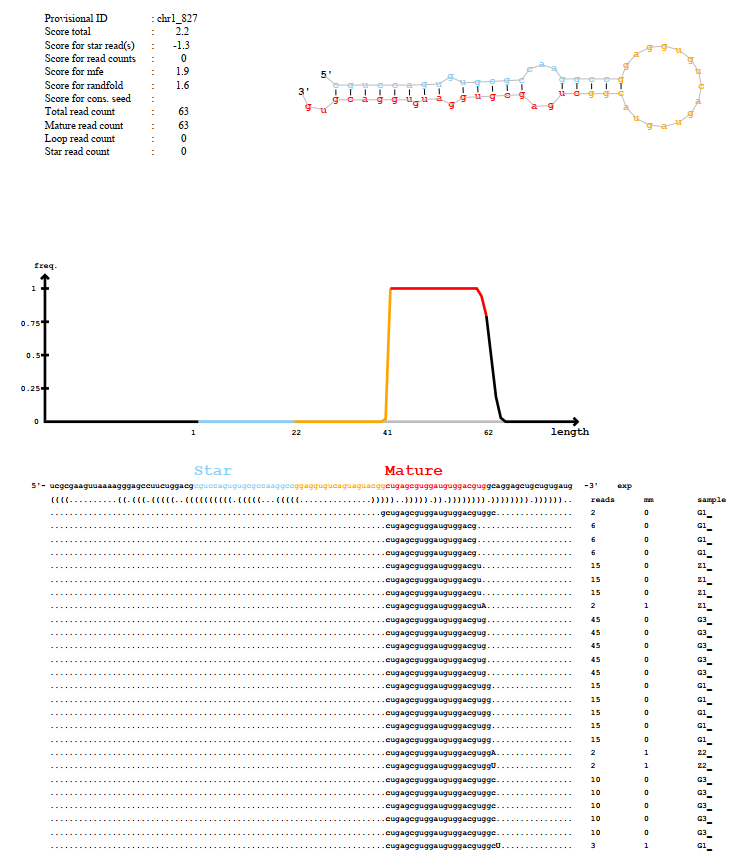


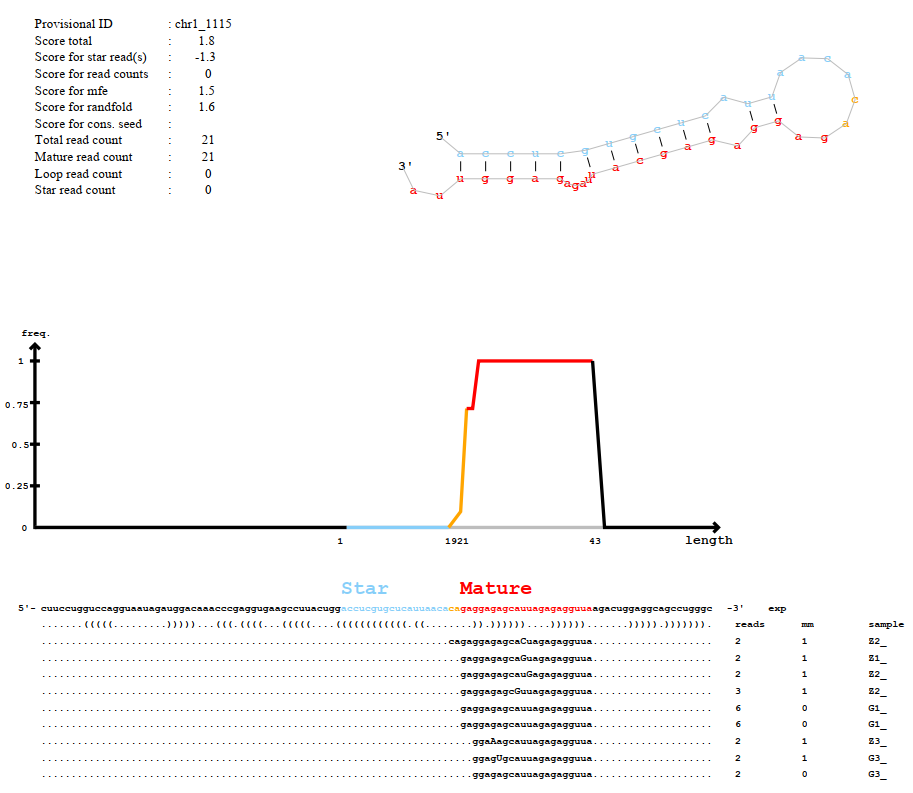


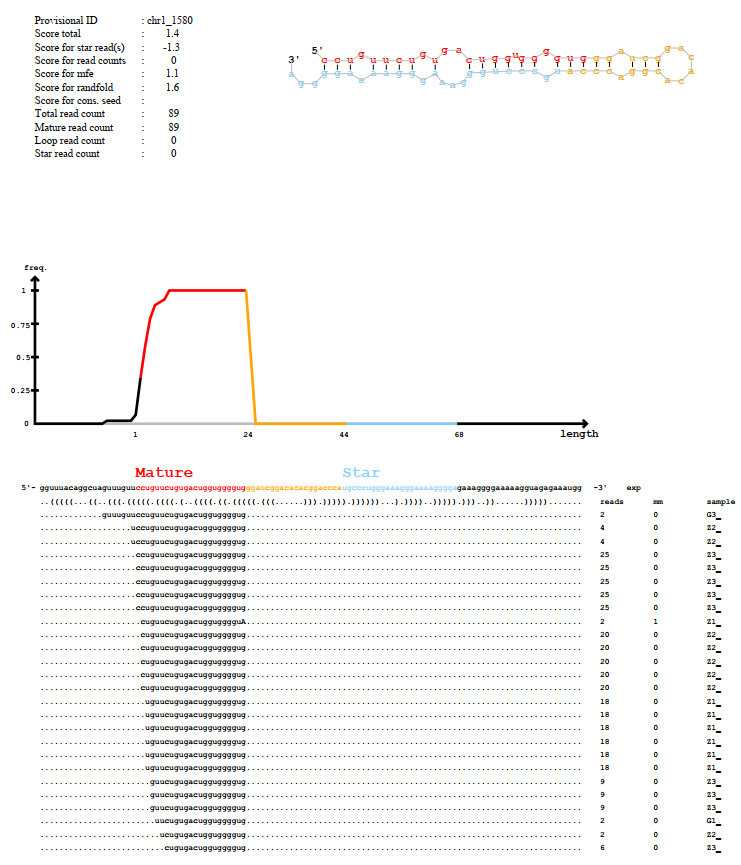


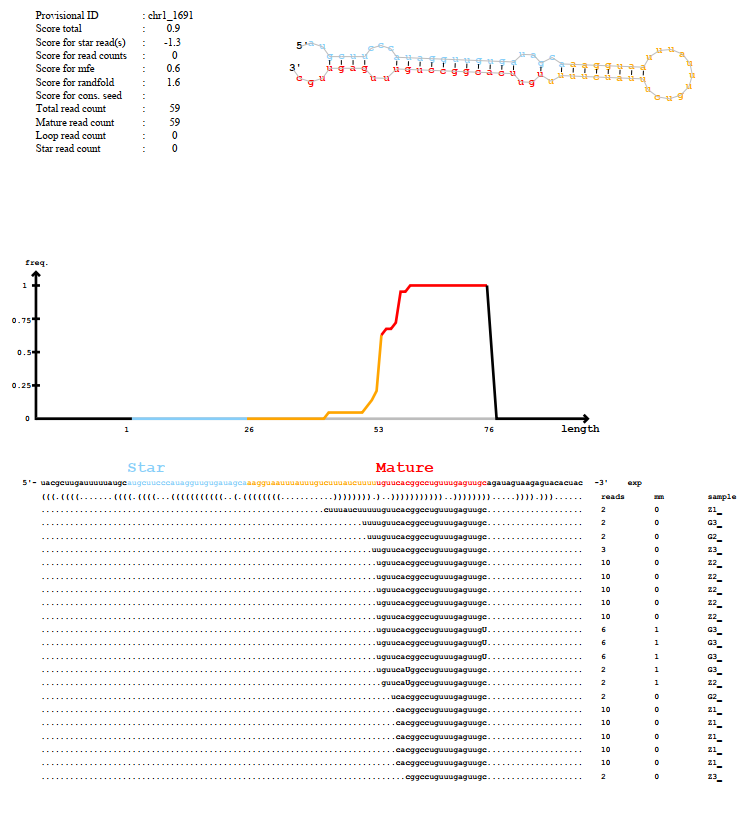


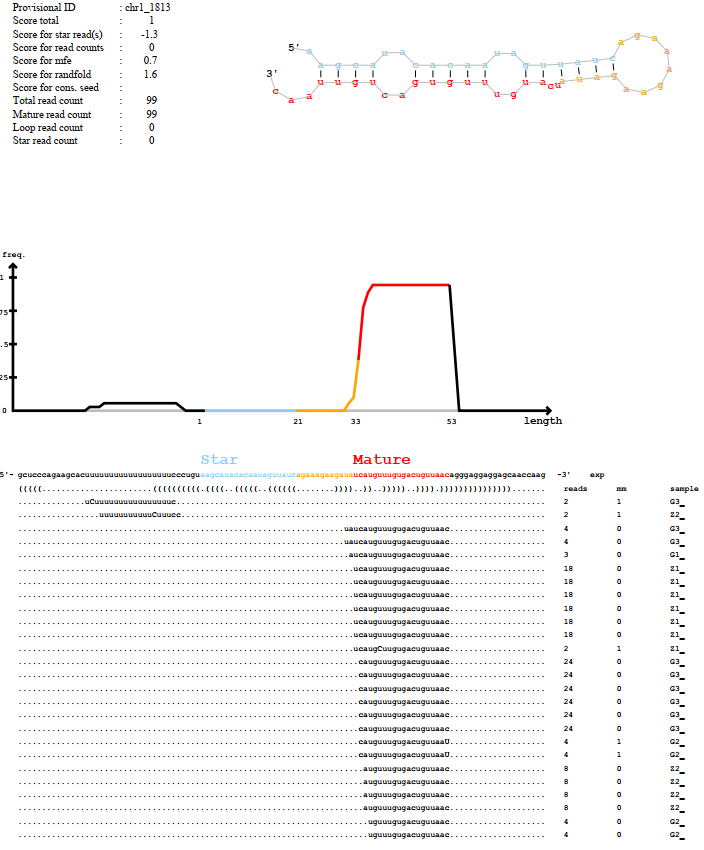


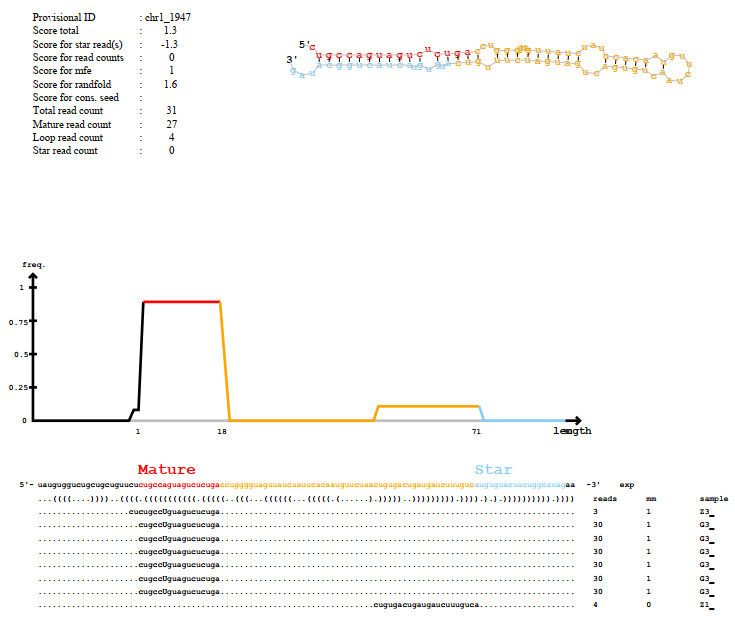


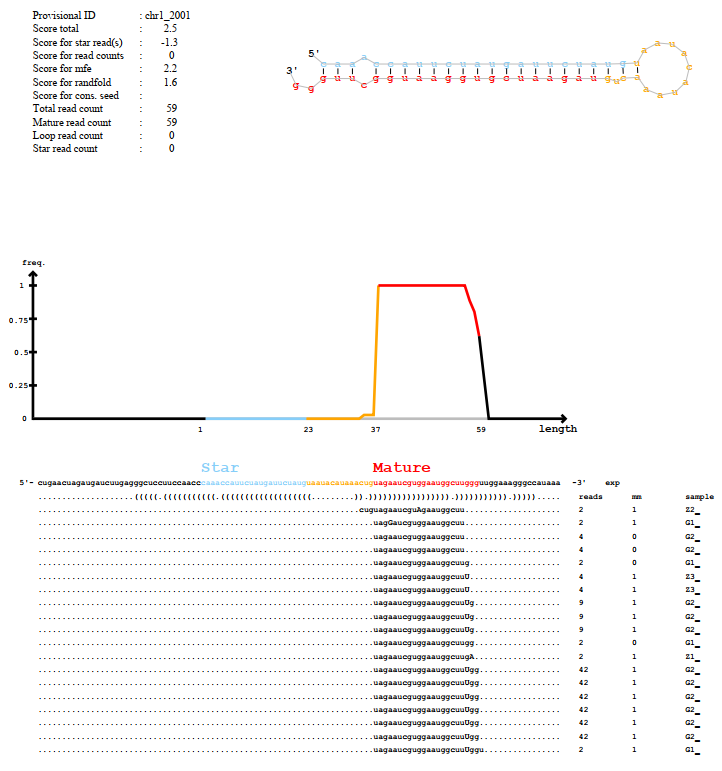


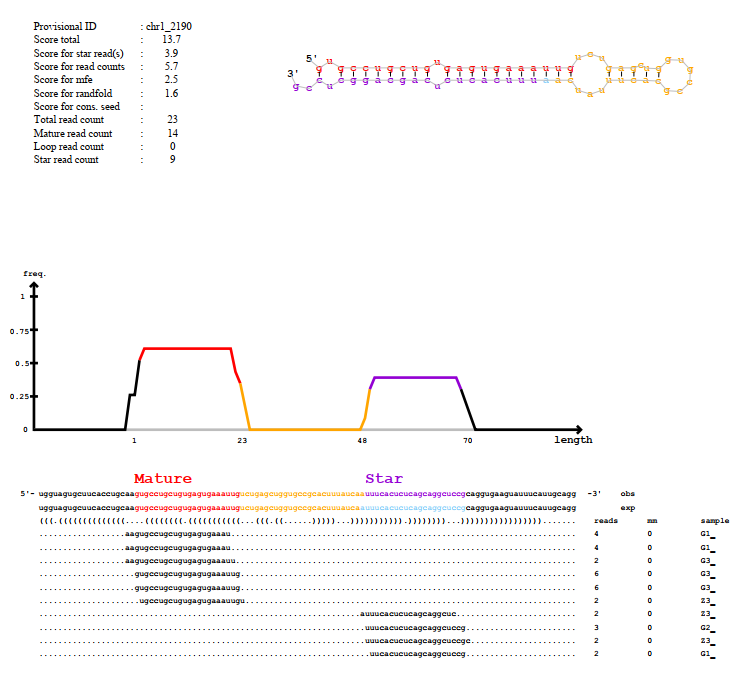


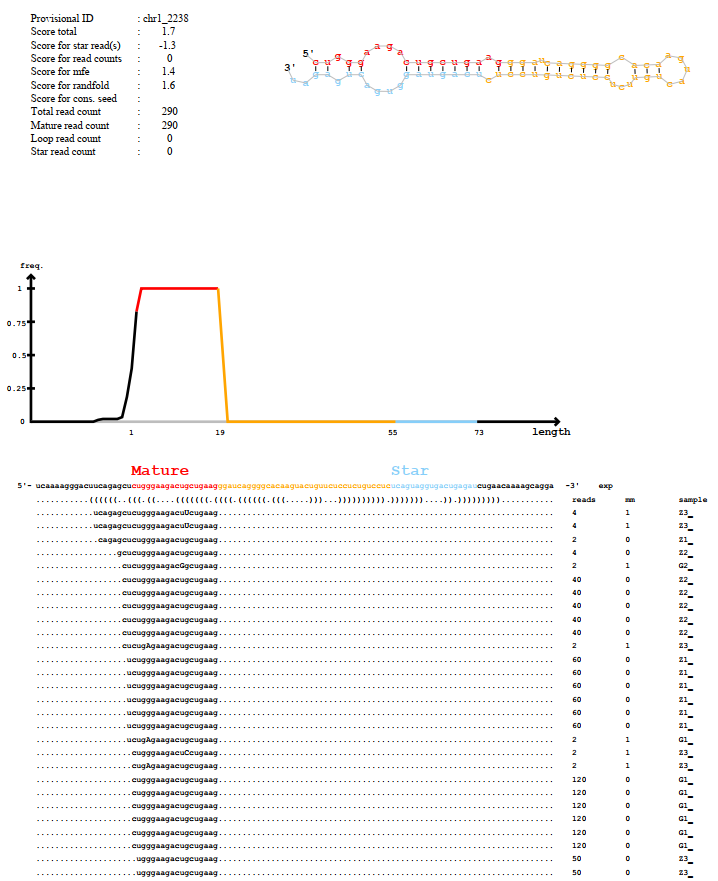


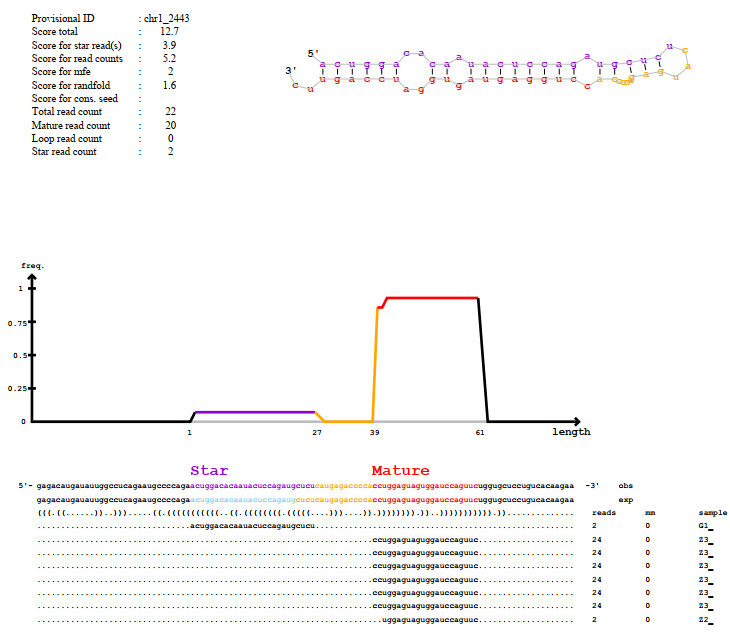


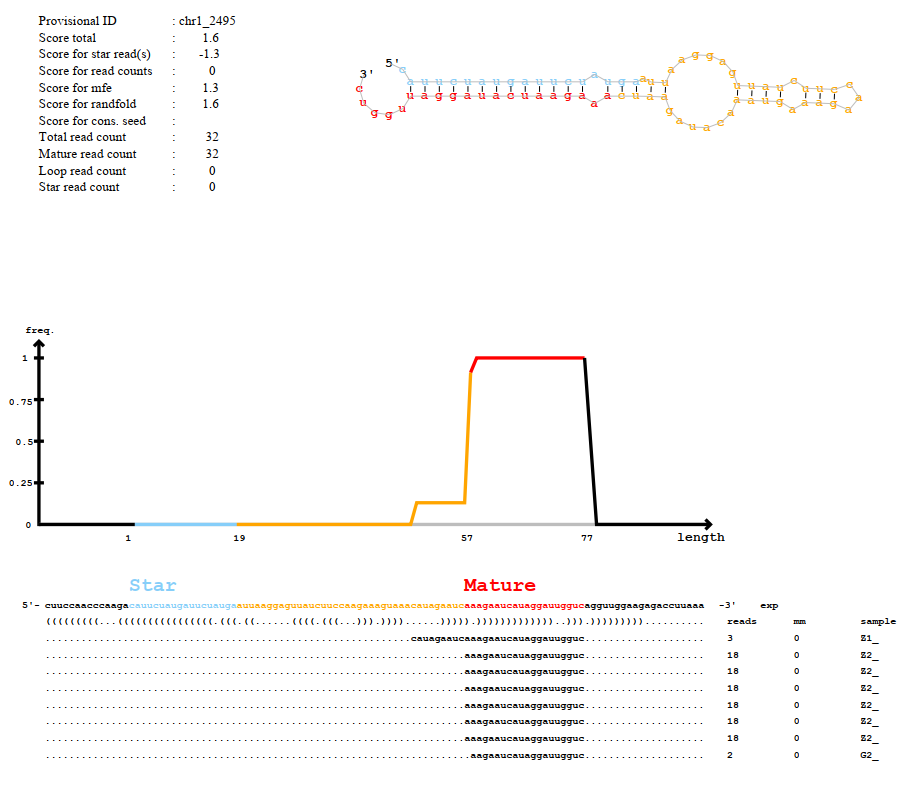


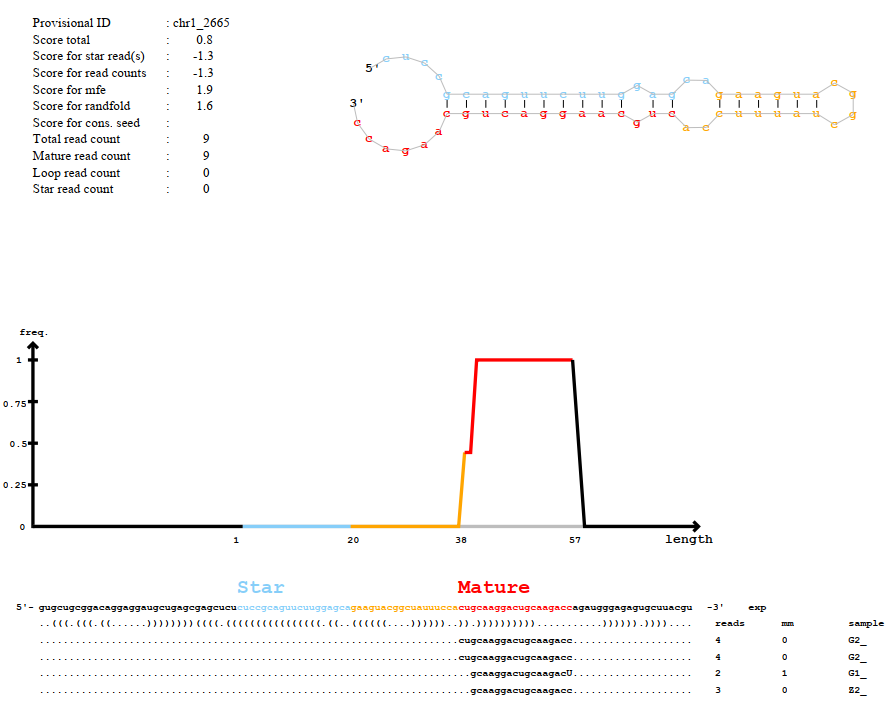


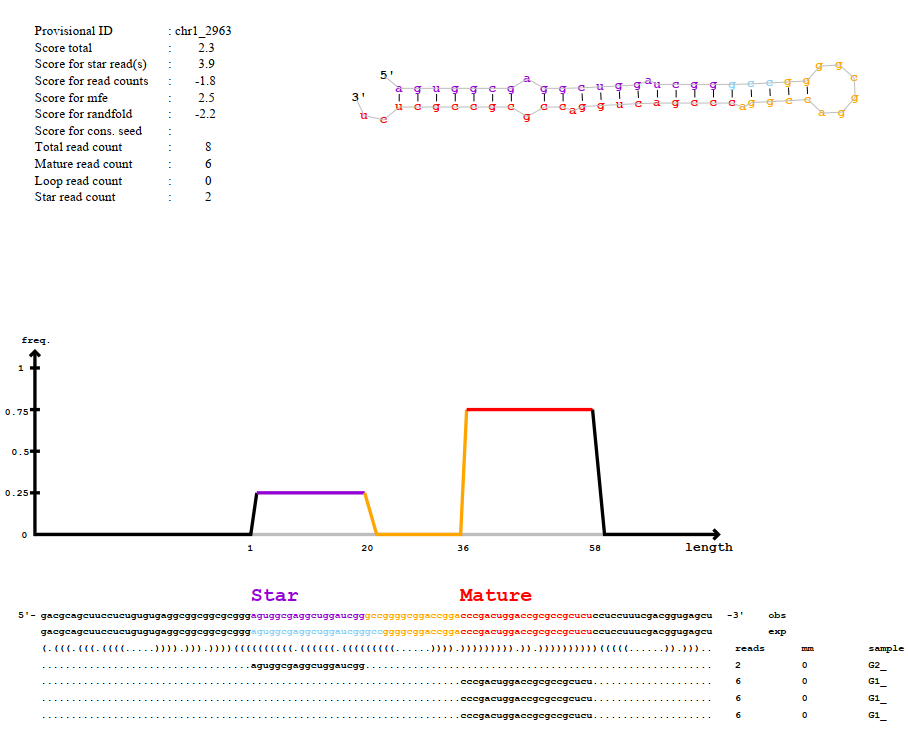

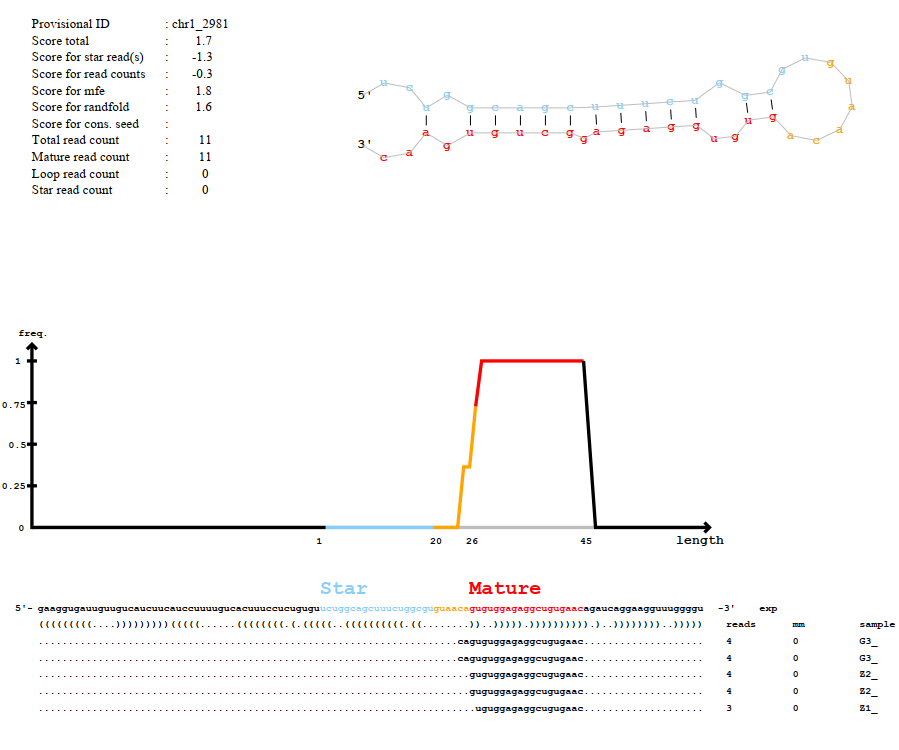

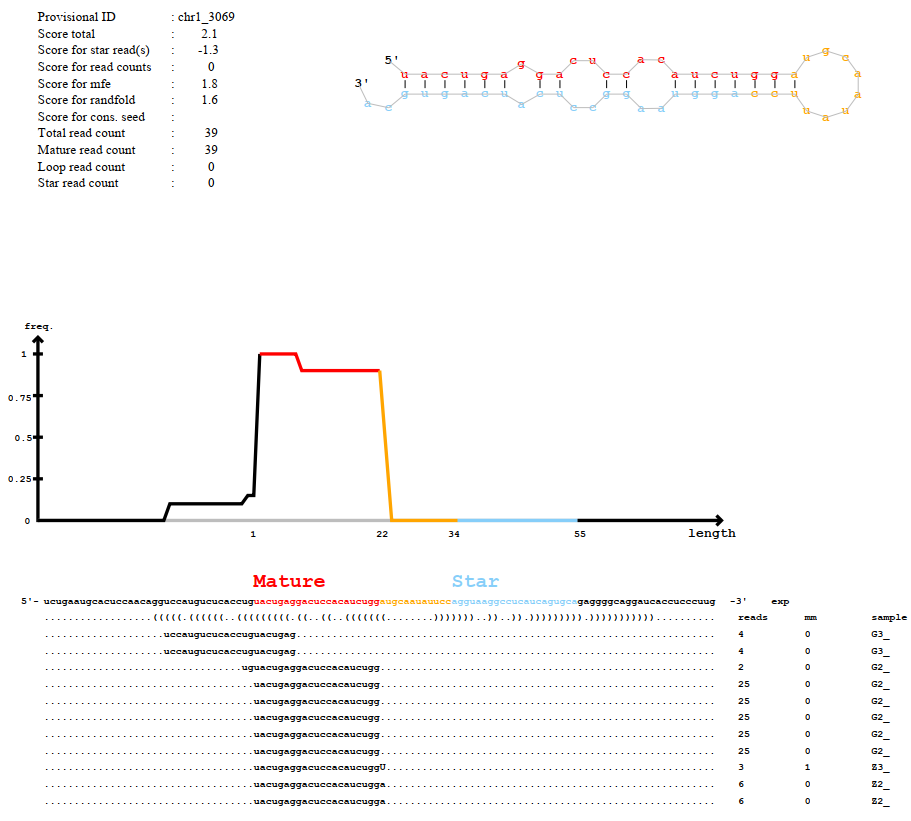

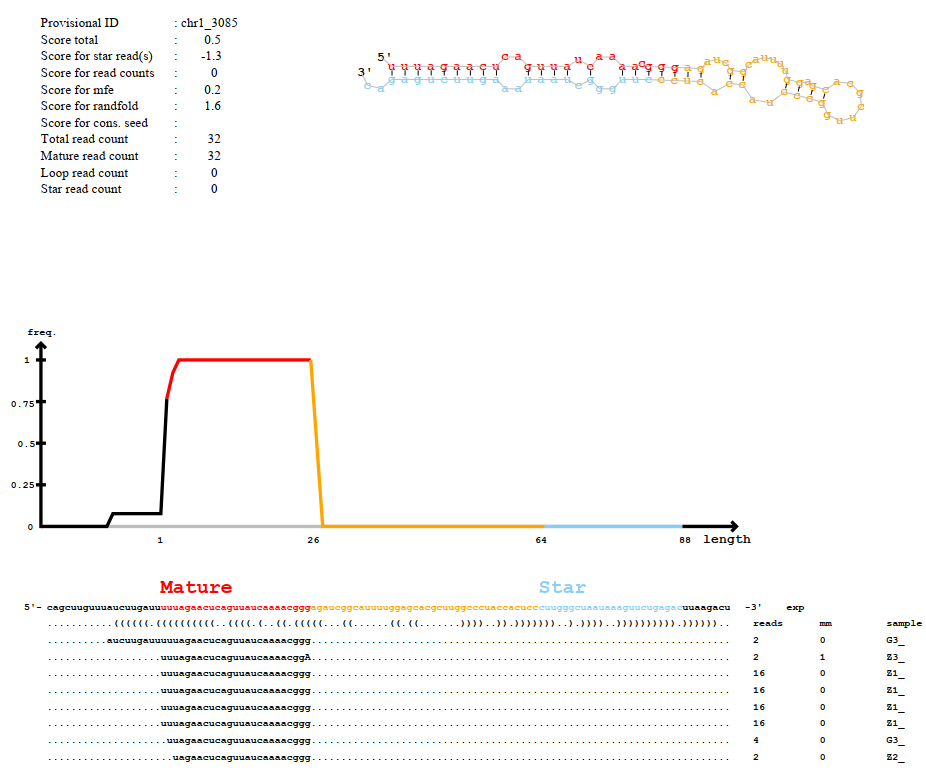

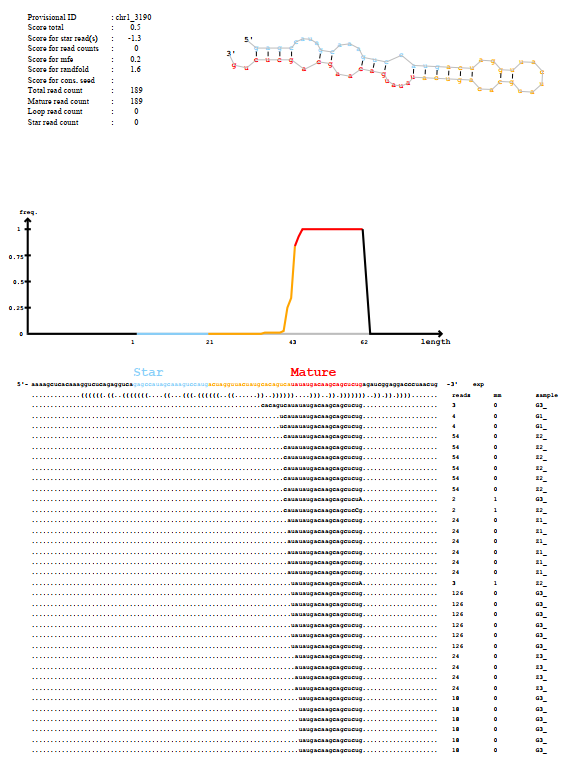

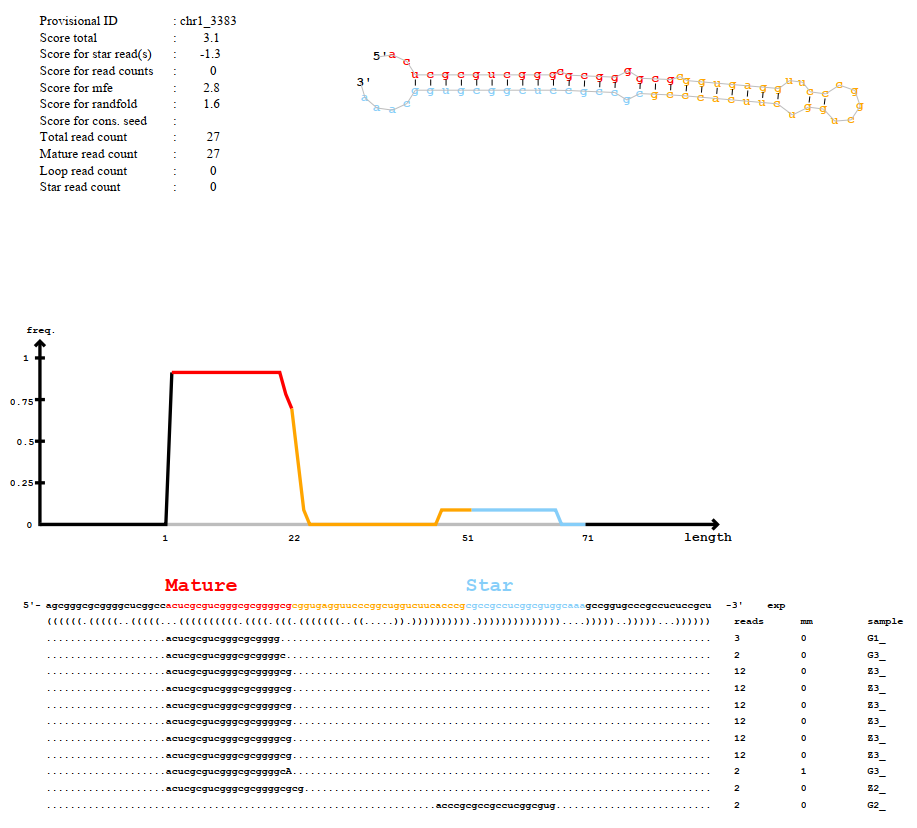

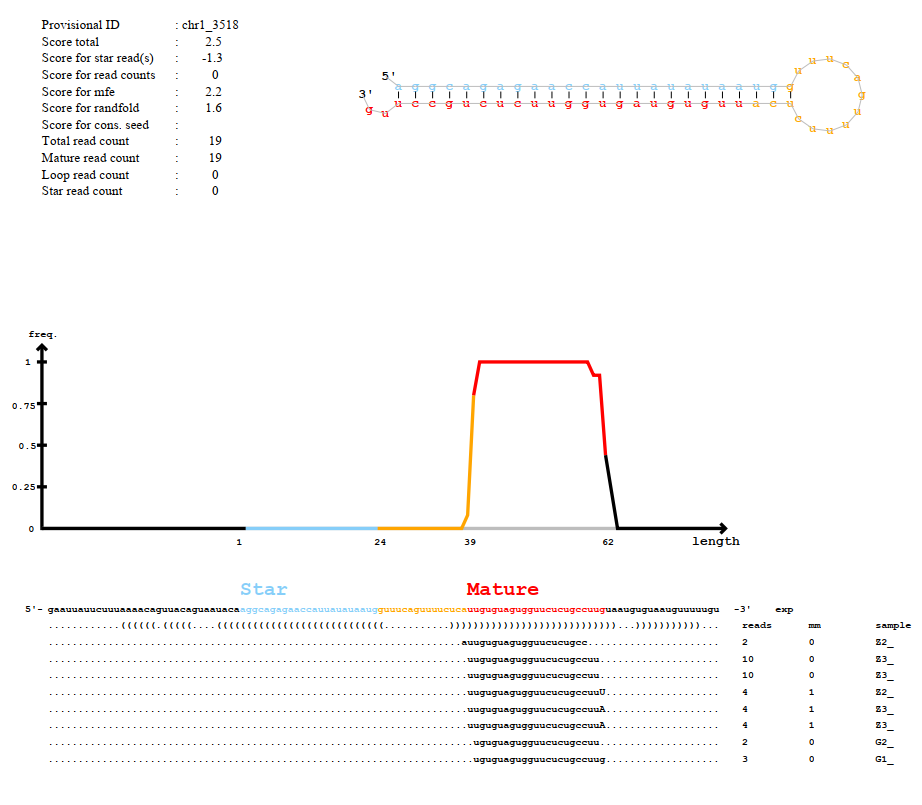

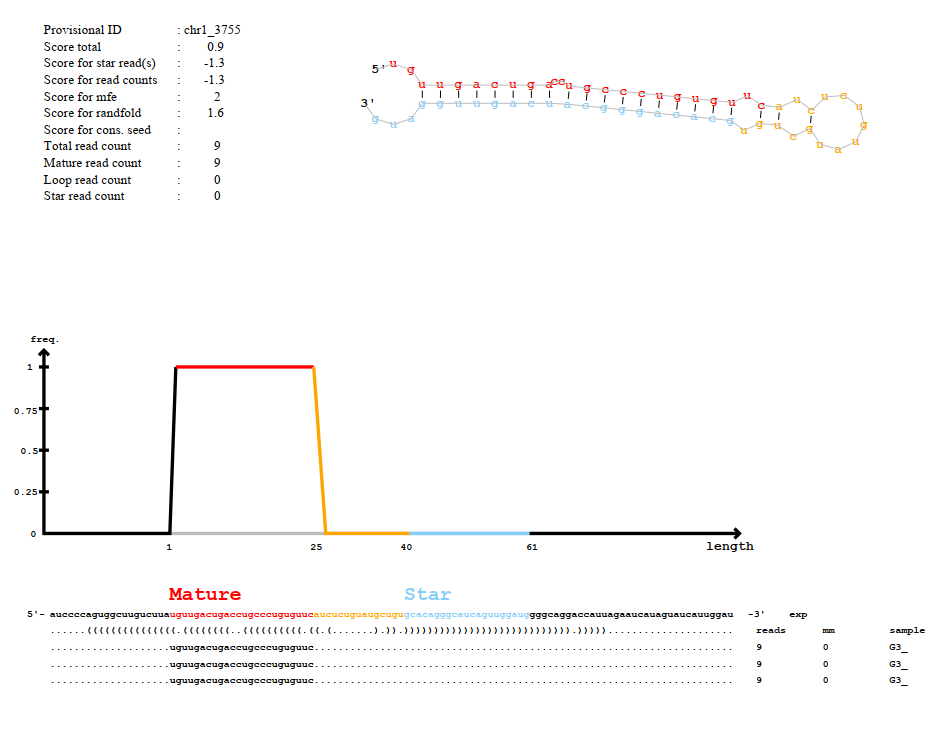

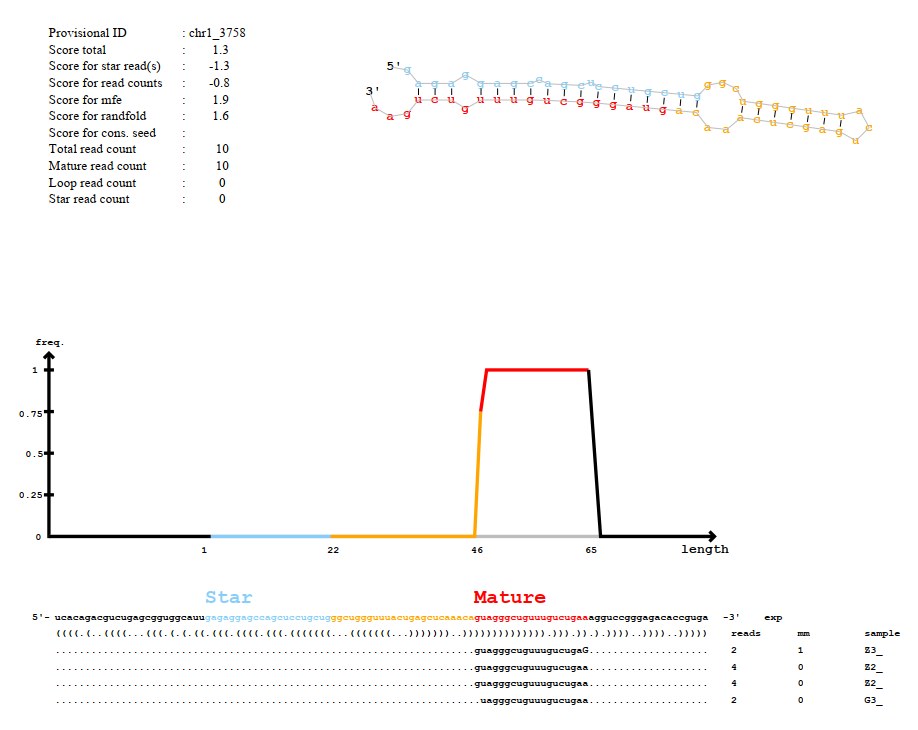

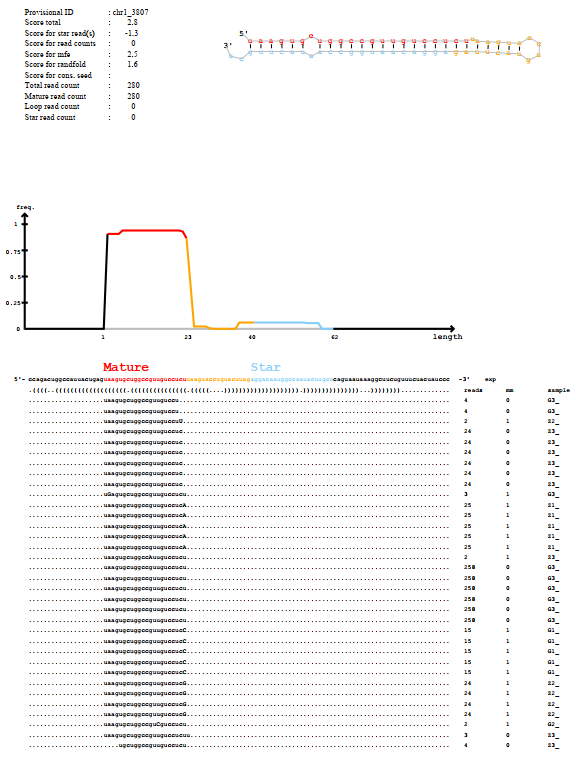

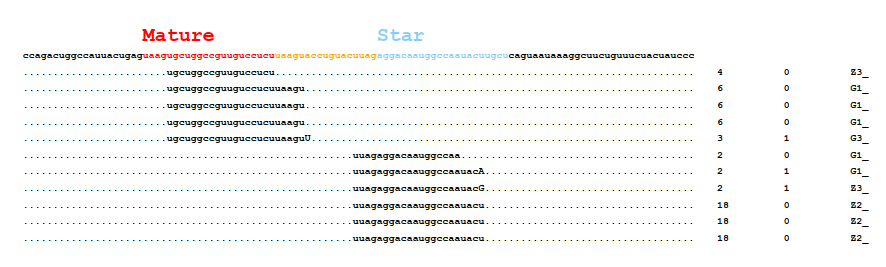

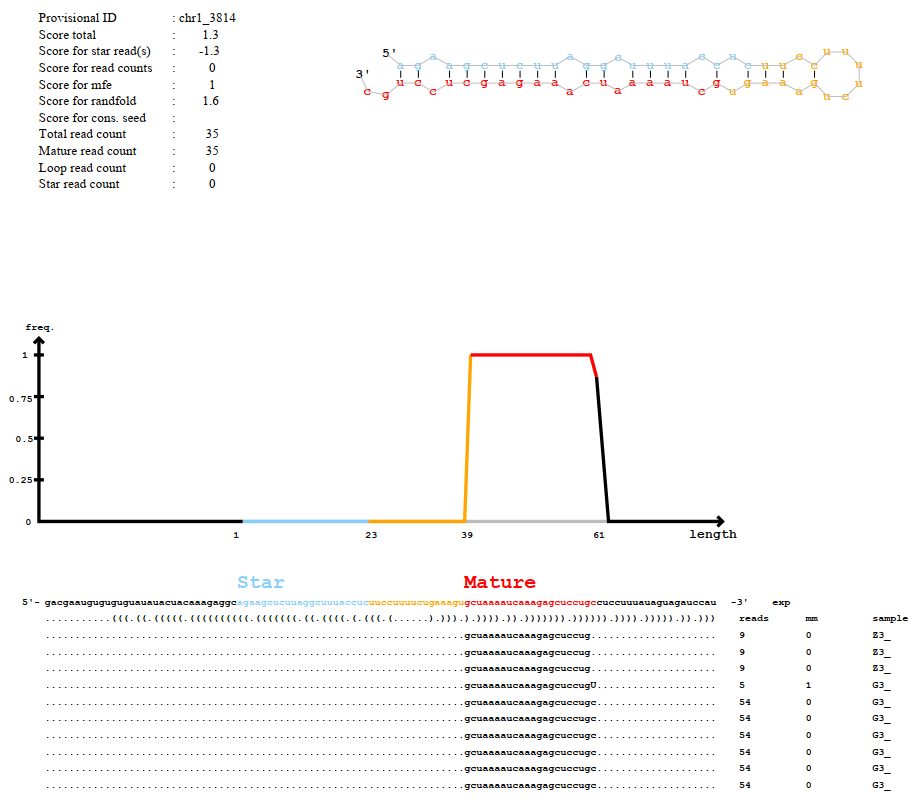

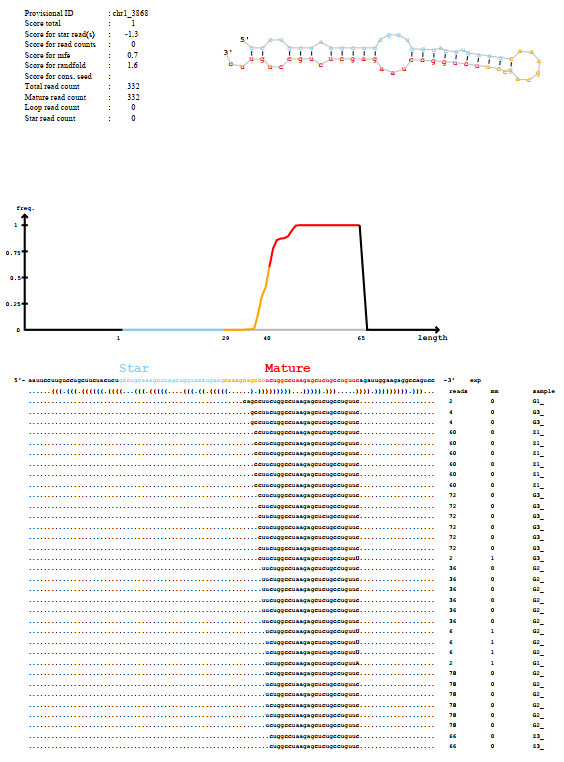


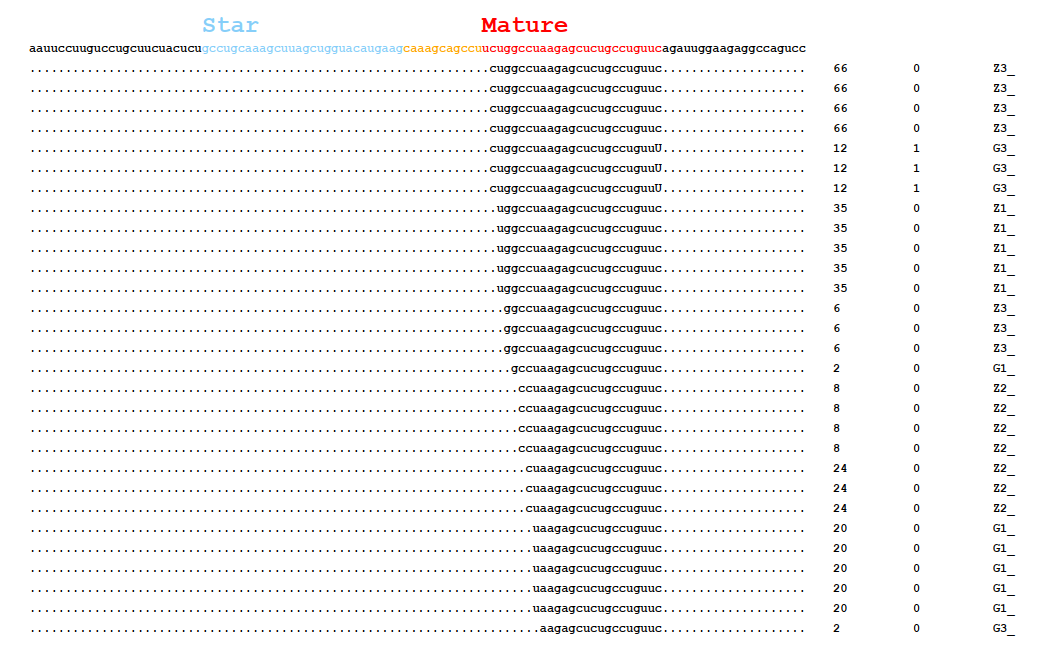

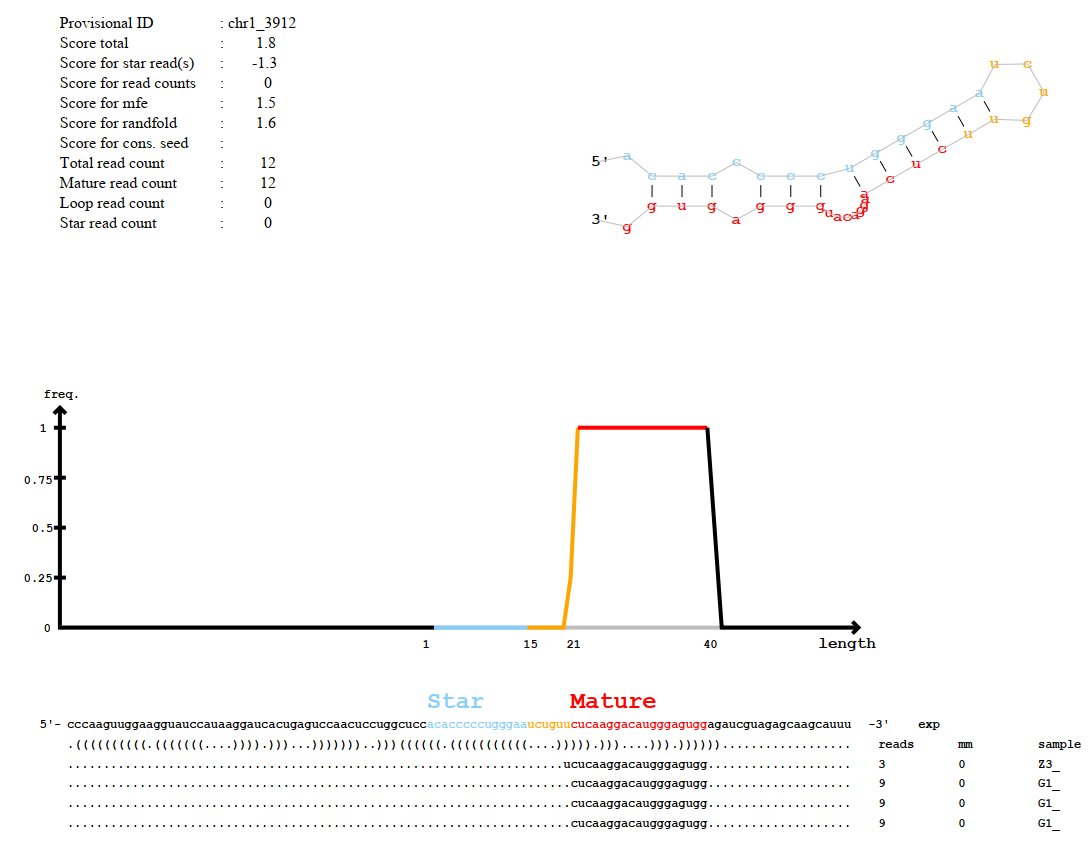

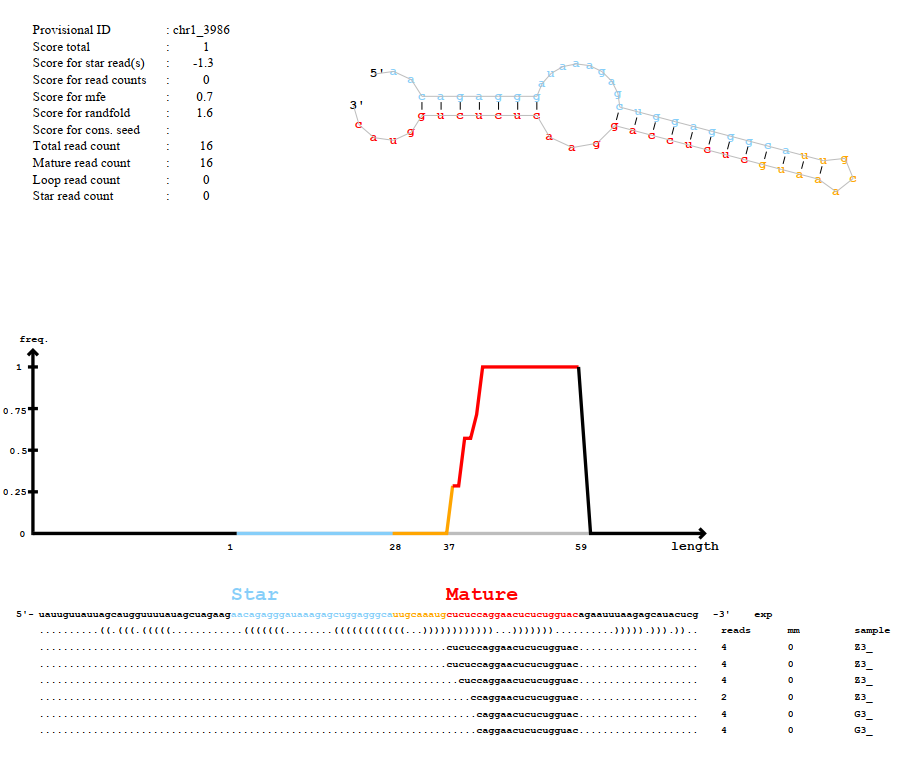

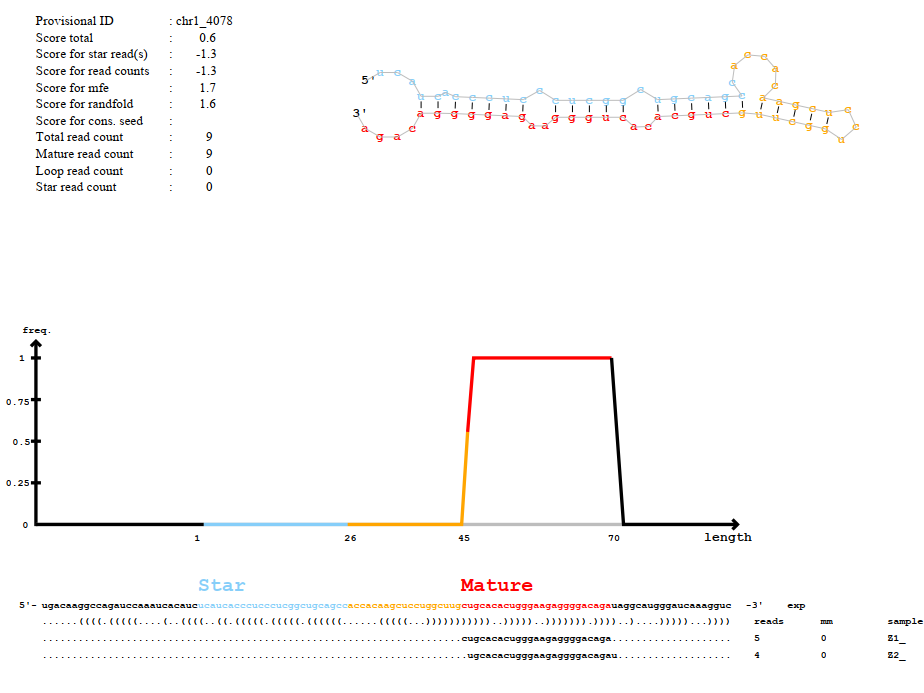

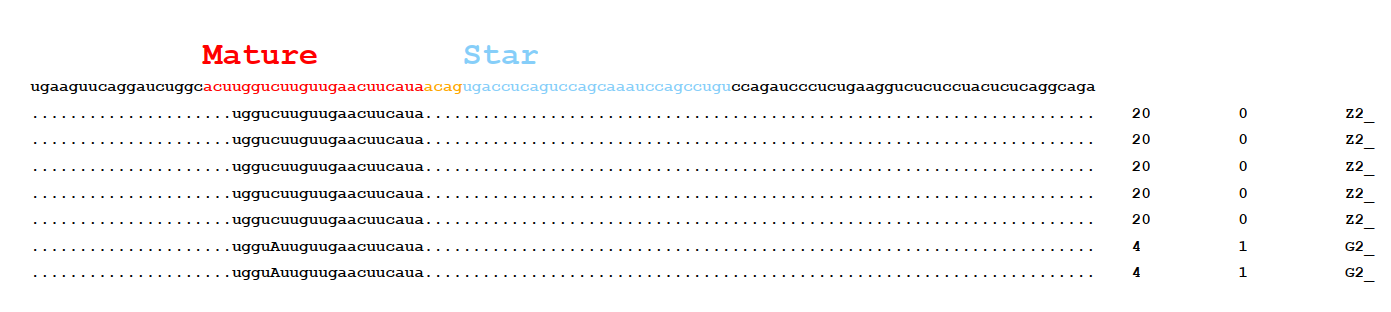

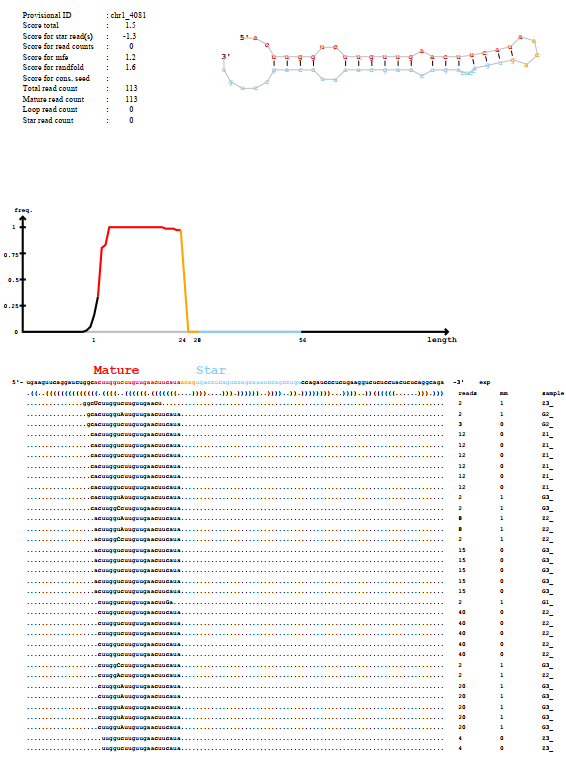

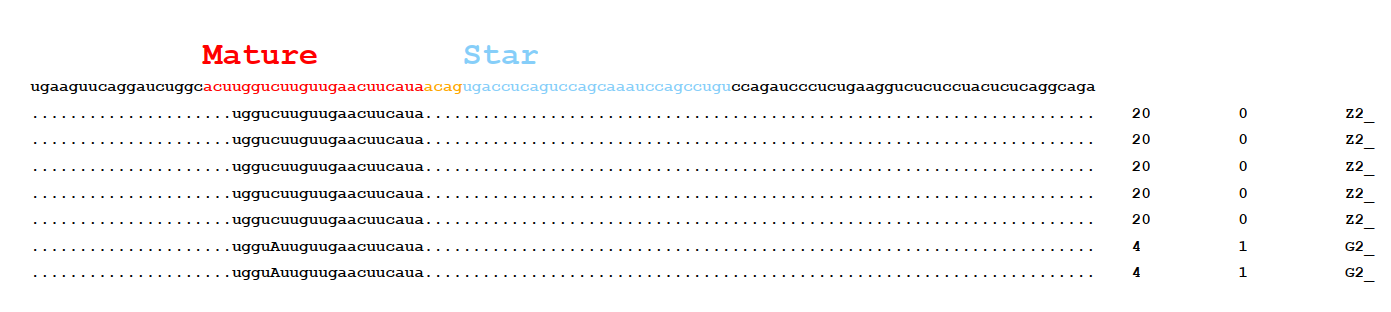

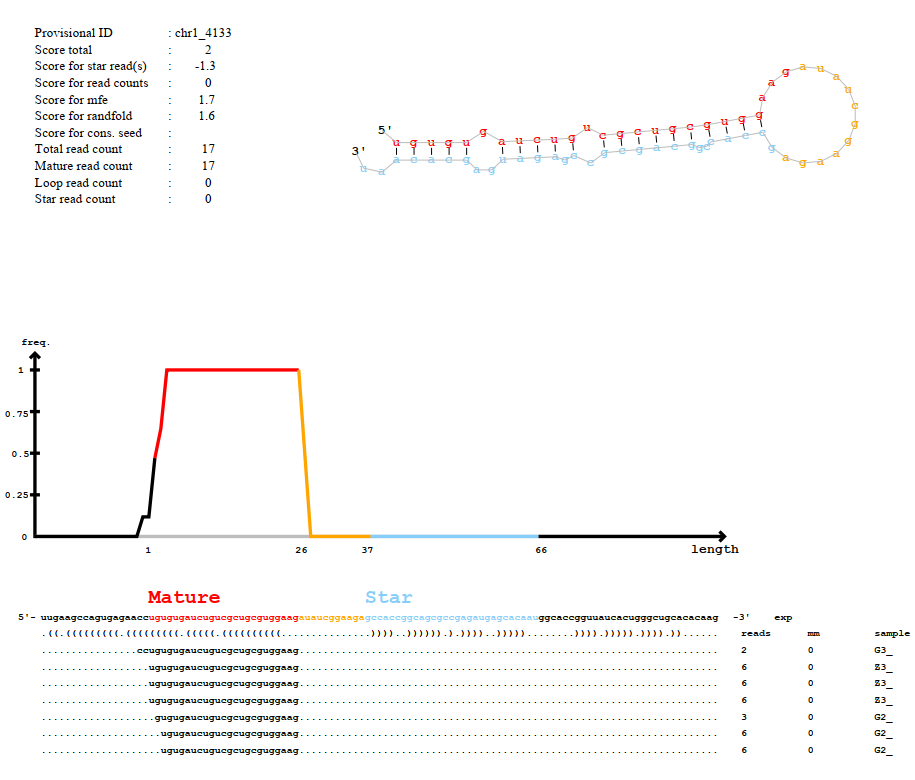

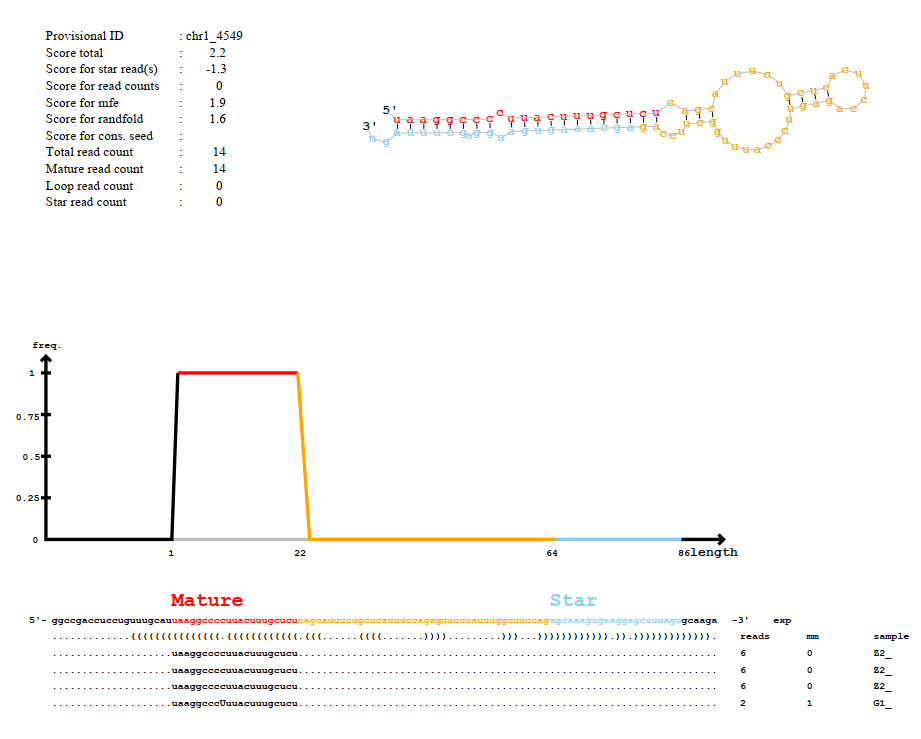

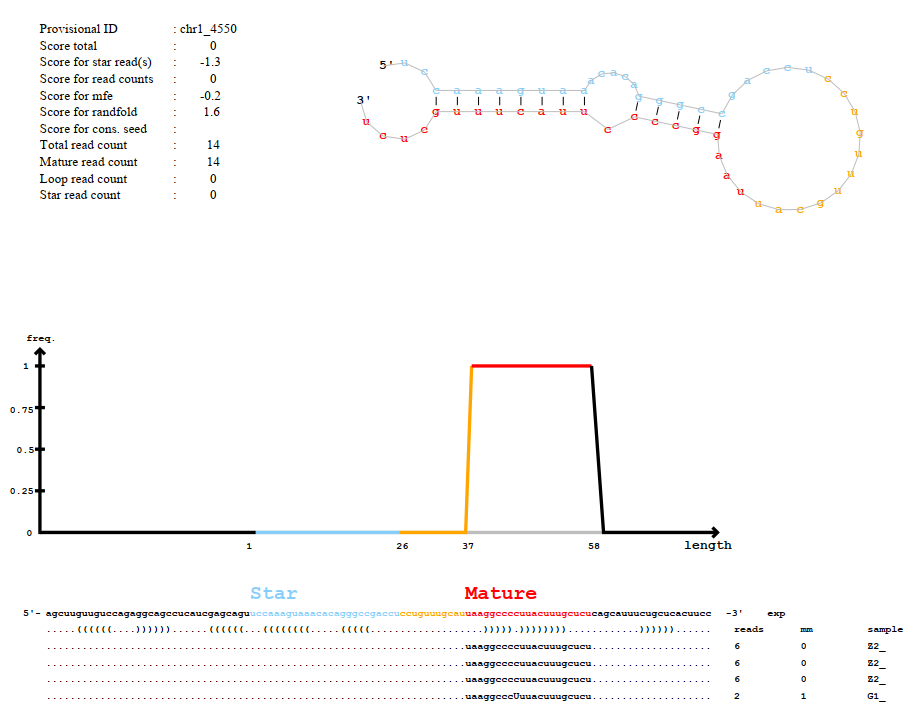

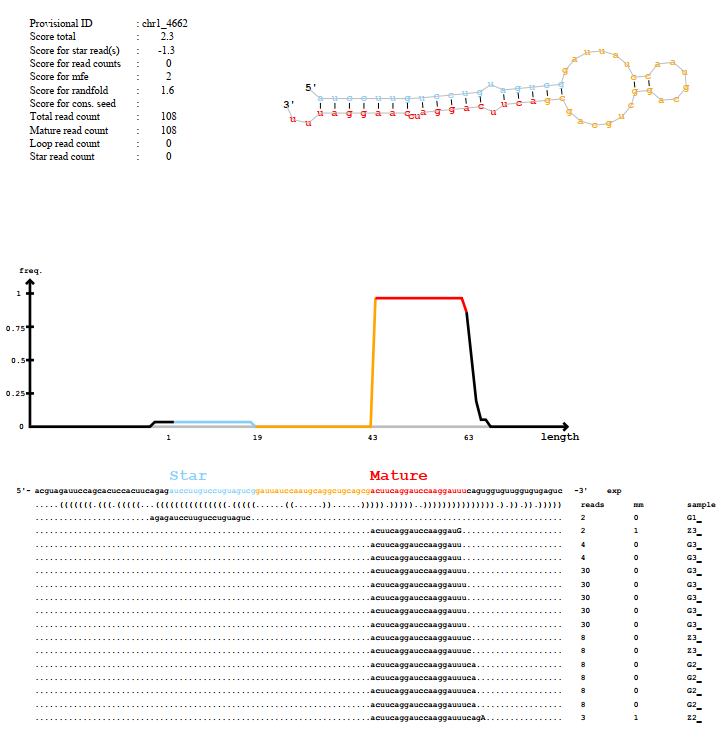

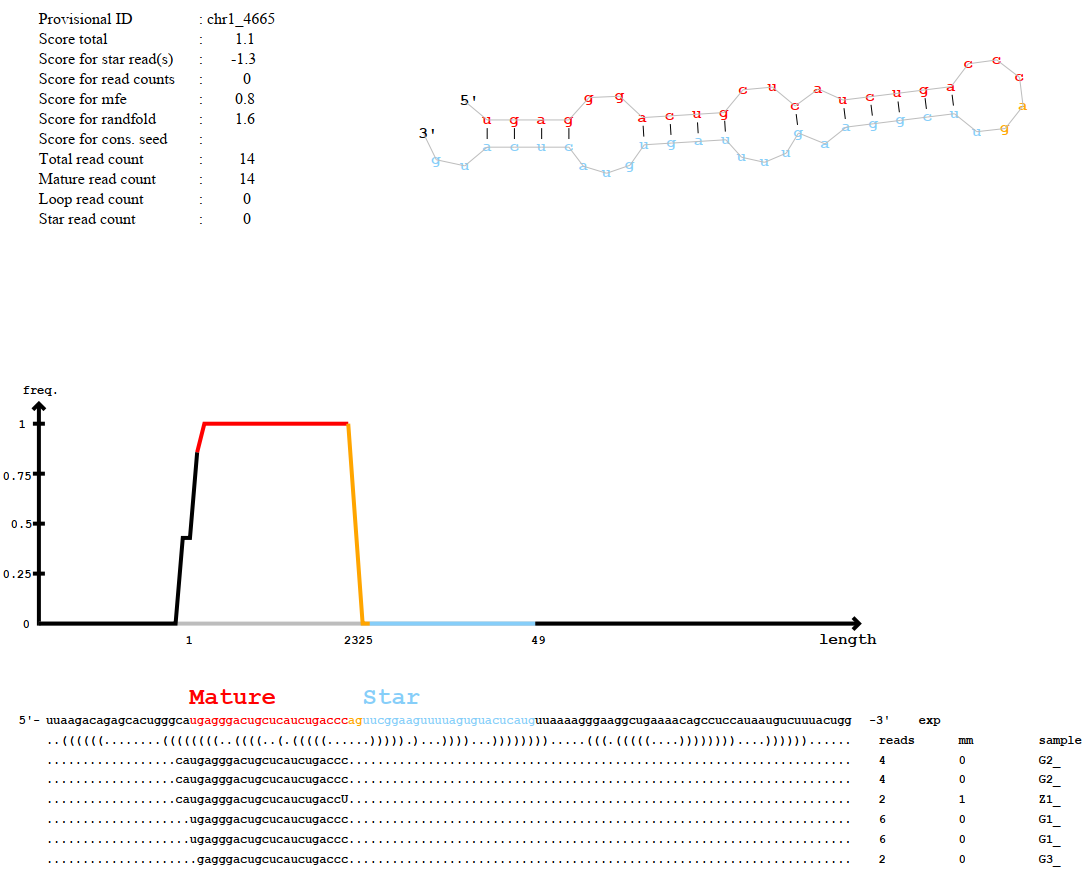

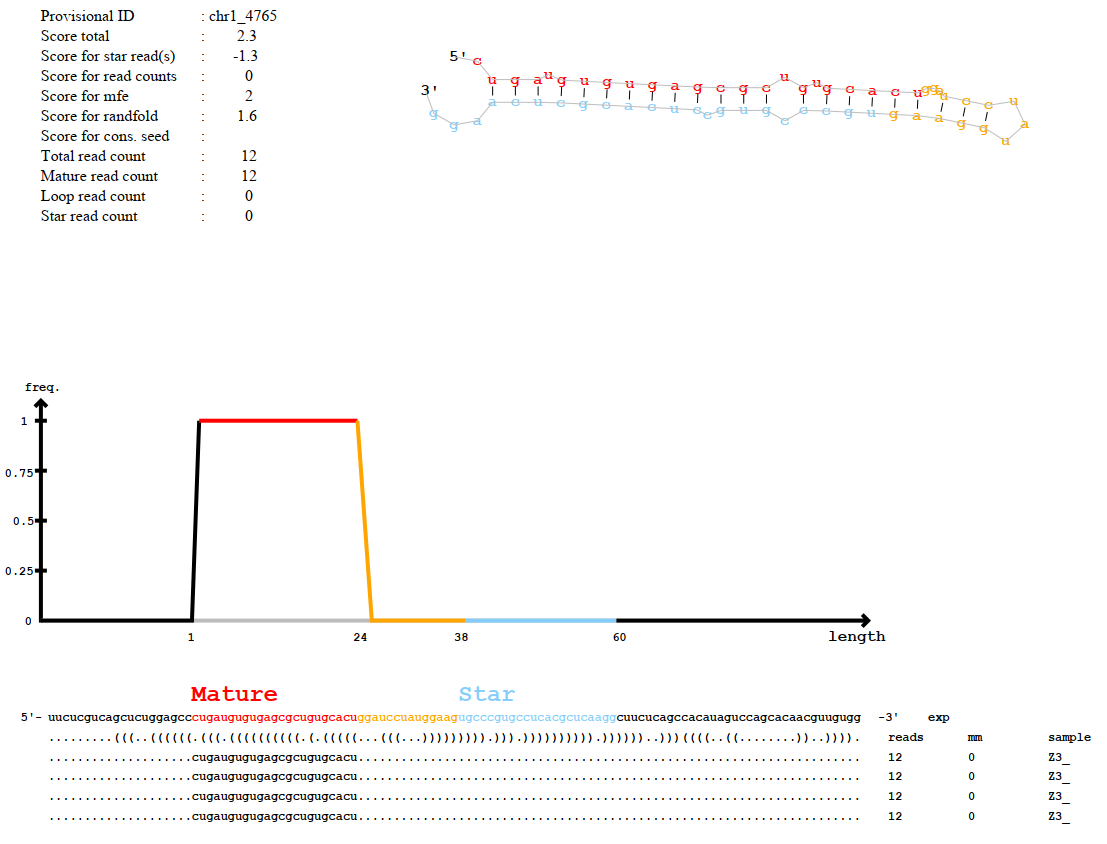

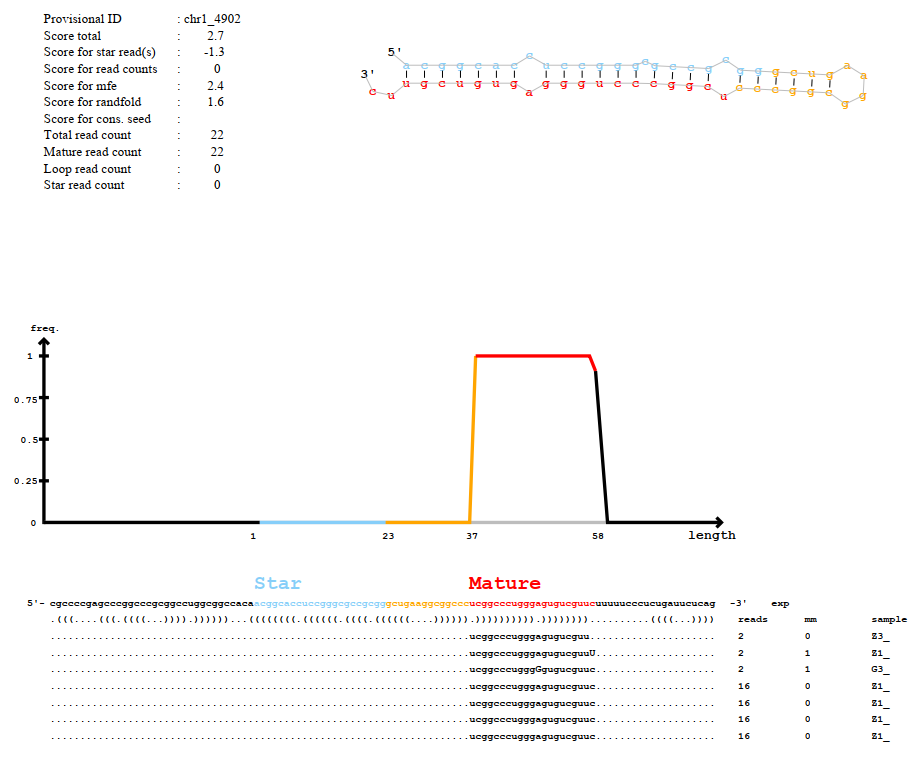


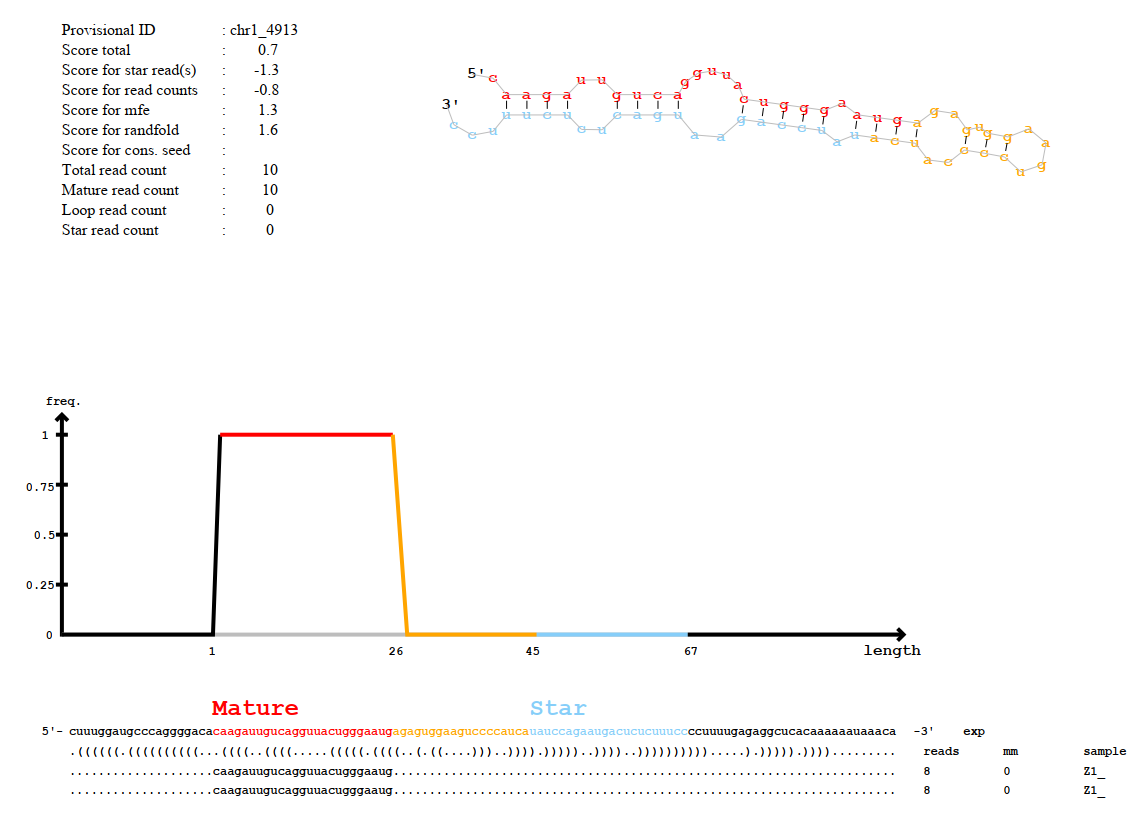


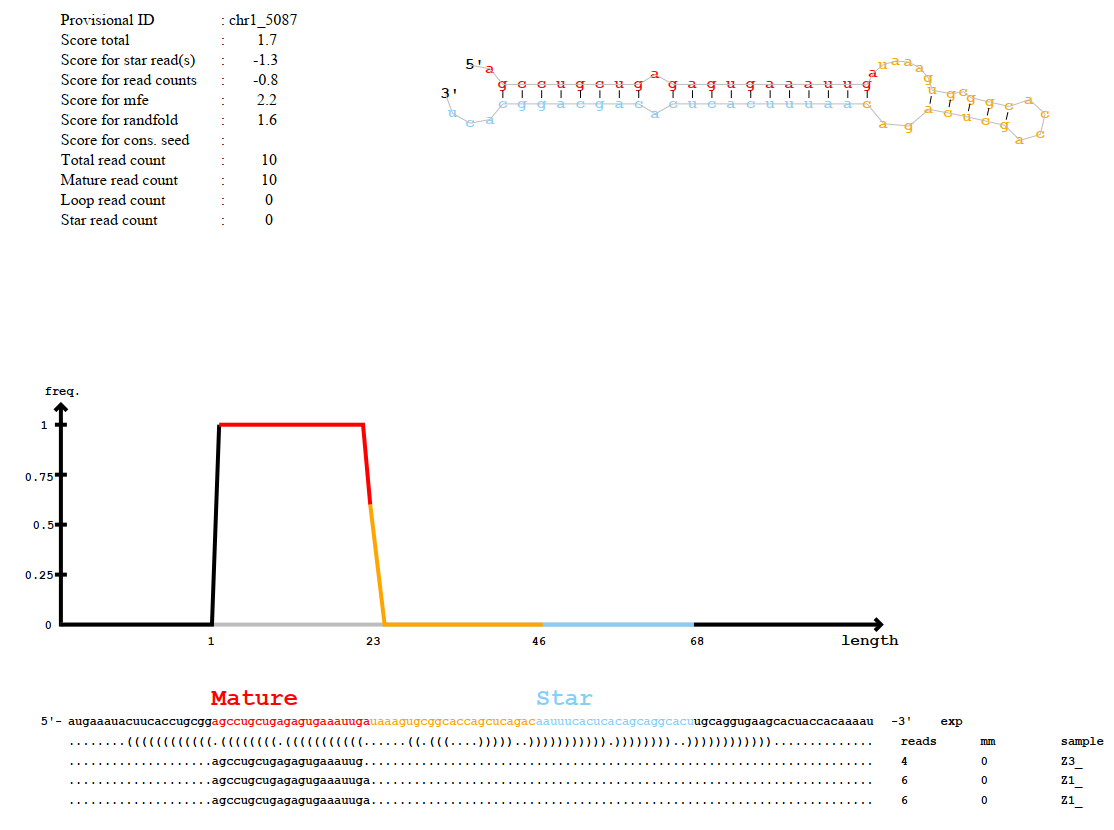

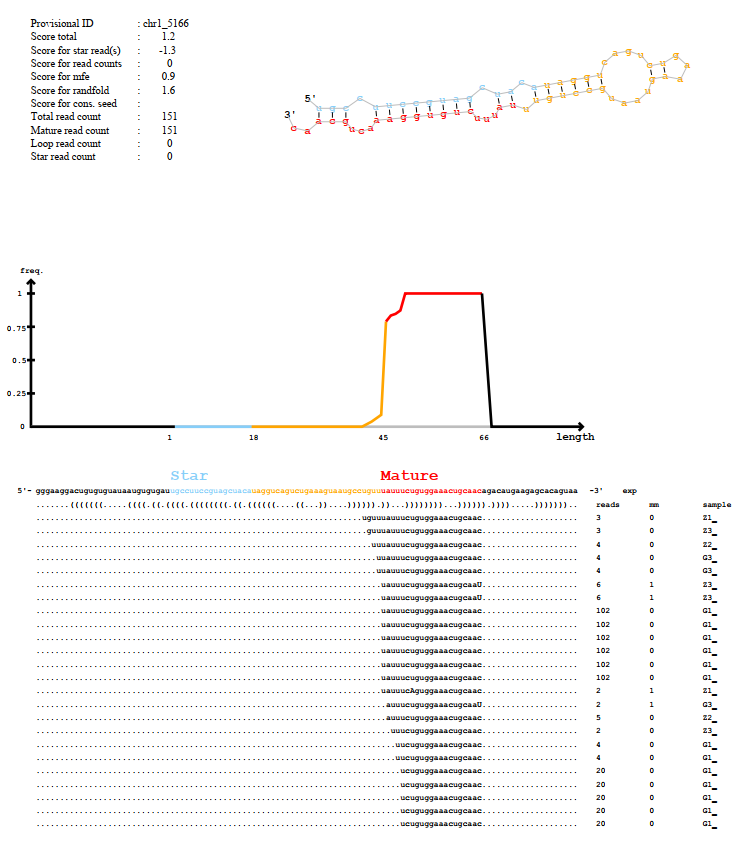

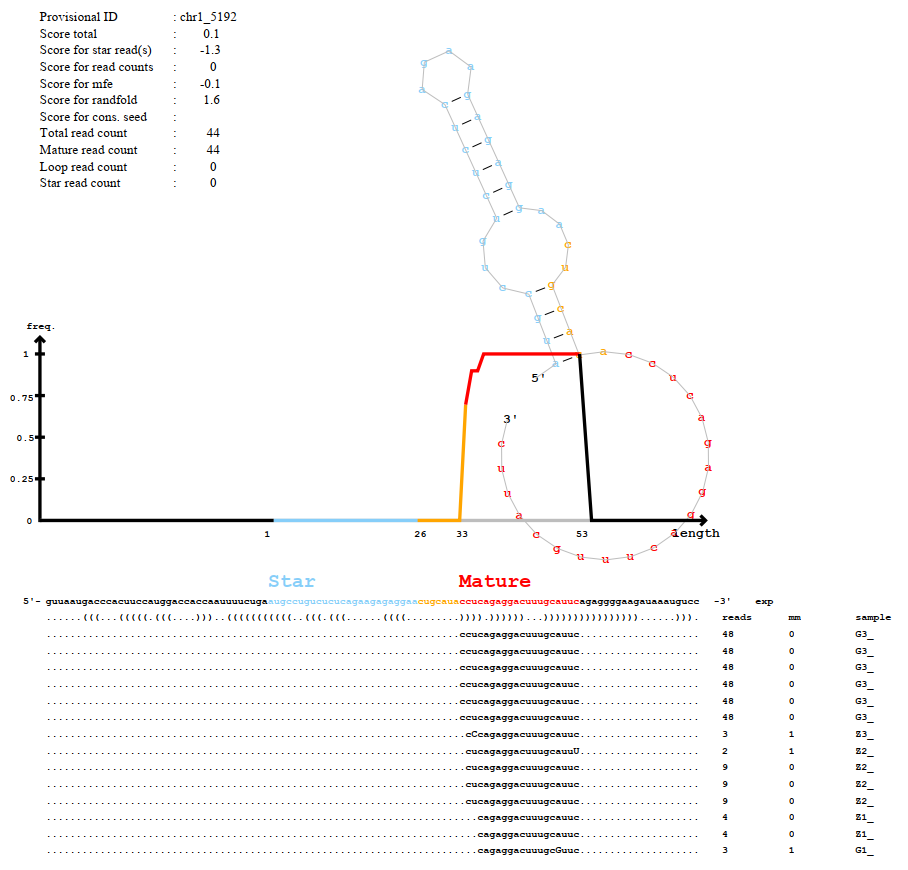

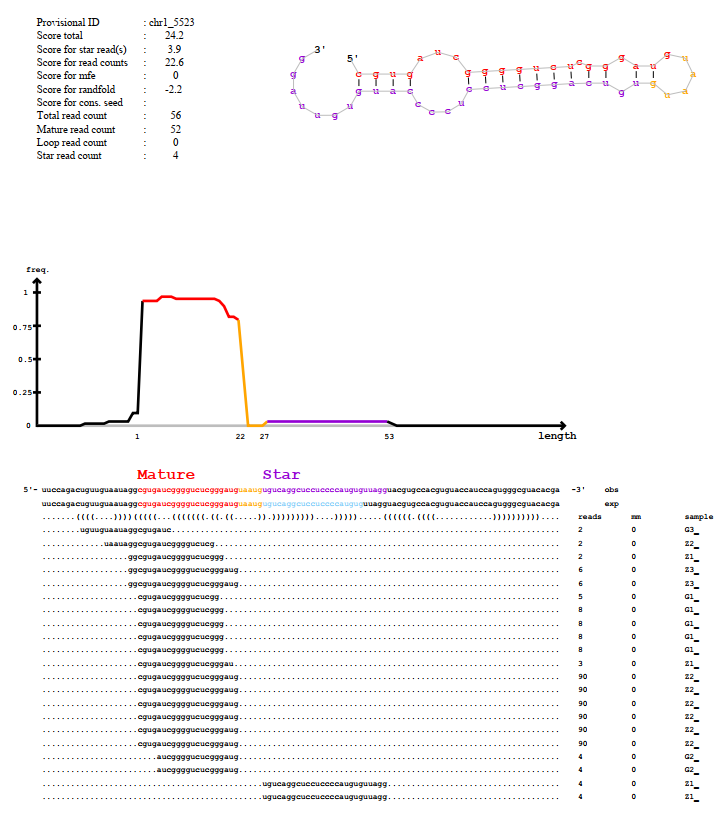

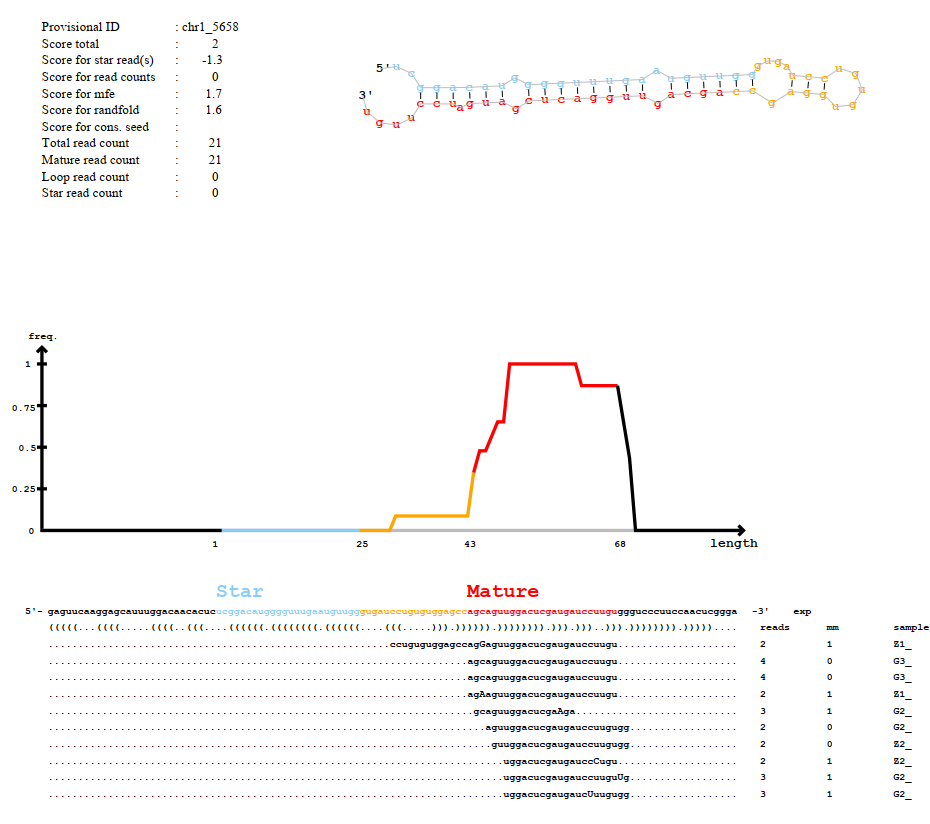

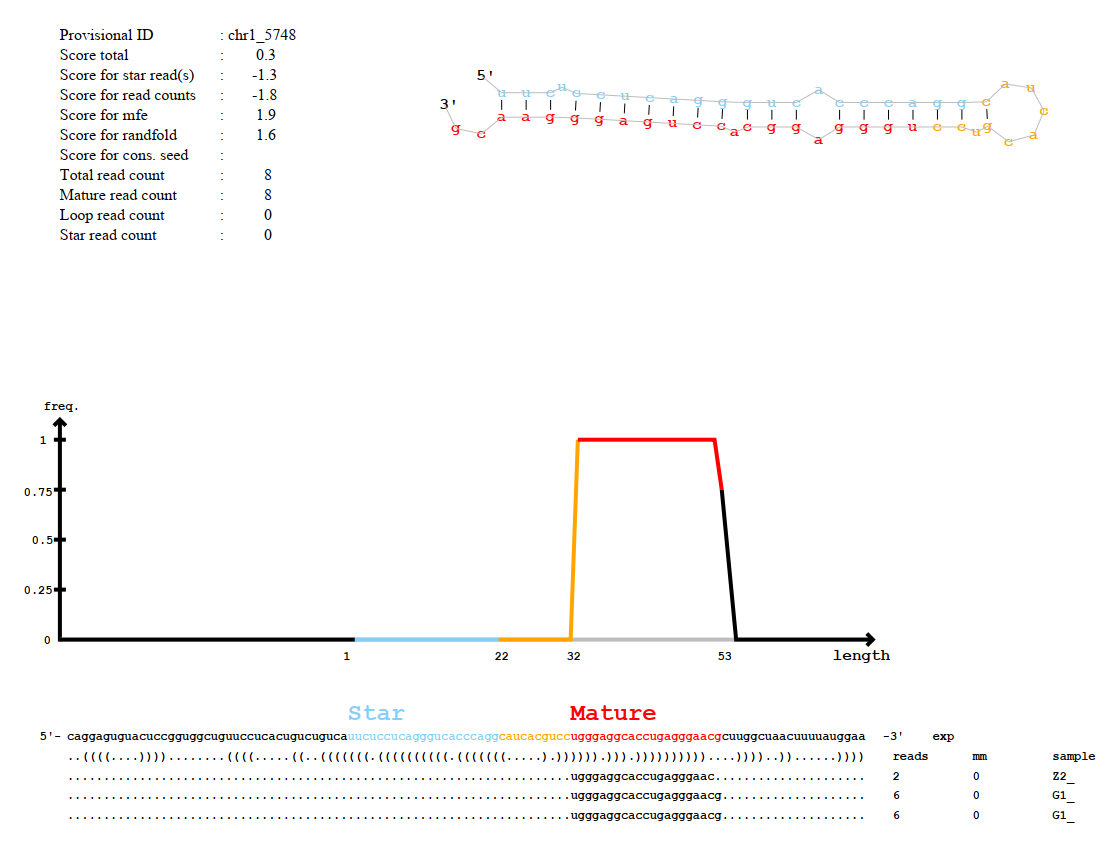

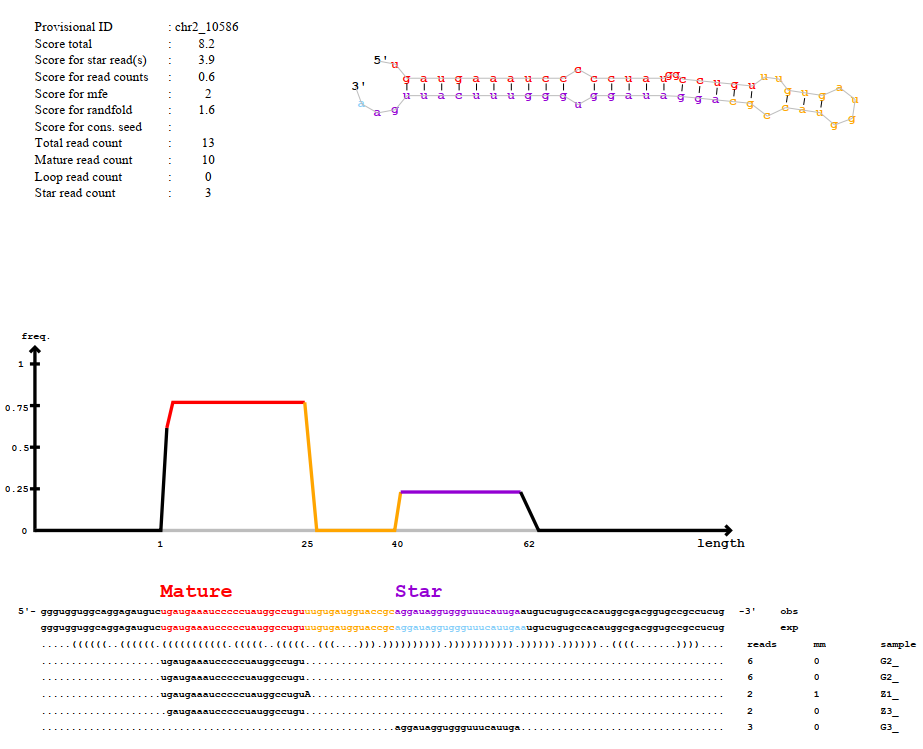

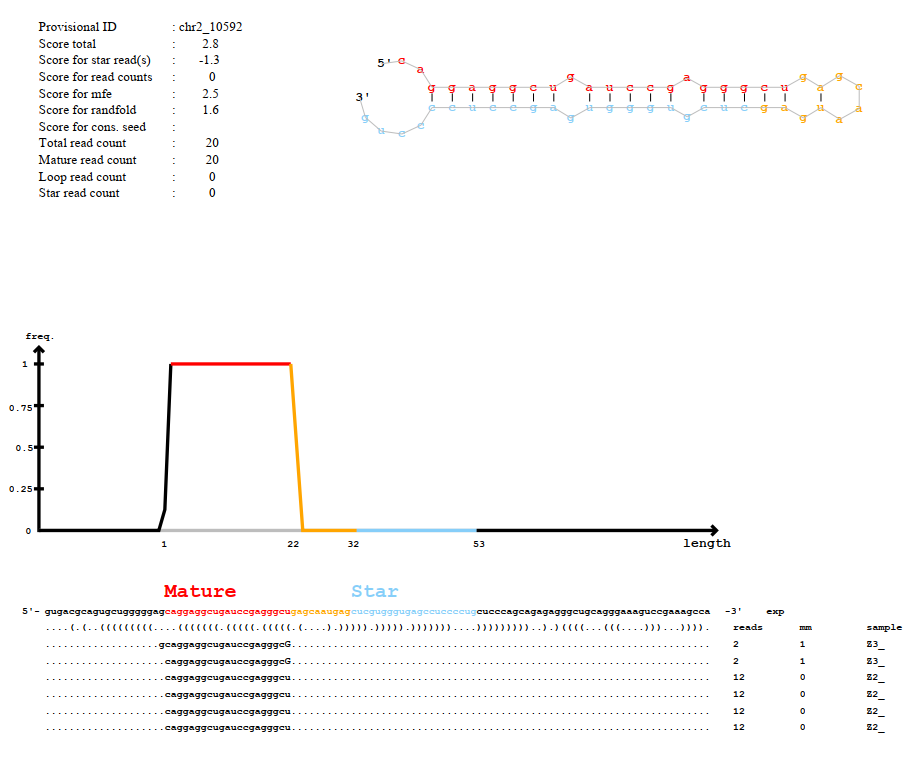

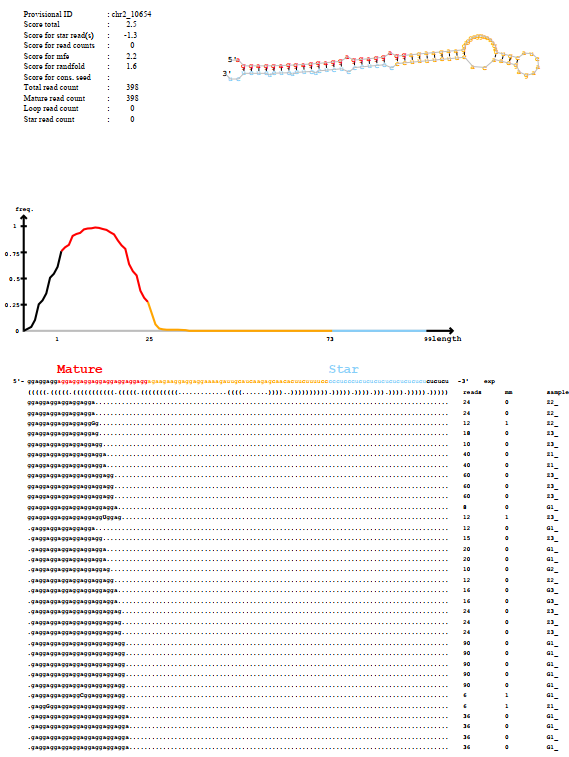

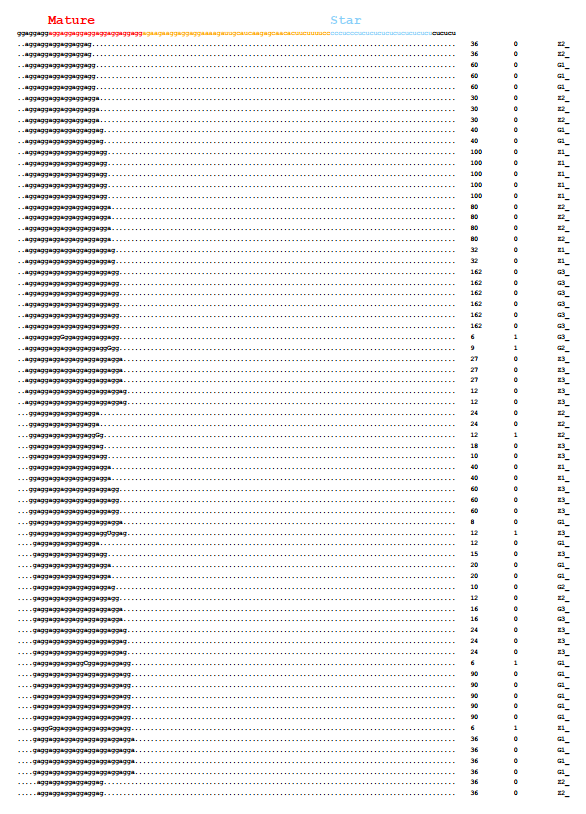

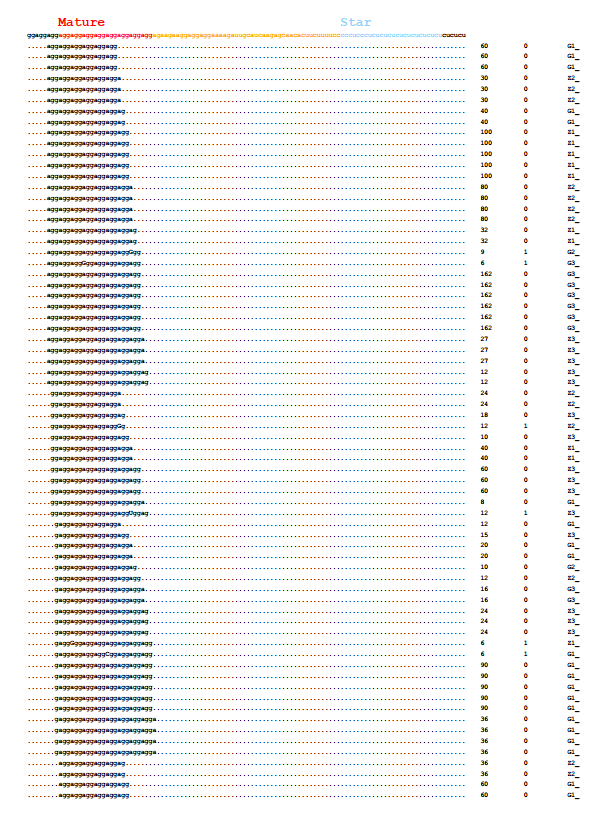

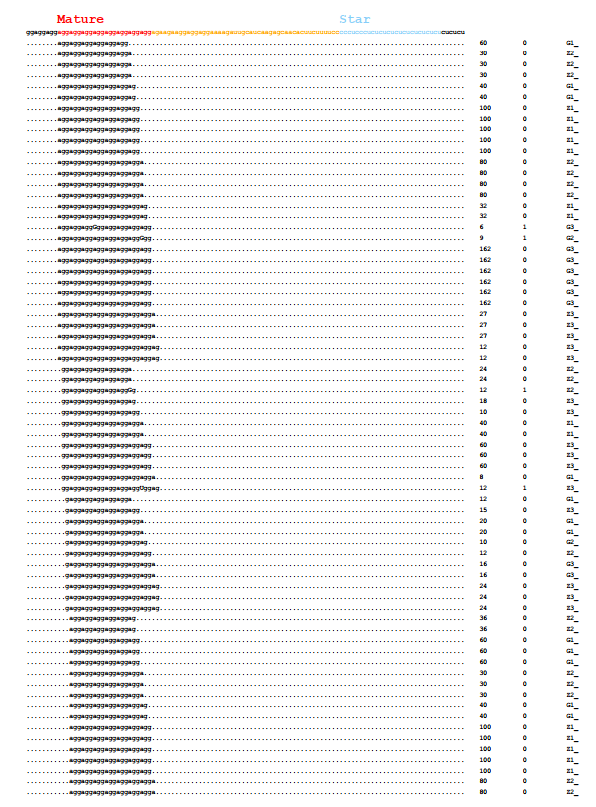

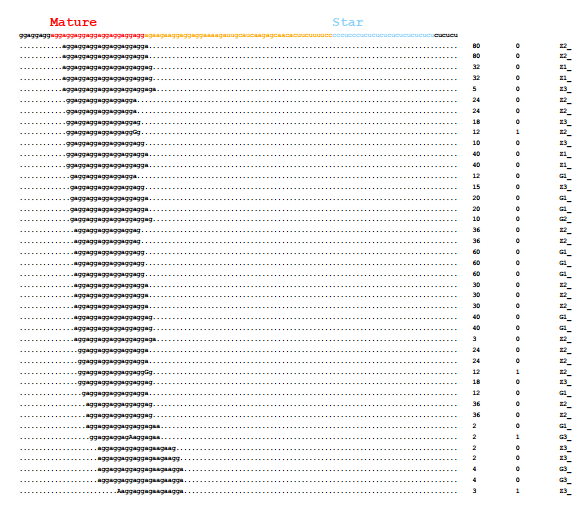


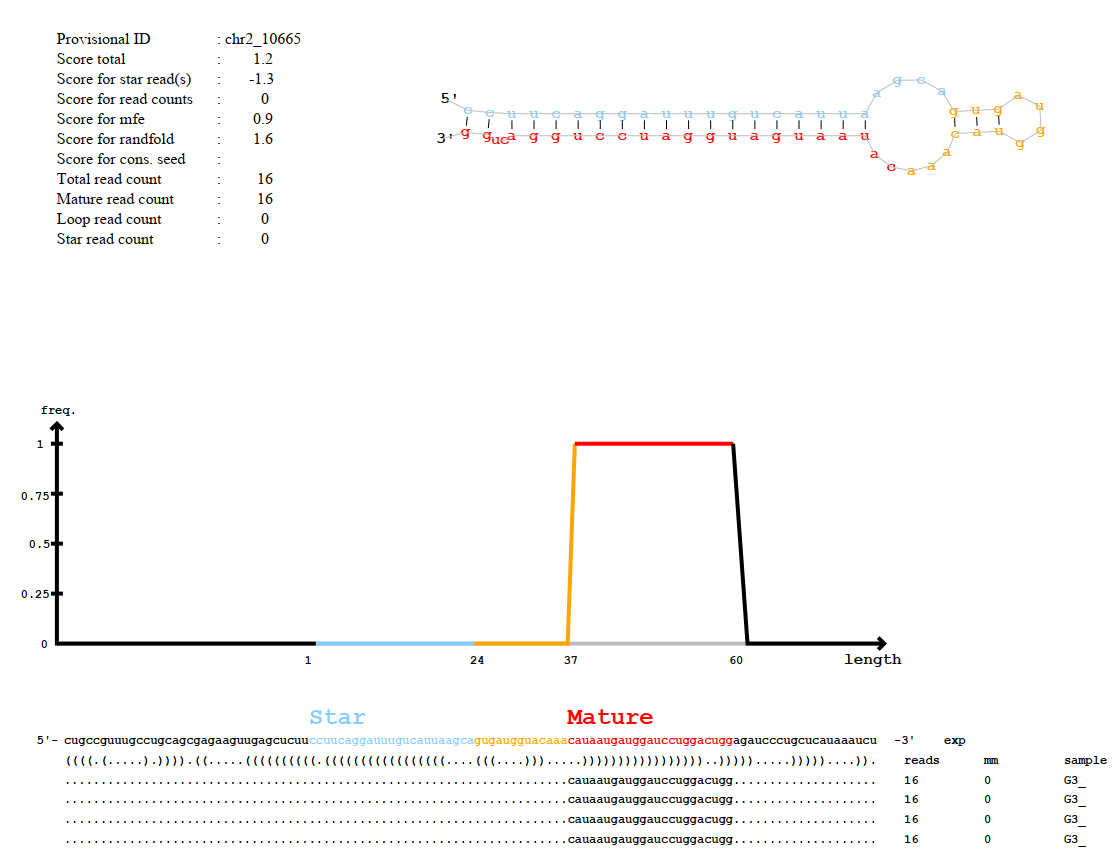

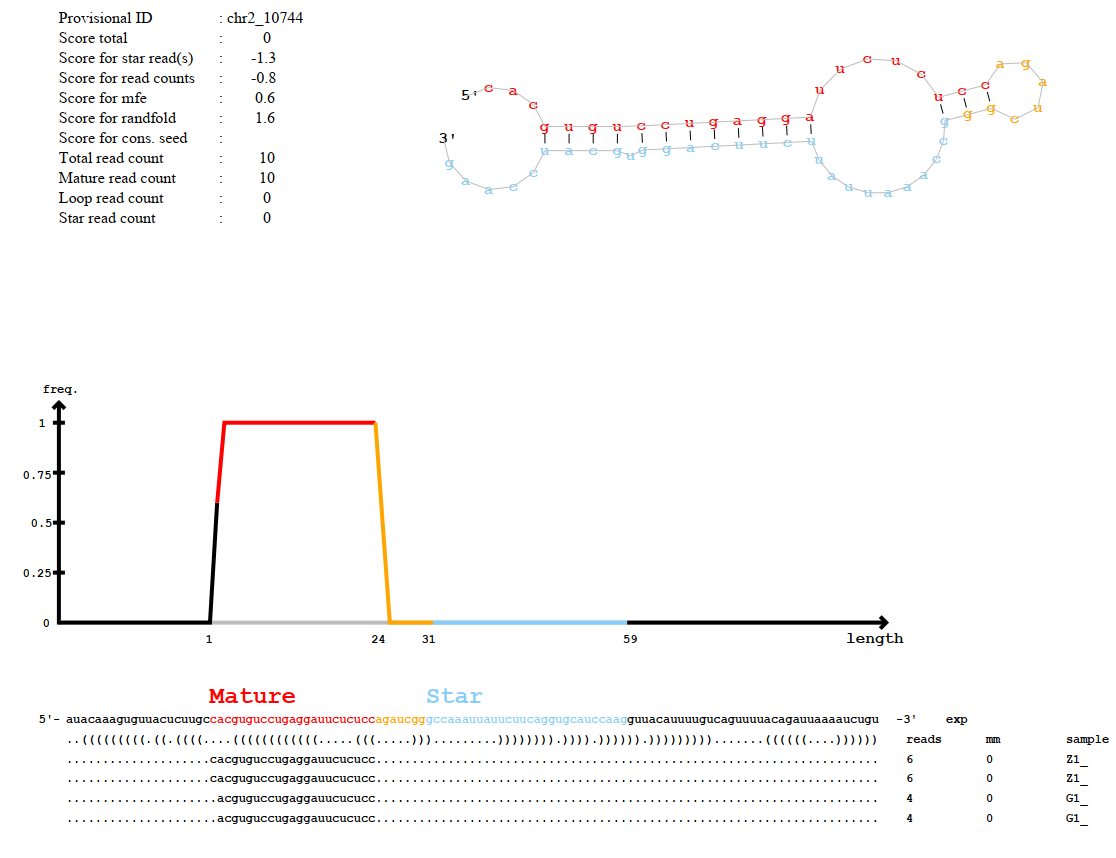

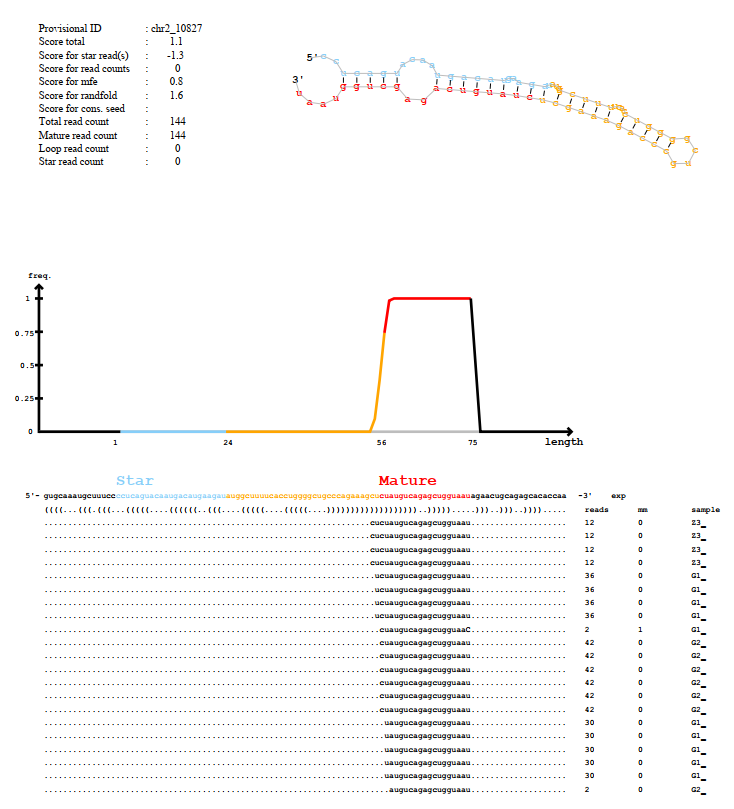

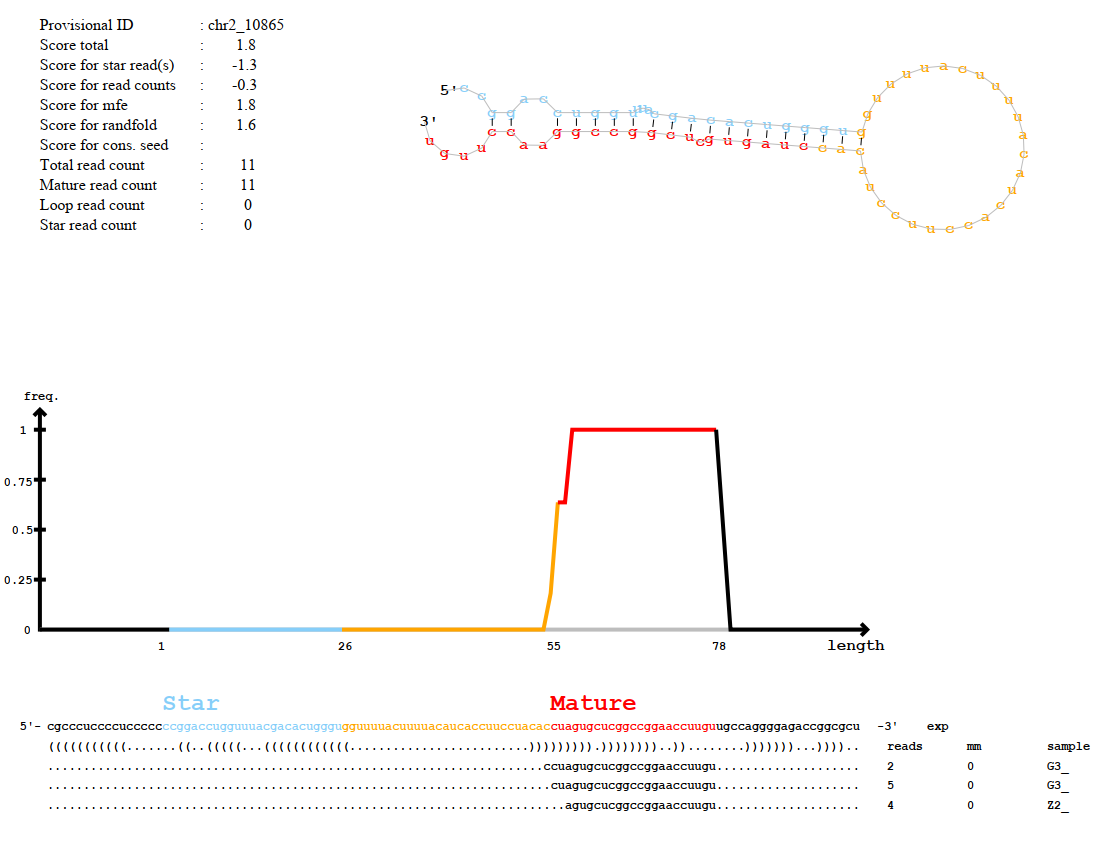

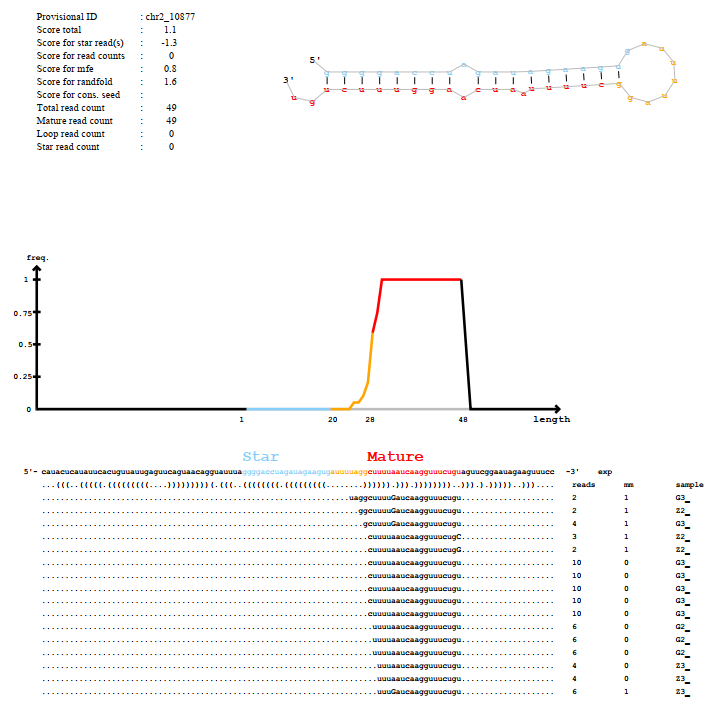

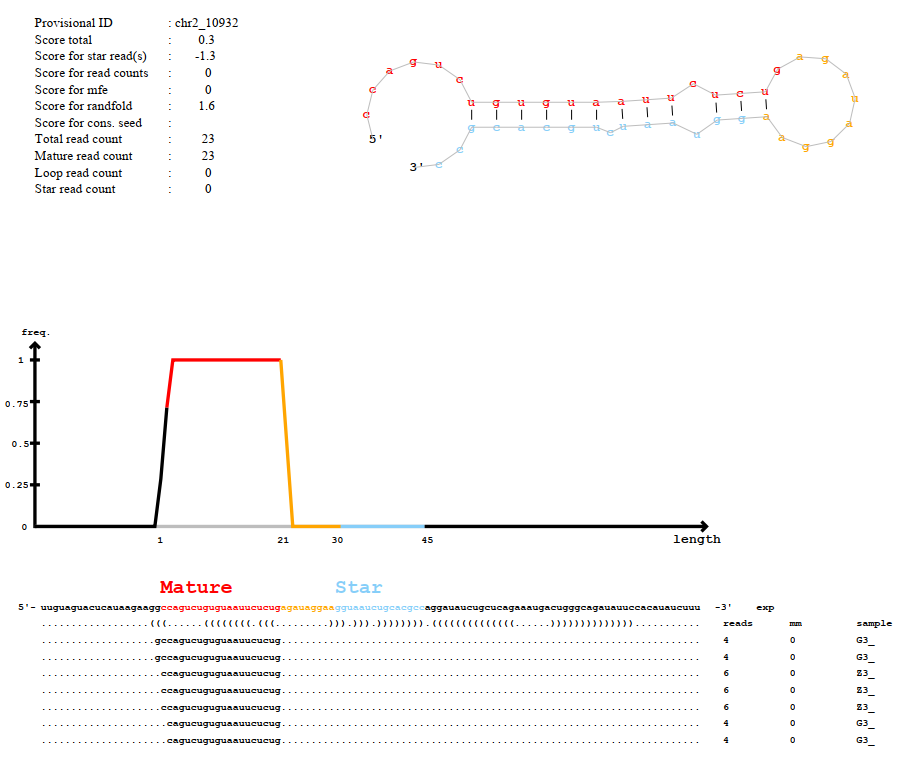

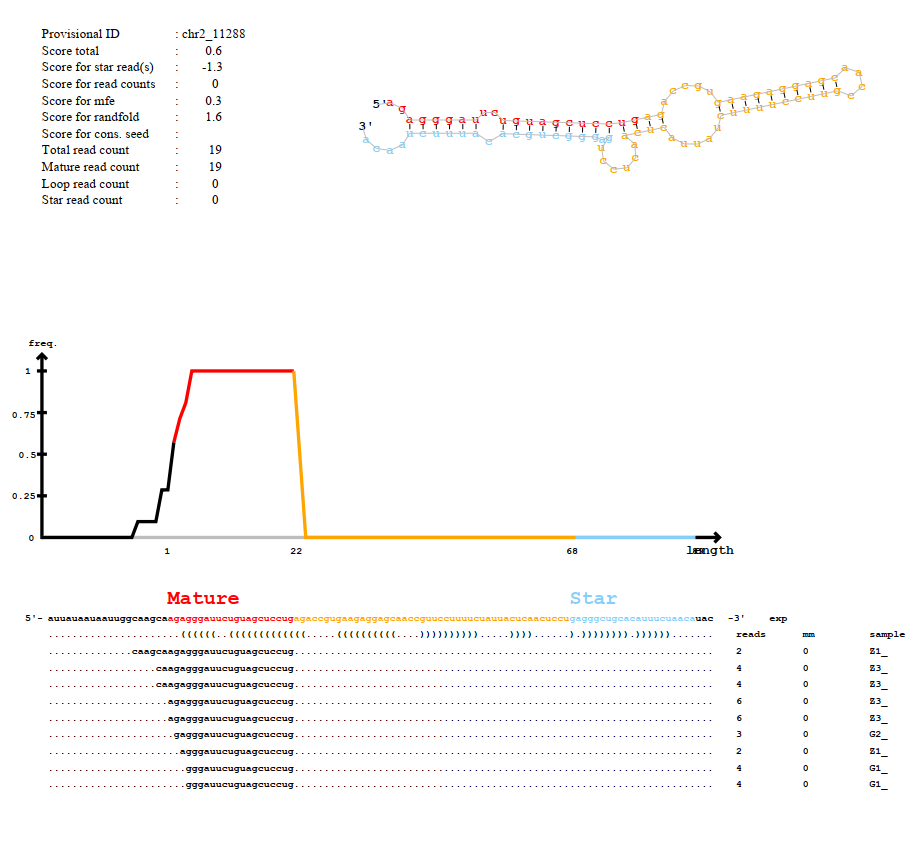

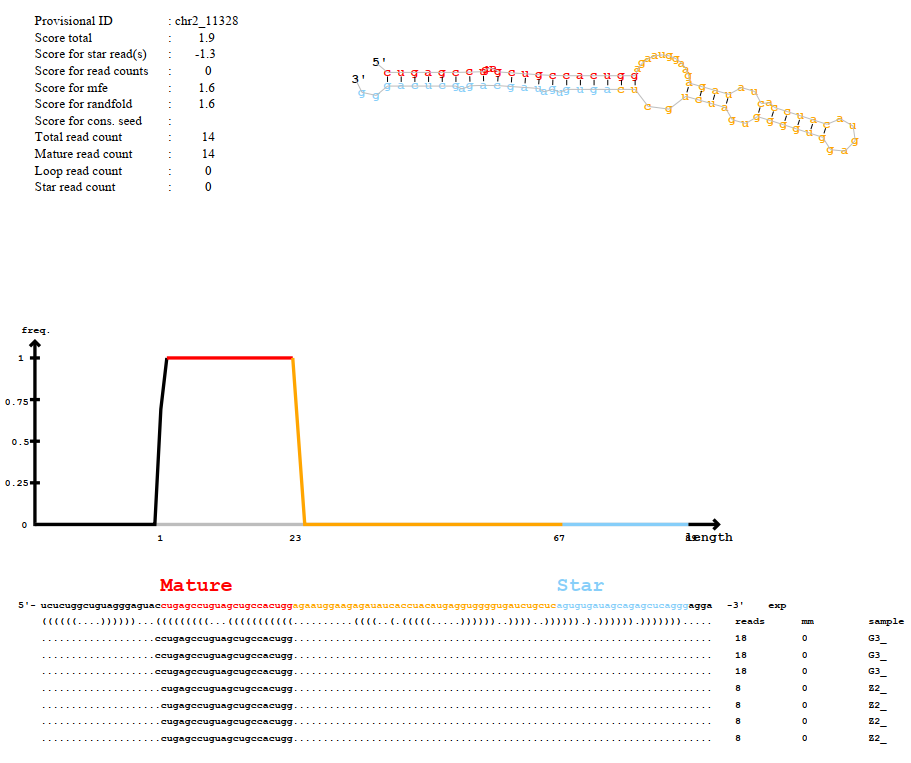

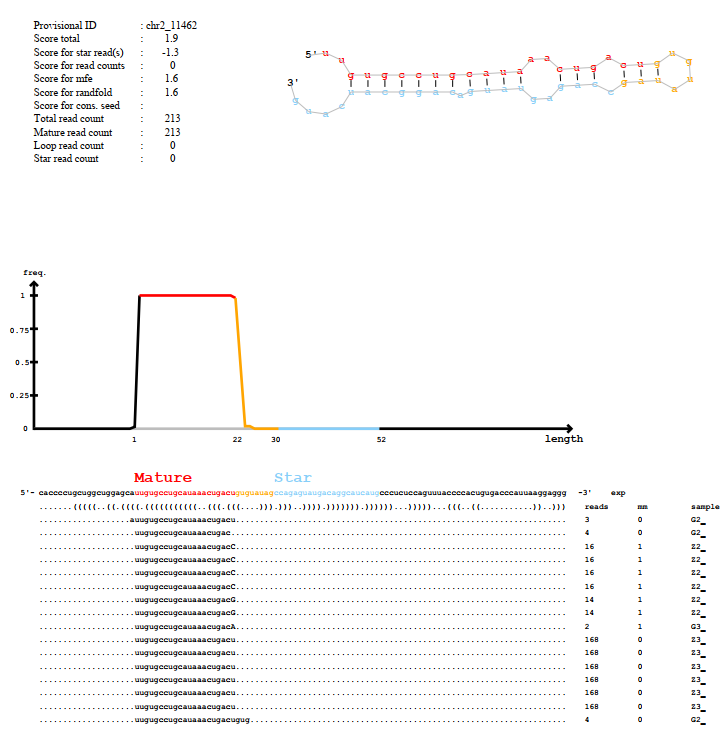

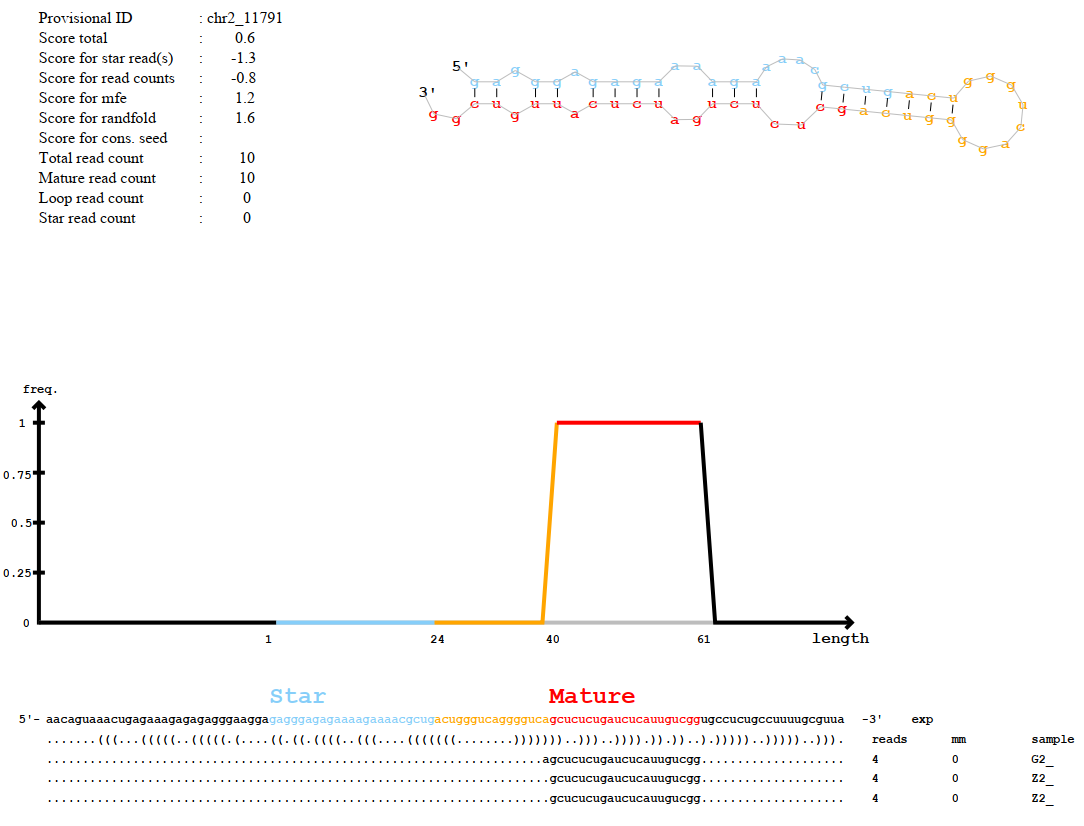

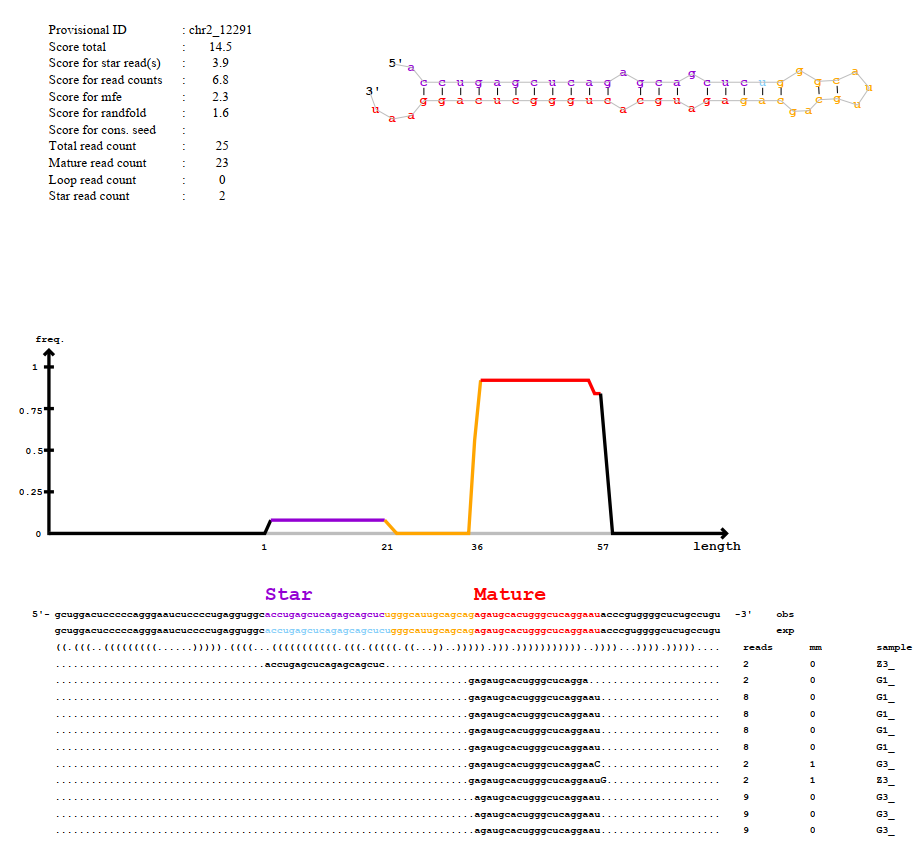

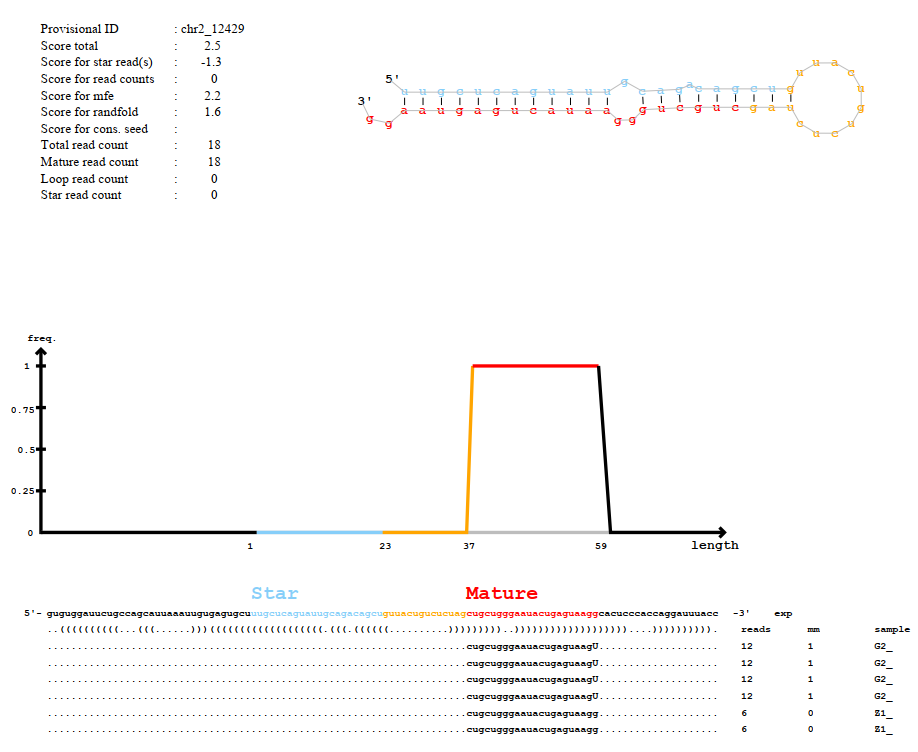

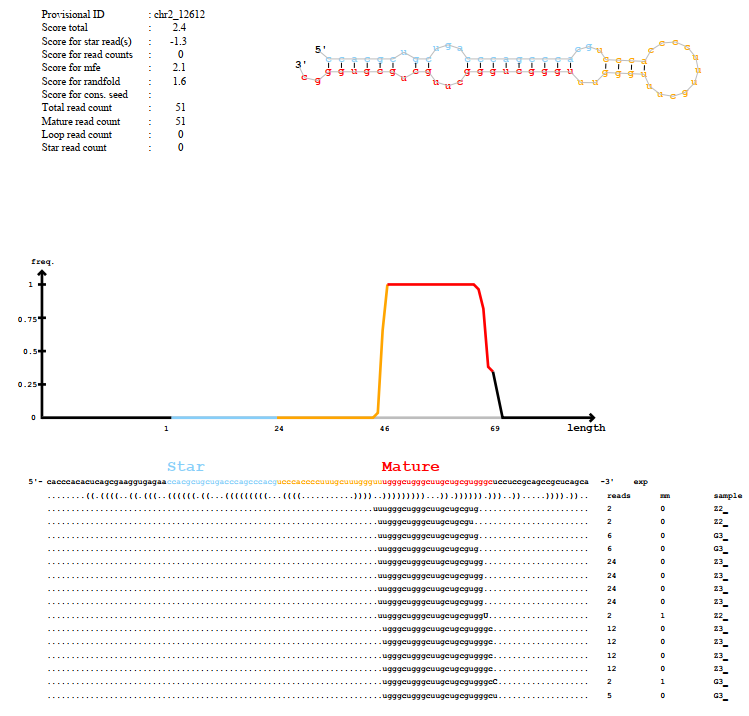

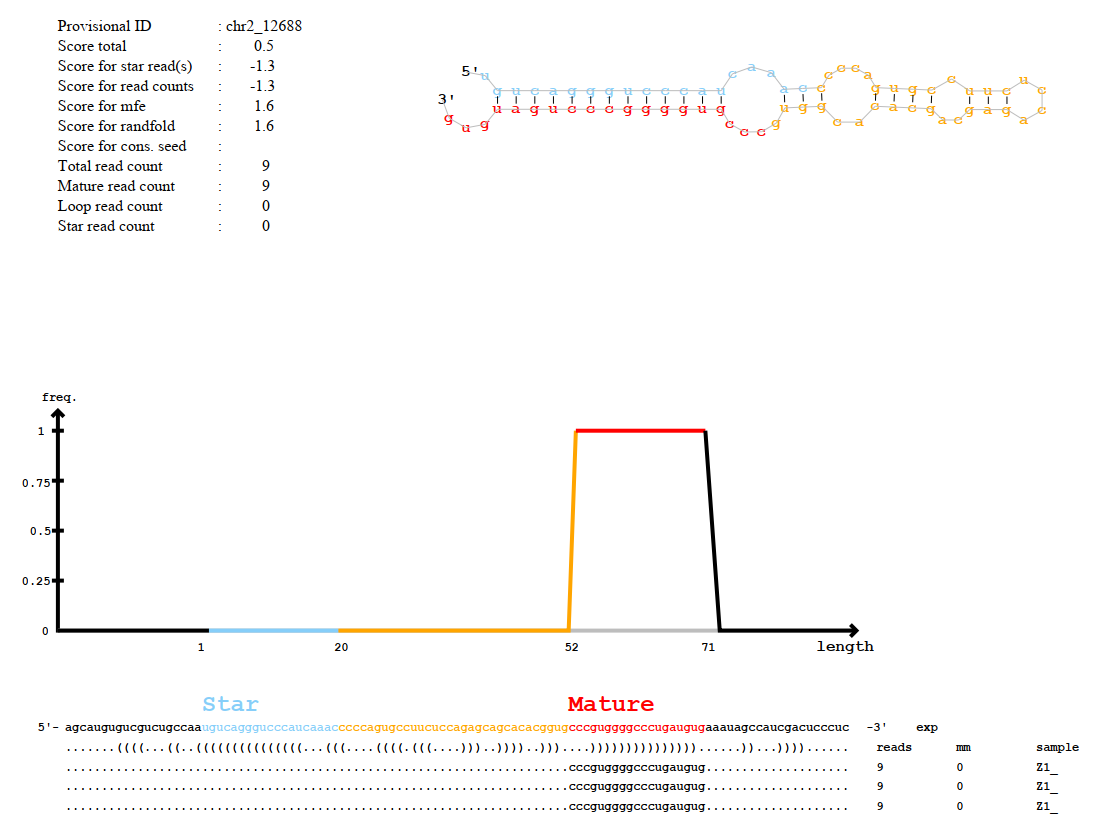

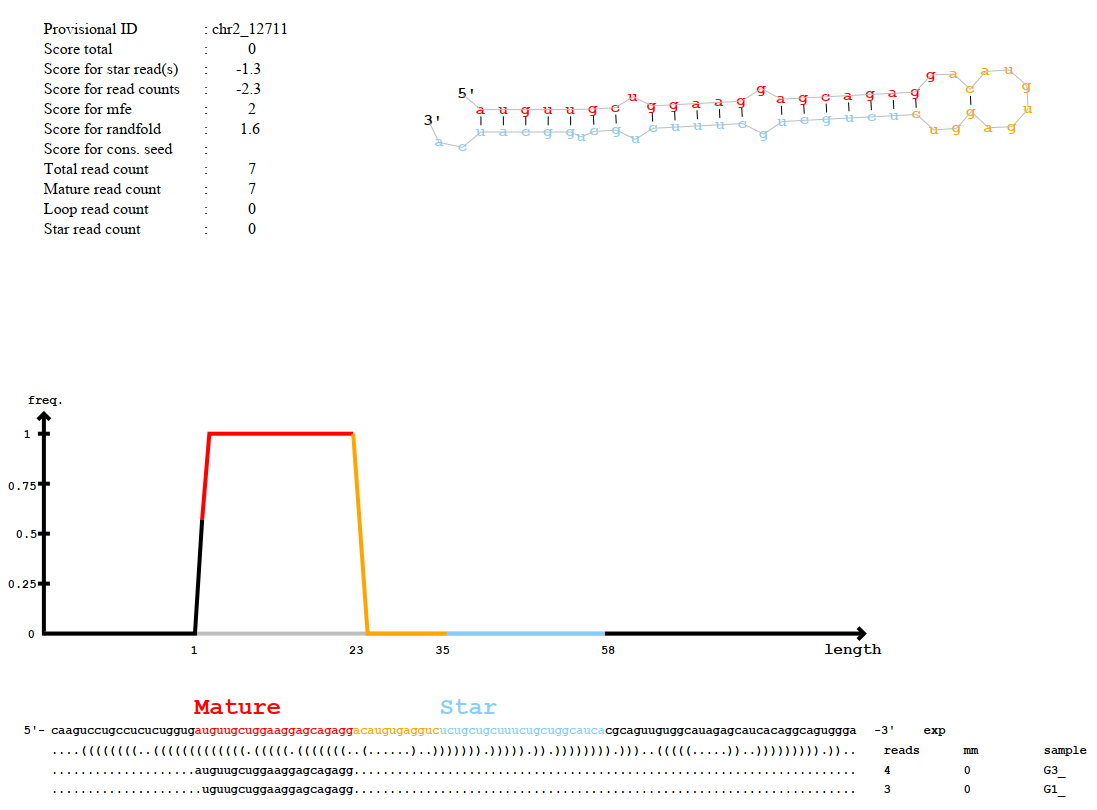

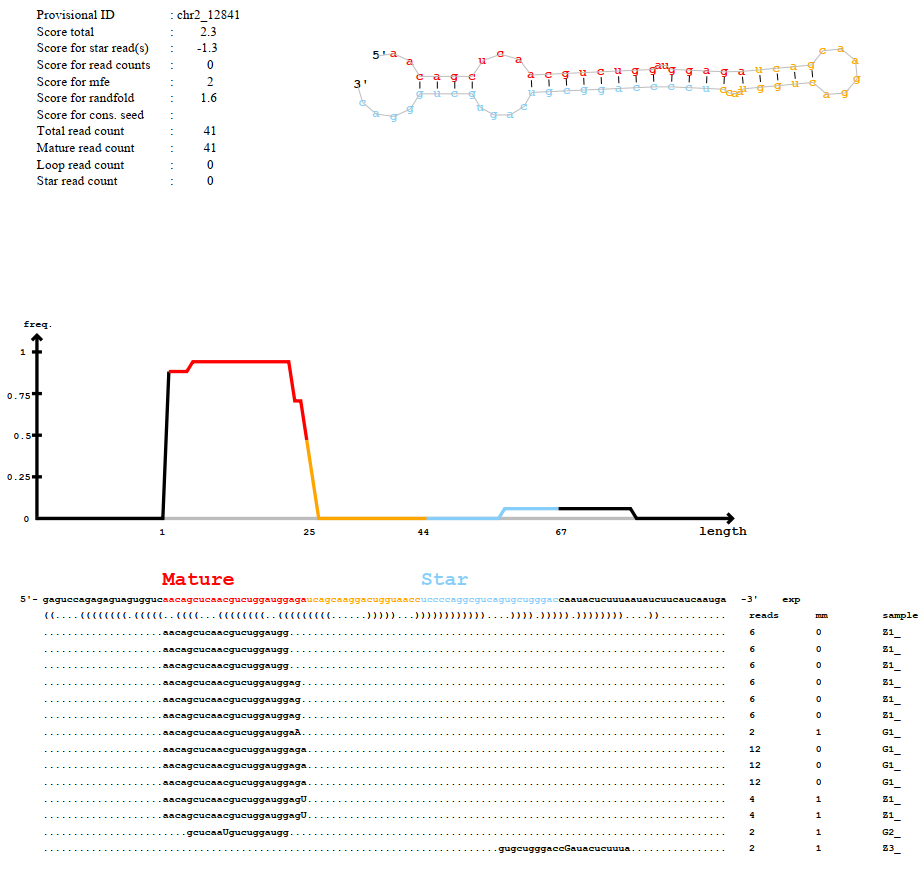

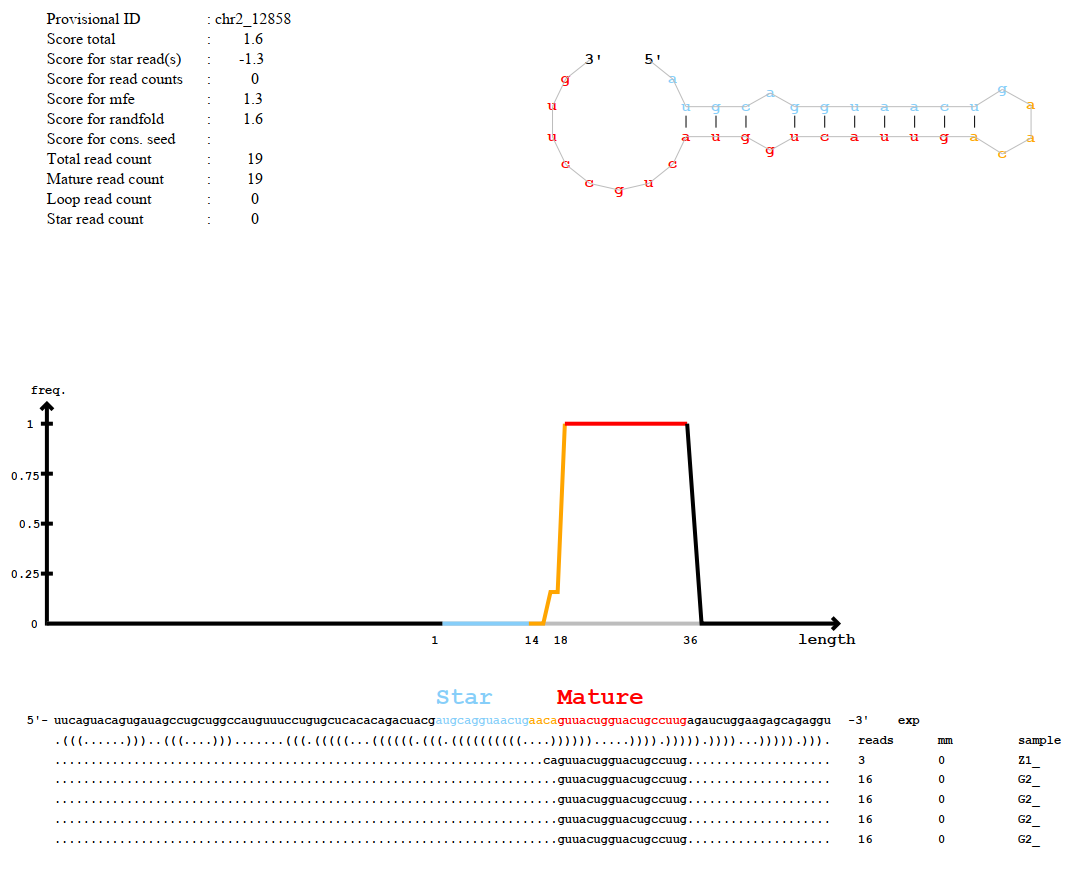

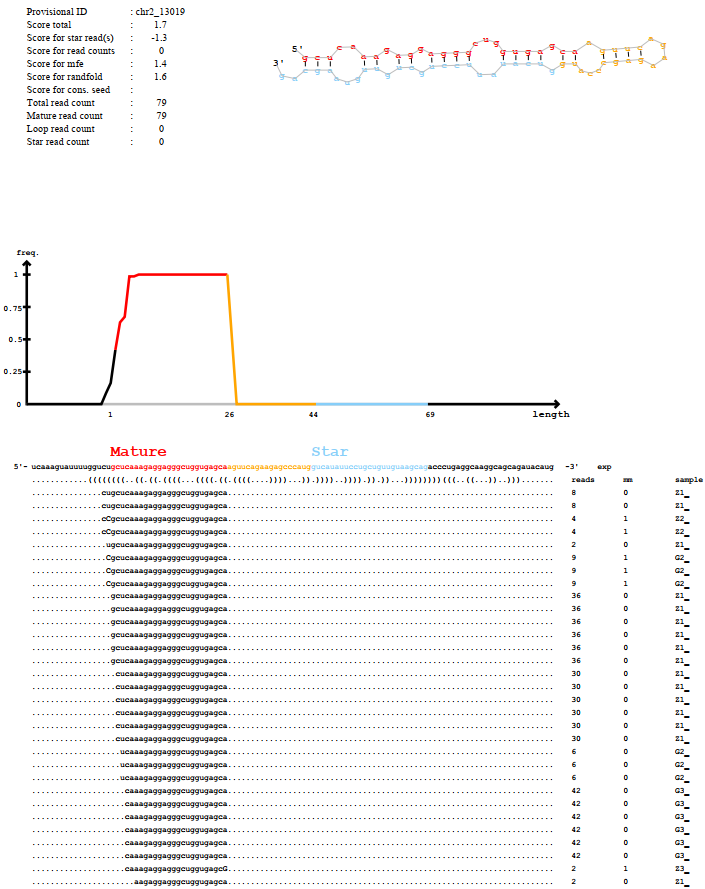

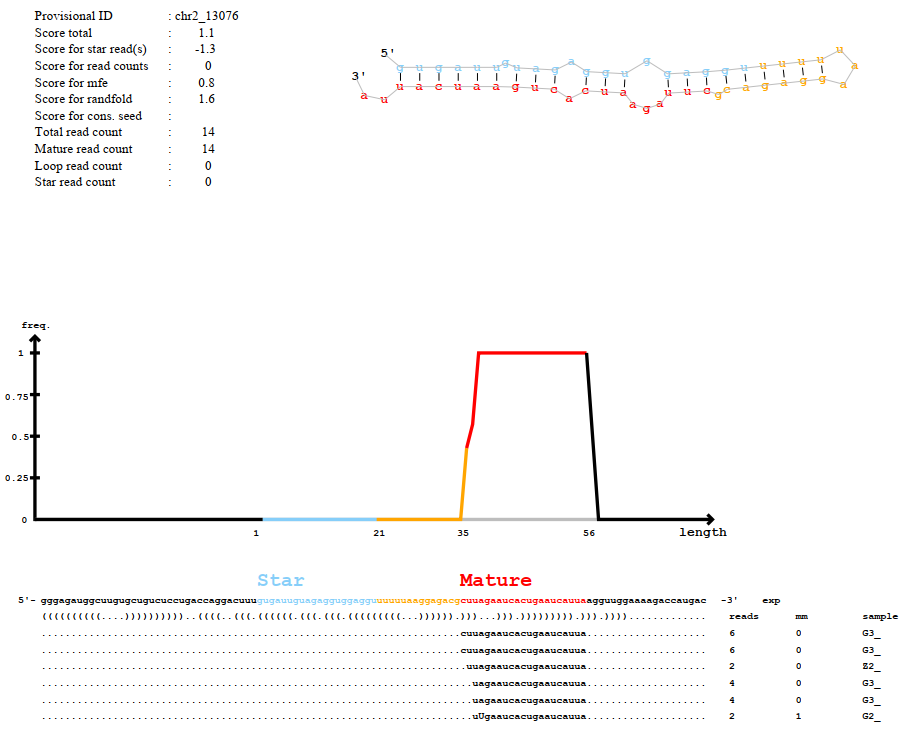

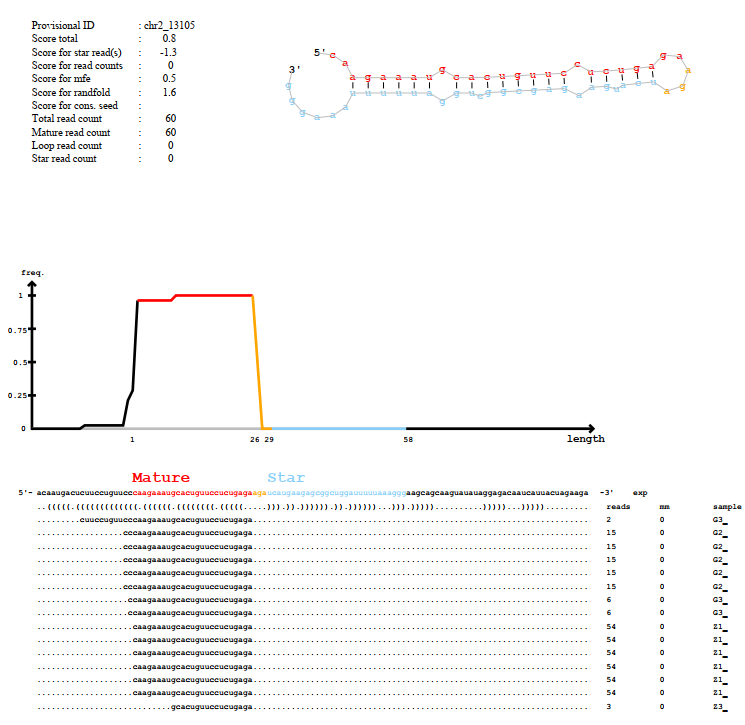

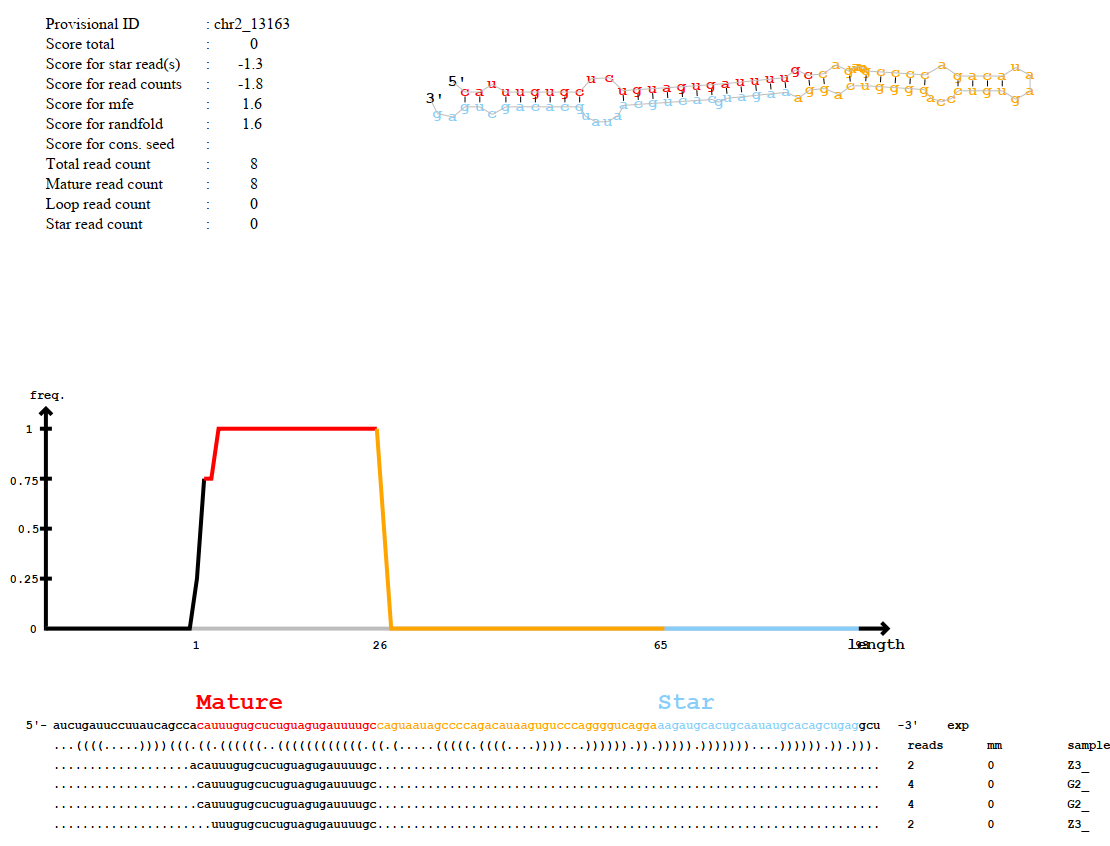

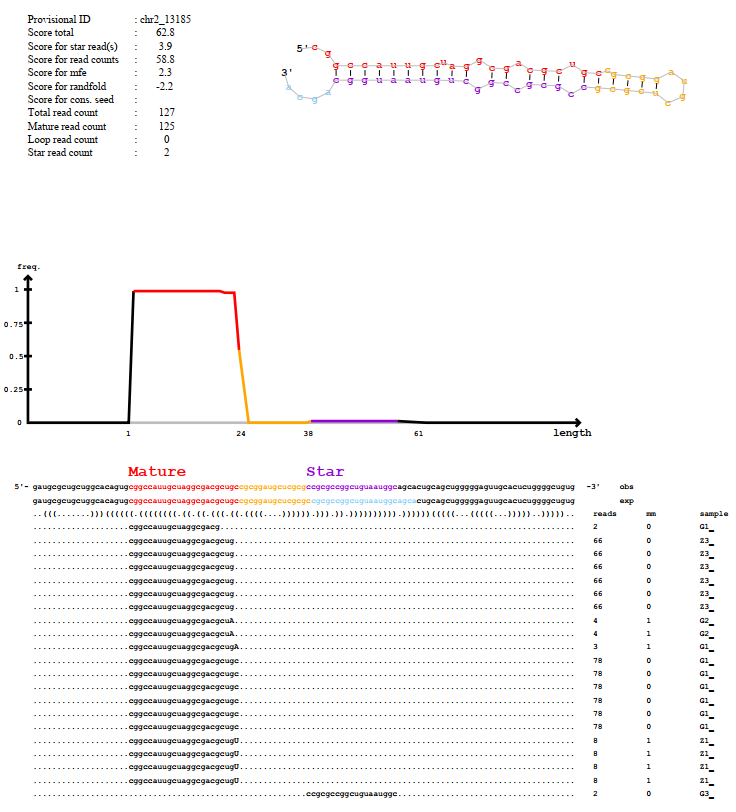

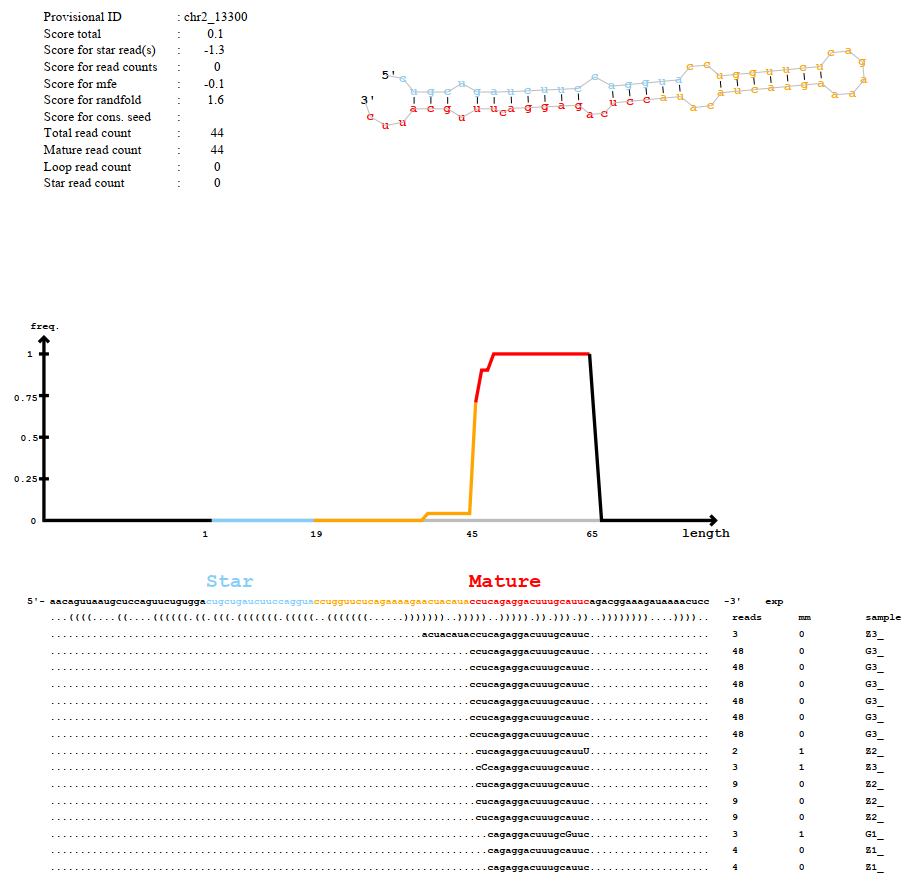

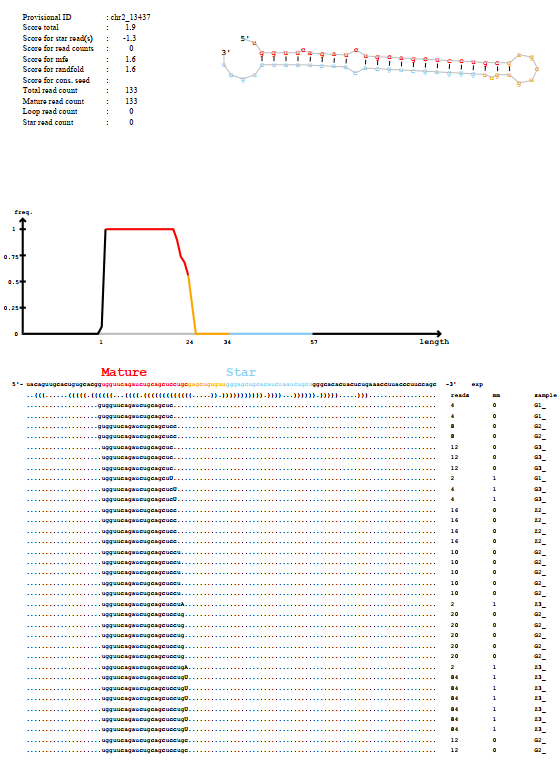


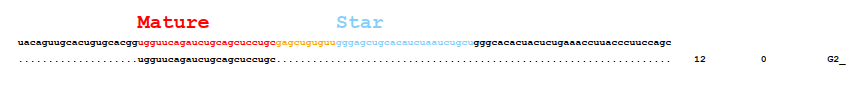

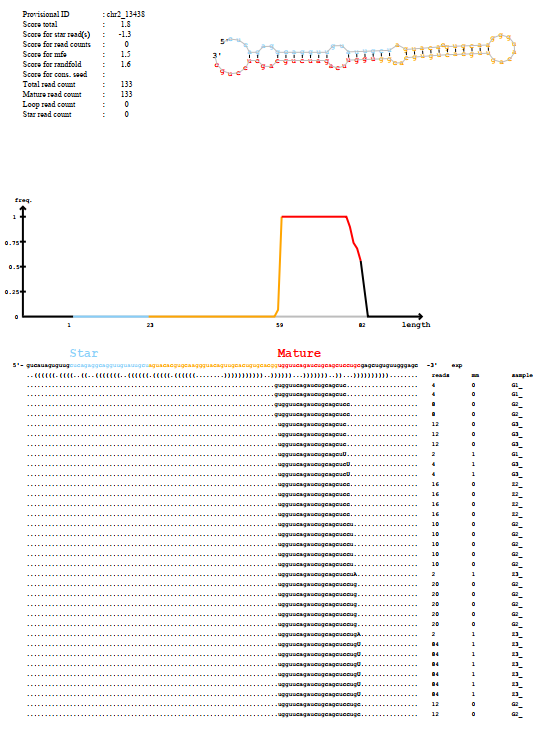

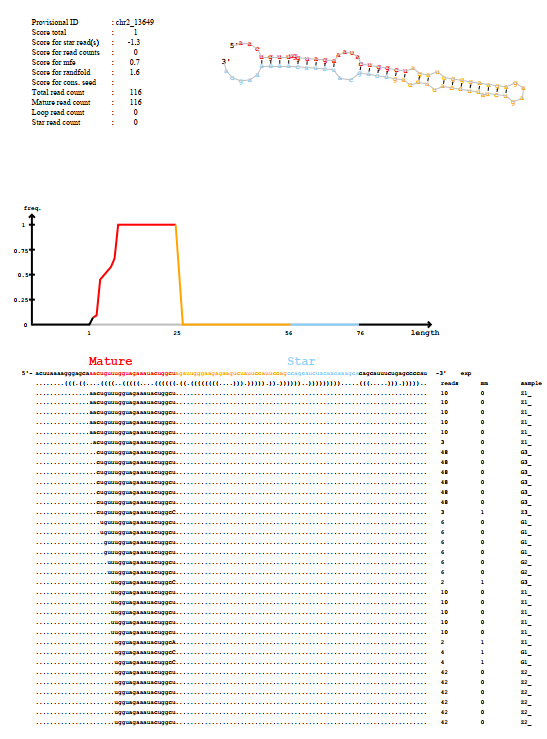

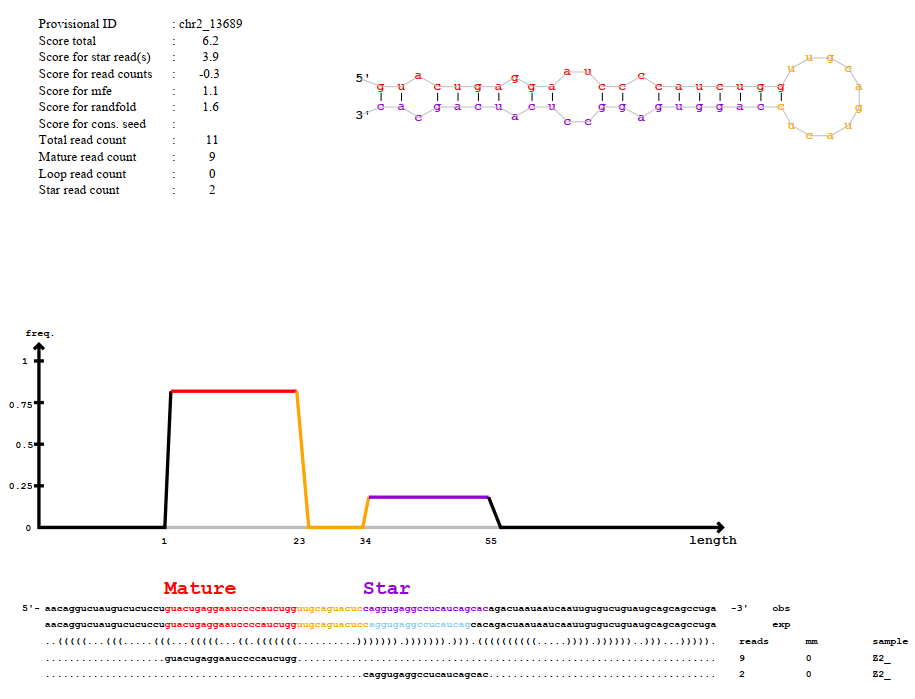

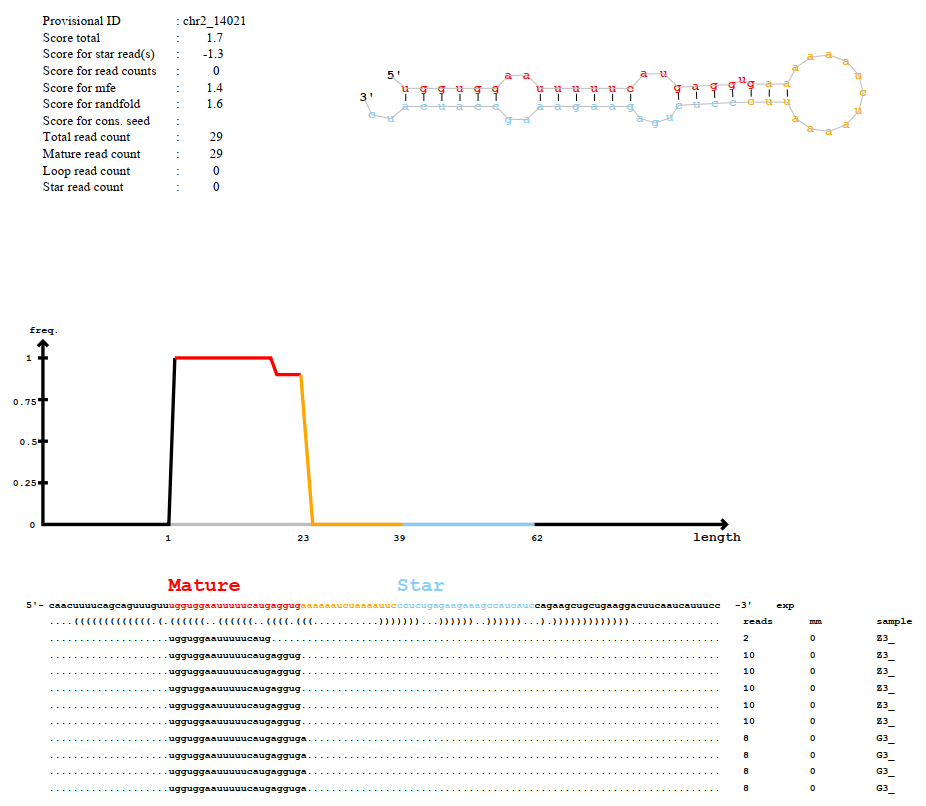

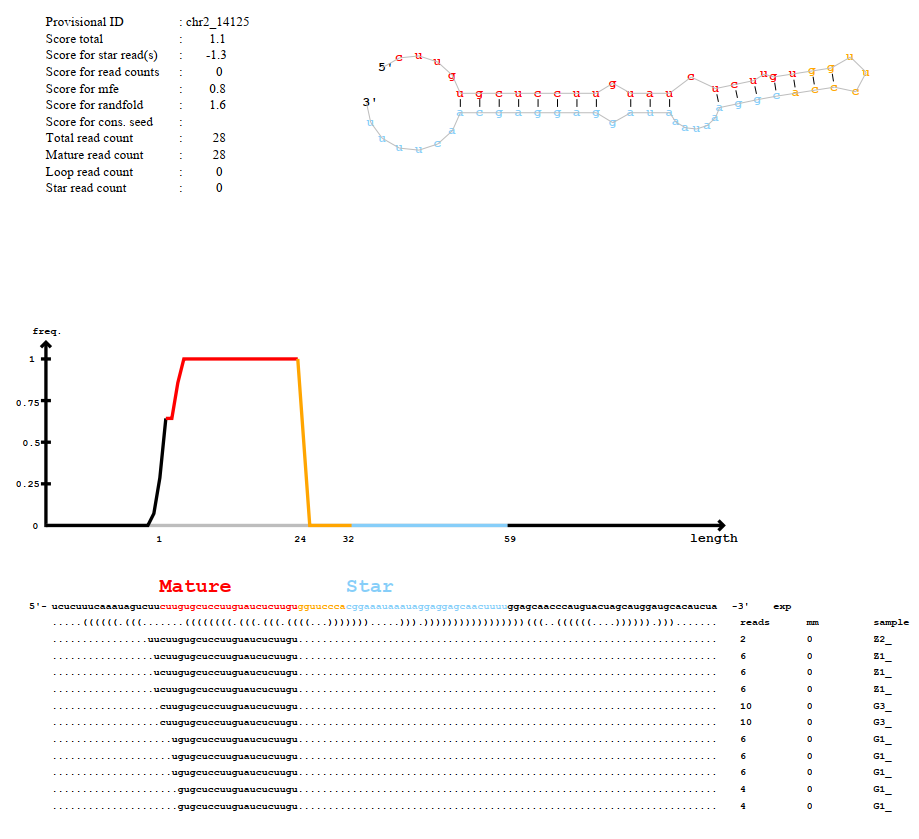

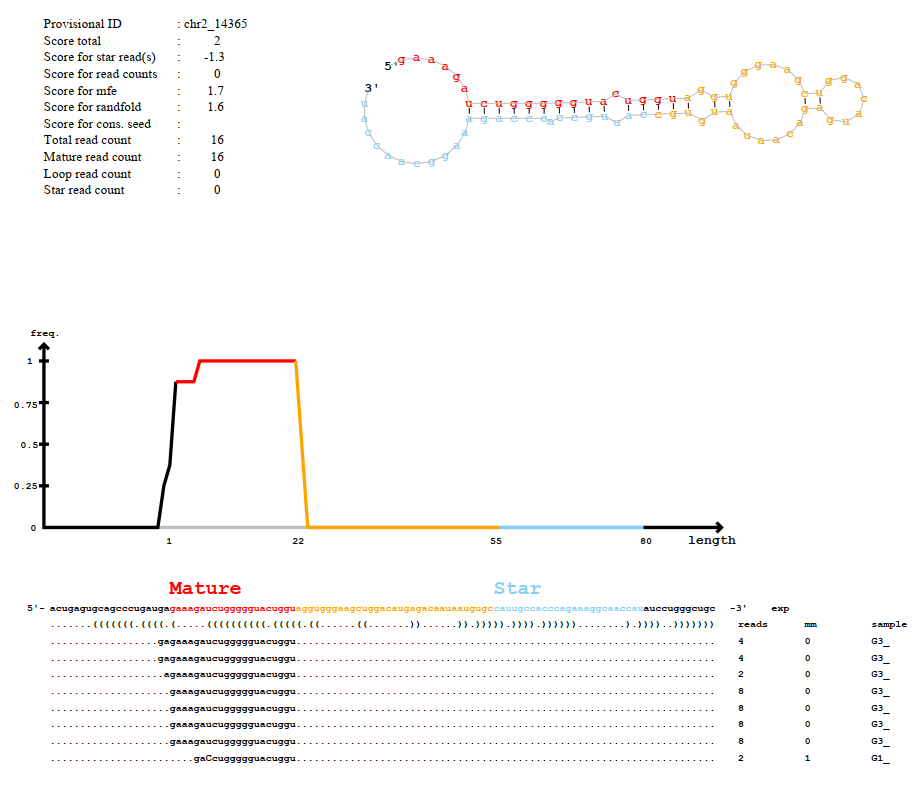

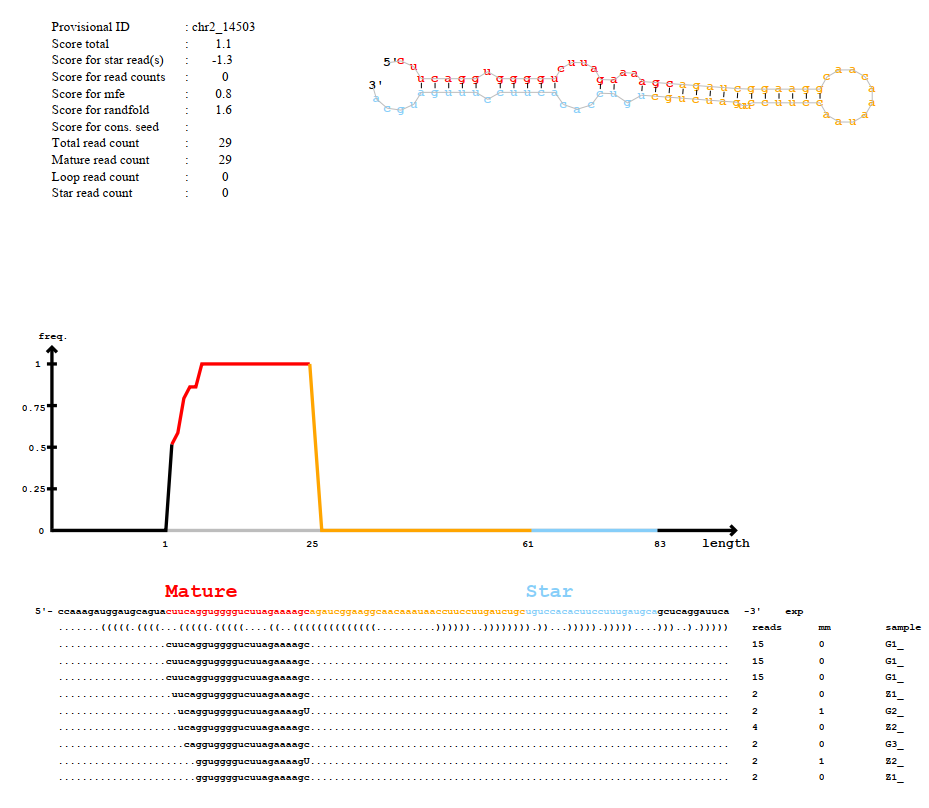

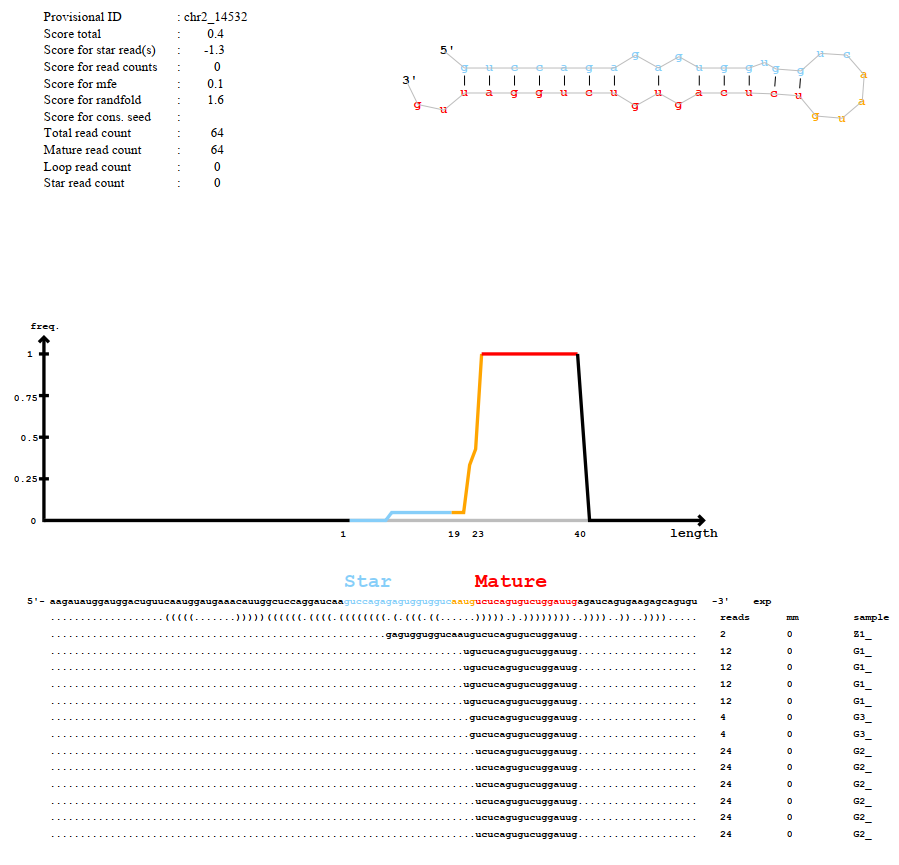

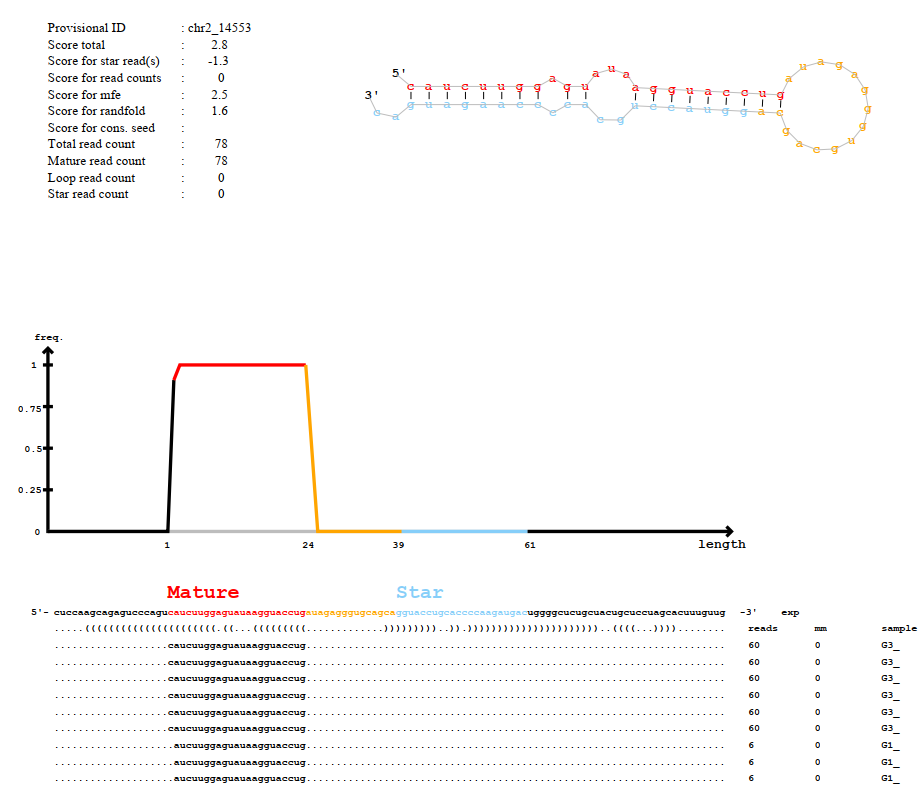

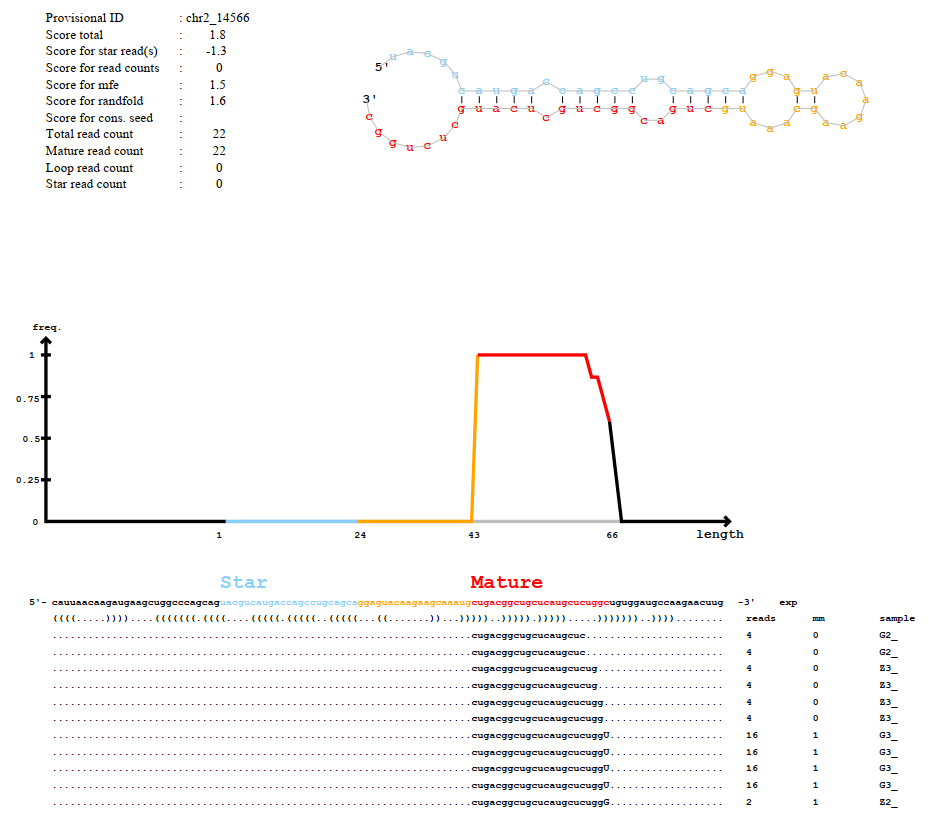

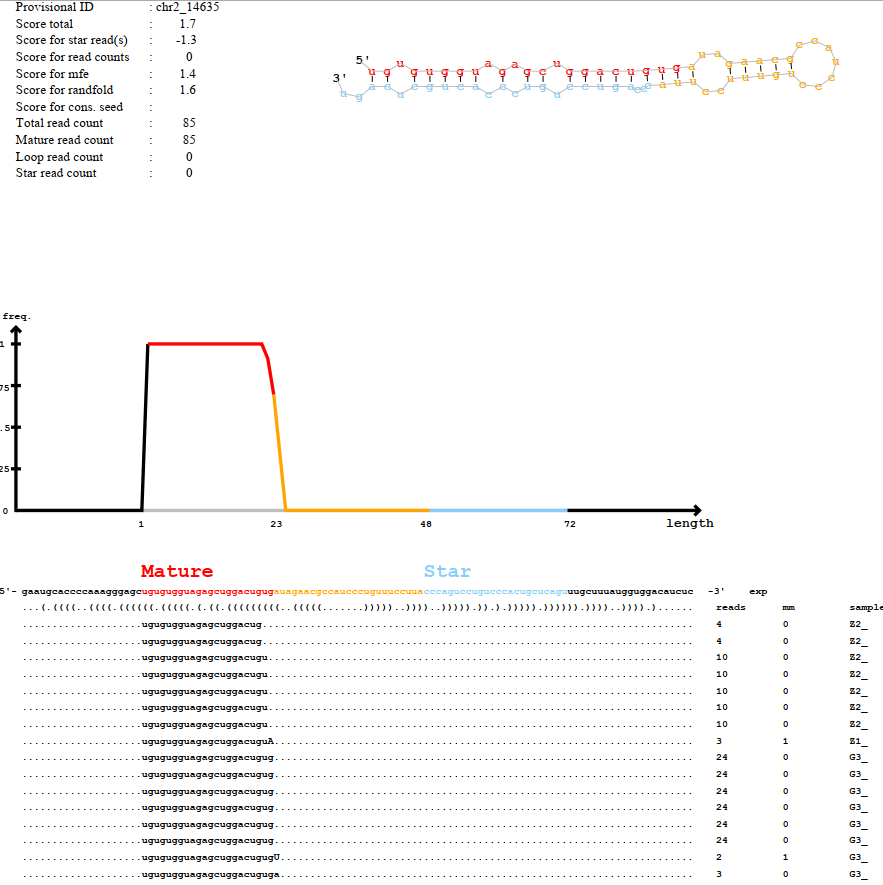

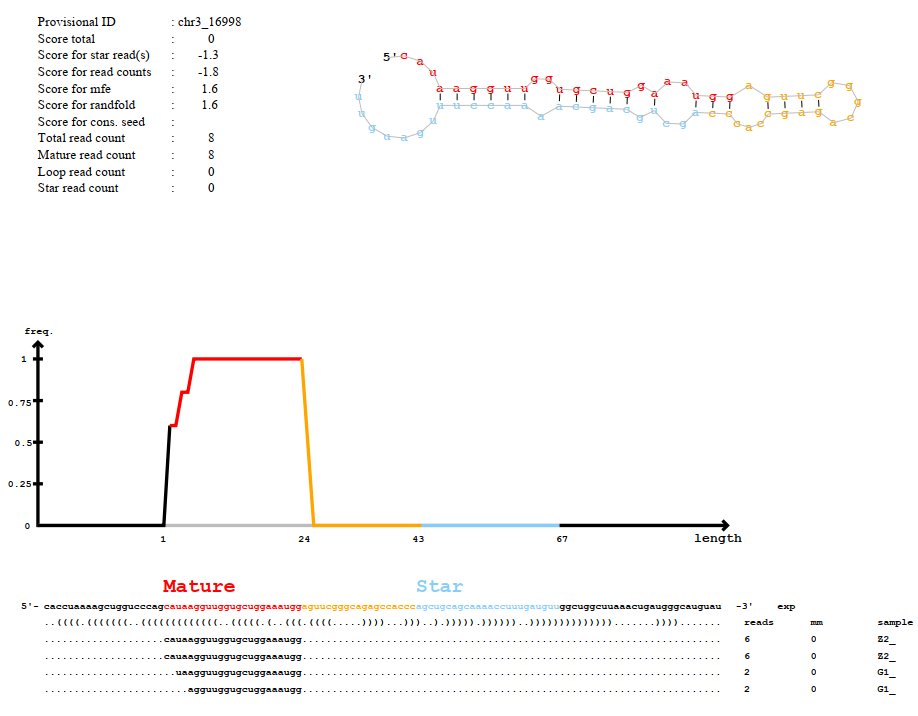

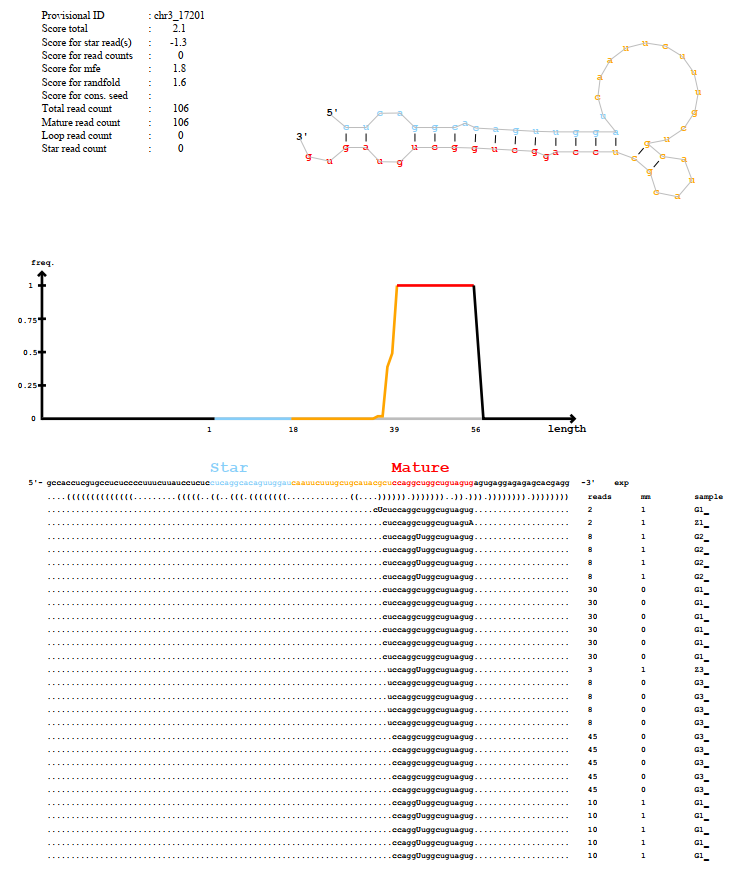

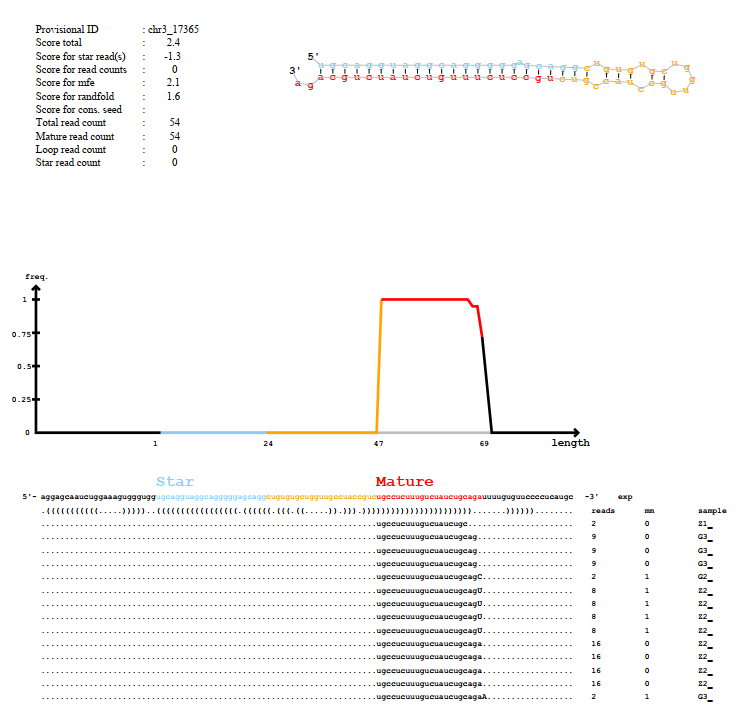

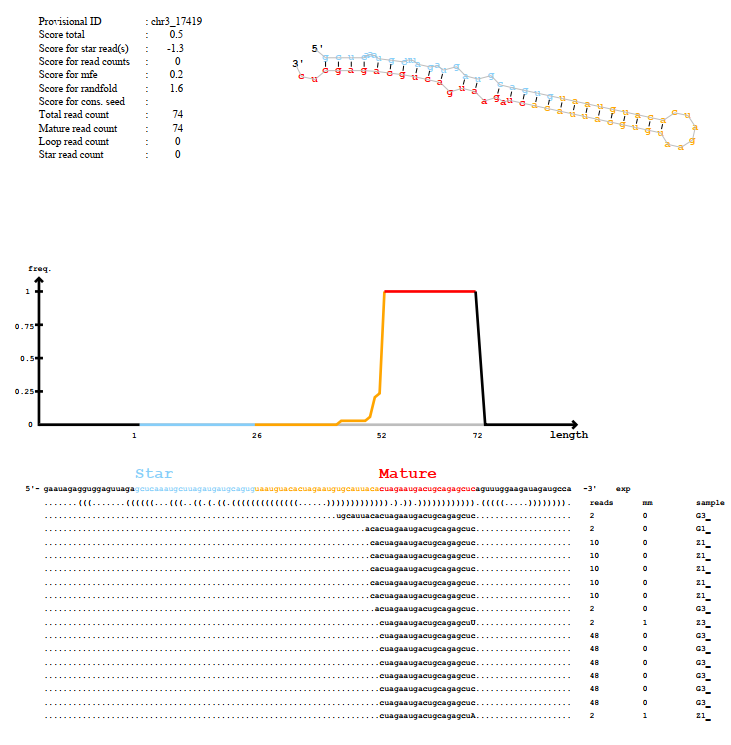

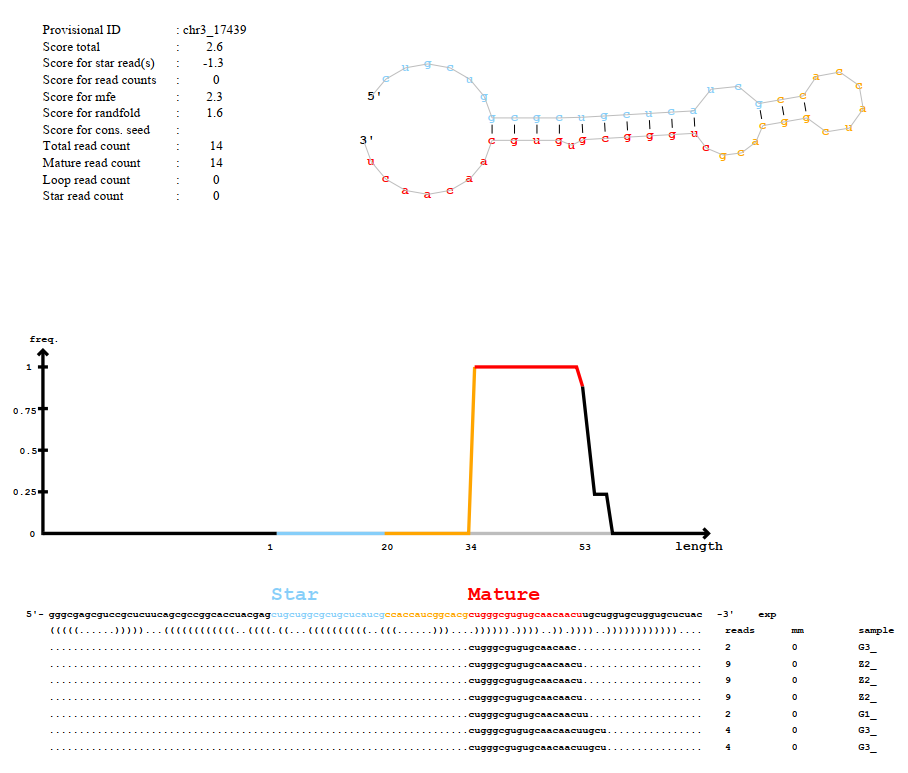

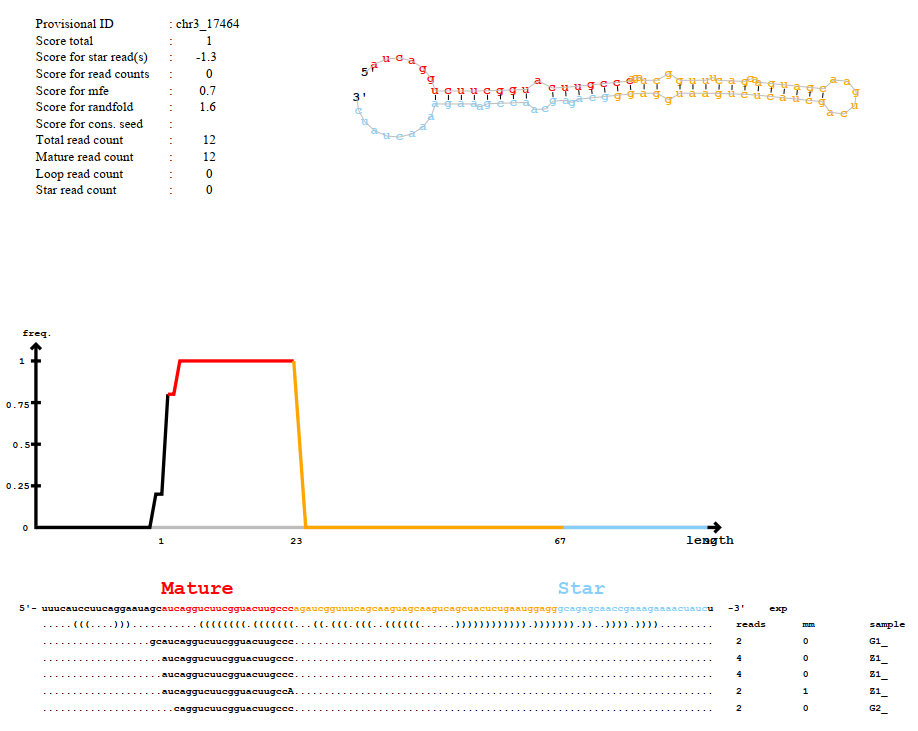

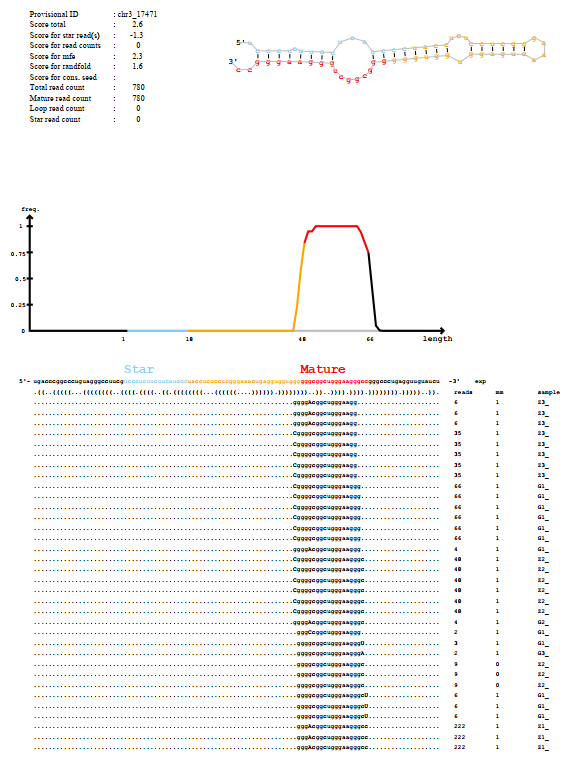

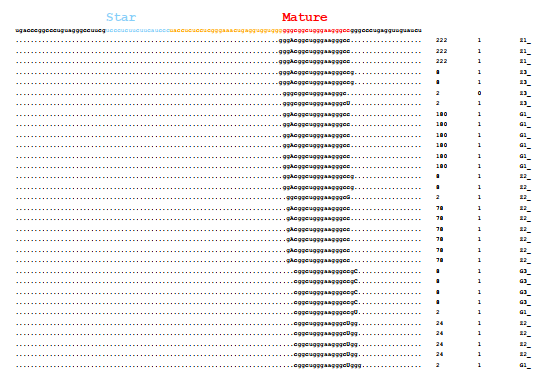

Supplement: Supplementary file 1 — FigS1 [file JCMM-24-6178-s001.docx]
