# Supplementary material for: Analysis of deep sequencing exosome‐microRNA expression profile derived from CP‐II reveals potential role of gga‐miRNA‐451 in inflammation
Source: J Cell Mol Med. 2020 Apr 19;24(11):6178–90. doi: 10.1111/jcmm.15244 (PMC7294135; doi:10.1111/jcmm.15244)
Supplement: Supplementary file 2 — TableS1 [file JCMM-24-6178-s002.docx]

**Supplementary Table 1: Sequences of primers used for RT-qPCR**

| **Name** | **Primer sequence( (5ʹ-3ʹ)** | **Accession No.** |
| --- | --- | --- |
| RT-gga-let-7d | CTCAACTGGTGTCGTGGAGTCGGCAATTCAGTTGAGACTATGCA | MIMAT0001161 |
| gga-let-7d-F | CTGGTAGGAGAGGTAGTGGGTTGC |  |
| gga-let-7d-R | ACTGGTGTCGTGGAGTCGGC |  |
| RT-gga- miR-451 | CTCAACTGGTGTCGTGGAGTCGGC  AATTCAGTTGAGAAACTCAG | MIMAT0003775 |
| gga-miR-451-F | GTAGGAAACCGTTACCATTACTGACTC |  |
| gga-miR-451-R | ACTGGTGTCGTGGAGTCGGC |  |
| RT-gga- miR-133c-3p | AACTGGTGTCGTGGAGTCGGCAATTCAGTTGAGGCAGCTGG | MIMAT0001176 |
| gga-miR-133c-3p-F | CTGGTAGGTTGGTCCCCTTCAA |  |
| gga-miR-133c-3p-R | ACTGGTGTCGTGGAGTCGGC |  |
| RT-gga- miR-233 | CTCAACTGGTGTCGTGGAGTCGGCAATTCAGTTGAGGGGGTATT | MIMAT0001140 |
| gga-miR-223-F | GTGTCAGTTTGTCAAATACCCCCTC |  |
| gga-miR-223-R | ACTGGTGTCGTGGAGTCGGC |  |
| RT-gga-miR-193a-3p | CTCAACTGGTGTCGTGGAGTCGGCAATTCAGTTGAGACTGGGAC | MIMAT0007740 |
| gga-miR-193a-3p-F | GGTAGGAACTGGCCTACAAAGTCC |  |
| gga-miR-193a-3p-R | ACTGGTGTCGTGGAGTCGGC |  |
| RT-gga-miR-33-5p | CTCAACTGGTGTCGTGGAGTCGGCAATTCAGTTGAGGCAATGCA | MIMAT0001100 |
| gga-miR-33-5p-F | GGGTGCATTGTAGTTGCATTGC |  |
| gga-miR-33-5p-R | ACTGGTGTCGTGGAGTCGGC |  |
| RT-gga-miR-460b-5p | CTCAACTGGTGTCGTGGAGTCGGCAATTCAGTTGAGCACACAGC | MIMAT0007326 |
| gga-miR-460b-5p-F | GTCCTCATTGTACATGCTGTGTGC |  |
| gga-miR-460b-5p-R | ACTGGTGTCGTGGAGTCGGC |  |
| RT-gga-miR-202-5p | CTCAACTGAATTGCCGACTCCACGACACCAGTTGAGAAAGAAGT | MIMAT0003354 |
| gga-miR-202-5p-F | CTGGTAGGTTTCCTATGCATATACTTCT |  |
| gga-miR-202-5p-R | ACTGGTGTCGTGGAGTCGGC |  |
| gga-5s-rRNA-F | CCATACCACCCTGGAAACGC |  |
| gga-5s-rRNA-R | TACTAACCGAGCCCGACCCT |  |
| YWHAZ -F | AAAATGTTGTAGGAGCCCGTAGG | NM_001031343.1 |
| YWHAZ -R | TTGCTTTCTGCTTGCGAAGC |  |
| GAPDH-F | GAGGGTAGTGAAGGCTGCTG | NM-204305 |
| GAPDH-R | CACAACACGGTTGCTGTATC |  |
