# Supplementary material for: Analysis of deep sequencing exosome‐microRNA expression profile derived from CP‐II reveals potential role of gga‐miRNA‐451 in inflammation
Source: J Cell Mol Med. 2020 Apr 19;24(11):6178–90. doi: 10.1111/jcmm.15244 (PMC7294135; doi:10.1111/jcmm.15244)
Supplement: Supplementary file 3 — TableS2 [file JCMM-24-6178-s003.docx]

**Supplementary Table 2: Sequences of RNA oligonucleotides**

| **Name** | **Sequences (5ʹ-3ʹ)** |
| --- | --- |
| gga-miR-451 mimics | AAACCGUUACCAUUACUGAGUUUACUCAGUAAUGGUAACGGUUUUU |
| gga-miR-451 mimics NC | UUCUCCGAACGUGUCACGUTTACGUGACACGUUCGGAGAATT |
