# Supplementary material for: Analysis of deep sequencing exosome‐microRNA expression profile derived from CP‐II reveals potential role of gga‐miRNA‐451 in inflammation
Source: J Cell Mol Med. 2020 Apr 19;24(11):6178–90. doi: 10.1111/jcmm.15244 (PMC7294135; doi:10.1111/jcmm.15244)
Supplement: Supplementary file 4 — TableS3 [file JCMM-24-6178-s004.docx]

**Supplementary Table 3: Quality and data filtering of small RNA sequencing data**

| **Sample** | **MG1** | **MG2** | **MG3** | **NC1** | **NC2** | **NC3** |
| --- | --- | --- | --- | --- | --- | --- |
| Total reads 16170277 14223175 13992025 13682750 13785537 13435656  Bases(bp) 808513850 711158750 6996012250 684137500 689276850 671782800  length 50 50 50 50 50 50  Q20(%) 99.21 99.23 99.21 99.14 99.21 99.21  Q30(%) 99.38 99.43 98.4 98.21 98.39 98.39  GC(%) 51.56 51.56 51.71 52.56 52.17 52.06 | | | | | | |
| Clean reads 13102883 13001479 13000918 13000094 12611054 11975719  Clean rate(%) 81.03 91.41 92.92 95.01 91.48 89.13 | | | | | | |
