# Supplementary material for: Analysis of deep sequencing exosome‐microRNA expression profile derived from CP‐II reveals potential role of gga‐miRNA‐451 in inflammation
Source: J Cell Mol Med. 2020 Apr 19;24(11):6178–90. doi: 10.1111/jcmm.15244 (PMC7294135; doi:10.1111/jcmm.15244)
Supplement: Supplementary file 5 — TableS4 [file JCMM-24-6178-s005.docx]

| **Supplementary Table 4: Novel mature miRNA sequence and read counts (the novel miRNAs were named by their chromosomal location)** | | | |
| --- | --- | --- | --- |
| **miRNA_ID** | **Mature** | **Total_score** | **Count** |
| chrZ_28228 | acgggacggggcgggacggcgcu | 3.3 | 24 |
| chr4_22197 | uuuccaaccuucgugauucuga | 2.7 | 493 |
| chrUn_AADN03021535_30549 | cugagugacugaucccugcag | 2.6 | 74 |
| chr2_10877 | cuuuuaaucaagguuucugu | 1.1 | 49 |
| chr2_10654 | aggaggaggaggaggaggaggagg | 2.5 | 398 |
| chr1_4662 | acuucaggauccaaggauuu | 2.3 | 108 |
| chr1_1115 | gaggagagcauuagagagguua | 1.8 | 21 |
| chr1_3807 | uaagugcuggccguuguccucu | 2.8 | 280 |
| chr1_4078 | cugcacacugggaagaggggacaga | 0.6 | 9 |
| chr3_19782 | cagcgcugggcagugcacugc | 37 | 69 |
| chrUn_AADN03026535_30610 | ugugcgguugcagaagaagaag | 5528 | 10840 |
| chrUn_AADN03020043_31535 | ugugcgguugcagaagaagaag | 5528 | 10840 |
| chrUn_AADN03014885_30692 | ugugcgguugcagaagaagaag | 5528 | 10840 |
| chrUn_AADN03020858_30545 | ugugcgguugcagaagaagaag | 5528 | 10840 |
| chrUn_AADN03016324_30659 | ugugcgguugcagaagaagaag | 5528 | 10840 |
| chrUn_AADN03025781_31402 | ugugcgguugcagaagaagaag | 5514.1 | 10813 |
| chrUn_AADN03011747_30643 | ugugcgguugcagaagaagaag | 5528 | 10840 |
| chrUn_AADN03018731_31460 | ugugcgguugcagaagaagaag | 5528 | 10840 |
| chrUn_AADN03012872_30525 | ugugcgguugcagaagaagaag | 5528 | 10840 |
| chr19_10289 | ugugcgguugcagaagaagaag | 5528 | 10840 |
| chrUn_AADN03018872_31439 | ugugcgguugcagaagaagaag | 5514 | 10813 |
| chrUn_AADN03018260_30245 | ugugcgguugcagaagaagaag | 5528 | 10840 |
| chrUn_AADN03019802_31390 | ugugcgguugcagaagaagaag | 5528.1 | 10840 |
| chr3_19458 | uucuugcuagcuagcuucugugu | 6.8 | 10 |
| chr19_10241 | uugguggcugcaugcucucagc | 2243.8 | 4399 |
| chr1_2001 | uagaaucguggaauggcuuggg | 2.5 | 59 |
| chrZ_28624 | cauccccauuccacuccuagca | 2.2 | 9839 |
| chr4_JH375168_random_22258 | uucagggugcugcagagagc | 2.6 | 31 |
| chr1_3868 | ucuggccuaagagcucugccuguuc | 1 | 332 |
| chr23_15731 | cuacacugugcagcagcgaugc | 1.5 | 10 |
| chrUn_AADN03017741_30715 | ccgggcagggccgagcugagc | 87.1 | 174 |
| chrUn_AADN03020348_30830 | ccgggcagggccgagcugagc | 87.2 | 174 |
| chr1_1813 | ucauguuugugacuguuaac | 1 | 99 |
| chr3_19626 | aggggagaaggucaacugugcu | 2.9 | 29 |
| chr8_26240 | ccucagacaacagugugacuguaac | 2.2 | 27 |
| chr1_1691 | uguucacggccuguuugaguugc | 0.9 | 59 |
| chr4_21558 | ccaaagguggcucgcggaacug | 2.7 | 14 |
| chr20_14655 | uauuugggcuguaggcagaggac | 1.8 | 30 |
| chr20_15056 | uucucagguuagacaguccucg | 2.4 | 162 |
| chr2_14125 | cuugugcuccuuguaucucuugu | 1.1 | 28 |
| chrUn_JH376257_31421 | ggggcugagggagaggac | 2.3 | 888 |
| chr3_19044 | gcuuggcaugggacuggggaucucc | 2 | 20 |
| chr5_23545 | cuguucuuugaauucugu | 0.7 | 61 |
| chr15_8911 | uaggagaaccucugagaaucuuc | 1.7 | 18 |
| chr15_8913 | uaggagaaccucugagaaucuuc | 1.7 | 18 |
| chr3_18623 | cucaacugcagcugggaugaau | 1.6 | 43 |
| chrUn_AADN03018911_30995 | aacuggagagcugcuguugc | 2.3 | 12 |
| chr2_10744 | cacguguccugaggauucucucc | 0 | 10 |
| chrZ_27959 | cauaucuggacuauaggaacaac | 0.8 | 94 |
| chr5_23396 | ucucggucucggucugcaucu | 2 | 31 |
| chr20_14850 | aaccgcagcggcuguuggacgug | 2.1 | 100 |
| chr13_7846 | gcucuucuguaggcagg | 2.1 | 116 |
| chr21_15456 | agggacugcaccugauccagc | 2.2 | 66 |
| chr5_23175 | ggacggggcugggauccagg | 2.6 | 184 |
| chr12_7158 | uggugagaagcgugcuguggagc | 38 | 80 |
| chr5_23245 | ugagggaacucugguauugagaau | 0.1 | 9 |
| chr11_AADN03010436_random_7084 | ugccccuaugccagugcucagc | 67.7 | 137 |
| chr1_3518 | uuguguagugguucucugccuug | 2.5 | 19 |
| chrZ_28804 | agaagaggacgucuguguggcu | 2.3 | 20 |
| chr2_13185 | cggccauugcuaggcgacgcugc | 62.8 | 127 |
| chr2_14365 | gaaagaucuggggguacuggu | 2 | 16 |
| chr3_17471 | gggcggcugggaagggcc | 2.6 | 780 |
| chr9_27375 | caaguugggccugauccugug | 0.5 | 8 |
| chr2_13438 | ugguucagaucugcagcuccugc | 1.8 | 133 |
| chr2_13437 | ugguucagaucugcagcuccugc | 1.9 | 133 |
| chrUn_AADN03013128_30771 | guaagugugggaugcaggaa | 2.3 | 174 |
| chr11_AADN03010444_random_7082 | guaagugugggaugcaggaa | 2.3 | 174 |
| chr11_6546 | guaagugugggaugcaggaa | 2.3 | 174 |
| chrZ_29836 | gcugggaucuguagaaaaggc | 2.3 | 11384 |
| chr22_15555 | aagaggauugaauugcaagacu | 0.8 | 88 |
| chrUn_AADN03021587_31538 | acuggcacugugucauuggcac | 2.2 | 95 |
| chr1_3758 | guagggcuguuugucugaa | 1.3 | 10 |
| chr19_10191 | aagcuugccacugaagaacugc | 29.2 | 55 |
| chr2_13076 | cuuagaaucacugaaucauua | 1.1 | 14 |
| chrZ_28633 | cgaggggcggcggcggcgg | 3 | 46 |
| chr13_8215 | uaguucagcugucugugaaugagg | 139.9 | 271 |
| chr4_20978 | cuuuauguuggacuuuaagcu | 2.2 | 119 |
| chr1_3986 | cucuccaggaacucucugguac | 1 | 16 |
| chr15_8770 | ccgucgggcccgggggc | 2.8 | 861 |
| chrZ_28229 | ugcuucaaaagacuuagggugcu | 54.3 | 114 |
| chr1_2495 | aaagaaucauaggauugguc | 1.6 | 32 |
| chr28_16792 | gaggaggacgcagccaugugc | 30.4 | 56 |
| chr1_3383 | acucgcgucgggcgcggggcg | 3.1 | 27 |
| chr10_6486 | uuccugggcugcuuugaagc | 2.6 | 16 |
| chr2_13649 | aacuguuugguagaaauacuggcu | 1 | 116 |
| chr17_9649 | cagccuggagaggcagcugucc | 16.6 | 38 |
| chr2_14503 | cuucagguggggucuuagaaaagc | 1.1 | 29 |
| chr19_10202 | ucuggaauguuuauagugaggcu | 1.4 | 25 |
| chr17_9471 | uguggugcuguucugugcugggga | 2.8 | 28 |
| chrUn_JH375968_30860 | cugggacugaaacuccagc | 2 | 955 |
| chr1_2981 | guguggagaggcugugaac | 1.7 | 11 |
| chr26_16410 | gacuccgguggaaugaaggac | 593.8 | 1163 |
| chr2_14635 | ugugugguagagcuggacugug | 1.7 | 85 |
| chrUn_AADN03016005_30417 | gggggcagagcagaacc | 2.4 | 303 |
| chr4_21626 | ccuuguggguucugcuaguaug | 1.9 | 41 |
| chr4_20156 | cauugggagggcuuuaaacu | 1.3 | 117 |
| chr1_2238 | cugggaagacugcugaag | 1.7 | 290 |
| chr5_22771 | uaacuagaaggaaaugaauuc | 1.5 | 67 |
| chr5_23044 | uccgggacggcugggaa | 1.7 | 6248 |
| chr20_14826 | cuguaggaggugagaacc | 2.1 | 124 |
| chr1_4765 | cugaugugugagcgcugugcacu | 2.3 | 12 |
| chr1_5166 | uauuucuguggaaacugcaac | 1.2 | 151 |
| chrUn_JH375727_30461 | uuuagugugauaauggcguu | 2.2 | 42 |
| chrZ_28833 | uuuagugugauaauggcguu | 1.9 | 42 |
| chr1_1580 | ccuguucugugacugguggggug | 1.4 | 89 |
| chrUn_AADN03012082_30617 | aauggaacuccaauggaacucc | 2.3 | 52 |
| chr1_4081 | acuuggucuuguugaacuucaua | 1.5 | 113 |
| chr4_21950 | cucugaggaucuacgcucuugu | 1.7 | 19 |
| chr28_16886 | aggacaagggcaggggcagcucu | 0.6 | 8 |
| chr1_2665 | cugcaaggacugcaagacc | 0.8 | 9 |
| chr11_6522 | ugcugaagcacuccggcacgg | 24.9 | 46 |
| chr1_2190 | gugccugcugugagugaaauug | 13.7 | 23 |
| chr18_9886 | ccugcucugaggucugugugc | 2.1 | 82 |
| chr3_17849 | ucuugguugugacucuc | 1.4 | 14 |
| chrUn_AADN03012562_31795 | uggucuguacugguccguacug | 1.8 | 23 |
| chr18_9729 | cuccuacaggauuuggucucug | 1.6 | 39 |
| chr3_17439 | cugggcgugugcaacaacu | 2.6 | 14 |
| chr4_20132 | augggugcuggagaacugggcugg | 2.4 | 12 |
| chr1_1947 | cugccaguagucucuga | 1.3 | 31 |
| chrZ_27881 | ugguuuggguuggaagggacuuuga | 1.8 | 46 |
| chr15_9183 | uggagccucugucccaagggug | 2.6 | 40 |
| chr3_18070 | cucgcuguaagcaccuuuc | 0.8 | 11 |
| chr17_AADN03010797_random_9705 | guuggggcugcccuugguccc | 1.5 | 10 |
| chr25_16150 | cgugucucugggggccgucugu | 2.5 | 54 |
| chr2_13019 | gcucaaagaggagggcuggugagca | 1.7 | 79 |
| chrZ_28342 | aaguuguguacgugagccagug | 2.3 | 22 |
| chr14_8338 | ccaaaagugucgggaaggga | 1.6 | 74 |
| chr23_15727 | auucugugguucugacuccugc | 2.4 | 26 |
| chr5_23176 | ggacggggcugggauccagg | 2.1 | 131 |
| chr28_16841 | uucuucccugccggacgu | 2.3 | 21 |
| chr3_17834 | ucuugcuggagaucucugugu | 34.1 | 73 |
| chr17_9581 | auugggggcucugugggacc | 1 | 10 |
| chrUn_AADN03021961_31393 | aucagggcuccacagcugaacg | 14.7 | 35 |
| chr25_JH375218_random_16226 | uccccuauggggcucuggg | 1.8 | 10 |
| chr1_5658 | agcaguuggacucgaugauccuugu | 2 | 21 |
| chr27_16653 | acccauggcaaugagcaaaccug | 16.5 | 29 |
| chr19_10325 | uggugcucuguaggaacguaugg | 1.5 | 10 |
| chr2_12612 | ugggcugggcuugcugcgugggc | 2.4 | 51 |
| chr23_15615 | cagcucuguagaucugugugc | 2.1 | 177 |
| chr3_17201 | ccaggcuggcuguagug | 2.1 | 106 |
| chr7_25266 | cugcaguugcggguuuacgucugg | 1.2 | 20 |
| chr21_15300 | auguggcagggcagaggcu | 2.6 | 26 |
| chr11_6552 | agcgcggcgagcuggcggcugu | 9.6 | 15 |
| chr3_18433 | ugggcuugauugggcaugauu | 0 | 8 |
| chr2_11462 | uugugccugcauaaacugacu | 1.9 | 213 |
| chrW_JH375234_random_27739 | aagggcaaacccgaugggc | 2.5 | 217 |
| chrUn_JH375743_30499 | aucgaucaguggggcagcccc | 1 | 7 |
| chr10_6139 | ccacucugugugccugcccacc | 2.7 | 20 |
| chr3_18033 | cggacgaggcuucccgcgcugu | 3.2 | 48 |
| chr9_27206 | ugaggugcucugagcugcuugggu | 2.6 | 16 |
| chr4_20975 | caucugugggucugucaggc | 1.2 | 47 |
| chr2_14021 | ugguggaauuuuucaugaggug | 1.7 | 29 |
| chr12_7550 | acugguuggguagagcucuac | 1.2 | 26 |
| chr8_26123 | ccgagcgagcucugcagagccu | 13.4 | 31 |
| chrUn_AADN03019634_30649 | guacuggucuguacuggucugu | 2.6 | 19 |
| chr11_6908 | gccagugaacuccugacaag | 2.1 | 70 |
| chrZ_29815 | agcugugcucuguuccucaguuc | 48.5 | 93 |
| chrUn_AADN03021449_30588 | ugugccaugcagugcugugugc | 24.8 | 45 |
| chrUn_AADN03021449_30587 | ugugccaugcagugcugugugc | 2.8 | 43 |
| chr4_21498 | uggaccgcccgcuugguuagagu | 2.1 | 150 |
| chr4_19904 | ccugccugaccuggauguacagc | 2.3 | 23 |
| chr1_4549 | uaaggccccuuacuuugcucu | 2.2 | 14 |
| chr3_18048 | agcagaggaaaugggcuguucugu | 0.7 | 9 |
| chr4_21290 | ucugaacuucuugaaccacag | 1 | 14 |
| chr3_19652 | ucucauucugacuguguuc | 1 | 18 |
| chr4_19928 | ccugcugagccaggacgcagc | 2 | 19 |
| chr1_827 | cugagcguggauguggacgug | 2.2 | 63 |
| chr1_179 | ugagagcagcccacgcugagc | 2.7 | 12 |
| chr1_180 | ugagagcagcccacgcugagc | 2 | 12 |
| chr5_22649 | acugggacuccaugggcagg | 1.1 | 10 |
| chr4_20302 | ugagaccuucugcacacagcug | 1.6 | 11 |
| chrZ_29544 | caggccuggacacagaacucc | 1.7 | 163 |
| chr26_16342 | cugcuugcccaggaugggggu | 29.9 | 55 |
| chr2_12841 | aacagcucaacgucuggauggaga | 2.3 | 41 |
| chr10_6236 | ugcagugacgucucuucccc | 1.7 | 10 |
| chr18_10023 | ccugcuccagaagccuugucugg | 0.5 | 8 |
| chr6_24828 | uucucugaaagcuguuuccuc | 1.6 | 12 |
| chr2_12688 | cccguggggcccugaugug | 0.5 | 9 |
| chr3_18103 | auggcuguucuagcaguagauc | 2.2 | 336 |
| chrUn_AADN03015019_31571 | ccuguuggugucgggacuccg | 0.9 | 6 |
| chr15_9181 | ggacaggaugaaacucucagc | 0.6 | 8 |
| chrUn_AADN03021459_31803 | uggggacaccacggggacacug | 2.4 | 13 |
| chr1_3069 | uacugaggacuccacaucugg | 2.1 | 39 |
| chr9_27338 | ucggucugcauccacucugacu | 49.6 | 95 |
| chr2_12291 | agaugcacugggcucaggaau | 14.5 | 25 |
| chr19_10404 | ucccaguggcucuguaguguugu | 2 | 27 |
| chr17_9290 | acugauucagacuggagaaggg | 2.7 | 35 |
| chr1_5087 | agccugcugagagugaaauuga | 1.7 | 10 |
| chr4_20433 | cauuuggagggcuuuaaacu | 1.7 | 544 |
| chr11_6953 | ugguagaauuccucaagcac | 1.5 | 47 |
| chr18_10030 | cuccagcagcagcccagc | 0.7 | 9 |
| chr3_17464 | aucaggucuucgguacuugccc | 1 | 12 |
| chr13_7880 | ucaagggcugauagacauuc | 1.7 | 52 |
| chrUn_JH376078_31069 | uggggcugcagggcugggagaug | 2.7 | 50 |
| chr23_15781 | gugcugcggcgcggaacgguuc | 2 | 10 |
| chr1_4902 | ucggcccugggagugucguuc | 2.7 | 22 |
| chr5_23073 | cgugguuggguugugucagg | 2.2 | 12 |
| chr5_22686 | ccgcucugcucugcucuccc | 8.2 | 13 |
| chrLGE22C19W28_E50C23_27525 | ugagugcagaacuggacgug | 2.3 | 151 |
| chr2_13105 | caagaaaugcacuguuccucugaga | 0.8 | 60 |
| chrUn_AADN03012208_30379 | gugaggcugugcuccccccga | 2.4 | 61 |
| chr2_14566 | cugacggcugcucaugcucuggc | 1.8 | 22 |
| chrZ_28692 | cugguuuuguaucuugug | 1.7 | 19 |
| chr14_8726 | cagaacuacugaccggug | 2 | 223 |
| chr17_9607 | guagggacggggcagcgugcu | 4 | 12 |
| chr26_16392 | cggcuguaugacuucuguag | 2.1 | 12 |
| chr8_26267 | acugcugccggcgcuccuggga | 2.7 | 15 |
| chrUn_JH375654_30394 | ucuggcugcucagugcucugc | 2.5 | 47 |
| chr5_23164 | acugggagaucggauucggagcc | 2 | 73 |
| chr8_26341 | caggaacaacugaagugaauu | 1.5 | 45 |
| chr3_17365 | ugccucuuugucuaucugcaga | 2.4 | 54 |
| chr2_10592 | caggaggcugauccgagggcu | 2.8 | 20 |
| chr8_26127 | uucucagccuccauccaugguu | 0.4 | 8 |
| chr1_5748 | ugggaggcaccugagggaacg | 0.3 | 8 |
| chrZ_28399 | cuggacaagaacaucuguggguug | 0.8 | 29 |
| chr15_9127 | ccacugagcugcaaacug | 1.1 | 31 |
| chr22_15517 | cacguaucuguagggugaaaggaa | 0 | 8 |
| chr10_5928 | ccacugggaguccgacugcugag | 1.9 | 13 |
| chr1_4133 | ugugugaucugucgcugcguggaag | 2 | 17 |
| chr4_20491 | cuuuacuguagugccaccaugaa | 0.1 | 10 |
| chr3_17490 | uugauaggacagugguagcugu | 2 | 52 |
| chr21_15355 | cgcugucggccuguccuagc | 2.9 | 20 |
| chr1_3814 | gcuaaaaucaaagagcuccugc | 1.3 | 35 |
| chr2_10665 | cauaaugauggauccuggacugg | 1.2 | 16 |
| chr2_14553 | caucuuggaguauaagguaccug | 2.8 | 78 |
| chr2_12711 | auguugcuggaaggagcagagg | 0 | 7 |
| chr3_16998 | cauaagguuggugcuggaaaugg | 0 | 8 |
| chr19_10184 | agcccgugggugcugcagacgu | 9.6 | 16 |
| chr3_18922 | uugaguacacuguggaaaagc | 1.3 | 123 |
| chr8_26570 | ucagugaccacacucaggugu | 2.1 | 36 |
| chr19_10381 | uagggcugcugcugaauuccugc | 2 | 14 |
| chr3_17548 | cuuuggacuuaagggauuuu | 1 | 15 |
| chr15_8900 | uaugggaggaacugaaugacaug | 1.1 | 26 |
| chr12_7109 | caacgugggcuggacugg | 1.3 | 10 |
| chrZ_28318 | guugucagguccuguagggaugc | 1.3 | 22 |
| chr3_17529 | gguuuguaggaggcacgu | 1.2 | 22 |
| chr15_9025 | uagugaugggacuagaaacuug | 2.1 | 20 |
| chr26_16367 | ccugugcugucagaucuccuaag | 0.8 | 9 |
| chr7_25604 | uuaaucuagccugcucucuag | 1.9 | 49 |
| chr3_19170 | ucuugcagcagugagaaccaga | 2.2 | 17 |
| chr2_10827 | cuaugucagagcugguaau | 1.1 | 144 |
| chr17_9534 | cugggagucggagguguggc | 2.5 | 40 |
| chr28_16843 | aggugggcaaugcugagcuugguc | 0 | 7 |
| chr5_23219 | augcuggccagacagguaggagga | 2.4 | 16 |
| chr2_10586 | ugaugaaaucccccuauggccugu | 8.2 | 13 |
| chr2_11791 | gcucucugaucucauugucgg | 0.6 | 10 |
| chr1_2963 | cccgacuggaccgcgccgcucu | 2.3 | 8 |
| chr2_12858 | guuacugguacugccuug | 1.6 | 19 |
| chr12_7177 | gcagggucguccacuucuggcagc | 1.4 | 10 |
| chr17_9284 | cugcucugcuggucuguacugugc | 0.5 | 8 |
| chr3_18564 | ugacugggcugucaugguaac | 0.9 | 12 |
| chr1_4665 | ugagggacugcucaucugaccc | 1.1 | 14 |
| chr9_27475 | uggcugagcucagugggagcgc | 2 | 8 |
| chr11_6836 | ccugcccggcgccgucuguga | 2.9 | 12 |
| chr4_21042 | uccuugguuuugugcucgaagc | 1.1 | 10 |
| chr1_2443 | ccuggaguaguggauccaguuc | 12.7 | 22 |
| chr2_10865 | cuagugcucggccggaaccuugu | 1.8 | 11 |
| chr15_8815 | uuuauguuggcacugugcua | 0 | 7 |
| chr5_22747 | aaaaggauaugguuuugcuuc | 0.9 | 10 |
| chr10_6037 | cugcuugcgcugaaaccuccac | 0 | 8 |
| chr1_3912 | cucaaggacaugggagugg | 1.8 | 12 |
| chr11_7025 | caaauacggaccuguuugauc | 0 | 8 |
| chr17_9516 | ugccacuguagccgggccauc | 2.2 | 16 |
| chr2_13689 | guacugaggaauccccaucugg | 6.2 | 11 |
| chrUn_AADN03021983_30593 | ucucuauggggucucuguga | 2.4 | 15 |
| chrUn_JH375554_30268 | aaagcaggcugcaggaggaugc | 7.3 | 20 |
| chr13_7961 | ucuuacacugcagggcugagu | 1.7 | 25 |
| chrUn_AADN03024708_30580 | auacuggucuguacuggucugu | 1.6 | 44 |
| chr3_19174 | gggcucugagaucuucuugu | 0 | 8 |
| chr1_4913 | caagauugucagguuacugggaaug | 0.7 | 10 |
| chr2_13163 | cauuugugcucuguagugauuuugc | 0 | 8 |
| chr2_11328 | cugagccuguagcugccacugg | 1.9 | 14 |
| chrUn_AADN03012176_30432 | gaugggacccauaggucuaugg | 0.1 | 6 |
| chr1_3755 | uguugacugaccugcccuguguuc | 0.9 | 9 |
| chr2_12429 | cugcugggaauacugaguaagg | 2.5 | 18 |
| chr21_15423 | uuggguggacgguuggacuuga | 2.1 | 16 |
| chr4_21112 | aguuggagagauuuguagguuau | 0.7 | 14 |
| chr12_7574 | ugauagcugcgagaucugcgc | 0.9 | 9 |
