# Supplementary material for: Analysis of deep sequencing exosome‐microRNA expression profile derived from CP‐II reveals potential role of gga‐miRNA‐451 in inflammation
Source: J Cell Mol Med. 2020 Apr 19;24(11):6178–90. doi: 10.1111/jcmm.15244 (PMC7294135; doi:10.1111/jcmm.15244)
Supplement: Supplementary file 6 — TableS5 [file JCMM-24-6178-s006.docx]

**Supplementary Table 5: List of exosomal miRNAs presented in the exosomes (Fold Change and P-value; NC: Non-infection, MG: MG infection).**

| **miRNA_ID** |  | **NC**  **1** | **NC**  **2** | **NC3** | **MG**  **1** | **MG2** | **MG3** | **NC1,2,3** | **MG1,2,3** | **log2**  **foldchange** | **P-value** |
| --- | --- | --- | --- | --- | --- | --- | --- | --- | --- | --- | --- |
| gga-let-7d |  | 358.9 | 320 | 279 | 144.2 | 126 | 157 | 319.123 | 142.44 | -1.1638 | 1.12E-05 |
| gga-miR-193a-3p |  | 32.29 | 42.3 | 43 | 172.6 | 86.6 | 44.8 | 39.2133 | 101.3633 | 1.3701 | 1.53E-05 |
| gga-miR-153-3p |  | 11.03 | 8.7 | 9.1 | 17.8 | 15.4 | 14.8 | 9.6 | 16.0233 | 0.7391 | 2.46E-05 |
| gga-miR-451 |  | 2018 | 1984 | ## | 949.2 | 876 | 961 | 1908.72 | 928.78 | -1.0392 | 4.19E-05 |
| gga-miR-15a |  | 49.42 | 63.5 | 63 | 182.2 | 104 | 65.9 | 58.7467 | 117.3433 | 0.9982 | 4.69E-05 |
| gga-miR-133c-3p |  | 23.82 | 28.3 | 25 | 9.29 | 9.37 | 13.9 | 25.7933 | 10.8567 | -1.2484 | 5.16E-05 |
| gga-miR-138-5p |  | 37.02 | 46.9 | 52 | 92.14 | 75.5 | 52.6 | 45.2533 | 73.3933 | 0.6976 | 5.23E-05 |
| gga-miR-33-5p |  | 5.51 | 9.63 | 12 | 29.97 | 18.7 | 11.8 | 9.13 | 20.15 | 1.1421 | 6.86E-05 |
| gga-miR-223 |  | 102 | 61.5 | 47 | 25.39 | 26.3 | 27.8 | 70.0267 | 26.5067 | -1.4015 | 9.18E-05 |
| gga-miR-146a-3p |  | 4.53 | 6.68 | 4.8 | 7.46 | 10.3 | 11 | 5.3267 | 9.5867 | 0.8478 | 0.000107 |
| gga-miR-21-3p |  | 242.4 | 420 | 476 | 931.5 | 668 | 443 | 379.5 | 680.63 | 0.8428 | 0.000108 |
| gga-miR-460b-5p |  | 1.18 | 1.71 | 1.9 | 4.45 | 3.17 | 3.4 | 1.6 | 3.6733 | 1.199 | 0.000119 |
| gga-miR-144-5p |  | 87.42 | 66.8 | 73 | 39.92 | 38.3 | 32.3 | 75.5767 | 36.8367 | -1.0368 | 0.000217 |
| gga-miR-148a-5p |  | 17.72 | 18.6 | 19 | 45.15 | 31.7 | 18.1 | 18.4833 | 31.6367 | 0.7754 | 0.000446 |
| gga-miR-22-5p |  | 258.3 | 345 | 325 | 449.1 | 385 | 375 | 309.66 | 403.1933 | 0.3808 | 0.000643 |
| gga-miR-1574-3p |  | 4.73 | 3.11 | 2.1 | 1.05 | 0.96 | 0.62 | 3.3033 | 0.8767 | -1.9138 | 0.000682 |
| gga-miR-29a-3p |  | 227.2 | 268 | 253 | 480.3 | 341 | 256 | 249.28 | 359.1 | 0.5266 | 0.00069 |
| gga-miR-29c-3p |  | 227.2 | 268 | 253 | 480.3 | 341 | 256 | 249.28 | 359.1 | 0.5266 | 0.000694 |
| gga-miR-146a-5p |  | 1818 | 1888 | ## | 2207 | 2339 | 2409 | 1861.84 | 2318.27 | 0.3163 | 0.00072 |
| gga-miR-21-5p |  | #### | #### | ## | #### | ### | ### | 159766 | 200892.3 | 0.3305 | 0.000917 |
| gga-miR-130a-3p |  | 56.9 | 67.9 | 79 | 114.3 | 97.8 | 69.9 | 68.0433 | 93.97 | 0.4657 | 0.001056 |
| gga-miR-365-3p |  | 244.9 | 159 | 142 | 51.3 | 83.2 | 100 | 181.84 | 78.3133 | -1.2153 | 0.001204 |
| gga-miR-22-3p |  | 6121 | 8048 | ## | 10121 | ### | 7973 | 7446.54 | 9418.33 | 0.3389 | 0.001444 |
| gga-miR-15c-5p |  | 56.51 | 82.6 | 80 | 155.4 | 98.9 | 71.6 | 72.8867 | 108.6033 | 0.5753 | 0.002104 |
| gga-miR-193b-3p |  | 142.2 | 85.9 | 93 | 53.79 | 56.7 | 50.1 | 107.027 | 53.5367 | -0.9994 | 0.002109 |
| gga-miR-449c-5p |  | 4.53 | 3.11 | 2.5 | 0.79 | 1.24 | 1.39 | 3.3933 | 1.14 | -1.5737 | 0.002289 |
| gga-miR-138-1-3p |  | 24.41 | 23.5 | 17 | 11.52 | 11.3 | 9.27 | 21.5733 | 10.6933 | -1.0125 | 0.002451 |
| gga-miR-206 |  | 22.44 | 19.6 | 17 | 8.9 | 9.64 | 10.8 | 19.57 | 9.7867 | -0.9997 | 0.002655 |
| gga-miR-3535 |  | 5.32 | 3.42 | 4 | 1.57 | 1.52 | 1.7 | 4.24 | 1.5967 | -1.409 | 0.003015 |
| gga-miR-148a-3p |  | #### | #### | ## | #### | ### | ### | 302733 | 356582.8 | 0.2362 | 0.003322 |
| gga-miR-133b |  | 7.28 | 6.21 | 7.5 | 3.01 | 3.31 | 3.09 | 6.99 | 3.1367 | -1.156 | 0.003705 |
| gga-miR-29b-3p |  | 128.8 | 136 | 150 | 244.2 | 180 | 127 | 138.207 | 183.6267 | 0.4099 | 0.004178 |
| gga-miR-32-5p |  | 98.83 | 99.9 | 97 | 161.3 | 126 | 93.8 | 98.73 | 126.9867 | 0.3631 | 0.004205 |
| gga-miR-218-5p |  | 1016 | 1215 | ## | 1775 | 1498 | 1181 | 1194.4 | 1484.623 | 0.3138 | 0.004463 |
| gga-miR-27b-3p |  | 4663 | 5658 | ## | 8484 | 6802 | 4987 | 5358.53 | 6757.54 | 0.3347 | 0.004597 |
| gga-miR-449c-3p |  | 2.36 | 1.71 | 1.6 | 0.65 | 0.55 | 0.31 | 1.8867 | 0.5033 | -1.9064 | 0.004774 |
| gga-miR-200a-3p |  | 3691 | 4229 | ## | 4967 | 4891 | 4209 | 4018.05 | 4688.95 | 0.2228 | 0.005211 |
| gga-miR-122-5p |  | 64773 | #### | ## | 25453 | ### | ### | 51086.8 | 27944 | -0.8704 | 0.006302 |
| gga-miR-211 |  | 82.7 | 97.7 | 77 | 35.6 | 53.4 | 51.3 | 85.8967 | 46.7867 | -0.8765 | 0.006585 |
| gga-miR-204 |  | 82.7 | 97.7 | 77 | 35.6 | 53.4 | 51.3 | 85.8967 | 46.7867 | -0.8765 | 0.006587 |
| gga-miR-460a-5p |  | 8.66 | 12.6 | 16 | 24.87 | 14.9 | 13.8 | 12.4867 | 17.8333 | 0.5142 | 0.007638 |
| gga-miR-1559-3p |  | 7.28 | 7.92 | 8 | 11.65 | 9.5 | 9.27 | 7.7167 | 10.14 | 0.394 | 0.007861 |
| gga-miR-128-1-5p |  | 6.89 | 8.54 | 7.8 | 2.49 | 4.82 | 3.71 | 7.74 | 3.6733 | -1.0753 | 0.008342 |
| gga-miR-16-1-3p |  | 2.95 | 2.33 | 1.4 | 4.71 | 4.27 | 2.47 | 2.2367 | 3.8167 | 0.771 | 0.008348 |
| gga-miR-32-3p |  | 3.94 | 4.66 | 2.7 | 4.71 | 6.2 | 6.03 | 3.7667 | 5.6467 | 0.5841 | 0.008357 |
| gga-miR-1573 |  | 0.59 | 0.93 | 0.8 | 0 | 0 | 0.31 | 0.7733 | 0.1033 | -2.9042 | 0.008445 |
| gga-miR-133a-3p |  | 100.4 | 83.4 | 75 | 35.73 | 34.7 | 61.7 | 86.4 | 44.0367 | -0.9723 | 0.008678 |
| gga-miR-9-3p |  | 0.79 | 1.24 | 2.2 | 2.23 | 3.17 | 2.16 | 1.42 | 2.52 | 0.8275 | 0.010794 |
| gga-miR-15b-5p |  | 68.71 | 93.7 | 91 | 159.8 | 101 | 75 | 84.4433 | 111.82 | 0.4051 | 0.011331 |
| gga-miR-202-5p |  | 0.2 | 0 | 0 | 0.92 | 0.55 | 0.15 | 0.0667 | 0.54 | 3.0172 | 0.011886 |
| gga-miR-1727 |  | 4.53 | 5.28 | 5.1 | 7.33 | 6.75 | 6.18 | 4.9667 | 6.7533 | 0.4433 | 0.012006 |
| gga-miR-1553-5p |  | 1.18 | 1.55 | 0.8 | 0.26 | 0.28 | 0.31 | 1.1767 | 0.2833 | -2.0543 | 0.012467 |
| gga-miR-107-3p |  | 395.9 | 530 | 566 | 806.5 | 592 | 449 | 497.093 | 615.89 | 0.3092 | 0.013015 |
| gga-miR-1416-3p |  | 0.39 | 0.78 | 2.1 | 2.62 | 1.65 | 2.01 | 1.08 | 2.0933 | 0.9547 | 0.013786 |
| gga-miR-30c-1-3p |  | 163.2 | 140 | 149 | 78.92 | 93.9 | 96.8 | 150.727 | 89.87 | -0.746 | 0.014203 |
| gga-miR-1798-5p |  | 0.79 | 1.24 | 2.4 | 2.62 | 3.58 | 1.7 | 1.4733 | 2.6333 | 0.8378 | 0.015219 |
| gga-miR-146c-3p |  | 292.8 | 358 | 401 | 396.8 | 441 | 359 | 350.62 | 398.8333 | 0.1859 | 0.015593 |
| gga-miR-1662 |  | 7.48 | 11 | 9.4 | 12.56 | 10.7 | 11.6 | 9.2967 | 11.63 | 0.3231 | 0.015833 |
| gga-miR-449b-5p |  | 5.32 | 5.44 | 8.6 | 3.93 | 2.2 | 2.94 | 6.45 | 3.0233 | -1.0932 | 0.0165 |
| gga-miR-1769-3p |  | 31.7 | 23.5 | 25 | 26.83 | 32 | 39.9 | 26.8133 | 32.8867 | 0.2946 | 0.017275 |
| gga-miR-455-3p |  | 173.7 | 103 | 143 | 63.09 | 74.5 | 93.7 | 139.817 | 77.09 | -0.8589 | 0.018468 |
| gga-miR-3523 |  | 75.41 | 131 | 117 | 58.24 | 52.8 | 70.2 | 107.64 | 60.3867 | -0.8339 | 0.019354 |
| gga-miR-1720-5p |  | 6.69 | 5.9 | 6.4 | 3.53 | 2.75 | 3.25 | 6.3167 | 3.1767 | -0.9916 | 0.019358 |
| gga-miR-24-5p |  | 7.28 | 7.61 | 8 | 15.44 | 9.78 | 6.34 | 7.6133 | 10.52 | 0.4665 | 0.021273 |
| gga-miR-1784-5p |  | 0.2 | 0.16 | 0 | 1.31 | 0.41 | 0.15 | 0.12 | 0.6233 | 2.3769 | 0.023536 |
| gga-miR-6550-3p |  | 0.79 | 0.93 | 1.6 | 0.52 | 0.28 | 0 | 1.1033 | 0.2667 | -2.0485 | 0.023785 |
| gga-miR-20b-3p |  | 9.84 | 13.7 | 12 | 18.85 | 13.5 | 11.8 | 11.8667 | 14.7 | 0.3089 | 0.024599 |
| gga-miR-6555-3p |  | 0.59 | 0.47 | 0.5 | 0.79 | 1.79 | 0.93 | 0.5133 | 1.17 | 1.1886 | 0.024664 |
| gga-miR-1648-5p |  | 0.2 | 0.93 | 0.2 | 0 | 0 | 0 | 0.43 | 0 | -5.4263 | 0.025422 |
| gga-let-7a-2-3p |  | 10.43 | 6.37 | 8.9 | 3.4 | 4.68 | 5.1 | 8.57 | 4.3933 | -0.964 | 0.02626 |
| gga-miR-1783 |  | 0 | 0 | 0 | 0.52 | 0.28 | 0.15 | 0 | 0.3167 | 4.985 | 0.026481 |
| gga-miR-219b |  | 43.51 | 40.1 | 39 | 59.81 | 44.4 | 38.2 | 40.7433 | 47.4467 | 0.2197 | 0.027355 |
| gga-miR-199b |  | 1.38 | 17.9 | 24 | 6.94 | 3.58 | 2.63 | 14.3667 | 4.3833 | -1.7126 | 0.028687 |
| gga-miR-1434 |  | 60.84 | 103 | 97 | 93.71 | 106 | 100 | 86.8367 | 100.0167 | 0.2039 | 0.029125 |
| gga-miR-34b-5p |  | 17.92 | 19.3 | 20 | 35.08 | 22.3 | 15.6 | 19.1767 | 24.3333 | 0.3436 | 0.030022 |
| gga-miR-6696-3p |  | 0.39 | 0.47 | 0.2 | 1.31 | 0.69 | 0.62 | 0.34 | 0.8733 | 1.3609 | 0.03085 |
| gga-miR-6565-3p |  | 1.18 | 1.86 | 1 | 2.75 | 2.75 | 1.24 | 1.33 | 2.2467 | 0.7564 | 0.031179 |
| gga-miR-101-3p |  | 2045 | 2327 | ## | 3478 | 2558 | 1763 | 2222.48 | 2599.49 | 0.2261 | 0.032351 |
| gga-miR-1731-5p |  | 3.94 | 3.42 | 2.4 | 6.94 | 4.68 | 2.63 | 3.25 | 4.75 | 0.5475 | 0.033046 |
| gga-miR-130c-3p |  | 50.4 | 64.6 | 69 | 75.52 | 71.1 | 58.3 | 61.2933 | 68.2867 | 0.1559 | 0.03305 |
| gga-miR-218-3p |  | 0.39 | 0.62 | 0.5 | 1.96 | 1.1 | 0.46 | 0.4967 | 1.1733 | 1.2401 | 0.03384 |
| gga-miR-196-5p |  | 14.96 | 10.4 | 23 | 7.47 | 11.6 | 5.1 | 15.9867 | 8.05 | -0.9898 | 0.036893 |
| gga-miR-34c-5p |  | 9.06 | 9.47 | 10 | 17.15 | 10.9 | 8.19 | 9.57 | 12.0733 | 0.3352 | 0.036924 |
| gga-miR-215-5p |  | 298.9 | 304 | 298 | 400.1 | 333 | 265 | 300.08 | 332.5467 | 0.1482 | 0.037105 |
| gga-let-7c-5p |  | 5766 | 5387 | ## | 3005 | 3611 | 3975 | 5591.25 | 3530.463 | -0.6633 | 0.038567 |
| gga-miR-147 |  | 48.63 | 83.1 | 70 | 107.2 | 86.8 | 52.7 | 67.0733 | 82.2233 | 0.2938 | 0.040179 |
| gga-miR-1788-5p |  | 5.12 | 4.04 | 3.5 | 0.79 | 2.75 | 2.16 | 4.22 | 1.9 | -1.1512 | 0.041102 |
| gga-miR-181b-2-3p |  | 1.77 | 3.73 | 4.5 | 4.97 | 5.65 | 3.25 | 3.3167 | 4.6233 | 0.4792 | 0.042135 |
| gga-miR-181b-1-3p |  | 2.56 | 3.73 | 4.9 | 7.33 | 5.37 | 2.94 | 3.74 | 5.2133 | 0.4792 | 0.042649 |
| gga-miR-1a-3p |  | 766.3 | 783 | 888 | 910.4 | 892 | 792 | 812.437 | 864.6933 | 0.0899 | 0.043129 |
| gga-miR-1771 |  | 0.2 | 1.86 | 0.8 | 0.39 | 0.14 | 0 | 0.9533 | 0.1767 | -2.4316 | 0.043552 |
| gga-miR-1805-3p |  | 6.1 | 6.06 | 7 | 9.55 | 8.81 | 5.41 | 6.3867 | 7.9233 | 0.311 | 0.045531 |
| gga-miR-205b |  | 23.04 | 19.3 | 22 | 9.95 | 10.1 | 16.9 | 21.4167 | 12.2833 | -0.802 | 0.047107 |
| gga-miR-1779 |  | 5.32 | 4.81 | 2.2 | 7.2 | 5.92 | 3.71 | 4.12 | 5.61 | 0.4454 | 0.051668 |
| gga-miR-6586-5p |  | 0.79 | 1.55 | 1 | 1.31 | 1.38 | 2.63 | 1.0967 | 1.7733 | 0.6933 | 0.052244 |
| gga-miR-128-3p |  | 20539 | #### | ## | 24960 | ### | ### | 26551 | 28649.54 | 0.1097 | 0.053197 |
| gga-miR-15b-3p |  | 10.63 | 9.63 | 7.5 | 4.84 | 4.96 | 5.87 | 9.2467 | 5.2233 | -0.824 | 0.055907 |
| gga-miR-10b-5p |  | 1338 | 823 | 805 | 487 | 568 | 714 | 988.523 | 589.5667 | -0.7456 | 0.059689 |
| gga-miR-3538 |  | 73.44 | 47.4 | 59 | 46.07 | 35.4 | 22 | 59.8333 | 34.4733 | -0.7955 | 0.061095 |
| gga-miR-1590 |  | 0 | 0.47 | 0.2 | 1.05 | 0.41 | 0.46 | 0.21 | 0.64 | 1.6077 | 0.062217 |
| gga-miR-6652-5p |  | 1.38 | 0.93 | 1.8 | 0.13 | 0.96 | 0.31 | 1.3533 | 0.4667 | -1.5359 | 0.062453 |
| gga-let-7a-5p |  | 48719 | #### | ## | 26398 | ### | ### | 46188.3 | 30235.77 | -0.6113 | 0.06616 |
| gga-miR-1665 |  | 0 | 0.47 | 0.5 | 0 | 0 | 0 | 0.3167 | 0 | -4.985 | 0.066417 |
| gga-let-7j-5p |  | 48719 | #### | ## | 26398 | ### | ### | 46188.3 | 30235.77 | -0.6113 | 0.066617 |
| gga-miR-10a-3p |  | 69.7 | 65.2 | 67 | 78.53 | 66.5 | 67.6 | 67.4033 | 70.8667 | 0.0723 | 0.06692 |
| gga-miR-181a-5p |  | 10330 | #### | ## | 13884 | ### | ### | 11852.6 | 12366.24 | 0.0612 | 0.067636 |
| gga-miR-6641-3p |  | 0 | 0 | 0 | 0.39 | 0 | 0.46 | 0 | 0.2833 | 4.8243 | 0.0679 |
| gga-miR-24-3p |  | 6759 | 7335 | ## | 7956 | 7507 | 7405 | 7395.94 | 7622.863 | 0.0436 | 0.0694 |
| gga-miR-1717 |  | 0.59 | 0.62 | 1 | 0 | 0.41 | 0.15 | 0.72 | 0.1867 | -1.9473 | 0.073977 |
| gga-miR-383-3p |  | 1.18 | 0.31 | 0.6 | 1.83 | 0.83 | 0.93 | 0.71 | 1.1967 | 0.7532 | 0.081557 |
| gga-miR-1563 |  | 1.18 | 2.02 | 1.6 | 0.52 | 0.41 | 1.08 | 1.5967 | 0.67 | -1.2529 | 0.082309 |
| gga-miR-33-3p |  | 0.2 | 0.47 | 0.8 | 0.92 | 0.55 | 1.55 | 0.49 | 1.0067 | 1.0388 | 0.083888 |
| gga-let-7b |  | 2794 | 2550 | ## | 1463 | 1654 | 1984 | 2590.63 | 1700.113 | -0.6077 | 0.087421 |
| gga-miR-6564-3p |  | 0.2 | 0.16 | 0.5 | 0 | 0 | 0 | 0.28 | 0 | -4.8074 | 0.087525 |
| gga-miR-140-5p |  | 499.7 | 438 | 457 | 456.3 | 469 | 508 | 464.873 | 477.82 | 0.0396 | 0.088905 |
| gga-miR-1723 |  | 0.39 | 0.62 | 0.5 | 0.26 | 0 | 0 | 0.4967 | 0.0867 | -2.5183 | 0.088939 |
| gga-let-7a-3p |  | 446.3 | 380 | 393 | 411.1 | 413 | 431 | 406.407 | 418.2167 | 0.0413 | 0.089031 |
| gga-let-7k-3p |  | 446.3 | 380 | 393 | 411.1 | 413 | 431 | 406.407 | 418.2167 | 0.0413 | 0.089222 |
| gga-miR-6544-5p |  | 0.98 | 1.24 | 2.2 | 1.7 | 2.07 | 2.47 | 1.4833 | 2.08 | 0.4878 | 0.090896 |
| gga-miR-6705-5p |  | 1.58 | 1.4 | 1.9 | 1.18 | 0.28 | 0.62 | 1.63 | 0.6933 | -1.2333 | 0.094196 |
| gga-miR-142-5p |  | 540.8 | 583 | 523 | 428 | 355 | 307 | 548.997 | 363.28 | -0.5957 | 0.094254 |
| gga-miR-6570-3p |  | 0.39 | 0 | 0 | 0.52 | 0.28 | 0.46 | 0.13 | 0.42 | 1.6919 | 0.094364 |
| gga-miR-99a-3p |  | 5.51 | 4.66 | 3.3 | 2.75 | 2.07 | 2.47 | 4.5033 | 2.43 | -0.89 | 0.095809 |
| gga-miR-146c-5p |  | 70665 | #### | ## | 62030 | ### | ### | 68422.2 | 69553.24 | 0.0237 | 0.104283 |
| gga-miR-18a-3p |  | 50.99 | 48.6 | 54 | 30.1 | 34.2 | 37.4 | 51.0667 | 33.89 | -0.5915 | 0.106934 |
| gga-miR-456-3p |  | 899 | 1580 | ## | 972.6 | 1257 | 1942 | 1245.04 | 1390.843 | 0.1598 | 0.109954 |
| gga-miR-2128 |  | 0.2 | 1.71 | 1.8 | 2.09 | 1.38 | 2.01 | 1.22 | 1.8267 | 0.5824 | 0.110384 |
| gga-miR-1552-3p |  | 144.9 | 222 | 208 | 201.8 | 189 | 203 | 191.63 | 197.8133 | 0.0458 | 0.112249 |
| gga-let-7g-5p |  | 6475 | 5559 | ## | 4094 | 4548 | 3641 | 6039.78 | 4094.55 | -0.5608 | 0.114814 |
| gga-miR-499-5p |  | 194.1 | 225 | 194 | 293.8 | 223 | 147 | 204.093 | 221.1167 | 0.1156 | 0.116441 |
| gga-miR-181b-5p |  | 6224 | 6829 | ## | 7591 | 6690 | 5996 | 6709.45 | 6759.13 | 0.0106 | 0.117466 |
| gga-miR-1798-3p |  | 3.15 | 1.09 | 1.3 | 3.14 | 3.31 | 1.24 | 1.8367 | 2.5633 | 0.4809 | 0.12011 |
| gga-miR-2188-5p |  | 1.18 | 0 | 0 | 0 | 0 | 0 | 0.3933 | 0 | -5.2976 | 0.122498 |
| gga-miR-194 |  | 169.7 | 183 | 175 | 133.6 | 106 | 118 | 176.087 | 119.2933 | -0.5618 | 0.123242 |
| gga-miR-16c-5p |  | 949.2 | 987 | ## | 1103 | 1062 | 910 | 1020.8 | 1024.967 | 0.0059 | 0.126635 |
| gga-miR-302b-5p |  | 0.79 | 2.17 | 1.1 | 2.23 | 1.38 | 2.01 | 1.3567 | 1.8733 | 0.4655 | 0.126943 |
| gga-miR-34a-3p |  | 0.59 | 1.09 | 1 | 0.92 | 1.24 | 2.01 | 0.8767 | 1.39 | 0.6649 | 0.127145 |
| gga-miR-1682 |  | 5.91 | 6.52 | 5.6 | 2.62 | 2.34 | 5.26 | 6 | 3.4067 | -0.8166 | 0.128677 |
| gga-miR-3529 |  | 34.26 | 43.2 | 45 | 46.73 | 46.7 | 33.4 | 40.8167 | 42.27 | 0.0505 | 0.129264 |
| gga-miR-34c-3p |  | 0.39 | 1.4 | 0.6 | 0 | 0.55 | 0.15 | 0.81 | 0.2333 | -1.7957 | 0.129383 |
| gga-miR-29b-2-5p |  | 0 | 0.47 | 0.6 | 0 | 0 | 0.15 | 0.37 | 0.05 | -2.8875 | 0.130254 |
| gga-miR-187-3p |  | 372.1 | 704 | 644 | 523.3 | 593 | 707 | 573.117 | 607.7633 | 0.0847 | 0.131506 |
| gga-miR-458a-3p |  | 278.2 | 291 | 289 | 336.9 | 275 | 253 | 286.08 | 288.4067 | 0.0117 | 0.132057 |
| gga-miR-1747-5p |  | 10.83 | 11.5 | 10 | 11.26 | 11.3 | 12.1 | 10.8333 | 11.5367 | 0.0908 | 0.132608 |
| gga-miR-1457 |  | 0.2 | 0.62 | 0 | 0 | 0 | 0 | 0.2733 | 0 | -4.7724 | 0.132739 |
| gga-miR-6675-3p |  | 1.58 | 1.4 | 2.7 | 2.23 | 2.07 | 2.94 | 1.8933 | 2.4133 | 0.3501 | 0.136061 |
| gga-miR-1781-5p |  | 0 | 0 | 0 | 0.52 | 0.14 | 0 | 0 | 0.22 | 4.4594 | 0.137752 |
| gga-miR-1559-5p |  | 1272 | 1103 | ## | 1242 | 1270 | 1059 | 1186.8 | 1190.68 | 0.0047 | 0.138895 |
| gga-miR-6582-3p |  | 0.59 | 3.88 | 3 | 4.45 | 2.34 | 3.25 | 2.4967 | 3.3467 | 0.4227 | 0.139251 |
| gga-miR-193b-5p |  | 5.91 | 2.95 | 3.3 | 1.96 | 1.52 | 3.09 | 4.0667 | 2.19 | -0.8929 | 0.141935 |
| gga-miR-6557-5p |  | 0.2 | 0.31 | 0.2 | 0 | 0 | 0 | 0.2233 | 0 | -4.4809 | 0.145101 |
| gga-miR-1555-5p |  | 0 | 0.78 | 0.3 | 0 | 0.14 | 0 | 0.3667 | 0.0467 | -2.9731 | 0.145873 |
| gga-miR-1397-5p |  | 0.79 | 0 | 0.2 | 0.65 | 0.41 | 1.08 | 0.3167 | 0.7133 | 1.1714 | 0.149768 |
| gga-miR-18b-5p |  | 1.97 | 5.13 | 5.3 | 6.41 | 5.1 | 3.4 | 4.1167 | 4.97 | 0.2718 | 0.150707 |
| gga-miR-30b-3p |  | 5.51 | 3.26 | 3 | 1.96 | 2.34 | 2.32 | 3.93 | 2.2067 | -0.8326 | 0.155332 |
| gga-miR-1722-5p |  | 0 | 0.16 | 0 | 0.39 | 0.28 | 0.15 | 0.0533 | 0.2733 | 2.3583 | 0.155651 |
| gga-let-7f-5p |  | 14147 | #### | ## | 9230 | ### | 8980 | 14037.7 | 9739.83 | -0.5273 | 0.158184 |
| gga-miR-1786 |  | 0.98 | 2.17 | 1.8 | 0.13 | 0.41 | 1.55 | 1.6333 | 0.6967 | -1.2292 | 0.158583 |
| gga-miR-3532-5p |  | 0.79 | 1.24 | 1.6 | 0.26 | 0.55 | 0.77 | 1.2067 | 0.5267 | -1.196 | 0.161917 |
| gga-miR-31-3p |  | 0.79 | 1.55 | 2.1 | 1.31 | 2.2 | 2.32 | 1.47 | 1.9433 | 0.4027 | 0.163795 |
| gga-miR-202-3p |  | 0.59 | 0.16 | 0.3 | 0.13 | 0 | 0 | 0.3567 | 0.0433 | -3.0423 | 0.165594 |
| gga-miR-1555-3p |  | 0.59 | 0.16 | 0.3 | 0 | 0.14 | 0 | 0.3567 | 0.0467 | -2.9332 | 0.166803 |
| gga-miR-219a |  | 0.59 | 0.31 | 0.2 | 0.13 | 0 | 0 | 0.3533 | 0.0433 | -3.0285 | 0.167776 |
| gga-miR-1754-5p |  | 0.59 | 1.24 | 1.8 | 0.52 | 0.69 | 0.31 | 1.1933 | 0.5067 | -1.2358 | 0.169798 |
| gga-miR-217-5p |  | 3.35 | 4.5 | 3.3 | 3.8 | 5.92 | 3.25 | 3.73 | 4.3233 | 0.213 | 0.172668 |
| gga-miR-17-3p |  | 6.3 | 7.3 | 7.3 | 10.34 | 6.89 | 5.56 | 6.9733 | 7.5967 | 0.1235 | 0.172766 |
| gga-miR-1635 |  | 10.43 | 13.2 | 7.8 | 6.41 | 7.02 | 6.65 | 10.4733 | 6.6933 | -0.6459 | 0.178346 |
| gga-miR-199-3p |  | 3287 | 3659 | ## | 4966 | 3683 | 2756 | 3734.67 | 3801.953 | 0.0258 | 0.179228 |
| gga-miR-1690-5p |  | 0.39 | 0 | 0.3 | 0 | 0 | 0 | 0.2367 | 0 | -4.565 | 0.179334 |
| gga-miR-2131-3p |  | 147.9 | 193 | 207 | 191.2 | 205 | 150 | 182.507 | 182.14 | -0.0029 | 0.180928 |
| gga-miR-203a |  | 513.9 | 611 | 645 | 694.1 | 617 | 453 | 589.88 | 587.94 | -0.0048 | 0.191798 |
| gga-miR-454-3p |  | 117.9 | 68.7 | 73 | 42.8 | 59.6 | 66.2 | 86.42 | 56.2 | -0.6208 | 0.19182 |
| gga-miR-2954 |  | 802.3 | 953 | ## | 529.3 | 609 | 767 | 927.893 | 635.2467 | -0.5466 | 0.193216 |
| gga-miR-1807 |  | 0.2 | 0 | 0.5 | 0 | 0 | 0 | 0.2267 | 0 | -4.5027 | 0.194164 |
| gga-miR-6562-3p |  | 0 | 0 | 0 | 0.39 | 0 | 0.15 | 0 | 0.18 | 4.1699 | 0.196433 |
| gga-miR-1628 |  | 0 | 0 | 0 | 0 | 0.41 | 0.15 | 0 | 0.1867 | 4.2227 | 0.197753 |
| gga-miR-6559-5p |  | 0 | 0 | 0 | 0.39 | 0.14 | 0 | 0 | 0.1767 | 4.1432 | 0.198639 |
| gga-miR-30e-3p |  | 729.1 | 592 | 567 | 361 | 462 | 480 | 629.317 | 434.3333 | -0.535 | 0.200595 |
| gga-miR-1677-5p |  | 0.59 | 2.17 | 1.6 | 0.65 | 0.96 | 0.46 | 1.45 | 0.69 | -1.0714 | 0.200693 |
| gga-miR-6615-5p |  | 2.56 | 0.78 | 2.9 | 2.62 | 1.93 | 3.09 | 2.0667 | 2.5467 | 0.3013 | 0.201243 |
| gga-miR-6631-5p |  | 2.76 | 2.17 | 3.5 | 3.27 | 2.89 | 3.56 | 2.81 | 3.24 | 0.2054 | 0.206454 |
| gga-miR-1b-3p |  | 15.95 | 15.1 | 19 | 20.16 | 15.8 | 14.5 | 16.65 | 16.8433 | 0.0167 | 0.206942 |
| gga-miR-205a |  | 77.57 | 75.8 | 86 | 55.49 | 54.8 | 58.1 | 79.7467 | 56.1433 | -0.5063 | 0.207259 |
| gga-miR-222b-5p |  | 9.84 | 9.47 | 11 | 7.33 | 5.1 | 7.11 | 9.99 | 6.5133 | -0.6171 | 0.207304 |
| gga-miR-1551-5p |  | 11.62 | 14.8 | 11 | 16.1 | 10.3 | 12.5 | 12.5533 | 12.9833 | 0.0486 | 0.20937 |
| gga-miR-301a-3p |  | 5.51 | 4.35 | 4 | 4.58 | 4.27 | 6.18 | 4.6133 | 5.01 | 0.119 | 0.210743 |
| gga-miR-6553-3p |  | 0.59 | 0.78 | 0.2 | 0.26 | 1.24 | 1.08 | 0.51 | 0.86 | 0.7538 | 0.210874 |
| gga-let-7k-5p |  | 2104 | 1975 | ## | 2084 | 2167 | 1859 | 2110.86 | 2036.64 | -0.0516 | 0.211539 |
| gga-miR-1649-5p |  | 4.73 | 4.04 | 2.4 | 4.45 | 3.99 | 4.02 | 3.72 | 4.1533 | 0.159 | 0.216602 |
| gga-miR-1724 |  | 0.59 | 0.62 | 0.5 | 0.79 | 0.83 | 0.93 | 0.5633 | 0.85 | 0.5936 | 0.218293 |
| gga-miR-1306-3p |  | 105.7 | 125 | 129 | 110.3 | 108 | 132 | 120.073 | 116.77 | -0.0402 | 0.218708 |
| gga-miR-6710-3p |  | 1.77 | 0.78 | 1.1 | 1.31 | 0.83 | 2.63 | 1.22 | 1.59 | 0.3821 | 0.221979 |
| gga-miR-6548-5p |  | 1.38 | 0.62 | 1.1 | 0.92 | 0.28 | 0 | 1.0367 | 0.4 | -1.3739 | 0.222066 |
| gga-miR-3540 |  | 1.97 | 2.8 | 2.7 | 3.53 | 2.75 | 2.32 | 2.49 | 2.8667 | 0.2032 | 0.222231 |
| gga-miR-7472-5p |  | 2.95 | 2.64 | 2.1 | 3.01 | 3.03 | 2.63 | 2.5533 | 2.89 | 0.1787 | 0.223973 |
| gga-miR-6543-5p |  | 1.58 | 1.24 | 0.6 | 1.7 | 1.24 | 1.39 | 1.1533 | 1.4433 | 0.3236 | 0.226974 |
| gga-miR-3532-3p |  | 5.91 | 4.66 | 5.4 | 3.27 | 3.31 | 3.56 | 5.3267 | 3.38 | -0.6562 | 0.230853 |
| gga-miR-1654 |  | 0.59 | 0 | 0.2 | 1.05 | 0.28 | 0.15 | 0.25 | 0.4933 | 0.9805 | 0.232498 |
| gga-miR-1759-5p |  | 0.2 | 0.31 | 0.2 | 1.18 | 0.14 | 0.15 | 0.2233 | 0.49 | 1.1338 | 0.233781 |
| gga-miR-30c-2-3p |  | 91.94 | 84.2 | 85 | 62.3 | 63.2 | 60.3 | 87.0167 | 61.9333 | -0.4906 | 0.234717 |
| gga-miR-1456-5p |  | 166.4 | 161 | 200 | 136.5 | 127 | 110 | 175.63 | 124.3167 | -0.4985 | 0.238147 |
| gga-miR-6604-5p |  | 5.32 | 4.19 | 4.1 | 3.8 | 3.99 | 7.11 | 4.55 | 4.9667 | 0.1264 | 0.241823 |
| gga-miR-1796 |  | 0 | 0.16 | 0.3 | 0.52 | 0 | 0.77 | 0.16 | 0.43 | 1.4263 | 0.245947 |
| gga-miR-301b-3p |  | 6.69 | 3.42 | 3.5 | 6.54 | 3.86 | 4.64 | 4.5367 | 5.0133 | 0.1441 | 0.248562 |
| gga-miR-20a-3p |  | 0.39 | 0.16 | 0.3 | 0 | 0.14 | 0 | 0.29 | 0.0467 | -2.6346 | 0.255858 |
| gga-miR-216a |  | 0.2 | 0.31 | 1.1 | 0.13 | 0.28 | 0.15 | 0.54 | 0.1867 | -1.5322 | 0.256621 |
| gga-miR-6599-3p |  | 1.58 | 5.44 | 4.8 | 4.19 | 4.27 | 4.64 | 3.93 | 4.3667 | 0.152 | 0.260367 |
| gga-miR-6556-5p |  | 0.2 | 0.16 | 0.2 | 0 | 0 | 0 | 0.1733 | 0 | -4.1152 | 0.262605 |
| gga-miR-1595-3p |  | 0.2 | 0.16 | 0.2 | 0 | 0 | 0 | 0.1733 | 0 | -4.1152 | 0.263161 |
| gga-miR-1738 |  | 0.2 | 0.16 | 0.2 | 0 | 0 | 0 | 0.1733 | 0 | -4.1152 | 0.264249 |
| gga-miR-6548-3p |  | 4.73 | 6.37 | 5.4 | 2.09 | 2.89 | 5.26 | 5.5033 | 3.4133 | -0.6891 | 0.267148 |
| gga-miR-1460 |  | 0 | 0 | 0.2 | 0.13 | 0.14 | 0.46 | 0.0533 | 0.2433 | 2.1905 | 0.273953 |
| gga-miR-6662-3p |  | 0.59 | 0.78 | 0.6 | 0.26 | 0.14 | 0.46 | 0.67 | 0.2867 | -1.2246 | 0.275037 |
| gga-miR-6559-3p |  | 0.2 | 0 | 0 | 0.13 | 0.14 | 0.46 | 0.0667 | 0.2433 | 1.867 | 0.275265 |
| gga-miR-6557-3p |  | 0.2 | 0 | 0.8 | 0.13 | 0.28 | 1.7 | 0.3333 | 0.7033 | 1.0773 | 0.281937 |
| gga-miR-200b-3p |  | 9578 | 7786 | ## | 5780 | 6623 | 6437 | 8693.56 | 6279.8 | -0.4692 | 0.285416 |
| gga-miR-1792 |  | 0.39 | 0 | 1 | 0.79 | 0.55 | 0.77 | 0.4467 | 0.7033 | 0.6548 | 0.287036 |
| gga-miR-19a-3p |  | 131.5 | 69.4 | 75 | 57.33 | 97.4 | 139 | 92.06 | 97.8367 | 0.0878 | 0.287566 |
| gga-miR-383-5p |  | 401.4 | 400 | 416 | 365.2 | 374 | 409 | 406.02 | 382.6933 | -0.0854 | 0.288103 |
| gga-miR-1698 |  | 1.77 | 1.55 | 1.6 | 1.05 | 2.07 | 2.63 | 1.6367 | 1.9167 | 0.2278 | 0.291269 |
| gga-miR-6561-5p |  | 0 | 0 | 0 | 0 | 0.55 | 0 | 0 | 0.1833 | 4.1961 | 0.293604 |
| gga-miR-6555-5p |  | 0.79 | 1.24 | 0.5 | 0.52 | 1.38 | 1.55 | 0.8367 | 1.15 | 0.4589 | 0.294475 |
| gga-miR-3524a |  | 1.38 | 0.78 | 1.3 | 2.49 | 0.55 | 1.24 | 1.1433 | 1.4267 | 0.3195 | 0.297096 |
| gga-miR-1564-5p |  | 0 | 0.31 | 0.2 | 0 | 0 | 0 | 0.1567 | 0 | -3.9699 | 0.297277 |
| gga-miR-137-5p |  | 0.39 | 0.47 | 0 | 0.13 | 0 | 0 | 0.2867 | 0.0433 | -2.7271 | 0.298124 |
| gga-miR-2127 |  | 0 | 0 | 0 | 0.52 | 0 | 0 | 0 | 0.1733 | 4.1152 | 0.302938 |
| gga-miR-7447-5p |  | 0 | 0 | 0 | 0.13 | 0.28 | 0 | 0 | 0.1367 | 3.7729 | 0.303601 |
| gga-miR-30c-5p |  | 5465 | 4928 | ## | 2887 | 3434 | 4391 | 5007.27 | 3570.663 | -0.4878 | 0.304337 |
| gga-miR-1741 |  | 0 | 0 | 0 | 0.13 | 0.28 | 0 | 0 | 0.1367 | 3.7729 | 0.304438 |
| gga-miR-6589-5p |  | 0 | 0 | 0 | 0.13 | 0.28 | 0 | 0 | 0.1367 | 3.7729 | 0.304841 |
| gga-miR-1775-3p |  | 0 | 0 | 0 | 0.13 | 0.28 | 0 | 0 | 0.1367 | 3.7729 | 0.305691 |
| gga-miR-1676-5p |  | 0 | 0.31 | 0 | 0.26 | 0 | 0.77 | 0.1033 | 0.3433 | 1.7326 | 0.311556 |
| gga-miR-4732-5p |  | 0.39 | 0.16 | 0 | 0 | 0 | 0 | 0.1833 | 0 | -4.1961 | 0.314043 |
| gga-miR-10a-5p |  | 4041 | 3776 | ## | 2222 | 2698 | 3475 | 3912.81 | 2798.403 | -0.4836 | 0.314454 |
| gga-miR-29c-5p |  | 0.39 | 0 | 0.2 | 0 | 0 | 0 | 0.1833 | 0 | -4.1961 | 0.314797 |
| gga-miR-7443-5p |  | 2.95 | 1.24 | 4.8 | 1.83 | 1.65 | 1.85 | 2.9867 | 1.7767 | -0.7494 | 0.317005 |
| gga-miR-126-3p |  | 2814 | 2605 | ## | 2555 | 2350 | 2410 | 2591.26 | 2438.327 | -0.0878 | 0.317675 |
| gga-miR-6549-5p |  | 2.17 | 1.71 | 2.1 | 1.44 | 0.83 | 1.24 | 1.9833 | 1.17 | -0.7614 | 0.319652 |
| gga-miR-1560-5p |  | 0.39 | 0.16 | 0.5 | 0.13 | 0 | 0.15 | 0.3433 | 0.0933 | -1.8795 | 0.326638 |
| gga-miR-1703-5p |  | 3.74 | 3.73 | 2.4 | 1.57 | 2.07 | 2.63 | 3.2867 | 2.09 | -0.6531 | 0.328345 |
| gga-miR-184-3p |  | 706.6 | 618 | 646 | 430.5 | 430 | 564 | 657.04 | 474.8667 | -0.4685 | 0.328352 |
| gga-miR-1708 |  | 0.59 | 1.09 | 0.2 | 0.92 | 0.69 | 0.93 | 0.6133 | 0.8467 | 0.4653 | 0.329048 |
| gga-miR-1582 |  | 0 | 0.62 | 0.2 | 0 | 0 | 0.15 | 0.26 | 0.05 | -2.3785 | 0.33447 |
| gga-miR-1680-5p |  | 0.2 | 0 | 0 | 0 | 0.14 | 0.62 | 0.0667 | 0.2533 | 1.9251 | 0.336349 |
| gga-miR-6566-5p |  | 1.18 | 1.09 | 0.5 | 0.92 | 1.1 | 1.39 | 0.9167 | 1.1367 | 0.3103 | 0.336584 |
| gga-miR-7477-3p |  | 0.39 | 0 | 1 | 0 | 0.28 | 0.15 | 0.4467 | 0.1433 | -1.6403 | 0.339496 |
| gga-miR-138-2-3p |  | 16.73 | 14.3 | 12 | 8.64 | 10.6 | 10.8 | 14.3167 | 10.0233 | -0.5143 | 0.341761 |
| gga-miR-1729-5p |  | 13.58 | 18.5 | 14 | 18.59 | 14.3 | 12.2 | 15.4067 | 15.04 | -0.0348 | 0.343026 |
| gga-miR-214 |  | 150 | 175 | 213 | 143.5 | 120 | 127 | 179.18 | 130.1167 | -0.4616 | 0.350551 |
| gga-miR-429-5p |  | 1.38 | 0.31 | 0.2 | 0.26 | 0.41 | 0 | 0.6167 | 0.2233 | -1.4656 | 0.351282 |
| gga-miR-1655-5p |  | 0.79 | 0.47 | 0.5 | 0 | 0.28 | 0.46 | 0.58 | 0.2467 | -1.2333 | 0.352974 |
| gga-miR-1552-5p |  | 287.5 | 329 | 310 | 240 | 266 | 372 | 308.9 | 292.87 | -0.0769 | 0.353048 |
| gga-miR-155 |  | 63.99 | 49.9 | 41 | 26.31 | 38.6 | 43.4 | 51.5233 | 36.1 | -0.5132 | 0.353636 |
| gga-miR-199-5p |  | 2242 | 1374 | ## | 1108 | 1156 | 1319 | 1672.19 | 1194.317 | -0.4856 | 0.353915 |
| gga-miR-7465-3p |  | 3.35 | 2.33 | 1.4 | 2.88 | 3.44 | 1.55 | 2.37 | 2.6233 | 0.1465 | 0.35426 |
| gga-miR-130b-5p |  | 12.01 | 23.8 | 19 | 11.13 | 12.5 | 14.4 | 18.2867 | 12.6767 | -0.5286 | 0.354472 |
| gga-miR-6648-3p |  | 3.15 | 4.5 | 4.6 | 3.4 | 2.75 | 6.96 | 4.0867 | 4.37 | 0.0967 | 0.355059 |
| gga-miR-1467-3p |  | 1.18 | 0.93 | 0.8 | 1.7 | 0.83 | 0.93 | 0.97 | 1.1533 | 0.2497 | 0.355421 |
| gga-miR-1813 |  | 1.38 | 1.24 | 0.8 | 0.52 | 0.83 | 0.46 | 1.14 | 0.6033 | -0.9181 | 0.355954 |
| gga-miR-16-2-3p |  | 1.18 | 1.86 | 2.1 | 1.96 | 2.07 | 1.55 | 1.7033 | 1.86 | 0.127 | 0.361045 |
| gga-miR-1551-3p |  | 0 | 0.31 | 1.1 | 0.65 | 0.55 | 0.77 | 0.4733 | 0.6567 | 0.4725 | 0.381333 |
| gga-miR-1683 |  | 0.98 | 1.09 | 0.3 | 0.52 | 0 | 0.62 | 0.7967 | 0.38 | -1.068 | 0.387065 |
| gga-miR-1453 |  | 0.98 | 1.55 | 1 | 1.57 | 1.38 | 1.08 | 1.16 | 1.3433 | 0.2117 | 0.38795 |
| gga-miR-6552-5p |  | 1.18 | 1.24 | 0.8 | 0.65 | 1.1 | 0 | 1.0733 | 0.5833 | -0.8797 | 0.393883 |
| gga-miR-6659-3p |  | 1.77 | 1.24 | 3 | 1.96 | 1.24 | 3.4 | 2.01 | 2.2 | 0.1303 | 0.396126 |
| gga-miR-302d |  | 0.2 | 0 | 0.8 | 0.13 | 0 | 0.15 | 0.3333 | 0.0933 | -1.8369 | 0.396868 |
| gga-miR-125b-5p |  | 3927 | 4603 | ## | 3628 | 3227 | 3251 | 4533.43 | 3368.763 | -0.4284 | 0.400592 |
| gga-miR-1664-3p |  | 1.77 | 3.42 | 3.2 | 1.7 | 1.65 | 2.16 | 2.79 | 1.8367 | -0.6031 | 0.400779 |
| gga-miR-6546-5p |  | 1.18 | 0.93 | 3 | 3.4 | 1.1 | 1.39 | 1.71 | 1.9633 | 0.1993 | 0.406971 |
| gga-miR-7456-3p |  | 0 | 0 | 0.2 | 0.26 | 0.14 | 0.15 | 0.0533 | 0.1833 | 1.782 | 0.407204 |
| gga-miR-6547-3p |  | 0.2 | 0.16 | 0.3 | 0.13 | 0 | 0 | 0.2267 | 0.0433 | -2.3883 | 0.408554 |
| gga-miR-181a-3p |  | 45.68 | 40.1 | 41 | 37.96 | 40.5 | 39.1 | 42.3667 | 39.1867 | -0.1126 | 0.408737 |
| gga-miR-6551-3p |  | 0.2 | 0.62 | 1.4 | 0 | 0.28 | 0.77 | 0.75 | 0.35 | -1.0995 | 0.409685 |
| gga-miR-92-3p |  | 6298 | 6872 | ## | 4305 | 4575 | 5205 | 6308.79 | 4694.883 | -0.4263 | 0.413016 |
| gga-miR-6558-3p |  | 0 | 0 | 0.2 | 0.13 | 0.28 | 0.15 | 0.0533 | 0.1867 | 1.8085 | 0.413852 |
| gga-miR-1600 |  | 0 | 0 | 0.5 | 0 | 0 | 0 | 0.16 | 0 | -4 | 0.413907 |
| gga-miR-6633-5p |  | 0.2 | 0 | 0 | 0.13 | 0.28 | 0.15 | 0.0667 | 0.1867 | 1.485 | 0.415329 |
| gga-miR-1602 |  | 0 | 0 | 0.5 | 0 | 0 | 0 | 0.16 | 0 | -4 | 0.416719 |
| gga-miR-200a-5p |  | 444.4 | 493 | 534 | 456.4 | 469 | 419 | 490.417 | 448.08 | -0.1303 | 0.416941 |
| gga-miR-6608-5p |  | 0 | 0 | 0.5 | 0 | 0 | 0 | 0.16 | 0 | -4 | 0.418529 |
| gga-miR-1608 |  | 0 | 0 | 0 | 0.39 | 0 | 0 | 0 | 0.13 | 3.7004 | 0.420827 |
| gga-miR-130b-3p |  | 5078 | 4720 | ## | 4094 | 4210 | 5034 | 4845.29 | 4446.013 | -0.1241 | 0.421152 |
| gga-miR-30e-5p |  | 1280 | 1136 | ## | 966.1 | 1090 | 1102 | 1140.57 | 1052.857 | -0.1154 | 0.426604 |
| gga-miR-490-5p |  | 0.39 | 1.4 | 1 | 0.92 | 0.14 | 0.31 | 0.9133 | 0.4567 | -0.9998 | 0.428439 |
| gga-miR-1736-5p |  | 0.79 | 0 | 0 | 0.13 | 0 | 0 | 0.2633 | 0.0433 | -2.6043 | 0.429871 |
| gga-miR-6543-3p |  | 2.76 | 1.55 | 2.7 | 1.44 | 1.65 | 1.39 | 2.3367 | 1.4933 | -0.646 | 0.434533 |
| gga-miR-7462-5p |  | 0 | 0.16 | 0 | 0 | 0.28 | 0.31 | 0.0533 | 0.1967 | 1.8838 | 0.445941 |
| gga-miR-6590-3p |  | 1.18 | 2.02 | 1.4 | 1.83 | 1.38 | 1.7 | 1.5433 | 1.6367 | 0.0848 | 0.44703 |
| gga-miR-183 |  | 57.88 | 64.5 | 61 | 54.58 | 55 | 57.2 | 60.98 | 55.5733 | -0.1339 | 0.449661 |
| gga-miR-1556 |  | 0.2 | 0.47 | 0 | 0.13 | 0 | 0 | 0.2233 | 0.0433 | -2.3665 | 0.458717 |
| gga-miR-100-3p |  | 0 | 0.47 | 0.2 | 0.65 | 0.14 | 0.31 | 0.21 | 0.3667 | 0.8042 | 0.460809 |
| gga-miR-6594-5p |  | 0.39 | 0.16 | 0 | 0 | 0.28 | 0.77 | 0.1833 | 0.35 | 0.9331 | 0.462014 |
| gga-miR-1689-5p |  | 0 | 0 | 0.2 | 0.39 | 0 | 0.15 | 0.0533 | 0.18 | 1.7558 | 0.463578 |
| gga-miR-1591-3p |  | 0 | 0.16 | 0.5 | 0.13 | 0 | 0 | 0.2133 | 0.0433 | -2.3004 | 0.464473 |
| gga-miR-122-3p |  | 0 | 0.16 | 0.5 | 0.13 | 0 | 0 | 0.2133 | 0.0433 | -2.3004 | 0.464633 |
| gga-miR-6593-5p |  | 0 | 0.16 | 0.5 | 0.13 | 0 | 0 | 0.2133 | 0.0433 | -2.3004 | 0.46515 |
| gga-miR-1801 |  | 0 | 0.16 | 0 | 0 | 0.14 | 0.46 | 0.0533 | 0.2 | 1.9078 | 0.470545 |
| gga-miR-18a-5p |  | 29.73 | 28.4 | 38 | 25.91 | 28.9 | 33.7 | 32.0533 | 29.51 | -0.1193 | 0.470621 |
| gga-miR-1684b-3p |  | 235.5 | 248 | 241 | 179.6 | 165 | 201 | 241.33 | 181.9733 | -0.4073 | 0.472378 |
| gga-miR-9-5p |  | 25.2 | 20.7 | 23 | 28.79 | 19 | 16.4 | 22.8133 | 21.3933 | -0.0927 | 0.478509 |
| gga-miR-1736-3p |  | 1.58 | 4.19 | 2.4 | 2.09 | 2.2 | 1.08 | 2.72 | 1.79 | -0.6036 | 0.480978 |
| gga-miR-1668-5p |  | 0.2 | 0.31 | 0.3 | 0.13 | 0.14 | 0 | 0.2767 | 0.09 | -1.6203 | 0.481952 |
| gga-miR-1609 |  | 0 | 0.62 | 0.2 | 0.39 | 0.55 | 0.31 | 0.26 | 0.4167 | 0.6805 | 0.483308 |
| gga-miR-1451-5p |  | 25.79 | 16.8 | 23 | 18.85 | 15.7 | 13.8 | 21.98 | 16.1033 | -0.4488 | 0.484161 |
| gga-miR-30d |  | 9040 | #### | ## | 6586 | 8644 | ### | 9485.35 | 8679.943 | -0.128 | 0.487551 |
| gga-miR-1805-5p |  | 0.39 | 0.78 | 0 | 0 | 0.41 | 0 | 0.39 | 0.1367 | -1.5125 | 0.487895 |
| gga-miR-1650 |  | 0.79 | 0.93 | 1 | 0.26 | 0.41 | 0.93 | 0.89 | 0.5333 | -0.7389 | 0.490986 |
| gga-miR-135a-2-3p |  | 12.21 | 20.7 | 21 | 14.4 | 14.2 | 10.7 | 17.9 | 13.0867 | -0.4519 | 0.493864 |
| gga-miR-221-5p |  | 714.9 | 389 | 413 | 273.2 | 392 | 442 | 505.607 | 368.82 | -0.4551 | 0.4955 |
| gga-miR-1764-3p |  | 4.13 | 5.28 | 8.1 | 4.58 | 3.86 | 3.86 | 5.84 | 4.1 | -0.5103 | 0.498036 |
| gga-miR-365-2-5p |  | 64.38 | 54.7 | 52 | 38.48 | 36.5 | 52.6 | 57.02 | 42.51 | -0.4237 | 0.50446 |
| gga-miR-135a-5p |  | 530 | 401 | 451 | 404.6 | 410 | 438 | 460.543 | 417.44 | -0.1418 | 0.506297 |
| gga-miR-7452-5p |  | 0.2 | 1.09 | 0 | 0.26 | 0.14 | 0.15 | 0.43 | 0.1833 | -1.2301 | 0.507036 |
| gga-miR-103-2-5p |  | 0 | 0.16 | 0.2 | 0 | 0 | 0 | 0.1067 | 0 | -3.4155 | 0.509022 |
| gga-miR-1668-3p |  | 0.59 | 0.31 | 1.6 | 1.18 | 1.24 | 0.46 | 0.83 | 0.96 | 0.2099 | 0.510807 |
| gga-miR-6564-5p |  | 0 | 0.16 | 0.2 | 0 | 0 | 0 | 0.1067 | 0 | -3.4155 | 0.511206 |
| gga-miR-1618-5p |  | 0 | 0.47 | 0.3 | 0 | 0.14 | 0.15 | 0.2633 | 0.0967 | -1.4451 | 0.514142 |
| gga-miR-6516-3p |  | 0.2 | 0.16 | 0 | 0 | 0.41 | 0.31 | 0.12 | 0.24 | 1 | 0.514274 |
| gga-miR-128-2-5p |  | 1.38 | 0.47 | 1.3 | 1.44 | 1.1 | 0.77 | 1.04 | 1.1033 | 0.0852 | 0.514691 |
| gga-miR-6654-3p |  | 0 | 0 | 0 | 0.13 | 0.14 | 0 | 0 | 0.09 | 3.1699 | 0.514807 |
| gga-miR-6709-5p |  | 0 | 0.16 | 0.2 | 0 | 0 | 0 | 0.1067 | 0 | -3.4155 | 0.515153 |
| gga-miR-1780 |  | 0 | 0 | 0 | 0.13 | 0.14 | 0 | 0 | 0.09 | 3.1699 | 0.515165 |
| gga-miR-7b |  | 331.9 | 195 | 176 | 195.5 | 213 | 251 | 234.15 | 219.59 | -0.0926 | 0.515668 |
| gga-miR-456-5p |  | 0 | 0 | 0 | 0.13 | 0.14 | 0 | 0 | 0.09 | 3.1699 | 0.516708 |
| gga-miR-1812-5p |  | 0 | 0 | 0 | 0.13 | 0 | 0.15 | 0 | 0.0933 | 3.2219 | 0.518608 |
| gga-miR-1648-3p |  | 0.2 | 0 | 0.2 | 0 | 0 | 0 | 0.12 | 0 | -3.585 | 0.519099 |
| gga-miR-1730-3p |  | 0 | 0 | 0 | 0.13 | 0 | 0.15 | 0 | 0.0933 | 3.2219 | 0.519263 |
| gga-miR-3531-5p |  | 0 | 0 | 0 | 0 | 0.14 | 0.15 | 0 | 0.0967 | 3.2735 | 0.520083 |
| gga-miR-1649-3p |  | 0 | 0 | 0 | 0 | 0.14 | 0.15 | 0 | 0.0967 | 3.2735 | 0.520324 |
| gga-miR-1763 |  | 0.2 | 0.16 | 0 | 0 | 0 | 0 | 0.12 | 0 | -3.585 | 0.520661 |
| gga-miR-3524b-3p |  | 0.2 | 0 | 0.2 | 0 | 0 | 0 | 0.12 | 0 | -3.585 | 0.521159 |
| gga-miR-7460-5p |  | 0.2 | 0 | 0.2 | 0 | 0 | 0 | 0.12 | 0 | -3.585 | 0.521243 |
| gga-miR-7442-5p |  | 0.2 | 0.47 | 0.3 | 0.92 | 0.28 | 0.15 | 0.33 | 0.45 | 0.4475 | 0.52693 |
| gga-miR-20a-5p |  | 845.4 | 823 | 950 | 763.1 | 820 | 757 | 872.487 | 779.7633 | -0.1621 | 0.529739 |
| gga-miR-1740-5p |  | 0.79 | 0.31 | 1.1 | 0.65 | 0.96 | 0.93 | 0.7367 | 0.8467 | 0.2008 | 0.530718 |
| gga-miR-6669-3p |  | 1.97 | 0.78 | 1.6 | 1.31 | 0.69 | 0.62 | 1.4467 | 0.8733 | -0.7282 | 0.531635 |
| gga-miR-29b-1-5p |  | 11.81 | 10.1 | 8.4 | 3.66 | 7.16 | 10.7 | 10.1133 | 7.1633 | -0.4976 | 0.53433 |
| gga-miR-1685-5p |  | 0.59 | 0.47 | 0.3 | 0.39 | 0.69 | 0.62 | 0.46 | 0.5667 | 0.301 | 0.538733 |
| gga-miR-1677-3p |  | 56.31 | 58.9 | 67 | 50.39 | 55.6 | 56.9 | 60.71 | 54.3033 | -0.1609 | 0.545759 |
| gga-miR-6669-5p |  | 1.18 | 0 | 0 | 0.26 | 0.14 | 0 | 0.3933 | 0.1333 | -1.561 | 0.549932 |
| gga-miR-6542-3p |  | 75.21 | 78.3 | 83 | 68.58 | 68.9 | 73.4 | 78.8367 | 70.29 | -0.1655 | 0.551041 |
| gga-miR-1578 |  | 0.2 | 0 | 0 | 0 | 0 | 0.62 | 0.0667 | 0.2067 | 1.6318 | 0.553225 |
| gga-miR-6575-5p |  | 1.18 | 1.55 | 0.6 | 0.92 | 0.55 | 0.62 | 1.1233 | 0.6967 | -0.6891 | 0.557411 |
| gga-let-7g-3p |  | 3.54 | 3.73 | 4.3 | 3.53 | 3.03 | 1.55 | 3.8533 | 2.7033 | -0.5114 | 0.560907 |
| gga-miR-6516-5p |  | 3.35 | 2.64 | 4 | 3.93 | 4.41 | 1.55 | 3.3233 | 3.2967 | -0.0116 | 0.56148 |
| gga-miR-6623-3p |  | 0.2 | 0.31 | 0 | 0.13 | 0.55 | 0.15 | 0.17 | 0.2767 | 0.7028 | 0.563042 |
| gga-miR-130a-5p |  | 13.98 | 10.1 | 13 | 5.63 | 9.64 | 11.8 | 12.2667 | 9.0067 | -0.4457 | 0.567651 |
| gga-miR-1626-3p |  | 0.39 | 0.62 | 0.6 | 0.65 | 0.69 | 0.62 | 0.55 | 0.6533 | 0.2483 | 0.572865 |
| gga-miR-1603 |  | 0.79 | 0.31 | 0 | 0.13 | 0.14 | 0.15 | 0.3667 | 0.14 | -1.3892 | 0.573888 |
| gga-miR-1692 |  | 0.39 | 0 | 0.2 | 0.39 | 0.41 | 0 | 0.1833 | 0.2667 | 0.541 | 0.575283 |
| gga-miR-6606-5p |  | 0.79 | 0.16 | 0.2 | 0.92 | 0.41 | 0 | 0.37 | 0.4433 | 0.2608 | 0.581495 |
| gga-miR-1769-5p |  | 0.39 | 0.31 | 0 | 0.26 | 0.41 | 0.31 | 0.2333 | 0.3267 | 0.4858 | 0.581624 |
| gga-miR-3530-5p |  | 1.58 | 2.17 | 1.9 | 0.92 | 1.24 | 1.7 | 1.8867 | 1.2867 | -0.5522 | 0.582768 |
| gga-miR-1663-5p |  | 5.12 | 4.97 | 4.9 | 2.62 | 2.48 | 5.56 | 5.0067 | 3.5533 | -0.4947 | 0.585853 |
| gga-miR-3525 |  | 12.99 | 12.4 | 11 | 9.29 | 13.6 | 10.7 | 12.29 | 11.2 | -0.134 | 0.586053 |
| gga-miR-6574-3p |  | 0.59 | 0.16 | 0.3 | 0 | 0 | 0.46 | 0.3567 | 0.1533 | -1.2184 | 0.586368 |
| gga-miR-100-5p |  | 13037 | #### | ## | 10542 | ### | ### | 14617.8 | 11266.68 | -0.3757 | 0.586772 |
| gga-miR-1560-3p |  | 59.85 | 71.1 | 80 | 54.19 | 64.7 | 68.9 | 70.3267 | 62.62 | -0.1674 | 0.588999 |
| gga-miR-1329-3p |  | 0.2 | 0.62 | 0.2 | 0 | 0.41 | 0 | 0.3267 | 0.1367 | -1.257 | 0.591617 |
| gga-miR-1464 |  | 3.74 | 4.35 | 4.5 | 2.49 | 5.51 | 4.02 | 4.18 | 4.0067 | -0.0611 | 0.594924 |
| gga-miR-1612 |  | 0.98 | 0.62 | 0.2 | 0.52 | 0.55 | 0.93 | 0.5867 | 0.6667 | 0.1844 | 0.596773 |
| gga-miR-6647-5p |  | 0 | 0 | 0 | 0.26 | 0 | 0 | 0 | 0.0867 | 3.116 | 0.597163 |
| gga-miR-6572-3p |  | 0 | 0 | 0 | 0 | 0 | 0.31 | 0 | 0.1033 | 3.3688 | 0.597495 |
| gga-miR-6577-5p |  | 0 | 0 | 0 | 0 | 0.28 | 0 | 0 | 0.0933 | 3.2219 | 0.598147 |
| gga-miR-429-3p |  | 1849 | 1512 | ## | 1180 | 1398 | 1342 | 1690.47 | 1306.437 | -0.3718 | 0.598259 |
| gga-miR-1623 |  | 0 | 0 | 0 | 0 | 0.28 | 0 | 0 | 0.0933 | 3.2219 | 0.598648 |
| gga-miR-1657 |  | 0 | 0 | 0 | 0 | 0 | 0.31 | 0 | 0.1033 | 3.3688 | 0.59922 |
| gga-miR-1631 |  | 0 | 0 | 0 | 0.26 | 0 | 0 | 0 | 0.0867 | 3.116 | 0.599276 |
| gga-let-7i |  | 15375 | #### | ## | 10909 | ### | ### | 14606.9 | 11343 | -0.3648 | 0.599292 |
| gga-miR-1663-3p |  | 9.06 | 11.7 | 10 | 9.95 | 6.61 | 11.6 | 10.2433 | 9.3833 | -0.1265 | 0.59964 |
| gga-miR-1742 |  | 0 | 0.31 | 0 | 0 | 0 | 0 | 0.1033 | 0 | -3.3688 | 0.599927 |
| gga-miR-1606 |  | 0.39 | 0 | 0 | 0 | 0 | 0 | 0.13 | 0 | -3.7004 | 0.600571 |
| gga-miR-6682-3p |  | 0.39 | 0 | 0 | 0 | 0 | 0 | 0.13 | 0 | -3.7004 | 0.601865 |
| gga-miR-1731-3p |  | 0 | 0.31 | 0 | 0 | 0 | 0 | 0.1033 | 0 | -3.3688 | 0.60199 |
| gga-miR-1672 |  | 0 | 0.31 | 0 | 0 | 0 | 0 | 0.1033 | 0 | -3.3688 | 0.603849 |
| gga-miR-1640 |  | 0.2 | 0 | 0.2 | 0 | 0 | 0.77 | 0.12 | 0.2567 | 1.097 | 0.604216 |
| gga-miR-19b-3p |  | 124.2 | 75.6 | 75 | 62.17 | 80 | 110 | 91.54 | 84.08 | -0.1226 | 0.605772 |
| gga-miR-6688-3p |  | 0 | 0 | 0.3 | 0 | 0 | 0 | 0.1067 | 0 | -3.4155 | 0.605845 |
| gga-miR-6581-5p |  | 0 | 0.31 | 0 | 0 | 0 | 0 | 0.1033 | 0 | -3.3688 | 0.605941 |
| gga-miR-1591-5p |  | 0 | 0.16 | 0.5 | 0.13 | 0.28 | 0.62 | 0.2133 | 0.3433 | 0.6866 | 0.606603 |
| gga-miR-1607 |  | 0 | 0.31 | 0 | 0 | 0 | 0 | 0.1033 | 0 | -3.3688 | 0.606967 |
| gga-miR-1564-3p |  | 0 | 0 | 0.3 | 0 | 0 | 0 | 0.1067 | 0 | -3.4155 | 0.60701 |
| gga-miR-221-3p |  | 28431 | #### | ## | 16331 | ### | ### | 25560.8 | 19707.85 | -0.3752 | 0.607448 |
| gga-miR-6673-3p |  | 0 | 0 | 0.3 | 0 | 0 | 0 | 0.1067 | 0 | -3.4155 | 0.607465 |
| gga-miR-6658-3p |  | 0 | 0.31 | 0 | 0 | 0 | 0 | 0.1033 | 0 | -3.3688 | 0.60999 |
| gga-miR-1707 |  | 0.79 | 1.24 | 0.5 | 0.26 | 0.83 | 1.7 | 0.8367 | 0.93 | 0.1525 | 0.614689 |
| gga-miR-1706 |  | 0.39 | 1.4 | 0.5 | 0.26 | 0.41 | 0.77 | 0.7567 | 0.48 | -0.6567 | 0.615596 |
| gga-miR-6675-5p |  | 0.59 | 0.16 | 0.2 | 0.52 | 0.28 | 0.31 | 0.3033 | 0.37 | 0.2868 | 0.618295 |
| gga-miR-15c-3p |  | 1.18 | 0.78 | 1.4 | 0.65 | 1.79 | 1.08 | 1.13 | 1.1733 | 0.0542 | 0.618907 |
| gga-miR-6572-5p |  | 0.59 | 0.31 | 0.5 | 0 | 0.28 | 0.46 | 0.46 | 0.2467 | -0.8989 | 0.621942 |
| gga-miR-1737 |  | 0.39 | 0.78 | 0.2 | 0.26 | 0.14 | 0.31 | 0.4433 | 0.2367 | -0.9052 | 0.622618 |
| gga-miR-6544-3p |  | 0 | 0.47 | 0.6 | 0.39 | 0.14 | 0 | 0.37 | 0.1767 | -1.0662 | 0.624057 |
| gga-miR-3537 |  | 0 | 0.62 | 0.5 | 0.13 | 0.41 | 0 | 0.3667 | 0.18 | -1.0266 | 0.625632 |
| gga-miR-222a |  | 6654 | 7966 | ## | 4237 | 5934 | 9143 | 7160.89 | 6438.13 | -0.1535 | 0.627136 |
| gga-miR-1711 |  | 1.18 | 1.71 | 1.1 | 1.44 | 1.65 | 0.93 | 1.3333 | 1.34 | 0.0072 | 0.635245 |
| gga-miR-6696-5p |  | 0 | 0.47 | 0.2 | 0 | 0.69 | 0.31 | 0.21 | 0.3333 | 0.6664 | 0.63837 |
| gga-miR-1625-5p |  | 10.04 | 7.92 | 11 | 7.07 | 11.4 | 8.19 | 9.8033 | 8.8967 | -0.14 | 0.640591 |
| gga-miR-7475-5p |  | 0.2 | 0.47 | 0.8 | 0.13 | 0.28 | 0.46 | 0.49 | 0.29 | -0.7567 | 0.644268 |
| gga-miR-133a-5p |  | 0.79 | 1.4 | 1.6 | 1.57 | 1.79 | 0.46 | 1.26 | 1.2733 | 0.0151 | 0.644371 |
| gga-miR-1580 |  | 0.59 | 1.4 | 1.3 | 0.79 | 0.69 | 0.62 | 1.0867 | 0.7 | -0.6345 | 0.648686 |
| gga-miR-1613 |  | 0.79 | 0 | 0 | 0 | 0 | 0.31 | 0.2633 | 0.1033 | -1.3499 | 0.648729 |
| gga-miR-449a |  | 4.33 | 4.04 | 6.2 | 5.76 | 5.51 | 2.32 | 4.8567 | 4.53 | -0.1005 | 0.65325 |
| gga-miR-301a-5p |  | 14.18 | 13.8 | 21 | 14.79 | 14.5 | 14.8 | 16.49 | 14.6967 | -0.1661 | 0.658091 |
| gga-miR-6656-5p |  | 0.39 | 0.47 | 0 | 0.39 | 0.69 | 0 | 0.2867 | 0.36 | 0.3285 | 0.661666 |
| gga-miR-6609-3p |  | 0.39 | 1.24 | 0.8 | 0.92 | 1.24 | 0.31 | 0.81 | 0.8233 | 0.0235 | 0.662142 |
| gga-miR-489-3p |  | 5.12 | 3.57 | 3.2 | 2.36 | 2.62 | 3.71 | 3.9567 | 2.8967 | -0.4499 | 0.664421 |
| gga-miR-6708-5p |  | 0 | 0.16 | 0 | 0.26 | 0.14 | 0 | 0.0533 | 0.1333 | 1.3225 | 0.667707 |
| gga-miR-6547-5p |  | 0 | 0.31 | 0.2 | 0 | 0.14 | 0 | 0.1567 | 0.0467 | -1.7465 | 0.669587 |
| gga-miR-1611 |  | 0.2 | 0.31 | 0 | 0 | 0.14 | 0 | 0.17 | 0.0467 | -1.864 | 0.669758 |
| gga-miR-7476-3p |  | 0 | 0.16 | 0.3 | 0 | 0 | 0.15 | 0.16 | 0.05 | -1.6781 | 0.670535 |
| gga-miR-1550-3p |  | 0 | 0.31 | 0.2 | 0 | 0 | 0.15 | 0.1567 | 0.05 | -1.648 | 0.671056 |
| gga-miR-6694-3p |  | 3.54 | 1.09 | 1.3 | 1.96 | 1.93 | 1.7 | 1.9667 | 1.8633 | -0.0779 | 0.67223 |
| gga-miR-6655-5p |  | 0.39 | 3.26 | 1.8 | 1.83 | 0.69 | 1.08 | 1.8 | 1.2 | -0.585 | 0.673039 |
| gga-miR-7441-5p |  | 0 | 0.16 | 0 | 0.13 | 0 | 0.31 | 0.0533 | 0.1467 | 1.4607 | 0.674088 |
| gga-miR-1712-3p |  | 12.99 | 9.94 | 10 | 7.2 | 8.81 | 9.43 | 11.09 | 8.48 | -0.3871 | 0.675246 |
| gga-miR-6625-5p |  | 0.39 | 0.16 | 0 | 0 | 0.14 | 0 | 0.1833 | 0.0467 | -1.9727 | 0.675358 |
| gga-miR-6611-5p |  | 0.79 | 0.47 | 0.2 | 0.65 | 0.41 | 0.46 | 0.4733 | 0.5067 | 0.0984 | 0.680532 |
| gga-miR-2131-5p |  | 77.18 | 40.1 | 40 | 25.13 | 37.7 | 55.8 | 52.4967 | 39.5567 | -0.4083 | 0.681081 |
| gga-miR-3064-5p |  | 0.79 | 0.62 | 0 | 0.65 | 0.41 | 0.46 | 0.47 | 0.5067 | 0.1085 | 0.6914 |
| gga-miR-144-3p |  | 121.3 | 197 | 173 | 199.3 | 105 | 67.2 | 163.75 | 123.7533 | -0.404 | 0.695021 |
| gga-miR-30b-5p |  | 104.9 | 97.4 | 87 | 102.4 | 81.5 | 69.6 | 96.3333 | 84.4833 | -0.1894 | 0.706887 |
| gga-miR-1604 |  | 11.03 | 11.2 | 15 | 11.52 | 11.2 | 10.4 | 12.44 | 11.0133 | -0.1757 | 0.707731 |
| gga-let-7f-3p |  | 92.14 | 70.7 | 69 | 46.59 | 61.8 | 72.3 | 77.28 | 60.2567 | -0.359 | 0.710317 |
| gga-miR-6554-3p |  | 0.39 | 0.16 | 0.2 | 0.13 | 0.14 | 0 | 0.2367 | 0.09 | -1.3951 | 0.710666 |
| gga-miR-106-3p |  | 158.9 | 143 | 146 | 110.2 | 135 | 145 | 149.26 | 129.91 | -0.2003 | 0.713814 |
| gga-miR-18b-3p |  | 10.43 | 11 | 12 | 6.28 | 10.1 | 13.5 | 11.0767 | 9.9267 | -0.1581 | 0.714925 |
| gga-miR-1744-3p |  | 0.98 | 1.55 | 1 | 1.57 | 1.52 | 0.46 | 1.16 | 1.1833 | 0.0287 | 0.715085 |
| gga-miR-26a-3p |  | 4.73 | 3.88 | 2.5 | 1.96 | 3.31 | 5.1 | 3.7167 | 3.4567 | -0.1046 | 0.715199 |
| gga-miR-153-5p |  | 0.98 | 1.09 | 0.5 | 0.79 | 0.14 | 1.7 | 0.85 | 0.8767 | 0.0446 | 0.71546 |
| gga-miR-20b-5p |  | 395.7 | 394 | 463 | 346.3 | 374 | 363 | 417.423 | 360.8733 | -0.21 | 0.716217 |
| gga-miR-1467-5p |  | 1.77 | 0.93 | 1.9 | 1.05 | 0.69 | 2.78 | 1.5367 | 1.5067 | -0.0284 | 0.717861 |
| gga-miR-1416-5p |  | 225 | 348 | 343 | 224.6 | 227 | 264 | 305.333 | 238.58 | -0.3559 | 0.720013 |
| gga-miR-1465 |  | 0 | 0 | 0.3 | 0.13 | 0.28 | 0.15 | 0.1067 | 0.1867 | 0.8072 | 0.720676 |
| gga-miR-7445-3p |  | 0.2 | 0 | 0.2 | 0.26 | 0 | 0.31 | 0.12 | 0.19 | 0.663 | 0.721069 |
| gga-miR-23b-3p |  | 2243 | 1926 | ## | 1542 | 1630 | 1685 | 2043.84 | 1618.84 | -0.3363 | 0.725559 |
| gga-miR-146b-3p |  | 49.22 | 55.5 | 54 | 42.01 | 52.2 | 43.3 | 52.81 | 45.83 | -0.2045 | 0.727076 |
| gga-miR-1747-3p |  | 0.2 | 0 | 0.5 | 0.13 | 0.14 | 0 | 0.2267 | 0.09 | -1.3328 | 0.730064 |
| gga-miR-1772-5p |  | 0 | 0.47 | 0.2 | 0.13 | 0 | 0.15 | 0.21 | 0.0933 | -1.1704 | 0.732073 |
| gga-miR-489-5p |  | 0 | 0 | 0.2 | 0.39 | 0 | 0 | 0.0533 | 0.13 | 1.2863 | 0.73416 |
| gga-miR-216b |  | 3.35 | 2.64 | 2.1 | 2.36 | 1.79 | 1.7 | 2.6867 | 1.95 | -0.4624 | 0.73541 |
| gga-miR-1716 |  | 0.39 | 1.71 | 1.3 | 1.96 | 0.55 | 0.77 | 1.1233 | 1.0933 | -0.0391 | 0.739646 |
| gga-miR-30a-5p |  | 8633 | 6988 | ## | 5740 | 7011 | 6428 | 7363.37 | 6393.247 | -0.2038 | 0.74418 |
| gga-miR-1593 |  | 0.39 | 0.78 | 0 | 0.39 | 0 | 1.08 | 0.39 | 0.49 | 0.3293 | 0.745934 |
| gga-miR-6666-3p |  | 0.2 | 0.31 | 0.3 | 0 | 0.28 | 0.15 | 0.2767 | 0.1433 | -0.9493 | 0.746406 |
| gga-miR-1728-3p |  | 0.2 | 0.31 | 0.3 | 0.26 | 0 | 0.15 | 0.2767 | 0.1367 | -1.0173 | 0.746555 |
| gga-miR-1306-5p |  | 3.15 | 5.75 | 4.6 | 3.14 | 4.82 | 4.33 | 4.5033 | 4.0967 | -0.1365 | 0.749772 |
| gga-miR-6610-3p |  | 0 | 0 | 0.3 | 0.39 | 0 | 0.15 | 0.1067 | 0.18 | 0.7544 | 0.751834 |
| gga-miR-1581 |  | 0.39 | 0 | 0 | 0 | 0.41 | 0.15 | 0.13 | 0.1867 | 0.5222 | 0.75345 |
| gga-miR-1452 |  | 0.39 | 0 | 0.2 | 0.13 | 0.41 | 0.15 | 0.1833 | 0.23 | 0.3274 | 0.753545 |
| gga-miR-1329-5p |  | 46.07 | 44 | 32 | 33.64 | 34.9 | 28.1 | 40.7167 | 32.2067 | -0.3383 | 0.753584 |
| gga-miR-6573-5p |  | 0 | 0.47 | 0.2 | 0 | 0.28 | 0 | 0.21 | 0.0933 | -1.1704 | 0.755626 |
| gga-miR-1781-3p |  | 5.12 | 5.44 | 5.4 | 4.58 | 5.1 | 4.48 | 5.3233 | 4.72 | -0.1735 | 0.756655 |
| gga-miR-6646-3p |  | 0.59 | 0.16 | 0 | 0 | 0.28 | 0 | 0.25 | 0.0933 | -1.422 | 0.757294 |
| gga-miR-222b-3p |  | 35.83 | 43.8 | 38 | 32.59 | 31.4 | 29.1 | 39.1067 | 31.0167 | -0.3344 | 0.760307 |
| gga-miR-6668-3p |  | 0 | 0.31 | 0.2 | 0 | 0.41 | 0.31 | 0.1567 | 0.24 | 0.615 | 0.763456 |
| gga-miR-1687-3p |  | 0 | 0.47 | 0 | 0.13 | 0.28 | 0.31 | 0.1567 | 0.24 | 0.615 | 0.763962 |
| gga-miR-1776 |  | 0.39 | 0 | 0.2 | 0.39 | 0.28 | 0 | 0.1833 | 0.2233 | 0.2848 | 0.764296 |
| gga-miR-1764-5p |  | 0 | 0.16 | 0.3 | 0.39 | 0 | 0.31 | 0.16 | 0.2333 | 0.5441 | 0.764339 |
| gga-miR-6599-5p |  | 0.59 | 0 | 0.3 | 0.13 | 0.28 | 0 | 0.3033 | 0.1367 | -1.1497 | 0.766023 |
| gga-miR-135a-1-3p |  | 7.88 | 7.61 | 10 | 11.13 | 6.47 | 4.95 | 8.5033 | 7.5167 | -0.1779 | 0.767166 |
| gga-miR-31-5p |  | 1484 | 1408 | ## | 1341 | 1226 | 1128 | 1545.61 | 1231.493 | -0.3278 | 0.77315 |
| gga-miR-1641 |  | 1.58 | 0.78 | 1.1 | 0.65 | 1.1 | 0.62 | 1.1567 | 0.79 | -0.5501 | 0.774455 |
| gga-miR-1568 |  | 0.2 | 0.62 | 0 | 0.13 | 0.28 | 0 | 0.2733 | 0.1367 | -0.9995 | 0.775753 |
| gga-miR-1745 |  | 0.2 | 0.16 | 0.6 | 0.13 | 0.28 | 0.15 | 0.3333 | 0.1867 | -0.8361 | 0.776819 |
| gga-miR-142-3p |  | 129 | 130 | 105 | 125.7 | 93 | 69.4 | 121.15 | 96.0067 | -0.3356 | 0.779071 |
| gga-miR-23b-5p |  | 1.97 | 2.17 | 1.8 | 1.05 | 1.65 | 2.78 | 1.9633 | 1.8267 | -0.104 | 0.783483 |
| gga-miR-6651-5p |  | 2.95 | 2.02 | 0.8 | 1.57 | 1.93 | 0.77 | 1.9233 | 1.4233 | -0.4343 | 0.786148 |
| gga-miR-1550-5p |  | 1.38 | 0.31 | 1.1 | 0.39 | 0.69 | 1.55 | 0.9333 | 0.8767 | -0.0903 | 0.7985 |
| gga-miR-1651-3p |  | 0.2 | 2.33 | 0.5 | 0.13 | 1.24 | 1.7 | 1.0033 | 1.0233 | 0.0285 | 0.799036 |
| gga-miR-1782 |  | 1.38 | 1.4 | 1.3 | 0.52 | 1.52 | 0.93 | 1.35 | 0.99 | -0.4475 | 0.799099 |
| gga-miR-301b-5p |  | 27.96 | 31.1 | 27 | 25.13 | 24.8 | 24 | 28.6867 | 24.6267 | -0.2202 | 0.800462 |
| gga-miR-1451-3p |  | 5.32 | 6.99 | 8.4 | 6.02 | 6.47 | 5.56 | 6.9133 | 6.0167 | -0.2004 | 0.8025 |
| gga-miR-1666 |  | 5.51 | 9.63 | 7.6 | 7.2 | 4.96 | 7.73 | 7.59 | 6.63 | -0.1951 | 0.804205 |
| gga-miR-1720-3p |  | 0.2 | 0.16 | 0.5 | 0.13 | 0.69 | 0.15 | 0.28 | 0.3233 | 0.2074 | 0.805589 |
| gga-miR-190a-5p |  | 1.58 | 1.24 | 1.1 | 0.79 | 1.38 | 1.55 | 1.31 | 1.24 | -0.0792 | 0.805629 |
| gga-miR-3539 |  | 0.2 | 0.47 | 0.3 | 0.26 | 0.28 | 0.62 | 0.33 | 0.3867 | 0.2287 | 0.808265 |
| gga-miR-135a-3-3p |  | 21.46 | 22.4 | 31 | 19.89 | 19.3 | 20.7 | 25.1067 | 19.96 | -0.331 | 0.809134 |
| gga-miR-1557 |  | 0 | 1.55 | 0.2 | 0 | 0 | 2.01 | 0.57 | 0.67 | 0.2332 | 0.809203 |
| gga-miR-19b-5p |  | 0.59 | 0.31 | 0.2 | 0.52 | 0.41 | 0.15 | 0.3533 | 0.36 | 0.0271 | 0.810346 |
| gga-miR-551-3p |  | 0.2 | 0.16 | 0.6 | 0.52 | 0.28 | 0.31 | 0.3333 | 0.37 | 0.1507 | 0.810885 |
| gga-miR-6552-3p |  | 1.97 | 3.11 | 3 | 2.09 | 2.48 | 2.78 | 2.7 | 2.45 | -0.1402 | 0.813502 |
| gga-miR-1739 |  | 0 | 0.16 | 0.8 | 0.13 | 0.41 | 0 | 0.32 | 0.18 | -0.8301 | 0.814003 |
| gga-miR-200b-5p |  | 59.06 | 104 | 108 | 66.1 | 68.7 | 79.9 | 90.4767 | 71.58 | -0.338 | 0.816034 |
| gga-miR-375 |  | 62.41 | 65.5 | 80 | 52.75 | 63.5 | 60.9 | 69.2633 | 59.0467 | -0.2302 | 0.818986 |
| gga-miR-6645-5p |  | 0 | 0.31 | 0.5 | 0.65 | 0.28 | 0 | 0.2633 | 0.31 | 0.2356 | 0.819346 |
| gga-miR-1684a-5p |  | 0.4 | 0 | 0.6 | 0 | 0.56 | 0 | 0.3467 | 0.1867 | -0.893 | 0.823965 |
| gga-miR-126-5p |  | 57.88 | 49.2 | 41 | 46.46 | 36.4 | 35.4 | 49.2233 | 39.4067 | -0.3209 | 0.827504 |
| gga-miR-6664-3p |  | 0.39 | 0.62 | 0.6 | 0.52 | 0.41 | 0.15 | 0.55 | 0.36 | -0.6114 | 0.829034 |
| gga-miR-6643-5p |  | 0 | 0.78 | 0 | 0 | 0 | 0.46 | 0.26 | 0.1533 | -0.7622 | 0.832468 |
| gga-miR-6660-3p |  | 2.95 | 2.95 | 2.5 | 1.31 | 2.62 | 3.71 | 2.8133 | 2.5467 | -0.1436 | 0.834842 |
| gga-miR-92-5p |  | 32.49 | 30.9 | 36 | 24.61 | 27.3 | 33.1 | 33.1667 | 28.32 | -0.2279 | 0.834853 |
| gga-miR-460b-3p |  | 0.2 | 0.62 | 0.2 | 0.79 | 0 | 0.31 | 0.3267 | 0.3667 | 0.1666 | 0.835906 |
| gga-miR-124c-3p |  | 0.79 | 0.47 | 0.5 | 0.13 | 0.28 | 0.77 | 0.58 | 0.3933 | -0.5604 | 0.837224 |
| gga-miR-6573-3p |  | 0.79 | 0 | 0.8 | 0.52 | 0.28 | 0.15 | 0.53 | 0.3167 | -0.7429 | 0.837246 |
| gga-miR-130c-5p |  | 0.2 | 0.47 | 1 | 0.52 | 0.41 | 0.15 | 0.54 | 0.36 | -0.585 | 0.837275 |
| gga-miR-7460-3p |  | 4.33 | 2.49 | 1.6 | 2.88 | 1.79 | 2.78 | 2.8033 | 2.4833 | -0.1749 | 0.838078 |
| gga-miR-1685-3p |  | 0 | 0.78 | 0.3 | 0.79 | 0 | 0.31 | 0.3667 | 0.3667 | 0 | 0.846538 |
| gga-miR-16-5p |  | 722.6 | 622 | 633 | 539.6 | 537 | 524 | 659.25 | 533.4933 | -0.3054 | 0.850017 |
| gga-miR-140-3p |  | 4752 | 4543 | ## | 3767 | 4123 | 3406 | 4648.98 | 3765.45 | -0.3041 | 0.853252 |
| gga-miR-6608-3p |  | 0.98 | 0.78 | 0.5 | 0.52 | 0.41 | 0.62 | 0.7467 | 0.5167 | -0.5312 | 0.853496 |
| gga-miR-6665-5p |  | 0.79 | 0.62 | 1 | 0.52 | 0.41 | 0.77 | 0.7867 | 0.5667 | -0.4732 | 0.858282 |
| gga-miR-125b-3p |  | 549.7 | 646 | 647 | 536 | 538 | 483 | 613.95 | 518.8367 | -0.2428 | 0.860452 |
| gga-miR-187-5p |  | 52.37 | 55.8 | 56 | 43.06 | 44.9 | 44.4 | 54.5467 | 44.1067 | -0.3065 | 0.864179 |
| gga-miR-1454 |  | 2.17 | 2.95 | 2.5 | 2.62 | 2.2 | 1.08 | 2.5533 | 1.9667 | -0.3766 | 0.866833 |
| gga-miR-3594-3p |  | 0.2 | 0.93 | 1.3 | 0.92 | 0.69 | 0.62 | 0.8 | 0.7433 | -0.1061 | 0.874027 |
| gga-miR-6582-5p |  | 0.39 | 1.4 | 0.8 | 1.05 | 0.55 | 0.15 | 0.8633 | 0.5833 | -0.5656 | 0.878557 |
| gga-miR-3530-3p |  | 2.36 | 6.37 | 4.1 | 4.45 | 3.86 | 2.94 | 4.29 | 3.75 | -0.1941 | 0.879001 |
| gga-miR-7455-3p |  | 0.98 | 1.09 | 1 | 0.39 | 0.96 | 0.93 | 1.0067 | 0.76 | -0.4056 | 0.880216 |
| gga-miR-7 |  | 754.7 | 538 | 485 | 395.3 | 490 | 557 | 592.373 | 480.7067 | -0.3013 | 0.886165 |
| gga-miR-10b-3p |  | 3.35 | 6.83 | 5.6 | 5.5 | 3.31 | 4.79 | 5.25 | 4.5333 | -0.2118 | 0.889555 |
| gga-miR-1684a-3p |  | 47.65 | 35.3 | 31 | 28.79 | 36.2 | 31.7 | 37.92 | 32.2333 | -0.2344 | 0.891409 |
| gga-miR-365-1-5p |  | 2.95 | 2.49 | 2.2 | 0.65 | 1.65 | 3.56 | 2.5567 | 1.9533 | -0.3884 | 0.891744 |
| gga-miR-490-3p |  | 1.38 | 1.09 | 1 | 0.65 | 1.1 | 1.39 | 1.14 | 1.0467 | -0.1232 | 0.893685 |
| gga-miR-30a-3p |  | 597.3 | 565 | 536 | 400.1 | 479 | 503 | 566.223 | 460.74 | -0.2974 | 0.894499 |
| gga-miR-34b-3p |  | 1.18 | 2.02 | 0.6 | 0.92 | 1.1 | 0.93 | 1.28 | 0.9833 | -0.3804 | 0.899968 |
| gga-miR-106-5p |  | 297.3 | 264 | 302 | 206.1 | 252 | 268 | 287.84 | 241.8733 | -0.251 | 0.906673 |
| gga-miR-7449-3p |  | 1.38 | 1.86 | 0.5 | 1.44 | 0.28 | 1.08 | 1.24 | 0.9333 | -0.4099 | 0.91127 |
| gga-let-7j-3p |  | 119.5 | 110 | 105 | 69.63 | 90.4 | 113 | 111.693 | 90.9367 | -0.2966 | 0.919538 |
| gga-let-7c-3p |  | 7.48 | 7.61 | 10 | 5.89 | 6.2 | 9.58 | 8.4767 | 7.2233 | -0.2308 | 0.920997 |
| gga-miR-1774 |  | 0.59 | 3.11 | 0.8 | 1.05 | 0.83 | 1.7 | 1.5 | 1.1933 | -0.33 | 0.926459 |
| gga-miR-3536 |  | 3.15 | 1.86 | 0.8 | 1.31 | 2.2 | 1.55 | 1.9367 | 1.6867 | -0.1994 | 0.930602 |
| gga-miR-99a-5p |  | 22685 | #### | ## | 20421 | ### | ### | 28556.7 | 23320.99 | -0.2922 | 0.949718 |
| gga-miR-146b-5p |  | 8253 | 6203 | ## | 4114 | 5523 | 6859 | 6685.52 | 5498.777 | -0.2819 | 0.958674 |
| gga-miR-26a-5p |  | 19447 | #### | ## | 14773 | ### | ### | 19354.4 | 15938.05 | -0.2802 | 0.971225 |
| gga-miR-455-5p |  | 468.8 | 525 | 528 | 357.8 | 417 | 475 | 507.21 | 416.87 | -0.283 | 0.971575 |
| gga-miR-1729-3p |  | 11.03 | 13.5 | 15 | 13.35 | 11 | 8.96 | 13.3233 | 11.11 | -0.2621 | 0.980135 |
| gga-miR-193a-5p |  | 331.9 | 470 | 428 | 305.4 | 315 | 397 | 409.76 | 339.0333 | -0.2734 | 0.981225 |
| gga-miR-17-5p |  | 502.6 | 479 | 563 | 431.9 | 436 | 408 | 514.987 | 425.0533 | -0.2769 | 0.987414 |
| gga-miR-103-3p |  | 4162 | 4336 | ## | 4099 | 3724 | 3212 | 4453.18 | 3678.347 | -0.2758 | 0.991226 |
| gga-miR-34a-5p |  | 73.63 | 74.2 | 78 | 73.56 | 62 | 51.6 | 75.2133 | 62.39 | -0.2697 | 0.993315 |
| gga-miR-1756b |  | 0 | 0 | 0 | 0 | 0.14 | 0 | 0 | 0.0467 | 2.2234 | 1 |
| gga-miR-1574-5p |  | 0 | 0 | 0 | 0.13 | 0 | 0 | 0 | 0.0433 | 2.1144 | 1 |
| gga-miR-135b |  | 0 | 0 | 0 | 0.13 | 0 | 0 | 0 | 0.0433 | 2.1144 | 1 |
| gga-miR-7442-3p |  | 0 | 0 | 0.2 | 0 | 0 | 0 | 0.0533 | 0 | -2.4141 | 1 |
| gga-miR-7478-5p |  | 0.2 | 0 | 0.3 | 0.13 | 0 | 0.15 | 0.1733 | 0.0933 | -0.8933 | 1 |
| gga-miR-1572 |  | 0 | 0.16 | 0 | 0 | 0 | 0 | 0.0533 | 0 | -2.4141 | 1 |
| gga-miR-137-3p |  | 0.2 | 0.31 | 0 | 0.13 | 0.14 | 0 | 0.17 | 0.09 | -0.9175 | 1 |
| gga-miR-6579-5p |  | 0 | 0 | 0.2 | 0 | 0 | 0 | 0.0533 | 0 | -2.4141 | 1 |
| gga-miR-6568-5p |  | 0 | 0 | 0 | 0 | 0.14 | 0 | 0 | 0.0467 | 2.2234 | 1 |
| gga-miR-1676-3p |  | 0.59 | 0 | 0.2 | 0.39 | 0 | 0.31 | 0.25 | 0.2333 | -0.0997 | 1 |
| gga-miR-1644 |  | 0 | 0 | 0 | 0.13 | 0 | 0 | 0 | 0.0433 | 2.1144 | 1 |
| gga-miR-1785 |  | 0 | 0 | 0 | 0.13 | 0 | 0 | 0 | 0.0433 | 2.1144 | 1 |
| gga-miR-7455-5p |  | 0 | 0 | 0 | 0.13 | 0 | 0 | 0 | 0.0433 | 2.1144 | 1 |
| gga-miR-7456-5p |  | 0 | 0 | 0 | 0 | 0 | 0.15 | 0 | 0.05 | 2.3219 | 1 |
| gga-miR-1562-3p |  | 0 | 0.16 | 0 | 0 | 0 | 0 | 0.0533 | 0 | -2.4141 | 1 |
| gga-miR-551-5p |  | 0 | 0 | 0 | 0 | 0.14 | 0 | 0 | 0.0467 | 2.2234 | 1 |
| gga-miR-1678 |  | 0 | 0.47 | 0 | 0.26 | 0 | 0 | 0.1567 | 0.0867 | -0.8539 | 1 |
| gga-miR-6699-5p |  | 0 | 0 | 0 | 0 | 0.14 | 0 | 0 | 0.0467 | 2.2234 | 1 |
| gga-miR-1646 |  | 0 | 0.16 | 0.2 | 0 | 0.28 | 0.15 | 0.1067 | 0.1433 | 0.4255 | 1 |
| gga-miR-6597-3p |  | 0 | 0 | 0.5 | 0.13 | 0.28 | 0.15 | 0.16 | 0.1867 | 0.2227 | 1 |
| gga-miR-1680-3p |  | 0.2 | 0 | 0.2 | 0 | 0 | 0.46 | 0.12 | 0.1533 | 0.3533 | 1 |
| gga-miR-1630 |  | 0 | 0 | 0 | 0 | 0.14 | 0 | 0 | 0.0467 | 2.2234 | 1 |
| gga-miR-1597-5p |  | 0 | 0 | 0 | 0 | 0.14 | 0 | 0 | 0.0467 | 2.2234 | 1 |
| gga-miR-1587 |  | 0 | 0 | 0.2 | 0 | 0 | 0 | 0.0533 | 0 | -2.4141 | 1 |
| gga-miR-1768 |  | 0 | 0 | 0 | 0 | 0 | 0.15 | 0 | 0.05 | 2.3219 | 1 |
| gga-miR-1639 |  | 0.39 | 0 | 0 | 0.13 | 0 | 0.31 | 0.13 | 0.1467 | 0.1744 | 1 |
| gga-miR-1762 |  | 0 | 0.16 | 0 | 0.13 | 0 | 0.15 | 0.0533 | 0.0933 | 0.8077 | 1 |
| gga-miR-6618-5p |  | 0 | 0.16 | 0 | 0 | 0 | 0 | 0.0533 | 0 | -2.4141 | 1 |
| gga-miR-6563-3p |  | 0 | 0 | 0 | 0 | 0 | 0.15 | 0 | 0.05 | 2.3219 | 1 |
| gga-miR-1710 |  | 0 | 0 | 0 | 0 | 0.14 | 0 | 0 | 0.0467 | 2.2234 | 1 |
| gga-miR-1689-3p |  | 0 | 0 | 0.2 | 0 | 0 | 0 | 0.0533 | 0 | -2.4141 | 1 |
| gga-miR-215-3p |  | 0.2 | 0 | 0 | 0 | 0 | 0 | 0.0667 | 0 | -2.7377 | 1 |
| gga-miR-1642 |  | 0 | 0 | 0 | 0.13 | 0 | 0 | 0 | 0.0433 | 2.1144 | 1 |
| gga-miR-6613-3p |  | 0 | 0 | 0.2 | 0 | 0 | 0 | 0.0533 | 0 | -2.4141 | 1 |
| gga-miR-1726 |  | 0 | 0 | 0 | 0 | 0 | 0.15 | 0 | 0.05 | 2.3219 | 1 |
| gga-miR-6627-3p |  | 0 | 0 | 0 | 0 | 0.14 | 0 | 0 | 0.0467 | 2.2234 | 1 |
| gga-miR-1588 |  | 0.2 | 0 | 0 | 0 | 0 | 0 | 0.0667 | 0 | -2.7377 | 1 |
| gga-miR-6574-5p |  | 0 | 0 | 0 | 0 | 0.14 | 0 | 0 | 0.0467 | 2.2234 | 1 |
| gga-miR-1699 |  | 0 | 0 | 0 | 0.13 | 0 | 0 | 0 | 0.0433 | 2.1144 | 1 |
| gga-miR-6693-3p |  | 0 | 0 | 0.2 | 0 | 0 | 0 | 0.0533 | 0 | -2.4141 | 1 |
| gga-miR-1614-5p |  | 0 | 0 | 0.2 | 0 | 0 | 0 | 0.0533 | 0 | -2.4141 | 1 |
| gga-miR-122b |  | 0 | 0 | 0 | 0.13 | 0 | 0 | 0 | 0.0433 | 2.1144 | 1 |
| gga-miR-6667-5p |  | 0 | 0 | 0 | 0 | 0.14 | 0 | 0 | 0.0467 | 2.2234 | 1 |
| gga-miR-7477-5p |  | 0 | 0.16 | 0 | 0 | 0 | 0 | 0.0533 | 0 | -2.4141 | 1 |
| gga-miR-6687-3p |  | 0 | 0 | 0.3 | 0 | 0 | 0.15 | 0.1067 | 0.05 | -1.0936 | 1 |
| gga-miR-6663-5p |  | 0 | 1.09 | 1.1 | 0.39 | 0.83 | 0.46 | 0.7333 | 0.56 | -0.389 | 1 |
| gga-miR-1748 |  | 0 | 0 | 0 | 0 | 0.14 | 0 | 0 | 0.0467 | 2.2234 | 1 |
| gga-miR-6639-5p |  | 0 | 0 | 0 | 0 | 0.14 | 0 | 0 | 0.0467 | 2.2234 | 1 |
| gga-miR-101-1-5p |  | 0.79 | 0 | 0.2 | 0.13 | 0.55 | 0 | 0.3167 | 0.2267 | -0.4823 | 1 |
| gga-miR-7438-5p |  | 0.79 | 0.62 | 0.5 | 0.26 | 0.83 | 0.31 | 0.63 | 0.4667 | -0.4329 | 1 |
| gga-miR-2130 |  | 0.2 | 1.09 | 1 | 0.39 | 0.55 | 0.77 | 0.7467 | 0.57 | -0.3896 | 1 |
| gga-miR-1673 |  | 0 | 0.16 | 0.3 | 0.13 | 0.14 | 0 | 0.16 | 0.09 | -0.8301 | 1 |
| gga-miR-1659 |  | 0.2 | 0 | 0 | 0 | 0 | 0 | 0.0667 | 0 | -2.7377 | 1 |
| gga-miR-1758 |  | 0 | 0.16 | 0 | 0.13 | 0 | 0.15 | 0.0533 | 0.0933 | 0.8077 | 1 |
| gga-miR-216c |  | 0 | 0 | 0 | 0.13 | 0 | 0 | 0 | 0.0433 | 2.1144 | 1 |
| gga-miR-6615-3p |  | 0.2 | 0.31 | 0.2 | 0.13 | 0.28 | 0.31 | 0.2233 | 0.24 | 0.1041 | 1 |
| gga-miR-1694 |  | 0 | 0.16 | 0 | 0 | 0 | 0 | 0.0533 | 0 | -2.4141 | 1 |
| gga-miR-6628-3p |  | 0 | 0 | 0 | 0.13 | 0 | 0 | 0 | 0.0433 | 2.1144 | 1 |
| gga-miR-3607-3p |  | 0.39 | 0.31 | 0 | 0.13 | 0.14 | 0.15 | 0.2333 | 0.14 | -0.7368 | 1 |
| gga-miR-1596-5p |  | 0 | 0.16 | 0 | 0.26 | 0 | 0 | 0.0533 | 0.0867 | 0.7019 | 1 |
| gga-miR-3534 |  | 3.54 | 2.02 | 3.7 | 2.36 | 2.48 | 2.94 | 3.0733 | 2.5933 | -0.245 | 1 |
| gga-miR-6605-5p |  | 0 | 0.31 | 0.2 | 0.13 | 0 | 0.15 | 0.1567 | 0.0933 | -0.7481 | 1 |
| gga-miR-6590-5p |  | 0 | 0 | 0 | 0 | 0 | 0.15 | 0 | 0.05 | 2.3219 | 1 |
| gga-miR-2129 |  | 4.33 | 5.13 | 3.3 | 3.4 | 3.31 | 4.02 | 4.2667 | 3.5767 | -0.2545 | 1 |
| gga-miR-1585 |  | 0 | 0 | 0 | 0 | 0.14 | 0 | 0 | 0.0467 | 2.2234 | 1 |
| gga-miR-6701-3p |  | 0.2 | 0 | 0 | 0 | 0.14 | 0.15 | 0.0667 | 0.0967 | 0.5358 | 1 |
| gga-miR-1647 |  | 0.2 | 0 | 0 | 0 | 0 | 0 | 0.0667 | 0 | -2.7377 | 1 |
| gga-miR-6697-5p |  | 0 | 0 | 0 | 0 | 0 | 0 | 0 | 0 | 0 | 1 |
| gga-miR-6578-5p |  | 0 | 0.31 | 0 | 0.13 | 0.28 | 0 | 0.1033 | 0.1367 | 0.4042 | 1 |
| gga-miR-7476-5p |  | 0 | 0 | 0 | 0 | 0.14 | 0 | 0 | 0.0467 | 2.2234 | 1 |
| gga-miR-1599 |  | 0 | 0 | 0.2 | 0.13 | 0 | 0 | 0.0533 | 0.0433 | -0.2998 | 1 |
| gga-miR-1617 |  | 0 | 0 | 0 | 0 | 0 | 0 | 0 | 0 | 0 | 1 |
| gga-miR-1765 |  | 0 | 0.16 | 0.2 | 0.26 | 0 | 0 | 0.1067 | 0.0867 | -0.2995 | 1 |
| gga-miR-133c-5p |  | 0 | 0 | 0 | 0 | 0 | 0 | 0 | 0 | 0 | 1 |
| gga-miR-6614-3p |  | 0 | 0 | 0 | 0 | 0 | 0 | 0 | 0 | 0 | 1 |
| gga-miR-6551-5p |  | 0 | 0 | 0 | 0 | 0 | 0 | 0 | 0 | 0 | 1 |
| gga-miR-6650-5p |  | 0 | 0 | 0 | 0 | 0 | 0 | 0 | 0 | 0 | 1 |
| gga-miR-1669 |  | 0 | 0.31 | 0 | 0.13 | 0 | 0.15 | 0.1033 | 0.0933 | -0.1469 | 1 |
| gga-miR-6644-3p |  | 0 | 0 | 0 | 0 | 0 | 0 | 0 | 0 | 0 | 1 |
| gga-miR-6671-5p |  | 0 | 0 | 0 | 0 | 0 | 0 | 0 | 0 | 0 | 1 |
| gga-miR-6704-5p |  | 0 | 0 | 0 | 0 | 0 | 0 | 0 | 0 | 0 | 1 |
| gga-miR-1814 |  | 0 | 0 | 0 | 0 | 0 | 0 | 0 | 0 | 0 | 1 |
| gga-miR-1660 |  | 0 | 0 | 0 | 0 | 0 | 0 | 0 | 0 | 0 | 1 |
| gga-miR-101-2-5p |  | 0 | 0.16 | 0 | 0 | 0.28 | 0 | 0.0533 | 0.0933 | 0.8077 | 1 |
| gga-miR-6549-3p |  | 0.2 | 0 | 0.5 | 0.13 | 0.14 | 0.15 | 0.2267 | 0.14 | -0.6954 | 1 |
| gga-miR-6576-5p |  | 0 | 0 | 0.2 | 0 | 0.14 | 0 | 0.0533 | 0.0467 | -0.1907 | 1 |
| gga-miR-1728-5p |  | 0.2 | 0.62 | 0.8 | 0.52 | 0.28 | 0.46 | 0.54 | 0.42 | -0.3626 | 1 |
| gga-miR-1790 |  | 0 | 0 | 0 | 0 | 0 | 0 | 0 | 0 | 0 | 1 |
| gga-miR-6670-5p |  | 0 | 0 | 0 | 0 | 0 | 0 | 0 | 0 | 0 | 1 |
| gga-miR-1733 |  | 0 | 0 | 0 | 0 | 0 | 0 | 0 | 0 | 0 | 1 |
| gga-miR-6569-5p |  | 0 | 0 | 0 | 0 | 0 | 0 | 0 | 0 | 0 | 1 |
| gga-miR-1700 |  | 0.59 | 0.16 | 1.1 | 0.13 | 0.69 | 0.62 | 0.62 | 0.48 | -0.3692 | 1 |
| gga-miR-7446-3p |  | 0 | 0 | 0 | 0 | 0 | 0 | 0 | 0 | 0 | 1 |
| gga-miR-7454-3p |  | 0 | 0 | 0 | 0 | 0 | 0 | 0 | 0 | 0 | 1 |
| gga-miR-2126 |  | 0 | 0 | 0 | 0 | 0 | 0 | 0 | 0 | 0 | 1 |
| gga-miR-1618-3p |  | 0 | 0.16 | 0.8 | 0.26 | 0.28 | 0.15 | 0.32 | 0.23 | -0.4764 | 1 |
| gga-miR-1466 |  | 0 | 0 | 0 | 0 | 0 | 0 | 0 | 0 | 0 | 1 |
| gga-miR-1a-2-5p |  | 0 | 0 | 0 | 0 | 0 | 0 | 0 | 0 | 0 | 1 |
| gga-miR-1554 |  | 0 | 0 | 0 | 0 | 0 | 0 | 0 | 0 | 0 | 1 |
| gga-miR-6595-5p |  | 0 | 0 | 0 | 0 | 0 | 0 | 0 | 0 | 0 | 1 |
| gga-miR-6674-3p |  | 0 | 0 | 0 | 0 | 0.14 | 0 | 0 | 0.0467 | 2.2234 | 1 |
| gga-miR-7441-3p |  | 0 | 0 | 0 | 0 | 0 | 0 | 0 | 0 | 0 | 1 |
| gga-miR-757 |  | 0 | 0 | 0 | 0 | 0 | 0 | 0 | 0 | 0 | 1 |
| gga-miR-1569 |  | 0.59 | 0.93 | 1.6 | 0.52 | 1.1 | 0.93 | 1.0367 | 0.85 | -0.2865 | 1 |
| gga-miR-1756a |  | 0 | 0 | 0 | 0.13 | 0 | 0 | 0 | 0.0433 | 2.1144 | 1 |
| gga-miR-6580-3p |  | 0 | 0 | 0 | 0 | 0 | 0 | 0 | 0 | 0 | 1 |
| gga-miR-6598-5p |  | 0 | 0 | 0 | 0 | 0 | 0 | 0 | 0 | 0 | 1 |
| gga-miR-1553-3p |  | 0.39 | 1.55 | 0.6 | 0.65 | 0.69 | 0.77 | 0.86 | 0.7033 | -0.2902 | 1 |
| gga-miR-1616 |  | 0 | 0 | 0 | 0 | 0 | 0 | 0 | 0 | 0 | 1 |
| gga-miR-1561 |  | 0 | 0 | 0 | 0 | 0.14 | 0 | 0 | 0.0467 | 2.2234 | 1 |
| gga-miR-1772-3p |  | 0 | 0.16 | 0.3 | 0 | 0.14 | 0.31 | 0.16 | 0.15 | -0.0931 | 1 |
| gga-miR-7445-5p |  | 0 | 0 | 0 | 0 | 0 | 0 | 0 | 0 | 0 | 1 |
| gga-miR-7459-5p |  | 0 | 0 | 0 | 0 | 0 | 0 | 0 | 0 | 0 | 1 |
| gga-miR-1619 |  | 0.2 | 0 | 0 | 0 | 0.14 | 0 | 0.0667 | 0.0467 | -0.5143 | 1 |
| gga-miR-302c-3p |  | 0 | 0 | 0 | 0.13 | 0 | 0 | 0 | 0.0433 | 2.1144 | 1 |
| gga-miR-6571-3p |  | 0 | 0 | 0 | 0 | 0 | 0 | 0 | 0 | 0 | 1 |
| gga-miR-6560-5p |  | 0 | 0 | 0 | 0.13 | 0 | 0 | 0 | 0.0433 | 2.1144 | 1 |
| gga-miR-7480-5p |  | 0 | 0 | 0 | 0 | 0 | 0 | 0 | 0 | 0 | 1 |
| gga-miR-1696 |  | 0 | 0 | 0 | 0 | 0 | 0 | 0 | 0 | 0 | 1 |
| gga-miR-6583-5p |  | 0 | 0 | 0 | 0 | 0 | 0 | 0 | 0 | 0 | 1 |
| gga-miR-1734 |  | 0 | 0 | 0 | 0 | 0 | 0 | 0 | 0 | 0 | 1 |
| gga-miR-6587-3p |  | 0 | 0 | 0 | 0 | 0 | 0 | 0 | 0 | 0 | 1 |
| gga-miR-6560-3p |  | 0 | 0 | 0 | 0 | 0 | 0 | 0 | 0 | 0 | 1 |
| gga-miR-1703-3p |  | 0 | 0 | 0 | 0 | 0 | 0 | 0 | 0 | 0 | 1 |
| gga-miR-6579-3p |  | 0 | 0 | 0 | 0 | 0 | 0 | 0 | 0 | 0 | 1 |
| gga-miR-1725 |  | 0 | 0 | 0 | 0 | 0 | 0 | 0 | 0 | 0 | 1 |
| gga-miR-1579 |  | 0 | 0 | 0 | 0 | 0 | 0 | 0 | 0 | 0 | 1 |
| gga-miR-1697 |  | 0.39 | 0 | 0.3 | 0.26 | 0.28 | 0.15 | 0.2367 | 0.23 | -0.0414 | 1 |
| gga-miR-1462-3p |  | 0 | 0 | 0 | 0 | 0 | 0 | 0 | 0 | 0 | 1 |
| gga-miR-1688 |  | 0 | 0 | 0 | 0 | 0 | 0 | 0 | 0 | 0 | 1 |
| gga-miR-1719 |  | 0 | 0 | 0 | 0 | 0 | 0 | 0 | 0 | 0 | 1 |
| gga-miR-6577-3p |  | 0 | 0 | 0 | 0 | 0 | 0 | 0 | 0 | 0 | 1 |
| gga-miR-1622 |  | 0 | 0 | 0 | 0 | 0 | 0 | 0 | 0 | 0 | 1 |
| gga-miR-1463 |  | 0 | 0 | 0 | 0 | 0 | 0 | 0 | 0 | 0 | 1 |
| gga-miR-6707-5p |  | 0 | 0 | 0 | 0.13 | 0 | 0 | 0 | 0.0433 | 2.1144 | 1 |
| gga-miR-1718 |  | 0.2 | 0.16 | 0 | 0 | 0 | 0.15 | 0.12 | 0.05 | -1.263 | 1 |
| gga-miR-7444-3p |  | 0 | 0 | 0 | 0 | 0 | 0 | 0 | 0 | 0 | 1 |
| gga-miR-7448-3p |  | 0 | 0 | 0 | 0 | 0 | 0 | 0 | 0 | 0 | 1 |
| gga-miR-6642-5p |  | 0 | 0 | 0 | 0 | 0 | 0 | 0 | 0 | 0 | 1 |
| gga-miR-460a-3p |  | 0.2 | 0.16 | 0 | 0 | 0.14 | 0 | 0.12 | 0.0467 | -1.3615 | 1 |
| gga-miR-6584-5p |  | 0.2 | 0 | 0 | 0 | 0 | 0.15 | 0.0667 | 0.05 | -0.4158 | 1 |
| gga-miR-6648-5p |  | 0 | 0 | 0 | 0 | 0 | 0 | 0 | 0 | 0 | 1 |
| gga-miR-196-1-3p |  | 0 | 0 | 0 | 0 | 0 | 0 | 0 | 0 | 0 | 1 |
| gga-miR-1658-5p |  | 0 | 0 | 0 | 0 | 0 | 0 | 0 | 0 | 0 | 1 |
| gga-miR-6578-3p |  | 0 | 0 | 0 | 0 | 0 | 0 | 0 | 0 | 0 | 1 |
| gga-miR-6714-3p |  | 0 | 0 | 0 | 0 | 0 | 0 | 0 | 0 | 0 | 1 |
| gga-miR-124c-5p |  | 0 | 0 | 0 | 0 | 0 | 0 | 0 | 0 | 0 | 1 |
| gga-miR-1624 |  | 0 | 0 | 0 | 0 | 0 | 0 | 0 | 0 | 0 | 1 |
| gga-miR-1671 |  | 0 | 0 | 0 | 0 | 0 | 0 | 0 | 0 | 0 | 1 |
| gga-miR-7459-3p |  | 0 | 0 | 0 | 0 | 0 | 0 | 0 | 0 | 0 | 1 |
| gga-miR-6569-3p |  | 0 | 0 | 0 | 0 | 0 | 0 | 0 | 0 | 0 | 1 |
| gga-miR-7461-5p |  | 0 | 0 | 0 | 0 | 0 | 0 | 0 | 0 | 0 | 1 |
| gga-miR-103-1-5p |  | 0 | 0 | 0 | 0 | 0 | 0 | 0 | 0 | 0 | 1 |
| gga-miR-7471-3p |  | 0 | 0 | 0 | 0 | 0 | 0 | 0 | 0 | 0 | 1 |
| gga-miR-1793 |  | 0 | 0 | 0 | 0 | 0 | 0.15 | 0 | 0.05 | 2.3219 | 1 |
| gga-miR-6686-3p |  | 0 | 0 | 0 | 0 | 0 | 0 | 0 | 0 | 0 | 1 |
| gga-miR-1458 |  | 0 | 0 | 0 | 0 | 0 | 0 | 0 | 0 | 0 | 1 |
| gga-miR-1808 |  | 0 | 0 | 0.3 | 0.13 | 0.28 | 0 | 0.1067 | 0.1367 | 0.3575 | 1 |
| gga-miR-6561-3p |  | 0 | 0 | 0 | 0 | 0 | 0 | 0 | 0 | 0 | 1 |
| gga-miR-1670 |  | 0 | 0 | 0 | 0 | 0 | 0 | 0 | 0 | 0 | 1 |
| gga-miR-6580-5p |  | 0 | 0 | 0 | 0 | 0 | 0 | 0 | 0 | 0 | 1 |
| gga-miR-6706-5p |  | 0 | 0 | 0 | 0 | 0 | 0 | 0 | 0 | 0 | 1 |
| gga-miR-1562-5p |  | 0 | 0 | 0 | 0 | 0 | 0 | 0 | 0 | 0 | 1 |
| gga-miR-7439-5p |  | 0 | 0 | 0 | 0 | 0 | 0 | 0 | 0 | 0 | 1 |
| gga-miR-6629-5p |  | 0 | 0 | 0.2 | 0 | 0 | 0 | 0.0533 | 0 | -2.4141 | 1 |
| gga-miR-6607-5p |  | 0 | 0 | 0 | 0 | 0 | 0 | 0 | 0 | 0 | 1 |
| gga-miR-6702-5p |  | 0 | 0 | 0.2 | 0 | 0 | 0.31 | 0.0533 | 0.1033 | 0.9546 | 1 |
| gga-miR-1799 |  | 0 | 0 | 0 | 0 | 0 | 0 | 0 | 0 | 0 | 1 |
| gga-miR-1845 |  | 0 | 0 | 0 | 0 | 0 | 0 | 0 | 0 | 0 | 1 |
| gga-miR-1615 |  | 0 | 0 | 0 | 0 | 0 | 0 | 0 | 0 | 0 | 1 |
| gga-miR-1740-3p |  | 0 | 0 | 0 | 0 | 0 | 0 | 0 | 0 | 0 | 1 |
| gga-miR-6593-3p |  | 0 | 0 | 0.2 | 0 | 0 | 0 | 0.0533 | 0 | -2.4141 | 1 |
| gga-miR-1576 |  | 0 | 0 | 0 | 0 | 0 | 0 | 0 | 0 | 0 | 1 |
| gga-miR-7463-3p |  | 0 | 0 | 0 | 0 | 0 | 0 | 0 | 0 | 0 | 1 |
| gga-miR-124b |  | 0 | 0 | 0 | 0 | 0 | 0 | 0 | 0 | 0 | 1 |
| gga-miR-1715-3p |  | 0 | 0 | 0 | 0 | 0 | 0 | 0 | 0 | 0 | 1 |
| gga-miR-6553-5p |  | 0 | 0 | 0 | 0 | 0 | 0 | 0 | 0 | 0 | 1 |
| gga-miR-6700-3p |  | 0 | 0 | 0 | 0 | 0 | 0 | 0 | 0 | 0 | 1 |
| gga-miR-6562-5p |  | 0 | 0 | 0 | 0 | 0 | 0 | 0 | 0 | 0 | 1 |
| gga-miR-1661 |  | 0 | 0.31 | 0.2 | 0 | 0.28 | 0 | 0.1567 | 0.0933 | -0.7481 | 1 |
| gga-miR-6602-5p |  | 0 | 0 | 0.2 | 0 | 0 | 0.15 | 0.0533 | 0.05 | -0.0922 | 1 |
| gga-miR-1766 |  | 0 | 0 | 0 | 0 | 0 | 0 | 0 | 0 | 0 | 1 |
| gga-miR-6624-3p |  | 0 | 0 | 0 | 0 | 0 | 0 | 0 | 0 | 0 | 1 |
| gga-miR-1601 |  | 0 | 0 | 0 | 0 | 0 | 0.15 | 0 | 0.05 | 2.3219 | 1 |
| gga-miR-302c-5p |  | 0 | 0 | 0 | 0 | 0 | 0 | 0 | 0 | 0 | 1 |
| gga-miR-7474-3p |  | 0 | 0 | 0 | 0 | 0 | 0 | 0 | 0 | 0 | 1 |
| gga-miR-7481-5p |  | 0 | 0 | 0 | 0 | 0 | 0 | 0 | 0 | 0 | 1 |
| gga-miR-1759-3p |  | 0 | 0 | 0 | 0 | 0 | 0 | 0 | 0 | 0 | 1 |
| gga-miR-1730-5p |  | 0 | 0 | 0 | 0 | 0 | 0 | 0 | 0 | 0 | 1 |
| gga-miR-196-2-3p |  | 0 | 0 | 0 | 0 | 0 | 0 | 0 | 0 | 0 | 1 |
| gga-miR-7480-3p |  | 0 | 0 | 0 | 0 | 0 | 0 | 0 | 0 | 0 | 1 |
| gga-miR-1c |  | 0.2 | 0 | 0 | 0 | 0 | 0.31 | 0.0667 | 0.1033 | 0.6311 | 1 |
| gga-miR-1667-3p |  | 0 | 0 | 0.2 | 0 | 0 | 0 | 0.0533 | 0 | -2.4141 | 1 |
| gga-miR-1803 |  | 0 | 1.24 | 0 | 0.26 | 0.14 | 0.46 | 0.4133 | 0.2867 | -0.5276 | 1 |
| gga-miR-7472-3p |  | 0 | 0 | 0 | 0 | 0 | 0 | 0 | 0 | 0 | 1 |
| gga-miR-458b-3p |  | 0 | 0 | 0 | 0 | 0 | 0 | 0 | 0 | 0 | 1 |
| gga-miR-449b-3p |  | 0 | 0 | 0 | 0 | 0 | 0 | 0 | 0 | 0 | 1 |
| gga-miR-1571 |  | 0 | 0 | 0 | 0 | 0 | 0 | 0 | 0 | 0 | 1 |
| gga-miR-1575 |  | 0 | 0 | 0 | 0 | 0 | 0 | 0 | 0 | 0 | 1 |
| gga-miR-6712-5p |  | 0 | 0 | 0 | 0 | 0 | 0 | 0 | 0 | 0 | 1 |
| gga-miR-1732 |  | 0 | 0 | 0 | 0 | 0 | 0 | 0 | 0 | 0 | 1 |
| gga-miR-1757 |  | 0 | 0 | 0 | 0 | 0 | 0 | 0 | 0 | 0 | 1 |
| gga-miR-2188-3p |  | 0 | 0 | 0 | 0 | 0 | 0 | 0 | 0 | 0 | 1 |
| gga-miR-6622-3p |  | 0 | 0.16 | 0 | 0 | 0 | 0 | 0.0533 | 0 | -2.4141 | 1 |
| gga-miR-6653-3p |  | 0 | 0 | 0 | 0 | 0 | 0 | 0 | 0 | 0 | 1 |
| gga-miR-367 |  | 0 | 0 | 0 | 0 | 0 | 0.15 | 0 | 0.05 | 2.3219 | 1 |
| gga-miR-7451-3p |  | 0 | 0 | 0 | 0.13 | 0 | 0 | 0 | 0.0433 | 2.1144 | 1 |
| gga-miR-762 |  | 0.2 | 0.16 | 0 | 0 | 0.14 | 0 | 0.12 | 0.0467 | -1.3615 | 1 |
| gga-miR-458b-5p |  | 0 | 0 | 0 | 0 | 0 | 0 | 0 | 0 | 0 | 1 |
| gga-miR-302b-3p |  | 0.2 | 0 | 0.8 | 0.26 | 0.14 | 0.46 | 0.3333 | 0.2867 | -0.2173 | 1 |
| gga-miR-1456-3p |  | 1.18 | 0.47 | 0.6 | 0.13 | 0.83 | 0.93 | 0.7633 | 0.63 | -0.2769 | 1 |
| gga-miR-1770 |  | 0 | 0.16 | 0.2 | 0 | 0 | 0.31 | 0.1067 | 0.1033 | -0.0467 | 1 |
| gga-miR-1667-5p |  | 0 | 0 | 0 | 0 | 0 | 0 | 0 | 0 | 0 | 1 |
| gga-miR-1643-3p |  | 0 | 0 | 0 | 0 | 0.14 | 0 | 0 | 0.0467 | 2.2234 | 1 |
| gga-miR-7482-3p |  | 0 | 0 | 0 | 0 | 0 | 0 | 0 | 0 | 0 | 1 |
| gga-miR-1812-3p |  | 0 | 0 | 0 | 0 | 0 | 0 | 0 | 0 | 0 | 1 |
| gga-miR-1626-5p |  | 1.18 | 0.31 | 0.8 | 0.92 | 0.41 | 0.62 | 0.7633 | 0.65 | -0.2318 | 1 |
| gga-miR-6711-5p |  | 0 | 0 | 0.2 | 0 | 0.14 | 0 | 0.0533 | 0.0467 | -0.1907 | 1 |
| gga-miR-1795 |  | 0 | 0 | 0.3 | 0.13 | 0 | 0 | 0.1067 | 0.0433 | -1.3011 | 1 |
| gga-miR-7467-5p |  | 0 | 0 | 0 | 0 | 0.14 | 0 | 0 | 0.0467 | 2.2234 | 1 |
| gga-miR-6676-3p |  | 0 | 0 | 0 | 0 | 0 | 0 | 0 | 0 | 0 | 1 |
| gga-miR-1459 |  | 0 | 0 | 0 | 0 | 0 | 0 | 0 | 0 | 0 | 1 |
| gga-miR-3533 |  | 0 | 0.31 | 0.2 | 0 | 0.41 | 0 | 0.1567 | 0.1367 | -0.197 | 1 |
| gga-miR-6690-5p |  | 0.2 | 0 | 0.2 | 0 | 0.14 | 0 | 0.12 | 0.0467 | -1.3615 | 1 |
| gga-miR-6585-5p |  | 0 | 0 | 0 | 0 | 0 | 0 | 0 | 0 | 0 | 1 |
| gga-miR-1565 |  | 0 | 0 | 0.2 | 0 | 0.14 | 0 | 0.0533 | 0.0467 | -0.1907 | 1 |
| gga-miR-6692-5p |  | 0 | 0 | 0 | 0 | 0 | 0 | 0 | 0 | 0 | 1 |
| gga-miR-7439-3p |  | 0 | 0 | 0 | 0 | 0 | 0 | 0 | 0 | 0 | 1 |
| gga-miR-7437-3p |  | 0 | 0 | 0 | 0 | 0 | 0 | 0 | 0 | 0 | 1 |
| gga-miR-1788-3p |  | 0 | 0.62 | 0.2 | 0.26 | 0.14 | 0.31 | 0.26 | 0.2367 | -0.1355 | 1 |
| gga-miR-1633 |  | 0 | 0 | 0 | 0 | 0 | 0 | 0 | 0 | 0 | 1 |
| gga-miR-1760 |  | 0 | 0 | 0 | 0 | 0 | 0 | 0 | 0 | 0 | 1 |
| gga-miR-6640-5p |  | 0 | 0.16 | 0 | 0 | 0.14 | 0 | 0.0533 | 0.0467 | -0.1907 | 1 |
| gga-miR-1629 |  | 0 | 0 | 0 | 0 | 0 | 0 | 0 | 0 | 0 | 1 |
| gga-miR-1811 |  | 0 | 0 | 0 | 0 | 0 | 0 | 0 | 0 | 0 | 1 |
| gga-miR-7473-3p |  | 0 | 0 | 0 | 0 | 0 | 0 | 0 | 0 | 0 | 1 |
| gga-miR-6616-5p |  | 0 | 0 | 0 | 0 | 0 | 0 | 0 | 0 | 0 | 1 |
| gga-miR-1664-5p |  | 0 | 0 | 0 | 0 | 0 | 0 | 0 | 0 | 0 | 1 |
| gga-miR-458a-5p |  | 0 | 0 | 0 | 0 | 0 | 0 | 0 | 0 | 0 | 1 |
| gga-miR-1705 |  | 0 | 0 | 0 | 0 | 0 | 0 | 0 | 0 | 0 | 1 |
| gga-miR-6566-3p |  | 0.2 | 0 | 0 | 0 | 0 | 0.15 | 0.0667 | 0.05 | -0.4158 | 1 |
| gga-miR-1397-3p |  | 0 | 0 | 0 | 0 | 0 | 0 | 0 | 0 | 0 | 1 |
| gga-miR-7450-5p |  | 0 | 0 | 0 | 0 | 0 | 0 | 0 | 0 | 0 | 1 |
| gga-miR-1620 |  | 0 | 0 | 0 | 0 | 0 | 0 | 0 | 0 | 0 | 1 |
| gga-miR-3607-5p |  | 1.58 | 1.4 | 1.1 | 1.18 | 1.24 | 0.93 | 1.3633 | 1.1167 | -0.2879 | 1 |
| gga-miR-6632-5p |  | 0 | 0 | 0 | 0 | 0 | 0 | 0 | 0 | 0 | 1 |
| gga-miR-6556-3p |  | 0 | 0 | 0 | 0 | 0 | 0 | 0 | 0 | 0 | 1 |
| gga-miR-7440-3p |  | 0 | 0 | 0 | 0 | 0 | 0 | 0 | 0 | 0 | 1 |
| gga-miR-6637-3p |  | 0 | 0 | 0 | 0 | 0 | 0 | 0 | 0 | 0 | 1 |
| gga-miR-6575-3p |  | 0 | 0 | 0 | 0 | 0 | 0 | 0 | 0 | 0 | 1 |
| gga-miR-1752 |  | 0 | 0 | 0 | 0 | 0 | 0 | 0 | 0 | 0 | 1 |
| gga-miR-6565-5p |  | 0 | 0 | 0 | 0 | 0 | 0 | 0 | 0 | 0 | 1 |
| gga-miR-1806 |  | 0 | 0 | 0 | 0 | 0 | 0 | 0 | 0 | 0 | 1 |
| gga-miR-6621-5p |  | 0 | 0 | 0 | 0 | 0 | 0 | 0 | 0 | 0 | 1 |
| gga-miR-7450-3p |  | 0 | 0 | 0 | 0 | 0 | 0 | 0 | 0 | 0 | 1 |
| gga-miR-1584 |  | 0 | 0 | 0 | 0 | 0 | 0 | 0 | 0 | 0 | 1 |
| gga-miR-7446-5p |  | 0 | 0 | 0 | 0 | 0 | 0 | 0 | 0 | 0 | 1 |
| gga-miR-6700-5p |  | 0 | 0 | 0 | 0 | 0 | 0 | 0 | 0 | 0 | 1 |
| gga-miR-1638 |  | 0 | 0 | 0 | 0 | 0 | 0 | 0 | 0 | 0 | 1 |
| gga-miR-7453-5p |  | 0 | 0 | 0 | 0 | 0 | 0 | 0 | 0 | 0 | 1 |
| gga-miR-1773-5p |  | 0 | 0 | 0 | 0 | 0 | 0 | 0 | 0 | 0 | 1 |
| gga-miR-6620-5p |  | 0 | 0 | 0 | 0 | 0 | 0 | 0 | 0 | 0 | 1 |
| gga-miR-1651-5p |  | 0 | 0 | 0 | 0 | 0 | 0 | 0 | 0 | 0 | 1 |
| gga-miR-7467-3p |  | 0 | 0 | 0 | 0 | 0 | 0 | 0 | 0 | 0 | 1 |
| gga-miR-1749-5p |  | 0 | 0 | 0 | 0 | 0 | 0 | 0 | 0 | 0 | 1 |
| gga-miR-1715-5p |  | 0 | 0 | 0.3 | 0.13 | 0 | 0.15 | 0.1067 | 0.0933 | -0.1936 | 1 |
| gga-miR-6542-5p |  | 0 | 0 | 0 | 0 | 0 | 0 | 0 | 0 | 0 | 1 |
| gga-miR-1815 |  | 0 | 0 | 0 | 0 | 0 | 0 | 0 | 0 | 0 | 1 |
| gga-miR-1636 |  | 0 | 0 | 0.3 | 0.13 | 0 | 0.31 | 0.1067 | 0.1467 | 0.4593 | 1 |
| gga-miR-1746 |  | 0 | 0 | 0 | 0 | 0 | 0 | 0 | 0 | 0 | 1 |
| gga-miR-1598 |  | 0 | 0 | 0 | 0 | 0.14 | 0 | 0 | 0.0467 | 2.2234 | 1 |
| gga-miR-1754-3p |  | 0 | 0 | 0 | 0 | 0 | 0 | 0 | 0 | 0 | 1 |
| gga-miR-7483-3p |  | 0 | 0 | 0 | 0 | 0 | 0 | 0 | 0 | 0 | 1 |
| gga-miR-1634 |  | 0 | 0 | 0 | 0 | 0 | 0 | 0 | 0 | 0 | 1 |
| gga-miR-6689-3p |  | 0 | 0.16 | 0 | 0.26 | 0 | 0 | 0.0533 | 0.0867 | 0.7019 | 1 |
| gga-miR-1693 |  | 0 | 0 | 0 | 0 | 0 | 0 | 0 | 0 | 0 | 1 |
| gga-miR-7479-3p |  | 0 | 0 | 0 | 0 | 0 | 0 | 0 | 0 | 0 | 1 |
| gga-miR-1675 |  | 0 | 0 | 0 | 0 | 0 | 0 | 0 | 0 | 0 | 1 |
| gga-miR-184-5p |  | 0 | 0 | 0 | 0.13 | 0 | 0 | 0 | 0.0433 | 2.1144 | 1 |
| gga-miR-3528 |  | 0 | 0.16 | 0 | 0 | 0 | 0 | 0.0533 | 0 | -2.4141 | 1 |
| gga-miR-7470-5p |  | 0 | 0 | 0 | 0 | 0 | 0 | 0 | 0 | 0 | 1 |
| gga-miR-7454-5p |  | 0 | 0 | 0 | 0 | 0 | 0 | 0 | 0 | 0 | 1 |
| gga-miR-1791-5p |  | 0 | 0 | 0 | 0 | 0 | 0 | 0 | 0 | 0 | 1 |
| gga-miR-1787 |  | 0.2 | 0 | 0 | 0 | 0 | 0.15 | 0.0667 | 0.05 | -0.4158 | 1 |
| gga-miR-1625-3p |  | 0 | 0 | 0 | 0 | 0 | 0 | 0 | 0 | 0 | 1 |
| gga-miR-6678-3p |  | 0 | 0 | 0 | 0 | 0 | 0 | 0 | 0 | 0 | 1 |
| gga-miR-1595-5p |  | 0 | 0 | 0 | 0 | 0 | 0 | 0 | 0 | 0 | 1 |
| gga-miR-7482-5p |  | 0 | 0 | 0 | 0 | 0 | 0 | 0 | 0 | 0 | 1 |
| gga-miR-1567 |  | 0 | 0 | 0 | 0 | 0 | 0 | 0 | 0 | 0 | 1 |
| gga-miR-7464-5p |  | 0 | 0 | 0 | 0 | 0 | 0 | 0 | 0 | 0 | 1 |
| gga-miR-7458-5p |  | 0 | 0 | 0 | 0 | 0 | 0 | 0 | 0 | 0 | 1 |
| gga-miR-6630-3p |  | 0 | 0 | 0 | 0 | 0 | 0 | 0 | 0 | 0 | 1 |
| gga-miR-1592 |  | 0 | 0 | 0 | 0 | 0.14 | 0 | 0 | 0.0467 | 2.2234 | 1 |
| gga-miR-1a-1-5p |  | 0 | 0 | 0 | 0 | 0 | 0 | 0 | 0 | 0 | 1 |
| gga-miR-1596-3p |  | 0.2 | 0 | 0 | 0 | 0.28 | 0 | 0.0667 | 0.0933 | 0.4842 | 1 |
| gga-miR-1594 |  | 0 | 0 | 0 | 0 | 0 | 0 | 0 | 0 | 0 | 1 |
| gga-miR-7469-3p |  | 0 | 0 | 0 | 0 | 0 | 0 | 0 | 0 | 0 | 1 |
| gga-miR-6600-3p |  | 0 | 0 | 0 | 0 | 0.14 | 0 | 0 | 0.0467 | 2.2234 | 1 |
| gga-miR-6698-3p |  | 0.79 | 0.93 | 0.8 | 0.26 | 0.28 | 1.55 | 0.84 | 0.6967 | -0.2699 | 1 |
| gga-miR-6680-3p |  | 0 | 0 | 0 | 0 | 0 | 0 | 0 | 0 | 0 | 1 |
| gga-miR-7457-3p |  | 0 | 0 | 0 | 0 | 0 | 0 | 0 | 0 | 0 | 1 |
| gga-miR-1686 |  | 0 | 0 | 0 | 0 | 0 | 0 | 0 | 0 | 0 | 1 |
| gga-miR-6642-3p |  | 0 | 0 | 0 | 0 | 0 | 0 | 0 | 0 | 0 | 1 |
| gga-miR-6691-5p |  | 0 | 0 | 0 | 0 | 0 | 0 | 0 | 0 | 0 | 1 |
| gga-miR-1691 |  | 0 | 0.31 | 0.5 | 0.39 | 0.28 | 0.15 | 0.2633 | 0.2733 | 0.0538 | 1 |
| gga-miR-7444-5p |  | 0 | 0 | 0 | 0 | 0 | 0 | 0 | 0 | 0 | 1 |
| gga-miR-124a-3p |  | 0 | 0.16 | 1 | 0.26 | 0.41 | 0 | 0.37 | 0.2233 | -0.7285 | 1 |
| gga-miR-6695-5p |  | 0 | 0 | 0 | 0 | 0 | 0 | 0 | 0 | 0 | 1 |
| gga-miR-1750 |  | 0 | 0 | 0 | 0 | 0 | 0 | 0 | 0 | 0 | 1 |
| gga-miR-1558 |  | 0 | 0 | 0 | 0 | 0 | 0 | 0 | 0 | 0 | 1 |
| gga-miR-7448-5p |  | 0 | 0 | 0 | 0 | 0 | 0 | 0 | 0 | 0 | 1 |
| gga-miR-3526 |  | 0 | 0 | 0 | 0 | 0 | 0 | 0 | 0 | 0 | 1 |
| gga-miR-6619-5p |  | 0 | 0 | 0 | 0 | 0 | 0 | 0 | 0 | 0 | 1 |
| gga-miR-7478-3p |  | 0 | 0 | 0 | 0 | 0 | 0 | 0 | 0 | 0 | 1 |
| gga-miR-302a |  | 0 | 0 | 0.2 | 0 | 0 | 0.15 | 0.0533 | 0.05 | -0.0922 | 1 |
| gga-miR-1653 |  | 4.73 | 5.44 | 5.9 | 5.24 | 4.41 | 3.86 | 5.35 | 4.5033 | -0.2486 | 1 |
| gga-miR-1658-3p |  | 0 | 0 | 0 | 0 | 0 | 0 | 0 | 0 | 0 | 1 |
| gga-miR-1637 |  | 0 | 0 | 0 | 0 | 0 | 0 | 0 | 0 | 0 | 1 |
| gga-miR-454-5p |  | 0 | 0 | 0 | 0 | 0 | 0 | 0 | 0 | 0 | 1 |
| gga-miR-7465-5p |  | 0 | 0 | 0 | 0 | 0 | 0 | 0 | 0 | 0 | 1 |
| gga-miR-7453-3p |  | 0 | 0 | 0 | 0 | 0 | 0 | 0 | 0 | 0 | 1 |
| gga-miR-1702 |  | 0 | 0 | 0 | 0 | 0 | 0 | 0 | 0 | 0 | 1 |
| gga-miR-6591-3p |  | 0 | 0 | 0 | 0 | 0 | 0 | 0 | 0 | 0 | 1 |
| gga-miR-7464-3p |  | 0 | 0 | 0 | 0 | 0 | 0 | 0 | 0 | 0 | 1 |
| gga-miR-1687-5p |  | 0 | 0 | 0 | 0.13 | 0 | 0 | 0 | 0.0433 | 2.1144 | 1 |
| gga-miR-466 |  | 0 | 0 | 0 | 0 | 0 | 0 | 0 | 0 | 0 | 1 |
| gga-miR-7468-5p |  | 0 | 0 | 0 | 0 | 0 | 0 | 0 | 0 | 0 | 1 |
| gga-miR-6550-5p |  | 0 | 0 | 0 | 0 | 0 | 0 | 0 | 0 | 0 | 1 |
| gga-miR-6603-3p |  | 0 | 0 | 0 | 0 | 0 | 0 | 0 | 0 | 0 | 1 |
| gga-miR-1632-5p |  | 0 | 0 | 0 | 0 | 0 | 0 | 0 | 0 | 0 | 1 |
| gga-miR-6545-3p |  | 0 | 0 | 0 | 0 | 0 | 0 | 0 | 0 | 0 | 1 |
| gga-miR-7452-3p |  | 0 | 0 | 0 | 0 | 0 | 0 | 0 | 0 | 0 | 1 |
| gga-miR-7451-5p |  | 0 | 0 | 0 | 0 | 0 | 0 | 0 | 0 | 0 | 1 |
| gga-miR-6558-5p |  | 0 | 0 | 0 | 0 | 0 | 0 | 0 | 0 | 0 | 1 |
| gga-miR-6567-3p |  | 0 | 0 | 0 | 0 | 0 | 0 | 0 | 0 | 0 | 1 |
| gga-miR-1656 |  | 0.2 | 0.16 | 0 | 0 | 0.14 | 0 | 0.12 | 0.0467 | -1.3615 | 1 |
| gga-miR-1749-3p |  | 0 | 0 | 0 | 0 | 0 | 0 | 0 | 0 | 0 | 1 |
| gga-miR-1712-5p |  | 0.2 | 0.47 | 0.3 | 0.52 | 0 | 0.15 | 0.33 | 0.2233 | -0.5635 | 1 |
| gga-miR-6703-3p |  | 0 | 0 | 0 | 0 | 0 | 0 | 0 | 0 | 0 | 1 |
| gga-miR-6587-5p |  | 0 | 0 | 0 | 0 | 0 | 0 | 0 | 0 | 0 | 1 |
| gga-miR-29a-5p |  | 2.56 | 1.4 | 1.1 | 2.75 | 0.83 | 0.62 | 1.69 | 1.4 | -0.2716 | 1 |
| gga-miR-1681 |  | 0 | 0 | 0 | 0 | 0 | 0 | 0 | 0 | 0 | 1 |
| gga-miR-124a-5p |  | 0 | 0 | 0 | 0 | 0 | 0 | 0 | 0 | 0 | 1 |
| gga-miR-1804 |  | 0 | 0 | 0 | 0 | 0 | 0 | 0 | 0 | 0 | 1 |
| gga-miR-1597-3p |  | 0.39 | 0.16 | 0.2 | 0.26 | 0.14 | 0 | 0.2367 | 0.1333 | -0.8284 | 1 |
| gga-miR-1767 |  | 0 | 0 | 0 | 0 | 0 | 0 | 0 | 0 | 0 | 1 |
| gga-miR-7438-3p |  | 0 | 0 | 0 | 0 | 0 | 0 | 0 | 0 | 0 | 1 |
| gga-miR-217-3p |  | 0 | 0 | 0 | 0 | 0 | 0 | 0 | 0 | 0 | 1 |
| gga-miR-1589 |  | 0 | 0 | 0 | 0 | 0 | 0 | 0 | 0 | 0 | 1 |
| gga-miR-499-3p |  | 0.2 | 0 | 0 | 0.26 | 0 | 0 | 0.0667 | 0.0867 | 0.3783 | 1 |
| gga-miR-1632-3p |  | 0 | 0 | 0 | 0 | 0 | 0 | 0 | 0 | 0 | 1 |
| gga-miR-7479-5p |  | 0 | 0 | 0 | 0 | 0 | 0 | 0 | 0 | 0 | 1 |
| gga-miR-6713-3p |  | 0 | 0 | 0 | 0 | 0 | 0 | 0 | 0 | 0 | 1 |
| gga-miR-3064-3p |  | 0 | 0.16 | 0 | 0.13 | 0 | 0 | 0.0533 | 0.0433 | -0.2998 | 1 |
| gga-miR-7461-3p |  | 0 | 0 | 0 | 0 | 0 | 0 | 0 | 0 | 0 | 1 |
| gga-miR-1570 |  | 0 | 0 | 0 | 0 | 0 | 0 | 0 | 0 | 0 | 1 |
| gga-miR-1794 |  | 0.39 | 0.16 | 0 | 0.39 | 0 | 0 | 0.1833 | 0.13 | -0.4957 | 1 |
| gga-miR-6601-3p |  | 0 | 0 | 0 | 0 | 0 | 0 | 0 | 0 | 0 | 1 |
| gga-miR-7483-5p |  | 0 | 0 | 0 | 0 | 0 | 0 | 0 | 0 | 0 | 1 |
| gga-miR-1713 |  | 0 | 0 | 0 | 0 | 0 | 0 | 0 | 0 | 0 | 1 |
| gga-miR-7473-5p |  | 0 | 0 | 0 | 0 | 0 | 0 | 0 | 0 | 0 | 1 |
| gga-miR-7458-3p |  | 0 | 0 | 0 | 0 | 0 | 0 | 0 | 0 | 0 | 1 |
| gga-miR-1816 |  | 0 | 0 | 0 | 0 | 0 | 0 | 0 | 0 | 0 | 1 |
| gga-miR-6677-5p |  | 0 | 0 | 0 | 0 | 0 | 0.15 | 0 | 0.05 | 2.3219 | 1 |
| gga-miR-6545-5p |  | 0 | 0 | 0 | 0 | 0 | 0 | 0 | 0 | 0 | 1 |
| gga-miR-6567-5p |  | 0 | 0 | 0 | 0 | 0 | 0 | 0 | 0 | 0 | 1 |
| gga-miR-1761 |  | 0 | 0 | 0 | 0 | 0 | 0 | 0 | 0 | 0 | 1 |
| gga-miR-6571-5p |  | 0 | 0 | 0 | 0 | 0 | 0 | 0 | 0 | 0 | 1 |
| gga-miR-1722-3p |  | 0 | 0 | 0 | 0 | 0 | 0 | 0 | 0 | 0 | 1 |
| gga-miR-6641-5p |  | 0 | 0 | 0 | 0 | 0 | 0 | 0 | 0 | 0 | 1 |
| gga-miR-7466-5p |  | 0 | 0 | 0 | 0 | 0 | 0 | 0 | 0 | 0 | 1 |
| gga-miR-1714 |  | 0 | 0 | 0 | 0 | 0 | 0 | 0 | 0 | 0 | 1 |
| gga-miR-16c-3p |  | 0 | 0 | 0 | 0 | 0 | 0.15 | 0 | 0.05 | 2.3219 | 1 |
| gga-miR-1773-3p |  | 0 | 0 | 0 | 0 | 0 | 0 | 0 | 0 | 0 | 1 |
| gga-miR-1695 |  | 0 | 0 | 0 | 0 | 0 | 0 | 0 | 0 | 0 | 1 |
| gga-miR-6649-5p |  | 0 | 0 | 0 | 0 | 0 | 0.15 | 0 | 0.05 | 2.3219 | 1 |
| gga-miR-7457-5p |  | 0 | 0 | 0 | 0 | 0 | 0 | 0 | 0 | 0 | 1 |
| gga-miR-7443-3p |  | 0 | 0 | 0.2 | 0 | 0 | 0 | 0.0533 | 0 | -2.4141 | 1 |
| gga-miR-1753 |  | 0 | 0 | 0 | 0 | 0 | 0 | 0 | 0 | 0 | 1 |
| gga-miR-7475-3p |  | 0 | 0 | 0 | 0 | 0 | 0 | 0 | 0 | 0 | 1 |
| gga-miR-1800 |  | 0 | 0 | 0 | 0 | 0 | 0 | 0 | 0 | 0 | 1 |
| gga-miR-1802 |  | 0 | 0 | 0 | 0 | 0 | 0 | 0 | 0 | 0 | 1 |
| gga-miR-7440-5p |  | 0 | 0 | 0 | 0 | 0 | 0 | 0 | 0 | 0 | 1 |
| gga-miR-1354 |  | 0 | 0 | 0 | 0 | 0 | 0 | 0 | 0 | 0 | 1 |
| gga-miR-6576-3p |  | 0 | 0 | 0 | 0 | 0 | 0 | 0 | 0 | 0 | 1 |
| gga-miR-6546-3p |  | 0.2 | 0.31 | 2.4 | 0.79 | 0.69 | 0.77 | 0.9667 | 0.75 | -0.3662 | 1 |
| gga-miR-6685-3p |  | 0 | 0.16 | 0.5 | 0 | 0.55 | 0 | 0.2133 | 0.1833 | -0.2187 | 1 |
| gga-miR-1621-3p |  | 0 | 0 | 0 | 0 | 0 | 0 | 0 | 0 | 0 | 1 |
| gga-miR-6596-5p |  | 0 | 0 | 0.2 | 0 | 0 | 0 | 0.0533 | 0 | -2.4141 | 1 |
| gga-miR-1583 |  | 0 | 0 | 0 | 0 | 0 | 0 | 0 | 0 | 0 | 1 |
| gga-miR-107-5p |  | 0 | 0 | 0 | 0 | 0 | 0 | 0 | 0 | 0 | 1 |
| gga-miR-7469-5p |  | 0 | 0 | 0 | 0 | 0 | 0 | 0 | 0 | 0 | 1 |
| gga-miR-1610 |  | 0 | 0 | 0 | 0 | 0 | 0 | 0 | 0 | 0 | 1 |
| gga-miR-6581-3p |  | 0 | 0 | 0 | 0 | 0 | 0 | 0 | 0 | 0 | 1 |
| gga-miR-7447-3p |  | 0 | 0.16 | 0 | 0 | 0 | 0.15 | 0.0533 | 0.05 | -0.0922 | 1 |
| gga-miR-6683-3p |  | 0 | 0 | 0 | 0 | 0 | 0 | 0 | 0 | 0 | 1 |
| gga-miR-1627-3p |  | 0 | 0 | 0 | 0 | 0 | 0 | 0 | 0 | 0 | 1 |
| gga-miR-1704 |  | 0 | 0 | 0 | 0 | 0 | 0 | 0 | 0 | 0 | 1 |
| gga-miR-6568-3p |  | 0.2 | 0.31 | 0.2 | 0 | 0 | 0.46 | 0.2233 | 0.1533 | -0.5426 | 1 |
| gga-miR-6684-5p |  | 0 | 0 | 0 | 0 | 0.14 | 0 | 0 | 0.0467 | 2.2234 | 1 |
| gga-miR-7462-3p |  | 0 | 0 | 0 | 0 | 0 | 0 | 0 | 0 | 0 | 1 |
| gga-miR-6588-3p |  | 0 | 0 | 0 | 0 | 0 | 0 | 0 | 0 | 0 | 1 |
| gga-miR-1744-5p |  | 0.2 | 0 | 0 | 0.13 | 0 | 0 | 0.0667 | 0.0433 | -0.6233 | 1 |
| gga-miR-6626-5p |  | 0 | 0 | 0 | 0 | 0 | 0 | 0 | 0 | 0 | 1 |
| gga-miR-7481-3p |  | 0 | 0 | 0 | 0 | 0 | 0 | 0 | 0 | 0 | 1 |
| gga-miR-6649-3p |  | 1.77 | 1.86 | 1.1 | 1.7 | 0.96 | 1.08 | 1.58 | 1.2467 | -0.3418 | 1 |
| gga-miR-1586 |  | 0 | 0 | 0 | 0 | 0 | 0 | 0 | 0 | 0 | 1 |
| gga-miR-1b-5p |  | 0 | 0 | 0 | 0 | 0 | 0 | 0 | 0 | 0 | 1 |
| gga-miR-19a-5p |  | 0 | 0 | 0 | 0 | 0 | 0 | 0 | 0 | 0 | 1 |
| gga-miR-6554-5p |  | 0 | 0 | 0 | 0 | 0 | 0 | 0 | 0 | 0 | 1 |
| gga-miR-1701 |  | 0 | 0 | 0 | 0 | 0 | 0 | 0 | 0 | 0 | 1 |
| gga-miR-3594-5p |  | 0 | 0 | 0 | 0 | 0 | 0 | 0 | 0 | 0 | 1 |
| gga-miR-6677-3p |  | 0.59 | 0.16 | 0 | 0 | 0.14 | 0.46 | 0.25 | 0.2 | -0.3219 | 1 |
| gga-miR-1778 |  | 0.2 | 0 | 0 | 0 | 0 | 0 | 0.0667 | 0 | -2.7377 | 1 |
| gga-miR-365b-5p |  | 0 | 0 | 0 | 0 | 0 | 0 | 0 | 0 | 0 | 1 |
| gga-miR-1721 |  | 0 | 0 | 0 | 0 | 0 | 0 | 0 | 0 | 0 | 1 |
| gga-miR-1462-5p |  | 0 | 0 | 0 | 0 | 0 | 0 | 0 | 0 | 0 | 1 |
| gga-miR-1809 |  | 0 | 0 | 0 | 0 | 0 | 0 | 0 | 0 | 0 | 1 |
| gga-miR-6612-5p |  | 0 | 0 | 0 | 0 | 0 | 0 | 0 | 0 | 0 | 1 |
| gga-miR-1655-3p |  | 0.2 | 0 | 0 | 0 | 0 | 0 | 0.0667 | 0 | -2.7377 | 1 |
| gga-miR-7474-5p |  | 0 | 0 | 0 | 0 | 0 | 0 | 0 | 0 | 0 | 1 |
| gga-miR-6661-5p |  | 0 | 0 | 0 | 0 | 0 | 0 | 0 | 0 | 0 | 1 |
| gga-miR-27b-5p |  | 28.94 | 25.5 | 25 | 19.76 | 21.1 | 24.4 | 26.3533 | 21.75 | -0.277 | 1 |
| gga-miR-1751-5p |  | 0.59 | 0.62 | 0.3 | 0 | 0.41 | 0.93 | 0.51 | 0.4467 | -0.1912 | 1 |
| gga-miR-6563-5p |  | 0.39 | 0 | 0 | 0 | 0.14 | 0.15 | 0.13 | 0.0967 | -0.4269 | 1 |
| gga-miR-7466-3p |  | 0 | 0 | 0 | 0 | 0 | 0 | 0 | 0 | 0 | 1 |
| gga-miR-1674 |  | 0.2 | 0 | 0 | 0 | 0.14 | 0 | 0.0667 | 0.0467 | -0.5143 | 1 |
| gga-miR-7468-3p |  | 0 | 0 | 0 | 0 | 0 | 0 | 0 | 0 | 0 | 1 |
| gga-miR-1614-3p |  | 0 | 0 | 0 | 0 | 0 | 0 | 0 | 0 | 0 | 1 |
| gga-miR-1709 |  | 0 | 0 | 0 | 0 | 0 | 0 | 0 | 0 | 0 | 1 |
| gga-miR-6685-5p |  | 0.39 | 0.62 | 1 | 0.39 | 0.41 | 1.08 | 0.6533 | 0.6267 | -0.06 | 1 |
| gga-miR-6634-5p |  | 0 | 0 | 0 | 0 | 0 | 0 | 0 | 0 | 0 | 1 |
| gga-miR-1627-5p |  | 0 | 0 | 0 | 0 | 0 | 0 | 0 | 0 | 0 | 1 |
| gga-miR-1777 |  | 0 | 0 | 0 | 0 | 0 | 0 | 0 | 0 | 0 | 1 |
| gga-miR-1679 |  | 0 | 0 | 0 | 0 | 0 | 0 | 0 | 0 | 0 | 1 |
| gga-miR-6672-3p |  | 0 | 0 | 0 | 0 | 0 | 0 | 0 | 0 | 0 | 1 |
| gga-miR-1743 |  | 0 | 0 | 0 | 0 | 0 | 0 | 0 | 0 | 0 | 1 |
| gga-miR-6596-3p |  | 0.39 | 0 | 0 | 0.26 | 0 | 0 | 0.13 | 0.0867 | -0.5844 | 1 |
| gga-miR-7463-5p |  | 0 | 0 | 0 | 0 | 0 | 0 | 0 | 0 | 0 | 1 |
| gga-miR-1789 |  | 0 | 0 | 0 | 0 | 0 | 0 | 0 | 0 | 0 | 1 |
| gga-miR-6570-5p |  | 0 | 0.16 | 0.2 | 0.13 | 0 | 0.15 | 0.1067 | 0.0933 | -0.1936 | 1 |
| gga-miR-1577 |  | 0 | 0 | 0 | 0 | 0.14 | 0 | 0 | 0.0467 | 2.2234 | 1 |
| gga-miR-1652 |  | 0 | 0 | 0 | 0 | 0 | 0 | 0 | 0 | 0 | 1 |
| gga-miR-1605 |  | 0 | 0 | 0 | 0 | 0 | 0 | 0 | 0 | 0 | 1 |
| gga-miR-1735 |  | 0 | 0 | 0 | 0 | 0 | 0 | 0 | 0 | 0 | 1 |
| gga-miR-6681-5p |  | 0 | 0 | 0 | 0 | 0 | 0 | 0 | 0 | 0 | 1 |
| gga-miR-3531-3p |  | 0 | 0 | 0 | 0 | 0 | 0 | 0 | 0 | 0 | 1 |
| gga-miR-1645 |  | 0 | 0 | 0 | 0 | 0 | 0 | 0 | 0 | 0 | 1 |
| gga-miR-1775-5p |  | 0 | 0 | 0 | 0 | 0 | 0 | 0 | 0 | 0 | 1 |
| gga-miR-1643-5p |  | 0 | 0 | 0 | 0 | 0 | 0 | 0 | 0 | 0 | 1 |
| gga-miR-1797 |  | 0 | 0.16 | 0 | 0.13 | 0 | 0.15 | 0.0533 | 0.0933 | 0.8077 | 1 |
| gga-miR-7471-5p |  | 0 | 0 | 0 | 0 | 0 | 0 | 0 | 0 | 0 | 1 |
| gga-miR-7449-5p |  | 0 | 0 | 0 | 0 | 0 | 0 | 0 | 0 | 0 | 1 |
| gga-miR-6657-3p |  | 0.2 | 0 | 0 | 0 | 0.14 | 0 | 0.0667 | 0.0467 | -0.5143 | 1 |
| gga-miR-6635-5p |  | 0 | 0 | 0 | 0 | 0 | 0 | 0 | 0 | 0 | 1 |
| gga-miR-7437-5p |  | 0 | 0 | 0 | 0 | 0 | 0 | 0 | 0 | 0 | 1 |
| gga-miR-6679-5p |  | 0 | 0 | 0 | 0 | 0 | 0 | 0 | 0 | 0 | 1 |
| gga-miR-1755 |  | 0 | 0 | 0 | 0 | 0 | 0 | 0 | 0 | 0 | 1 |
| gga-miR-190a-3p |  | 0 | 0 | 0.3 | 0.13 | 0 | 0.15 | 0.1067 | 0.0933 | -0.1936 | 1 |
| gga-miR-6592-3p |  | 0.39 | 0.16 | 0 | 0 | 0.28 | 0.15 | 0.1833 | 0.1433 | -0.3552 | 1 |
| gga-miR-1621-5p |  | 0 | 0 | 0 | 0 | 0 | 0 | 0 | 0 | 0 | 1 |
| gga-miR-7470-3p |  | 0 | 0 | 0 | 0 | 0 | 0 | 0 | 0 | 0 | 1 |
| gga-miR-6617-3p |  | 0 | 0 | 0 | 0 | 0 | 0 | 0 | 0 | 0 | 1 |
| gga-miR-1791-3p |  | 0 | 0 | 0.3 | 0 | 0.28 | 0 | 0.1067 | 0.0933 | -0.1936 | 1 |
