# Supplementary material for: Analysis of deep sequencing exosome‐microRNA expression profile derived from CP‐II reveals potential role of gga‐miRNA‐451 in inflammation
Source: J Cell Mol Med. 2020 Apr 19;24(11):6178–90. doi: 10.1111/jcmm.15244 (PMC7294135; doi:10.1111/jcmm.15244)
Supplement: Supplementary file 7 — TableS6 [file JCMM-24-6178-s007.docx]

**Supplementary Table 6: Predicted target genes of DEGs**

| **miRNAs** | **Target Genes** |
| --- | --- |
| gga-let-7d | HRAS SPATS2L ELP6 BCMO1 SGMS1 CFI DNMT3B ATP6AP1 MRPS17 CDKN1B LDB3 FBXW11 COX14 SFTPA1 HPS1 BPGM LYG2 IGF1 PALM AHR C18ORF42 OVAL TMEM26 CINP PAH CD247 ELOVL6 PTGES3 LOC431324 NRG4 TANGO2 UQCRH CRYBB1 ANKRD40 TANK TSC22D3 RNF141 NAGA SNRNP200 ODC1 DDT RNASEL CYB5A CD82 JAK2 KRIT1 FDFT1 GJA1 WDR18 HSPH1 TMEM170A MAP6 SLC17A5 GLOD4 GCM1 EGFL7 PRIMPOL FOS SOCS2 CREG1 POLE3 SERPINE2 AANAT MAT1A LINGO1 CMC2 PARP4 ORC5 RGS7 VIP ITGB2 IAH1 RABL2B ZFYVE21 ABCC4 SLIT1 PCMTD1 ATG5 ANKDD1A ZBTB2 COL9A3 TBC1D15 TSKU LOC431499 GTF2H4 C12H3ORF37 BET1L BRINP1 RASGEF1C ERLIN1 DOHH N6AMT2 IRF7 EDA2R P4HA2 MGAT3 DNAJC15 MGEA5 PTPRZ1 BCAP29 PGK1 IP6K2 PAN2 KLHL6 UBL7 GRK4 OPN4 TPST2 ANGPTL2 ST3GAL5 MAP2K2 C8H1ORF27 PRRX2 IFIH1 NARFL WRAP73 NPC2 TGFBR1 PPHLN1 TMEM68 RAF1 SERTAD2 TYRO3 SUPV3L1 DNAJA1 ZNF639 PDLIM7 SERHL2 STRADA EIF5B TBCK IL22 CDH11 COX17 CETP FARSA AZIN1 PSME4 THY1 TSPAN1 MPC1 CTSL2 ARHGAP19 DLD SSTR2 CENPH LIG4 TMEM121 TRIB2 VDAC1 NFIL3 PYGB POPDC2 CDC42BPA AC113404.1 MAPT SSU72 CDKN1A HNRNPH1 CD24 COL4A2 DAZAP1 SEC11A PMAIP1 RFC1 AP1G1 PTRH2 RB1 SZRD1 MTIF2 ST3GAL3 ELF1 C11H16ORF70 CCL20 LXN WBP2 NUDT5 SLC18B1 PBRM1 KCNT1 RSU1 USP12-like EMP1 UBE2V2 GOLGA7 APELA TAF11 SLMO2 SLC34A2 TST SULT1B ASIP E2F1 SLC39A13 FZD5 MYOCD JUN SOCS6 PRKRIP1 PCNP CIRBP HDAC4 SOD2 HOXB1 TMEM184B ALDH1A3 ITGB1 FZD7 EHMT1 SLC9A2 MET PCMT1L TBX5 NUB1 SALL4 TRIM8 C7H2ORF69 TBX22 PIK3CB MYBPC3 WDR91 YPEL5 CLDN2 STX6 CPZ DAGLB THYN1 B3GAT1 MYH10 SCN9A NVL CHCHD4 GHSR GCC1 NPPC CNOT1 GLRX MXRA8 TMLHE LRIG3 C1ORF146 UBE2R2 TRMU MORC3 CLDN1 AVEN HDAC11 FAM49A RAB19 PIP5K1B CELF2 UBE3C CAPN1 SCPEP1 TMED5 CALM1 CLTB YIPF3 SETD6 SLCO2A1 ALKBH3 PAFAH1B1 HNRNPR FAM118B TCEANC2 SERINC3 ATP5G3 ELL GSTT1 ATP4B KIAA1467 VPS4B WNT2B OSTF1 PRLR CTGF POFUT1 RAB3IL1 AP1S3 CSRP2 DDAH1 PPARG ESF1 CDH20 MYOM2 WDR61 RGL1 PNO1 KIAA1671  RP11-49K24.9 SDF4 FMOD GPR149 CST3 ITM2B SP1 TPM2 BTN1A1 ARHGAP26 RARS2 NEIL1 PLEKHB2 9-Sep GEMIN2 ARHGAP25 RRP7A APOH MRRF LEF1 FMO3 SLC8A3 ST3GAL2 CLTC T MTMR8 CETN1 BTBD9 PRL SDCBP TTC7A CTSA GATA4 ROR1 PLEKHF2 IL12RB2 COL5A1 STIM1 RPS14 AKAP2 EPGN LOC426914 TRIM14 SYNGR3 FEN1 RGS16 ATP1B3 ANKRD27 UBE2G1 PPP2R4 RFTN1 SOCS5 PPP2R5C AP2A2 CHMP4C SCYL3 ANAPC13 LMBRD2 BSG CYP1C1 PDGFB NFU1 BIN1 CLSPN GLUL EED METTL16  RP11-724O16.1 RP4-613B23.5 SEPSECS PMS1 GJA4 DTYMK NEUROG1 COL22A1 THRSP PRC1 GNGT2 PRKAA2 SLC38A4 NFRKB RAD51D COL12A1 EGFR SCAF4 TCP11L2 SSB MGLL DPH1 GTF3C3 CHST10 HCCS INIP THOC3 TBK1 B4GALT2 GNG2 ADCK1 MXD4 DYRK2 ALDH3A2 CDH8 EAF2 MGAT4A YARS USP10 CAPN3 NINJ1 MYLK2 PRKAA1 ID2 GTDC1 SH3KBP1 POR DYNLL2 TBL1XR1 CUTC PYGO1 EFHD1 PIK3CA ORC3 TRPV4 HIBCH PTTG1 NFAT5 PGR SPINZ FAHD1 RALGAPB ACTN1 ARMC7 GALNT6 UTS2B OAT PDE6H MTPN TUBA3E PDLIM5 RAD54L NUCB2 FRS2 TRAF5 HACD3 KLHL15 ANKRA2 SAMM50 ALB SLC26A5 GEM VLDLR LOC420849 SERINC1 RNH1 CLN8 GBAS UBXN10 TOP1 TXN2 PCASP2 FAM192A ACOT9 PRKAR1A CLEC3A WBP4 KPNA1 BIRC2 TBX4 KCNMB1 CAPN11 ADORA2B STK40 ARHGAP29 USP1 SCOC SRGAP1 RHNO1  RP1-309K20.6 RNASEH2B ODF2 RP11-20I23.1 NHLRC2 THBS1 STRAP ATP6V0D2 DYM TSN CALD1 IGFBP4 NRXN1 ACSL1 FUBP1 MKKS TMEM229B PPME1 FAM60A DYNC1LI2 SLC9A3R1 LOC422090  CTD-2410N18.5 ESR1 ETFDH LOC422926 INTS2 NR2E1 SLC16A7 AMIGO2 PRPH2 ABI1 SEMA3D E2F4 STAT4 SLC1A2 CD40 COX16 FBXW2 PDCD5 PLEKHO1 CHRNA6 ATP6V1D HIC2 PPM1M MAP1LC3B CBX3 OCM SEMA3A GRIA2 SMIM18 ATG7 LGALS3 RRAD ADA UFSP2 AREG GJB6 CHADL ITGA8 ASF1A SMAD1 TAPBPL PDGFC ASB7 WRB GALNT1 RHOJ KIAA1191 ADAM10 KCTD7 AP4B1 CBX1 LOXL2 NME5 PRKAB2 VAV2 CDPF1 GTF2H1 FZD6 CEP112 AATF BLOC1S5 TMEM254 ARHGEF39 NDE1 SUCLG1 MBL2 YWHAG MAOA CDA MESDC2 MTX3 RTN4 EYA2 LOC100857579 GNB1L MAFF FBXL16 RP11-87C12.2 NPAS2 CCDC127 SIX1 PTN CACNA1B C7H2ORF76 USPL1 PSMC3 SSBP1 PPP1R9B PPP6R3 G2E3 DNMT1  RP11-290H9.2 PPP1R2 VDR ADIPOQ TADA2A MGAT4C PCGF2 FAM46C EIF4A2 CDH6 NRK EIF2AK2 C28H19ORF10 CLIP1 IMPG1 VSNL1 P2RX4 PLEKHM1 AP3M1 AKR1B10 CST7 LOC769139 BZW1 CSRP3 RAD52 GAPVD1 HAO1 PDP1 ZNF800 HAPLN1 PPDPF HMGA1 PUF60 API5 WDR92 EDN2 QDPR MAPKAPK5 RHOBTB1 CISD1 PARK7 CDX4 BORCS5 TMEM164 PERP2 LYRM4 DEPDC6 CHP1 SOX10 NRP1 MPST DAAM1 PDGFD PEX13 TEC ALAD HES5 DIO2 PREP ARNTL TUBB2B ADORA3 LOC396531 RRP12 ORM1 C3AR1 SATB1 SLC16A3 MINA ACSBG2 SNAP91 HMGCL GLRX5 CHD1 BRMS1L FOXN2 CD4 PFN2 DRG2 F13A1 ELAVL4 SEC22B TNFAIP8L1 PARN CAMK2D IRAK2 EPHA1 E2F6 C1D INCENP MPPED2 NT5C3A MSRB3 GNG5 ARMC1 IPPK CHN1 FAM3C NDUFS1 CHD2 LOC419112 WDR1 EXOC2 MEF2A GMFB PSEN2 MYL2 CAT XIRP1 USP37 TXLNB C10H15ORF61 MORN4 EIF5A2 GINM1 ANKRD16 RARRES2 C11H19ORF12 RINT1 FZD4 NFKB2 RASSF2 YBX3 ESD HMGCS1 RP11-514O12.4 DUSP6 CHAF1A TLR7 GLI2 ENOX2 MARCO FANCC CCK ZIC1 MBOAT1 JAK1 GPR107 CCDC174 PHLDA2 CNR1 CKMT2 TMEM45A 5-Sep ZDHHC18 ARL14EP C15H12ORF49 USP6NL PRRG4 KLHL14 CNOT7 PTPRO CD200 ECE1 KCTD2 EDNRB ARR3 CD276 MAP3K14 PRLH GIGYF2 GLCCI1 TNFRSF18 PAPD7 WNT11 GSTA3 EIF6 RECQL5 DDX6 CANX CDCA4 VCAN ATF7IP CDC42SE2 XIAP TMOD4 CD99 CUL2 ACLY CEPT1 NELFA SPI1 TMEM129 ACOX1 SYCE3 ASH2L EPB41 CNTN2 CHRDL1 NEK6 LGI2 SLC19A1 LOC769121 LOC100859249 CSF3 PBX3 SNCB MSN PTH1R RNF152 LOC426385 PKM RPAP3 RAB8A LOC396380 SLC25A22 N4BP2L2 SLC6A9 MRPL20 FAM46A AC025048.1 CAMKMT ADH1C STAU1 MAP2K1 ZNF767 MIB2 TMEM104 CTSB KCNH6 UQCC RAG2 ABHD13 DYDC1 EI24 CGA ASNSD1 LOC395647 RNF4 LYSMD2 CCKAR NFKB1 NGF VGLL2 GOSR1 TCP11L1 MXD1 ACAP2 ENTPD8 DMTF1 MMP9 TRIM59 ACTR6 SHH TXNDC5 FAM173A NEK7 CCNE2 S100Z CD93 FGF10 NRG1 XPO5 FAM26E LYRM1 KLHDC2 PDS5B SMCO4 SKIL DRAXIN ADSS LOC421975 TRIAP1 DHX38 PPARD BZW2 MYH1B LOC693265 SLC7A6OS HMHA1 CCDC6 GNPTG PPP4R2 CDCA7L EXOC5 KATNBL1 GABPA SRC 5-Mar GSN MON2 STOML1 GOT1 FGFR2 LOC415756 MYH1C EPYC CNP SERPINF1 ANXA11 FK27 KCTD9 CASQ2 FEM1B IL8L2 BUB1B LOC428335 TMOD3 FYTTD1 DPF3 CASP6 RECQL MAGI3 SLBP MED9 QARS DEK RANGAP1 LOC100858381 TSPAN3 CFAP36 TPI1 INTS7 LOC418667 UBAP1L SPP2 TTL PTK7 PGM1 FAM18B1 PLAC8L1 PTPN6 WDR5 ASB12 CPNE1 CPS1 RUNX1 IFNGR2 RGS4 TRIM39.2 UGP2 MEAF6 LPAR4 RNF13 SMARCE1 DYNC1LI1 CPSF6 RBP GFI1B SLC46A3 NDEL1 HHATL OTUD6B CRH WWP2 ACADSB GPR137B PLEKHJ1 RLBP1 YTHDF3 ICOSLG TOB1 AHCYL1 PRNP SMIM3 GTF2H5 LHFPL5 DCTD TMEM180 SLC39A9 TXNRD3 SOUL BRAP HNMT DNAJC12 MYH9 SDR16C5 STK17A ARHGAP15 METTL22 MAP2K5 CTTN DEXI TIMM8A GPATCH2 BRCA1 EIF1AY ADAM9 IL21R CCNA2 SSX2IP KATNB1 TBX18 KIAA1143 ALDH1A1 TRPC4AP CTCF IL8L1 TOLLIP LOC425362 H2AFY2 SYT1 STXBP3 BRCA2 LOC100859722 NUDC RREB1 N6AMT1 ANK3 LOC431325 ZFHX3 PDPK1 RHOG REEP5 STX17 VPS45 EGR1 SCIN CEP63 AKR1A1 ZW10 TSPO IVD AKT1 NDUFC2 DSTN GAD1 15-Sep GPATCH11 MRPS7 DNAJB12 MFGE8 LZIC FGFR1OP2 RASGRP3 FLNB SLC47A1 NUTF2 USP7 MYO5A HNRNPH3 ACTR2 MTF1 SPG21 RQCD1 NCOA4 PBLD CD36 SPATA2 NT5C2 FASN ATOX1 N4BP3 CYTH1 PTP4A1 TBL3 MFAP1 TXNRD1 CCDC80 AL158801.1 DCT HOPX HCLS1 CD151 CHD7 NKAP AGO4 STX7 MRPS33 CSF3R LYPLA2 MLX TLR4 PDIA4 RNF185 TMEM230 UPRT SMIM15 HERC3 HMGN1 PTGS2 KIF2A ELP3 SCNN1A NUMB RFFL HAUS2 DNAL4 FIGF CD1C KLHL20 PNAT10 HIPK3 FADS2 CASP18 NEDD1 VNN1 AHSA2 NUP50 EPHA7 ADAT1 TMED8 ZNF609 RASSF5 CLOCK MAFB GANC TAX1BP1 CACFD1 SOCS4 DCK MAPK6 RFNG LYN DDX4 MAPKAP1 NAT ENO1 CTD-2370N5.3 ST3GAL1 COL6A2 AvBD8 DPP4 MUM1 6-Sep C11H19ORF40 KCND2 APCDD1 SCG5 LRRC28 DCAF13 INSIG2 PCGF5 ROM1 PSMD1 ALCAM KDSR MYL9 SLN ACP2 CDK10 RNF14 UGT8 PAIP2 MITF PMP22 SLC25A14 NR5A2 ERGIC2 PHTF2 LMO4 TOB2 SH3BP2 KBP RGS19 WAPAL TRAPPC3 CBFB SYNCRIP TMPO KIF4A GMPR RORA STX2 COL1A2 C3H6ORF154 STAU2 HOOK1 WASL MELK TNC  DYNLT3 RP11-77K12.7 C1H12ORF73 GPR174 IRF2 PEX2 DAP APOV1 PKIG CHUK SUPT3H FAM210A CACNA1D OXNAD1 P2RY1 OSTN FOXD3 ATP6V0E1 GJC2 SDK1 INO80 NCK2 PDE4B RAB35 HN1 CDH1 SLC2A2 CEBPG ZBTB34 CYB5B ARHGDIA CPT2 C1H11ORF54 PCM1 METAP1 IGF2 ART4 RASSF3 MYO1C MYH1A SCYL2 HINTW PLA2G7 SHOC2 CRTAP TNFAIP6 GID8 AIDA GSTO1 SASS6 QSER1 MYD88 LOC427470 SIGIRR GTF3C5 MYL12A RAB10 PLIN4 FUNDC1 SYK PSMD3 GNB5 VAC14 ANAPC10 DNAJC5 RCAN3 LOC100859616 SLC24A1 FAM213A ZFAND6 MAVS SLC35G2 ABLIM1 DHFR GJB1 ELOVL5 CDH17 TDRD7 PLCXD1 KPNA2 KRT5 HMGA2 NOP56 EPN2 ACBD5 SERPINH1 OAZ2 CMPK1 TEAD4 RIPK1 LRRK2 FGF1 FYB BMPR2 RUVBL1 FAT3 PRKCI TFEB CPT1A SRF DCAF7 LOC420160 TERF1 PDGFRA LHX9 FLT4 TSSC4 ZC3H6 SUB1 SPG7 SGMS2 METTL2A DDX1 CTNNB1 SPPL2B IL5RA BMP5 VTN BTF3L4 HAS2 BEAN1 HVCN1 FOCAD C12ORF57 ZNF302 NCOA1 ELMOD2 PLIN2 EFHC2 IL10RA FBXO8 MEF2BNB RBM48 C4H4ORF29 VPS50 NEMP2 MTHFS CUEDC2 HAVCR1 COL8A1 COX6C ABCA1 CCR8 PRRG1 SETD3 NR2E3 OGN CD200R1L AKR1D1 BAK1 FGFRL1 STK10 VMO1 TMEM141 KCNJ8 SKP2 HPGDS NGEF JUP CCR5 AICDA SETD4 FGFBP2 TMEM123 C1H2ORF49 PLIN1 GFPT1 RPL6 CYP1A4 EMB FBLN5 TCEB1 E2F5 AAMP NR5A1 RNF11 RPS12 ATP13A4 ELK3 FOXA2 YTHDC1 SLC16A8 TNS1 ATG9A NR1D2 HOXB5 RGS20 AP2M1 MGME1 DHRS3 PPM1B GCLM UBE2G2 EIF2B2 TNFRSF19 RNF111 HDAC7 TADA1 EXOC6 RTKN2 ZNF706 ANXA7 CNGA3 ACACA REL PITX2 KTN1 OPN5L1 PROM1 F-KER TOP2B SLC15A1 C1H7ORF60 MIF FAH NCF4 CREB1 CYP1A1 CECR1 ECHDC3 PPFIBP1 HNRPK RGS6 MFAP3 FAM13B TUBAL3 RAB40B POP4 ACTR5 MYH11 GHRH IMPDH2 PEX11G MTFR1 HPCAL1 RPL29 PAQR8 CHIC1 TUBGCP2 PIK3CD RER1 ACO1 PSEN1 EIF2B3 RP11-101E3.5 TBC1D1 LCP1 C14H17ORF103 GFOD2 SNX10 FAM20B SST C8H1ORF52 NFKBIZ PRPSAP2 COL6A3 YWHAZ CELF1 ZDHHC21 DCBLD2 USP45 ARFIP1 ARL6IP5 TPH1 MASP1 PRPF3 RALGPS2 PHF20L1 AARS2 CDK6 SLC7A9 GET4 ENSA TJP2 ZP2 LIN52 RALBP1 MRPL44 STK32A RP11-295K3.1 BLEC1 PIGY AMY1A TULP1 APLF GLT1D1 NOTCH2 MBLAC2 SLC40A1 IL6ST VMA21 ATM RPL35A NCL FBXO34 MST4 ELMO3 KLF6 TOMM7 UGGT2 LUM ZNF512B RASA3 CMKLR1 XKR8 TMEM178B SRSF3 TDP2 LPP TYRP1 MRPL38 ANGPT2 MVB12B SPTSSB RGS9BP MIER1 FKBP8 CTXN3 AAMDC NSG1 AvBD9 SUGP1 MXI1 AMPD3 HOXD8 PPP1R12B BCL2 DDB2 MAD2L2 GATA2 MAFA SENP8 KNSTRN TACR1 FAR1 PQLC2 TNNT3 TICAM1 CLDND1 RPS19BP1 COPS8 ACAN STRA6 P2RY14 FOLR1 BTC ZDHHC17 CANT1 ALDH1A2 FAM177A1 FOXO1 CPA6 TNNI2 SLC35E3 STK11 IFITM5 SLC51A CORO1C DFNA5 SET RHBG DUSP10 REG4 EVL SDR42E2 NUSAP1 SNX24 AAR2 ZBTB17 TNFSF8 RRAGC BBS4 RABGAP1L POT1 ELAVL1 KDELR2 NME2 MPP5 GINS1 CD3E MDH1 OPNP INVS MAPK9 SLC17A9 LIMD2 BLOC1S6 ZFYVE1 KIF23 BRE SPCS1 CYP24A1 IKBKB GORASP1 TOP3A OSBPL2 HDGFRP2 WWOX ST13 MGP WNT8A TMEM70 PCDH10 GPX7 USP15 MALL RGS17 POLK SERPINB10 CCDC93 UGDH PLAU AvBD10 TARSL2 TNNT2 KCNA4 AR RP11-403P17.5 ENTPD2 TPRA1 HMG20A SLC35B1 POLDIP3 CIAPIN1 CDV3 OCX36 AvBD1 VPS53 MOV10 ZYX FAM214A LPGAT1 DCUN1D1 HNRNPDL KERA RDH10 PPIB TMEM184C SPERT PMPCB VDHAP IRX4 PARS2 ORMDL2 MAD2L1 TEAD1 CDH5 LOC100859148 KLHL24 8-Mar PPP1R8 C14ORF2 CRYAB EYA4 COPB1 TMEM106B MAL LOC422426 NCOA7 USP48 RPL15 ARRDC1 DKC1 NAPB COX7A2 TGS1 MTMR2 PIP4K2A RIPK2 LOC395991 TTLL5 MLNR CDC27 MOB4 RPSA YWHAQ FAM105A RPA1 ABRACL REXO2 NCF1 LCLAT1 FAM103A1 NPL LOC770548 ORAI2 SRRD NAA50 RBM12 NHLH1 FSHB TINAG SLC25A15 H1F0 BLOC1S2 CETN2 CYP51A1 WASH1 SLC11A1 SERPINI1 POPDC3 GDPD5 MICAL1 CEP19 PHC1 LEPROT HESX1 ERICH1 ADARB1 STK11IP AGPAT9 DCLRE1B DNAJB9 LOC419851 TAF12 TBX6 PLEKHA2 UBXN2A CRYL1 IRF4 LMX1B TNR FAM65B SMIM19 HMGCR CD1B ALG12 IKZF5 PACSIN2 MED6 PTX3 PDDC1 DUSP4 FGB RAB28 CHAC1 ABHD17B BORCS7 GAS8 WDFY2 YPEL2 YWHAB CSNK2A2 ECI1 FZD3 SNAP23 DEAF1 HNRNPD DNAJC6 CENPF G3BP1 NR1H3 GAS2 CCDC167 FZR1 PTPRG C13H5ORF15 RBMS1 PUM1 GABRA1 GREM1 FABP6 DCX PCK1 TREM-B1 DMD LOC426218 TWIST1 ABCF2 ADCK3 SUMO3 YRK FSHR LYRM9 ARFGAP2 RASA2 CARD11 VSX2 RAB14 LOC770639 VPS41 MRPL37 SPC25 KCNMB4 SMIM5 SYNPR CSRP1 LOC424740 STAT1 HOXB4 CBLN2 LOC772071 MATR3 RGS18 HGF TASP1 TEX264 PREPL FUT9 RLIM CHST3 RCHY1 PFKP EIF2S1 S100B H3F3C ERBB3 LDLRAD4 IFNGR1 SF3A1 CTNNA2 SELO TBC1D14 ID3 PODXL PLN FGF14 EIF4G2 ENO2 FAP COL18A1 PTPN2 CD72 TNFRSF1A SGTA RBM12B FAM126A RAB3GAP2 TMEM189 NAV3 ACBD3 CSDE1 RUNDC3B PRDX1 RAB5A WDSUB1 AC005943.2 MTMR3 CMTM8 GFRA2 TRH ZFP92 VIT CDC25A SRSF1 RBM25 GJC1 CSNK1D PLAG1 SLCO4A1 CHM SPP1 NOV DNTT LOC420419 SLC12A7 INPP5K NOTCH1 LDHA CTPS2 PARPBP JMJD4 RNF20 SGK196 WDR45B RNF139 GDF9 ERCC3 CREBL2 FBLN1 TRPC3 SYTL1 KIF5C NREP TARDBP CCNE1 EDNRA WFDC1 SERPINA1 PAX6 DMA ARID4A NDP LFNG ST6GALNAC4 GATAD2A PDCD6IP NCALD NEUROD4 FAM76A FXYD6 CSPG5 OC3 SRP68 TMED11 LYRM2 MAPRE2 FLOT2 PTH FHIT ARFGAP1 SNX20 DNAJB6 C4BPA ARHGDIB C10ORF2 VDAC2 ETV6 DHRS11 DAD1 KCNN2 FTO CNTN5 PROKR2 PAK1 CKB SLC31A2 CBLN4 PRTG TRAPPC4 OVALX GAS2L3 SGK3 PAFAH1B2 PTDSS1 LOC416354 RAC2 C26H6ORF106 RNF34 ADD1 TNIP2 BTD P2RY8 RPL4 CREM CNOT2 WDR70 NCAN PANK4 TRPM7 HIC1 CYP46A1 RBM19 GALK2 BUB1 AARS POU1F1 AQP9 NFYA NTRK2 ATP6V1G1 NKX3-2 ZC3HAV1 COX4I1 CUX1 UBE2J1 IRAK4 UBN1 SGPL1 P4HA1 TMEM39B G0S2 TOX3 CLPX MBP ENTPD1 NKX2-6 ESRP2 GART SEPP1 C2ORF88 PLA2R1 RELL1 IRF1 STAR STRA13 TSPAN15 ZNF384 STUB1 GPBP1L1 CHRM3 MTHFD2 SMPX RAP1GAP2 MAGI2 RABL3 SYNM FKBP4 GCHFR TSPAN13 AKAP9 ACTC1 SMPDL3B WIPF1 RHOB MMACHC CCPG1 RPRD1A C1QTNF2 PPP3CB TNFSF10 FAM114A1 PACSIN3 APPBP2 EEF1A2 ATF4 CDH7 TBC1D22A VWA9 GTF2E2 COL6A1 ATAD5 ULK3 TAPT1 RP11-145E5.5 C1GALT1 ACAD8 ARIH1 RBBP4 CNDP2 SNX16 STK24 LCMT1 ANGPTL5 FAM222B HAGH PLEKHB1 SUV39H2 SMAP2 KANK1 INSIG1 TMEM120B SMIM14 CDH13 ASTL COPA BIVM RTN1 ANO5 IMMP1L SNRNP35 FBXL21 GSTA FAM98A COLEC12 MTMR9 NAP1L4 DDX47 TNS3 ADIPOR2 MBD3 ZCCHC6 PCMT1 MTSS1 FABP5 DDA1 LRRC57 IMMP2L LOC100858797 SCFD1 PRPF38A GDI2 GATM SLC16A1 GJA5 DUT SLIT3 SOCS3 CR1L SRPR ZCRB1 RAB33B RPIA GNB1 SNN PAAF1 C1H12ORF23 LOC418666 CIR1 ZNF622 C3H2ORF43 CITED4 COL17A1 ZFYVE19 FAM45A ELMO1 GLP2R TAF5 STRBP BHLHE40 GABRG2 PHKG1 FLI1 RBMX CFLAR UBLCP1 C20H20ORF24 ZFP64 TAF8 TACC3 CCL1 EIF1 APOB C26H6ORF89 ICMT PSPH CTNNBIP1 BPIFB2 ZNF335 FOXM1 RBM24 COG4 FAM122A APBB1IP HABP4 CL2 CASP14 TMCO3 SIX6 RBM5 RAB11A CRADD CNTRL SMIM12 UCHL5 BASP1 CD44 BMP10 XPO1 TECTA ANP32B SLC38A2 BCL6 SERINC2 BFSP1 LIMK1 AVD HERC2 CPSF4 PEX5 C3H6ORF120 ATP1B4 TNFAIP1 PSMD14 TBL1X CXCL14 COPE CTNNA3 KBTBD4 NBL1 DTWD2 FSTL1 IRF5 NME3 SLC35A3 GHRHR SPPL2A UBE2V1 CHTF8 TMEM173 FRZB MRPL46 BTBD10 FAM102A LIFR SERPINA4 CRCP LOC100859427 CCDC18 MARCKSL1 PELI1 LOC100858504 IL16 SLC16A9 RHOT1 ARF1 TSPAN6 DNASE1 ARFGAP3 A4GALT RAB32 RHOC SZT2 SALL1 CMTM7 KLHL18 UBL3 LSM8 HP1BP3 MRPS26 WSB1 MCCC1 TMEM175 SGCB AGTPBP1 AvBD6 PIK3R5 ZBED4 PDE3B PARD3 FK21 NFASC PIAS2 NUDT19 ZBTB26 EPCAM BF2 PSME3 ZSWIM7 SEPT2L DCN FHL5 HSPA4L RAB9A UBIAD1 ZFAND5 EXOSC2 OCLN CYP11A1 PLS3 NR2C1 CXCR7 HSD3B2 ELOVL1 NCLN FBXW5 STARD4 TMEM56 FPGT BAZ2B LIN28B SSR1 MCEE CYP2D6 HYAL6 ESR2 NRTN DDX42 PRTFDC1 RAN ABCC1 RHOA ANXA2 LRRC59 ITPR1 BUB3 SNX12 NELFB SRP14 GGNBP2 SLC37A3 MCL1 JMJD6 KIF3B TRAPPC2 LUZP2 DAPP1 CD80 PTBP1 KPNA4 PIGA RRP1B SRSF5A TP53I11 PHKB KIAA0020 ACVR1 VAMP7 CAB39L TCF7L2 HDAC9 MYLIP NKIRAS2 USMG5 RRN3 CNTN1 CTDSPL2 FAM175B SLX4IP ST6GALNAC1 BID ARIH2 TLN1 BORA OLFM1 LYSMD3 JARID2 GNAI1 SERPINB6 NUP93 KLHL13 K123 NECAP2 STAT5B OPTN BTG4 NAIF1 CDC42 ZCCHC17 TRABD PIGR SMIM4 LHCGR EDC3 BRSK2 KLF11 SRSF2 RAD21 CD8B TWIST2 F2 KDR RP5-1021I20.4 C1S PSMA4 SULT1B1 NEURL1 GPR89B WWP1 P2RX5 ACAA2 HACE1 CATH1 LOC418424 PDLIM4 GPR126 MAL2 USP13 |
| gga-miR-33-5p | PRKCI ART4 TNFAIP8L1 CLSPN SERTAD2 SLC16A3 GMPR DAGLB MGEA5 STK10 SMPDL3B RNF11 AP4B1 C11H19ORF12 PFN2 ATP6V0E2 CD200 TARDBP SPINZ FGFRL1 HMGA1 DDX4 EEF1A1 HARS TMOD3 CD276 GBAS PRPF3 TMEM120B TRPV4 FAM192A WDR92 NUDT5 XPO5 PABPC1 SH3BP2 RHOJ FGF19 BTBD1 RALGPS2 VDAC2 DHRS3 YEATS4 CHERP LUM FAM126A FABP6 PAIP2 TXNRD3 CREB1 PAFAH1B1 P4HA1 EMB CDH7 RBM24 9-Sep PRRG1 STX2 TBCA RIPK1 USP6NL PSEN2 C26H6ORF89 TIPIN RAG2 FGF1 OAF PLEKHJ1 C12ORF57 CD36 RALBP1 E2F5 RAD21 POLDIP3 RIPK2 WDFY2 CRYL1 TWIST2 RAD51 RAD52 FABP5 ATP6V0A4 GUCA2B OLFM1 AZIN1 CIRBP TBCD CDCA4 ZDHHC8 DRAXIN SYTL1 AC005943.2 GABRA1 PRDX1 APOH MESDC2 MED6 DUSP10 INSIG2 PSEN1 CTDSPL2 SLC2A2 LZIC PTPRO FAHD1 HNRNPD MPP5 IL1RN ITGB1BP3 RBM12B MAGI2 CHMP2A NUDT3 XPO1 KCNA4 NAE1 MIB2 TCP11L1 BCL6 HOXB1 UBE2V2 ADH5 NHLRC2 FZD4 TFEB CFL2 CPT1A PTBP1 MSMO1 ABHD17B PDS5B C1H21ORF33 RECQL5 SCOC PRLR WASH1 PIP4K2A MYF6 LOC100859756 ST3GAL5 ABCC4 PLEKHF2 MORN4 CNTN5 ETV6 FYTTD1 NEK7 ECI1 ERI1 SRF SELO YPEL2 ENS-1 RNF7 IFRD1 SEMA7A ZFHX3 ITPR1 BAK1 WNT2B BEAN1 LY6E RAB28 EPYC PDE3B NCOA1 AC113404.1 RAP1GDS1 CCDC6 MTPN IPPK FAT3 OCM ANKRD16 GART PPP1R8 UBL3 YTHDF3 PCDHGC3 NELL2 CNTN1 ELOVL1 ZBTB26 BIN1 NAA50 RP11-290H9.2 RFC1 TSEN2 PLK1 TAPBPL DDT VTN PEX13 XRCC6BP1 PPHLN1 PRNP C11H16ORF70 FLOT2 ALC LRRC57 TMEM189 VIT OVALX ROM1 G3BP1 ROR1 PNISR COX14 DAP SCD PROKR2 SMAP2 RBMS1 RRAD RAB3GAP2 TMEM68 ELAVL1 TMEM173 LEF1 FHOD1 LOC100857840 FMOD FGL2 CLPX MYLK2 RFTN1 NUP93 TMEM41B HDAC11 C1H12ORF23 GPATCH2 C12H3ORF37 RAB3IL1 SATB1 CNOT1 CLDN3 SPG7 PEX2 FAM46A BIVM VPS41 DEXI FOXO1 SDR16C5 MXD1 ARPP19 CSPG5 MRPL28 CAB39L AC025048.1 CCR8 ABLIM1 CRYZ PFKP ACTG1 RRAGC PSMD14 CDC42 PDCD6IP MET SURF6 HJURP MSRB3 MED9 HSPA4L CD24 POPDC3 GCLM ELK3 LOC100858381 MRPL15 RAB14 TNIP2 WIPF1 CCK RPS14 PLIN4 SLC8A3 CACFD1 SGK1 TP53INP1 PKM FAM46C CRIPT C2ORF88 MYL9 ARG2 CDH6 HOXD11 PAX6 CDC42SE2 GJC2 RNF4 RUNDC3B ARFIP1 NUDT19 SUGP1 SLC6A9 JMJD6 DAAM1 N6AMT2 REL TECTA GNB5 NCL NKX3-2 INSIG1 NAP1L4 SLC1A2 RPL29 LOC422926 STAU2 PIGA ATP6AP1 BBS2 VPS45 DCN TMEM178B NR2C1 ZFYVE1 XIRP1 SLC9A2 CUEDC2 AGMAT DEK ARIH1 FXYD6 POLE3 PIGR SNX16 CD80 PLA2G7 BRAP NCSTN RAB24 SNX10 BIRC2 SOCS4 DMD SF3A1 ARID4A CASP14 SREBF1 FEN1 HRAS RTN1 RAP1GAP2 GLI2 STK24 PHKG1 PARS2 GAS2 ATP4B RPA1 DENR JAK2 GNG11 POFUT1 5-Sep PROM1 DDB2 T ST3GAL2 MVB12B SLC17A9 RP11-101E3.5 SPP2 FBXW5 CSRP3 DUT CRIM1 NGF BLOC1S6 CLTB STK11 YARS BID TBC1D23 FOXN2 BUB1 WRB PALM ATP12A ARHGAP21 PLEKHM1 NECAB3 YPEL5 TMED8 ACAN HAVCR1 KCNMA1 DFNA5 SUB1 CYP51A1 PYY SBNO1 CBFA2T3 PSMA4 SMIM19 ZDHHC21 C15H12ORF49 ABRACL TWIST3 ABHD13 CHST10 TREM-B1 IFNGR1 CDH11 FZD7 RPL17 MGST1 MEOX1 PPARD GALK2 MCCC1 ST6GALNAC4 LOC431324 NEDD1 CTBP1 AAMDC MYD88 TANGO2 ST13 TMEM230 JAK1 SERPINA4 NT5C3A ADA TGFA ATG9A SNX24 GPR107 B3GAT1 ZCCHC6 NR1H3 HSPA9 DOLPP1 ENSA EXOC8 CLDND1 NFIL3 RAF1 GTF2H5 ZFAND6 MAP2K5 RP11-5A19.5 PSPH MST4 TPRA1 GLT8D2 API5 TRIM59 MBD3 PAQR8 PTN CSDE1 TDRD7 UBE2V1 KIAA1671 TSKU SLX4IP GABRG2 CHM IKZF5 CHORDC1 RBM19 POT1 IHH RGS6 TSPAN14 E2F4 FAM65B RGS20 HCLS1 STK11IP ARF1 STOML1 PPM1M ACBD3 SRC NEK6 RP11-49K24.9 LIG4 MFAP3 LOC431499 RHOB DCBLD2 TUBAL3 ENTPD8 TBX4 PTH NT5C2 BCL2 SUCLG1 CHAC1 EXOC2 FZD10 CASP6 NELFB GJA1 GJA5 MAPT ACBD5 KLHDC2 NRGN MYO1C PCGF2 DYNLRB2 CEBPG CSF3R ZNF302 SNCB USMG5 CTCF CRIP1 AKAP2 SEC23B SMAD6 S100B ARNTL2 TRAPPC2 CDH20 SERPINB10 TAX1BP1 VAMP7 TM4SF1 CEPT1 CDKN1A PLA2R1 MEPE MMP2 ACACA VMO1 TBX18 PMP22 C26H6ORF106 PDP1 SRSF2 DEPDC6 DCT IL8L1 METAP1 NCAN CENPF RASGRP3 RAB11A S100A10 ABI1 RBPMS2 DCAF7 FIBIN AKIRIN2 FAR1 MYH1A MCL1 EIF2B2 LOC100859586 NDUFC2 ELMOD2 FZR1 MLX GOT1 COL17A1 ARFGAP1 TAPT1 AR GABRB3 RER1 TM2D3 AARS2 SLC46A3 PTTG1 IRF4 TLDC1 TOX3 ACSBG2 LYN SPPL2B SLCO4A1 RDH10 RAB9A DEAF1 PLN FAM60A EXOC6 UQCRFS1 CSRP2 BARX1 SETD3 PLEKHA2 CHIC1 EYA2 GPR146 RBM12 TRABD CCNK TMEM106B CDX2 TNFAIP1 ALAD PIK3CD CD74 NR1D2 RPL15 PDLIM5 PRC1 BCMO1 PTPRZ1 NEUROG1 BTG4 SCFD1 C7H2ORF69 ZBTB2 CHAF1B GJC1 TMEM129 GLRX5 NAGA LOC100858439 SLC35E3 LOC426385 FGFR1OP2 DNAJC6 ENTPD1 SMPX MPPED2 HAS2 CDPF1 LYSMD3 VPS53 ENPP4 PAPD7 SYNGR3 FYB LYRM1 IL13RA2 SRSF3 RHOG PITX2 PDLIM7 PSMB1 SLC41A2 GTF2E2 SYT12 ANO5 TLR4 WASL ANKRD40 SMIM5 LOC770548 XIAP SLC7A9 CMPK1 SMIM8 CYP4V2 PLAU CIR1 LOC395159 RABGAP1L ZFP92 C10H15ORF59 RREB1 7-Sep RAB33B POR PBX3 HPGDS LOC396479 CDC27 OCX36 RHNO1 SHOC2 CETN2 RGS19 LDLRAD4 GEMIN2 FAM222B DNAL4 SIX1 DNAJB9 ZNF512B FAM49A C4BPA ARR3 NDUFAF2 RSL24D1 TNNT3 RFFL STAT5B EIF3I SERINC3 SNRNP200 CDC25A CLK2 CUL2 OTUD6B RNF111 AMY1A 6-Sep NDEL1 MAP3K14 DPP4 VCAN ALG12 C14H17ORF103 MTMR3 STAU1 PARD3 FLNB FBXL21 MFI2 LCLAT1 CDX1 RAB8A LGI2 F2 CRCP PSMA6 C11H19ORF40 MYL4 N4BP2L2 FBXO34 NDUFV3 NCALD FAM213A FKBP8 PHKB ARHGAP19 PTGR1 CCDC61 ELMO3 ASB7 NHLH1 AATF SRPR PUF60 RLBP1 TSSC1 AIDA ID2 MYOZ2 EXT2 FUNDC1 ADAM9 KDSR RGL1  RP11-20I23.1 TBL1X LMBRD2 KIAA1191 ARL6IP5 RP11-403P17.5 TTL CRADD DCK PPM1B USP45 TAF7 WSB1 CACNA1D RTN4 NRF1 GALNT1 LUZP2 TXNDC5 SSTR2 CLTA GJB1 FAM114A1 TEX264 ABCA1 GID8 C20H20ORF24 PRTG GPR171 EVL ACSL1 NTRK2 PTDSS1 NKAP SH3BP5 ZCCHC17 FHL5 MTX3 NR3C2 AVEN GOSR1 EXD2 STARD4 PRIMPOL KDR HIBCH CALM1 WDR18 FANCC DNAJB12 RHOA IL5RA PRKAR1A FLT4 ST6GAL1 TCF15 GHRH CHST3 HESX1 SNN YWHAZ IRX4 P2RY14 G0S2 ARFGAP3 GHITM CDCA7L EXOC3 FZD5 TNNI2 VWA9 STUB1 JMJD4 HNRNPH3 TCP11L2 PLEKHB2 MGAT3 CANT1 BAZ2B SERPINI1 MAGI3 CSRP2BP NPAS2 MAPRE2 HMGCS1 RBM25 ADPRH BSG ANK3 LOXL2 TMED5 TMX4 FOXD1 MTMR9 TNS3 PTGS2 NUDC OPN5L1 LOC416354 MFN1 HIC2 ASNSD1 VPS18 RARS2 ANKRA2 ZYX CSNK1E GLRX LOC420419 TNFRSF19 DKC1 BCL2L1 YWHAB PARK7 CFLAR MAP6 TMEM170A NRTN C7H2ORF76 SLC35B1 RBM5 STK17A ATF7IP LPGAT1 CNGA3 PIK3CB PPME1 PKIG NRG4 LIMD2 PNRC2 HYAL6 CITED4 EFHD1 RORA SOCS3 CDV3 POPDC2 NCLN LOC420849 TRIB2 GMFB MAOA MYOM2 SLC16A8 DPH1 KTN1 NFKBIZ ZDHHC17 CTNNBIP1 RGS17 EGFL7 LOC424740 LOC396531 MTMR2 EPGN TYRO3 TEAD4 WBP4 CAV3 CMTM7 TMEM229B IL16 RASSF5 ODC1 ALKBH3 MMP10 RINT1 GDPD5 IMPG1 ENOX2 CKMT2 TBX22 CCNE1 CTSB ZFYVE21 ST6GALNAC2 FAM105A AMIGO2 ITGA1 OVAL LCP1 SPG21 ASH2L SGMS2 TACC3 LOC427470 PLAG1 TTPAL UBLCP1 CDH5 NR5A1 TMEM184B ENO1 SEC22B APOV1 HDAC4 SLC16A1 MRPL38 PGR BRSK2 SRSF1 SRGAP1 RCHY1 CNTRL CLDN1 RNF13 ADD1 PLS3 BTF3L4 MATR3 LOC422426 ENTPD6 EEF1A2 KLHL13 P2RY1 KCTD9 DDX6 AXIN1 MGLL UBE3C GATA2 TACSTD2 BRINP1 CD200R1L CAMK2D ESR2 ORC5 TIAM2 FLI1 C3H6ORF120 KDELR2 CPA6 BTBD9 ADCK3 SOCS2 LIN9 ANKRD27 ALDH1A1 TADA2A LOC396224 LYVE1 PTPN11 ST3GAL3 BBS4 DNAJC12 LGALSL CHRDL1 PSPC1 MBLAC2 ASIP ERGIC2 FBXW11 TERF1 FHIT SCYL2 INO80 S100Z SLC26A5 BORCS5 CCR5 AKT1 C4H4ORF29 BASP1 TSSC4 LGALS3 LFNG STRAP 8-Mar DYNC1I2 LOC415756 SLIT1 APBB1IP APCDD1 CNTN2 JUP TYR SLIT2 FOCAD MOB4 COL18A1 DLX6 GPR89B RAB40B ACOT9 MCFD2 P4HA2 MXI1 SOX14 PRKAA2 PHAX CHD1 DHCR24 SPP1 EDN2 C9ORF69 EIF4G2 SSR2 GNB1 SLC19A1 TNFAIP6 B3GAT2 CCNA2 ELF1 ZNF767 CRTAP KPNA1 CMKLR1 GNG2 YBX1 C1H2ORF49 IKBKB BMP10 CTNNA2 FAM118B SRSF7 EDC3 GAPVD1 CANX DMTF1 TP53I11 MAPK6 PDPK1 CHRM3 EDA2R KPNA2 MSN GNPTG PDGFRA GTF3C5 RECQL SLC20A2 NGEF MAPKAP1 DNAJB6 ILK DAPP1 SPPL2A PYROXD1 CELF2 ZFAND5 INCENP STRBP ZNF639 SYNCRIP LOC421975 UGT8 PCMTD1 PHTF2 RPL6 IRAK4 MRPL20 MYH11 HMG20A GLP2R ERBB3 FSHB CCNC STAT4 INTS2 ACVR1 CSNK1D A4GALT TXNRD1 CELF1 EMP1 KCND2 FAM20B MINA SET VSX2 SLC47A1 SUMO3 CD44 COL22A1 GTDC1 RB1 NFASC CTNNA3 STX6 SLC38A4 ORAI2 DDA1 SMIM18 OGN PTP4A1 KDM5B RLIM IMMP1L MORC3 ELOVL6 NBL1 LYPLA2 CACNA2D1 TAF3 FAM18B1 ITGB2 HHATL  RP3-461F17.3 PLEKHB1 COL8A1 RP5-1021I20.4 EPB41 TASP1 FUT9 CNOT2 AMPD3 PYCR2 NME1 KCTD2 KIF5C COX15 PCGF5 DAZAP1 CNP SDCBP CNR1 GREM1 ARHGDIA LXN TMEM56 ACLY RNF141 CHD2 KIF23 DTYMK ORC3 RGS9BP TPH1 TOLLIP PPP1R7 SLC47A2 LYRM9 PPP2R5C NREP CD247 WDR45B TRPM7 PLEKHO1 RP1-309K20.6 CD93 PPP1R2 KIF3B SLU7 TMEM104 ATM IL6ST ADIRF LMBRD1 HDAC9 RBBP4 RALGAPB N4BP3 GIGYF2 GPR39 C1GALT1 SGK196 MTMR8 SCIN CLOCK TBL1XR1 FGFBP2 COX16 MAP7 SALL1 NAPB ANKRD26 FAM45A IL8L2 SYNPR H2AFY RMND5A TPST2 NAV3 CTGF TRIM55 SLC17A5 MPC1 MAL2 NEMP2 PACSIN2 GNB1L CD151 RBBP7 SERPINE2 PCM1 SCYL3 LPP EYA4 MGAT4A WWP2 OSTN NUP50 GAD1 SPATS2L PPP1R21 ASB9 PMS1 PDE6H TCF3 FASN CLIP1 GATAD2A OVALY HIPK3 ZP2 NUCKS1 YRK COX6C ERH TEAD1 DTWD2 KLHL18 C1H12ORF73 CISD1 LIFR ABCE1 JMJD1C TWF1 ABCC1 SUPT3H BET1L SLC37A3 AGO4 TGFBR3 SDR42E2 GFPT1 RNF185 TBC1D1 PPP6R3 SERPINH1 APOD GUCA1A CBFB BPGM BORA PODXL TSPAN12 CCDC127 KCNMB1 STIM1 NELFA CLN8 FAM183A WFDC1 C5H11ORF74 ANXA7 SLC39A13 SAMSN1 CPSF6 CHP1 BHLHE40 UBN1 SLC35A3 UQCC NT5C3B PDGFB HMHA1 PDZK1IP1 SLC38A2 SKP2 FBXL16 PYGB ANGPTL2 STK17B WDSUB1 CALD1 STX17 SENP8 SMARCE1 SKIV2L2 PRORSD1 HSD11B1L ZCRB1 GLOD4 PBRM1 ANXA11 SYK KLHL20 FAM172A SMIM12 AKAP9 OSBPL2 TNC PIK3R5 KCTD7 PDGFA SZRD1 CYB5B CDH17 PUM1 DNAJC5 UBE2G1 LPAR4 DNAJC15 RABL3 NUDT7 SPTSSB GPR174 GET4 LSM3 SNX12 BCL10 P2RX5 KLHL24 GEM CREM TNFRSF1A C1H7ORF60 GJA4 CREG1 GPR143 H1F0 ELOVL5 SEPT2L PELI1 TLR1B LHX1 SFTPA1 SNAP91 POU4F3 RCAN3 ATP6V0E1 NINJ1 ACADSB ATP6V0B C13H5ORF15 TEC ESRP2 GLT1D1 CAT TAF12 MBP FSHR ALOX5AP |
| gga-miR-128-1-5p | ZNF767 SGK3 FAM222B DMD CANT1 TMEM123 CTGF RBFA PMP2 POT1 RAC2 COPB1 SERPINI1 MEAF6 GSTO1 HESX1 MST4 SSRP1 CCDC93 BPGM SNAP91 BIRC2 TBCA RBM25 SPTSSA BLCAP LAPTM4A NFIB STRA6 GATM CAV3 RAP1GAP2 TGFBR2 CYP11A1 SLC16A1 OLIG2 LYRM4 ASB8 TAF2 CDKN1A N6AMT2 ZDHHC18 GBGT1 ERI1 CRCP TCF15 ZDHHC8 SEPSECS RDH10 EPS15 TARSL2 SPRYD7 ZFYVE1 LOC396365 RRP1B DCAF7 UBXN2A SZRD1 SLC20A2 NCK2 TNNI2 TNS3 SLC19A1 TNFAIP6 CDH2 SDK1 GHITM CNIH1 NUDT5 SLC22A7 TMOD4 ATAD5 ELF1 SPG21 APBB1IP FZR1 NDUFS1 RPAP3 UQCC STX18 FAM46C UBE2K HHATL TBX4 IL8L1 MTO1 USO1 FAM126A CHEK2 CPA6 RALGPS2 PBX3 TMEM41B COX14 ESR2 ENTPD6 USP48 C1H2ORF49 LOC100859427 NPFFR1 CCNE2 DLX6 RASA3 MYL9 RAB19 ST3GAL1 CDC42SE2 PUF60 CXCR1 TOX3 SLC38A2 XPA SAMHD1 RHOT2 ALCAM BID FBXO7 BRE MAPK6 CLSPN BIVM RP11-834C11.12 PSEN2 CELF2 USMG5 UGGT2 SMC2 SLC6A9 FUNDC1 TM2D3 NKAP MED24 ARHGAP26 RBM12B LCP1 CSRP3 HNRNPA2B1 P2RY14 GLT8D1 SLC35A3 EPGN PLAG1 ARFGAP1 H1F0 MED27 ZNF335 RRM1 NFAT5 COMMD7 MGAT4C TMEM237 DOLPP1 LSM6 EIF2AK2 GORASP1 FGL2 HK1 SCG3 RAP2C WDR92 SLC8A3 PAX7 ACAP2 TAF7 AARS2 ACVR1 GATA4 SNX10 CNTRL RFC2 HMGCS1 PLA2R1 TRAPPC3 IL18R1 HIBCH ZBTB2 SDR42E2 OTX2 FANCC UTS2B STUB1 TAOK3 NPRL2 KRIT1 IRF2 KPNA4 IFNGR2 ZNF800 DEAF1 NREP SSTR2 LRTOMT FOCAD LXN CLIP1 UBN1 NFRKB DOLK RBL2 CLEC3A GLUL B4GALT7 CIAPIN1 ALAD CYB5R3 NDEL1 ATP6V0E1 POLD3 SPTSSB HHEX HDAC3 TTC7B PANK4 MYOZ2 QRICH1 P2RX5 TNNT3 PCGF5 BRCA2 PPP1R2 AHCTF1 IL16 HNRNPDL CASP18 SRP68 ORC5 PRPF19 BZFP1 FAM173A GDF3 PHF21A SEMA3D CRYBB3 COL18A1 LAMTOR5 GGA3 PIP4K2A C5H15ORF41 MPC1 DGKZ YWHAZ 15-Sep CLU SLC48A1 PDLIM7 SYNCRIP SLC25A36 RFC4 TMEM229B ILK PRLR SOX10 BCL10 CSPG5 BLOC1S6 POLK KATNBL1 XBP1 PRKAA1 PAX9 KPNA3 ARHGAP25 COL8A1 SAT1 IGFBP4 STRBP AVD FAM45A FHIT MRPL48 ZFP92 WBSCR22 SOCS2 SLIT2 ELMO3 MESDC2 XPO1 DUSP4 ADARB1 CD24 CTNNBIP1 ST7L INTS2 GCM1 HDAC2 SH3BP5 GTF2H1 LOC772096 RAN SLCO4A1 S100A10 TRMU LOC426914 PDS5B DIO1 VDAC2 UBAP1L VGLL4 SZT2 HMHA1 HCCS ST3GAL3 RAB18 ALDH3A2 RPRD2 F2 PRIMPOL CD93 RPL6 ARHGDIB SDC2 DAD1 CBLN2 CCNL2 PARPBP MOV10 CDH6 PDGFB HSPA9 TCF3 EXOC6 GNG13 MXI1 BIN1 EEF2 HOXB3 NRXN1 RASA4 DUT DDX27 HNRNPH1 MAOA VPS4B HOPX ANXA1 PIP5K1A EFTUD2 PHLDA2 DCK CNBP PEX10 CCDC50 IARS2 SH3KBP1 PDS5A ARIH2 SGMS2 ACE PRR5 RTKN2 SERPINB10 TP53INP1 DEK CYP51A1 ADAM17 RFNG MASP1 FBXL12 ABHD12 ATP6V1D ATP5B PRKAB2 ADAM9 PPAT ZCCHC6 AP3S1 LSM5 TMEM56 ZDHHC5 MAFK NDUFA12 IL6ST LGALS3 TSPAN13 FBXL21 DAZAP1 PHKG1 ABCF2 RNF123 EREG LOC396531 HIC1 KLHL24 FYTTD1 DNAJB12 IVD KBTBD4 RABL2B DAPP1 LOC422926 DCTD C13H5ORF15 APOD CMC2 MAPKAP1 DPF3 MMADHC PLCXD1 LOC395647 MCCC1 CACNG1 CNOT7 NRG4 MTHFD2 IRF4 COL14A1 MYLK2 ARFIP1 TADA1 C15H12ORF49 ACTR2 KNSTRN ADCK3 MSMO1 PRELID1 PSME4 THOC3 ADIRF CYB5R2 TBX22 CLPTM1L LOC418424 RAX RIPK2 TBL1XR1 ASAH1 GLIPR1L CCDC101 TBL3 ITGB5 FAT3 TMED10 KHDRBS1 ACBD6 ABCC6 LCLAT1 CSNK1E UBN2 ANKRD16 CNR1 SH3BP2 CCNE1 TYW5 RARRES2 TADA2A ACTG1 ABHD17B FABP6 GNG2 IL10RA B4GALT6 SUPT3H FARSA CDH5 RASSF2 TNFRSF13C EI24 DDOST TMEM208 KRT5 ANK3 STAU2 CHD1 NME3 PRRC1 AKTIP POLDIP3 UBA52 LOC100858447 TSPAN1 FOLR1 FAM214A BMP15 STK32A CEP63 PTRH2 ACSBG2 FAM105A COPS4 CNPPD1 VAMP7 CTD-2410N18.5 RECQL SLC16A3 HSD17B1 HYKK LFNG JUN VIMP UNC5B MBL2 NUDT1 CDH7 MAL2 UGDH CMTM3 C3H6ORF203 RAD51 MAFA CBLN4 CUL2 IGF2 MFN1 TMX4 LOC100859756 POPDC2 COA7 SYNM IFT81 CYB5A AGPAT9 LECT1 PARS2 FMOD ICMT STX17 KLHDC4 IRF1 JUP HMGCR PKM MYOD1 EPHB6 SPATS2L HES5 KLF11 CALM1 MGME1 N4BP2L2 COL4A1 SERPINB6 ARMC1 SUB1 PCBD1 IL5RA RPP38 CLDND1 DNAJC16 NEK7 DHRS3 JMJD4 CHM ZNF639 SGCB CAB39L PNAT3 TM4SF1 5-Sep CD200R1L WDR24 C11H16ORF70 BAK1 VEGFA VNN1 DCLRE1B RNF4 CISD1 RNF7 FKBP8 WDR43 AATF TEX264 ADAL TUBB TRIM37 EIF4G2 ID2 WWP2 TUBA3E CD72 MPST SLC46A3 GPR149 HNRPK TIMM8A HPCAL1 NDUFB1 E2F5 ATP1A1 PLEKHM1 SHFM1 FAR1 MBLAC2 CRYBA1 UCK2 KIF18B RNH1 PRPH2 CRYAA NHLH2 PDLIM5 TRAF7 ADA CLDN3 RGS19 HAGH GJA1 RGN MCM6 SLC35B1 ZFAND5 YAF2 DUSP10 PDK3 C1GALT1 GLRX3 NR2E1 P2RX1 GIP CDCA7L KDELR2 CCND3 TERF1 SERINC2 CTSA CEBPB IRG1 ZCRB1 ZIC1 NDUFB2 MAT1A PPME1 ELMOD2 F13A1 CASQ2 CDIP1 IPPK TECTA RPS4X SPINZ POU2AF1 FSHB TCEANC2 YEATS4 RALBP1 GYPC PCMTD1 JMJD6 COL2A1 XIAP RNF152 CD72AG ARL6IP5 ARHGAP21 TGFBR3 AGA C12ORF57 ENTPD8 TRIM71 WBP4 NRBP1 EHMT1 TIAM2 VTN NUDT19 TP53I11 TMEM70 TRAF5 TAGLN TMEM104 CYP1A1 RNF141 PPP1R21 DMTF1 TOMM7 SLC17A5 CCDC6 GJC1 HNRNPLL INHBA PDGFD ALYREF KERA MBD4 WFDC1 BSX CCL17 OPN1LW MYH10 MFAP3 TXLNB SNN TBC1D22A RPS14 RUFY1 HSD11B1L TSEN2 TAF1A PPIL3 GPR174 TSPAN6 ADCY5 FBXL16 RUNDC3B CDH20 LOC100858381 SMARCE1 BTF3L4 SFTPA1 DHCR24 LOXL2 RPL31 SEC31A DEXI ANG SPPL2A PLOD1 ZDHHC21 MPP1 YWHAE EXD2 HSDL1 CLDN1 MARCKSL1 N4BP3 CD1B RECQL5 NFASC FHL5 CSNK1D MRPS6 BOK HSPD1 AMIGO2 COL9A1 NT5C3B CHCHD4 AK6 ZFAND6 TMEM138 RAB8A UBLCP1 DNAL4 AC113404.1 KLHDC2 SMPDL3B EXOSC2 KCTD7 ERICH1 FASN ALDH1A3 PDGFC CRIPT EEF1A1 DDX5 KIAA0907 RABL3 RBM19 BRD8 MSRB1 SHISA2 THRSPB MAP2K1 CLINT1 AANAT NME1 SIGIRR SOX11 MYL12A C1ORF95 CDC42 PSMD14 ID4 LHFPL5 TPRA1 RABGAP1L PTPRO AMY1A SLC17A9 CLEC3B CECR1 ATOX1 ATP1B1 SLN MTHFD1 DESI2 PPP2CB NFKB1 GNB1 STARD4 NEK6 FOXM1 MTMR9 CTSS COPE HYOU1 AHSA2 NECAB3 CEBPG RP11-5A19.5 ELAVL1 ALKBH3 MAP2K5 IHH GPX7 NFIC PYGB MAP2K2 RPRD1A HERC2 BTD RP11-87C12.2 PRLH CENPO SLC7A6OS UBE2G2 NR1H4 UBXN2B HYAL6 PAPD7 NELFA NCLN CDH1 IGF2BP1 MCFD2 WRB UBE3B PRTG VCAN ASNS YRK TMED5 DYM ACACA PLA2G7 AICDA HABP2 GFRA2 RAB5A PRKAA2 TCEB1 G0S2 HAUS6 GMPR TEC CUX1 DMA TPST2 CRABP1 ZP2 ANKRA2 APEH MYH1B CLN5 MBNL3 CBX3 SPPL2B FAM18B1 NTMT1 DUSP6 CYTH1 TYRP1 RPL29 AvBD8 CATH1 FAM133B SLC35E3 TBC1D23 SFR1 SERINC1 RFFL GATAD2A LRIG3 FBXW5 MAD2L2 THAP5 LSM8 LOC770548 ST13 PLCZ1 APCDD1 PHOSPHO1 LOC421975 RPRD1B TUBB4B TIPIN MVB12B DCBLD2 PDPK1 SYK NCALD CEP112 PTTG1 TBCD UBE2R2 MSRB3 PIAS2 NEUROG1 PDGFA DNAJB9 KCNAB1 EXOC2 ARL14EP SRSF11 FOXN2 PLIN1 ANKRD26 C1QTNF2 CLDN5 ARR3 RPL32 TMEM141 GLRX5 PPP1R3E PKIG DHCR7 GET4 PEX2 TXN2 PDLIM4 VCP INPP5K RGS6 PRDX1 C15H12ORF65 CD3E TRH ARID4A TARDBP TMEM121 NDUFC2 FOXC2 PBX1 ETV5 KLHL15 COL6A2 TRAFD1 SOCS3 PNRC1 XCL1 VGLL2 POLR2L TMEM167A RBPMS2 DDA1 RASGEF1C GRIA2 LLPH EFTUD1 BTBD1 EIF4A3 RCHY1 TNFAIP8L1 POU1F1 COX4I1 MAGI3 C12H3ORF37 TRIB2 RSL24D1 SLC9A2 GNGT2 LOC426218 CDA GNLY CACNA1B LYN STK24 PTK7 CISH ATP6V0C DRG2 RP11-403P17.5 MORC3 DNA2 MFGE8 ITGB1BP1 STK17B PREPL DYNLT3 IPO13 TAF11 PITX2 APOA1 TMEM254 TUBGCP2 TCP11L2 BCS1L UBXN10 GPRIN2 PTDSS1 SERHL2 SLC2A2 PPP1R8 LBX3 STIM1 ING4 GFPT1 POLR3H RNF11 SERTAD2 ADCK1 MUM1 ELOVL6 CINP GABRG2 PDE4B NDUFA5 TRAPPC11 ZBTB17 TLN1 RPL35 MAPRE2 GCC1 MDGA1 GLP2R TRIM8 SELO THBS1 BORA FBXO9 RCAN3 CNOT1 FAM114A1 CRH DSTN NFYA NGEF LOC431499 FGF19 DENR FAM53A MMP10 SLC39A9 MYO1C RLBP1 PCGF2 MBP HMG20A INSIG2 LOC100858504 SRSF5A DHX15 WNT8A TOMM6 USP6NL IRAK2 LIN7B COMMD4 FZD4 LYSMD3 VPS41 GUCA1B PDP1 CEP41 LGALSL CDC25A PPM1B ROR1 STK40 SDR16C5 ZBTB26 SNX3 ATP6V0E2 C1H11ORF54 GJB1 TPT1 COX7A2L SEPT2L STK17A SLC34A2 CCDC18 ZNF326 RANGAP1 AARS ATP2A2 RGS9BP NEMP2 EIF2B3 PRKCI LRRC28 RAB3GAP2 FOXO1 SSB ARHGDIA CSNK2A1 FECH GSKIP PEX7 RAD54L VSX2 LOC100502566 HNRNPD TNFRSF19 FEN1 CAPN1 MMD ANAPC10 TRIP13 EFHD1 MYH1A GJA5 UBE2G1 SLC1A2 AGO4 NECAP2 PLEKHJ1 VPS37C GLCCI1 PEMT SRP14 MBNL1 CHADL IMPG1 NUP93 CYP46A1 STRA13 PDK1 GDI2 NFE2L1 BSG API5 CBFB CREB1 LPGAT1 CKAP5 HOXB1 BNIP3L APPBP2 CCNK PTGER3 PYGL PQLC2 CXCR7 SLCO2A1 MTIF2 SLC26A6 SUV39H2 YBX3 PTGS2 PXN SCOC RP11-514O12.4 F-KER PLEKHB1 ERCC3 COG4 TMEM175 YARS AC005943.2 ISLR2 P4HA1 DKC1 CERK ADIPOQ DNMT1 ADORA2B RAB3IL1 LOC427470 TMEM65 WASH1 PHF5A TWF2 ABHD17C SUMO1 POLH LOC396009 ACBD3 ZYX DAP PTPRZ1 UBA2 CPNE1 SPP1 CCR8 E2F4 IKZF5 LIMK1 SCNN1A CDKN2A MFAP1 DHRS11 PEX5 S100Z OSTM1 TACC3 PCYT2 AR LHX1 LSM3 KCNH6 SLC40A1 TAF5 CTNNA2 EEF1A2 TOB1 SLC16A7 DRAXIN CDKN1B OCX36 NBL1 MRPS26 DCUN1D1 CLN6 SLC30A6 FTSJ3 STAT1 SEMA7A PSME3 COPS7A OXCT1 RAD17 GPR146 MIP BRAP RNF111 RAD52 FAM118B PLEKHO1 NEDD1 CDKN2B MEOX1 SASS6 MALL EIF1 DNM1L HDAC11 ESRP2 GBAS SUPT20H NKX3-2 EARS2 NKX2-6 SOX17 DGCR6 OVALX GNG5 DDX42 RB1 BUB3 TMEM5 ELMO1 SGK1 MTMR2 ZCCHC17 RBM38 RPSA CDK6 BBS2 CLCN7 EGFL7 YIPF5 STK11 IP6K2 BTBD10 DYNLL2 PTPN1 NRF1 ZFHX3 GRK4 ABHD13 SETD6 PAIP2 TSN RAD21 SYPL1 ARL2BP GCLM SPP2 CBL OAT TMEM230 EPHA7 OPN5L1 COL9A2 CD82 EPHA5 CSRP2 GBE MET PSMA3 FZD5 LOC395991 TMEM180 NR1H3 ACVR2B CHST3 BHLHE22 CNGA3 RRN3 TXN CETN2 POLE3 PSEN1 SALL1 KANK1 ENTPD1 USP7 NAPEPLD AKT1 RP1-309K20.6 ATG9A NRG1 PIK3CA TMED3 CPT1A PPDPF DCAF13 GALNT1 TMEM68 CEPT1 NBN HMGB2 RRP7A CAMKMT PPP1R16B CERS1 TBC1D15 PHTF2 KIF1BP FTH1 METAP1 PLK1 TMEM258 FADS2 NFE2L2 CTDSPL2 GHRH CLSTN1 FUT9 PCK1 PMP22 OVAL DHX38 RALGAPA1 KLHL20 DDT PLEK2 PLEKHF2 RAB33B MYBPC3 MOXD1 LAMTOR3 LGI2 ZC3H6 PFN2 ZNF706 CD276 CRYBB2 ECE1 TRPC4AP GFRA1 MIB2 KRT6A BCL6 USP37 AP2M1 PIGA YTHDC1 MTSS1 TMEM189 TRABD CRYAB ST6GALNAC1 BRINP1 PSMD1 TCP11L1 HPGDS RP11-195F19.29 GATA3 DUSP1 RIC8A CLTB ACTR3 PLK1S1 FLOT2 HAVCR1 ATE1 ORAI2 RNF220 RBM48 CD151 NCOA4 CIRBP SNAP23 CXCL14 SLC26A5 RALGAPB AKAP17A HAGHL RHOT1 NINJ1 DOHH SMIM18 NAV3 ZW10 B3GAT1 AC025048.1 LOC100858797 PLN FGF1 WNT2B CYP24A1 BRSK2 NCF4 RBP1 SSR2 CHST10 JDP2 TAX1BP1 TPH1 XIRP1 SNCG CD320 PSPH AAR2 TMEM136-1 ASIP LMBRD2 FBXW11 PPHLN1 SLC25A15 STAT5B RTN4 LMX1A SCLY ULK3 AZIN1 GEM TNNT2 NOS2 IGF2BP3 MPZL3 EPYC GCHFR CD200 CTSB IFRD1 ASF1A SERPINE2 ANXA2 DHFR ANP32B CHMP7 CCKAR NKX2-2 TXNRD1 KDSR RAB10 TLR21 FK27 LOC100859148 TMOD3 CTCF CIR1 TMEM57 LDLRAD4 FIBIN NRP1 RASGRP3 GTF3C5 SPI1 ANP32E TBC1D1 SLC9A3R1 ACTN4 BEND7 STX6 TGFBR1 PELI1 PDE6H ARPC1A MTPN ENOX2 LPAR2 UBE2F LOC431316 FOXD1 CLDN2 SERPINA1 FAM20B BRD2 CTD-2116N17.1 ST6GALNAC4 SPATA2 RMI1 PRKAB1 KIF2A PSMD3 RBM12 SLC47A2 NUP58 MYOM2 GOT1 FBXO8 RAB28 BORCS7 NSMCE2 TRIM27.1 TNFSF10 TTC27 RPS2 LMO2 ADSL CPA5 LOC772071 CTPS2 COX17 FXYD6 TBK1 ERBB3 OPTC TMEM173 PIGY METTL22 MGEA5 CLK2 SMPX RASSF5 RAB9A DNMT3B AMD1 EIF4A2 C7ORF73 GDPD5 ENSA DNAJC2 MAP7 MORN4 C7H2ORF69 PODXL LOC769139 RPLP0 MAFF FKBP4 SMIM20 CCK PLEKHA3 NQO1 TDRD7 ACTB SPHKAP SPTY2D1 KDR RRAGC HMGA1 PNPLA2 LOC431325 PSMA4 YY1 BCAP29 PPP2R5C HIPK3 PSMC3 PARD3 PDCD6IP CL2 SATB1 ACAD8 KCNMB1 APOA4 USP15 ACAA2 PTPRC TAF1B RER1 CRADD BMPER CAMK2D CCDC61 PLEKHA2 NEIL2 MKKS SLC35C2 CD28 CHUNK-1 EPRS YIPF1 C2ORF88 ERGIC2 ARPC4 PIGK MTMR3 RGS20 TULP1 APELA MRPL38 CCR5 REL ALDOC NPTN GAS2 PARD6B ACTR5 HDAC7 ZNF384 PRKRIP1 RIPK1 PCSK7 GABRB3 SGTA RFXANK YPEL5 SIX6 GJC2 FSTL1 SEPP1 CSDE1 PAK1IP1 VDR TWIST2 NTRK2 CELA1 GLRX IL6RA MELK ARMC7 WIPF1 C1S AP4B1 ITPK1 LOC100859249 SIRT5 KIF3B VAV3 ST3GAL2 WRAP73 MID1IP1 P2RX4 PPP1R12B MPPED2 KDELR3 CDK10 CHMP2A LOC100859616 LMBR1 NCDN HEP21 COL22A1 TMEM164 HDAC9 WWC1 TXNRD3 IDUA PCMT1L PGA B3GNT2 PHKB TMEM55A MRPS11 TBX6 EYA4 FURIN ALDH4A1 CKB PIK3R5 SMIM12 C1D LOC100857840 VIT BAP1 PSKH1 RAE1 RPS19BP1 LCMT1 BHLHE40 CACNA1S GIGYF2 HVCN1 ELN MLF2 P2RY1 B4GALT2 HIF1A VPS35 RNASEH2B SRSF5 SRPR ZC3H15 MRPL34 MYD88 BAG5 KCNMB4 WDR91 IRF9 ANKRD40 DENND6A RPS12 TLDC1 TM4SF19 TTL CCDC28B PTPN11 TSKU FGF14 LOC417192 ZPBP2 CPSF6 EGFL6 WDR82 TTPAL DTWD2 COL6A3 PAQR8 KIAA1467 VPS45 THOC5 KLHL2 SAP130 KRTAP10-4 DCAF12 KCNA4 SPTLC2 VMO1 CMTM7 MAFB FANCL IMMP1L ACP2 LRRK2 SDSL CRIM1 KPNA1 TEAD1 COL5A1 NUMB SYNGR3 AMT ASH2L NTSR1 FDFT1 MRPL20 CHMP6 LIN52 ATP1B3 FBXO34 SP5 CLOCK HES4 WEE1 CIDEC CCDC127 PARK7 PIGR MAP3K14 T HNMT LOC420849 NUF2 SNCB KIF20A TWSG1 FMO3 MYH11 STX7 GUCA1A TACR1 GANC EIF5 YWHAQ SBNO1 FGFRL1 ARHGEF3 RP11-290H9.2 TRIM39.2 NCAM1 LIFR ARC CITED4 FOXA2 SMIM19 BET1L C20H20ORF24 SNRNP40 C4H4ORF29 BCL11A ANGEL1 IFI6 STAT3 DCN VDAC1 ARF1 TNC PTPRG GNAQ THYN1 MRPS17 C1H7ORF60 PIK3CB SERPINA4 ATP2A3 VWA9 MBD3 CRYL1 MB PPP2R2D ST6GALNAC2 LOC426385 TMEM88B HSBP1 CDH17 WASL ALC AREG GPR126 LPAR4 PLAU PCNP RGS16 IGHMBP2 HNF4beta ABLIM1 VPS53 HARS NPL FGB NDC80 ROMO1 SLMO2 PPARD ANGPTL2 SLC47A1 MGAT3 MOB4 AGO3 STX2 COL1A2 CD74 KPNA2 SPCS1 C3H6ORF154 HSBP1L1 CHRDL1 PPP1CB NUDC RBP5 CETP OTUD6B PCMT1 RP4-613B23.5 RAG2 SULT1B1 QARS TSPAN3 TOP3A AP2A2 BTC CD38 IK HSP25 TAF8 EXOC5 AHCYL1 C14ORF2 INSIG1 HCLS1 TOB2 GAD1 CHORDC1 ANKRD52 FAM192A ZSWIM7 VRK1 RNF13 CNOT2 NR3C2 RELL1 CD99 MAP6 CD86 ARIH1 ITGAV FLI1 PABPC1 LYPLA2 ARHGAP40 RAB22A 8-Mar NRN1 GPX2 PTN CREBL2 SIKE1 DZANK1 CBX4 INO80 NUP107 CBFA2T2 ATP6V0A4 RBBP4 WIF1 MZT1 KPNA6 BRCA1 NKIRAS2 AIDA DAZL TJP2 WDR1 CRTAP RBMX NGF FAM102A ASB9 CHMP2B HRAS FZD7 THRSP PRC1 COL6A1 TYRO3 PBLD RAD51D MIF TMEM120B ARNTL WSB1 C28H19ORF10 LOC420419 EFNB1 ODF2 GXYLT1 AAMDC DCTN1 GPR39 HIC2 P4HA2 MRPL50 PTPN6 NAPB MSANTD2 RNF34 GLP1R KLHL14 TNFRSF18 USP45 ARID3B LOC428335 MAPT RIT1 RPL34 CCDC167 PARP1 RAF1 CNOT8 DDX6 RHOB PEX13 PRRG1 TFIP11 SARS BLOC1S5 PROKR2 GJA4 ATP6V0D2 RREB1 HOOK1 RHOA SLC12A7 CMKLR1 DYNC1I2 NRBF2 MGLL C1H12ORF23 SMIM8 OVALY OLFM1 RAB35 PDGFRA SNX16 ACLY ALDH1A2 CCNC LOC100859039 LOC419429 SNX13 NECAB1 KDM5B LOC416354 LYRM2 UGT8 ATP5I OSTN NF2 MEF2BNB GABPA COCH NDP ADPGK CBX1 LBH PMAIP1 JAK3 PTP4A1 C1GALT1C1 KIAA0020 EMP1 SEPHS1 CACNG3 AKR1B10 CNDP2 ANXA6 PPP2R4 PRPF3 GLOD4 ALKBH2 RP11-49K24.9 SULT1B LOC693265 PAK1 E2F6 SLC38A4 PRRX2 FAM76A GPM6A RGS17 SLU7 NR5A2 RPIA PMEL C1ORF146 FZD6 CETN1 GMNN PIAS1 TREM-B1 ATF4 CKMT2 EIF5B TRAPPC2 AP3M1 RNF185 SELK ENO1 HSP90B1 C5H15ORF57 C26H6ORF106 GNPTG LHX9 NFKBIZ MRPL41 PLIN4 GOSR1 P2RY6 SMIM15 TPM1 EDN2 PALM UBL3 FAM86A KCND2 ATM MRPS16 LRRC57 BCL2 RSFR LZIC HAO1 RNF14 MTMR8 SOX3 SDF4 RRAS2 PCM1 USP12-like APC2 TANK RFTN1 PAX6 PPFIBP1 CRY1 CD5 EMB CHRNA7 COPS2 PLIN2 APOC3 TMED8 DPM2 CRIP1 THY1 HSPA5 MDH1 BAZ2B CCNA2 BMP10 CNP MAGI2 FAM122A PIK3CD VPS18 SSU72 TLR4 DCLK1 K123 RP11-20I23.1 KATNB1 FABP5 RASSF3 STX16 LUZP2 NFIX EDA2R S100B TXNDC5 NR2C1 HS2ST1 RHOG CELF1 LOC420209 ARHGAP19 COX19 RAB14 CANX TMEM129 RAB32 CD80 TCF12 FZD1 HIAT1 COPG1 FETUB LYRM9 TSC22D3 GART FBLN5 G3BP1 OSBPL2 TMLHE IL13RA2 SERPINH1 ATIC MYL4 PAN2 SCAMP1 ZMYND19 LIMD2 SLC37A3 LOC770684 ACAN CHD2 NUDT7 MCL1 TMEM30A ASNSD1 RGS4 LOC422426 MPZL2 CCL19 NRGN RBM5 CTNNA3 FGF2 DNAJB14 MGST1 TICRR NONO NAA50 MATR3 MPP5 TSSC4 NCBP1 CACNA2D1 EIF5A2 INVS CDC26 CNTN5 KIAA1191 HMBOX1 NRAS DNAJC6 RP11-26J3.4 THPO MTX3 VPS50 IL1RN PTGES3 MRPS25 GLI2 PDDC1 BTN1A1 FAM49A SLX4IP GPR89B MXD4 UMPS DNTT ASCL1 HDAC4 COL17A1 MCM3 TRPV4 DIO3 HDX KLHL18 GNG11 YTHDF1 WWOX HACD3 ALG11 YWHAG GMFB MLNR ELOVL4 FBLN1 CPSF4 NPAS2 MDM1 CDPF1 ORC1 PRKAR1A SRF CYP2D6 CACFD1 SCG5 CTSK TMEM170A SYCE3 IFI27L2 KIAA1671 8-Sep BEAN1 PPIB AP3S2 SYNPR IBA57 ETS2 ATP6V0B EDC3 ACSL1 SLC7A9 DEPDC1B JAK1 HMGA2 PFKP E2F1 DDB2 XKR8 CHP1 SNX12 RGP1 SCPEP1 EIF6 IMPDH2 SIRT3 NT5C2 COPS5 C11ORF31 ANKMY2 GTF2E2 BUB1B RBMS1 DNAJB6 LOC776816 LMO4 MLPH FEM1B ST3GAL5 CLN8 TNFAIP1 C26H6ORF89 RPL9 FBXL18 CAMK2A C14H17ORF103 C5H11ORF96 ARRDC1 GNOT1 RDH5 MSX1 FYN ANO5 SMIM3 CHMP4B MICAL1 STAT4 WNT5A A4GALT BRPF1 C8H1ORF27 TAPT1 YPEL2 SUMO3 NAGA EYA2 ERH SEC11A CAPN3 LOC395926 C7H2ORF76 PNISR B3GAT2 IRX4 SNX6 MTERF3 NOTCH2 CABP2 JARID2 AvBD1 SNCA KTN1 CTNNB1 PCDHGC3 PTBP1 MRPS33 VAC14 STMN3 DYRK2 RP3-461F17.3 GAS2L3 UBL7 PKP4 PRNP INCENP SLIT1 BARX1 KIF23 EPN2 SOCS4 CSRP1 CREM ZDHHC17 PUM1 PSMG3 ZNF512B HADH TAPBPL OCM MMP9 NEURL1 ASB3 PPP4R2 MRPL44 OGG1 EDEM1 POU4F3 SLC35G2 XPO5 ITGB2 ITM2C GFI1B ADD1 RP11-101E3.5 FRS2 6-Sep TBL1X EDF1 UBIAD1 CMPK1 NSA2 CDX2 CASP2 RAX2 ELL LIN9 ASB7 HGF STK11IP UFSP2 DNASE1 POFUT1 NPC2 CDKN3 CDH11 BMP4 TTLL12 CD36 GOLGA7 ASXL2 FDX1 MEMO1 EMC1 ART4 MYBPH MYLIP UBE2V2 MSN DAGLB SLC25A22 GTF2H4 TRPM7 NTM CDC27 PHPT1 BTBD9 ANXA7 SYTL1 TMCO3 LARP4 MGAT4A TEF CHRM3 SCIN EIF1AY AVEN GATA2 WDFY2 SIX1 SLC41A2 ATF7IP MAP2K3 ID1 YIPF3 AKAP2 PECR ARFGAP2 GPATCH2 RRAD TPI1 HAS2 CD44 NDUFA4 LSG1 DEPDC6 SALL4 GSTT1 SUCLG1 SLC9A4 VDHAP RHOC IRF7 NAIF1 APTX HN1 CD247 GFOD2 LUC7L3 PMS1 MAF AK1 SLC39A13 PHC1 TBX5 MYH9 FIGF LEF1 TSTA3 MCAM PSMC1 STXBP3 LEPR MMACHC SVIP TMEM184B AAMP TUBAL3 NRTN ZFYVE19 IRAK4 PER3 YWHAB SRC CHAC1 C3H6ORF120 DR1 SOUL WDSUB1 NPPC ANXA11 ABCA1 ODC1 LOC415756 ESF1 CUEDC2 GPBP1L1 RGS18 APLP2 GAPVD1 DCT SETD3 LPP TMEM106B 5-Mar FGFR2 PPP3CB FYB MRPS7 VPS16 GID8 FZD3 GPR50 ESD PARN CRYBB1 DYL1 MRRF RORA PGR ALG12 PLP1 RPS6KB1 SMIM5 MED8 SOX1 SNRNP35 SORD SOCS5 CDV3 PBRM1 SRSF1 NDE1 CPSF2 RNF20 SETD4 TANGO2 BICC1 CSNK2A2 FAM60A RBP 9-Sep RP11-196G11.1 CSF3R CHERP RNP GSPT1 SPRY2 ELOVL1 TRIM59 KLHL13 TNS1 POPDC3 NAP1L4 C3H2ORF43 CLPX ASB6 MINA ETV6 NCOA1 CPT2 COX6C UCP3 TOP1 CCM2 QSER1 SCYL2 DPH1 ST6GAL1 LOC422214 CAV2 ENHO CDCA4 POLR2H UBE2V1 CENPF ITGB1 SGTB IL2RG DNAJC5 PPP2R2A ANKRD27 CACNA1D RHBG PACSIN3 VAV2 GABRA1 GNAT1 PTH CASP6 CFLAR LUM STOML1 NME5 RP5-1021I20.4 CUTC ANGPTL5 CAPZB DAAM1 EIF3L NCAN AvBD4 NR5A1 PNRC2 LOC424740 MTFR1 CCZ1 CNTN1 WDR5 EIF2S1 SNRNP200 ARFGAP3 BHLHE23 TFEB BORCS5 EXT2 PAFAH1B1 SET GALNT6 STK10 ZNF302 PACSIN2 FZD10 ALDH1A1 TOP2B KBP DIAPH1 MAVS RRP12 WDR18 TAL1 TSPAN15 GTDC1 TMEM131 CHUK ACSL5 SMIM7 ANGPT1L KCTD9 TSSC1 AGMAT NUP50 DBX2 SERBP1 SYT1 DCX MYST2 ABRACL KCTD2 FLT4 MLX ERLIN1 GNB5 GNB1L SEC23B C10H15ORF61 ACOT9 PDLIM3 MYEF2 GTF2A1 PDCD5 TPM2 NEUROG2 BBS4 FAM213A SGPL1 C11H19ORF12 ACBD5 ARL1 MXD1 NFIA SGK196 CPNE2 PHACTR1 FOS STRADA GTSE1 CSF3 C10H15ORF59 PDHA1 FBXW2 NOC2L MYF6 FAM175B KDM3A MAL SNRPD3 VPS39 NCAPH EPB41 GLT1D1 HP1BP3 PNPLA6 PIGBOS1 CDK1 SENP8 KIF5C SP1 GREM1 CRYZ GNAI1 NADK MRPL28 SRSF2 HOXB4 IL21R NSUN4 AMPD3 PAFAH1B2 EHD3 COMMD8 ENTPD2 NFIL3 CD81 LEPROT MAPRE1 NFKB2 PLEKHB2 STAR ACADSB TEAD4 ENS-1 SRGAP1 SYT12 GPALPP1 DFNA5 BCL2L1 MCTS1 SEC22B HDGFRP2 CHMP1B ASCC3 BASP1 PEX11G BLM ORC3 CENPH CD8B TSPO RLIM RAB40B CASP14 ZNF609 GPR171 SHOC2 AXIN1 ORMDL2 |
|  |  |
| gga-miR-133b | LPAR2 SUCLG1 PAAF1 EEF1A1 CTNNA2 SUB1 MAPK6 OPN1SW RAB9A PRKCI MGLL XPO5 ARMC1 7-Sep SLC2A2 ACVR1 GNG11 CHRM3 EPN2 VTN GATA4 FUNDC1 HBAA ACTN4 PEX5 MCMBP PROM1 MAP6 RRAGC LCLAT1 SPTY2D1 LIFR HIBCH DDT API5 CTSK SLC9A8 DAAM1 ZFAND6 CDA OPN5L1 CNP BNIP3L TP53INP1 BIVM CTNNB1 FAHD1 RBM12B COPB1 MYOCD PBX1 POLK FANCC CCZ1 TECTA HDLBP PLEKHB1 MBL2 DPM2 FUT9 GNB5 CD99 GFRA2 DAP SOX10 PLIN1 STK32A PPM1B WBP4 ARHGDIA ARFGAP1 FIBIN SLC35A3 CKB COL12A1 CUTC RBM24 HOPX LMBRD2 DHRS11 AR PRLR FAT3 SRSF2 PDLIM5 EDC3 NAIF1 CCKAR EFTUD2 SMPX CARS ARIH1 DHRS3 PKIG RAC2 MTA1 ETFA PHF21A SEPT2L CTD-2116N17.1 OPNP USP45 LIMK1 S100A1 IMPG1 PRPF3 IRX4 5-Mar DCTN2 AMBP PMP22 NTMT1 DAPP1 CRCP AKAP2 ABHD17B COPG1 PANK4 MRPL34 AMPD3 THOC5 CDKN1B FAM105A PSEN2 FAM103A1 HNRPK 5-Sep CNBP C1H11ORF54 MAPKAP1 GID8 COL8A1 ATG9A B4GALT2 TCF15 MGEA5 RIT1 WDR83OS APP UBE2V2 SRC ZBED4 NME5 SIKE1 SBNO1 ETS1 BORCS7 TMIGD1 A4GALT SSRP1 RALGPS2 ITGB1BP3 CDH17 SYNGR3 CPT1A TIMP2 MLNR LOC396531 CNGA1 MCTS1 CNR1 RAB14 CDH20 MSRB3 PIT54 LOC418667 AP2M1 BARX1 SEPN1 AvBD1 ALDH1A1 BTN1A1 TMLHE CMKLR1 ST3GAL1 STX16 PTPRO LOC420860 IRF1 SERHL2 SOUL BRD2 FLT4 LOC420849 UTS2B FEN1 NEIL1 TIPIN C1H21ORF91 ARFGAP3 PRORSD1 FSTL1 CKMT2 ABTB1 RSL24D1 NEK7 KCNN2 CNN2 HNF4beta PARP1 SPPL2A ARPC4 RPL34 ST3GAL2 UBL7 PLIN2 RNF152 MSN NGFR EHD3 MMADHC CDH7 FKBP8 ATM FOXO1 THOC3 LMX1A CHD2 VDAC1 TMX4 RASSF5 TOX3 KPNA1 PAIP2 WDR92 G3BP1 SRGAP1 REL METAP1 RPP38 CBFB ANGPTL2 GTF3C5 LOC776816 HPGDS IL2 GJC2 STRA6 STK17B PRDX1 RCHY1 PDE6H HNRNPR CLN8 ARHGEF3 ENTPD1 FYB TDRD7 CDKN1A PSMD4 GPR149 STX17 CASC4 CBY1 TRAF5 ZNF800 CDC25A NFYA BHLHE40 ZYX RAD9A CIDEC SLCO4A1 PTP4A1 AGO4 SEC22B GPR83 FZD7 NCAM1 OSTN CTSS SNN MAFA TRA2A INHBA ARFIP1 MYH10 NRN1 C14ORF2 HERC3 RPL31 EARS2 SGK1 RAD51D UTP15 MTIF2 POPDC2 SET MMP16 PLEKHM1 AGPAT9 IHH EDN2 TIMM8A KIAA1143 PPP6R3 THY1 NGEF TCEANC2 POLDIP3 KRIT1 DHCR7 TOP3A CMC2 ZDHHC17 SLC1A2 NCBP1 HCCS GOLGA7 MSANTD2 ACTB EPB41 MMD CTSA PKM SRSF5A ESR2 EXOC6 YRK CTD-2370N5.3 DNTT TMEM121 MARCKSL1 CMTM7 SLC6A9 FGFR2 GATAD2A MYL9 SYTL1 ABCC1 PDGFB ZDHHC8 NCAN BANP TWSG1 SPRYD7 GNG5 LIN52 TMEM129 PFN2 RNF103 TNC ZNF410 TP53I11 NQO1 SMPDL3B P2RY1 NCDN ITGB2 TRIM59 ENTPD8 BMP4 TANGO2 NR5A2 CDCA4 TWISTNB GLUL CELF1 CTDSPL2 RGN RBPMS2 ELAVL1 CACNA1S TWIST1 IGFBP3 HOXB4 PTPRU DEK NCSTN NR5A1 TCIRG1 NTN1 CRYZ C26H6ORF106 ADCK3 SEMA7A KTN1 CD5 RDH10 CACNA1B ST6GALNAC1 NUDT19 CLIP1 XPO1 HDGFRP2 SUMO1 NEMP2 ASB7 FZD1 ORC3 NECAB3 UBE2V1 S100Z SLC22A7 SULT1B1 HVCN1 SASH3 WDR44 SATB1 ATP6V0B FSHB WIPF1 RBMX TBC1D1 PCGF2 AZIN1 CCR5 ANXA7 ETV6 SMIM7 C3AR1 NUDC PMM2 CCPG1 LAMTOR3 TJP2 YTHDF1 E2F4 SYK NAGA LYRM1 JARID2 AKT1 TOMM6 USP48 NEUROG2 PRRC1 FPGT RHOG MTFR1 RAD18 UGT8 ELMO3 RFTN1 ZFP92 BRSK2 CLK2 RAB10 BASP1 GMPS GNG2 ALAD PRR5 CATH1 ATP13A2 LRP5 VAC14 PHKB PTBP1 SGK196 FOXA2 BCL2L1 MFAP1 VCP CYP46A1 TMEM104 BCL2 ERP29 FXYD6 VMO1 ELAVL4 EIF1 SSTR2 GNB1 FAM213A PHYHIPL IAH1 PAX6 PALM GAPVD1 PCSK7 HOXD8 MTMR2 PSMA5 BRCA2 ART4 JMJD6 EBF2 EMB ERBB3 SAP130 NUP50 CLDND1 HCLS1 FAM172A CEP63 SMIM3 STAT5B DKC1 CLDN3 MUM1 TLDC1 MGAT3 TANK MEPE RBM25 WDSUB1 FBXL12 GALNT1 OLIG2 ERICH1 RIC8A NF2 SCOC RPS6KA1 FBXO34 LOC416354 MXI1 LOC770548 SYT13 FAM46C LOC419851 KLHL10 MAGI3 RP11-49K24.9 SPTSSA GCHFR ABRACL ZCRB1 AIFM1 OPA1 MOB4 HDAC11 HESX1 CRH MAVS FKBP4 ACADSB GJC1 SREBF1 HS6ST1 CCR8 LOC422926 NUP93 ABCA1 MCL1 SULT1B RRP7A FGL2 UBE4A DNAJC5 FAM60A PRRG1 SNCB IRF4 CNOT1 STXBP3 CD80 TMEM170A ITGB5 MYO5A RP11-87C12.2 SGOL1 MYOM2 RGL1 RAN SLCO2A1 SLC25A14 ACACA TPI1 RPS19BP1 APOA1 PCMTD1 ZNF706 UBE2G1 ANKS1B C1H2ORF49 ZFAND5 ANG CACNA2D1 KCNJ2 COL18A1 ELOVL6 GPR89B LMO4 IKZF5 RAD21 ADORA1 DCUN1D1 SERPINA4 RHBG RAB33B ARF1 ADSL CHM WLS TRPV4 CERK VDR ASNS HTR1A PAN2 P4HA1 FAM46A MYBPH VPS33B RASGRP3 PNPLA6 VDAC2 TM2D3 RHOA TWIST2 LOC420419 ETV5 NRP1 TARDBP MRPL37 SFRP2 SETD3 ETFDH KIF3A WDFY2 CENPO INSIG1 LOC415664 CCT2 NEURL1 DDX6 NUCB2 WRB CASP2 TMOD3 CANT1 BTBD9 MTMR8 ILK BBS2 ZBTB26 KPNA4 ANKRD16 CXCR7 LIMD2 TAPBPL NUDT5 RBM5 C12ORF57 TAF3 YEATS4 DLST ALKBH3 IL5RA AGO1 BTBD10 C15H12ORF65 ARHGAP29 TNFAIP8L1 TRIM71 RMDN1 CCDC28B C10H15ORF59 IMMP1L TMEM184C THADA TBL3 SEPP1 MPP5 CRYL1 KCNA4 GMPR WFDC1 KIF18B SEC13 RAD17 BEND7 YWHAH FZD10 MPPED2 GPATCH2 OST4 TRAPPC13 PPDPF WWC1 ARID4A MTMR3 TMEM180 ATF4 AAMP GLOD4 STX7 RALBP1 TMEM56 DMTF1 NEUROD1 CCNC CASP3 LUZP2 CHERP FHOD1 COX6C XIAP IGF1R YWHAG FAM76A PPFIBP1 CA2 PARK7 SYNGR1 MYBL2 SUMO2 BMP15 CEPT1 ANXA6 ACBD6 LGALS3 TCP11L2 VCAN SALL1 DTWD2 RHOC TBX22 TOB2 CD3E ICMT SQLE ACVR2A EHMT1 IGF2R WDR3 ISL1 RAP1GAP2 CSRP3 FAM173A C14H17ORF103 MBD3 SLC16A3 LYRM4 NDUFB2 ENTPD2 FBXW5 FN1 NRBP1 ATF7IP SNX2 RIPK1 UBLCP1 DCAF7 MAP1LC3B CSNK1E IL13RA2 PARPBP STARD4 ORAI2 TCEA1 COA7 TMEM141 ACAP2 CNTN2 HYAL6 FAM122A TPRA1 GJA4 MEF2D CTSB IPPK POLR3H NAMPT MEF2A IL8L1 SNX12 SCNN1A ORAOV1 PDS5B STAR CHRDL1 HDAC7 S100B COL9A2 PDGFRA FBXW11 ULK3 HABP2 CRYBB1 PPP1R2 DNAJC6 ZNF767 ZFYVE1 TALDO1 SOX14 SLC47A1 PPP3CB ATP6V0E2 LOC427470 CD276 IPO13 TMED8 TRAFD1 BBS4 ZIC1 VPS41 GHITM MYL12A GLT8D1 SGTB PTH NTPCR KIAA1191 PAFAH1B1 AC113404.1 EIF2D PARP4 PAPD7 GEMIN4 TCF7L2 SERPINE2 NIP7 YTHDC1 CDC42SE2 MAFF RNF4 NSMCE2 C1ORF146 ZBTB2 GAD1 PDE4B YWHAB BTK PEX2 CALM1 UBN1 OTX2 ARL8A ZBTB17 CDPF1 C4H4ORF29 FLI1 CNIH1 AARS2 SLC26A5 MTHFD2 TMEM230 RPL32 RAB35 DYM EEF1A2 FASN TDRD3 CNDP2 TBK1 BAZ2B CHP1 PCM1 STIM1 RGS4 TEX264 SLC17A9 IRF2 MATR3 FZD4 GTF2A2 IL6RA PAK1 CLEC3A GARS PIK3CB RAB11A FLOT2 VPS35 CANX FAM114A1 ZNF639 SMC2 BRINP1 ACTG1 RBBP4 SYT12 APOC3 PREPL PRELID1 VIT COL4A1 NRTN MYH1B CUEDC2 ST6GALNAC4 HPS1 TRPC4AP WNT5A BID CHST10 TFEB DOLK CD1C MYD88 SOCS3 TPM2 NFKB2 HIPK3 PRIMPOL CNOT7 YIPF3 PRTG IGF2BP3 COL6A1 KIF11 RAB32 AMIGO2 RNF111 SLIT1 PPARD CRYBB2 SOX1 E2F6 TYRO3 IL1RN TCEB1 ARRDC1 RMI2 ENOX2 RP4-613B23.5 SDF4 ASNSD1 KLHL13 FOXM1 RECQL CLOCK AANAT DR1 GLT1D1 MYLIP ENO1 RELL1 SZRD1 USP6NL RGP1 RGS16 SUPT3H STOML1 BORA KCNJ8 PPP2R4 SDR16C5 PTPN9 LOC769139 DNM1L CUX1 PSME3 JUP LONP2 APOOL F2 FABP6 SRSF1 TMEM120B ATP6V0D1 LRRC59 WASL PRKD1 LGI2 NFAT5 HIAT1 SOCS4 CRTAP AC025048.1 VAV2 TPH1 KLHDC2 RBL2 ARHGAP26 ZSWIM7 DDB2 P2RY6 SLX4IP CDK1 STAT1 AXIN1 PSMC6 SMYD2 ADD1 LDLRAD4 NRXN1 ZFHX3 GATSL2 STK10 SGMS2 PPP2CB HHATL ACP2 IL10RA PPP1R8 UTP6 RBMS1 STK11 ENSA LBH SPATS2L RNF25 HSPA8 ACLY RNF14 LOC395991 MYH1A POLD3 HMGN2 PUS7 AREG FAM195B NAA35 SEC31A GPX2 LIN9 RPA1 ALCAM NCLN RDH5 MAOA TNFRSF19 ACAN GNB1L CASP6 TSC22D3 CCNK INO80 TEAD1 PTDSS1 CPA6 MRPS33 RAD52 PUF60 SKP2 ASIP PIK3CD FAM104A GNLY KERA RGS6 MAPRE1 FAM65B NINJ1 FHIT COG4 FAR1 NFASC RLIM UCK2 SLC35E3 RFXANK MIB2 BAK1 WDR24 VAMP7 BIRC2 SLC20A2 COX16 TBL1XR1 FZD5 STX2 PIP4K2A MRPS16 LOC420160 C3H6ORF203 CCNE2 BTC IL6ST CDH6 STRBP ETV1 RNF13 DFNA5 CRABP1 NR1H3 RP11-195F19.29 APPBP2 DCBLD2 RAE1 AP4B1 SLC25A6 GALK2 GALNT6 NFE2L1 ORMDL2 DCTN1 YWHAQ NCOA1 TERF1 PSMD3 ANXA11 C26H6ORF89 PDPK1 GLP2R KCNA2 CBLN2 LECT1 ENS-1 ACE TBX18 P4HB MEOX1 COX7A2 ATP4B C7H2ORF76 NAP1L4 GDI2 HMGB2 SERTAD2 VGLL4 PDP1 MLPH TM4SF1a HACE1 STK11IP KPNA2 IGFBP2 RP11-403P17.5 PSMD14 PPP1R3E GIGYF2 AAMDC PPME1 DYNC1I2 CCM2 COL17A1 AHSA2 LCP1 LPP BUB1 SMARCE1 EEF2 MXD1 CORO7 NCALD SERINC1 IDUA THYN1 CDCA7L CLP1 PLN P2RY14 DNAJB12 WBSCR22 GFOD2 TBL1X FOXD1 RHOT2 LGALSL COX4I1 EIF6 VSX2 ST6GALNAC2 9-Sep VPS4B NDP PABPC1 GART DCT RHOB CPNE1 GFRA1 PFKP FGFRL1 TPST2 ARHGAP15 TOX ABHD12 CSF3 GJA1 TNFAIP1 SLC25A22 APTX PSMC3 NEUROG1 RAX2 PHTF2 ERH GABRA1 CKS1B KNSTRN ABHD13 ZP2 WDR18 GABRE LRRC57 FYTTD1 SSB GLRX C3H6ORF120 MYLK2 EIF2B3 PRRX2 S100A10 PSMD1 KIAA1671 CHRNA6 EXOC8 WASH1 CSNK2A2 CSDE1 TMCO3 SRP68 VNN1 RELA ADA NCOA4 CCDC61 EI24 TRIM55 TNNT3 UBE2G2 DCN SERINC2 FAM192A SNX16 LOC396224 ZMYND19 MED9 ALG12 OSBPL2 MCFD2 TTC7B EDA2R LYRM9 NCKAP1L CHST3 ZNF512B CD151 SRF TBCA SUV39H2 DLX6 LXN AIDA TGIF1 DNAJC12 CBL PLEKHB2 RABL2B CCNB3 SH3BP2 BZFP1 MYOD1 ACOT9 LPAR4 YPEL2 RHNO1 NOP56 EPHB2 SLC31A2 MTMR9 ERLIN1 AHCYL1 SGPL1 PLCXD1 RABGAP1L KLHL2 PODXL EPGN NPC2 PIGBOS1 SNRNP200 CHUK ITPK1 CKAP5 RP11-20I23.1 MORN4 PIK3R5 HIC1 PSEN1 DDX4 FAM49A TNFSF10 PLEKHJ1 DNASE1 FBXO7 YBX3 MYH1C TOP2B ANAPC10 SEPSECS GREM1 AGO3 FURIN EGFR UBIAD1 EXOC5 KLHL24 ST3GAL5 PCMT1 MAP2K5 CREBL2 MAP3K14 RFC1 KCNH6 FBXL21 SYNPR HNRNPLL MVB12B NBL1 TBX4 CACFD1 B4GALT7 SLC8A3 COL6A3 HOXB1 CHRNB2 GHRH OCX36 FSHR ZDHHC5 ACSBG2 NDEL1 ART1 FAM21A CNTN5 PTX3 TNNI2 HSBP1L1 DAGLB UBN2 CREB1 TNFRSF1A ARNTL MYO1C ABI1 ATP13A4 CMPK1 MRAS KIAA1467 WWP2 OVALY LOC424740 YIPF4 PARN TSKU XIRP1 FZR1 TOMM7 PHLDA2 RUNDC3B CINP DENR UGDH WWOX ELN TICAM1 CNGA3 TLN1 FBXL16 UVRAG AvBD5 CYB5B PTN CDX2 RGS17 6-Sep CCL19 PLIN4 LEFTY2 RALGAPA1 RAB3IL1 LSG1 IL16 CSRP2 NT5C2 MFAP3 SMIM5 MYL4 JAK1 KCTD14 HSD11B1L RP11-834C11.12 CPSF6 ARGLU1 MTSS1 QARS PACSIN2 CDC42 RGS9BP NAPB CD36 SH3BP5 ARHGAP19 RREB1 OCLN PMS1 KDR CLN5 TLR4 BPGM SLC19A1 PIK3CA RPRD2 CBX4 C5H15ORF57 FMOD NFKBIZ TMEM173 SLC16A1 DDX47 NR2C1 MED24 SPG21 LOC100858797 MID1 APCDD1 CDC27 ACBD3 N4BP3 ATP1B1 DPH1 DNA2 NME3 FGF1 MRPL53 DRAXIN DNAJA1 TMEM55A SCG5 RANGAP1 PTRH2 CTGF RBM19 SLC39A13 PDLIM7 ALDH1A3 LOC772071 PDLIM4 NUMB CSPG5 APC2 LOC395647 PIP5K1A EYA4 GDPD5 LFNG B4GALT1 ZC3H14 GNAT1 PEMT PRNP FAM20B PDCD6IP TTL DEXI PAQR8 C1H12ORF23 GJA5 ETS2 RAD51 SLMO2 RBM22 CDH5 SPINZ SLC39A9 RNF185 CLINT1 CCT4 MXRA8 MSGN1 PNRC2 RSFR PPHLN1 CACNG3 TSPAN15 ZNF384 CNOT2 CIRBP LOC419112 DPYSL2 YPEL5 YBX1 ATOX1 H1F0 TAF2 MYH11 CEBPB CD82 CHPT1 TULP1 C1H7ORF60 PRKAA2 WDR82 NKIRAS2 C1GALT1C1 POLE3 ANO5 C11H19ORF12 MAPT LRTOMT PDIA3 B3GAT1 RPS12 SHOC2 USMG5 MCCC1 FOLR1 GNAQ RP11-290H9.2 NECAB1 LOC426385 GLYR1 IFT81 ARHGAP8 FAM222B PLA2G7 TMEM123 ARHGAP25 NRG4 IRF7 LHFPL5 SEPW1 SNAP23 KLHL14 P2RX4 SLC9A2 GARNL3 DCK HMGCS1 NONO TF NHLH1 PLAG1 C1GALT1 ADAM9 CYP51A1 GBX2 ALDH4A1 MYEOV2 PCGF5 IRAK4 ORC5 CASP14 ARL8BL DNMT3B BHLHE22 BTF3L4 LEF1 AvBD4 CDKN3 NR2E1 TTPAL PTPRZ1 RP11-145E5.5 DBN1 ANP32B RAB8A PELI1 GBE FOXC1 CLDN2 DNAL1 TMOD4 SPATA2 CUL2 ALDH3A2 NECAP2 HMGA1 MET MAFB PHF5A NGF MFGE8 HMG20A TNFRSF18 MAGI2 PLEKHO1 RABL3 CBX3 C5H15ORF41 TRIM39.2 GJB1 CBX1 LAMP2 PRC1 RTN4 CHAC1 DESI2 PSPH SGTA SLC40A1 HAS2 ZDHHC21 NDUFB1 VGLL2 TSPAN6 PAFAH1B2 ELOVL5 CPNE2 RECQL5 CAB39L SCAF4 8-Mar PIGR BRAP SIGMAR1 RGS19 ZNF330 CDK10 SSTR1 SLC38A2 RER1 ST3GAL3 HCRTR2 CAMK2D TMEM68 RHCG TXN2 DUSP10 HS6ST2 EPHA1 RASSF2 OAT F2RL1 YWHAZ RNF11 KDM5B C13H5ORF15 CNOT10 HSPD1 PHKG1 KCNAB1 TRAF7 AvBD12 LOC421975 DEAF1 MYH9 NBN PNRC1 SLC34A2 HSP25 RNF123 ALC BTBD1 KCTD2 GPX3 SERPINB10 SNAP91 RP2 SPP2 CHMP2A PDCD10 MBNL1 SLC38A4 AC005943.2 FAM45A SP5 TSTA3 TRIP13 CREM VPS53 ARL2BP CACNB4 NFIL3 BEAN1 RAG2 HDAC4 SMIM18 IFI27L2 COL22A1 ERN1 CD24 ZC3H15 TPM1 MGAT4A FBXO8 HMGCR CLEC3B TEF STRAP RAB24 BRCA1 BSG STX6 TSPAN3 DOLPP1 PBRM1 PYGB FYN UNC5B DNAJB6 ANP32E LRRTM3 B4GALT6 HNMT MXD4 RASSF3 TAF8 SALL4 PSMD2 ERGIC2 TNS3 BET1L FBXO32 CAPN1 TAF5 PLAU MFN1 ANKRD10 NAE1 PPP2R2A HES5 SMIM12 CCDC167 CTNNA3 KLHL18 TMEM184B NAV3 GTF2H4 MELK SH3KBP1 NEK6 ANKRD27 SRGAP3 PIAS2 SMIM15 CNOT8 EXOC2 TADA2A WDR45B GNRHR RAF1 ELK3 KLHL20 VEGFA PLA2R1 AKAP9 POC1B MOV10 WDR77 BORCS5 APBB1IP MGAT4C OVALX TEAD4 NOTCH1 K123 GFPT1 ATP2A3 ARIH2 KIF3B KDSR ELL CYTH1 CD200 DYNLL2 FAM118B CENPH ATP5B EGFL7 NFIX NR2E3 TMEM167A VPS50 TRABD FHL5 ATP6V0E1 LY75 ASB3 HMHA1 PUM1 KCND2 STMN1 HAVCR1 KIF5C AGTPBP1 NELFA BRE ST6GAL1 FZD3 CD200R1L KANK1 FGFBP2 FDFT1 LYPLA2 C11H16ORF70 ELOVL1 VWA9 ALKBH2 ADARB1 STUB1 DHX38 BET1 CCDC101 COG7 TOP1 HRAS ABCF2 ODC1 CSF3R HIC2 SOX17 GTDC1 TMEM70 SELO NKX3-2 FAM126A STAT4 SERPINI1 TACC3 STK40 ELF1 SMAP2 ECE1 RLBP1 ID3 E2F1 CYP1A4 UBXN10 UBE2F TRAPPC3 IGFBP4 ACBD5 ATP6V0C PIGY RALGAPB LBX3 NOTCH2 STAU2 MYL1 CERS1 FAM214A CLTB C7H2ORF69 LOC100502566 C2ORF88 LOC100859039 |
|  |  |
| gga-miR-133c-3p | KLHL14 DYNC1I2 CXCR7 5-Sep SP5 TLDC1 WDR92 CAB39L ULK3 KLHL10 CCL19 SRC DCTN1 PLIN1 TRIAP1 SRSF2 HYAL6 ACAP2 CPNE1 MYL4 PGA PAPD7 VWA9 TMEM55A FBXO34 CASP6 FKBP8 VCAN GATA4 SNN KLHL13 IGF2BP3 PPP1R12B SGMS2 AARS2 MAVS RAE1 RELL1 DNAJC6 RDH10 PLEKHB2 NEK6 TMEM70 FEN1 PHKG1 FAM173A ITGB2 ANKRD16 RAD18 ABTB1 MYL1 PRORSD1 N4BP3 NINJ1 PDP1 RP11-834C11.12 MELK PTPRU IDUA ARL8BL BLOC1S2 AMPD3 PIK3CB ZNF384 BANP C1GALT1C1 F2 SPPL2A LOC420160 LOC396224 GFRA2 CANT1 FGFR2 ATP4B STARD4 CUEDC2 BBS2 FOXM1 MSN SH3BP2 CCT2 BRCA2 ENOX2 TSPAN6 PWP1 TBCA CEPT1 SRSF1 RABGAP1L HOXB4 PAN2 8-Mar HMGCS1 PIP5K1A HMGN2 OCLN ABCF2 BHLHE23 MAGI2 SOX11 STXBP3 DNAL1 GJC2 HVCN1 EIF5A2 RTKN2 WASL SPATS2L ABRACL PNO1 TAF8 CAMKMT STX2 EPN2 ARGLU1 TRAFD1 LUZP2 TMEM230 DAGLB RAD52 TRIM71 FABP6 CD3E ELOVL5 FGFRL1 RALBP1 NOP56 GOLGA7 AIDA CBL TGIF1 RP2 POLR3H TPRA1 GLT1D1 CKMT2 HCRTR2 CORO7 PNRC2 SYNGR3 PLEKHM1 LGI2 MGEA5 SLC38A2 RGS9BP FZD1 ANXA11 RAF1 SLC39A13 A4GALT MTIF2 PDIA3 TBX6 NELFA CTNNA3 EBF2 STIM1 STK17B ORC5 NSMCE2 CCR8 DCTN2 GBX2 RALGAPB ITPR1 KIAA0020 RAD51 UBE2J1 STRA6 C1D IL6RA ERP29 CDC27 KCNAB1 STRAP ESR2 TDRD7 ELK3 LIN9 CCNB3 OSTN TRAF5 MYLIP ABLIM1 ZFAND6 COL4A1 FYN FYB CDKN1B MRPS33 PXN NFIX COL18A1 CNN2 MMD BPGM RGP1 ST3GAL1 GPX3 ABI1 TANGO2 HSBP1L1 MLNR MRPL34 CUL2 CLDND1 ZNF767 E2F6 MAGI3 SASS6 PLIN4 PTRH2 FXYD6 MPP5 TMEM167A MCFD2 HDAC7 STK17A ARFIP1 GCLM HPS1 TMEM141 IGFBP3 DHX38 CNGA1 GABRE HESX1 AAMP FAM46A BPNT1 PARP1 COX7A2 BTN1A1 RALGAPA1 ZDHHC21 ZSWIM7 GPR149 SEMA7A GNRHR PSPH SMYD2 BCL2 HS6ST1 INVS CBX3 CCDC28B FAM177A1 PTBP1 ARIH2 TMLHE MED9 FBXW5 TMCO3 HMGA1 TAF2 COX4I1 SLC38A4 LBH HHATL PDPK1 FBXL16 TMEM208 ATOX1 ANXA7 ZMYND19 SLC47A1 COPE ALDOC KANK1 ASB3 TWIST2 WFDC1 NCOA1 FABP5 CDH7 STK32A ING3 SMIM18 NR1H3 AIFM1 MCCC1 HSD11B1L GBE CDH6 KIAA1467 SNAP91 HERC3 NCDN NBL1 API5 RIT1 PEX2 PRPF3 PCSK7 YWHAB POFUT1 ST3GAL3 WNT5A FDFT1 LY75 PAQR8 SOX10 CMKLR1 SMC2 LYRM1 LAMTOR3 ZDHHC5 ACTG1 ASNSD1 RNF20 GNAQ XPO1 TOX USP45 PARPBP TADA2A APPBP2 ZNF410 JMJD6 DBN1 CEBPG ZNF706 NEUROG1 ALKBH2 AGO4 FAM65B DNAJA1 NDP CHRDL1 DAAM1 CHST3 TRIP13 NFKBIZ AC005943.2 MTA1 IL8L1 WDR77 C14H17ORF103 IMPG1 AP4B1 ZBTB17 HABP2 COL8A1 FZD10 DOLK MEOX1 SET EARS2 EDA2R WDR82 ARID4A ARL2BP HCCS ABCA1 NUP50 ATG9A LOC415756 HPCAL1 CSRP3 RSFR HOPX TMEM164 TBC1D1 RHOC UNG ALKBH3 SRGAP3 TBL3 RGS6 SGPL1 PMM2 SSRP1 HDGFRP2 YWHAZ ENPP2 MFGE8 AKAP2 SLC22A7 WDR24 ARFGAP3 SPTSSA BIRC2 FBXL21 CASP3 MYOCD VPS4B SLC39A9 RGN MED24 TLN1 OCX36 SERPINB10 PPFIBP1 SEPN1 CD80 XIRP1 PEMT NFRKB KIF5C CDK1 FOXC1 RHOT2 RHBG SELO ST6GALNAC1 IL16 KCNA2 NBN C2ORF88 ATP13A2 SOX1 WNT2B NEUROD1 ENTPD8 ENSA TRAF7 PTX3 BARD1 RPS19BP1 C1ORF146 TDRD3 CREB1 UNC5B KERA TSPAN3 PHYHIPL PAAF1 APOOL FSTL1 C7H2ORF76 RER1 AREG ALDH1A3 PHLDA2 C14ORF2 CERS1 EIF2D ABHD13 TCEB1 MSGN1 SLC35E3 YPEL5 ORAOV1 UBE3B DNAJB6 SPP2 RGS16 NECAP2 NAIF1 MPPED2 SEC13 NSUN4 ACLY TCEANC2 SPATA2 NFKB2 UTS2B ENS-1 PIP4K2A MYH1B PDLIM7 GPX2 UBE2F RCHY1 CDH17 FZD3 ARHGEF3 OVALX DPM2 CKB SCAF4 LCMT1 LBX3 IGFBP4 RGL1 CREBL2 INTS2 PDE4B TNNI2 MLPH CACNA1B LRTOMT FKBP4 STAT4 WNT7A HPGDS SLC10A7 FAM105A CD200 UBA52 SFTPA1 TGFBR3 RRAGC KIAA1671 RMDN1 VPS53 CALM1 PPP3CB MBL2 TSPAN1 PNPLA6 ALDH1A1 HMGCR SLC1A2 PFN2 EGFL7 MYOM2 TRIM39.2 DNAJC5 C15H12ORF65 SUMO2 FZD6 PARK7 AHR RABL2B CACNB4 SLC2A2 ZIC1 ARHGAP26 WDR18 IRF4 PKM TNFRSF1A HSPA8 GCHFR COPG1 ENO1 PAX6 CLN8 LEPROT PPP2CB GTF3C5 RIC8A RHOG MXI1 POU1F1 SCN9A UBE2G1 SLC17A9 RPA1 BAG5 LMX1A INO80 POC1B NUCB2 MYEOV2 ANAPC10 ARHGDIA RAB8A PNRC1 SEC22B EXOC2 FAM20B CANX BEND7 SLC25A6 TMPO RBM24 SERPINH1 LOC419112 COPB1 SCG5 SLCO4A1 ACTB DDB2 ACVR2A SERINC2 SNCB UBN2 MORN4 S100Z PDZK1IP1 RELA ADAM9 MTMR2 PABPC1 ASNS CDH5 ARRDC1 INTS8 RP4-613B23.5 MGAT4C IFI27L2 SLC9A2 HES5 RPL31 TNFRSF18 CBFB SH3KBP1 EPHB2 RAC2 FBXL12 SRSF3 RNF11 TBX22 LCLAT1 HBAA RP11-87C12.2 DLX6 WDFY2 DRG2 RGS19 RASSF3 EDN2 CSNK1E JARID2 WRB DNASE1 EHD3 TIPIN PRELID1 CD1C TPH1 CRTAP PRR5 INHBA PSEN2 RP11-20I23.1 ATP5B LOC100858797 FAM213A RAB9A C4H4ORF29 CLN5 TMEM173 LRRTM3 ANKRD10 SRP68 ETS1 RDH5 TWISTNB GDPD5 XIAP SALL4 PUS7 IRF2 ELOVL6 KTN1 CBX4 TRAPPC13 FAM126A MYH1A PRTG E2F4 SAP130 PPP2R4 ACACA F2RL1 MTO1 DNM1L HSP25 ZC3H15 PCGF2 RAB3IL1 VEGFA FASN FAM3C STK24 IRAK4 MAP1LC3B CHAC1 ZNF330 PLEKHJ1 VAC14 PSEN1 AGTPBP1 KLHL2 ADIPOR2 KLF11 GABPA FBXO7 ALAD PRRG1 LEFTY2 SEPP1 IL13RA2 STAR CRYBB2 SERINC1 AMY1A MUM1 LXN GFPT1 MMADHC SULT1B1 C5H15ORF57 ACP2 CDCA7L CHD2 NRP1 ESF1 PGR SOCS3 MAP2K5 CLINT1 RP3-461F17.3 GNB1 AvBD5 HDAC4 SSTR1 PSMD2 CKS1B LPAR2 THOC5 FOS REL RBPMS2 FOLR1 HDAC11 LRP5 ETV5 SYNPR MARCKSL1 7-Sep TWIST1 VGLL2 ICMT ATM RECQL5 ALC SLC20A2 FAM214A LOC772071 CRH HNMT CACNG3 ORMDL2 DYNLL2 ALDH3A2 TEAD1 OPN1SW PLEKHO1 PRRC1 THOC3 NEUROG2 PREPL MID1 ELF1 RECQL EIF1 SPINZ TP53I11 MAPRE2 DUSP10 CASP14 HIAT1 ZP2 HOXD8 SRGAP1 GALNT1 CACNA1S SOX14 CHP1 NRBP1 COL17A1 RMI2 CACFD1 BIVM TNFAIP1 UBE2V2 IGF2 CCNE2 CDH20 SDR16C5 IGFBP2 PBX1 ODC1 RALGPS2 C11H16ORF70 EYA4 PIGBOS1 C1H7ORF60 BTF3L4 PCMT1 MYD88 POLDIP3 GNG11 FHIT RGS17 PPARD IGF1R CNOT7 FAM122A AKT1 RUNDC3B FZD7 CDCA4 RNASEH2B PIGY UQCC LOC420860 TCEA1 CCK ERGIC2 PRIMPOL POLK BTBD1 CECR1 MEPE S100B CCPG1 INTS9 LCP1 CNOT8 CD36 SMARCE1 ETV6 DRAXIN LOC418667 GJA5 RNF123 CNOT2 P4HA2 LOC100859148 KCNH6 MAPT CERK CD276 MRAS CD99 CCNC RAP1GAP2 CPT1A C26H6ORF89 IRF7 BSG PMEPA1 TMEM129 ITGB1BP3 KIF18B BIN1 NUDT19 SUB1 KPNA4 TMEM68 GALNT6 CTNNA2 MTMR8 UTP15 RAG2 CDC42SE2 BRAP LMBRD2 UBE2G2 CNBP WIPF1 FIBIN SYT13 AvBD1 FLI1 FSHB BARX1 GFOD2 NONO VGLL4 GTDC1 VPS33B CIRBP RPS4X DDX47 PTPRO HS2ST1 LOC100502566 ANG KLHL24 CENPH NCSTN RBM25 B4GALT7 VTN CNR1 WDR1 DDX4 BCL11A CTNNB1 SCG3 APOC3 ZFAND5 STX7 IDH3A LFNG AANAT TOB2 DIO3 YRK C10H15ORF59 GFRA1 ZNF639 BNIP3L WBP4 ASIP SIKE1 LGALSL ACADSB SOCS4 UBE2H THRSP CCR5 PRLH ABHD12 WWC1 MTHFD2 PIK3CA RGS4 MXD4 NR5A1 C1H2ORF49 PBRM1 UTP6 ARHGAP29 PLAU 6-Sep NECAB3 SLC6A9 C1H12ORF23 FAM175B NGEF NCOA4 STX17 ERICH1 RLIM RASSF2 ST6GALNAC2 NF2 BRD2 NKAP HAS2 TRAPPC3 PRLR SLC12A7 STK11 CLIP1 PDLIM4 FAM104A UBE2I KDSR ERLIN1 PHF5A MGLL PRRX2 MAPKAP1 MYO5A GPR83 IL5RA DAPP1 MRRF TMOD3 DPH1 TERF1 MSRB3 BEAN1 GATSL2 CAMK2D NFAT5 VPS50 KIF3A MEF2A PRKD1 RAD17 TMEM180 CLDN3 PIGR GART SSB EIF6 FOXD1 SUCLG1 CIR1 AGO3 GPR107 B4GALT2 ARF1 SMPX RBM22 PLA2R1 PTP4A1 SGK196 NR2C1 PDGFB LOC769139 FZD4 PSME3 YIPF1 MFAP3 ART4 EPGN CRYBB1 UGDH DHRS3 AZIN1 LAMP2 SOUL TMEM104 BCL2L1 LOC770548 KBTBD4 BTBD9 CDX2 SLC34A2 DNA2 SQLE COG4 EFTUD2 GEMIN4 CRCP NAV3 KLHDC2 LAP3 RPL32 METAP1 P2RX4 TEF ARFGAP1 ZDHHC17 ABCC1 BZFP1 DFNA5 INSIG1 HDLBP UBXN2A FADS2 NCALD NFASC AKAP9 CUTC NR2E1 ACBD6 GJC1 SMIM5 PLCXD1 SLC26A5 RAB33B CRYL1 ATP13A4 MYO1C AMBP SMIM15 IL10RA SLC25A22 FGFBP2 CYTH4 LOC420419 COL9A2 ACSBG2 MIB2 DNAJB12 DLST TPI1 POLE3 APBB1IP OTX2 MRPL53 STK10 FAR1 AAMDC MAP6 FN1 USP6NL TOMM7 ADCK3 PPP6R3 TRMU PYY STX16 MAPK6 BID PCM1 DAZAP1 TCP11L1 ETFA KNSTRN LOC395991 MAFA RNF152 WBSCR22 FAM210A KLHL18 CHRNA6 CDKN1A CCT4 RLBP1 NCBP1 YWHAQ ZDHHC8 CHRM3 CCDC101 PLA2G7 RASSF5 CNTN2 PFKP GREM1 SLMO2 LIN52 NFE2L1 VIT FAM21A KDR DTNBP1 C1H21ORF91 NCKAP1L EPHA1 RNF14 CPNE2 ID3 SLC40A1 TSTA3 PPP1R3E ZNF800 ERN1 WDSUB1 UBLCP1 FBXO8 GARNL3 GJA4 BAK1 TBK1 GHRH APP COA7 ELAVL4 SLC35A3 STAT1 BMP4 PRKCI RTN4 NAT10 GLUL FAM195B DCT SPG21 G3BP1 IL1RN E2F1 TMED8 PHKB SUV39H2 NGFR CBLN2 FGL2 ZBED4 ADAM10 PARN CD5 POLD3 USMG5 AP2M1 MRPS16 ACAN PARP4 RPP38 E2F5 ORC3 WDR45B IRX4 CASP2 CTSA SERPINE2 RBM19 SATB1 TAF7 CYB5B SLCO2A1 VDAC2 CAPZB FOXO1 C12ORF57 RNF111 UVRAG SGTA H1F0 PKIG CPSF6 CPA6 K123 RSL24D1 SEPT2L PPARG YIPF4 PTH CDK10 RAB24 ADD1 RPRD2 ANO5 GTF2H4 EHMT1 NEMP2 C13H5ORF15 UBXN10 RNF141 TEX264 SKP2 TOP1 NKX2-5 GNLY TMEM229B OSBPL2 KCNA4 NCAN CSNK2A2 PRDX1 PTDSS1 RPS6KA1 GID8 IFNGR1 PPM1B SMIM7 PDCD6IP GLT8D1 PSMD4 COX16 LOC100859586 CTGF TNS3 CTD-2370N5.3 CSF3 PLN SMPDL3B UBE2V1 SREBF1 P2RY6 WDR3 MCL1 CEP63 MBNL1 PDGFRA RBL2 RAN RHOB TBX18 VNN1 BAZ2B C5H15ORF41 CDA RAB35 SLC37A3 CISD1 FAM49A KCND2 FBXW11 HACE1 SEPW1 WDR83OS CNOT10 9-Sep LIMD2 GAD1 PANK4 NAA35 MYBPH NECAB1 CTSB AMIGO2 HNRNPLL TTPAL ACYP2 CD247 IL6ST DEK PMS1 GNG5 XPO5 PPP1R8 MEAF6 CNP TCIRG1 RP11-77K12.7 DPYSL2 CTD-2116N17.1 KCTD9 STK11IP ERH ZYX TCP11L2 DNTT LGALS3 ATP6V0C CNDP2 RAB14 DCAF7 CSPG5 FAHD1 CARS ADSL VDR EGFR USP12-like ATP6V0E2 FAM45A MATR3 RNF103 SPTY2D1 BCS1L ZFYVE1 S100A1 ACBD5 ZNF512B DCN GPR89B TOP2B STAT5B LOC422926 AvBD12 RHCG SASH3 SLIT1 OAT THADA ST6GAL1 SH3GLB1 LPAR4 ATP1B1 RASGRP3 SOX17 ARMC1 SHOC2 SMAP2 ST3GAL5 MTFR1 SFRP2 FAM118B CA2 GTF2A2 GLRX BET1L MAPRE1 GAS8 COL22A1 NR2E3 AHSA2 LOC420849 STOML1 HOXB1 ADA ANKRD40 HSPA9 THYN1 ANXA6 ZBTB2 NRG4 PELI1 BUB1 MSANTD2 CNTN5 TJP2 CYP51A1 COX6C RBMX CHRNB2 SLC9A8 MAOA EXOC5 EEF2 GALK2 SYT12 SSTR2 CINP STMN1 C1H11ORF54 ATP6V0D1 TNNT3 FANCC NCLN TMEM184C EDC3 USP48 MCMBP FHOD1 RAD51D LOC776816 C5H11ORF96 BHLHE40 DCBLD2 LONP2 AGO1 NTN1 GNAT1 SUMO1 PCMTD1 TMEM121 CATH1 CCKAR FPGT TICAM1 CEBPB YEATS4 OST4 PIAS2 POPDC2 HMG20A FAM60A RBMS1 ADORA1 PDE6H MAP3K14 CTSK ART1 LOC100859039 OPA1 BHLHE22 RRP7A SNRNP200 PPME1 P4HB TRIM55 TP53INP1 FAM46C SNAP23 SETD3 MAFB NAE1 IHH GNGT2 IRF1 NFYA KCNJ8 CLEC3B GDI2 TMEM170A IKZF5 ARPC4 RHOA MYH1C C11H19ORF12 NEK7 NKIRAS2 CDKN3 TNFRSF19 CTSS KCTD14 PTTG1 FBXO32 COL6A3 CYP46A1 PRKAA2 AR ATP6V1D HMGB2 VPS37C TANK DPF3 TIMP2 TNS1 MYL12A CBX1 CYP1A4 CDPF1 RAB11A HMHA1 RP11-145E5.5 SGK1 BBS4 DCK YPEL2 AvBD4 HAVCR1 ARNTL PPP2R2A CCZ1 CSF3R EPB41 SNX2 SMIM12 LDB2 RBM12B RREB1 PDS5B P2RY1 TACC3 GLI1 RFXANK BTC GATAD2A CSNK1D RAX DEXI NUP93 ATF4 STAU2 NR1H4 KIAA1191 NTPCR KPNA1 RAB32 BET1 SCNN1A EIF2B3 ANKS1B HRAS QARS ACVR1 PPM1M TCF7L2 NOTCH2 BRINP1 CHPT1 ETV1 SLC16A3 TPM2 GJA1 NDEL1 CNGA3 IMMP1L BTBD10 MTMR3 NRG1 RFTN1 SIGMAR1 MGME1 JUN SBNO1 GPATCH2 NRXN1 DMTF1 PIK3R5 CHERP IPO13 KIF11 CRABP1 EXOC6 UBIAD1 GNB1L MAFF DENR FZR1 PALM MRPL37 ORAI2 LHFPL5 OVALY NUDC CLEC3A ETS2 MGAT3 HNRNPR BTK CD82 APC2 B3GAT1 YTHDC1 ANP32E NAMPT ST6GALNAC4 RNF13 NAPB FUT9 TMEM120B YBX1 ELAVL1 COX14 FAM222B BORA LOC396531 TULP1 WASH1 PLAG1 SYTL1 WDR44 TYRO3 DEAF1 ZBTB26 FAM103A1 DNMT3B CACNA2D1 NDUFB1 ACOT9 SZT2 PAFAH1B1 TIMM8A DDX6 FAT3 RAX2 FURIN TPST2 GIGYF2 MBLAC2 P2RY14 SPRYD7 FZD5 MMP16 ELMO3 CLK2 MTSS1 MXD1 CD151 NUMB LMO4 MBD3 PRC1 TEC PCGF5 TMEM56 NEURL1 SZRD1 5-Mar KIF3B ITGB5 TRABD MVB12B TLR4 WDR91 PTPRZ1 MRPL45 FLOT2 MYH11 TALDO1 NPC2 TTL KPNA2 BASP1 APCDD1 KLHL20 NDUFB2 PIK3CD LOC416354 DESI2 MYH10 ELL ARHGAP25 MYL3 THY1 NR5A2 LYRM9 PHTF2 TMEM178B SNX12 MEF2D SERPINA4 C3AR1 TMEM184B VAV2 CNIH1 RBBP4 ISL1 LRRC59 DCUN1D1 CHUK IL2 DTWD2 KIAA1143 TOX3 APOA1 GLOD4 FUNDC1 B4GALT6 SLC25A14 FGFR1OP2 PAIP2 ERBB3 YTHDF1 RABL3 PSMD1 CTDSPL2 ITPK1 SDK1 CLP1 TRIM59 PAFAH1B2 RNF185 ARL8A TFEB FAM76A CYTH1 DYL1 PPP1R2 GABRA1 HIBCH RNF4 WWP2 PRNP ELN ENTPD2 RP11-195F19.29 VAMP7 TXN2 NTMT1 NAGA GNB5 ARHGAP19 DNAJC12 MIER1 CACUL1 EXD2 YIPF3 FSHR GHITM DKC1 PSMD3 CRYZ ATP6V0E1 DR1 TMEM254 PTPN9 CUX1 FOXC2 CCM2 WWOX ADARB1 MTMR9 FLT4 CSRP2 TAF3 GMPR NT5C2 SUPT3H TNFSF10 FMOD CCDC167 RANGAP1 LYRM4 ZC3H14 FAM192A YBX3 CLPX ETFDH SEC31A PLEKHB1 PYGB PTN EEF1A2 PROM1 ALCAM MGAT4A YWHAH ALDH4A1 DHCR7 ARIH1 NIP7 NHLH1 SYNGR1 GTF2A1 BORCS5 STRBP SDF4 OPN5L1 OLIG2 TNFAIP8L1 SLC8A3 PIT54 PMP22 TRPV4 SMIM3 TF HS6ST2 CLOCK B4GALT1 SYK CHMP4C KCNMB1 OPNP HIPK3 UCK2 ANGPTL2 SULT1B VPS35 MEF2BNB STK3 LOC426385 LSG1 CD200R1L TRPC4AP CLTB VCP EMP1 KRIT1 CBY1 TBL1X MYL9 RFC1 ACTN4 KCTD2 UBL7 HNRPK ARHGAP15 UGT8 SLC16A8 CSDE1 ECE1 ZFHX3 NUDT5 PDCD10 GNG2 MYH9 BORCS7 MYBL2 SGTB AXIN1 NEIL1 AC025048.1 ATF7IP PLIN2 ZFP92 PPDPF PUM1 RNF25 SERHL2 ATP2A3 SALL1 FOXA2 ANP32B TWSG1 RBM5 DOLPP1 VMO1 SLC16A1 IFRD1 RP11-290H9.2 LOC395647 KCNJ2 MOV10 AHCYL1 TAPBPL LOC424740 TMX4 TOP3A ENTPD1 EXOC8 COG7 DYM IPPK CDC42 P4HA1 SCOC GLYR1 CCDC61 RAD9A COL12A1 PIGK AC113404.1 AGPAT9 C7H2ORF69 LRRC57 VSX2 BMP15 ASB7 IGF2R ARHGAP8 ADORA3 HNF4beta PSMC3 ADI1 CMC2 TSPAN15 NME5 EPT1 ARHGDIB APTX EI24 CMTM7 NAP1L4 CENPO TTC7B LOC421975 LDLRAD4 STK40 NOTCH1 LOC419851 SRSF5A PUF60 STUB1 TBL1XR1 MTTP CDC25A KCNN2 CREM TSKU MBP ELOVL1 NGF RPS12 C3H6ORF120 CD24 PODXL PEX5 EMB CHST10 ZCRB1 NKX3-2 BRSK2 ILK NREP ABHD17B C1H21ORF33 LIMK1 HSPD1 TAF5 ACE C3H6ORF203 CCNK MET FHL5 TM2D3 JUP VPS41 PHF21A TCF15 PPHLN1 ALG12 S100A10 CHM FGF1 HIC2 ACBD3 HTR1A FAM172A PSMA5 GJB1 ZW10 SNX16 TEAD4 PSMD14 SERTAD2 CMPK1 TMIGD1 TBX4 IFT81 SGOL1 YWHAG DHRS11 PAK1 MOB4 TECTA TNC MCTS1 RHNO1 RP11-403P17.5 GLP2R CLDN2 SRF TM4SF1a NCAM1 KDM5B CAPN1 LYPLA2 C1GALT1 USP15 TSC22D3 MFN1 NQO1 RPL34 NFIL3 ST3GAL2 P2RX5 CNOT1 SLC31A2 EEF1A1 SLC51A APELA GAPVD1 BRCA1 HCLS1 SERPINI1 GMPS NRN1 ANKRD27 SEPSECS TOMM6 PSMC6 ID1 DDA1 PDLIM5 MXRA8 TRA2A RIPK1 CKAP5 MYLK2 NRTN CHMP2A LEF1 RAD21 GARS MYOD1 UBN1 HIC1 COL6A1 TMOD4 LOC415664 SLC19A1 WLS PACSIN2 DDT NME3 CASC4 C26H6ORF106 TARDBP JAK1 UBE4A IAH1 ATP6V0B TAF11 LECT1 SLX4IP DAP LIFR SH3BP5 FAM114A1 TMEM123 TPM1 CIDEC RP11-49K24.9 STX6 LPP VDAC1 BRE RAB10 FYTTD1 CELF1 |
| gga-miR-138-1-3p | CALM1 NFASC RBM12 MEAF6 ZBTB17 SLC35A3 LIMD2 LOC425362 LZIC SLC47A1 LCORL MYBPH TMIGD1 MBP ENS-1 A4GALT LMX1A CUL2 NEURL1 TNFAIP8L1 RALBP1 KLHDC2 TSPAN6 PRRC1 NEDD1 DLD BORA GJA5 IMMP1L RAB35 TCP11L2 PCMT1 CDCA7L RAD21 CRIPT CDH8 CACNG3 PDS5B TRIP13 TNNT3 RECQL RAD54L IDS DKC1 KCTD9 IFIH1 MAFA TRAPPC3 SYTL1 GLUL ARFGAP3 PHKG1 GLCCI1 PALM NCOA1 RAC2 KIAA1143 PAX6 PSME4 DNAJA1 AATF PTPRU API5 TNS1 CD151 MAPK6 TRIAP1 RXRG CCDC101 CDPF1 FXYD6 AR PIGK MET PFKP IKZF5 TLR1A CNTRL TNS3 INTS2 SMIM19 GRIA2 SGMS2 FAM192A C1H2ORF49 KCTD7 TEAD1 ENOX2 FUT9 SERPINI1 GLT1D1 CTDSPL2 UBIAD1 TMEM180 RARS2 OPN5L1 RBM48 SNX2 PYGB MTHFD2 WBP4 FEM1B FOXO1 SLC8A3 PSEN1 EPN2 T SLC35E3 HIPK3 IHH UBE2V2 ARIH1 SOCS4 PARN PCK1 CST3 ATF7IP CD1C IL6ST PUF60 SOX11 MAPT CAPN1 ENSA APBB1IP COL5A1 ZBTB34 E2F5 APELA LCP1 FGF1 SSR2 ERBB3 GPR89B PTDSS1 LIFR PIK3R5 FAM76A TRIM59 MYH1A TSPAN1 MRPS26 DIO3 MTMR2 OAT IGSF1 PARP1 GNG2 RGS9BP DCUN1D1 POU1F1 RNF7 USP15 IMMP2L TPRA1 RAB11A SH3BP2 CDCA4 SKP2 CPQ FAM45A BORCS5 FBXO8 ANGPTL2 CASP14 COL6A3 PPHLN1 LUZP2 NEK7 ST6GALNAC2 5-Sep BRINP1 TXNRD1 SERPINE2 ACBD3 LEF1 JAM2 FGF10 ACSBG2 STK17A ACAN ZDHHC21 RGS17 WWP2 DYNC1LI2 HAS2 TBC1D23 TSKU PIK3CA COL22A1 PCDHGC3 ZNF512B TMEM120B SLC46A3 RHOA ZFAND6 RNF20 AP2M1 FAM49A ANXA11 F2 ATP6V0E1 CDKN1B KDR ABCA1 GALNT1 SCIN ABCF2 VPS41 WRB CCDC18 BASP1 ZFYVE19 MEPE ANKRD26 AC113404.1 GDPD5 COPE MYH11 ZNF767 ARFGAP2 PAQR8 LOC424740 FAM105A STUB1 ADARB1 MAOA HDAC4 PDE3B UQCRH LOC426914 HACD3 RGS4 NGF GNB1L CD36 CD200R1L ELF1 LUM RLIM CRYAA BET1L BORCS7 PRNP FYTTD1 CD44 RASSF5 LOC422926 GLP2R LOC100859039 NAA50 FZR1 LYRM1 HERC3 MTPN C1H7ORF60 CD247 E2F4 ARHGAP25 HMG20A TMEM41B TNKS2 HDX HERC2 BRAP NUDT5 CHRDL1 S100A1 VCAN REL SRPR SMIM12 QRICH1 PPP2R4 EDNRB2 HYAL6 AC025048.1 STX2 KIF5C USP6NL RFC1 CLOCK HMGA1 CNPPD1 PLA2G7 NR1H3 ZW10 BRD2 KIF18B TEX264 AC005943.2 SEPSECS ELK3 ATP6V0D2 CRYL1 GLOD4 MRPL45 RAB32 MMP13 POFUT1 GOLGA7 CREB1 CNTN1 HDAC7 IL16 DCAF7 ARHGAP26 RP11-403P17.5 ORC5 MPZL2 S100B IGFBP4 FGFR1OP2 PKM BBS2 SSR1 PDDC1 SLCO4A1 NAIF1 GINM1 TEAD4 ANP32B RUNDC3B ALDH1A1 ANKRD27 POPDC3 FGFRL1 CDC42SE2 VIT CNGA3 FZD3 PIK3CB TMEM104 PRLH WDFY2 SCOC PTPRO CSNK1D BSG ARFGAP1 VTN RNF4 RGS16 RARRES2 CENPF CDH5 IRF4 TLR21 GALNT6 TSC22D3 TRABD FAM20B RBPMS2 AHCYL1 NBL1 FOXC1 GLRX UGGT2 TMEM170A SPP2 GBAS GOSR1 CDC2L1 CTSB BLOC1S2 SUMO3 CSPG5 FOCAD NAP1L4 HPCAL1 C26H6ORF106 CTNNA2 C5H14ORF166 CCDC93 BHLHE40 GNOT1 EDF1 SH3BP5 SEPT2L FMOD UGDH RP11-49K24.9 ZFP92 COL18A1 CCDC61 RNF141 E2F1 YRK SLC17A9 RAF1 COL17A1 XIAP DDA1 BIK RNF25 LOC100859427 LPP SUPT3H C1QTNF2 FMO3 TMC2 APCDD1 CANT1 IAH1 PNPLA6 CNTN2 RP11-834C11.12 NOTCH2 HABP4 FBXL21 ZP2 NR2C1 TANGO2 IRF7 MAP1LC3B ESRP2 EGFL7 CANX ORMDL2 PKIG MAP6 CMTM7 MSN RP11-145E5.5 SERPINH1 SNCG CPSF6 GNG5 PIGR RAB3GAP2 MFGE8 C1GALT1 ARFIP1 TYRO3 LOC396531 IPO13 CENPH TCEB1 FOS HPGDS FOXN2 WDR45B DYM CYP8B1 USP45 AGO4 SLC39A13 C2ORF88 LOC100859249 CUEDC2 FASN ELOVL1 RAB33B CD200 CAPZA1 SLC1A2 PTP4A1 TDRD7 RFC2 BAK1 SMPX SSTR2 SNRNP200 NINJ1 GTDC1 MVB12B SYNM BRD8 HOPX FAM126A HIC1 LOC396009 GIGYF2 CLPX STAU2 S100A10 CDH20 KLHL18 GSTO1 G3BP1 CKMT2 CHRM3 TNC ST3GAL3 TAPT1 ADD1 ARF1 SLC19A1 VPS53 P4HA2 MAPRE1 EYA4 ANK3 INO80 CCR5 PDHX TERF1 AAMDC RNF13 DEPDC6 RASGEF1C PLEKHM1 CREM AANAT DNAJB6 TNFRSF19 NUP93 PHTF2 RP11-5A19.5 SNX24 C26H6ORF89 CDC42 UGT8 SOCS3 AIDA C4H4ORF29 TFEB ASIP CAB39L KIAA1671 SATB1 CD80 ENTPD8 CHD2 LCLAT1 PLEKHB1 CHORDC1 FBXW11 FUNDC1 LOC426385 TXLNB KTN1 NECAB3 DEXI BUB1 MYLK2 LYRM9 DCX EXOC6 H1F0 CAT NEIL1 KIAA1467 MPP5 FRZB TBK1 TBL1X SLC25A26 LOC428335 PRC1 RPS14 RFXANK PRR5 CD74 GPATCH2 AKT1 XRCC6BP1 XIRP1 UCHL5 LOC408038 SGK196 CD3E ST3GAL2 NDUFB2 AGMAT BRMS1L NUMB RFNG ACP2 IGHMBP2 RHOC PLAG1 PAPD7 CSDE1 SLC26A5 BID NECAP2 ERI1 SLC26A6 SF3A1 TCF15 C1H12ORF23 COL8A1 FBXL16 GAD1 CLDN3 NFYA SELO FHIT RABGAP1L NRTN SET DDX42 PCGF2 THYN1 RBM5 BAZ2B PQLC2 NEK6 TTL NME1 HCLS1 DAAM1 TMEM184C TNFAIP6 STK11 AKTIP NR5A1 MYD88 ST13 MON2 ERH PHC1 PTBP1 MMACHC BTBD10 SERPINA1 GEMIN2 NT5C2 RASGRP3 TAF3 TRAF5 RPRD1A AKAP2 TMEM230 MGME1 SUGP1 PSMD14 SPATS2L ZCRB1 PRIMPOL ADCK3 MYO1C CRYBB1 PRKCD DNAJB12 KLHL13 ALAD CDC25A EPGN DOLPP1 KLHL20 AvBD4 NCAN SENP8 NGEF CELF1 EIF6 YWHAB C11H16ORF70 MYL9 LIN9 ETV6 CPNE1 TBL1XR1 GJA1 LOC770548 PACSIN2 CDH7 FLT4 SZRD1 JAK1 SRGAP1 DRAXIN CHRNB2 IFNAR1 STK11IP MTMR3 RBM25 WASH1 MTX3 CRTAP N4BP2L2 CBLN4 IL2RA MPC1 PDGFB BRSK2 RANGAP1 TRPV4 SMIM5 FHL5 YPEL5 TXNDC5 FANCC GAPVD1 UBE2G1 FRS2 HDAC9 APLF DCK PEX2 NUP50 C15H12ORF49 MCM3 AMPD3 ZNF410 RREB1 FLNB 8-Mar TRIB2 CLIP1 CCKAR MORN4 PELI1 ITGB1 SETD3 GCLM LOC100859722 TMEM57 SLC25A22 TADA2A FBXO34 C14H17ORF103 POLDIP3 ERLIN1 CIR1 FKBP8 CNTN5 AMIGO2 PSPH RP1-309K20.6 DYNC1I2 PBX3 CPA6 SYNGR3 ULK3 FAM118B RBM19 HIBCH NME5 ATP6AP1 ALDH1A3 SALL4 YPEL2 TRMU CTGF CSRP2 VIP KLHL24 GJC2 WBP2 PDPK1 PODXL RAG2 PIP4K2A NREP EXOC5 RGS6 MYEOV2 CHADL CDH17 NFIX NECAB1 STRBP MBD3 SBNO1 CPT1A TP53I11 FAM213A ITGB2 CXCL14 PARP4 POT1 MGAT3 SRC ADCK1 MLX 9-Sep MFAP3 B3GAT1 DHRS11 TIPIN OSTN LOC420419 FYB PIAS2 RBM22 NCALD TSPAN3 NRXN1 FABP6 LEPROT TWIST2 POPDC2 PAFAH1B1 JMJD6 PFN2 CTD-2370N5.3 CDH6 USP37 PCMTD1 GFOD2 RDH10 SYK SPCS1 FAM222B LDHA ETFDH MGEA5 C7H2ORF69 PPM1M WFDC1 SLC16A3 DMTF1 MKRN2 SLC9A4 BRE MAP3K14 NFIL3 RFTN1 MTMR8 VDHAP EDC3 SEMA7A TMOD4 PLEKHO1 RALGPS2 FDFT1 CMKLR1 RAB10 RALGAPB FAT3 |
| gga-miR-144-5p | SETD3 POPDC2 SLX4IP PKIG CPA6 CNTRL CDKN1B CSNK2A2 SPINZ SDF4 FHL5 STARD4 TMPO SGPL1 ABHD13 RHOG SGK196 CENPH ATP6V0E1 RALBP1 KLHL18 FAM105A IMPG1 IRF1 RABL3 BLOC1S6 CIR1 SNN SLC26A5 AR SYTL1 PRKRIP1 IRF4 GPRIN2 TTL SGMS2 POT1 ARF1 H1F0 JAK1 TMEM184B YTHDF3 PPP6R3 CHRDL1 LOC419112 ZCCHC17 GDPD5 FAM126A PLIN1 SEMA7A LMX1A ACOT9 RPL15 TMEM120B PDGFRA GJA1 A4GALT RRAGC PTN PAFAH1B1 LYSMD3 FUT9 8-Mar TNFRSF19 STAU2 DHRS3 WASH1 FYB PDE6H CMTM7 PEX2 ZFHX3 GJA5 GREM1 NT5C2 ACADSB NRN1 MTFR1 CSRP3 LYRM9 BRAP VPS41 ZNF767 FLI1 SMPDL3B UBXN2A CCL1 GNB1L DPF3 ARFGAP1 ART1 NFRKB TMEM123 CST3 PAQR8 VIT CHRM3 PPP4R2 PIK3R5 LOC427470 BAZ2B GLRX CANX KCTD7 LCLAT1 NOC2L ASIP TPST2 IVD ZC3H14 FBXL16 RELL1 EGR1 HDAC9 EGFL7 TMEM167A ALAD CDV3 RGS17 PACSIN2 CECR1 CDPF1 GALNT1 BHLHE40 MGEA5 RASGRP3 SLC17A5 MTMR8 DNAJC5 SMIM18 ATG9A BID PIGR PPP1R12B S100A10 LEPROT NECAP2 EPYC STX2 COL22A1 CDH6 FAM49A CCPG1 PCM1 RALGPS2 OPN5L1 CUEDC2 IMMP1L USP6NL RBM5 KLHL13 SUB1 NOTCH1 FOXO1 TFEB RBM12B HNRNPD AGTPBP1 POLE3 TBX18 FAM222B SELO NEK6 VCAN IL5RA PRIMPOL HDGFRP2 CNTN5 GTDC1 FBXO34 THOC3 LPP HAS2 CTSB COL8A1 CDH20 ARIH1 ALG12 ZDHHC21 NRXN1 KPNA1 SOCS2 PLCXD1 FBXO8 SNCB IKBKB INO80 SPRY2 ROR1 UBE2G1 NECAB3 PDHX GTF2H4 DIO1 MAGI3 PDE3B MTPN PSME4 CACNA2D1 ZNF639 TRAPPC3 ANP32B RGS6 TMX4 MX1 C4H4ORF29 CLIP1 FAM45A OVALY LOC416354 CTXN3 CD151 NAV3 WIPF1 ACVR1 SLC15A1 EIF4G2 PTPRO HPGDS KERA AMIGO2 KBTBD4 RAF1 NAA50 TBL1XR1 DDA1 MIER1 IL16 GAPVD1 RP11-403P17.5 PHF21A AGO4 CD72 RFTN1 PLEKHO1 RTKN2 SSB SNX16 RPS27L CTD-2410N18.5 ITGB2 N4BP2L2 LUZP2 FAM177A1 TPH1 JMJD6 TMEM175 FZD4 MRPL20 AKAP2 TMEM129 CREB1 CAMK2D PTRF BRINP1 NAP1L4 RREB1 DEXI DROSHA SOCS4 PCMTD1 VMO1 B3GNT5 USP45 CUL2 NADK SUPT3H CLTB SCD ALG6 CHD7 STX6 RORA PRDX1 APELA SLC1A2 ACAN LOC418811 MRPS14 FGF1 CRIPT ETV6 KLF6 PYGB SRPR EDEM1 CETN2 B3GAT1 NKRF TAOK3 ATOX1 FHIT PPME1 PRLR DAP ST3GAL3 RP11-20I23.1 FABP6 SPATS2L MAOA NREP MAP2K1 TXLNB RBM19 RPIA CSRP2BP MCEE CHP1 CNTN2 BUB1 SH3BP2 SGCB PNO1 KIF5C TP53I11 LFNG TRABD FAT3 FANCC NCOA1 NFIL3 ALDH1A1 RASA3 KIAA1671 GPR89B GATA3 ENTPD8 SNRNP200 ACSL1 UGT8 NELFA SEPT2L IL6ST RINT1 RGS4 GINS1 TNFRSF18 MAL2 EXOC6 FAS CTDSPL GSKIP SBNO1 GFPT1 RFNG CIDEC SOCS6 PLEKHA2 ACAA2 RNF152 FZR1 PAFAH1B2 TMEM56 ABHD17B AREG PPAT CAB39L APCDD1 ELOVL6 APBB1IP NUP93 BORCS7 SDK1 COL9A2 CNP SYNCRIP CCR5 GEMIN2 BTD NCALD RP1-309K20.6 MYH11 RSL24D1 TNFAIP8L1 MTMR3 PGR CAMKMT LOC424740 ASNSD1 PIGA ANK3 MYO1C P4HA1 GJC2 MSN CDC42SE2 DNTT LCP1 CHD2 ZDHHC17 GPATCH2 CCDC167 SERPINI1 REL NFASC SULT1B1 PIK3CB PSPH KDR KTN1 PRKCI NGF PRRG1 TWIST2 MPP5 PCK1 QDPR PDLIM5 SPPL2B MAP6 EYA4 HIPK3 LIFR MYOM2 MAPK6 C1H2ORF49 RNF13 TEAD1 OLA1 CHST10 SCNN1A BRSK2 STK10 TSPAN3 RAB8A FAM214A CD44 NGEF SPPL2A CDKN1A CNIH1 RBP4B CRTAP UBXN10 CD99 SATB1 CTNNA2 ELF1 ZFAND6 BASP1 SLC25A15 ABLIM1 TNC RNF11 FAR1 ETFDH NR2C1 ADARB1 WDR1 G3BP1 STRBP PFN2 LYRM2 KRIT1 CTDSPL2 ADD1 THADA MIB2 RPL9 CD200R1L BBS2 LOC417800 HMGN3 CSPG5 SF3A1 TOLLIP MAPT IKZF5 TEAD4 SERPINA1 ULK3 FBXW11 LEF1 TNS3 LOC100859616 INTS2 RASGEF1C LOC431499 NAGA SERPINA4 TAPT1 HAO1 TBL1X SMPX TRMU ORC3 HIC2 LIMD2 SET LMBRD2 TRIB2 MGAT4C ACACA SMIM5 |
| gga-miR-193a-3p | GBX2 SH3BP2 TNFAIP8L1 SGTA SGCB RPIA PIK3R5 TRPM7 CABP2 C2ORF88 LOC431499 CREM GLUL VTN STMN1 TPRA1 BORA POPDC2 ADIPOR2 FMOD LBFABP VNN1 BTF3L4 RECQL WASL XIRP1 MYD88 BID PEX11G PRKAR1A SCP2 DCUN1D1 SPPL2A NRP1 KCNA4 ANKRA2 ZDHHC18 TPM1 FOXD1 ANGPTL2 KBP SMIM5 MAP6 CDH20 RTN1 MTPN IGF2 YWHAB CPN1 HSPH1 CREB1 ALAD SLC9A3R1 RNF13 LPAR4 NAA50 API5 KCNH6 ELMO3 FABP6 SRSF1 SNAP91 SLC16A1 RGL1 SERPINE2 MTX3 CANT1 PPP1R2 NUDT5 DAZAP1 HAVCR1 CALCRL EIF5A2 MYH11 BRSK2 ENTPD1 CSRP2 SLC17A5 DYNLT3 QRICH1 PRNP BORCS5 FAM126A RCAN3 ACAN TLN1 LFNG FAM46C OVALX CD8B VCAN APOC3 DCAF7 HOXB1 C4H4ORF29 FAM45A RBM5 PIGY GGNBP2 INTS7 NINJ1 ZNF384 ORAI2 ESRP2 PLAG1 MAGI2 TEX264 EDNRB VSX2 B3GAT1 ARFGAP2 MSANTD2 ELAVL1 PUF60 PRRX2 FHIT ENTPD2 OTUD6B IL6ST MYL12A PHTF2 NUSAP1 CIDEC SGK1 LOC100502566 SZRD1 RGS16 POLE3 SEC22B EXT2 CLSTN1 FEN1 YPEL5 CD5 CTNNB1 FYB SLC38A4 GPBP1L1 PHF21A WWP2 DR1 RNF141 AMIGO2 FDFT1 CBLN2 CLCN7 CAT NOTCH2 DCLRE1B VAMP7 5-Sep AKAP2 FBXO34 PFKM TNNT3 LOC769486 RRAD NUP50 CDC27 TRAF5 GFPT1 GLI1 RAE1 ZCCHC6 TSPAN6 PDLIM5 RIPK1 CREBL2 HOXD8 NGEF PPP1R8 ZP2 PKIG FZD5 RRP1B CTSS SRSF2 DDT FZR1 ARF1 CSRP3 SERPINB10 CBX3 DLX6 SUPT3H APPBP2 CSNK1E ANXA7 ACP2 PLIN2 COL8A1 PRLR WASH1 TF CISD1 C15H12ORF49 SERHL2 PTN SPG21 XPO5 RPL32 GCC1 N4BP1 RALBP1 RBBP4 GNPTG DDX47 TNS3 PAN2 GPATCH2 NEK7 C1H2ORF49 HAS2 PDLIM7 ITGB2 PLS3 ETS2 ST3GAL2 JAK2 CLEC3A ACOT9 PIK3CD CD82 SH3BP5 ZNF330 KLHL13 PPP2R5C EYA4 HSPA4L CNOT7 GLP2R CLPX VIT EAF2 FBXL16 SNX12 ZW10 ZDHHC17 SLC35E3 BMP15 FPGT TOX3 CANX SEPT2L GMPR PDGFD FLOT2 SSRP1 COQ5 BPGM SDK1 MET ATG9A FHL5 PFN2 DUT PLEKHO1 DNASE1 RBPMS2 RAB14 STX6 POFUT1 PPP6R3 SLC19A1 SLC2A2 DPH1 USP45 GLT1D1 BAK1 DFNA5 TLDC1 LOC429492 CCNE1 SLC7A6OS RAG2 TBL1X EXOC2 ATF7IP AR SLC26A5 GLRX5 PSME3 C14H17ORF103 PSMD14 CD200 GNB1L AHR CINP ECE1 CDCA7L ADCK1 EDN2 AHCYL1 ICMT ERLIN1 HCLS1 CDCA4 F2 PLEKHB2 EIF6 LYRM9 8-Mar CHST10 KLHDC2 STIM1 PRC1 AGO1 AP2M1 KCNJ8 PDS5B DCBLD2 ATP6V0E2 TAF8 MGAT3 BLOC1S6 CNR1 BEAN1 CD72 RECQL5 MIB2 TMEM254 FAM222B UBE2V2 IRF8 CD80 NOC2L A4GALT ANXA11 GAD1 RFNG GNG2 RREB1 RGS19 VPS45 DLD YTHDF1 C1QTNF2 GAS2 SMIM3 SULT1B1 LEF1 SLC8A3 MCCC1 TNFRSF19 KIF3A BIK PRPSAP2 PPARD ST3GAL3 RBM25 TGFBR3 PAK1 SLC25A4 DEAF1 LUM DNAJC12 OSBPL2 DNAJC6 MED9 ZFHX3 YWHAQ MCL1 CDK6 TMEM230 CAMK2D ADD1 ORC1 PSEN2 HDAC7 SLC6A9 LCMT1 PRRG1 SET ACLY RPL31 TIAM2 EXOC5 TMEM173 TCEB1 RIC8A ZCCHC17 C10H15ORF61 ARIH1 PALM NFRKB STARD4 RAD21 HIPK3 NR1H3 SLC26A6 CTBP1 PLCXD1 PUS7 CD44 RBM12B ANKRD27 XKR8 SLC47A1 ENO2 FAM49A PDE6H BRAP SGMS2 RPL39 SPPL2B NCOA1 CMKLR1 STAT1 GDPD5 YPEL2 LOC396479 SLC9A2 TSKU SETD3 CSPG5 ERGIC2 ORC3 CRTAP QARS NPPC GJA5 MPPED2 CASQ2 FAM175B CTNNA2 TOP1 ODC1 SERPINB6 SEPSECS IRAK4 PTP4A1 NCALD CLDND1 IKZF5 ETV6 UGT8 CYTH1 CHUNK-1 SLC38A2 MGLL HABP2 RP4-613B23.5 KTN1 NOP56 FBXW5 NCAN RAC2 DNAL4 TRAFD1 LOC100858447 GLRX DNAJC5 MYO1C GTDC1 PLAC8L1 SARS RNF185 TRAPPC2 MPZL2 KCTD7 MTMR3 SRSF5A TP53INP1 TSSC4 SLC16A3 RUVBL1 PAX6 IRF7 AQP9 CDH7 RB1 TLR21 REXO2 FLI1 NDUFA5 PYGB MAL2 NAV3 S100B ELAVL4 NUDT19 PARN SLC39A13 GPALPP1 UBE2G2 CDK10 BET1L PPFIBP1 CNP PNAT3 FEM1B PIGR ALCAM EMB PPHLN1 TMEM56 ELOVL1 CACNA1S LYPLA2 C1D VPS4B CAB39L PDPK1 LOC693265 GSTT1 KCTD2 CHD2 AP1G1 GABRB3 PIK3CA TMIGD1 XPA TMED5 HIBCH TARDBP DHFR LOC419851 EIF1AY EMP1 CHM GNB1 RALGAPB NAE1 TRAPPC3 MTMR8 ZFP92 LSM14A NPL CR1L ZYX SYT12 MAP1LC3B HIC2 STK11IP SLC35B1 STK11 CPA6 PPP2R2D SGPL1 ZCRB1 CHRDL1 EGFL7 MAPK6 EIF4G2 TXNRD1 QSER1 CECR1 TWIST2 ENS-1 COX17 CUEDC2 PTDSS1 LOC770639 UBN1 TANK GAPVD1 IGHMBP2 CSNK1D CNOT2 EPN2 PSEN1 PROM1 NRN1 IFNAR1 SFTPA1 PFKP KCNN2 MALL IRX4 MTMR2 PELI1 BCAP29 WDR18 DPF3 PAPD7 YEATS4 CD200R1L GALNT1 VPS41 TAF12 FAM76A DMD MGP CITED4 OLIG2 TOB2 JMJD4 N4BP2L2 AP3M1 NDE1 PAFAH1B2 MSN C1GALT1 ST3GAL1 INHBA TRPC3 ATP5G3 RHOC TRIM59 SOCS2 VPS53 NEK6 ZNF622 SLC46A3 EXOC6 IL16 NOS2 APOD RAB3GAP2 WDSUB1 ST6GAL1 MTHFS NAPB FAM105A RBM24 HSPA8 OVAL DNAJB6 PIK3CB NTMT1 TNR CD151 ACE DKC1 PLEKHM1 NRTN FGB RFXANK NDP VDAC2 BCL2L1 GPR89B DYM RP11-403P17.5 CETP SRGAP1 MYO1G TARSL2 NKAP CUX1 FGFBP2 FUT9 TMED3 APOV1 TMED8 CRYBB1 FANCC TMEM229B COL22A1 CASP14 BASP1 MRPS26 ACTB SRF LGALSL AMPD3 CNBP FAM122A RAP1GDS1 PSMA5 DNAJA1 SREBF1 K123 MAP3K14 PSPH ABHD13 PTPRA NEUROG1 LOC424740 GCM1 TEAD4 SATB1 HMG20A FGF10 DAP ALDOC GJA1 RP11-20I23.1 STRADA VAC14 MT4 PRKRIP1 AvBD1 PLA2R1 CARS ANP32B TTL NUDC WFDC1 JMJD6 ACBD3 PREPL AIDA KLHL2 FBLN5 GAPDH CLIP1 SLC1A2 P4HA1 HERC3 H3F3B SRSF3 CRYBB3 RFTN1 PEX13 AKT1 BTC PTPN6 ADCK3 YWHAZ TMEM170A BBS4 CDC42SE2 FAR1 SGMS1 SMIM18 ALDH1A3 STRBP CDH8 BTBD1 RAD54L GID8 CDH5 SMPX TRPC4AP UFSP2 RHOA GCLM PPP1R3E SYNM TAPBPL ALDH3A2 HPGDS C7H2ORF69 CTSA ID2 RAB35 RPS14 ARHGAP8 OPN5L1 TMEM30A GART CEBPG DYNLRB2 CUTC AZIN1 ABCA1 MIER1 TRIM71 SNRNP200 FAT3 PTPRU ATP6V0E1 DCN PCGF2 STX17 VGLL2 AGMAT STUB1 MPC1 PRKCI ARHGDIA ST3GAL5 PLA2G7 RABGAP1L YRK YY1 MAOA TMEM180 PEX2 DAD1 CCK PSMD3 PCMTD1 SNX10 TSPAN1 C26H6ORF89 SPATS2L GATA4 RLIM SCPEP1 XPO1 CSF3R STX2 PIGK NGF MON2 ATM SMIM19 YIPF3 HRAS PPM1B GUCA1A CAPN1 PDK4 NR3C2 TLR4 C13H5ORF15 MATR3 ZFP64 TMLHE MUM1 SLIT1 HDAC4 STAT4 NR2C1 GORASP1 GATAD2A USP6NL RASSF5 RGS17 PPME1 RAB24 LOC419429 VPS33B FAM213A NSMCE2 ESR2 G3BP1 PTGS2 LCP1 SCG5 CRYAA NECAB1 DMRT1 DHCR7 FBXO8 EDA2R RP11-49K24.9 TNNI2 HMGCS1 MYH1A IMMP1L JUP GLT8D2 UNG BHLHE22 NT5C2 CDPF1 GET4 ITGB1BP3 LOC421975 MORN4 TMEM120B RNF14 LOC420419 TMOD3 KPNA1 MPP5 DMTF1 TRA2A NME3 SLC20A2 PAPOLA MXI1 RRAGC LOC396531 ATP5I NHP2 SERPINI1 POPDC3 ZNF512B CMTM7 ASIP STK32A EDF1 ZDHHC21 PDGFB MAPKAP1 MKKS HYAL6 PSMC6 RORA RCHY1 ARL6 PABPC1 RTN4 TYRO3 MBD3 E2F4 CDH6 MYL9 STAT5B BLOC1S5 MST4 CUL2 RNF220 BRINP1 FTH1 FOXO1 FGF14 NELFA ALG12 FADS2 KIAA1191 DAAM1 CENPO STK10 ARFGAP1 RAP1GAP2 TDRD7 CLOCK PARK7 UBIAD1 ZNF706 MYH1C CHP1 RASA3 TMEM68 TSPAN3 F2RL1 CRYL1 HPRT1 SYTL1 KCND2 FGFR2 CTDSPL2 SNX16 DDX42 UBE2G1 SSTR2 CXCR7 GHRH NFAT5 PPP2R4 UQCRFS1 SFR1 RAB11A LPP TLE4 AANAT RALGPS2 ZIC1 ENO1 MSRB3 SOUL STAU2 SMPDL3B CYB5A PARPBP T GABPA CD86 CNTN5 SDCBP LCLAT1 C1H11ORF54 CDC42BPA GFI1B PAQR8 USMG5 ATP1B1 PPP2R2A TOP3A ANK3 WWOX PDGFRA FSTL1 LAMP1 STXBP3 S100A10 NAP1L4 ZBTB17 RNF152 LIFR AGPAT9 NBN FGFR1OP2 RPS6KB1 NFIL3 TMEM121 OVALY SRPR RP11-196G11.1 FGF1 LMO4 6-Sep COL17A1 REL FOCAD NECAB3 OSTN UBAC1 CYP1C1 LIN52 WBP4 CPZ ADA KIAA1467 RRP7A ELK3 HMGA1 SYNGR3 ARRDC1 TMEM104 JAK1 BRE RBMX NEURL1 MAL PTK7 ARHGAP19 PAFAH1B1 CDC25A TBK1 SLC41A2 BHLHE40 EHMT1 EIF2S1 FSHR PAAF1 TMEM208 ACSL1 CCNA2 STX16 LOC770684 MEAF6 SRSF5 MOB4 AC113404.1 SERTAD2 FOXA2 ACVR1 LIMD2 GOLGA7 ARFIP1 KIF3B DLST PHKG1 COL4A1 SBNO1 THY1 RBM19 FBXL21 RASGRP3 MGEA5 DEXI BCL10 DHRS11 RAB5A ZBTB2 ARNTL FAM177A1 LOC776816 NPC2 NCSTN UGP2 XIAP SAP130 CTCF PCSK7 PKM LUZP2 MBLAC2 ROR1 RHOBTB1 PPP1R16B IL8L1 TBL1XR1 LEPROT CCR5 GMFB ADPRH KERA ELF1 LOC408038 ALDH1A1 TMEM70 VPS35 TEAD1 NRXN1 FLNB ENSA ENOX2 RFFL HIST1H3H CPNE1 LMX1A CMPK1 TNC CORO7 DNAJB12 CALM1 KLHL24 AGTPBP1 TSEN2 CKB PMS1 PDP1 APELA ZFAND6 DUSP10 BAZ2B TBX22 SAMM50 TP53I11 PHACTR1 SLX4IP PTGES3 TRABD CNGA3 MVB12B OTX2 CPT1A KLHL18 HHATL DDX10 C1H12ORF23 EYA2 TACC3 SNCB GLI2 MARCKSL1 HAPLN1 ULK3 GALK2 TAF7 PRLH SSU72 TRIAP1 COG4 ST6GALNAC1 CHRM3 RAB9A PACSIN2 CTTN TMEM123 KIF5C FXYD6 PAIP2 TBP DHRS3 TLR1A RHOG RAD52 TBC1D1 ACAP2 TFEB |
| gga-miR-199b | HNMT ELOVL4 EDA2R RAB32 RP3-461F17.3 HMGB2 DENR MIB2 PKIG UCK2 SPATA2 TMEM230 ACP2 PSPH GANC GALNT1 GPR89B MYO1C GCM1 ZMYND19 GNPTG CD276 PPFIBP1 NEURL1 KIAA1143 MICAL1 POLE3 UBE2G1 CACNG3 MSRB3 PDS5B FLNB COL22A1 UBE2V1 PGR SOUL KCTD14 RFFL C10H15ORF59 CNGA3 NRBP1 LYVE1 AP1G1 MAVS KCNMB4 CPT2 GCLM NT5C2 CD151 RNF20 ACE CTNNA2 CD80 ROM1 IPPK DAAM1 UCHL5 PREP EXOC3 BAK1 EDNRA PRKAA2 GNG2 GTF2H4 UBXN10 PPP1R9B MTHFS HARS PARK7 TAF8 PTGES3 TP53I11 C1GALT1 TWIST2 SLC2A2 RTN4 FAM102A HMGCR ALDOC RFC2 5-Sep RUNDC3B TNNI3K CRADD TRAF7 PPP1R3E ACVR1 WSB1 AANAT GARNL3 A4GALT ITPK1 RHOG COL6A1 ARHGAP26 CPA6 BORA PDCD6IP ACOT9 VDAC2 HYAL6 CD86 MAGI3 RNF34 NOP56 NAV3 EIF6 DHRS7C PNO1 NUDC RS1 NEUROD4 CHD2 RP11-403P17.5 TGFBR1 DNAJC5 FGF1 TNS3 TMEM120B DNAL4 BTBD1 DYNLL2 PTPRZ1 CELF1 ORMDL2 FLT4 LOC769121 MAGI2 FOS BCS1L ARFGAP1 TEAD1 ARL14EP CDKN1A OSTM1 S100Z PIGR RALBP1 SERINC1 8-Mar ST3GAL1 EIF4A3 CAPZA1 PCMT1 GLCCI1 SGTB KLHDC2 C26H6ORF106 ARF1 DEXI THOC3 PUF60 ORC1 BCAP29 MORN4 FGFRL1 AREG NR1H3 SDR16C5 PHACTR1 KIF2A EARS2 ERG PXN SNRNP200 LEPROT TOLLIP TARDBP TNFSF10 ZNF706 DNAJA1 ACSBG2 TBC1D1 PYGB TXLNB GNOT1 EGFR RAB33B CDX4 NAIF1 TBC1D22A DUT MBL2 SSTR2 GLRX VTN BIK SEMA7A BFSP1 KIF20A TMEM167A CAPN3 DYNC1I2 CD36 TNFAIP8L1 PNAT10 AC113404.1 PACSIN3 FAM98A TMEM254 HRAS TIMM9 TPRA1 LOC418667 KIF3B NPAS2 ZFP92 HPGDS C7H2ORF76 GLYR1 LOC776816 CETN1 CA2 WASH1 DNAJB12 SDK1 CLTB AP2M1 SET BAZ2B TLN1 CBY1 TNFRSF19 SOX10 AMY1A TNFRSF1A WIPF1 ERI1 CRIPT LOC770548 ARFGAP3 C18ORF42 B3GNT2 RBM12 CHTF8 FSHB NAA50 TRH ENOX2 PYGO1 RMI1 KIAA1671 PEX2 DDX47 STAR CHMP4C GPR171 TRIM59 RP1-309K20.6 LOC772096 PGM1 SZT2 POLK RB1 KLHL13 HIC1 PDLIM7 PRC1 FBXL16 NFASC MTMR3 PQLC2 DNASE1 SGK196 ODC1 TRAPPC3 DHRS3 NELFA RAP1GAP2 PHTF2 IMPG1 XPO5 SFRP1 C14H17ORF103 ST3GAL2 SMIM19 GAPVD1 SMCO4 STARD4 ERLIN1 CNTN5 NUDT5 MYL3 HIBCH NREP CPT1A MCM3 UBIAD1 GID8 RBMX NINJ1 OSTN FHL5 DYM SERPINA1 TCEANC2 ZDHHC21 NDEL1 ARRDC1 RHOT1 DEK TBK1 LCMT1 RELL1 AAR2 RBM5 ALKBH3 ARL6 SALL1 BTF3L4 POU1F1 THRSP RPL31 TADA1 LAMTOR3 TNFAIP1 TMEM175 EPCAM CREM DACH1 WDR92 NAA35 SLC38A4 NDP PRKAR1A AMIGO2 TMLHE P4HA1 CITED4 CBX3 TOP3A ACADSB TSPAN6 ALDH1A2 SCIN SNX12 FZR1 MCMBP FPGT RIPK1 CDH20 PPARD PRKCI SERTAD2 CCDC93 CPSF6 PAX6 ATP6V0B SEPW1 COL6A3 MEF2BNB GLI2 CDKN1B MAPT GATAD2A PAPD7 RABL2B CPNE1 RNF13 UGGT2 SETD3 BTG4 PLCXD1 RNASEL TRABD PLEKHB1 OPN1MSW CYP8B1 STRBP CDH1 MAT1A ADORA3 VCAN MUM1 S100B FUT9 WWP2 YTHDC1 G3BP1 AARS2 ANK3 TGFBR3 WBP2 SMIM14 OPN5L1 NRTN WFDC1 NR5A1 EXOC6 CSPG5 PCGF5 IFT140 LOC421975 SLX4IP SLC15A1 CUEDC2 GDPD5 SGK1 ST3GAL3 KIAA0020 AGA PRKCD MET ESF1 SDC2 DMTF1 NUP50 POPDC3 APCDD1 RNH1 TERF1 GLOD4 CTBP1 ORAI2 RALGPS2 CASP18 VGLL2 TOB2 PIK3R5 ADAT1 ID1 RAF1 ANKRD27 C13H5ORF15 ZYX WDR24 PEX11G MFAP3 WDSUB1 FAM18B1 LCLAT1 PSMD14 CASP14 SGPL1 COL9A2 GCC1 CUTC GHRH PUS7 PDPK1 GEMIN2 EPN2 PRPF38A RPRD1A CYP1A4 GINS1 P4HA2 PPP2R2D PIK3CB LYRM9 GFPT1 HOOK1 LOC429492 MRPS16 CYP2C45 JMJD6 GREM1 LDLRAD4 LUZP2 ORC3 IFNGR1 SSU72 ARIH1 BIN1 GJB1 SPATS2L RPS27L SMIM4 MBLAC2 GNG5 ALDH3A2 CPSF4 ADD1 APBB1IP MYH10 TMEM104 CBX4 ABHD17B RBM48 ETV6 BSG CTDSPL PTPN11 AR LOC431325 IKZF5 HAO1 RCAN3 DDX6 FOCAD GTF2E2 ZCRB1 MAOA CREB1 CDH7 HDAC4 KIF4A GIGYF2 LOC396531 SYT12 PLEKHF2 C7H2ORF69 KLHL2 CLIC2 TP53INP1 CMTM7 C3H6ORF203 GNAL HMG20A KPNA1 ADARB1 TXNDC5 ACACA SMIM18 AC025048.1 P2RX5 F2 STX17 CKS2 SERHL2 HMGCS1 RNF111 RAB3GAP2 SNN MYOM2 ARHGDIA CNR1 CACNG1 RORA SLC31A2 KLHL15 RAD51 AZIN1 UTS2B EIF1 PSME4 SMPX SLC16A3 VPS50 MAL2 C11H19ORF12 PPME1 KIF5C CHST10 IGFBP4 CDH17 COL5A1 CYP46A1 EPT1 FBXO8 VDAC1 HMGN3 CMKLR1 BHLHE40 CISD1 LOC769139 TRIB2 RAD52 MAD2L2 YPEL2 KIAA1467 ANXA11 TPST2 ASB9 FAM65B EXOC5 MXI1 FHIT SOCS4 PAIP2 YPEL5 OAT VNN1 IRF5 LUC7L3 DESI2 NAP1L4 EHMT1 FYB SLC47A1 ELL PBRM1 TEAD4 DPM2 PPP1R21 PKM PPP1R8 GTF3C5 POFUT1 PELI1 KTN1 CL2 PAK1 RAB8A TCP11L1 HSPA13 PLA2G7 SEMA3A S100A9 PTK7 P2RY14 DMRTB1 FBXW11 DMD EXT2 METTL2A RBM19 RAG2 GALK2 HHATL C5H11ORF58 FGB PPP3CB BRAP NECAB3 JARID2 AGO4 PYY DNAJC6 MRPL38 ARFGAP2 MELK DDA1 XKR8 MINA PCNP TBX4 LOC100502566 USP6NL HOXB4 ETS1 BASP1 LOC427470 SDR42E2 DCAF7 C11H19ORF40 OTUD6B FZD4 LPGAT1 EXOC2 MESDC2 PKIA CAB39L TBC1D15 ZNF800 SEC22B FAM126A TNFRSF18 ATP6V0E1 PDGFRA ALAD GATSL2 AVD SNCB C12ORF57 9-Sep MYD88 RLIM MAPRE2 PPM1B ARID4A RCSD1 TSPAN15 ATM GNB1L FDFT1 ANP32B BUB1 BORCS7 LOC100858381 AP3M1 CACFD1 AXIN2 MYBPC3 MOV10 LFNG ZNF767 PEX5 VSX2 RREB1 CCK ROR1 TCF15 LSM8 MIF RFTN1 EMB SLC25A15 FKBP8 FGFR1OP2 LEF1 FXYD6 WNT2B TMEM70 GJC1 SLC35A3 SRSF3 SOCS3 TMEM164 TMEM229B ASNS NEMP2 LOC424740 TLDC1 MAP3K14 PTN SSB C28H19ORF10 TAGLN UBXN2B SUGP1 ELOVL5 MXD1 REL AMPD3 KCTD2 MRPS33 SSR1 ZFP64 FOXM1 RASSF5 KLHL24 ENTPD8 RRAGC SLC40A1 TMOD3 DPYSL4 RP11-196G11.1 XIAP SNX16 BRSK2 CSNK1D PAQR8 HDGFRP2 KLHL20 LHX1 CD247 FAT3 DAP TEC CCDC167 JMJD4 E2F5 TEX264 CCNE1 LGI2 LSG1 ZCCHC6 ANGPTL2 SLC26A5 RP4-613B23.5 FANCC MFAP1 RRAD NKAP ANGPT2 SS2 PDLIM5 CSNK2A2 SLC25A22 MGLL NR2C1 LOC100859148 C4BPA HAVCR1 ACBD6 STUB1 MTMR9 VPS45 FADS2 DCUN1D1 HNRPK TCP11L2 PRTG IL16 SLC51A TXN2 FAM49A FZD6 GOSR1 PRLR ASH2L LOC431499 WBSCR22 C3H6ORF154 PRRG1 DHCR7 NRG4 SMIM12 SERPINA4 TNNI2 ACAN RHOA GJA1 RGS17 ADCK3 LIMD2 RNF139 TMX4 XIRP1 DCLRE1B FAM114A1 RASD1 LOC426385 ITPR1 GMPR PDHX AvBD8 EEF1A2 EFHD1 PSEN1 C1H7ORF60 SEPT2L SPP2 SZRD1 ETFDH XPO1 CTDSPL2 ZFAND5 TNFAIP6 FBXW5 EYA4 MTMR8 MYL12A C8ORF22 FAM118B WDR91 BRE LBFABP PTPRO CKMT2 TMEM184C CNOT1 CHAC1 LOC422926 JUP WNT7A STAT5B FAM60A CLPX AC005943.2 ZNF330 ESR2 DRAXIN RAB35 ASIP TMEM138 CDPF1 SRSF2 UGT8 ABCF2 ATP6V0E2 DDT TSPAN3 PPP2R5C STIM1 POR EPB41 ANXA7 MYLK2 TMED8 CEBPG HESX1 CLEC3A VPS41 ANKRA2 ENTPD6 RALGAPB PPP2R4 MSANTD2 RNF4 IGHMBP2 HIPK3 SRC NT5C3A PIK3CD SLC16A1 SCD CSRP3 TRAF5 C1D SLC39A9 ECE1 MATR3 TFEB BEAN1 TBL1X UGP2 IMMP1L HAPLN1 SPG21 BET1L SLC6A9 EDC3 CST3 ARNTL2 SUB1 C26H6ORF89 RASSF3 GLT1D1 CDC42SE2 TMEM56 BKJ NDUFB2 DPH1 SENP8 SLC46A3 DCBLD2 LYPLA2 BTD BPGM RAB24 ABCA1 LUM SERPINI1 ENO1 ZNF384 GOLGA7 TNMD MYL9 PLEKHJ1 RAB9A SLC37A3 CLOCK COL8A1 ANKRD16 RNF141 CD200 GPR149 LOC100858447 DSTN FGFR1 CD3E EIF2AK2 PRKAA1 DERA LOC422249 LOC419851 LIFR SPINZ C1H2ORF49 LOC420419 KCNA4 DAPP1 GJC2 SLC19A1 E2F1 SMIM5 SPPL2B SLC8A3 CDC2L1 OPTN SRSF5A HOXB1 PRMT3 PNAT3 NHLH1 LOC416354 ZBTB17 CDH6 SLC35E3 UBL7 EDN2 AAMDC IRX4 RAB14 NEK6 CSRP2 MPP5 SLC9A3R1 HAS2 JAK1 PLEKHM1 BRINP1 CMC2 ARHGDIB CSDE1 DFNA5 CANT1 PCMTD1 GUCA2B NCLN GNB5 ANO5 ELF1 SLBP AKAP9 UGDH FZD1 NBL1 RAD21 RHOBTB1 WDR45B PLEKHB2 TARSL2 TNNT3 RRP12 RBBP7 INTS2 CLDND1 DEPDC6 CHRM3 KCTD9 IL6ST COPE PTPN1 SLC17A5 RAB5A STK17A IFITM5 DTWD2 TMEM173 ALDH1A1 ABHD13 GORASP1 PRMT7 CNTN2 CD5 TBX18 ATP5C1 PMS1 CDC42 RECQL KCNA2 RANGAP1 ZNF512B FBXW2 RASGRP3 PODXL BORCS5 FAM26E CLINT1 UBN2 SCNN1A SYNGR3 GDI2 CALM1 CNPPD1 FAM222B LPP ADH1C CX3CL1 PFKP TTL NUP93 UBE2R2 CLTC SPTSSB KLHL18 PIGA DNMT3B CDC27 SMARCB1 SEPSECS RRP1B LOC395991 BCL2 AKT1 ICMT POPDC2 TSKU PTP4A1 POC1B TSPAN13 VIT EGFL7 RFNG MTFR1 DYNLT3 MGAT4A C10H15ORF61 CREG1 GLP2R SELO NFKBIA NCALD FBXO34 NUDT19 SLC1A2 MAP1LC3B SYTL1 C3H6ORF120 LCP1 IRF2 ARHGAP19 DOLK HSBP1L1 CTSS GTDC1 RAB11A VDR APOD CHRDL1 FAM76A CLEC3B TRIP13 LOC426913 LHFPL5 STAU2 TMCO3 CLIP1 SHISA2 FBXL21 UBE3B AHCTF1 FMOD RECQL5 ALG12 FASN CELF2 WBP4 MAP2K5 TREM-B1 NFIL3 TMEM184B PRDX1 CD200R1L LOXL2 CAPN1 MAD2L1 CRTAP CACNA1B GLIPR1L HOXD8 RAD54L TNNT2 CNBP MAPK6 BLOC1S6 MTMR2 TAF11 RER1 NEK7 ZP2 FAM105A DEAF1 CDCA7L WASL AP4B1 SPPL2A SLIT1 VRK1 VAMP7 FOXO1 H1F0 ARHGAP15 NGF MGEA5 NCAN TASP1 TSSC4 PPHLN1 TNC CCR5 STK10 PDE6H RABGAP1L SLC41A2 MOB4 NCAPH SPG7 PABPC1 TMEM106B ACVR2A PSEN2 CD44 COQ5 SPINW DDB2 GPATCH2 MYH1A MGME1 PHF21A MGAT3 ENTPD2 SLCO4A1 TMEM170A CEP112 DLST GPR107 FZD3 FAM192A PSME3 BLOC1S5 HIC2 YRK FAM177A1 FLOT2 KCNN2 SLC39A13 THYN1 INSIG1 MRRF SRPR UQCRH DNTT STK11IP YBX1 YWHAB SMAP2 RNF185 UBE2V2 GJA5 ULK3 EIF2S1 USP45 RGS6 ART1 DMRT1 CD8B VPS53 PLN MCL1 FSTL1 RBMS1 SGMS2 LOC396479 CAT EI24 PCSK7 FGFBP2 RP11-20I23.1 SUPT3H ENTPD1 PHLDA2 TSPAN1 CNOT2 MPST NCOA1 ATF4 TPH1 CCDC127 TAF7 RP11-49K24.9 STOML1 RHOC FABP6 HMGA1 CCNA2 MRPS6 SRGAP1 BRMS1L ZFAND6 RAC2 INO80 CDCA4 ODF2 BBS4 ARIH2 TCEB1 PRIMPOL IRF4 SF3A1 E2F4 CYTH1 CRCP WDR1 HSD11B1L WWP1 LGALSL PRKRIP1 AGPAT9 FAM20B SRF MAPKAP1 CANX YY1 CMSS1 UFSP2 TANK ATG9A NT5C3B PRPF3 ZDHHC8 TMEM180 CTD-2410N18.5 SCOC PAX9 CUL2 UBL3 PAFAH1B1 TIMM8A ATP1B4 B3GAT1 MYH11 SPCS1 SATB1 TEF FAM213A C20H20ORF24 PACSIN2 SCYL3 LDB2 CNOT7 STMN1 ITGB2 NEDD1 ACLY RUVBL1 BZFP1 ORC5 FGFR2 RABL3 VPS18 NGEF MRPS26 PLAG1 ELOVL1 TRPC4AP BTC CCDC6 CPSF2 HHEX ST3GAL5 BID FZD5 KHDRBS1 STAT1 ST6GALNAC1 ESRP2 RP11-145E5.5 SH3BP2 DAZAP1 FAR1 BRCA1 TYRO3 SNX10 LYN ELAVL1 MRPL45 EIF2B2 RMND5A MEAF6 ADAM9 MVB12B CDH5 PLEKHO1 SERPINE2 |
| gga-miR-202-5p | STAU2 OPN1SW PACSIN2 ABCA1 AC113404.1 ALDOC ZFHX3 OPN5L1 KCNN2 MGME1 FLNB NR1D2 CTNNA2 PLN ZNF302 UBE2G2 TMEM123 KLHL20 CLTB PTDSS1 NGEF BSG PRLR OSTF1 LOC418667 GLI2 RHOA MRPS7 CTNNA3 NUDC ACOT9 POPDC2 SELO EXOC6 DRG2 SETD3 HIC2 ARFIP1 RREB1 PAX6 SRPR WASL PLA2G7 FUBP1 GLP2R SYNGR3 SLC34A2 GCLM SDR16C5 PCGF2 CRIPT OSTM1 BAZ2B 8-Mar TMEM11 ST3GAL3 FUT9 ALG12 OTUD6B RNF14 CCDC61 VCAN LSG1 CDH5 RBM12 ARIH1 BRAP NUP93 WIPF1 PTBP1 ZW10 CSPG5 MAPRE1 PLIN2 RORB CREB1 MYL9 LOC416354 SLC26A5 GATA4 AHR SLC17A9 CMKLR1 TMEM138 E2F1 HAPLN1 CPSF6 LYSMD3 FEM1B RUNDC3B GLRX5 SCP2 RLIM NAV3 NFIX PSEN2 PDLIM5 TRPV4 LPAR4 CD36 CD200 MATR3 GHRH SLC16A1 BBS4 OAZ2 PTP4A1 MCL1 RECQL PLS3 RBM12B HYAL6 MYO1G CNOT1 RP11-77K12.7 RCAN3 DHRS11 WDR45B API5 CHAT2 PLAU PDP1 THY1 HTR1A G3BP1 HESX1 FAM76A YRK TMX4 DCAF7 TDRD7 CCNE1 SLCO4A1 NR2C1 SOCS5 ZNF512B SEC16B TMEM175 STAT4 IRF5 AARS2 TRAPPC3 FEN1 HCLS1 PSMD14 XIRP1 NOTCH1 WDFY2 LGI2 XIAP MORN4 CHD2 ANXA7 SLC6A9 PMAIP1 C1H12ORF23 G0S2 CECR1 ST3GAL2 SLC46A3 PARK7 C1GALT1 MRRF GREM1 IRX4 OCLN KIF2A MYH10 TLR1A PRRG1 PRC1 NCL CDKN1A NFIL3 LOC419112 NOV MESDC2 5-Sep LOC419429 GART NOTCH2 CD44 CHST10 SLC25A15 EPN2 PKM PFKP ATP1B3 C1H7ORF60 TPH1 ATF4 VPS53 ESRP2 ST3GAL6 USP45 BZFP1 PEX2 SLC16A8 ACTG1 LOC396479 TYRO3 PTTG1 ATP2A3 P2RY14 UGT8 ATF7IP GJA1 NBL1 APBB1IP S100A10 PNPLA6 TRMU CENPO SNRNP200 CALM1 HNF4beta RAD52 INSIG2 EPGN TOB2 ZFAND6 SEPT2L RELL1 TNC XPO4 SERPINA1 H1F0 RFTN1 B3GAT1 LIFR PKIG GET4 BEAN1 IL8L1 LOC769139 QRICH1 ELAVL1 NGF KIF5C FAHD1 ENOX2 KIAA1467 EIF5B RNASEL RGS17 MYD88 UTS2B PIK3CD NUMB CTD-2410N18.5 SMIM4 VDR STRBP CASP2 CAPN1 FAM49A HSPA9 HHATL PDE6H FAM65B DMTF1 TIMM8A EARS2 ABCC1 TMEM5 SPP2 FXYD6 PIAS2 SRSF1 PPP4R2 ATP6V0A4 PHTF2 ACACA CRTAP ANGPTL2 SLC25A22 PHLDA2 MRPL28 NT5C2 MEAF6 BCL2L1 CANT1 RAB14 GPR107 C1H11ORF54 ALDH1A3 TEAD1 ACSBG2 PIK3R5 C26H6ORF106 CITED4 ANXA6 ST6GALNAC1 RPL9 LPP SERPINE2 MYL12A KIF3B EYA2 CNR1 PIK3CB PTK7 UBE2G1 SERPINI1 TMEM178B CPA6 NFRKB PSME3 C3H6ORF203 STK11 PTN JMJD6 CDC42SE2 SERTAD2 K123 UBXN2A NAA50 SLC8A3 STAT5B JMJD4 POLE3 YWHAG RRP12 LOC420419 CHIC1 CAB39L MAP3K14 LRIG3 PHOSPHO1 LYRM9 PLAG1 CSRP3 AZIN1 FANCC DCN EMP1 SCN9A TMEM106B ADD1 GCC1 SRP68 RGS6 NCAN SMIM18 ASIP SUGP1 OCM FAM18B1 CDH17 RP11-20I23.1 PRMT3 RBM5 TRIB2 GBX2 CBFB NARS GAD1 CKMT2 PDS5B ANXA11 ACAN EDN2 MCM3 FGF1 TNNI2 BMP10 SNAP91 DNAJB12 EXOC2 SCIN ERGIC2 RAB3IL1 9-Sep ACSL1 TRPC4AP CLDND1 ST3GAL5 ALKBH3 LOC396009 TRABD SYT1 CSNK1D COL22A1 DCTN1 LOC426385 PIP4K2A CDH8 RAG2 RBP TNFRSF18 GJA5 CLIP1 SNN FBXL16 YPEL5 GDPD5 TFEB NCALD S100B SPPL2B CNTN1 TGFBR3 NFAT5 EEF1A2 TRIM55 RASGEF1C CD24 MSRB3 TTL VTN CUL2 CDV3 PLEKHB2 ETV6 SPTSSB RARRES2 LEF1 CLDN3 CLOCK MASP1 SSTR1 DRAXIN TBX5 RNF152 AATF A4GALT SEC22B MYBPC3 VIT LUZP2 NEMP2 ETFDH ARL6 SMIM12 PPP1R16B NR1H3 DPP4 PDPK1 ACVR1 CDH6 CDCA4 TMEM180 OVALX MBLAC2 TMEM170A POLDIP3 PTGS2 ACE WBP2 FAM3C MFAP3 MYH1C C7H2ORF76 FAT3 PRKCD HERC3 BPGM CXCR1 TMEM120B CNP UBAP1L TBL1X FAM126A LCLAT1 NREP RAB3GAP2 RNH1 RBM19 GTF2H4 TNS1 LGALS3 CDPF1 ARL6IP5 DEK ELMO1 WDR1 GFPT1 FOXA2 ALCAM HDAC4 RAB11A HNRNPDL CSNK1E SPPL2A CMTM7 POLR3H ZYX LFNG CHRM3 UGDH TSC22D3 PDGFD SHOC2 JUP PDGFRA PPFIBP1 C7H2ORF69 RAB9A SRGAP1 TSEN2 RP11-49K24.9 PECR SET CDCA7L TBL3 SNCA EXD2 HPGDS FGFRL1 USP1 XPO1 THOC3 BID NCOA1 CD276 KATNBL1 TMEM104 PMS1 ZBTB17 RHOB KLHDC2 PCDHGC3 COL8A1 CMPK1 HRAS FSTL1 AGO4 WDR91 MTMR8 ZNF767 TRIAP1 RALBP1 LEPR GNB1L ICMT NCOA4 YPEL2 KCNH6 FOXD2 HAS2 SLC16A7 SLC38A4 SMAP2 PODXL KDSR CD93 RAD21 NAP1L4 FZR1 ARF1 MGLL FAM175B EHF RP11-552F3.12 MBP SDK1 ELOVL6 KCTD9 ITGB2 HIPK3 PAQR8 MFGE8 MEF2BNB WDR44 NKX2-6 TPRA1 KLHL18 CTD-2116N17.1 TCP11L2 YWHAB ACADSB ENAH CDC42BPA CD247 F13A1 TMLHE MIB2 HMGA1 USP6NL TP53I11 STAU1 SLC9A2 RALGPS2 NECAB3 AKT1 MBD3 RAB33B CTNNBIP1 CTDSPL2 SRF SZRD1 APCDD1 ENTPD8 PLEKHB1 CTCF LIN52 JAK1 LOC428335 SATB1 IL6RA ADIPOR2 LHFPL5 FBXO34 CASQ2 UBN1 NRXN3 TNS3 SYCE3 ARFGAP1 HOXB4 AMIGO2 ARHGAP26 RP5-1021I20.4 TWIST2 BRINP1 FAM20B FZD5 ADA CACNA1D TMEM68 TPST2 SPATS2L MAPT PDHX KLHL24 VPS41 CDKN1B MSX1 KPNA1 RIC8A GCHFR TOP1 CNGA3 OC3 UBE2V2 NRTN PCMTD1 ELF1 ERLIN1 NME3 SH3BP2 FYB MAOA PPP2R4 NEUROG2 SMPX STARD4 SLC19A1 RTN4 LCP1 SLC1A2 CLN8 PELI1 AIDA FZD4 RIPK1 MGEA5 ITGB1BP3 RP11-834C11.12 ZDHHC17 OSTN ZDHHC21 CNTN5 FAM45A TBK1 ALDH1A1 FHIT TGFBR2 XPO5 RNF185 NRXN1 SULT1B GUCA2B GLUL WDR24 CCDC28B TAL1 RABGAP1L FHOD1 VPS4B RASGRP3 FBXO8 INSIG1 GAPVD1 STK11IP PEX13 TRIM8 CHP1 NME5 RP11-514O12.4 P4HA1 POR C14H17ORF103 BIRC2 DOLPP1 PAFAH1B1 ARHGAP19 TMEM41B ACOX1 PSME4 MPP5 6-Sep VNN1 PTPN6 COL18A1 PUF60 YBX3 HMGB1 MGAT4A MYBPH MTMR3 FBXL21 NRG4 CD80 RNF25 STUB1 ACAD8 CD72 NHLRC2 FGFR2 GINS1 KLHL13 GALNT1 LHX9 GOSR1 AGMAT PRKRIP1 BCL6 SGMS2 ELL KTN1 TEAD4 SNAP23 GNG13 PDCD6IP CA2 UBE2H GNB5 LDLRAD4 TMEM184B FAM105A DYNC1LI2 C1H21ORF33 DHRS3 CELF1 GTDC1 PSMD3 C26H6ORF89 ZIC1 BORA PGR MYO1C CRYAB LOC396531 COL6A3 SYT12 WBP4 RFFL SUPT3H PCMT1 PNISR PRKAR1A MIER1 CUEDC2 CDH20 CRYBB1 COPB1 CAT MET PLEKHM1 RCHY1 GSTT1 BASP1 CTSB DNAJC5 ULK3 SLC16A3 PROKR2 RORA RAP1GAP2 CDC42 CNOT7 EYA4 SGCB TNFAIP8L1 MPPED2 DNAL4 RGS4 BRCA1 HMG20A GPX7 CD151 RAB10 CHST3 C1H2ORF49 CLDN1 FBXW11 PAPD7 SYTL1 TMEM230 TCEB1 METAP1 TERF1 PTRF FHL5 ADCK3 VAMP7 BTG4 PIGR PRNP ELOVL1 PANK4 ZP2 MAP6 EPHX2 LOC776816 ORC3 FABP6 ARHGDIA KCND2 CDH7 ARRDC1 ORMDL2 TAPBPL DEXI CD200R1L RGS19 TSKU PPME1 GJB1 CDC25A IKZF5 FAR1 RAF1 RALGAPB PAIP2 SLIT1 HERC2 MGAT3 RBM48 NFASC TNFAIP1 ID3 PNAT3 C28H19ORF10 MAPK6 CBX1 H2AFY ATP6V0E1 FLT4 SLC20A2 DAAM1 LOC100502566 SDR42E2 BRSK2 COL9A2 ANP32B TBX22 GNAT2 NADK SALL4 MYH11 AHCYL1 SMIM5 CST7 RNF34 MYH9 PAAF1 FLOT2 TBC1D23 MAT1A EGFL7 SOUL TIPIN STIM1 PTPN11 LOC100859039 ABHD17B EGR1 FAM222B ZNF706 ASNS UBAC1 FGFR1OP2 ABCC4 PLAC8L1 TNFRSF19 |
| gga-miR-218-3p | RRP1B LPP C1D SLC22A7 HMGA1 COPB1 TBX6 PKM POLR3H NFIL3 SLC40A1 CNIH1 CUEDC2 SLC35A3 ARMC1 CHADL SGMS2 BPGM ST13 HDAC2 HAUS6 ZYX LOC415324 LOXL2 KDM5B GJC2 NFIA TMCO3 E2F4 AC113404.1 RASA2 IL2RA PUF60 UBE2F SLC26A6 PLEKHM1 TRPV4 LXN FGB SERPINA4 TCF3 NEURL1 OPN5L1 SUCLG1 CMPK1 KCNA4 KLHL18 CACFD1 C11H16ORF70 P2RX4 TDP2 FAM60A ABHD13 UBL7 LOC415664 B4GALT6 GJA5 ADH6 ETV1 USP12-like IL18R1 RBMS1 KDELR3 UVRAG ZNF800 LSM3 TARDBP BRE ATP6V0B ENO2 LOC422926 K123 NFASC CDCA7L TP53INP1 FBXO8 AKT1 NEMP2 RHOB ANK3 FK21 TBX22 STRBP RPS14 WWP2 IL10RA DHRS11 SLC2A2 AR KIF5C PAFAH1B1 B4GALT2 SYT13 CCL20 ALG12 INSIG2 BET1L URAH RAD51D CDPF1 WDR44 WDR24 PDGFB ASNSD1 MGST3 ARHGAP15 KDELR2 DUPD1 EIF6 RCHY1 VPS41 LOC396380 FAR1 MRPS16 JMJD6 VTN NRTN SASS6 TADA2A OLA1 PSMC3 CST7 LFNG ZFYVE1 SYT12 NME3 APCDD1 CAB39L CCNE2 CLOCK ATP6V1D MRPL38 PCDH10 POR SSB PFKM EFHD1 ALDOC CDH6 BMP5 RNASEL PIK3CB CDH11 DPH1 RECQL5 RASGEF1C ACBD3 C26H6ORF106 SETD4 ANKRD10 MRPL20 MAP2K5 RBM48 CCNL2 PTP4A1 NCALD PTPN9 TAGLN SLC37A3 TNFSF10 PLA2G7 MRPS6 ATOX1 CD80 KLHL24 TNFAIP6 SDR42E2 VDAC1 SPPL2B ACACA MAPKAP1 ELMO3 C10H15ORF59 BRSK2 CTDSPL2 YWHAZ YWHAB TPM2 ZDHHC21 NUP93 FUNDC1 CETP AVEN CCNK AAMDC NDUFB2 CASP14 HDAC11 SUB1 C15H12ORF49 RFNG SLC34A2 RALBP1 POU1F1 PDLIM5 RECQL PAN2 TSPAN12 GCM1 STX2 GABRG2 ATP6V0E1 FZD4 COX16 LOC422090 N4BP2L2 ARHGDIA CD200 TIMM9 STRA6 UBE2V2 ACVR1 RPRD1A RIPK1 IRAK4 HABP4 SH3BP2 SPP2 GID8 IGF2 MOB4 TTLL5 PTGS2 GTDC1 LMX1A NR5A1 IFITM5 C18ORF42 CNP TMEM120B SLC8A3 VDR DEXI MPPED2 CBL YWHAG CSTF3 PRC1 PAPD7 SOUL AANAT IRAK2 IGFBP1 TMOD3 EIF1 EGR1 PMS1 SERPINH1 FADS2 TM2D3 FLOT2 PELI1 FGF1 NCLN TSKU CDC27 RAP1GAP2 PFN2 SLC46A3 LOC100859039 CD151 PLAU C14H17ORF103 PTN CSF3R MYLK2 SZT2 WASH1 PARN SETD6 RSL24D1 CLN5 GFRA2 GPATCH2 CA2 SNRNP200 IMPG1 AHR CFAP36 CPNE1 AQP9 OVAL ST3GAL3 ATP6V0E2 CALM1 GHSR ZCRB1 TBL1XR1 AP2M1 TNNT2 CYB5A RAF1 EIF4A2 G3BP1 PLK1 SNN KIF3B EHMT1 HCLS1 PPAT PDE3B SERPINB10 CEBPB LYRM9 ITPR1 ETV6 STK32A PPP2R5C 15-Sep AHSA2 IGFBP4 PNRC1 HIC1 SMARCE1 FKBP8 DKC1 NRN1 NELFA POLK ESRP2 NOP56 PSMD3 PIP5K1A VPS37C MYH1A CNOT8 TMEM170A BASP1 ABCA1 AAMP RPS2 TRABD RNF185 SATB1 LOC420849 AREG PEX13 MRRF SYNGR1 CHD2 HIAT1 TANK TSPAN6 TMEM30A CSNK1E IAH1 ABI1 HDAC4 ARRDC1 AP4B1 SSTR2 ODF2 PUM1 ULK3 CDK2AP1 ANP32E IER3IP1 MORN4 TBC1D1 PEX2 PPP2R2D ELOVL6 TNS3 AZIN1 YPEL2 SLC25A26 CTBP1 OTUD6B POT1 SSU72 DCAF7 DUT CDC42SE2 PNAT3 SELO NKX3-2 GLRX GDPD5 TNFRSF1A RBM25 HERC2 CRYAB AGPAT9 USP45 ELF1 YBX1 RAB11A ATG7 CRYAA TMEM104 ENTPD1 SLC16A3 CTSB TEAD1 TPST2 LSG1 TCEANC2 SERPINI1 RPS6KB1 BRAP TFEB FAM172A CNR1 A4GALT ID1 POP4 MYOM1 PMP22 TAF8 MGAT3 RDH10 BRPF1 BORCS7 CCDC6 ATG9A LOC420419 CACNA1B FZD5 ABHD17C EXOC3 ATP6V1G1 NOTCH1 ENTPD8 LOC418667 SAMM50 TOP1 BID GHRH PIT54 KCNH6 LOC421975 PHKB CDV3 CHM ASB9 MXI1 SLC25A22 CRADD NKIRAS2 YTHDC1 NDC80 ALDH1A2 SSR1 MSRB3 PARP1 FURIN CANT1 GALNT18 GJB1 CLTB SEPSECS KDR TF FAM222B DEK APBB1IP HMGCR PTDSS1 XIRP1 S100A1 NMU CUL2 MET TMEM121 REL CD3E LIMK1 TDRD3 FUT9 MTIF2 ELAVL1 MAFB PLS3 LOC421740 RP11-20I23.1 RAD21 MMP9 GALNT6 FAM45A RAB10 FAM213A CYP2D6 FZR1 TCF15 GNB5 STK11IP LYN SERPINA1 ALDH3A2 WSB1 GNB1 B3GNT2 EEF2 PSPH COG4 ANAPC10 DDB2 CD72 FAM114A1 SZRD1 GSTO1 SMIM5 FBXO34 FAM175B MTMR8 NECAB3 ACP2 TTL PSMD14 GCLM TOB2 GARNL3 NT5C2 FAM105A PDGFRA GPR39 DCAF13 MYOM2 ABLIM1 LOC396531 T ITGB2 MCCC1 DOLPP1 ARFGAP2 CAT SUGP1 ATM CNDP2 GPR89B ZNF706 GATAD2A MXD4 GABPA PLEKHB2 EXOC5 EIF2S1 ADD1 SMPDL3B RLIM SLX4IP S100Z RGS6 UGGT2 FAM20B CRH SRSF1 TYRO3 ACSBG2 GREM1 RIPK2 JUP CXCR7 NAPB TRPC3 PHLDA2 PPHLN1 PLCZ1 AARS2 SLC16A1 5-Mar ST8SIA1 CCK PIP4K2A TBC1D15 ARNTL PHTF2 LOC420860 OC3 GOLGA7 KLHL20 GSKIP MRPL48 FBXL16 NEDD1 STK11 RUNDC3B MITF SFTPA1 STAU2 TBC1D22A GJA4 TIPIN SYNCRIP RFC1 MUM1 RNF14 GALNT1 LGALS3 KRIT1 IFNGR1 FK27 LCLAT1 DOLK CCR5 IMMP1L CRYL1 MICAL1 COX7A2 CNGA3 LIMD2 DRAXIN SLC39A13 ALCAM SPG7 ETS2 SNX12 OSTM1 GCC1 TNFRSF18 MXD1 DDA1 CKB MAP6 NRXN1 SMIM19 XPO5 RAG2 UBE2I CIR1 TLDC1 CNOT1 CDH7 TMEM68 TLR21 TMEM11 PAQR8 FYB TNNI3K UBE2H GNB1L SLC1A2 ADA FHL5 BHLHE22 PRDX1 AIDA CAPN3 RNF111 GET4 RP11-403P17.5 MPP5 TLR2A MGME1 CLDN2 DCN MEF2A INSIG1 NCAPD2 NAE1 GIGYF2 PRKAR1A FASN MFAP3 SGK196 FMOD DCTN2 PRIMPOL RP11-290H9.2 DYNLT3 KATNBL1 ST6GALNAC1 SNAP91 GRIA2 MTHFS MLX CATH1 ZP3 LUM OSTN RAB9A ARFIP1 HNRNPDL CDH5 LOC770684 TAPT1 ACOT9 UBE2G1 ALKBH3 DAAM1 IRF8 PRLR FANCC SF3A1 ZBED4 TNFAIP1 NCAN MATR3 BAZ2B BRD2 TSPO TMEM189 MAP1LC3B AMPD3 LIN52 OAZ2 PAAF1 EDA2R SLIT1 MCEE C1H12ORF73 RAD18 TNS1 OCLN SRPR LYRM1 ANKRD27 GART SDK1 PIK3R5 BSG WDR70 FLNB ORAI2 ADORA2B HABP2 STAT4 ACTN1 MTMR9 TRAF5 PFKP NHLH1 RAB14 NEK7 LOC416354 P4HA1 CDCA4 SEPT2L SERTAD2 ADAM9 SRP68 TAF1B MYH11 ZP2 USP6NL STX7 SEMA7A RER1 GABRB3 SLC47A1 RORA BORA GFOD2 SSR3 RBP5 FLT4 CDC42 PALM DNTT METAP1 SHOC2 RGS17 FOXO1 SEC22B SCIN FSTL1 RHOC FECH RTN4 CTCF DCBLD2 H3F3B EXOSC2 SOCS3 STAT1 KCTD7 TNNI2 COL14A1 CD86 VGLL2 RB1 UGT8 EIF5A2 PRMT3 FAM46A IKZF5 FAM46C TERF1 TAPBPL TXNRD1 OVALY MRPL50 AMIGO2 ACE HNRPK BMPR1B RREB1 DDX55 HPGDS MAP3K14 RASSF5 CREB1 CHRDL1 SOCS5 SLC17A5 DNMT3B C1H12ORF23 SPINZ PDPK1 KTN1 EEF1A1 DUSP10 SMIM15 CBLN4 LMBRD2 NUMB ADCK3 LOC427470 KIF2A SGTA SRF MIB2 COL17A1 NME5 ANKRD26 TSPAN1 CPSF6 AXIN1 DMTF1 SSTR1 CNTN1 RBBP7 CDK10 TRAFD1 SPG21 PPP1R16B CD99 UQCC XIAP GLI2 SYTL1 CSRP3 ORC3 AC025048.1 RABGAP1L TEC SDR16C5 EI24 SYNM RHOBTB1 PTBP1 SRSF2 NECAB1 GATA2 CD200R1L PPP1R8 FZD3 CLIP1 FGA GAS2L3 MCL1 STK40 DHRS3 LCMT1 MCFD2 C1H21ORF91 MGEA5 HOXB4 RBM22 EGFR STARD4 RFTN1 CYP24A1 ANXA7 IL8L1 TCEB1 KLHDC2 TMEM5 5-Sep WAPAL ZBTB2 PHF5A SGK3 CCPG1 STIM1 UBXN2B DHCR7 LOC396009 SOX11 LRRC59 CD82 FMO3 ORC5 ACLY SLC19A1 LOC100502566 BCS1L PKIG ELP6 CITED4 MAGI3 MBLAC2 OSTF1 RHOA ZFHX3 ALAD MVB12B TXLNB PLCXD1 CHN1 IL13RA2 TOX3 TRAPPC3 ADORA3 ACAD8 ANO5 RP11-49K24.9 CHST3 TFEC TBX5 ANXA11 SCNN1A ECI1 LSM5 DNAJC5 RNF103 ASS1 ZSWIM7 MSN HESX1 CTNNA2 COL6A2 ARFGAP3 RBM19 UPRT DNAJC12 ELK3 CEBPG ANKRD40 MPC1 CANX NAV3 P2RX1 WDR18 MBD3 TRIM59 SH3BP5 CECR1 RAC2 PAX6 ELL RAB24 UBN1 S100A10 ARL6IP5 MAFA EDEM1 AHCYL1 CRCP E2F1 C8H1ORF27 NBN FBXW11 CPT1A AAR2 SLC35E3 COL8A1 RABEPK VPS18 LGALSL SSX2IP DAD1 CISD1 RGS16 CD276 RHOG VPS45 TMX4 RAX2 DNAJB12 STX17 USP37 KITLG TMED5 PDHX VSX2 UBE2G2 CSNK1D AZIN2 TEX264 ETFDH TOMM6 MAFF CDKN1B RALGAPB WBP4 FAM118B HMGCS1 STX6 NUDT5 RAB33B EIF2B2 RGS9BP CD5 C1H21ORF33 AMY1A CTD-2116N17.1 YPEL5 TSC22D3 HPS1 GPR174 TCP11L1 NINJ1 BRINP1 RALGPS2 ABHD17B ACVR2A IL5RA B3GAT1 YWHAQ UBE2D3 RAB19 OVALX ATF4 FAM49A CDH17 TARSL2 PCMT1 CCSER2 WIPF1 MSX1 MSANTD2 COL9A3 PLEKHF2 SCFD1 CYP4V2 GAS8 MAP2K1 EYA2 EYA4 NUDT19 RP5-1021I20.4 WLS LYPLA2 PDP1 LOC425362 FGFR2 CAMK2D MYST2 EPN2 ARFGAP1 MLNR MASP1 HSPA9 LIFR CCDC101 DHX15 COL22A1 BHLHE40 NDUFC2 PIGY SERPINB6 8-Mar WDR92 C2ORF88 LZIC RP11-552F3.12 NAP1L4 TM4SF1 CAPN1 ENO1 PDCD6IP GPRIN2 WDR83OS HES5 ASIP BBS4 RGS19 ARIH2 SLCO4A1 CSPG5 RRAD ACBD6 ESR2 FAM76A TNNT3 BCL2L1 PLEKHB1 FAT3 PIGA GMFB PIGR MTMR2 WDR91 WBP2 GEM PHACTR1 TPH1 SYNJ2BP TAX1BP1 FAM53A MID1 PTRH2 ORC1 AGTPBP1 SOX10 ZC3H6 RAB3IL1 LYRM4 GAD1 YRK VAMP7 MEAF6 SETD3 ECE1 MAL2 TMEM184B MESDC2 ORMDL2 NTMT1 ARIH1 RBM24 KIAA0586 RAB35 GABRA1 GBGT1 CRTAP KCTD9 LUZP2 GNGT2 ODC1 TP53I11 FDFT1 FAM177A1 TPRA1 SYNPR HSPD1 NR2C1 ZNF384 FAM18B1 ENOX2 GTF2H4 DCUN1D1 CHMP4C RNF220 ZCCHC6 LOC431325 DIO1 SNX10 NR1H3 HMG20A CD247 HES4 TMED8 TECTA LECT1 ENSA VDAC2 UTS2B CACNA1D TMC2 TMEM141 BRCA1 ATP2A3 TMEM230 BIN2 PAFAH1B2 WFDC1 ELOVL5 SCYL3 PRRX2 CELF1 MYH10 MEF2D LEPROT KCNJ8 EEF1A2 APOV1 RASSF3 CCT6A SLC9A3R1 ATP2B1 EXOC6 RBM12 LOC426385 IRX4 PITX1 SLU7 PIAS2 PRTG SLC6A9 ARL6 CLDN1 RPA1 BAK1 PNPLA6 RPL31 NDEL1 CTGF SNCB PARK7 MAPT PDE6H DNAJB6 PANK4 IRF2 SMIM12 FABP6 INCENP VPS50 VCAN EGFL7 NAGA ARF1 RELL1 NFYA NCOA4 THADA VIT PPFIBP1 CHST10 SEC11A CRMP1 HIPK3 PLEKHJ1 RMND5A CSNK2A2 C1GALT1 IRF4 SMAD1 C28H19ORF10 NUP50 RAB8A ERICH1 LCORL BEAN1 QARS SLC25A15 DHX38 CHP1 PHC1 FBXO9 PIK3CA CMKLR1 STK10 RAD52 UBIAD1 LOC431499 ERI1 CCKAR PSEN2 SMIM18 SMIM3 GBX2 LOC396224 SLC17A9 ICMT ART4 SBNO1 CD93 CKMT2 SNAP23 CHRM3 KIAA1191 EDNRA EXOC2 CYTH1 TRAF7 SMPX RBM5 RS1 IL6ST SET PDGFD HYAL6 ELOVL1 HAS2 RGL1 STAT5B CDC25A NT5C3A C7H2ORF69 MRPS33 BTBD9 CNTN5 COA7 NFIX MAPRE1 RHNO1 CSRP2 TRIAP1 RGS4 NUDC ERGIC2 DAPP1 CHMP2A CSDE1 ADARB1 UBLCP1 GLUL TBL1X MGAT4A THYN1 EMB LEF1 PRRG4 C1H2ORF49 NR5A2 IPPK ST3GAL5 ZNF512B DCT ALG6 INO80 NREP AGO4 INTS2 NPC2 STOML1 CNTN2 KPNA1 TAF11 PPARD DRG2 BTBD10 IRF1 ALDH1A1 NGF NCOA1 TSEN2 SRSF11 SYK SUPT3H KCTD2 DTWD2 TIMM8A C5H15ORF41 TNC TEAD4 JAK1 LRP5 RABL3 HAVCR1 PUS7 SLC9A2 PLA2R1 PCGF2 PRKCI MAOA DYM RNF13 SPAST MYL9 MYD88 SOX3 PPP2R4 MRPL45 GCHFR PSEN1 PLIN2 SEC13 EPHA1 COL5A1 PAIP2 PPME1 NUB1 CCDC127 RNF4 RASGRP3 FXYD6 LOC424740 FHIT PIP5K1B LAMP2 TMLHE FBXL21 CTNNBIP1 MTMR3 PACSIN2 DFNA5 DYNLL2 BTC ZFAND5 MEF2BNB OAT AvBD10 RP11-145E5.5 ANP32B UFSP2 BCL11A SYNGR3 CSRP1 TMEM180 EDN2 CLSPN RPL9 RNF123 PPP1R2 NGEF HDX TNFRSF19 KIAA1467 GFI1B SERINC2 TNFAIP8L1 SPTSSB PBRM1 PLN LOC395926 SNX24 KLHL13 PTPRO GPR83 GATA4 PNO1 PLEKHO1 BTK TMEM207 IL16 COL18A1 ASH2L CD36 CDH20 SCOC NEK6 SERPINE2 MYH9 ACTR10 PDS5B DPM2 IMPDH2 PCMTD1 RRAGC TSSC1 UGDH LCP1 BLOC1S6 KCND2 TACC3 RBX1 NUDT21 STAR C26H6ORF89 ZFAND6 ARHGAP19 NOTCH2 6-Sep C10H15ORF61 TMOD4 TBX4 GLP2R C20H20ORF24 MYO1G ERBB3 FOS GLT1D1 VMO1 KDSR GUCA2B SLC38A4 DDX6 DNASE1 PLIN1 MAPK6 SLC26A5 MMD CD74 MRPL37 NTS ISL1 TRPC4AP SFR1 FAM126A STK24 PSME3 FAM65B SEPP1 ANGPTL2 FGF14 CPA6 TBL3 ACAN PLAG1 HNMT ADAL PRKRIP1 PRKAA2 CMTM7 PIK3CD TRMU SALL4 CREM KIAA0020 GTF3C5 RBM12B AATF BTF3L4 PCM1 MYO1C PEX11G DDX47 CLK2 TWIST2 GAPVD1 NBL1 GMPR CXCL14 S100B SCAF4 BORCS5 ZNF767 SPATA2 H1F0 PRRG1 NR2E3 AvBD4 CSF3 HIBCH DAGLB NOS2 GFPT1 SPATS2L SH3KBP1 ZFP92 PPP1R3E GNG2 JAM2 RP11-5A19.5 PEX5 GANC TCP11L2 VPS53 MGAT4C LGI2 FAM122A |
| gga-miR-223 | UBIAD1 PHTF2 CDC42SE2 PMP22 RREB1 CTDSPL2 NT5C2 UBE2V2 OTOR CNTN1 ACBD6 MSMO1 RAP1GAP2 RNF141 GEMIN2 ERGIC2 CLDN2 CHUK CAB39L PRKCI CHADL DYDC1 SERPINA1 BAZ2B MAVS CDH7 UGT8 CNOT1 ALDH1A3 AHR CSNK1D CREM ATP12A ETV6 PEX5 CHRM3 YPEL2 RAB8A SIGIRR FYB CPT1A BID FAM222B NRN1 RNF111 ANKRA2 ZFAND6 FGFBP2 NELFA PAFAH1B1 STARD4 DFNA5 OPNP MAP2K1 CDPF1 BTF3L4 ORAI2 C8ORF22 RFTN1 ZFAND5 CRCP FBXW2 GSTO1 RRAGC PTP4A1 CA2 BTBD9 SNX12 POPDC2 CREB1 TSPAN6 KLHL24 TP53INP1 CKMT2 PYY ALDH3A2 AvBD5 SYNM MGAT4A LGI2 LOC776816 MTMR8 C26H6ORF89 ACAN CCL17 KRT5 ALAD SSRP1 PRTG NEIL1 RCHY1 FBXO34 GATAD2A SMIM19 DENR NCOA1 SLC2A2 MXI1 CXCL14 MGP LEF1 GJC1 SERPINE2 TNNT2 MRPL45 PFN2 TCF15 SPP2 LRRC28 AMIGO2 HAS2 SERPINA4 BET1L P4HA1 NRTN ORC3 LOC416354 TMEM230 MTMR2 FBXL21 PDGFRA MRPS33 MAFB AR RGL1 ID2 SPPL2B NPAS2 RAG2 SYNPR GDPD5 PDGFB SPTSSA HIPK3 SH3BP2 AXIN1 ERICH1 UBXN2A RAF1 ACSBG2 ELOVL5 LRRK2 ST3GAL5 CRTAP UBE3C AP1G1 MAPKAP1 AARS CYP1A4 MIB2 ATG9A FGFR1OP2 GBX2 EXOC3 INCENP SYNCRIP RRP7A RBM5 A4GALT PPP2R4 TDRD7 MBD3 GATSL2 GCC1 IL5RA ZBTB2 RALGPS2 ADD1 WIPI2 CNTN5 CDCA7L TBX6 CSNK2A2 WDR18 STK40 BRINP1 TEAD1 WRN NMU CDH6 SLC15A1 ACVR1 FAM126A KIAA1671 ZFP64 TOB2 DRG2 RHOT1 AKT1 GPR174 TMEM104 GNGT2 PAPOLA DAZAP1 SGTA DCAF7 MSRB3 MRPL28 MRPL53 FAM49A HIC1 NAA50 SLC17A5 ACBD3 CD82 IFRD1 BORCS5 EPHA3 PIAS2 TPST2 ULK3 JAK1 ZP1 RALBP1 YEATS4 CITED4 CBLN2 DAAM1 USP12-like RP11-101E3.5 RP11-49K24.9 EPHA7 NECAP2 CMTM7 SOX1 PABPC1 PHF21A XIRP1 AKAP2 SYTL1 C1D TMEM184B IL6RA TADA2A TRIB2 OSTN RECQL5 EYA4 SGTB SATB1 LLPH POLK RBM48 DNAJC5 SEMA7A CNOT2 NAPB WNT2B CASP6 STAT4 ZBTB17 EXOC2 STK11IP PPFIBP1 FGF1 SLC8A3 RASGRP3 IRAK4 MSN C3H2ORF43 SLX4IP FBXO7 LUZP2 ALDH1A1 WAPAL RPIA SARS RNF103 ARFGAP1 TEAD4 CLTC AZIN1 RALGAPB CENPH MYH1B RTN4 ST3GAL3 ASNS CDC42 ZCRB1 CSRP3 NREP VDR B3GAT1 MYH11 SMIM4 TMEM41B UBXN10 SF3A1 FYTTD1 TMC2 PACSIN2 STRBP GAD1 TBL1X SLIT1 ST3GAL2 TMCO3 PCGF2 SLC22A7 SRPR FAM118B CDH17 NFIX FAM192A SNX3 PTPRO VCAN ARGLU1 DLX6 NFYA NCALD GLT1D1 GABRA1 TP53I11 BCL2 HPGDS G3BP1 ANP32B SOUL BRE TNFAIP1 LOC395647 WNT5A SGMS2 E2F5 AMD1 IPPK GFPT1 CD200 SEPT2L ARFGAP3 EPN2 ZC3H6 CD36 SLC26A5 PTBP1 CNIH1 SUV39H2 CHERP CLDND1 SDC2 LYPLA2 CPSF4 MFGE8 TBCA VSX2 EXOC6 PRRG1 TPRA1 SZT2 CAMKMT LOC431499 C26H6ORF106 PDPK1 ORC5 CDK6 CDA NME5 MAFF SERTAD2 UBN1 ATP6V0E2 CELA1 KCNA4 MAP1LC3B MBL2 STAU2 NOTCH1 CD80 RP11-20I23.1 NAT10 TSPAN1 RASSF3 EFHD1 ACLY CCR5 PPP6R3 CHD2 SLC17A9 HP1BP3 PKIG TMEM68 CDH20 DNAJB12 FMOD AGA CLTB PELI1 TMEM45A TRPC3 CFLAR SOCS4 PAX6 SCN9A TCEANC2 TBCK TSSC4 CACNA1S STXBP3 ITGB2 GTDC1 DYNLL2 BAK1 SLC39A13 YTHDC1 PAQR8 CETN1 LOC426385 PDLIM7 TMOD3 GPX7 LMO4 5-Sep PGR CDH11 STK11 MYO1C CTSL2 SERPINB6 C13H5ORF15 MGEA5 HIBCH ACADSB SSTR2 DEPDC6 C1H12ORF23 TNS1 CYP51A1 OPN5L1 GET4 PRKAA2 CASP14 PRC1 TNFRSF19 BTC CDKN1A ICMT ARFIP1 GART RORA IKZF5 RNF152 SMIM3 UBL7 DNMT3B DHRS11 DUSP10 LOC420849 KPNA1 LOC420419 RELL1 CSPG5 TWIST2 NEMP2 APCDD1 RGS4 ALG12 ARHGAP19 SNAP23 BRCA2 STX2 SENP8 TANK MGAT4C CTD-2116N17.1 GLOD4 KLHL13 FAT3 CRADD TMX4 8-Mar IL6ST FAM20B BLOC1S2 SGCB PLEKHB2 CDH5 ELAVL1 RPS6KB1 PSPH TNNI2 LMBRD2 ATP6V1G1 RNASEL MAFA TYRO3 FZD5 TBC1D23 MAP6 PARK7 FSHB SLC47A1 TRABD MGAT3 LMX1A CPA6 PHAX STK10 VPS50 RAB35 GTF2H4 MON2 DHRS3 ACTN1 NPC2 CRYL1 KNSTRN ACOT9 ZDHHC17 ZFHX3 MTPN STRAP ASB7 GHITM MAOA TRAF5 ZNF330 EDA2R ABCA1 RECQL PRLR GLUL ANO5 DOLK PSME3 TMEM170A SELO WASL ST6GALNAC2 FGFR2 ERI1 WRB NCOA4 JUP GPR39 FAM122A BCL2L1 FZD3 SUGP1 C2ORF88 FLOT2 GJA5 TNFRSF1A WDFY2 ECE1 PDS5B EGFL7 FEM1B APC2 SLC7A6OS LYRM9 SRSF5 PKM ANXA7 ELN AC025048.1 CSRP2 COL8A1 MTMR9 FOS FAM175B GLP2R FXYD6 GAPDH HRAS CNDP2 HCRT CTNNA2 6-Sep CACNA1B GLI2 LOC427470 CHAF1B ABHD13 CUL2 CLIP1 COL6A1 RAB14 MYL9 YBX1 MCL1 TNS3 FHL5 WBP4 PTPRZ1 SMPX ENSA RGS6 CD151 BASP1 POU4F3 TFEB ODC1 DDA1 ADARB1 C4H4ORF29 MPP5 RP11-403P17.5 KLHL20 BANP KIAA1191 OSBPL2 NINJ1 AAMP P4HA2 CANT1 RPL32 NOC2L MCCC1 MEF2A NGEF FBXO8 ZNF622 BPGM C1H7ORF60 CDV3 CD247 ING4 ATP6V0E1 WDR24 SLC51A SBSPON NFASC MALL POT1 ANGPTL2 PPP1R21 TMEM26 TMEM184C SNRNP200 MFAP3 SMAP2 CALM1 ARRDC1 ARHGEF3 NPPC FAM210A ARPP19 SYK HMGCR GALNT1 LDLRAD4 SET NR1H3 SRGAP1 SERPINI1 TMEM180 SCNN1A BTBD10 SULT1C3 VCP LYRM1 SMIM18 TRIM59 NT5C3A TBL1XR1 SLC38A4 TLR21 ABI1 TSKU LAMP2 KIF4A STRA13 CNR1 GSKIP PHF5A MTX3 FHIT DNAJA1 ENOX2 PROKR2 NHP2 UBE2G1 RGS9BP CD74 NFE2L1 RAD21 STAT5B ACVR2A LCP1 SORL1 VMO1 PAK1 ZFYVE1 RHOA ERBB3 TBC1D1 COX6C ANKRD27 CELF1 CLEC3A SNX16 SLC1A2 HAO1 MYLK2 H1F0 THY1 BIN1 ARIH2 MYH1C LOC100859427 FABP6 RNF139 FAM98A SERINC1 GAPVD1 EMB KCND2 VIT PDGFD NR2C1 FAM46C HMGCS1 NFIL3 TMEM30A PPM1M AHCYL1 KCTD2 NCAN STAT1 GAS2L3 FBXL16 IL2RA SEPSECS SUPT3H PRKAR1A PMS1 CD200R1L KTN1 MCEE TMED8 CHST10 ASIP COG4 FGF19 NDEL1 ZNF512B STX6 C12ORF57 ATF4 CAMK2A PCMTD1 SDK1 RNF14 FANCC RTN1 RPS14 LCLAT1 FAM3C SETD3 MLNR COL9A2 PAPD7 COL22A1 CNGA3 HDAC4 IPO13 BORA TTL UBE3B COL17A1 GABPA ESF1 RBMS1 KCNH6 NGF SLC25A6 RNF4 SLC35A3 RAB10 MAD2L2 SH3GLB1 BMP4 LPP MAPRE1 CCNK LOC770548 YRK FADS2 PCK1 EHMT1 STIM1 PXN ELMO3 PPDPF LOC422926 USP45 GJC2 TCEB1 T FEN1 MAGI3 ELOVL1 SUCLG1 SZRD1 KCTD7 RFNG PPME1 ADCK3 CNTRL SYT12 ABCC1 SLCO4A1 BSG PTGS2 EIF5A2 ENTPD2 HOOK1 SOCS2 FZD6 MORN4 HMGN2 MAPK6 HOXB4 NUDT5 TAF5 HYAL6 DPH1 FBXW11 MAPT TMEM120B MTMR3 ATP1A1 CIAPIN1 XIAP STUB1 ARF1 SRSF11 ENHO PUF60 TMEM138 FKBP8 YWHAB CBFB CPNE1 PPP1R2 GTF2E2 CPSF6 PIK3CB RBM12B NAA35 RABGAP1L SASS6 CHAC1 ACOX1 CAT TMLHE SLC31A2 PIGR CYTH1 WASH1 NOS2 SGPL1 SLC39A9 TNFAIP8L1 CDC25A PAFAH1B2 SEPP1 UBE2R2 RLIM ARIH1 LOC100502566 P2RX5 UGDH NKIRAS2 RBM19 C1H11ORF54 KIF3B INO80 BMP10 ARHGDIA KDM5B CMPK1 NUP93 ERLIN1 AMPD3 BRSK2 CALB1 GCHFR FBXW5 MET MVB12B GNB1L PLEKHO1 PFKP ABRACL PRNP ANKRD40 YWHAG GOSR1 PIK3R5 RP11-290H9.2 ELF1 RP5-1021I20.4 FAM105A AARS2 RNASEH2B PODXL NAP1L4 TGFBR1 HMGB1 CAPN1 C20H20ORF24 RGS17 GFRA1 TBX22 APBB1IP YTHDF1 VPS41 15-Sep C7H2ORF76 SEC22B HABP2 SPINZ HAVCR1 ARMC1 HESX1 DCT MMACHC CLDN3 PLEKHF2 CACNA2D1 TNC RABL3 CATH1 TAPBPL CTD-2410N18.5 DNTT ABHD17B IMMP1L ACAA2 DEXI CECR1 MAP7 P2RY14 RFFL NARS RAC2 ARHGAP25 PPARD AGO4 TMOD4 UCHL3 LIFR POPDC3 NOP56 UBL3 HMG20A JMJD6 ANXA11 SLC37A3 BRAP GATA4 SLC16A3 LOC396531 CLOCK LMBRD1 IGF2 PTGES3 ENTPD8 UBXN2B FUT9 DRAXIN SLC6A9 PEX2 |
| gga-miR-365-3p | CANX COL8A1 MIB2 CTNNA2 FBXL16 ABHD13 TNFRSF19 PODXL POLK FBXW11 FHL5 CREB1 ADAM10 WASL PIK3CB CSNK1E CPSF6 CDC42SE2 CDH7 ITPR1 CTXN3 GAPVD1 HESX1 TBL1X THOC3 COL22A1 CREM PLEKHO1 NT5C2 6-Sep ENSA MPP5 CTSB GDF9 CMKLR1 GPR149 UGGT2 TNS3 ARIH1 MGAT4C NECAP2 CAPN1 NGF CBFB FHIT ACVR1 MAPK6 CCDC101 CD4 LPP CRYAB OAZ2 USP48 FBXO34 ACBD3 NCAN PLEKHJ1 ENTPD1 CDK2AP1 NRTN SGK196 DFNA5 SOCS5 SMIM5 PPM1M LEF1 C1H12ORF23 SUGP1 CCDC61 HRAS TSKU CPA6 FYB CCDC167 LOC396380 BORA PDE6H TNNI2 PEX13 YRK NOTCH1 CBLN2 UBXN2B GJC1 CSPG5 NME3 LUZP2 CDA NCOA1 MRPS26 HOXB4 PPARD DLX6 PAIP2 RGS19 HDAC7 S100A10 PAFAH1B1 TNC AANAT CTBP1 ABI1 PTN TMEM104 NFYA FAT3 RGS9BP GJA1 ARFGAP1 HAVCR1 ASIP GDPD5 EXOC2 RP1-309K20.6 DAAM1 JMJD6 BID STK11IP LCLAT1 EPN2 SLC25A22 DNAL4 KLHL13 IRX4 PACSIN2 FIGF ABCA1 CLINT1 KCNJ8 TRPC4AP OSTN LOC420419 PAFAH1B2 ANKRD27 KTN1 CKMT2 RAD52 EYA4 PRLR BEAN1 DEXI GTDC1 CHRM3 HABP2 TWIST2 STX6 RAB14 UGT8 RAF1 PEX2 TPRA1 SPPL2A AIDA EXD2 RP11-514O12.4 STK10 NCSTN CTDSPL2 RELA EMP1 TMOD4 B3GAT1 FAM20B YWHAZ APBB1IP ENOX2 INO80 CANT1 APCDD1 SEPSECS SERPINE2 PHC1 SLC39A13 CNGA3 LPAR2 LCP1 RREB1 EEF2 FANCC AC113404.1 MAOA ELAVL1 SET GJB1 FZD6 FUT9 FAM177A1 DDT HDAC4 CNPPD1 LDLRAD4 OAT PHKB PRRX2 C15H12ORF49 RALBP1 SYTL1 NR2C1 NR1H3 DDX6 ZFAND6 CSNK1D SUMO3 CD200 PRKCI SLC1A2 PDS5B DRAXIN CLDND1 GLP2R ADD1 TBC1D1 P4HA2 TNFAIP8L1 CNTN5 OCLN GTF2H5 TAPBPL LOC426385 NADK GANC FGFBP2 FOXO1 HIPK3 BRAP TP53I11 CDCA7L HMGA1 NCALD BRINP1 RP11-101E3.5 CLIP1 EPB41 SUB1 WBP4 COL12A1 SLC17A9 MTMR3 USP45 GJC2 DPH1 EXOC6 CD151 VPS41 IL21R XIRP1 8-Mar BRSK2 KPNA1 KIF5C LOC424740 MGLL SLC16A1 FAM126A RASSF2 GREM1 P4HA1 SULT1C3 BBS4 ETV1 DCAF12 STIM1 NEUROG1 TRMU BTC GJA5 TNFRSF1A CDH6 RHOA FZD4 FAM76A BASP1 LOXL2 STAU2 GALNT6 PCMTD1 LOC420849 SMPX LOC428335 JAK1 TOMM6 VAMP7 SNRNP200 SUPT3H RASSF5 BAZ2B ABLIM1 PIK3R5 LUM MFAP3 TPM2 SSR2 OPNP ASNS SGK3 HHATL ZNF512B NKX2-6 PNPLA6 CD80 DHRS11 C1GALT1 HCLS1 STK11 CD200R1L CDH20 RGS17 TAF5 CRYL1 SGK1 OPN5L1 RNF141 SATB1 CSRP2 MMP13 PAQR8 SH3BP2 VIT CDH5 RABGAP1L P2RX5 EEF1A2 |
| gga-miR-449b-5p | CALM1 MIB2 FABP6 RDH10 TNFSF10 RABL3 ENTPD1 SOCS5 DAP CHUNK-1 EPN2 GLT8D1 AANAT USP37 NAIF1 PSMD1 TRH NOC2L G3BP1 OCM SLC12A7 GFOD2 SLC1A2 UBL3 PBLD CYP2C45 EXOC3 TRPV4 FAM76A DOHH ZSWIM7 ABI1 ORMDL2 SCYL3 PKM PRIMPOL BBS4 NUP50 TBX6 CLIP1 SMIM8 SSRP1 KLHL13 MARCKSL1 TBL1X ART4 PLEKHO1 HIF1A BSX CDH11 ALDH1A2 SRSF11 CPA6 RPRD1A SFTPA1 EXOC5 PIK3CD C2ORF88 CD79B EIF2B3 VCAN PPP4R2 HOOK1 TNFAIP8L1 CHST3 STRBP ITGA9 LAMB2 ZFYVE1 FECH ENTPD2 PSME3 SRC IRAK2 MPZL2 CDKN1A PLOD1 GLRX5 SNN CLTB ZNF302 ID1 TNFAIP6 CTBP1 CDX2 MALL CD40 COX6C GNOT1 KLHL15 F2 MRPS9 AL158801.1 USP48 RAD21 SOX17 EHD3 ACLY SALL4 FKBP4 MID1 TMEM208 YIPF1 CNTN5 KTN1 ANKRD16 NTS TMED3 FZD7 FAM222B APOV1 NDEL1 PACSIN2 SDC2 LY75 EDA2R AvBD8 PLK1 NCL FAM129A SH3BGRL3 FYTTD1 OPTC MAPT FAM46A FLOT2 CAV3 PTPN11 RHOBTB1 TCP11L2 CD247 CPZ SNRPG RBM22 MBD3 CYP2D6 UFSP2 MGEA5 WNT8A LOC100858504 CHRNA6 B3GAT1 EPHA7 TMEM56 TNFAIP8 CELA2A MICAL1 FGL1 HVCN1 TRIM27.1 SMC4 PDPK1 K123 CHORDC1 GALNT1 VPS45 NUDT1 SELO HAUS2 ARHGDIB DPM2 PNRC1 NOS2 YBX3 MYF6 IGF2R C12H3ORF37 CDK10 HZGJ SERBP1 SNX2 YIPF7 DHFR SLC40A1 LBX3 ABCC1 MAPK9 GOLGA7 SPG21 TMEM30A CPSF2 SRSF7 SLC9A4 SMIM12 MYO1F TMED8 CAV1 ANXA6 GOSR1 RASA3 KIF11 ORC1 LOC431499 B4GALT2 B3GAT2 GJA4 CYTH1 ENOX2 BAZ2B PPP2R2D RPAP3 LOC772096 SYNM GLI2 SLC9A8 STAU2 NCLN SOD1 WNT5A AGO4 UGT8 NEK7 RPIA VDHAP POGLUT1 FAM45A AMBP MTMR8 C20H20ORF24 RRN3 UGDH CETP NELFA PREP P2RX1 TAPBPL FGF19 HSD11B1L CBX3 ATP1B4 ORAI2 HPCAL1 IFNAR1 NTRK3 RPA2 CYP11A1 ATAD5 RSFR FAM86A NUMB CAMK2D TRAFD1 JAM2 ACVR2A ANKRD40 S100A9 ATP6V0B MRPS25 MOV10 LOC693265 LRRC28 CNPPD1 RP11-152F13.10 PHTF2 TSKU FOXA2 ST6GALNAC1 RNF141 RNF11 UTS2B RGS4 UCK2 COX16 BLOC1S5 TPH1 9-Sep MYL4 CDH1 BMP10 TSPO RAP2C CD200R1L COL6A3 SEPP1 CNP FOCAD RHOJ NAP1L4 ASB13 PNAT3 LXN ZIC1 VGLL2 CD36 C10H15ORF59 DYNLL2 SNRNP200 DCAF7 ABHD17B JAK1 NECAB1 ARFIP1 AGPAT9 UBE2V1 GNB5 BARX1 TGFBR2 NEURL1 C1GALT1 SGOL1 BMP4 LDB2 SPTLC2 ULK3 SPP2 NKAP SRF ID3 AP3S1 GEM CAMK2A CMPK1 APELA IMMP1L CABP2 ATP6V0E2 GNGT2 EPS15 RUNDC3B CLTC TNFRSF19 GFRA2 PRPSAP2 YIPF3 ELMO3 HNRNPR AKR1B1L USP6NL GPR146 FTO THRB ADARB1 CHTF8 ENO1 CKAP5 NCSTN DYNC1LI2 YWHAQ CCT6A TMEM229B BMPER CD4 SAT1 ZCCHC6 RPL9 PIP4K2A PPME1 SKP2 LIG4 TAF5 NFASC METAP2 SZRD1 PRRC1 CLP1 GPER1 C1D MID1IP1 SEMA3D ECE1 PPFIBP1 CCNL2 FBXW11 COX14 DTWD2 LOC100859722 HMGA2 TFAP2B FAM49A ARHGAP19 EDNRA BCAP29 SLC9A3R1 EIF1 SGPL1 PCNP CTSL2 SPPL2A KPNA2 ADD1 ATP5C1 ATP13A4 RAD52 AC113404.1 USPL1 ZNF767 MRPL48 RRBP1 CNTRL MON2 SGTB CCL19 NFIB CRYBB3 INTS7 TSSC1 CNOT2 TYRP1 PPP1R7 TMEM184B ETS1 MBD4 NUTF2 CWC15 YEATS4 ALKBH3 LOC100857579 RGS17 FOXN2 MPP5 YWHAE RELL1 MPPED2 PTPRA ARIH2 EPHA3 SLC18B1 PRKAA2 LOC425137 HMHA1 RHCE LOC395991 LMO4 MBP TADA2A FAM98A GCM1 TMEM230 PLEKHB1 PNRC2 PLCZ1 GLP2R KANK1 SOSTDC1 BRSK2 MFN1 CFAP20 SGCB HINT1 FLNB ACVR1 NADK PODXL ELOVL6 NR3C2 SPPL2B RFTN1 CD72 LOC428335 SNX3 HBE1 E2F4 LCLAT1 RRAS2 ANK3 LHX9 LOC426913 MSN C5H11ORF58 STAT4 FHIT LOC419851 CBLN2 GINS1 SRSF3 BTN1A1 SUPV3L1 MELK LRRC57 DHCR7 NFE2L1 C1H2ORF49 GSTO1 CHD2 RASL10A POLDIP3 TARDBP BCL10 INSIG1 DCN MCCC1 H1F0 SLC2A2 DLST FPGT LMNB1 QARS AMIGO2 NREP SELK MAP6 PAX9 NFKBIZ TP53INP1 NRXN1 VAV2 ESF1 WRB EXOC8 HAS2 PHKG1 ACP2 RNF220 PXN C1H11ORF54 ST3GAL5 C26H6ORF106 DAD1 CCDC93 GSTT1 NBL1 TEAD1 LOC421740 TAL1 GTF3C5 B3GNT2 CCNE1 FANCC SF3A1 GNAL WSB1 CYTH4 ST3GAL2 POMT1 SRGAP1 MCM8 SLC31A2 TMCO3 MSGN1 PPP2R2A ACOT9 FAM210A ACAP2 MAGI3 COPE CAT CRHR1 PBRM1 EIF6 ANGEL2 SGMS2 ABCF2 BRD8 SBSPON AP4B1 SLC16A1 EIF2S2 FOXM1 OVALX STAT5B IL1RN SPP1 NRG1 C10H15ORF61 RBM12 YPEL5 CLOCK IL18R1 FYB TMEM104 PPP1CC KNSTRN WAPAL COPS8 DPYSL4 ZDHHC21 CCDC101 VPS33B NMU MCFD2 PHACTR1 XIAP ARPC5 SCD MTIF2 KDSR RASGEF1C FOXC1 UBE3C ETV6 PTPRO CSRP1 EPB41 RAB28 TAGLN PRMT7 GLUL HS6ST1 PSME4 NGEF TADA1 CDCA7L SLC25A26 ALDOC REL SIKE1 PELO MZT1 MRPS26 NMB ADCK1 ATF4 C11ORF31 PIK3R5 CIDEC STK17A IMPG2 HDAC4 ARIH1 ACSL1 ASB3 IFI27L2 RBM5 WDR24 C1QTNF2 LFNG ATP13A2 DNAJC6 KCTD2 ASS1 ARNT TREM-B1 ACADSB BZW2 SBNO1 RNASEH2B TOP1MT TUBB2B PPP1R9B RNF13 RAD51D ATF7IP SYPL1 FSTL1 PLAG1 VMA21 NRK BCL2L1 GSTA3 ADAM33 BLEC1 UBL7 WWP2 AR PRR5 DUT STUB1 XKR8 TMEM41B PDE3B TNR CAMKMT PALM FTL NPAS2 VDR ANG METTL9 TTC27 LMBRD2 CSF3 NKX2-6 FAM53A FLT4 CENPF EIF5A2 DR1 AGO3 UBE2V2 CFAP36 SMIM19 FGFR1 MIER1 HOPX SNRPD3 PPP1R12B FSHB DERA CHRDL1 LOC416354 PRKCI CD24 PCSK7 RP5-1021I20.4 TMEM254 AKAP17A EYA4 BZW1 ARG2 RAB9A LSP1 ATP6V0E1 LRRN1 BRCA1 CPT2 CDC42SE2 TMEM26 KLHDC2 RRP12 CREBL2 PPARG UBIAD1 TLDC1 NRGN SARS RECQL5 CASP2 THOC3 H2AFY COLEC12 BUB3 BUB1 FAM118B C7H2ORF76 CDR2 FZD1 GUCA2B ETFDH IGSF1 FADS2 FAM214A PLAC9 ACBD6 TMEM38A C18ORF42 DCBLD2 CBLN4 AARS C1H12ORF73 PCASP2 NFU1 GET4 PDE6H YY1 ATG7 ARFGAP3 IRF2 GABRA1 COG7 HSPA2 SERHL2 CCDC6 ANGPT1L PFKP CHST10 PARN RP11-49K24.9 RUNX1 TBK1 LGI1 CDKN1B PAPOLA TSC22D3 C4H4ORF29 TSPAN8 TSSC4 ELF1 SYT12 CTNNBIP1 ZNF622 PUF60 CCR8 DNAJB12 ITGB1BP3 PTGES3 RPL32 PBK CBFA2T3 RTN4 SAMM50 MYH1B PDGFC AKAP9 NDE1 ACTC1 PCGF5 MTPN LOC100859427 AGA EEF2 GPR89B APOA1 MLNR IFITM5 ZCCHC17 LYRM9 TRPC4AP SHFM1 PREPL FOLR1 FAT3 LSG1 REEP5 FBLN1 WBP2 PITX2 PAFAH1B2 VGLL4 ZW10 PIGA TULP1 CBL PLS3 ZFYVE19 LEF1 MATR3 ILK RP3-461F17.3 KLHL24 RBMX PTPN6 TMEM237 BCMO1 HIPK3 CHN1 ZBTB2 CRYZ NTN1 MEPE EPHA1 ANXA1 MRPS17 CASP6 PHLDA2 FIGF SLIT1 SOX10 CRIM1 TIMM9 GORASP1 DRG2 HAO1 ARMC1 XPO5 STX6 MTMR9 OPA1 TRIP13 AAMP FOXO1 BASP1 NDP GIP TBCK ADAT1 KIF2A SCOC CACNA2D1 LOC776816 CCPG1 LOC395159 STK32A CKS1B RAB11B ARHGEF39 CAB39L BFSP1 HGF LOC769139 LPP MTA1 FAM192A LOC422090 RPL13 SYTL1 USO1 SERPINB10 MOB4 MPDZ NPHP1 ZFP64 MAFA C1H7ORF60 SPINW LOC420419 BORCS7 BIN1 NT5C3B HP1BP3 CALB1 PLEKHJ1 LOC426914 CCK HOXB5 DNAJC2 ARHGAP26 DDX3X CRABP1 ESRP2 C13H5ORF15 PAK1 MRPL34 FTSJ3 RAE1 BTBD10 SLC15A1 ATP6AP1 PEX11G TMEM189 AMY1A GPR137B SGK1 MCM6 PNPLA6 NFKBIA KPNA1 LOC769121 PPP2R5C OTC RSU1 SOCS3 INTS2 TMEM164 POLE3 CNTN1 CNBP NANS POT1 FRZB HYAL6 SETD4 ANXA11 C1H11ORF73 LHX8 CD80 NFKB1 IMP4 LBFABP MYH11 PRRG1 RIPK2 NAV3 GSKIP RRM1 CRELD2 GATSL2 KDR MYH1A ENO2 ATP6V0A4 MAP3K14 MEOX1 SERPINI1 PI15 CACNG3 RRH KLF6 RPS14 SWAP70 PNAT10 TBX4 TMEM141 TMEM45A CHRNA8 CTSB C7H2ORF69 PRKAA1 SLCO4A1 LYRM1 VNN1 CRADD SMIM7 MFAP1 GRK4 TMEM70 TMED11 FGFR1OP2 SYNJ2BP CKB BICC1 MAP7 UBXN2A SMIM15 CPQ KBTBD4 PPP2CA MAP2K5 LYG2 PARPBP CRISPLD1 FAM213A ANAPC10 COG4 CIRBP LMBR1 RMI1 ASH2L C17H9ORF16 MYO5A RGS7 SCNN1A SSU72 TDRD7 COX4I1 MGST1 EXOC6 STK11 MRPL37 RP11-145E5.5 GNG13 PTPRZ1 SMPDL3B DCTN3 PLAC8L1 LRRK2 TIMM8A PPP2R4 SNRPB GJC2 TCF12 TMEM184C UCP3 STRA6 GATA4 TTLL5 SUV39H2 SAMD13 CNIH1 FAM105A RGS6 ZDHHC18 NFYA INHBA ZNF800 RPS29 RPL35A GABRB3 XPOT HERC2 SLC26A6 IL9 TBL3 LOC417800 LOC396479 NFRKB SEPSECS MIP ZNF706 MAPRE1 HRAS HPS1 COL17A1 KCNMA1 IFNGR2 PROM1 SSB ZP2 ADH6 RAC2 C11H19ORF40 EIF4A2 PPARD CHIC1 HIC1 XPA HSPA8 CDA ERGIC2 FGFR3 ELK3 GTSE1 STAR QRICH1 TMEM106B DYRK2 ATP4B SLC38A4 MXD1 CEP19 SRRD TAF2 INIP HNF1A LSM5 ARGLU1 VSX2 SLC39A13 NECAB3 CR1L SFRP1 BF2 CTDSPL2 PITX1 CPNE2 CRK ZNF410 LYN KRIT1 ASB9 DYX1C1 SATB1 PCMT1 DDA1 NCAPD2 CASQ2 CCDC127 NGF UBAC1 SFR1 TPST2 ARL8A SPATS2L RALGAPB EIF2D NELFB INVS FAM26E TRPM7 RASSF5 SERTAD2 HIBCH OPN5L1 CYP51A1 KPNA4 TFEB TMED5 IGF2BP1 DNAJC5 ORAOV1 TCOF1 RRAD TSPAN12 MORN4 IFT140 NR2E1 PLIN2 KLHL20 PDLIM7 CANX TSEN2 AGTPBP1 NAA50 FAM103A1 BHLHE40 AZIN1 FLI1 SEC31A DDX6 NFIL3 C8H1ORF27 SOUL OVAL TCEANC2 EFTUD1 FAM172A RBP4B IL10RA TXNRD3 MYLK2 UGGT2 BMPR1B PDCD5 PIP5K1A RAB11A SLC7A9 DYM EXOC2 GPR143 SPATA2 DIO1 ERLIN1 CHAC2 CXCR1 MRPL38 DNASE1 PHAX PTDSS1 PRORSD1 HSPA13 C11H16ORF70 HSBP1L1 LOC422926 BRCA2 NR5A1 RTTN DPYS PDE6G SDF4 CLEC3B RPL15 P2RY14 TOB2 MITF LIFR PSMD14 JMJD4 MEF2BNB RHAG PIK3CB SOCS2 LYSMD2 USP1 MYH9 OXNAD1 NAPB TBC1D15 CSF3R SCPEP1 ASNSD1 PDE4B CD44 SPARC EFHC2 RPA1 JAK2 |
| gga-miR-449c-3p | MTHFD2 SUPV3L1 NR1D2 CDK6 ARPC4 VSX2 BRSK2 NFKBIA KIF18B UBE2G1 NDUFA4 TRIM27.1 RRAD SOCS2 SMARCB1 PERP2 STX17 FGF1 RAB3GAP2 MORN2 DAP PDIA3 SALL1 MYO5A FAM20B CREB1 PCMT1L DHFR FAM53A ICMT ANXA7 ACOT9 LHX1 COPE BRD8 BRAP HDAC7 TOLLIP OVAL SIX1 CSRP1 ST6GALNAC4 CRABP1 FAM60A PPARD IL5RA HIF1A AGTPBP1 MAOA RABGAP1L ESR2 DPF3 DSTN IFIH1 CSNK1D RP11-77K12.7 SDR42E2 COL9A3 ZFYVE1 MET NEUROG1 8-Mar LPGAT1 LOC416354 NIP7 TASP1 BTN1A1 PYGO1 VPS53 OLFM1 DDX6 EXOSC2 MMP9 CPA6 CD86 PAFAH1B2 CASQ2 HMGB2 ARFGAP2 LCMT1 ZFAND5 STX2 MAFB SLC37A3 LOC100858504 FBXO8 STOML1 C28H19ORF10 RANGAP1 PDP1 RLN1 PDLIM7 ARMC1 LPAR6 C26H6ORF106 RAP1GAP2 HMGCR CFLAR MAFA SLC25A26 ATP2A3 VPS45 CBFA2T3 BORCS7 RFTN1 GINM1 TRIM59 NOTCH2 ARF1 TXNRD3 MYOM2 THYN1 CDC42 TANGO2 GBE RNF185 BIN2 DLX6 AGPAT9 |
| gga-miR-449c-5p | C1H21ORF33 DDA1 EMC3 LOC419851 LOC427470 C5H11ORF96 NGEF DOLPP1 CATH1 BET1 OVALY SAT1 TRH OCLN EPHA7 ASNS SQLE FOLR1 IFIH1 TBL1XR1 FBXL21 SLC16A3 UBL3 STK11 HSPA5 PRPF3 ENTPD6 FAM65B FOXO1 PTPRC NRGN 15-Sep NAPB RBL2 B4GALT6 LIFR POLK GMFB ADORA2B FAM114A1 DPYS MAFF ADCK3 DMD FIBIN MYH1B GAS2L3 CTD-2116N17.1 FYB CDH17 SNRPG AvBD1 SIVA1 FSHR RBBP4 KHDRBS1 TNFAIP8 CYB5B TMEM39B BCL2L1 HSPA2 EDNRA EPB41 K123 KIF4A HSBP1L1 TMEM30A PFN2 YIPF7 YEATS4 IGF1R NR2C1 MCL1 FAP IFT140 RLN1 PRRX2 GJC2 SERINC3 YTHDF3 RAN WDSUB1 GINM1 LUC7L3 LIMK2 FZD3 GPR146 B3GAT2 RGS18 FBXW5 WWP1 TMEM123 KANK1 DYNC1LI2 BMP5 SOX10 HMBOX1 RNF103 CKAP5 WDR70 VDAC2 FZD1 PRELID1 PAK1 ANP32E MRPL15 TP53I11 TCEANC2 DAGLB XKR8 ST3GAL3 BCL2 LAPTM4A SRSF5A ESR2 BORCS7 ORC3 GNG13 FKBP8 NIP7 TNFAIP1 PPDPF BHLHE22 ANGPT2 BORCS5 ZFP64 RER1 ST13 FAM18B1 PDLIM5 FZD4 AMPD3 FLOT2 SLC22A7 PSEN1 TRDMT1 BIK TRA2B GLT1D1 CNTF TRAF7 5-Sep FSHB SPP2 UGP2 ANGPT1L CANX MSX1 GCLM HARS AVEN USP12-like ETV6 GANC IRF1 MRPS25 RPL6 XPO5 EIF2D SKIL YWHAZ TNNT3 C14H17ORF103 ELK3 TLN1 TBX5 FZR1 ADARB1 LOC100859427 LOC100502566 ZNF384 TMPO NOTCH1 HDAC9 MYL3 NREP SERPINE2 LSS PIK3CA FAM46A SSRP1 BMPR2 PIK3R5 COMMD8 COL14A1 EXOSC2 ASB12 PHF20L1 UBIAD1 MYLK2 CTNNA2 LOC396380 TRAFD1 ANO5 LHX1 LPAR4 PAX6 IRF2 ASF1A RASGEF1C COMMD10 HSPA9 GHSR SLC35E3 POLR3H OPTN WIF1 S100A1 PSMC5 CETP TFAP2A AAMDC S100Z RASD1 PSMD14 GTF2E2 PREP PKIA PTRH2 NCAPH CD3E CYP2D6 ZNF800 SUPV3L1 RPS6KA1 STRBP TLE4 CCNC STAU2 C5H15ORF41 TRPV4 CLK2 GTDC1 CSNK1D LHCGR MST4 ITPR1 UBE2K LOC769486 PLS1 TH IFNGR2 RS1 EPHX2 RBM25 CELA2A PRKAB2 CD151 RP11-514O12.4 RRP7A DUSP4 CTDSPL2 AARS2 HIC2 CLDN1 PEX2 CSDE1 GET4 EDA2R SLMO2 KLHL13 RAB24 BKJ RRAS2 XPO1 TMIGD1 HHATL TGS1 HVCN1 WDR24 PPP1R3E TAF1A IL10RA OXNAD1 FTH1 PPP2R2A LOC417192 APOA4 SLC38A2 C15H12ORF65 OCM EIF1AY SLC26A6 CCK TRAM1 NHLH1 PACSIN2 RBMX CX3CL1 CECR1 TNFRSF8 VPS35 NAP1L4 SHOX EPT1 ERLIN1 NKX2-6 PRR5 PIGR C1H7ORF60 GATAD2A CHMP4B HDAC11 RP4-613B23.5 CYP1A4 TXN2 MMP27 CAMKMT ACSL1 AP1S3 ACP2 AP4B1 PCNP MID1 ADH6 GJD2 SCNN1A PNRC1 ARF1 LOC425137 LOC420716 PEX5 TRPM7 FHL5 COG7 PLK1 PTPN2 UTS2B GLT8D1 MOXD1 CCNE2 AKAP17A ELL PLN PIP5K1B ATP4B NRXN1 GPR149 MEOX1 B3GNT2 STXBP3 PDE6H LHX8 PSMD4 A4GALT KCNJ8 RIOK2 PLEKHA2 GABRA1 PDCD2 TWSG1 RAB3GAP2 CDH5 TLR4 SZRD1 CIR1 FABP6 TM6SF1 TNC TOMM6 RPRD1A GNOT1 PAPOLA GTF2A1 CPA2 SNCB RAB8A GLRX KPNA1 MTMR9 RP11-371E8.4 PEX11G PPFIBP1 USPL1 MAPK9 DCTD GPR143 LIMD2 NCOA1 BEAN1 CARD11 ACACA C1H12ORF73 RGS19 CHN1 ST3GAL5 BLNK SAMM50 LBH HIPK3 RHOC CPSF2 GPRIN2 OPTC CD8B LOC416354 VGLL2 CHP1 XPOT TSC22D3 SNN GNRHR BLEC1 IFT81 ZNF706 FBXW2 THOC5 CTNNB1 SDF4 TNFRSF19 PABPC1 CNOT1 NEDD1 TMEM121 EXOC2 PSPH CASP18 SCIN STMN1 TCF15 ASS1 MFGE8 ELOVL1 COL8A1 SUCLG1 DHODH PPME1 NELL2 HSD3B2 YME1L1 NEUROG2 DHRS7C AL158801.1 PRC1 SLC17A9 E2F5 RALGAPA1 NADK P4HB RTN1 PSMD10 LYRM2 ITGB1BP3 CFDP1 C26H6ORF89 MSANTD2 ALDH3A2 CCR5 GDF9 SDR42E2 RAP1GAP2 GIGYF2 CBX1 BIRC2 BF2 C11H16ORF70 AHR CHCHD4 CHUNK-1 P2RY14 BPNT1 RAB10 CABP2 LDLRAD4 EXT2 CNR1 CCDC101 HINT1 KIF18B LRRN1 SOCS2 UTP15 RABL3 ACLY PTPN11 TMOD4 BTD PYROXD1 STK40 RAB19 VMA21 REL ZP1 C26H6ORF106 LOC420160 EPS15 SURF6 GPR89B MRPS6 SERPINA4 VPS50 AvBD10 PLIN2 TOP1 C7ORF73 VPS41 MGST1 HMGCS1 SVIP PTPN1 GRIA2 NRG4 MLX FXYD6 TSN SNX20 CREB1 RRM1 CRYBB3 MYL9 DIO1 C11H16ORF87 F13A1 CD72 CHRDL1 SSR1 LIN52 ATP1B3 UQCRH BSX G0S2 GFRA1 TRIM59 CHST10 TIMD4 RQCD1 CKM NR5A1 LOC100858381 MGLL CD247 ANKHD1 FAM103A1 SLX4IP CRYBB2 HMGCR ANXA11 TMEM184C PCGF2 SNX24 EDN2 CHST3 ASCL1 NT5C3A AKR1A1 TSPO RHOB CD1C TTLL5 JAK2 EPCAM FOXM1 COPE HMGN1 BUB1B MYO1C DLX6 SAMD13 DCT TMED3 SMC2 UBE2H RP11-145E5.5 ANGPTL2 ID4 CCDC58 NFIX DIO3 CTSL2 MIER1 AKR1B1L CALD1 DNAJA1 ORMDL2 PARPBP ADAT1 KLHL15 TMCO3 TMEM129 SLC39A9 GALNT1 CDC25A NFKB1 BRCA2 RFXANK DPYSL4 ADAM9 METTL9 GTF2H4 PACSIN3 F2 NFE2L1 WAPAL PPP1R16B NRBP1 CYP11A1 RPS14 RAB40B SMIM4 SPINZ LMBRD1 KLHL2 LBFABP ST6GALNAC4 CMKLR1 SGK1 ASB3 NFIL3 MAL2 MPST STAT5B LUM KIF5C PYGB RBP UBN2 PPP2R5C STIM1 FZD5 GDI2 BRI3BP DLST MCAM MRRF ZFYVE21 TEX264 BLB1 LBX3 KLHL14 YWHAQ COX6C HYAL6 ENO1 AANAT LGI2 JMJD4 RNF4 TMED5 WNT6 ABHD13 YBX1 SLCO4A1 LOC770684 MRPS16 PPP2CA S100A10 IL21R ARFGAP3 SMIM8 ZNF639 TULP1 LOC431499 RNASEH2B MYBPC3 GRK4 SPTLC2 C1H21ORF91 PPP1CC DPM2 LOC100859616 NEK6 TMEM178B PIAS2 EXOC6 CLDND1 IMMP1L C2ORF88 FAM98A E2F4 PRKAA2 HOXB4 SERPINB6 LSG1 PMCH DYNC1I2 NAIF1 CWC22 MGAT4C N6AMT1 LYRM9 SH3BP2 SCYL2 GAPDH OAT CDR2 P2RX1 ST3GAL2 CYP1C1 PGD PDDC1 SERINC2 PBLD DHCR7 CEP19 GHRHR JAM2 GSTT1 HMG20A RNF185 SRSF1 IL1RN SFR1 KDM3A RCAN3 RB1 BZFP1 KIAA1191 8-Mar ZFYVE1 VTI1A DNAJA2 MKRN2 MON2 FAT3 C5H15ORF57 RASL10A B4GALT2 GPATCH2 LOC418667 FRS2 FLI1 PAAF1 IER3IP1 YARS GH RHOG RAB27A HNRNPH3 PUS7 SLC15A1 WDR45B INTS7 CRIM1 CLU MED9 CDK10 PSME4 SLC17A5 TM2D3 WWP2 GPATCH11 ZCRB1 YPEL5 IRAK4 CELF1 KLHDC4 TRMU LOC419429 CRIPT ARC PBK ALAD KIAA1671 LOC421975 C7H2ORF76 CCDC93 LOC769121 RPA2 CRIP1 ZBTB26 C1QTNF2 COG5 PRPF38A PTPRZ1 GALNT6 CNTN2 EPGN PANK4 ELN IFITM5 IMPG1 PODXL VAV2 SLC35B1 ABHD17C GCC1 HIF1A SSR2 PLEKHB2 LRRK2 CTD-2410N18.5 VTN SZT2 HBE1 NAA35 CYTH1 CAPZA1 ILK RRN3 USP45 CD24 PRPSAP2 ID1 ECI1 KIF20A AMY1A C3H6ORF120 ANXA7 YWHAH KDSR MUM1 CSNK2A2 RSRC2 MYO1F SLC39A13 UBE3B EDC3 GATSL2 TMEM175 CLIC2 YBX3 NAA50 XCL1 OPA1 TOB2 CHERP PBX3 PARK7 LYRM1 CREBL2 MBNL3 CENPH SPINW RP3-461F17.3 SYPL1 ARHGDIB CNIH1 DNMT3B NEUROG1 TIMP2 CR1L FAM177A1 KPNA2 GLYR1 PITX2 AICDA PSMD3 E2F1 RRP12 SCG5 ECE1 KCTD7 PRKCD MAP2K5 CKB GAL PDHX TAF5 PLIN1 CBFA2T3 PDGFB TADA1 ACADSB RASSF3 MRPS26 MRAS RHOA UGGT2 TACR1 RPL35A GCM1 MTFR1 ANXA6 MAVS ZFYVE19 HIBCH CSTB NAT MAP1LC3B NECAB1 PLCZ1 PTPRO VPS33B ZFHX3 KCNT1 ICMT N4BP3 UBXN2B RPA1 WNT8A TEC SRPR TSTA3 GEM NR2E3 PCBD1 SOCS5 VDR APOH CKMT2 LOC100858797 CREM COL12A1 MYH1C MAP2K1 GNB1L FPGT LRIG3 POPDC3 DMRT1 GIP RP11-290H9.2 SLC6A9 PLIN4 COX7A2 NAV3 PLAU UBE2G1 SERTAD2 PHACTR1 CYP46A1 C9ORF69 VTG1 TPM2 DKC1 RP11-403P17.5 HDAC7 ANKRD26 RAB14 GHRH PAPD7 LOC422926 UBLCP1 UPRT APC2 SLC35A3 EED AKAP9 WDR92 EHMT1 SLC7A9 UBAP1L GNB5 EMP1 ATF4 PDLIM3 PARP4 RAB11B MYL4 AACS CNTN5 PSME3 MMACHC COL6A3 GAPVD1 ATP6AP1 ARHGDIA KPNA4 VMO1 CCL20 SCG3 MICALL1 VSX1 ARHGAP19 ALKBH3 DCLRE1B DYNLT3 TXNDC5 GLCCI1 CYB5R2 BASP1 RPL3 GTF2H1 LOC100859148 OLA1 H1F0 SPATS2L PGA FKBP1B SRSF7 LOC429492 SMARCE1 WNT5A HHEX ARFGAP1 TFEB SEMA3A REXO2 PHF5A ST3GAL1 LZIC PNPLA6 SYTL1 WFDC1 PCM1 IVD AVD JMJD7 G3BP1 WDR89 PBRM1 OSBPL2 OLFM1 TMEM17 BCS1L HMGA1 SCN9A BMP15 TRIM27.1 |
| gga-miR-451 | ACLY LIN52 CNPPD1 TSKU SSB ZW10 PLAU EARS2 FAR1 FYB NUP50 TDRD7 EDN2 ARFIP1 PCMTD1 EYA4 MGEA5 SDC2 LCMT1 SPATS2L BRINP1 SASS6 ADARB1 CTSB MYLIP C20H20ORF24 SDK1 CDCA4 DNMT3B PIK3R5 NT5C2 TWIST2 ANGPTL2 USP6NL LRIG3 NGEF PCMT1 SEC22B FAM49A STRBP MTMR8 MFAP3 STX7 FBXW11 SRSF1 BASP1 PPHLN1 CD200R1L PBX3 GFPT1 LDHA GPR143 ETV6 MPP5 CALM1 SQLE FHL5 RHOA ERGIC2 UBXN10 SMIM5 GLP2R LCP1 NR2C1 LECT1 SLC25A15 C4H4ORF29 IPO13 HMG20A LOC427470 BAZ2B HAS2 BAK1 IFT81 FANCC SNN EPHA3 ZCCHC6 RNF7 SATB1 MAP2K5 GOLGA7 CNGA3 MTFR1 PSPH DMTF1 ASH2L NCSTN PELI1 CTNNA2 NSG1 MAOA IFITM5 LPL RGS18 OC3 CEBPG FBXO34 RIPK1 STAT5B CHRM3 IL21R B3GNT5 WBP2 SYNPR CBX4 APCDD1 RREB1 RALBP1 KIAA1191 IKZF5 NFIX EEF1A1 TBL1X AKAP2 CYB5A FZD3 CSPG5 MGME1 KLHL15 TMEM68 STK10 MTMR3 TOLLIP NFIL3 CDC42SE2 COL8A1 DAAM1 C15H12ORF49 CD82 STIM1 PERP2 CDKN1B MYD88 FLT4 HDAC11 SEC11A MGAT3 RASGRP3 ARNTL2 KPNA1 PDE6H LYSMD2 COPG1 UGT8 BRSK2 CDH6 NINJ1 HDAC9 RALGPS2 FUNDC1 ASIP KLHL13 MXD1 SERPINA4 MYO1C DNAJC6 NFIA FBXL21 CALD1 ELOVL1 YPEL2 SGTA SEPSECS C11H16ORF70 MBNL3 CPSF6 NCOA1 SYNGR3 HIPK3 ELAVL1 PRRG1 NGF PRKCD RAB11A ZNF330 DCAF7 LOC420419 GFRA2 PAFAH1B2 TFEB IRAK4 GJA1 LMO4 LPP CSRP1 JARID2 ALCAM BBS4 SDSL PPP1R2 DFNA5 RPIA LOC421975 CSNK1D FAM45A PREPL ELF1 C1H12ORF23 PUM1 RP11-196G11.1 AMD1 RGS17 AMIGO2 DCBLD2 NSUN4 NEIL2 CPT1A RP11-834C11.12 SNRNP200 FAT3 SIGIRR CSNK2A2 RAP1GAP2 RAD21 UBE2V2 PLN SNX16 TMEM180 ANKRD40 15-Sep UGP2 CACFD1 CDH20 FGFR2 ZDHHC21 FUT9 AZIN1 CAB39L PPP3CB FAM222B TEAD1 VPS41 DUT P4HA1 FHIT CRTAP ETFA PTBP1 FAM118B COMMD8 CDC42 TAPBPL TRA2A PDGFB ZFAND6 VCAN GDPD5 BRAP METTL16 MUM1 PAQR8 ARHGAP21 USP45 CDH17 TRIAP1 RELL1 FEM1B KDELR2 PTPRG NCALD VPS4B SYK ARIH1 CNTN5 CD200 KDSR RSL24D1 GTDC1 ZFP92 RAB8A NRG4 GBE SMIM12 PKM CPA6 ZNF706 SERPINE2 CLTB RIPK2 HPGDS MPZL2 YRK PEX2 TRABD HMGCS1 PPP1R9B PECR CUEDC2 GALNT1 XIRP1 GDI2 RNASEL TNS3 TIMM8A KCND2 MPPED2 PCMT1L PCM1 GNB1L YWHAH SLC26A5 HACD3 XKR8 CD44 TEAD4 CSF3 SLC1A2 PDS5B RNF185 LGI2 CLIP1 PTPN11 LCLAT1 RNF11 DUSP10 OAT ADD1 GNG11 CDH5 C12ORF57 HDAC4 IL16 DNAL4 FZD4 OPN5L1 MAFA PIK3CB SERTAD2 MBP SYTL1 ZDHHC17 FAM105A ENOX2 SOCS3 HABP4 VAMP7 GMNN RCAN3 ELOVL6 CFLAR PIGR |
| gga-miR-460b-5p | GTF2H5 AARS2 GTF2H4 EXOC6 CEBPG MTMR3 PARK7 FAM213A FUT9 ZCRB1 MPPED2 FAM126A ATP6V0E2 LEPR ELMO3 IL10RA EHMT1 KDELR3 PCASP2 BRINP1 DLST UBE2V2 NAP1L4 KHDRBS1 FAM192A ZNF706 CLPX CHD1 WDSUB1 SLU7 FHOD1 PTTG1 C1H2ORF49 AC113404.1 HDAC7 TMEM41B RHAG TMEM254 EPYC TRPM7 GJA4 ADAM9 ATF7IP SRP14 GMPS OPN1SW LIMK1 GNG5 CNTN5 LRIG3 GDPD5 PACSIN2 RORA HSBP1L1 PTDSS1 RASGRP3 RAB11A PARP1 IPPK LHFPL5 CLSTN1 SLC17A5 RAD51D LPGAT1 APC2 AAMP MMP9 GNPTG TDRD7 TOMM6 SPRY2 ST3GAL5 TEAD1 AvBD5 BTC HES5 SLC47A1 FLOT2 KLHL18 RP11-371E8.4 RELA RGS17 CDK2AP1 TCF15 ACLY ORAI2 PARP4 RALBP1 SENP8 IKZF5 GLT1D1 CSNK2A2 INSIG1 RPAP3 CLIC2 GPR149 TXN2 CCDC61 PDE6H NGEF C11H16ORF70 SRGAP1 LIMD2 DHRS3 PIK3CD HNMT ADCK3 AMPD3 GRIA2 SYNGR3 PDPK1 DAGLB FAM105A HPGDS UBE3C CYB5A CNGA3 LMBRD2 OCLN TPH1 TAPBPL PRC1 POU1F1 FBXO8 PAFAH1B1 NEURL1 TNFAIP8L1 PNPLA6 GLRX PPP2R2A DRG2 5-Mar TFEB CASP6 MIB2 ALOX5AP RREB1 NREP NR2C1 NBL1 GLUL SMIM18 ATP6V1D DCK HMGA1 STAU2 OLA1 PHTF2 EDA2R DHX38 PELI1 MAPRE2 TNFRSF1A CDC42 LOC771066 EPGN ASB9 CANX SPCS1 NUDC IHH JAK1 PROM1 GSKIP BRE GALK2 LOC100858504 GMPR RORB SLC1A2 CBLN2 AIDA EXOC2 CLIP1 BRAP FAM46A FZD6 CHERP CDPF1 TRIM55 PIP4K2A EMB FOXO1 ZBTB17 CETN1 FGFRL1 CPNE1 B3GAT1 PPP6R3 SGK196 FZD3 IFNAR1 CD200 FZD1 CECR1 LGALSL PHKB TMEM104 DEPDC6 FOXD1 GBE HHATL NRF1 CSRP2 TMEM68 WDR24 TNNI2 FAM49A CTGF HDAC4 PARPBP STK11IP BAK1 RP11-834C11.12 ARPC4 LBFABP LIFR CSNK1D CDH5 HIBCH PBRM1 NDEL1 ATP6V0E1 BBS2 CRMP1 RNF185 HIPK3 PEX2 PRKAA2 F2 NRTN PPP1R16B EGR1 MST4 MAP1LC3B RHOA YTHDC1 USP37 NUMB DYNLL2 ATP1B3 RALGPS2 STX7 ASIP TCEB1 TMEM208 TANGO2 BTN1A1 DLD ANK3 TYRO3 CPA5 TRPC4AP MPC1 C1ORF146 XPO4 WIPF1 TRPC1 NEIL2 VTN NRP1 ZCCHC6 ZNF622 CNOT1 NCALD AP1G1 SSTR4 5-Sep XIAP BID MRPL46 CTNNBIP1 CCNA2 SLC35G2 KCNA4 SLC31A2 PDHX ETV6 ACE PLEKHF2 SLC39A9 SNCA ACAN MRPL38 VPS53 IFT81 GTSE1 LOC770548 CARS MELK TRPC3 MXD1 BCL2L1 TSPAN6 PTPRU UBXN2B CLEC3A PREPL CSPG5 VSX2 AvBD8 KLF11 POLDIP3 SEMA3D MBOAT1 HOPX AGMAT PEX11G PLEKHM1 CCM2 TAF11 CLDND1 DNAJC12 ORC3 CCDC101 ST6GALNAC2 FAM114A1 MAGI2 TBC1D22A IGLL1 RP5-1021I20.4 PCMTD1 CD72 RAB33B RNH1 ATP2A3 HSD11B1L CLSPN PRLR HMG20A SYTL1 IFIH1 FZD7 MXI1 CRCP RLBP1 STAT4 ADIPOQ LOC396531 FANCC NFE2L2 SH3GLB1 NR1H3 ARHGAP19 AAR2 FBXO34 SLC8A3 ARF1 TMEM229B COL4A1 GFRA2 LEPROT GATAD2A OLFM1 LIG4 CCDC6 RP11-20I23.1 ANKRD16 MBD3 RSL24D1 OXCT1 PPP4R2 NFIX ECE1 SDR42E2 BCL6 STUB1 NFKB1 DNMT3B GTDC1 USP7 DCN LOC420419 PAPD7 SPP2 SLC9A4 CCR8 SAP130 RHOT1 STARD4 UBIAD1 MYLIP MGAT4C LYRM9 EEF1A2 CSRP1 MEAF6 USP45 LOC427470 CD1B LRRK2 HN1 P4HA1 PRRG1 SCG5 SYT12 SNN SLCO2A1 TP53INP1 EPHA1 CEPT1 APOC3 RAD21 MAPKAP1 AP3M1 RABGAP1L SMIM12 NUP50 SMIM5 TSPAN3 ANGPTL2 GET4 TM4SF1a PLCZ1 CTSS HOXB5 DEK AHR YWHAB CASP18 SPTSSB BRCA1 ACTG1 MRPS6 LOC100859039 TPRA1 CNR1 E2F1 HERC3 ST6GALNAC1 SEPW1 PCM1 N6AMT1 AC005943.2 PTBP1 NKX2-6 AHCTF1 NELFA TADA2A WASH1 APOV1 UBAC1 SLC40A1 DPP4 ACSBG2 LOC426913 ZFAND5 IMMP1L CHRNB2 NKIRAS2 BAZ2B YBX3 CLTB EPCAM DYM NECAP2 PPP1R2 YRK ITGB2 MYLK2 TMEM30A MEPE DFNA5 CD247 RNF14 CNDP2 HNRPK FYB AC025048.1 TACC3 CXCR1 PLEKHB2 CLOCK CCDC93 SLC39A13 ZNF335 GLCCI1 POLE3 CHD2 GORASP1 MTX3 RHCG CD151 FUBP1 COX16 CXCL14 FDX1 NHLH1 SEMA7A GPATCH11 CTPS2 CAV3 SLC25A6 AR FADS2 NME3 ST3GAL2 THADA COL3A1 FAM122A ARHGAP29 MAP6 RBM24 MBP ELAVL1 LIN52 CCDC167 LOC396380 GLI2 SNX16 HOXB4 ZDHHC8 ALG12 OLIG2 E2F6 PLA2G7 GALNT1 CDH7 FAM60A CHM MMP13 SGTA MVB12B RECQL SLC35A3 ALDH4A1 PSMG3 SIX2 IL6RA SELO PRRX2 TAPT1 RNF7 IFNGR2 SETD4 UCK2 SH3KBP1 RPS6KB1 TUBAL3 TMEM170A SERTAD2 CHRDL1 STX2 TRAPPC3 LUZP2 FAM65B ZFAND6 CHUNK-1 KCNJ8 PALMD VPS4B PRKAA1 YIPF3 LOC428335 XPA EHF HRAS LGI2 TRMU UQCC GART TTL NHP2 BIVM JMJD6 TRIM59 PLCXD1 UBE2H INCENP UBLCP1 ANXA11 BORA EYA2 GREM1 ZP2 ESR2 TMOD4 ORMDL2 FAR1 ENSA SLC16A1 NINJ1 ZBTB34 DCLRE1B TMEM230 CSRP3 PIGBOS1 RBM5 SYK RHNO1 LOC772071 RGS7 PIGR PAAF1 VPS18 MRPS33 RCHY1 DNAJB9 TRIM39.2 RRP7A IVD MFAP3 ORC5 RTN4 MRPL34 PLEKHA2 HMGB1 AMPH RAB35 SLC12A7 CCKAR CBFA2T3 USPL1 ATP5S RAF1 SERPINA1 RARRES2 CMTM7 PRKAR1A MYEOV2 GALNT6 CELF1 INO80 NCSTN NOC2L FAM76A GHRH CASP14 MINA PTPRO ANG MAPT BORCS5 KIAA0586 NFYA HCLS1 G0S2 CCDC28B SCIN SNX12 BHLHE22 KIF23 TSSC4 GJB1 MX1 PTGES3 PGR SGPL1 TXNL4B FHIT GJA5 RFFL DPYSL2 INTS2 NUDT5 SLC26A5 PLN SRC MAOA SMIM7 UBE2G1 YWHAG RP11-195F19.29 BG8 VPS41 HDAC9 PAIP2 ZNF384 TMEM173 SLC34A2 DAAM1 RFTN1 EPT1 NFE2L1 SPP1 LOC420860 CIAPIN1 PDDC1 IGFBP4 EYA4 NFASC MMACHC CMC2 GDF3 XRCC3 ZNF800 CTDSPL2 NR5A1 RUNDC3B TWIST2 YBX1 ANKRD40 ELL B4GALT2 HOXB3 TMEM121 MOB4 TPI1 C26H6ORF89 FLNB ALAD BET1L IGF2BP3 APCDD1 HMGCR KCTD7 ARHGAP25 SRP68 LOC426385 PFN2 ST3GAL3 KLHDC2 RASA3 PIK3CB BHLHE40 WDR18 OPTN ERBB4 FHL5 KLHL24 GPR107 TP53I11 ABLIM1 GJC1 TMED8 MLX N4BP3 BORCS7 YWHAQ RAG2 CD36 GCC1 RGS19 SOUL PDS5B PTX3 PLEKHB1 RPN2 E2F5 ZNF512B FAM222B LRRN1 CBX4 FYTTD1 COPB1 ADAL SLC16A3 SDCBP GAD1 RAC2 PDLIM4 CRH KRTAP10-4 MAL2 CTNNA2 PDE3B AANAT CDC42BPA ZFHX3 SAR1B XPO1 OAT FAT3 DRAXIN ALKBH3 CMPK1 SOCS3 CDH20 UBXN10 PLEKHO1 C1D LEF1 C1GALT1 YWHAZ CREB1 XIRP1 GATA4 FAM118B TMPO TRAF5 KRIT1 TBCA COL9A2 NCOA1 LZIC NECAB3 COG5 CUX1 CCR5 IRX4 ABHD13 VPS45 CRTAP PFKP LCP1 EARS2 DNASE1 C14H17ORF103 RAD52 SERPINI1 PUM1 ARHGDIA CHST3 APOD TRIM8 RNF152 PXN MICAL1 UCP3 RAN KERA DDA1 PAN2 ABRACL CD1C FBXL16 STRBP STK11 CATH1 TSPAN1 CNPPD1 APBB1IP CD276 CPZ BTG1 COX6C LOC420849 CDCA4 LGALS3 FEN1 GBAS MAGI3 MPZL2 CNTN2 TXNDC5 ELK3 EI24 LOC395095 LCLAT1 ADORA1 TOP1 ADIPOR2 PDLIM5 MAPRE1 ALDH1A1 ZBTB26 FABP6 YWHAH CSF3 CTSA TEAD4 MYL9 ACAD8 CYP24A1 MCEE PSME3 9-Sep CDH6 PPP1R3E ISLR2 ATM PPP2R4 BCMO1 C7H2ORF76 PRDX1 ASNS C20H20ORF24 CFAP36 ARID4A FBXW5 CALM1 C1H12ORF23 TSKU RP11-403P17.5 PSMD2 GNLY SULT1B1 PAQR8 CYTH4 SLC35E3 FAM102A RAP1GAP2 FXYD6 QRICH1 RRAGC COX4I1 ZDHHC21 MYH11 DENR LPAR4 FBXW11 SLC16A9 IRF5 BARX1 SCPEP1 ANXA7 UBE2R2 ULK3 ZNF767 BRSK2 NECAB1 MTHFD2 CRYBB1 RAB40B TAF5 SGCB CUEDC2 IL21R JMJD4 FZR1 MTPN C1S NDP ARMC1 SMPDL3B PRIMPOL TEX264 SMIM3 ALDOC RSFR SH3BP5 RP11-49K24.9 WDR45B ENO2 GGNBP2 RAB3IL1 CDK10 PCK1 PPARD DDT SMPX ALDH1A3 ARFGAP2 MYOM2 SBSPON CYP46A1 TLR4 CBFB CRIPT PEX5 KIF5C ENTPD2 CISD1 FAM45A NCOA4 HMHA1 TMOD3 RGS4 PIK3R5 ACACA MXD4 H1F0 SDK1 GNG2 TBX22 ETFA MEF2BNB HMGCS1 UBXN2A LOC418811 KIAA1467 WDR5 CLDN3 PAX6 CBX3 HESX1 SLMO2 CDH17 GFOD2 CD3E MLF2 AZIN1 NTMT1 ADCK1 MFGE8 TMEM11 DNTT DNAL4 NUDT19 DNAJC5 EGFL7 AAMDC AGO4 RNF4 DEXI ANKRD26 FOXD2 UBE2J1 SLX4IP RBMS1 FECH LYSMD3 FDFT1 TXNRD3 SEC31A PHACTR1 RGS16 ACP2 DCAF7 |
| gga-miR-1553-5p | AMPD3 ALDH1A2 HAS2 CFL2 DRAXIN FURIN SLC6A6 NONO PSME3 GPR149 DHFR DCUN1D1 CTLA4 GNB1L MAP1LC3B ENTPD1 SLC9A8 MLX MXD1 GPATCH2 DNPEP EIF5A2 ASIP ATP6V0C GJC1 KPNA4 ANKRD10 THY1 ATM MPPED2 SGK1 SGMS2 PERP2 CBLN4 IFI27L2 P2RY14 GAPVD1 FUT9 TANGO2 PHTF2 NDUFS1 ABI1 PRORSD1 RPS12 GLCCI1 DEXI CD5 SLC8A3 RIC8A CCM2 ZNF384 ATG5 SLC35E3 MGEA5 BRCA2 RCSD1 DCN C13H5ORF15 TSPAN1 ALDH3A2 YRK NAGA NOC2L HABP4 UGT8 FAP ZNF706 PLAC9 CKB TAF12 ELAVL1 NQO1 NGEF PTGES3 SLC12A7 C15H12ORF65 CNOT1 CYTH1 AZIN2 TBC1D22A NAIF1 GATAD2A ARID3B CDH17 SMIM5 LOC422249 CD99 COL17A1 MET CKM ARID4A NRBP1 MGME1 TMX4 LIN9 CTD-2410N18.5 SRGAP1 TNIP2 BHLHE22 RFC1 HSBP1L1 SLX4IP SMAP2 ENS-1 PCDH10 PAX6 LOC420209 MSGN1 EHMT1 ANXA7 DPM2 FAM18B1 HNRPK ZP2 DAZAP1 NFKBIA GUCA1B PPM1M UBAC2 WDR92 SPPL2A ECE1 NEK7 CHTF8 CCNL2 FBXL12 PSMC6 G3BP1 SERPINB6 SH3BP5 UGGT2 KIF5C SYTL1 AC113404.1 FSHR NOP56 CCL19 CRH TPM2 NCAN RPS14 ESR2 NAE1 TNFRSF13C EPN2 ZBTB34 MINA FXYD6 COX16 MESDC2 CAMK2D P2RX1 RAB10 COA7 NR3C2 PFKP PIGK IL6RA ZFHX3 CPSF6 SOUL PEX11G MMP9 KRIT1 METAP1 PABPC1 BGLAP PRMT3 NUDT21 LHFPL5 C7H2ORF76 ST13 RNF220 DIAPH1 TSPO ADIRF FZD3 FAM210A ASNSD1 ABCA1 MSRB3 RRP7A AC005943.2 MEAF6 HMG20A RBPMS2 LMO4 POU1F1 PRPF3 SLC30A4 SERPINA4 GLRX RGS9BP RSL24D1 PPP2R2D TSPAN15 MGLL HMGA1 NDC80 RIT1 PACSIN2 POPDC2 FOS SLC20A2 TMOD4 CASP14 YIPF4 GPX2 SOCS3 SOX11 SLIT2 COL6A2 TRH RB1 TCF12 CTPS2 RECQL PPP2R4 UQCC CALM1 ID4 CISH PARD3 LYSMD3 PPFIBP1 GFOD2 TPH1 MYBPH EGFR DUSP12 RAB11A MYOZ2 PRLH EDC3 RBBP7 MYO1C CNP YPEL5 NTMT1 TRIP13 FAM49A PIGR POLR3H SOX17 CACNA1D MOV10 SLC46A3 ATP4B ACAP2 KLHDC2 SLC9A3R1 CDC20 MORN2 TMEM123 PMP22 RGL1 AAR2 APOH ST3GAL2 ZFAND5 FLT4 FAM177A1 RCAN3 CTNNB1 DUSP4 PDGFB COG4 PRKAR1A AGO3 SLCO4A1 SCG5 GJC2 FLII ENSA KCTD2 DDB2 FAM76A NOTCH2 PHF21A CDKN1B TMEM180 CD247 ZNF410 LOC422926 BUB1B HRAS CNGA3 WNT9A VEGFA STRBP KBP MLNR CORO7 OSTF1 TMEM121 MBD3 ERGIC2 TLR21 SLC10A7 RAB35 PLEKHF2 NEK2 SERPINB10 PRPS2 SMPDL3B RUVBL1 CPNE1 KLHL2 TMEM41B WLS RASSF3 NRXN1 RP11-87C12.2 MELK GDPD5 TMEM30A NRG1 SRSF7 CHUK DPH1 MYL9 SERHL2 FYTTD1 INPP5K ACBD6 DNMT3B YARS SLC2A2 SLC22A7 IGF2 PUM1 PAQR8 MAP6 ZCCHC6 IRF2 AvBD5 ZNF639 AREG TRAFD1 IRX4 LEPR PEMT PSPH CERK LDB3 STUB1 PTH UBIAD1 ALDH1A3 TLN1 GBX2 DNAL1 TEAD4 PLEKHM1 SH3BP2 STK17B VPS4B C5H15ORF57 LIMK1 LOC431316 PWP1 CUL2 C3H6ORF203 ORC5 ALAD N4BP3 ARHGAP26 BID SSTR2 RASSF5 CDKN1A ANAPC10 FAS PDPK1 SETD4 E2F5 SCPEP1 COX15 BTC KCNAB1 EGFL7 PAN2 DMTF1 NRK WIPF1 PLA2R1 CDR2 TMED5 HDAC9 ASB8 LBX3 MCL1 ENOX2 RNF4 DAPP1 STX7 ACTG2 COX4I1 CD3E RANGAP1 YME1L1 HMGCS1 LGALS3 KIF20A JAK2 ASB12 PTDSS1 HES5 KLHL20 ACACA SMIM3 B3GAT1 GAPDH SRP14 GAS2 EPT1 MYD88 CLDN3 RAB33B PIGBOS1 RBMS1 IVD NDUFS3 TBX22 LOC431499 NFKBIZ SP1 PHLDA2 MAP3K14 AHSA2 RNF14 TBK1 PYROXD1 RAB3IL1 ACADSB SRSF1 C14H17ORF103 5-Mar C10H15ORF59 ICMT ARL8BL MRPL20 LOC100502566 EPHA5 SPTSSA PLEKHB2 CBX4 BCL2L1 REL CTGF SLC25A22 MATR3 DNAJB12 SPHKAP GNAT1 CD74 ANKMY2 FZD1 PLA2G7 WFDC1 KDSR BMP4 ZNF302 LDHA NAP1L4 SLC39A13 FBXW11 NEURL1 CENPH CTNNA2 TNNI2 CD200R1L SLC18B1 ENO1 CSRP3 VIT VPS45 PPM1B SERPINA1 NFIX CREBL2 GJA5 TNFRSF19 RP11-196G11.1 MON1A CSRP1 SULT1B PTX3 NCSTN TAL1 MFAP3 FGFR2 RCHY1 CASP18 RPIA CAB39L SEPP1 DNASE1 CBX3 GET4 CASP2 PPP1R12B ADCK3 OAT ERN1 MRPL46 ORMDL2 SCLY PRNP VCP DR1 CD8B DMRTB1 AGMAT CDKN3 MORC3 POLD3 HAVCR1 AP2A2 IL6ST C28H19ORF10 ACSL1 FBXL16 TXN2 LOC396224 ANKRD40 SRP68 AL158801.1 ATP1A1 ENO2 ACVR2A ASB6 RNF34 EIF2S2 OSBPL2 STK11 CTSB TLR4 RPS19BP1 RORA CECR1 TAF2 NME3 HS2ST1 TMEM189 GAS8 CDKN2A PIP5K1A ZPBP2 GLP2R LOC415756 PPP2R2A NBL1 ZDHHC18 USP45 SMIM15 DNTT EHD3 SNX10 CKMT2 ACBD3 MST4 MTTP YAP1 ATF4 BLEC1 TMED3 PRRX2 PPP1CB LOC418424 GALK2 IGFBP2 APOA1 WNT8A FOXA2 PIAS2 SLIT1 UBE2G1 MED24 LOC419429 VTN TADA2A UBE2N NPHP1 PARN TSSC4 DDX10 APC2 HVCN1 BTN1A1 IL13RA2 ZBTB17 CIDEC INSIG2 PMS1 SLC35B1 KCTD9 GATA2 YIPF5 FHIT DNAJC7 CNEP1R1 LGI2 FABP6 STK32A NR2C1 MYH11 GYPC EXD2 SOX14 TUBGCP2 C15H12ORF49 ALKBH2 MERTK UBL7 PAK1 MYO1F PROM1 BORA ALDH4A1 CMKLR1 SEPSECS VPS50 PIP5K1B TCF21 HOXB4 ART1 COL9A1 ARHGEF3 EXOC8 CRYBB1 THADA MYOM2 FAM118B LOXL2 CYP1A4 A4GALT COL6A1 IRAK4 ACAN UBE2H KIAA0020 ZC3H14 GPR107 FLOT2 HDX RHOC ASB7 KIAA1143 FOLR1 RAB8A PRKAB2 MPZL2 PRELID1 RFXANK CACFD1 GNG2 CLDND1 FZR1 ZBTB2 NR2E3 SREBF1 NDUFB2 CAMK2A TREM-B1 PPP6R3 RPRD2 ANK3 TCIRG1 CCR8 NRGN G2E3 RP2 ANP32B NFYA PREPL KRT6A ALDH1A1 RUNDC3B EFHD1 ARHGAP19 SNCA ST6GALNAC1 NHLH2 RP5-1021I20.4 PARPBP DLD PSMD3 WWC1 MYF6 VWA9 RRP1B STAT5B JUN GTF3C5 CACNA1B GNB5 PTPRO OTUD6B CSNK1D EYA2 CREB1 ASB9 QRICH1 SFTPA1 SBNO1 TCEANC2 FIGF RGS19 CNN2 SETD3 SRPR HACD3 PCMTD1 SPINZ ZDHHC17 GTF2H4 MARCKSL1 ACLY NKX2-5 NOS2 PAFAH1B2 LOC772071 MTERF3 BRD2 SLN C1H21ORF59 MAVS MAGI3 RP11-295K3.1 OSTN RPL34 MRRF MAL2 SOCS2 IPPK DDA1 EYA4 ARL6IP5 LOC421975 MAPKAP1 SET AKIRIN2 HBG1 GALNT1 ELOVL6 CLEC3A RNF103 STX16 KDM5B GPX3 DUSP10 PLEKHO1 CDH1 RNASEH2B UBLCP1 STX17 HIPK3 NINJ1 RGS6 SLC38A4 CSDE1 RALBP1 TBL1XR1 E2F4 BPGM GHITM KRTAP10-4 NFKB2 TSPAN6 STX6 MAP2K3 RIPK2 SULT1B1 SLC16A8 MGAT4A HERC2 MMD APP ZC3H15 NDP MRPL38 STK10 CAPZB VPS33B NSUN4 CLPX BRCA1 SMIM20 GLUL RIPK1 SIX6 ADA MUM1 PPIB MFAP1 YWHAH PTPRZ1 GALNT6 WSB1 TIMP2 WDR45B CD72 DNAJB9 CEBPG ORAI2 BLOC1S2 RP11-290H9.2 TWSG1 SLC47A2 FAM195B UBE3B TM2D3 NAA35 KCND2 PDZK1IP1 MAOA VSX2 GHSR ARHGDIA SIRT5 CLSPN MAPRE2 SMC2 ARMC1 NPPC LYSMD2 DEPDC6 THRSP THPO LIG4 LRP5 CDV3 BRSK2 ADCK1 PLEKHJ1 HOPX CCDC28B SUV39H2 PIK3CB ARHGDIB MYL12A PDGFRA ATG4A FGF1 NELFA PLN SRSF10 E2F6 IRF7 PIP4K2A PTP4A1 YWHAE NR1H4 AATF GNPTG OST4 CXCL14 DAGLB FSTL1 POLDIP3 ERICH1 RAG2 RNF152 RAB14 OCX36 HMGCR GLRX5 IPO13 COL18A1 TBCD THYN1 GIGYF2 BRINP1 FBXW2 TRIB2 PPP1R8 PFKM CMTM3 ACE 9-Sep PSEN1 AKTIP FAT3 5-Sep SERINC2 TNS1 PTRH2 NECAP2 ACTB EIF4G2 BCL10 AKAP2 TNNT2 IL1RN JUP ABTB1 EIF1 XRCC3 NFE2L1 PDE6H KIAA1671 B4GALT1 AGTPBP1 SLC35G2 NUDT7 AP2M1 COX6C BZW1 FBXW5 TPST2 TBL1X DPP4 AGO1 8-Mar VCL ADAM10 CD200 ERP29 RRBP1 ARFGAP1 LMBRD2 CHAC2 CNDP2 CRY1 CNTRL CEBPB MTMR2 NKRF SNX24 ATP6V0A4 DCAF12 BASP1 USP6NL RBM12B RPL31 FZD6 ITGB2 RASGRP3 HOXB5 YWHAG BFSP1 TLCD1 STX2 RALGAPA1 C1D GHRH CETP BBS2 MDM1 PDDC1 TNFSF10 TMEM56 NCALD YTHDF1 ID3 ANXA11 DCT ANP32E MPP1 ZMYND19 RFNG RALGAPB RBP5 SENP8 PANK4 TMEM175 STARD4 OVAL CCK RMDN1 DDX6 TARDBP MAD2L2 HDAC7 CHADL MASP1 LYN TIMM8A EDF1 PLOD1 ABHD13 PAX7 CCL17 JAK1 FMOD COL9A2 HSD11B1L SF3A1 CLP1 YWHAQ UBAP1L TOMM6 POLE3 HNRNPH3 C20H20ORF24 CDPF1 SYNM FHOD1 |
| gga-miR-1573 | SLC16A1 NUCKS1 UQCRH DCN FOXD3 RPS6KA1 ZFYVE1 JUP FAM114A1 MYO1C CALM TRPV4 HMG20A TPT1 LOC100859148 HPGDS HSDL1 MEF2BNB HCCS HSD3B2 CYP24A1 ACVR1 CIDEC CDV3 RSL24D1 ZNF512B HOXB4 CENPF ACAT2 K123 EIF2D SLC2A2 CRYZ NDP ADARB1 SBNO1 CALM1 PCM1 RGS7 DUSP6 MCTS1 PCGF2 IRAK4 LPAR2 PDGFD WFDC1 EIF6 OAT NAGA BCMO1 SULT1B PDCD5 ELF1 RPL35A PYGB AARS2 ASNS PSMA5 ELOVL5 NFAT5 6-Sep RAD54L RFNG FSHR CD99 MYL12A PSMA3 KDSR MIB2 C14H17ORF103 CCK CRADD MINPP1 PWP1 HMGN2 MBD3 FBXL18 GRAP FAM126A NECAB3 8-Sep CHRDL1 PUF60 FBXL21 HDAC7 MRAS NCBP1 HN1 SLC47A2 TCF3 DCAF13 RRAGC SCFD1 GPR171 PDE4B KBTBD4 GLIPR1L IFT27 CELF1 FAM122A IGFBP1 CYP51A1 MEAF6 CUL2 TERF1 RPL9 KCTD7 PDHX KCNA4 FAM49A AANAT LIN52 TSC22D3 ERBB3 FZD4 PDZK1IP1 PRKAB2 PLIN2 RAG2 MYH11 TRIAP1 IMPDH2 MOB4 ASH2L LOC771066 SLX4IP PNRC2 KLF11 UBE2G2 LYSMD3 GPATCH11 PTP4A1 SELK RIPK2 MAOA AMIGO2 DCBLD2 PIP4K2A KCNH6 RPN2 KIF3B PTN GMPR KRT15 DEK CRY1 COL2A1 TRAFD1 SOCS6 CTD-2410N18.5 LDLRAD4 EPGN HSD11B1L NCAPH HMGCR HS2ST1 CTBP1 RARA SLC46A3 ALDH1A3 TSTA3 BTC TMIGD1 GMNN GPM6A LBH RNF220 PCGF5 ACTN4 RP11-403P17.5 F2RL1 BRSK2 SMIM3 CBX3 TTC7B ASB6 COL22A1 FEM1B SYNM DNM1L SRP14 CRABP1 ZNF384 ACAD11 SOX17 ST6GAL1 UTP15 SNCA 8-Mar KLHL20 CALB1 SEPSECS ST6GALNAC1 PQLC2 TAF11 ANKRD10 NELL2 RECQL5 VPS50 ARFGAP3 SRPR ACOX1 LYRM9 IFI27L2 USP15 CENPO IL8L2 SYNGR3 POT1 CLSPN TSPAN13 FZD10 DUT YWHAZ MRPL53 KBP CMKLR1 ABCC1 KANK1 RER1 AP2A2 WWP1 APC2 ANKRD40 CAV3 GCHFR VEGFA SCYL2 MAFA RGS6 HNRNPH3 IRF7 MID1 CHST3 MRPL37 TMC2 ELAVL4 KRTAP10-4 INO80 MAP2K5 GCLM VPS45 LOC395926 RFFL RLIM LOC426913 LYPLA2 MAPRE1 GHRH ZBTB2 ABHD17C SUCLG1 LOC422426 RGS4 ORMDL2 TNNT3 GORASP1 CD5 RQCD1 ANKDD1A CKM TTL AAR2 SRRD MAL2 FAXDC2 EGFL7 RCHY1 NKIRAS2 FK21 LOC420209 TBK1 ALAD C5H15ORF57 ZFAND5 ADAM9 FMO3 MFAP1 CLN5 UBE2V1 ATF4 TNFRSF1B DYNLT3 ASL1 ATG9A SATB1 ANXA7 DDOST DDX47 DEXI LOC395647 ABTB1 CCDC61 DNMT3B L3MBTL2 HDLBP ST3GAL5 MRPS14 SH3BP2 ENSA MMADHC CACNA1B G3BP1 PSMC6 TM4SF19 ACTR6 UCP3 CLDN1 GATAD2A SMARCE1 ALC ELAVL1 CASC4 SLC40A1 TMX4 NUP93 SCLY SOCS4 TESC GUCA1B GLI2 TYRO3 TMEM41B ACACA MAPRE2 NCOA1 TSPAN3 CERS1 SIX2 KPNA1 NHLH1 MBD4 KLHL18 CHMP1B GANC CBX1 LOC422090 NEUROG1 15-Sep SOX14 EFNB1 FZD3 ATP6V0D1 ARHGAP21 IMMT GEM P4HA2 RAX2 RAF1 STRA6 CREB1 PAPD7 MUM1 SMCO4 LBX3 HDAC3 ATP1A1 CLEC3B TXNL4B EIF1 FBXO34 RAB19 TEAD4 NQO1 KCNAB1 TRIB2 TMED8 MYO5A COX17 SLC47A1 FMOD BMP15 HJURP RFC2 PIK3CB EDC3 DCK GPR149 ID4 MDH1 CFL2 ARFIP1 MTHFD1 ARGLU1 CRYAA DR1 MET DPH1 DRAXIN TOP2B VIT TMEM138 YAP1 DNAJA2 S100A10 S100A9 FLI1 ALCAM CAMK2D NAA50 KCNN2 TREM-B1 RP2 CDK10 LOC693265 TRA2A APTX TCP11L2 MXD1 PRIMPOL CIR1 H3F3B SUPT3H BLCAP UGP2 TIMM13 GID8 NDUFS3 POLR2B ABHD17B RALBP1 IPO13 POLK ETS1 EEF2 ZDHHC8 PCK1 CDH11 CD40 LOC396531 SYNGR1 MGAT3 FAM192A TADA2A UMPS MRPS6 DIO3 TMED3 SH3GLB1 SERBP1 PTPRO LHFPL5 FAM45A LOC772071 G0S2 SLC19A1 TM4SF1 SLC26A5 CTPS2 ANGPTL2 CD200R1L ENOX2 STK11 TANK GUCA2B BSG CDK2AP1 SLC35C2 HESX1 TBX4 JAK2 TXNRD2 CNTRL TMEM229B IGFBP4 ARHGDIB ABCA1 KCNA2 ALKBH2 NELFA SEPP1 WDR24 PUS7 EPB41 USP28 PPP2CA BIRC2 MAP6 RPS12 DPM2 TRIM59 F10 COG5 PMS1 GLT1D1 COL6A2 ENTPD6 CNR1 LOC418667 LOC421975 TXNRD1 NFKB1 HBG2 CHAC1 CBL JMJD1C ID2 PDCD10 KIF3A SEPT2L SEC23B PTPRG ATOH8 SETD4 CDKN1A QSER1 CXCR7 ZFAND6 NEUROG2 PIK3CD FLII TAF2 STK11IP PHF5A IREB2 ATG4A RASSF2 ABI1 TAPBPL CTNNA3 COX6C CACUL1 STAT5B PHKB ARPC4 NOTCH2 TRIP13 USP6NL FAM213A GJA5 RASL10A DEAF1 IL6ST AMPH HAUS6 GYPC A4GALT WDR92 METTL16 EIF3J TAL1 MTHFD2 TFEB TMEM189 INSIG2 CNGA1 PDGFB RASGEF1C AvBD4 RASSF3 CACNA2D1 KNSTRN GNB1L SSTR4 CYP1A1 TXN2 TNFRSF13C CLIP1 PPM1M COL17A1 PSME4 LOC776816 CX3CL1 SCCPDH SYCE3 PREPL PRLR GAD1 PEX2 GABRA1 AvBD12 RMDN1 PABPC1 BFSP1 SERPINI1 RLBP1 STX6 KTN1 DHRS11 TNFRSF19 ARC FOLR1 APLF SLC34A2 GNRHR DIAPH1 TRPC4AP MRPL44 PELI1 NCSTN OPNP ZNF622 TCF15 TRABD CDKN2B IL13RA2 SYPL1 GPR89B HOXA9 ASIP CD82 PRKAR1A MFGE8 PTPRZ1 GBAS COL9A3 ST6GALNAC4 IGFBP2 METAP1 APELA MFAP3 CENPC TSSC1 CSNK1D TNFSF10 HBAA TARDBP SH3BP5 ACO1 ROR1 ACLY VAC14 MRPS25 TNNI2 FASN CINP GXYLT1 SNX27 UBN1 NDUFB1 RABGAP1L CHD2 HHATL LEF1 TBL1XR1 CEBPG ACTR5 C1ORF146 PAFAH1B1 RNF4 C3H6ORF154 LHX1 STXBP3 ELMO3 DAZAP1 TAGLN TAF7 DEPDC6 UBE2G1 CREBL2 NADK FAM102A ARMC1 BRD2 SULT1B1 GZMA GLP2R INSIG1 LSG1 CD1C C3AR1 NEUROD1 AKIRIN2 LOC419851 FBXW5 RTN4 GABRB3 TPH1 KCTD4 NGF POP4 TOX3 ORC1 CDC27 GAS8 PDGFC CD320 FK27 IKZF5 FAM18B1 AK1 MINA CKB RNH1 USPL1 MBP AC113404.1 SERHL2 TPM2 CHAC2 MBNL2 HNRNPA2B1 ATP6V0C OSBPL2 CYR61 RPS19BP1 NIP7 SLIT1 9-Sep STARD4 VDAC2 GJC2 NME3 NECAB1 CHERP RPIA PLN TXNDC5 ACVR2A MTFR1 SRSF1 WNT5A PHYHIPL TSPAN15 WSB1 MRPS18A PPP1R8 BCL11A AGO3 RP11-87C12.2 CISD1 FKBP8 TAF8 CDCA4 SSTR2 CD247 MPP1 MVB12B MORC3 ZNF767 TMEM121 RAD21 PIGY GTDC1 TWIST2 DLST RP11-195F19.29 GLUL CYP1A4 MRRF PIGBOS1 RBMX JMJD4 ANPEP AP3M1 GAS2L3 LOC420160 ENTPD1 SDF4 ERGIC2 SGCB LOC395095 CSRP2 SHISA2 HBE1 SFRP1 PRRX2 UBL7 MARCKSL1 GPM6B RAB9A ARHGAP8 BORA ARIH2 TBL1X NSUN4 TOMM6 RGP1 EIF2B2 LYSMD2 PPP4R2 RBL2 MYH1B RP11-514O12.4 DPH3 ATP6V1G1 HDAC9 RASGRP3 IL2RG AGRN VCP RORA NECAP2 DACH1 CTLA4 MBL2 SRC F13A1 FGFBP2 LOC100859586 ELK3 TOP2A CCNL2 NRTN TMEM104 CITED4 SLC7A6OS CCDC167 RINT1 WNT2B SMIM12 C1H2ORF49 RRBP1 TAF3 DYL1 C26H6ORF106 APOA1 NFASC WLS PPP2R2D GABPA GNG5 RNF152 PRRG1 ETV6 HDGFRP2 HVCN1 LIMD2 EIF3I DNAJC12 CIAPIN1 TJP2 KDELR2 SYT12 PLEKHF2 P2RY14 EXOC8 IP6K2 STT3B RHNO1 INCENP EXT2 WDR91 MDGA1 MGEA5 AARS NT5C3B GSKIP SIRT5 NUDT5 C1H11ORF54 FGA SOCS3 AKAP2 CBX4 ATOX1 KLHL24 CCKBR MST1 RB1 IFNGR1 SLN PSEN1 RAC2 ITPK1 TRAPPC2 PDK3 HIC1 NEURL1 RHCG NT5C2 CHRNA7 HNRPK GIGYF2 SPPL2A FAM210A MAP1LC3B RP11-49K24.9 MSN TRIM55 SDR42E2 RPRD1A BTBD10 SCYL3 CHRM3 TPM1 MAVS MBLAC2 RPL32 HAUS2 C7H2ORF69 PLIN1 BBS4 ARID3B NFKB2 YY1 SMIM15 PPP1R12B LIMK1 CHCHD4 SOSTDC1 TMEM88B SLIT2 CSNK2A2 TDRD7 FHIT CLPX COPB1 ARL8BL PPP3CB IL21R MPPED2 SCG3 SOUL SERTAD2 SLC39A13 RAB3GAP2 PTGS2 BLNK POLE3 LOC420419 C1H21ORF91 BEND7 RSFR RBM7 ANG GALK2 BBS2 DAPP1 CD72 STAT3 NR1I3 ATG5 SLC1A2 SASH3 RAN RAX USP12-like GHSR IGF2 MRPL50 VCL MSX1 C1D TMEM68 CARD11 COL8A1 FABP6 RNF13 ZNF706 CCNE2 QARS SLC25A15 SARS LOC769121 CTCF TBC1D1 PPP6R3 LOC428335 GSTZ1 TRAPPC11 RNF111 FBXO7 LUM ALG12 KIF18B PPP2CB CLSTN1 QRICH1 GLRX3 GALNT6 TAOK3 MEF2A HYAL6 DNAJB12 SRSF3 TMEM184C ACTB PYY OST4 EGFL6 AKTIP SERPINE2 CYB5R2 KCNJ8 C12ORF75 CCDC101 RAB8A FAM105A DENR ACSBG2 MGAT4A BGLAP MAPKAP1 CASP14 ATP6V1D ELP6 RRP1B LGALSL SLC17A5 AC025048.1 LIN9 NOP56 SLC30A4 CPA6 SOCS2 CLDN3 ZC3HAV1 CDH20 KCTD14 CLOCK SCNN1A SMIM7 PTBP1 GFOD2 RIPK1 SOX2 TARSL2 SELO LCLAT1 PTTG1 PLK1 NBL1 RPL31 EARS2 KCTD2 ST7L TMEM180 KDM5B DNA2 USP45 LOC769139 KRT75 PDE6H TIMM8A RBM12 ZDHHC18 COMMD4 EDNRB2 CANX CYP46A1 EPHB2 WBP2 KRT6A CTSB DDX6 IL6RA IGF1 ATAD5 GFRA2 TMEM5 CCL20 ORC3 MGAT4C C10H15ORF59 OC3 ADAM10 PLA2R1 CCDC58 COG4 SMYD2 PSMD1 YRK FGFR1OP2 ZDHHC5 CCDC28B DRD4 RALGPS2 HARS CNTN5 VDAC1 MYL1 WDR44 RP11-196G11.1 VNN1 ABHD13 RAP1GAP2 ABCF2 AKAP9 MATN1 BLOC1S2 LMX1A FAM222B LBFABP MRPL46 ARFGAP1 CRTAP BTK ID1 |
| gga-miR-1574-3p | OPTN SYK DYRK2 DIO1 CDKN1A BORCS7 JMJD6 WNT9A FAM18B1 LRRN1 COG4 CNTN1 OTUD6B NADK ADAM9 IL8L1 ALKBH3 GNG13 HARS HERC3 UNC5B COX6C IRAK2 DPM2 SLC41A2 RPL22 RAP1GAP2 SKIL MBP ZDHHC8 SMPDL3B SLC17A9 C18ORF42 HNRNPA2B1 TXN2 MYH1B SCNN1A GLUL ARMC1 CHRM3 SCIN LCMT1 ESF1 PSMD12 DHX38 ST3GAL3 BORA FUT9 HIC2 FLOT2 SERPINE2 NR1H3 PARP4 GCM1 PTPRZ1 SLC38A4 CBLN4 GJA5 HNRNPD PHB PPP2R2D CFLAR 5-Sep TRAPPC4 ALG12 CLINT1 P4HA1 LOC428335 BZW2 SNX12 KLHL2 KLF6 SAMM50 PDGFRA FPGT RABGAP1L RFFL STK32A SALL1 DLX6 ERGIC2 STAT4 RGS4 ADCK3 GFPT1 DYL1 NFYA RPS6KB1 ST6GALNAC2 TRA2B TSTA3 PRKAR1A KPNA4 KCTD9 HMGCR SGCB LOC769121 PDGFB C9ORF69 SPP2 CISD1 C1H21ORF33 AANAT PDLIM5 LUM ACTB PAFAH1B2 GPR39 MEPE MYO9B DDX6 MYL4 RPN2 IRF7 NFE2L2 GMPR RDH10 ZNF609 COL18A1 B3GAT1 FABP5 ZFYVE21 LOC770548 SLC16A8 HMGN3 BLOC1S5 SRRD EFTUD1 TOP1 CHERP CCNE1 GAS8 ASB9 UBLCP1 TM2D3 AIDA TREM-B1 KCNJ8 RP1-309K20.6 FAM177A1 MASP2 MMADHC ANXA5 LIG4 NCAN JAM2 NREP CPQ CANT1 KIAA0586 LSM14A C10H15ORF61 CTDSPL2 API5 LPAR4 STX2 ABHD13 MFAP3 MAPRE1 TMEM254 SZRD1 ARL6IP5 YPEL2 EPN2 RNF34 HVCN1 TMEM184C UBN2 ESD ECI1 C1QTNF2 MST4 HNF1A PARD3 CTBP1 BASP1 CDK10 LOC429492 CLIP1 DCK CHST6 TSPAN1 CRIM1 HERC2 ZC3H6 AvBD9 HPGDS NFIB RGS6 PDE3B TOB2 AvBD6 COPS7A INSIG1 ABI1 PSMD3 LOC770639 TANK VSNL1 ARL1 PPP1R21 NDUFB1 CNR1 HIBCH QDPR TTLL5 RGS17 AKR1B1L FAM122A IRAK4 SPATS2L TIMD4 BLOC1S2 CELF2 STMN3 AKTIP DAP TAF5 C8ORF22 ARFGAP1 IGHMBP2 ACVR1 SBNO1 RAB5A CLDN2 PTGR1 PLN RASSF2 FZD6 HOXB4 TRAF5 TLR2A COX4I1 ACADSB RRP7A SLC22A7 B3GNT2 NDEL1 NMU NUP93 NINJ1 BRE TEC LOC100859427 GAPDH PSPH ARHGAP8 PCMT1L TGM4 CDH11 9-Sep LOC426385 LOC420209 EIF6 TOLLIP TCF3 CNP POLDIP3 FGFR1OP2 N4BP3 BID CYB5A CBFA2T3 SLC16A1 NECAP2 NDUFC2 MCEE STK11IP GTF3C5 GLRX ALDH3A2 SDR16C5 CDH8 KIF5C LAMB2 PACSIN3 DCLRE1B VSX2 KIF4A TNFAIP8L1 UGT8 HPGD SHOC2 PDS5B ZFAND6 WBSCR22 RLIM KDR TRPC3 PPP4R2 HAVCR1 MSN TNC CCL19 IGFBP4 DRG2 COX16 PLS3 BSG A4GALT LOC100859586 ATG7 REXO2 GMFB MGME1 GOSR1 MYF6 TLR4 PSMC2 HDGFRP2 ATG4A NEIL2 TMED5 ASNS GABPA FAM65B LCP1 POFUT1 FASN RNF13 SPI1 RP11-195F19.29 PLEKHO1 C14ORF2 FOCAD PIAS2 LFNG NGEF NFIL3 GJC1 BMP5 VCAN THAP5 ZNF800 GARNL3 AKT1 TNS3 LOC418424 CD1C YTHDF3 CDH6 ABLIM1 KBP VPS35 TBC1D1 C4BPA NEMP2 GNAT2 TDRD7 NARS HOOK1 RHCG MAP3K14 ORC3 GAPVD1 CIRBP RAG2 EIF4A2 PAK1 C1D SELO GLCCI1 AGO4 NECAB3 CPSF2 ANKRD27 ALCAM LCLAT1 METTL2A PBX1 RREB1 BBS4 AMD1 RHOJ CDK6 NUP107 OLFM1 C1H21ORF91 APBB1IP BAK1 ENSA PRKAB2 PTGS2 PTP4A1 CECR1 NPL GAS2L3 POPDC3 TSPAN13 BTN1A1 RBBP4 CTCF GNG5 ENS-1 ADCY5 SNX16 RSL24D1 RPL12 SLC25A26 WIPF1 BFSP1 MSGN1 LGALS3 RNF7 SMIM4 ARNTL DUSP10 RELL1 SNRPE PSMD1 ELOVL6 FBLN5 RBM7 LMO4 GATM LPAR6 PCGF2 LOC420160 CAT CTD-2116N17.1 MPZL2 LDHB STK24 UBL3 MINA FTL PHAX AGA BCS1L EFNB1 TSKU CRYBB1 PSME4 CNOT1 SF3A1 VMO1 SRPR RUNX1 CCK TBL1X ARF1 GALNT6 ASNSD1 ABHD17B MBD3 SCOC MYH9 FAM214A PEX5 JUN NME2 USP12-like NEURL1 ARFIP1 F-KER RTKN2 SDF4 OVALX MCM3 CD200 BPGM CPZ LOC100859249 CACFD1 GLRX5 C5H14ORF166 C4H4ORF29 RAP1GDS1 NCK2 TMEM26 TMEM121 FAM192A SUV39H2 PMP2 CASP18 DYNC1LI2 ETS2 SGPL1 RHOB YBX3 CSRP2 RPL31 FBXW2 MAP1LC3B SDK1 ENTPD1 NCSTN CSTB RHAG KPNA1 FAR1 GPR149 ORMDL2 SLMO2 HSPA4L SYNM SALL4 CYP2D6 KRT6A GNGT2 ECHDC1 LZIC CYB5R2 PNRC2 SLX4IP CACNA1B OAZ2 NELFA LIN9 BCL2 BEAN1 RIC8A PDE6C LEPROT TBX5 MTF1 DOHH DNAJB6 MYL10 YIPF3 NKX3-2 C7H2ORF69 GAL LSM6 PRKAB1 PPP1R9B WDSUB1 ZFYVE19 NAT10 RAD51 SLC16A3 C1ORF146 DIO2 PPHLN1 AHCTF1 MYL12A SAR1B IRF1 SNX2 MRPL38 VTN IMMP1L ACP1 UBE3B TEAD4 TCF7L2 ZBTB2 CD276 HMGA1 PIP4K2A BRAP ELF1 TBCK ANKH SLC18B1 TASP1 CCDC6 GOLGA7 UFSP2 RBM48 ZCCHC17 GATA4 TP53INP1 AvBD4 DKC1 GPR107 SYTL1 MAD2L2 MATR3 LHFPL5 HSPH1 STAT5B EIF5B C20H20ORF24 SETD6 LIN52 AAR2 LIN28A SLC35B1 C8H1ORF52 SLC19A1 PLEKHB1 NHLH1 RNF185 ARRDC1 FSHB TECTA MPP5 TNIP2 ADIRF RLBP1 EDC3 MMD GLP2R ETFDH TBC1D22A MRPL20 LHX9 RAB11A FZD3 LYSMD2 ZFHX3 ALDH1A2 GABRB3 KPNA2 SNCG SPC25 HES5 DLD S100A10 DAGLB PBK MEAF6 B4GALT2 OVAL MLNR SLC6A9 PLAC8L1 TMEM173 GYPC PLEKHF2 VAV2 CTNNA3 PLK1 RAB3IL1 SMPX CETN1 AP4B1 SLC12A7 UBE2G1 RBP RBM12 INTS7 DRAXIN TMEM68 SRP68 FZD4 NEUROD4 CDH20 RASGRP3 CCL17 PALM CCNL2 SLBP PIGA SOX18 PIP5K1A LOC100859616 TOMM6 DCT PRTG PSMD14 LOC776376 SMIM18 SLU7 NUDC EPB41 SYCE3 SMIM8 ADIPOR2 EIF2S1 ZP2 RARRES2 CCDC127 8-Mar GREM1 EDF1 TMEM56 CD82 RPS14 SSTR2 PPM1M TMOD3 IRF4 THRB CRADD PARP1 ZSWIM7 RFTN1 ST6GALNAC1 VRK1 CCNC CNOT2 ARHGAP21 NUDT7 TMEM167A WRN SGTA IBA57 HPRT1 PAIP2 CHAF1A F13A1 EXOSC2 PCM1 CR1L CDC42SE2 MMP9 LSM8 MRPL45 RAB24 TAF11 KCNJ2 CHORDC1 HDAC4 FGFBP2 METAP2 CSRP3 WNT11 MFN1 RASGEF1C NPPC BZW1 ATG9A LAMA1 PQLC2 PACSIN2 LOC424740 GTF2E2 CDCA4 WBP2 GINM1 RBM12B HMGCS1 MYH10 CREG1 RP3-461F17.3 ZDHHC5 CL2 RGS20 CPA6 TNFRSF19 GPR137B NUCKS1 LOC396531 CFAP20 CFC1B FZD1 GNG2 HABP4 ACACA SLC6A4 PTN ANK3 BRINP1 AKR1A1 C13H5ORF15 RNASEL CASP6 ISLR2 P2RX1 GFOD2 CDH5 PAQR8 ATP12A NAPB RABL3 MFGE8 CDK2AP1 LOC693265 PTPRA VPS50 BTC TMCO3 STRAP ANKRD16 STAT1 NR5A1 PSEN2 PDCD6IP P4HA2 TMPO ZBTB26 TXNL4B IDS FOLR1 SOX10 PEX2 KIAA1191 TRMU BMP4 FAM98A ZYX STARD4 IFNGR2 NKX2-6 MBL2 DOLK IGF2 NDUFA4 SLC15A1 PTPRG LDLRAD4 ASAH1 SRC LOC422249 UBB BTBD9 MKRN2 CPNE1 ENPP4 MOB4 ELMOD2 ACAD8 SNX10 TYRO3 TAPT1 TMEM129 UBE2I C10H15ORF59 AvBD2 VNN1 HNRNPDL NDE1 FLT4 PHF5A RAD21 C5H15ORF57 PPDPF MTMR8 CNGA3 TACR1 ROM1 ACTN4 15-Sep PRPF3 LOC769139 TRIM39.2 ITGB5 CITED4 CARS RTN1 CLN8 C14H17ORF103 TRH CHM RP11-403P17.5 STX7 SPG21 LUZP2 ADD1 SUGP1 NRG4 NDUFV3 ANXA11 EFHD1 CASP14 GSKIP SUPT3H STX6 ADORA3 KCND2 TP53I11 HIPK3 KDM5B MTIF2 NFKB2 FAM222B RCHY1 RRAD HHATL EPCAM UBN1 MORC3 STK11 C1GALT1 CNOT10 ANXA13 DPP4 HSD3B2 RAB9A EEF2 PDE4B PSEN1 MYLK2 SET FZD10 CNTRL TSPAN8 MIB2 PIGK CCR8 ZP1 TMEM41B SOCS3 TFAP2A CDC25A CTNNA2 TCOF1 NPPB NFAT5 OSTM1 EDNRB MED9 METAP1 RHOG TAF8 CRTAP H3F3B DNAJC6 IMPDH2 DUSP4 PPIB PDDC1 GEMIN2 FGFR2 EXOC6 ARIH1 PDGFC LOC420419 S100Z CSPG5 RAB35 TACC3 CBX3 LOC100502566 KTN1 RALGPS2 AMY1A IFT27 MSX1 ACTC1 FGF10 KLHL13 RAD52 NDUFS1 UBE3C KLHL20 TYRP1 LOC427470 HCCS MAL MCL1 YPEL5 IFITM5 SMARCE1 CD320 RGS9BP RGS19 LYSMD3 SGTB TAOK3 TUBGCP2 BCL2L1 CTSA DYX1C1 FLNB COL4A2 NFIX TDP2 BCMO1 CDV3 GNB1L PHF20L1 FBXL16 SLC25A6 CYTH1 CHD1 ARL6 DNAL4 STRA6 HIST1H103 USP6NL DNAJB9 QRICH1 AC113404.1 VPS4B RHBG RAD51D PFN2 MBNL3 BRD8 PKIG ADAM10 ANKRD26 VAMP7 PGK1 PELO ERI1 TOP3A CANX WDR5 CPT1A P2RX5 NOC2L SH3KBP1 PLA2R1 DHRS3 CIAPIN1 USP15 CKMT2 LOC100859148 FHL5 HIC1 DYNLRB2 DDA1 TMEM184B KCNIP2 TANGO2 |
| gga-miR-1648-5p | ARHGAP29 MTIF3 HNF4beta KATNB1 PCK1 FOXO1 NTPCR ID2 UBE2V2 SLC17A9 OPTC ZBTB34 PDLIM4 CNR1 DEXI NRP1 AC113404.1 ZBTB2 EPS15 AMPH TEC RPS27L SOX10 PLAC8L1 VPS29 SERINC1 DYM RDH10 P2RX5 HMGB1 EPHA5 MGAT4C SERINC3 WBP2 SMPDL3B SRSF11 PSME4 TIMD4 TF BRPF1 INVS HDAC7 VCPIP1 BAP1 MATR3 FAM98A FURIN CDC20 YWHAE UCHL5 C11H16ORF70 ETV6 FZD4 KDM5B MPHOSPH8 ITGB1BP3 HAT1 FOLR1 CHP1 CHTF18 C8H1ORF27 APTX NOTCH2 RP11-20I23.1 KTN1 TTLL12 MICAL1 UBA2 C1H21ORF91 SRC MTIF2 GPR89B NR5A2 TAL1 CLN5 GPRIN2 LOC431325 COL5A1 OSTN BHLHE23 C20H20ORF24 ARHGDIB LY75 GPX2 EIF5A2 PLEKHM1 PIK3R5 LCMT1 GNGT2 HDAC2 PRKCD SGCB SGMS2 FZD2 FGF14 TMEM141 NMRAL1 ENTPD1 YIPF5 BIK CSRP3 AvBD1 LOC395991 ANXA11 ELN HCLS1 AvBD10 CELF1 MEF2D S100A10 C14H17ORF103 CYTH1 ABHD13 NDUFB2 SKIL DAAM1 IRF1 BMP10 RPL9 CPA1 TMOD4 METTL2A PSMD3 TPI1 IRX4 SEC22B SRSF1 KIAA1671 IFRD1 CKS1B GUCA1A DCBLD2 ENSA BNIP3L HNRPK CNTN2 LOC420419 VPS37C SH3GLB2 BCL11A CDH6 ZC3H6 GPR39 CCNE1 HIPK3 NFE2L1 TPCN3 MYL3 HSP90B1 SMIM12 PMS1 SYT12 KIF3B KDR BLCAP STAT1 CNBP ST6GALNAC4 COPG1 SLC40A1 SHOC2 HSPH1 SNAP23 CD36 TNFSF10 CLDND1 GNAI1 WBP4 PRKD1 SLC25A15 PAFAH1B2 TOMM6 SSX2IP PDS5B YME1L1 NDP TMEM229B PTPRZ1 DCTN6 SLC6A2 RFFL PALM SRSF5 ZFP92 AKAP17A CPSF2 TCF15 LRRTM3 ARL6 DTNBP1 SOCS3 UBE2J1 IFNGR2 PSMA5 KIAA0020 MAP3K14 CCNL2 PRLR SOUL STK17B DUSP10 EIF2S1 ATF7IP SYNJ2BP UCK2 APOC3 RPL34 ACBD5 RTKN2 DYNC1LI2 LOC776816 SERPINB10 ABHD17B FAM133B RFNG HYKK MTMR2 FADS2 GUCA2B PPP2R2D RBBP7 PYGB HNRNPD SNRPE BCS1L TMEM184B ABHD17C CDC42 DDX4 PDP1 MED9 RECQL SNX24 MYD88 FXYD6 SNAP91 COPB1 WRN LOC426914 MCEE DEK SREBF1 UGP2 CHMP4B MYH10 MET SIX1 VIT LOC429492 DIO1 CHRM3 SDR16C5 GMNN PHKG1 LUM DSTN KLHL6 ABCC1 SALL1 MRPL45 OPN5L1 LHFPL5 DTWD2 LEPROT COL9A1 SRRD APP PGRMC1 ETFDH MAP2K5 ORAOV1 SERINC2 KPNA1 LOC100858447 NUP93 PRKAA1 PAN2 NAE1 PDPK1 ASAH1 ELK3 GSTK1 TRIP13 HACE1 ATM ALKBH2 ETV1 KIAA1191 PREP EIF1AY ANKRD52 HIBCH FZD5 NFASC TRPM7 KCNT1 ATG9A ACBD6 LEF1 ASNSD1 RAP2C CBFB CSRP1 POLDIP3 OSTC PSME3 NEDD1 ANK3 PPDPF HAVCR1 CSNK1E ACOT9 RPS14 DUSP6 MCTS1 DLX6 RRM1 FKBP4 TPM1 AC005943.2 WDR3 GLUL ORMDL2 ID3 TP53INP1 FLOT2 SLC46A3 GNPTG TSEN2 PHACTR1 URAH IREB2 BRAP BIVM SLC7A6OS KLHL24 FAM20B BCL6 PDZD11 CXCR7 RNF185 ECE1 TIPIN BLOC1S2 SERPINA4 NR1H4 CIR1 MAP7 RPS6KA1 PCMT1L RREB1 TEAD4 GID8 NFRKB XCL1 ERI1 LIMK2 NEIL2 NFIL3 FSHR NUCKS1 RCSD1 CRADD MCCC1 DGKZ FUNDC1 CHRNA6 RBM25 WDR82 IFNGR1 DLD NF2 CLU HDGFRP2 GORASP1 TMEM129 ANXA7 PUM1 UBAC2 PHF21A GEM BCL2L1 GNG5 SMIM18 TMEM184C GNB5 CDH20 LOC100858504 OSTM1 GBX2 KATNBL1 PCASP2 RPL29 RAD21 VCP ARPC1B SOX1 MYST2 IHH RUNDC3B IRF7 EDA2R SLMO2 GARNL3 ARHGAP26 ANAPC10 DDB1 SLIT2 PEBP1 DTYMK NEUROG1 SPINZ CDR2 WIPF1 ZFAND5 TAX1BP1 EIF2D RBM38 LOC100858439 YARS ATF4 POFUT1 ZDHHC17 PAX7 WDSUB1 DMTF1 PROM1 FAM136A SLC2A2 SERBP1 VPS33B PELI1 EDNRB2 ARHGEF3 CEPT1 ELOVL1 TSPAN6 BAZ2B MYH9 TUBAL3 TGM4 AP2M1 NDUFC2 NOS2 GJA4 SLX4IP NFE2L2 LOC769121 VPS16 CEBPB PLEKHJ1 SPTLC2 C15H12ORF65 LUC7L2 PLIN2 SEPT2L MGME1 PHKB PDCD5 ANAPC13 MYL12A GIGYF2 SENP8 CD38 TGFBR3 PREPL TBC1D1 FBLN1 RHOBTB1 LDLRAD4 TIMP3 FAM46C PLLP BICC1 YIPF1 HDAC4 EEF2 PRELID1 DNAL1 STAT5B SRSF2 RP11-5A19.5 CACNB4 TMEM121 ST6GALNAC1 DOHH TNIP2 TXNL4B PKM EGFL6 AP4B1 LDB3 STRAP TGFB3 NUSAP1 PACSIN2 ENPP2 AAMDC LYRM9 SPP2 KIF20A TWIST3 GFOD2 RALBP1 NCSTN TULP1 FAT3 IDH3A RAB8A AGMAT DAP F2RL1 MYBPH UBQLN4 7-Sep IER3IP1 KRT7 CNOT1 TFAP2A SERPINH1 MYF6 PNPLA6 GLIPR1L DNAL4 KCNIP2 UFSP2 RP11-87C12.2 LETM1 MYOM2 HMGA1 CLIP1 GLI2 FEN1 ESF1 C1S CAB39L PLK1 DAD1 E2F5 MPST IL2RA RPL31 STK24 ETV5 DNAJC17 CAPN1 PRNP HPCAL1 FMOD RBMS1 ANGPTL2 ARHGAP19 TIMM8A CREB1 PTPN9 C7H2ORF76 C5H15ORF57 PLEKHB1 MESDC2 FAM65B NDUFA8 GALK2 SLC19A1 H1F0 GPR149 MDH1 CCDC127 LPAR2 EARS2 CD247 CBL POR VAC14 ANO5 WNT5A TP53I11 CTNNA2 CACNG3 COL22A1 ARID4A AP2A2 RAD17 ARL8A ARFGAP3 PDLIM5 REL JMJD1C BUB1 LOC396009 NDE1 TMEM5 GAL3ST2 RGS6 ARFIP1 CMTM3 CBFA2T3 S100B SF3A1 EXOSC2 RPS4X CKM GEMIN2 MYBPC3 YPEL5 GJC2 TMEM55A SIX6 CCM2 SMAP2 DPYS TEF TAF1B MAP2K2 EMP1 PTX3 STK10 GTF3C5 MGLL POT1 AREG UBL3 PSMC3 POU2AF1 TNFRSF1A FAM45A GPR126 SPTSSA PIP5K1B ESD GPR174 MCAM LMO4 WFDC1 SCN9A PRLH XPO1 KPNA4 ATP2B1 PRPF19 UGT8 CTSS THPO RIPK1 BZFP1 SSR2 ASNS CFL2 UBE4A CREBL2 ARHGAP40 FZD1 LGI2 HOXB4 EPT1 POU1F1 SPATS2L TAF7 CNOT10 SRPR PAX9 RGS17 MPP1 MPP5 LOC428335 PIAS2 CD72 LFNG THOC5 ING3 NME1 MYLIP MEAF6 NUDC ANKRD40 CEP19 PSEN2 CHUK CUX1 MAPT SMARCE1 LOC770639 GLT1D1 TMCO3 ATP6V0E2 MEMO1 SRL LOC100859039 PLEKHO1 TMEM123 MLPH FYTTD1 CALML4 DDX42 EMB CHD2 FBXO7 LZIC CHPT1 GTDC1 TMED2 VNN1 AARS SUB1 NEUROD4 TM4SF1a 8-Mar THYN1 SMIM5 SIRT5 RORA ALCAM TERF1 ID1 RRP1B FGF19 OvoDA1-2 RNF34 NFYA FGFRL1 BKJ OPN1LW REEP3 C28H19ORF10 NCAN FOXM1 ITGB2 COPS5 NFIC ZNF767 MCM3 EIF2B2 YIPF3 COX6C DNAJB14 IGF1R ZNF800 SP5 CLIC2 NEIL1 NRN1 CETN1 TMEM120B CTSO FDFT1 RHOA SSRP1 ACADSB MLNR FASN HIC1 DCUN1D1 TMED10 TWIST2 SRGAP1 BRCA2 C5H15ORF41 GTF2A1 MST4 NRBP1 AMPD3 RPIA LOC420849 FABP6 SNX16 SLC9A4 CNOT8 CHAC1 RP11-195F19.29 CLDN2 LOC417800 DCAF7 ZMYND19 HZGJ LOC769139 ERICH1 CACNA1S EIF3J PRDX1 UBE3B FZD3 SDR42E2 VTN E2F4 GALNT1 TUBGCP2 RAB11A DCT PIP5K1A TRPV4 ABCC6 TNS3 MAFB RLIM PDHX PTRF GSTO1 LPAR4 TLR4 CCPG1 RPL37 RABGAP1L FEM1B NR2E1 PPP1R8 CCDC50 PBX3 ICOSLG WASH1 PKD2 CLN8 B3GNT2 ANKRD10 P2RY6 ATP1B1 FBXO32 CKB TMEM57 COL6A2 CDKN1A TFEB TRIAP1 SLC25A4 GREM1 SPINK5 NDUFA4 ADIPOQ NKIRAS2 LCLAT1 PMPCB FAM21A FTH1 P2RY14 GNG2 RALGAPA1 TNNI3K EGR1 ACACA CACFD1 SYK GPATCH2 RB1 GLOD4 C3H6ORF154 OCM RQCD1 YRK DNAJB12 PUS7 SLC35E3 DHRS11 GJC1 RNF141 ENO1 MTPN KCND2 DNAJC2 RASSF2 MSGN1 AP1S3 SLC7A9 GNE ENTPD8 AIDA CRYBB1 ST6GALNAC2 TBL1X UBXN2B TPH2 NPC2 ITGB1 FBXO9 TM2D3 AGRN SCIN MRPS7 CNGA3 RP4-613B23.5 TMEM180 SERHL2 CLEC3A CRH EIF4A2 DLX5 GET4 HES5 PUSL1 C11H19ORF12 RAD52 FANCL PPIB INCENP SH3BP5 SLC25A36 TOLLIP NEUROD1 KLHL20 NADK ANXA2 PRMT3 ZNF410 BMP15 PODXL TBCK SCG5 WNT11 TNFRSF19 DR1 P2RY1 OAZ2 ZCCHC6 FLI1 CTNNA3 ACOX1 PPM1M LOC419112 FOXC2 NECAB1 SOCS4 ENOX2 SLC16A3 FAM122A KCTD14 RHBG LOC396479 RSL24D1 GINS1 NR5A1 DYL1 BAG5 LOC396380 NRG1 PDGFB ADORA3 IL6ST TUBB EMC1 PSMD1 HBE1 CCDC28B NEK7 HAUS6 RBMX PRKRIP1 GNAT2 MYH1B TNFRSF18 TOX3 TPRA1 UVRAG MRPS16 FAM18B1 UBE2H PRKCI SERPINA1 RIPK2 CLSTN1 CDH17 IPO13 APELA TRIM55 RBM24 USP45 WWC1 IVD KCNAB1 MRPS26 UBE2V1 METAP1 LDHB DNAJB9 CRIP1 C3H6ORF203 AZIN2 LMBR1 NT5C3B DPYSL4 GLCCI1 BTBD10 LGALSL XRN2 C11ORF31 ORC5 DPH1 FZD6 INO80 NRGN PCNP NECAP2 TCP11L2 ACTG2 BIRC2 MPZL2 NFIX AP3S1 WDR45B TECTA BRD2 IGF2R LOC693265 |
| gga-miR-1771 | PSME3 CST3 IL8L2 MAGI3 BRSK2 RFFL SPPL2A RALGPS2 KCTD7 LYPLA2 RAB32 QRICH1 CHST10 DFNA5 PCM1 LOC419851 ALDH3A2 CYP11A1 IRF5 ALDH1A1 SDK1 RAD52 SERPINI1 FBXL21 DRG2 RHOA RNF14 AvBD1 NAIF1 RB1 C15H12ORF49 CCDC58 ELOVL1 MET IRAK4 PDCD6IP RP11-49K24.9 GFPT1 SLC38A4 CTNNA2 ST3GAL3 SERTAD2 ACTR5 SZT2 C10H15ORF59 TXNDC5 SGPL1 IL6ST ST3GAL1 OSTN CLDN1 CSDE1 FAM177A1 CHP1 GALNT6 DKC1 PPME1 GATA4 UTS2B FBXO8 MPST HAS2 MAFA PRLR PHLDA2 PARPBP SCIN ACP2 K123 STRBP NME5 ATP13A4 GANC CASP14 HIBCH ST6GALNAC2 FYB MYL9 C3H6ORF203 LCLAT1 APCDD1 ATP6V0E2 CLEC3A RAP1GAP2 NFIX ERLIN1 CYP51A1 RABGAP1L RP5-1021I20.4 NSUN4 SSRP1 PKIG LYG2 TGFBR3 FBXW5 EMP1 TCF15 WDR44 GNB5 AHR EVL DHRS11 FZD7 PLEKHO1 KPNA4 TNNT3 SMIM12 LUZP2 CANX PDGFRA SLC9A4 PMAIP1 CD3E RP3-461F17.3 TARSL2 TGFBR1 PIGK SOUL NEK6 ANGPT2 G2E3 SEPT2L ZBTB17 HIC1 FSTL1 ACBD6 DPH1 CLDND1 HOXB4 SUGP1 ZNF302 FGFR1OP2 GET4 MBL2 PLA2R1 CD82 S100B SSTR2 ST6GALNAC4 CHRM3 NR1H3 CRISPLD1 FAM118B CNTN5 NEDD1 ABCF2 CNBP SET HOXB5 NOTCH1 CLDN2 VPS41 CASP3 RBBP7 PIK3CA ACLY SMIM8 DCUN1D1 TBC1D22A LOC396531 LOC420419 PROKR2 PUF60 LRRC57 C26H6ORF106 COX17 BIRC2 SIRT3 CNP NPAS2 CREM FAM20B RPL31 CHRDL1 E2F4 MCL1 NUDT5 MGEA5 UGGT2 SLC47A1 LPGAT1 FGFR2 RHOG TOMM6 FIBIN PELI1 COPB1 SLMO2 FHIT ANXA11 CCR5 CHIC1 MTMR9 MBD3 NRP1 KRTAP10-4 CCNE1 RNF20 P4HA1 JAK1 JAM2 FXYD6 CPT1A HNRPK ALG6 LIN52 LOC427001 HIPK3 CRADD KDM5B STAT4 VSX2 ANKRD16 CSNK1E CITED4 ALKBH3 CTSB EPYC MAOA MSRB3 ELAVL1 DEK PAIP2 LSG1 KBP CSNK2A2 POT1 KLHL20 UBXN2B ST6GALNAC1 HDAC7 DEXI MAP2K2 CCDC101 ACTG1 PLEKHF2 AR WBP2 CLTB ACOX1 SGK3 LPP PEX2 PRKRIP1 ZCCHC6 MAT1A FZD1 ELMOD2 CARD11 PTH TWSG1 DYX1C1 GFOD2 WIPF1 SLC9A3R1 RHNO1 RBM12 SLIT1 GPR149 C20H20ORF24 MYO5A CCDC93 LOC100859427 TMPO HNMT SYK FGF1 DAZAP1 MAP2K1 GEM AHSA2 AKAP2 RTN4 HNRNPR SLC35E3 SLC46A3 ENO1 NUDC LYSMD3 NAV3 ZBTB34 SMIM5 HAUS2 WDSUB1 FOXA2 CYP46A1 DYNLL2 ASNSD1 AC025048.1 PTP4A1 RP11-87C12.2 COQ5 ANXA6 LOC395991 FBXL12 KDR FRZB ZP2 AC113404.1 SNRNP200 HARS LOC418667 PAX6 NKRF FAM175B GLT1D1 TEX264 S100A10 TMOD3 ST3GAL2 UBE2R2 TREM-B1 ARFGAP3 STK24 FAT3 DDX4 RBMS1 UBE2V1 HERC3 GALNT1 CD44 DYNC1LI2 WIPI2 USP45 IFT81 FLOT2 DCT TIMM9 CDC27 CNR1 KLHL7 SUV39H2 MGME1 MYH1B TMEM106B PPARD TAL1 SLC16A3 ITGB1 IHH CDV3 SASS6 DLST EFNA5 ENS-1 RREB1 NOV PDP1 RPIA DPF3 MYD88 ALDH1A3 RAB9A CIRBP KIAA1191 BET1L CAB39L FZD5 IL16 RAB11A WRB ACOT9 FAM60A STX17 NT5C2 POLE3 USMG5 LOC776816 FABP6 TMEM104 TMEM230 BASP1 VIT SNX10 SLC34A2 GTF2H4 PPP2R4 NEMP2 MLNR ACACA CD1C MXD1 ANKRD27 SNN PUM1 FOLR1 RECQL5 LIN9 TNS3 BID VAMP7 STAT1 MTMR3 QDPR STK10 CDC25A ROM1 YWHAG PBX3 CRYAA GPR89B VDR CKMT2 THRSP LSM3 DYM RAF1 GMFB CPA6 CUL2 BORCS7 CHIR-IG1-5 BAK1 COPG1 MGAT3 ASF1A EDN2 HDAC9 ERNI IRF1 LOC415756 SGTB EPHB6 MAP7 SPP2 FEN1 YIPF3 PSEN2 PSPH TPST2 CLK2 FLI1 SDF4 PSEN1 CD72 ESR2 INCENP SYTL1 SMCO4 ATP6V0E1 CYP2D6 PTPN11 SLC26A6 UBE2V2 SLC39A13 PPP2R5C SIGIRR SZRD1 LOC421975 PLEKHM1 TSPAN12 LIMK1 LIMD2 MYBPH ATF7IP IGFBP1 TMEM184C JMJD6 CELF2 AKIRIN2 COG4 BF2 ZFYVE1 ID2 TEAD4 TMEM41B GLRX CFLAR PLEKHJ1 RNF25 ST3GAL5 PTPRA RASL10A ATP6V1D ABHD17B ATP2A3 AMY1A TTPAL DYDC1 RGS19 NR2E3 RRP1B FDFT1 FAHD1 MAPK6 ARF1 KLHL24 CTBP1 ARHGDIA NEURL1 RAB35 SMIM18 FAM49A T P4HA2 SMIM3 GREM1 TLE4 FANCC OSBPL2 MAVS N4BP3 GFRA1 PEX11G UQCC SETD3 CREG1 CTSA HAVCR1 GLI2 TDRD7 BG8 CTDSPL MAP1LC3B CMTM7 TRIM27.1 MFAP3 CD1B LHFPL5 GATAD2A DRAXIN RAB8A METTL16 KLHL15 BCL6 UGT8 CDK6 FMO3 KIF5C SERPINE2 COLEC12 GTF3C5 PNAT10 ARIH1 SNAP23 IRX4 AKT1 PDE6H PIGY EPB41 EIF6 SP1 RALGAPB COL8A1 NCLN ERH LOC422926 TAPBPL SYNM PTN CHERP TBK1 SALL4 SGK196 PMS1 POPDC2 FBXO7 SLC25A22 SYNJ2BP KPNA2 STRA6 BANP UBN1 C1GALT1 SRC STXBP3 VPS45 RP11-834C11.12 MXD4 TSC22D3 HINT1 MAPRE2 TMEM131 KIAA1671 TMEM175 IL10RA INHBB PLEKHA2 IRF7 TTL SRP68 ORAI2 ELMO3 LIFR MRPL34 CMC2 PRORSD1 PAQR8 WBP4 TOB2 MRPS33 RCHY1 BTF3L4 DCN EXOC6 VDAC2 OTUD6B ABCA1 NR1H4 TRPV4 USP6NL FUNDC1 SPINZ QSER1 VGLL2 CTNNB1 MTIF2 BTN1A1 NR2C1 COL22A1 WDR89 NFKBIZ IRF4 SOCS5 PGA DUSP10 ANXA7 PTBP1 SALL1 CHM MYLK2 TLN1 PAFAH1B1 SELO VNN1 TBL3 RDX KLHL18 DAPP1 MYO1C C3H6ORF154 TEAD1 SKP2 MEF2BNB VMO1 SNRPE AZIN1 CSPG5 CMKLR1 PLEKHB1 CLDN3 SMIM19 SPPL2B B3GAT1 UBAC1 C4H4ORF29 FHL5 C1H12ORF73 MYLIP TACC3 SLC1A2 FYTTD1 ADAM9 HDAC4 SERPINA1 GABRQ CCDC61 TNFRSF19 MESDC2 TNFAIP6 LOC424740 GJC2 PRR5 APPBP2 MAPK9 FOXO1 GDPD5 PRC1 C11H16ORF70 PIGR H1F0 SRGAP1 TWIST1 PCMT1 WDR5 MAP3K14 GATAD1 SEPSECS THY1 TMX4 JUP VPS53 PHTF2 SEC31A MGAT4A TRIAP1 RP11-77K12.7 ELP6 TBL1X SLC40A1 C11H19ORF12 CANT1 ORC3 LOC770548 CTD-2410N18.5 CPSF6 RP4-613B23.5 CAPZA1 SLC16A1 OPN5L1 MCEE EGFL7 GPATCH2 TSPAN6 CTDSPL2 PLIN2 SAMM50 GLOD4 BRINP1 CRYAB NDUFB2 IGF2 FAR1 LYRM9 GORASP1 COX16 DCAF7 GLUL NDEL1 MCM3 BTBD10 ACTC1 TRIB2 DEPDC6 ATM ALDOC PIK3R5 SEMA7A TSPAN15 GABPA ICMT CDH6 NAP1L4 SENP8 MAP6 DOLK PKM PIAS2 GCLM ENTPD2 SNX16 SOCS3 TERF1 ARL6IP5 TANGO2 NRN1 NBL1 CAT ERBB3 RHOB SERPINH1 TNC RAD21 ZFYVE21 C1H21ORF33 COL17A1 ADCK1 CDH20 ENAH FOS CCL19 SLC41A2 BUB1 RGS17 DHRS3 TRPM7 SDR16C5 MPPED2 MPP5 TNFRSF18 ZNF800 WBSCR22 SCPEP1 FBXO34 PEX13 FAM46A DTWD2 EPHA3 EYA2 TOX3 PDGFC GFRA2 RBM12B TAF3 SUPT3H CCKAR ARIH2 GOLGA7 ATP6AP1 GLIPR1L CALM1 OSTM1 ENOX2 ENTPD6 RP11-290H9.2 AMIGO2 GID8 POFUT1 SLC8A3 RASA3 6-Sep ADARB1 S100A1 RBM25 LCP1 TNFAIP1 ST13 ACAN CACNA1D PLAG1 DNMT3B CD151 HIC2 MRPL45 ZNF622 PACSIN2 SUMO3 ZFP92 ARRDC1 KLHDC2 NUP93 GPR107 RSL24D1 PRMT3 YPEL2 CDPF1 LOC426385 PSMD14 STK40 FAM126A SCNN1A ALCAM MYH11 RASGRP3 CACNA2D1 BPIFB2 ARHGAP19 PNPLA6 SRF SLC35A3 ETV6 EPGN ECI1 MRPS14 GJA5 EDF1 CSRP3 LOC420209 PCMTD1 CPNE1 EARS2 FAM122A OSTF1 PODXL HACD3 PPP1R16B NRK CSRP2 DDA1 PPP1R2 SMPX ERGIC2 EYA4 HMHA1 AP2M1 MRPS26 API5 ZFYVE19 KATNBL1 HYAL6 ENO2 RECQL TBC1D1 KDSR RABL3 LGI2 RFTN1 SNX12 IGFBP4 RGS6 FGFBP2 RP11-20I23.1 MVB12B SMPDL3B EIF5 CDH7 RNF141 GART VTN ADD1 ELOVL4 TXN2 PAPD7 TRAPPC3 NDUFV3 DNASE1 FUT9 TM2D3 TP53I11 NINJ1 MTPN PEX5 FAM65B STK11 MICAL1 RGL1 MINA HMG20A PTDSS1 TBX22 CYTH1 DMTF1 FAM45A PHF5A GAPVD1 FBXL16 HRAS CPNE8 LRRTM3 ORC5 MBP KERA CD200 PPP4R2 CKAP5 CNGA3 COL18A1 5-Sep TMEM170A GIGYF2 ACE VAC14 FAM213A DCK ACSL1 CACNA1S ASB7 FZD6 KCNMB4 ELF1 IMPG1 MRPS16 MORN4 EDA2R LOC416354 ZFHX3 ENTPD8 ATOX1 RNF13 ANGPTL2 MYOM2 PFN2 C8H1ORF52 PAFAH1B2 PPP2R2D RP11-403P17.5 COL5A1 CHD2 WDFY2 YTHDC1 NUP50 NOC2L SLC25A15 CDCA7L XCL1 ESRP2 MAPRE1 HDGFRP2 TMED11 TRPC4AP PLEKHB2 ASIP EXOC2 MBLAC2 IL8L1 GJA1 PRNP SRSF1 NFASC WASL UBLCP1 IVD MAPKAP1 HSPH1 BRD2 STK32A PPHLN1 HBE1 RBM5 NGEF SLCO4A1 RIPK2 GLP2R BPGM PHC1 POLK XIRP1 ANK3 SGTA BAZ2B WFDC1 ALG12 IGHMBP2 CRTAP IMMP1L MGLL CUEDC2 SLCO2A1 ACBD3 TRIM8 SGMS2 CSNK2A1 CLIP1 C7H2ORF69 FAM98A EIF4G2 DAP RAG2 8-Mar NCOA1 NOTCH2 TAF5 TMEM120B PERP2 TMEM184B BORA GSTT1 CDC42 NRTN BCL2L1 MELK MAP2K5 ARPC1A CNPPD1 TSKU SCN9A SLC7A9 PIP4K2A FBXW11 DMD GTDC1 PDHX CCNE2 FBLN5 NFYA IFNGR1 ARHGAP25 NCSTN HMGB1 OVAL MRPL53 OvoDA3 SOCS4 NCOA4 CDH1 CISD1 FZD3 TSPAN1 STX2 HOXB1 KLHL14 PSMC3 AAR2 CLPX ACAP2 UBE2G2 NECAB3 MYH1A MRPS17 KTN1 MMACHC NCALD UGDH TXNRD1 NPC2 CXCR1 PTPN2 RAB40B SATB1 NUCB2 TMLHE PIK3CB FSHB RIPK1 NADK AATF RLIM SSU72 STIM1 MAFB PTPRO TGIF1 ECE1 MTMR8 FASN PDS5B TCEB1 RAB33B HHATL TYRO3 TNFAIP8L1 NKX2-6 TAF12 CDC42SE2 BHLHE40 TLDC1 ARMC7 IKZF5 NAPB RTKN2 SLC6A9 ATF4 AGO4 PRIMPOL RAB3GAP2 SNX13 SULT1B PIAS1 PHACTR1 TSTA3 DDX42 LOC100858504 TMEM180 ACVR1 RGS4 AREG TWIST2 CDH5 ERICH1 SH3BP2 ZNF512B TNS1 RPS6KB1 SERINC1 RBM48 KLHL13 GCC1 CSNK1D DNAJB12 EAF2 KCTD9 FAM105A CD8B RNF4 HMGA1 ENSA ARFGAP1 STK17A MYL12A TXLNB ZDHHC21 SRSF5A MCCC1 ZYX GNG2 FAM192A IPPK YWHAZ RPA1 CD36 CFAP36 AAMDC RHOJ ADIPOR2 TFEB FOXC2 IKBKB MYOD1 GHR AvBD8 LEPROT UBIAD1 GNB1L PDPK1 CASP6 ELMO1 TIPIN C26H6ORF89 RBBP4 MEAF6 PFKP DOLPP1 OAT RNF34 PLCXD1 SEPP1 CACNA1B PQLC2 TMED8 LFNG SEC22B RPAP3 USP12-like DNAJC5 EEF1A2 PHF21A ENTPD1 SKIL CD86 APBB1IP PRRG1 TPRA1 NGF MRPS7 MAPT MTHFD2 IRAK2 HABP2 SPINK5 CREB1 CEBPG DAAM1 WASH1 BIN1 SLC17A9 GUCA1A METAP2 SRSF2 ANP32B RAD51D GSKIP MGAT4C CDCA4 C1D GJC1 MAGI2 LOC427470 RALBP1 AGPAT9 AKR1A1 RBM19 GINS1 PDGFB AvBD10 SBNO1 POLR3H CNTN1 STAU2 WDR91 CD200R1L SLC26A5 S100Z NUDT19 AGMAT STK11IP RNF111 TAPT1 SYNGR3 RUNDC3B INO80 XIAP PALM PCK1 SYT12 PPM1M LOC428335 MYBPC3 HCLS1 GABRA1 CMTM8 BRAP STX6 CELF1 RNH1 KIF2A SERPINB10 ZC3H6 ARNTL NPPC CLOCK MSN SLC19A1 KBTBD4 UBE2G1 STRAP PTGS2 A4GALT LEF1 DDT STAT5B LSM5 BORCS5 PLN RNASEL KCND2 STARD4 POLDIP3 DCX OVALY DERA CAPN1 COPS7A C10H15ORF61 CYP24A1 AP1G1 MIER1 MOB4 VPS35 CBX1 HPGDS YRK YEATS4 MRAS TRAF5 SLC47A2 TMEM68 LGALSL TRA2B STOML1 CRCP CHUNK-1 PPP1R8 BEAN1 PDDC1 UCK2 TBC1D23 C1H12ORF23 PHKG1 GOSR1 PDLIM5 ITGB2 SPATS2L ZFAND6 INSIG1 LMX1A SEC61G LOC417800 MATR3 PAK1 MBNL3 RAB14 G3BP1 USP15 F-KER OCLN FZR1 AKTIP PIK3CD SULT1B1 FLT4 TADA2A DHCR7 DNAL4 BCL10 CASP18 F2 NAA50 TOLLIP YWHAB C13H5ORF15 HNF4A RHOC C1H2ORF49 IL6RA ACSBG2 MITF RORA CRIPT BRD8 FAM222B PPM1B XPO5 MTX3 PYGB RRAGC UBE3B LMBRD2 ACADSB LYN FMOD MIB2 NCAN AHCYL1 |
| gga-miR-1783 | LOC408038 T CD74 NFAT5 XKR8 TAPT1 6-Sep VCAN NFKB1 CHST3 PCMT1 SERTAD2 CALM COL12A1 WBP2 GNOT1 CMKLR1 IL6ST MAP2K5 BIN1 RIPK1 RHOA RBM7 EMP1 ADARB1 SREBF1 ERICH1 FOXC1 ANPEP CDR2 YBX3 AKAP9 MAGI2 USP37 RDH10 DAGLB POLE3 NRBP1 TLR4 PPP1R16B RAB9A YIPF4 S100A9 CCM2 QDPR ADD1 NEBL HPS1 CLDN3 ATG9A UBL3 CKB MPP1 FRZB EYA2 UGT8 P2RY1 P4HA2 RNF185 MYH1C RHOT1 RASSF2 ARHGDIA LOC395926 IRG1 C15H12ORF65 NCOA7 RNH1 GRK4 HAO1 HMGN1 GZMA RBP RPS6KA1 ASIP PARP1 MBNL3 SMIM5 BIVM BHLHE22 CA2 HYAL6 SRF ALCAM FAM102A MESDC2 MYO1F CPA6 DPH6 SUPT3H MAL NSUN4 CD99 BCS1L DCT PLEKHA2 NEUROG1 GPR107 KCTD9 GPX2 C8H1ORF27 LDHA F2 TMEM138 MPPED2 PDGFRA RFFL SEPSECS SLC26A5 RPL9 EFTUD2 MRAS GNB5 SYNCRIP IGF2BP1 HMBOX1 PSMA5 SNRPB COL14A1 TNIP2 WIPF1 TRAF5 EPHB6 ZNF335 C14ORF2 HOXB1 CCDC127 PGR ACOX1 DCX GNG2 GPX3 CNOT1 GHRH GCLM RAB19 MTO1 MTHFD1 P2RY6 PHKB JUP XPA CENPO ANP32E CBFA2T3 CHUK TRPC4AP CARS DYL1 DYNC1LI2 UBE2R2 B3GAT1 CTDSPL2 PHF13 RP11-894J14.5 EPHB2 ACTG1 UBA52 CBL MAPRE1 LRRN1 AvBD5 KIF18B FAM129A TSPAN3 C3H6ORF120 FLI1 FAR1 ENTPD2 SSX2IP PMS1 BRSK2 RNF220 KLHL18 PPP1R7 NUP37 BET1L RPIA NUSAP1 AvBD2 ABCA1 INCENP ELF1 CLU TCF15 YIPF3 PQLC2 PLCXD1 ENTPD8 TPM1 PRELID1 XPO5 NUMA1 SLC38A2 NCOA1 ANXA7 RBPMS2 CNOT7 OSBPL2 PRPF3 KATNBL1 CLPX MAFK DCBLD2 TRIM59 PTPRG TACC3 SPPL2A TJP2 IRF1 FAM114A1 EARS2 SLC31A2 VNN1 KLF6 EFHD1 P4HA1 CUL2 MTTP RCHY1 E2F1 SPINZ PDZK1IP1 EI24 IFITM5 CBX3 CYP46A1 PLAU TAGLN PIP5K1B DEAF1 ARRDC1 SIGIRR HDAC3 SF3A1 RHOT2 LOC422214 SASH3 CREBL2 SLC30A4 PACSIN2 TNS1 TXNDC5 SERINC1 CRYL1 C5H15ORF57 TPST2 OLIG2 CTD-2116N17.1 EDF1 SUB1 ACVR1 POP4 PNISR 5-Mar LHFPL5 WBSCR22 C7H2ORF76 ALDH1A1 UBE2V1 DZANK1 ENAH ASB3 SETD3 EYA4 GOLGA7 SLC7A5 PALMD SDR42E2 S100A10 RORA SLC35C2 LOC426385 PIP5K1A SLC7A6OS LOC100502566 OGG1 RBM48 EXOC6 L3MBTL2 GTF3C5 BTG1 GLT8D1 PBK FBXL16 RASA2 SPTSSA IPPK HAGHL TSC22D3 MAP3K14 ARF1 YWHAZ MIER1 PYGL KLHL14 CSRP3 INS HERC2 PROKR2 WWC1 RRP7A SSR2 MYO1G DNASE1 PLIN1 CYB5R2 FAM213A EMB CLK2 ARR3 GNAQ USP15 ARID4A ERI1 ADAL TST CRYAA AKIRIN2 PNPLA6 BLOC1S6 TMEM230 ERN1 SMC5 CAMK2D CD79B HAUS2 WDR18 TEF SSTR2 MORC3 DEPDC6 MGAT4C NME1 TMED11 CXCL14 SLC39A9 PPP4R2 GLIPR1L SLC40A1 EIF1AY ARPP19 EXOC7 AC005943.2 FIGF VCL BTC CRIP1 ELOVL5 ERBB3 CITED4 FBXW11 BRINP1 MEAF6 FGF19 PAK1 MIB2 RSU1 ECHDC1 LOC769121 SOX18 DMD TPM2 SNAP91 CYP8B1 PPP1R21 BPIFB2 SERPINE2 SNX12 STK24 SMARCE1 MTMR3 EIF4A3 FZD10 NR2E1 CMPK1 ZSWIM7 GATAD2A GHR BLCAP STRAP PPM1M EPGN MERTK COL5A1 ZNF767 ZBTB26 TRMU ARHGAP40 DUSP12 BBS2 ALDOB TMED3 SOCS6 CKMT2 RHOC CELF1 HBG2 FZD3 PWP1 DNAJB14 ZFAND5 PHACTR1 NKX2-5 FUT9 TARS 5-Sep SGMS2 ATAD5 FGFR1 COL4A1 N6AMT2 SERPINA1 GNGT2 RAB3GAP2 HS6ST1 VEGFA ELOVL6 UTS2B DMRT1 HAS2 VPS37C LOC100859039 CSNK1D SRGAP3 SQLE ESRP2 DHRS3 PPP1R8 NR1H3 TLR21 CRIPT FBXW2 FZR1 SPATS2L RAB10 CFL2 WDR82 NFIA EIF2D MFI2 ZC3H15 TNFRSF18 API5 FTO C2ORF88 COL17A1 ETFDH NAE1 RRAD IL6RA SLIT3 STAT4 RER1 SOX2 NADK COX16 PHC1 LMBRD1 MRPL50 FASN ERLIN1 LOC421975 ACTR2 RDH5 CDV3 CLSPN DDOST C1D RAB35 SULT1B1 PPARD NEK6 GSTT1 PARK7 WWP2 ITGB1BP3 CNP CRIM1 TMEM17 ELOVL1 CBLN2 DUT NEUROG2 CBFB RAD54L REEP5 RGP1 ORAI2 TTLL5 NT5C2 BPNT1 ADCK1 CMTM7 COX4I1 TBX6 RB1 CASC4 FAM98A RAD18 DYNLL2 SDC2 CD247 STAR LPGAT1 PTDSS1 PCM1 PSPH RAD52 MDM1 ANGPT2 HMGN2 HMGB2 PREP PRNP TAX1BP1 ZFHX3 HDAC11 DEXI LHX9 MOB4 BRAP TNFRSF19 CDKN2B ALYREF DIO3 BORCS5 ABI1 MSX2 PFKP PDGFD PBLD SGPL1 PHKG1 NFIL3 PPP1R2 MCMBP EDN2 ENPP4 ENHO ELL RABL2B CCDC6 FKBP8 WDR91 ACTG2 SLC12A7 CD44 CPNE1 NREP CFAP20 AK1 SRP68 WNT5A TCF7L2 FBXO34 UGGT2 MICAL1 PRKAR1A NEDD1 ORC1 RP5-1021I20.4 CSF3 INIP HEP21 SCAF4 RBM12 CISH CHMP1B NAA50 KRTAP10-4 PDP1 THRSP ERH ASNSD1 UBLCP1 CEBPB VSX2 LOC420419 AAR2 CCR8 C28H19ORF10 RAC2 DAAM1 HIC1 BHLHE40 IL1RN KBTBD4 ELK3 CLP1 KIAA1191 PDLIM5 RREB1 NAPB HES5 SURF6 AOX2 PLEKHJ1 NGEF DCTD CPT1A SOCS5 MRPL34 C1H21ORF91 STX2 SEPT2L PMP22 KLHL2 CLTC MST4 RP1-309K20.6 TRIP13 TMEM121 NEUROD1 CLDN1 BRI3BP OVAL FBXL18 CTGF TSSC1 PSME4 MST1 CDC42BPA PCGF2 GINM1 CYB5B MEF2A PARP4 IRAK4 LHX1 DCAF12 GREM1 RAD21 ZNF410 POFUT1 ARL6IP5 GATSL2 SLC46A3 NHP2 FBXO7 GTF2A1 ACAN GXYLT1 OVALX HPGDS SHH SNRNP35 LOC415756 15-Sep PLS1 CACNA1S NR1D2 GORASP1 SFTPA1 BVES ITGB2 NKX3-2 SMAP2 IMMP1L DHRS11 GINS1 ARFGAP2 LGALSL DESI2 RBM5 UQCC NFKBIZ NUP50 LEPROT RAB22A GLI2 ORC3 RAG2 HNRNPDL TARSL2 TEAD1 TMEM26 ASH2L FMOD SNCA FZD7 LIMK1 ASB8 CIR1 FBXO8 NKIRAS2 C14H17ORF103 LBX3 COL18A1 CYP24A1 STAT5B B4GALT6 DUSP1 SLC47A1 INO80 CYP1C1 ETS1 CNGA1 TMEM254 NRGN TARDBP FRS2 HOXB4 MPST NCLN BAIAP2L1 PSMA4 SSU72 ABLIM1 ZNF622 SLC26A6 LMX1A COPE ITGA9 YME1L1 ELMO1 CAV3 MGP RALGPS2 GPR160 SLC25A6 ST3GAL3 IL16 NFASC RARA AHCTF1 RMI1 SLC35E3 FZD6 PDPK1 TUBB ACSL1 PPP1CB ARPC4 NHLH2 TAL1 UTP15 KIF20A DDT IL5RA PRLH DYNLT3 RHOJ SIKE1 EVL VPS18 UMPS NRG1 NUDT5 YIPF5 SERPINI1 PYY NFIC TRIM71 P2RX4 LCLAT1 NUP93 RAX2 COL9A3 TMEM129 HIPK3 RGS16 CTNNA2 CRMP1 PEX10 CNDP2 FAM60A ZDHHC21 SMIM3 MXRA8 CDK6 KLHDC4 CDK10 VAV2 EIF6 MYEOV2 MYBPH HHATL UBL7 FGL2 PARN POLR3H ESF1 EXD2 NRG4 ST3GAL6 CAT SELO THADA EIF5B NINJ1 TWF2 NFIB ARL5B RCSD1 WFDC1 KTN1 BTN1A1 LOC100859148 PDCD5 SYTL1 DOLK TMEM175 RIPK2 RPL34 LOC424740 WLS AVD ACADSB BORA DUSP10 BSG MTIF2 NR5A1 FANCL MEOX1 PRKCI CRYAB CHRNA6 8-Mar C10H15ORF61 SNCB CERK SLC39A13 SZRD1 FOXD1 RBM25 TSPAN15 DNAJC5 PARPBP ALDH4A1 AXIN1 MLNR ACAD8 C3H6ORF154 PRTG SMYD2 KCTD7 LPAR4 HNMT GET4 AK6 RAP2C TRAPPC2 MCM3 MAFB PCGF5 SLMO1 AvBD8 MAPKAPK5 NUCKS1 HDAC1 DRAXIN PDE4B WIPI2 EPHA5 ELMO3 KCTD4 DNAJB9 NEURL1 MAPT ANAPC10 EIF1 AGO3 TUBGCP2 BID SPI1 RNF34 TCEANC2 PLS3 PTPN6 TSPAN14 TBX5 ZDHHC5 TRPM7 KHDRBS1 RNF152 SENP8 UBXN2B CAMKMT TXNRD1 LGALS3 APOV1 RQCD1 LRPAP1 ZPBP2 INPP5K PTPN2 WDR89 DDX4 DCXR PLEKHA3 RNF141 DPYSL4 GNRHR SMARCB1 TYW5 CDX2 DTNBP1 TCP11L1 PRKD1 AvBD10 LOC395991 DCTN1 UGDH SCOC FLNB TSKU YPEL5 NCL TMEM30A LOC419112 SLC34A2 KIF2A MET GANC EPCAM TNNT2 FYTTD1 CCDC58 VAMP7 TMEM65 MAP1LC3B AP4B1 LOC421740 SGOL1 PHF21A AL158801.1 TTC7A IL10RA 8-Sep TAF5 RGS19 NUTF2 BLOC1S2 PHYHIPL TOX3 CALM1 GPRIN2 WBP4 ARHGAP25 COG5 ENS-1 GNAT2 CATH1 CDKN1B RPS14 FAM46A UVRAG USP45 TLN1 LOC396009 BMPER ATF7IP IGFBP4 DAPP1 PEX5 9-Sep ACTR5 CNBP CDH5 TIMM13 DCN PIGK DNAJC12 PDHX FXYD6 RPS12 VPS33B CRTAP FABP6 THYN1 ANK3 VPS50 RP11-403P17.5 FGF1 DDB2 BAK1 KDELR3 RABL3 AMPD3 RECQL5 ADAM33 NOTCH2 TIMMDC1 MRPS7 SCLY CFDP1 PHLDA2 PIGR DIAPH1 TMEM164 MTHFD2 NEIL1 HNRNPD GBX2 LOC431316 MMP13 MTX3 |
| gga-miR-1784-5p | STOML1 ARF1 STK32A KANK1 ANO5 SEPT2L VPS41 BPGM GTF3C5 ARHGDIA TNFRSF19 TYRO3 CPA6 C1H7ORF60 POLK EIF2S1 LOC424740 NFIX CANX XIRP1 INSIG2 ULK3 OGG1 UGT8 CTSS USP45 AGO4 C11H19ORF12 MGLL YPEL5 TMEM173 PRRX2 JMJD1C FGF14 FZD7 THYN1 SNCA LUZP2 RPA1 MSN CAPN3 PKIG ASB7 BTC GID8 CSDE1 NFYA HMGB2 FAM122A DNAJB6 LRPAP1 PANK4 IFNGR2 RNF4 TRPC4AP TNFRSF1B ADH6 CDCA7L HSDL1 FANCC NAT10 NKAP MRPL45 FAXDC2 TARDBP CPN1 CSPG5 TOLLIP AP4B1 TSKU DYNLL2 ATG5 ASNS F2RL1 FIBIN SSRP1 TP53I11 FHL5 CSRP2 G3BP1 LECT1 MAP3K14 CD1C TANK GJB1 CRYL1 MTHFD2 COG4 NRXN1 GARNL3 IFI6 ARRDC1 IHH CDH20 MPP5 PAX6 ACADSB TEAD4 SELO ZFYVE21 C7H2ORF69 GPM6B DCX BSG CARS ARPC4 THOC3 MTA1 P4HA2 SULT1B1 TSSC4 LOC415756 C1H12ORF23 FOXN2 CNOT2 B3GAT1 C1ORF146 FEN1 KPNA1 TLR7 CRADD ATP6V1G1 ZYX C15H12ORF49 PDPK1 MIER1 TNFAIP8L1 HERC3 ABCC1 RUNDC3B WDR82 CDV3 LUC7L2 FAM49A MYL12A PPP1R2 GNB5 HARS KDSR ORC5 PRDX1 PHF21A EXOC2 RPIA TBC1D1 OXCT1 CTD-2116N17.1 PRLR MYOM2 ABCA1 IRF4 LRRC59 ARIH1 WASH1 C26H6ORF89 CAMK2A ZDHHC5 CHMP2A TECTA STRAP PBX3 BAK1 RAB32 KIAA1467 SLC47A2 SLC12A7 PSMD3 PAX9 TJP2 SLC47A1 XPO1 EDF1 NME3 ACAN HIPK3 PHYHIPL CD99 FSHR LOC422926 RABL3 WIPF1 E2F4 STX2 EPB41 MESDC2 NOVA1 RPS2 OVAL TMEM229B ADAT1 SUMO1 MAPT MYD88 BIN1 SLN RALGPS2 KCNA4 VPS53 MTPN ASB8 PCGF2 RSL24D1 TMEM56 MET BID SUPT3H STK17B RAB8A EXOC5 TMEM178B TOMM6 PACSIN2 NR2C1 RGS18 ELOVL5 ORAI2 MAP1LC3B RAD21 CNPPD1 JAK1 LPP SZRD1 PSEN2 SLC26A5 SALL4 CFC1B LCP1 TXNDC5 MRPS33 COL22A1 ALG12 RGS9BP KLHL20 SDK1 MRPL20 PAQR7 ANXA7 LOC100859586 SLC7A6OS PUM1 USMG5 TRABD NCK2 TOP1 EGFL6 AMY1A FBXO32 SENP8 FAM126A FZR1 UBE2R2 TCIRG1 TSPAN6 SLC9A2 FAM222B TDRD7 MIB2 MFAP3 OTUD6B GCLM BTN1A1 PLS3 NFRKB MBD3 ELMO3 DNAJC12 TMED3 PTX3 LOC100502566 RALGAPB DAAM1 BEAN1 RBM12 DNAJC7 NBL1 MEF2BNB YWHAH TBL1XR1 DNASE1 CLDN3 GTF2H4 BG8 WFDC1 PLEK2 TPST2 TMEM180 RFTN1 GART CKMT2 SLC34A2 PIK3CA SMIM5 ZFYVE1 NAA50 NEMP2 TXN2 GFRA2 SRF CD4 CHIC1 MMACHC MYL9 RAP1GAP2 DOLPP1 RDH10 RPRD1A HMGCR TGFBR3 TRAPPC2 FAM20B GAS8 NRK LFNG SYTL1 TMLHE TERF1 LOC396479 LYRM2 FGFRL1 NR2E3 USP12-like UBXN10 RFFL CHAF1B CLDND1 CDC42 MGAT3 RBM22 COL9A2 HOXB3 APOV1 XRCC3 LSM8 TFEB RASA2 LDB3 CTGF GNG5 F2 SNRNP35 APCDD1 SMPDL3B CTDSPL2 RHOC CETN1 PARK7 NEUROG2 ACTN2 GABPA GREM1 CMC2 SRSF2 MYBPC3 MAFF ETV6 RP11-514O12.4 ZBTB17 LMO4 PUF60 ITGB2 SLC31A2 JMJD6 SERPINH1 CLPX IL5RA ERGIC2 MXD1 SUCLG1 PPHLN1 RLIM PHACTR1 HIBCH CX3CL1 TMOD3 ERICH1 SMIM12 YME1L1 GFPT1 NRGN MRPS7 PDCD6IP C10H15ORF59 POT1 SLC16A3 STX6 FBXW5 RAB9A NRTN ARFGAP2 TCP11L2 ASB12 PALM STX16 SPPL2B MED9 NDUFC2 UBE2V2 PRKAR1A RP11-20I23.1 RP11-145E5.5 OVALY EPN2 CAV1 TMEM164 HIC2 HOXC9 PLAU LEF1 LZIC OCM CREM TNC CFAP36 S100Z KIAA1143 GHRH DPYSL2 HAVCR1 BORA TRPM7 EIF4G2 CASP3 SNX12 KLHDC2 NME1 SPG21 GJC1 PALMD SPG7 BCL2L1 GOT1 STAU2 GJC2 WBP2 FADS2 DDA1 CRTAP PAH HIC1 RBMS1 RNF14 FAM105A SLBP INO80 NEK6 ALAD PCMT1 RNF34 CHRNA6 WDSUB1 BANP XIAP ABTB1 CYP1A1 MTMR8 PELI1 KIF3B SOCS4 FXYD6 CTNNB1 SCP2 HMGA1 ADCK3 CHAC1 RNF141 PRPS2 XPO5 ELOVL6 NDUFB1 PGR DNAJB9 FAM210A STAT5B BBS4 CUEDC2 ICMT ASIP PTPRO PLEKHA2 LYRM9 SLX4IP TBL1X MXRA8 NINJ1 IFRD1 DNAJC5 FZD4 MAOA CLK2 PSME3 PTPRG CUL2 FBXL21 SLC35E3 NEIL1 VPS50 AMPD3 NFASC AAMDC FIGF CEBPG SNCB TSSC1 PDGFRA LOC420419 FAM118B PRNP SMPX LOC395647 CPT1A SLC7A5 DMTF1 ASAH1 CNDP2 ALCAM CCR8 CDC42BPA GLRX PIGY HMGCS1 ID2 STK10 SIRT3 MPPED2 PBRM1 CDH6 GMFB THRSP SLC38A4 LMBRD2 AMPH RABGAP1L GJA5 TP53INP1 GNE SLC16A7 LEPROT SSB PODXL ST3GAL2 KDM5B NEK7 SEMA7A CD5 LRIG3 DHRS7C TOB2 CLEC3B TOMM7 P4HA1 CKS2 CCR5 FOXO1 NAP1L4 NFIL3 TIAM2 ACTG1 TMEM70 MASP2 ETFA VDR GATSL2 8-Mar T TMEM129 ACLY HRAS TACC3 PTBP1 ARHGAP29 MYH1B SETD6 RP11-87C12.2 RBP5 CDH5 DFNA5 TNNI2 GATA4 PFKP BRSK2 SNN GFOD2 SLC25A15 NGEF NCOA4 MTMR3 MASP1 SPP2 ATP13A4 DHRS3 RPS6KB1 BRAP SLC16A1 SIX1 ENTPD1 SOX2 ETFDH THY1 NMU NR0B2 TAPBPL WBP4 RANGAP1 SOUL PRIMPOL TBX4 ENTPD8 OSBPL2 TRIM27.1 STK17A MAP2K3 GYPC DCXR NOTCH1 MYO1C DDX42 OLFM1 CDC2L1 CTSB DNAJB14 TWIST2 DCUN1D1 PRRG1 C3H6ORF120 MBL2 PPME1 GEMIN2 CKM ZDHHC21 DEPDC6 CDH7 LOXL2 PIGA SYNGR3 CRYBB1 CYP2D6 APTX PLIN2 RASSF3 GOSR1 RAB14 PLEKHO1 RHOT1 NOS2 PLCXD1 ESR2 VCAN EEF1A2 SDCBP AvBD4 LIFR NSG1 TRIAP1 CMKLR1 LIMD2 CYP51A1 MYH10 CAB39L HS6ST1 SYNJ2BP CDPF1 HACE1 MAP2K5 PRKAB1 NRBP1 SEC61B CNP DAPP1 DNTT RP11-290H9.2 CD200R1L AP3M1 MAD2L2 ELOVL1 DYNC1I2 CD36 SMIM7 DNAJB12 DHFR SCLY MTMR2 PRKCI VTN ZCCHC6 EDN2 MCM8 FZD3 UTP15 MSRB3 PSMD2 SLCO4A1 SERTAD2 CCDC50 ZDHHC17 COL17A1 CBY1 CDKN1A DCAF12 FLOT2 GTF3C3 CLDN2 DUT TNS3 EMB NR1H3 ALG6 RRP7A SLC15A1 DAZAP1 ACP2 CDH17 PPP2R2D CHRM3 GLUL CD82 C26H6ORF106 DCLRE1B LOC100859039 TMEM170A MLF2 ELAVL1 NUP93 ARHGAP19 FOXN4 ELF1 CHMP4C LDLRAD4 LYPLA2 BTD KRIT1 GLT1D1 SDR16C5 ARHGEF39 PLEKHJ1 AKTIP CNTRL SMIM8 FBXO34 ATP6V0E1 NOP56 FYB OSTF1 SFRP2 RALBP1 SCNN1A COG5 HMHA1 BORCS5 LOC431499 DNAL4 GLP2R PSPH GMPR HNF4beta SLC19A1 RAB10 AKT1 OTX2 GDPD5 CCDC6 DENR NAIF1 GAS2 SMIM19 SCOC RP5-1021I20.4 STK11IP LGALSL TMEM120B ERLIN1 CCDC167 PDCD10 MVB12B ALDH1A3 USP37 SSR3 ZSWIM7 ELL GABRA1 SLC2A2 ACACA CPSF4 KCND2 UBE2G2 TLN1 RBBP4 USPL1 NSMCE2 SPINZ HSPD1 SLC40A1 TTL CLOCK WDR45B EGFL7 TMEM68 FABP6 RNF185 LOC416354 FGFR2 CIRBP SH3BP2 EIF1 PTRF GNAI1 MRRF EIF1AY MTIF2 MPC1 MAGI3 EDA2R CRY1 ZNF622 INCENP SHOC2 SLC6A6 RAD51D PARN CD80 PEX2 OSTM1 NADK KLHL13 WRAP73 ORC3 NOC2L ESRP2 CLTB SYT12 CNOT1 TRPV4 RHOA LCLAT1 WWP2 PSMD14 ECI1 PRKRIP1 PAN2 PRKD1 NTS FAM213A P2RY14 PIK3R5 NAGA ZFAND5 DRAXIN RGS6 ZNF302 SRSF3 SRSF1 AARS2 TWSG1 MORN4 GAL BASP1 CTNNA2 ACBD5 ZFAND6 MBLAC2 PROM1 TCEB1 PHLDA2 DNMT3B SCN9A STXBP3 KDR TMEM41B ZCRB1 SMIM18 TMED8 NCOA1 AC025048.1 SERPINE2 ACOT9 PDHX PLEKHM1 KCNMB1 C1GALT1 LOC100858797 KTN1 ST3GAL5 ANP32B CSNK1E HMGN2 MSMO1 NUMB K123 FGFBP2 FAR1 FASN CECR1 EYA4 RECQL5 DMD PNAT10 TMEM104 NDUFA5 AP2M1 MPHOSPH6 TRAF5 SPATS2L COL18A1 NCLN TRH SLC25A22 MAP7 PDLIM5 EYA2 DIO2 SLC17A9 FMOD RNF111 ACSBG2 KCTD2 HP1BP3 USP6NL TMEM123 APBB1IP CRMP1 IRX4 FGF1 SATB1 AAMP FBXW11 AMIGO2 HSPA9 PAQR8 AKAP2 TOX3 PLEKHB2 PPFIBP1 UGDH PAK1 MYH9 APELA GLI2 KCNH6 COL5A1 VDAC2 CEPT1 CD151 PTH NEURL1 POLDIP3 STAT4 SNX16 PLEKHF2 KCNJ8 THBS1 GAD1 NR5A1 WDR24 ERBB3 CSNK1D TRAPPC3 PPP1R12B MATR3 RIPK1 ACVR2A ZNF639 INTS2 ALDH1A1 UBN1 SDR42E2 PDLIM7 PIK3CD SETD4 BRINP1 FAT3 EXOC6 IRF7 HPGDS PLA2G7 UBL3 BRCA1 CRIPT MELK SNRNP200 TEX264 CACNA1D NUDT1 ADD1 ANAPC10 PEX13 H1F0 PAFAH1B2 PIK3CB MYL4 SOCS2 NUP50 SSR2 CSRP1 ENO1 SGTB TNFRSF1A CIR1 CISD1 CXCR7 BHLHE22 |
| gga-miR-1788-5p | SLC34A2 TMEM104 CCK C1QTNF2 ELOVL1 UTS2B ANKRD16 LCLAT1 PCMTD1 SNCA NADK CATH1 ANKRD26 NUP50 BUB1B SMYD2 CR1L ATP6V0A2 ZCCHC17 RGS4 ACADSB RGN MEOX1 BRE RAB32 ASB9 MPP1 RAD52 RPS6KA1 VWA9 TRAPPC3 STIM1 NEMP2 IL10RA RFC4 FEN1 ACSL1 NR5A2 NCSTN CCNE2 AGRN HERC3 FBXO34 PACSIN2 TCP11L2 MLNR ACOT9 TMEM45A UBE2G2 RP11-5A19.5 SNRNP35 LDLRAD4 TSNAX RAP1GAP2 SPG21 FGF1 ACSL5 EDNRA SUPT3H BHLHE22 MYH10 RBP5 ANGPTL1 WBP2 SATB1 UBE3B PPDPF UBAC2 SMIM3 CD24 PEX2 TEX30 CCNE1 ACTB CTSB DUT CEP63 NAIF1 ATP12A RBM19 DNAJC24 RGL1 SLC25A26 PARK7 SDR42E2 ANKRD27 CELF1 PDLIM7 CTD-2410N18.5 KTN1 SLC38A2 TMED10 ANKRD44 AC005943.2 SLC26A5 TPH1 ZFP64 PDGFA MRPL53 DHRS3 ARHGAP26 EPGN TXNDC5 TULP1 ARFGAP3 C1H12ORF23 TNFSF10 GTF2H5 BTC ARRDC1 TIMM8A ACTN1 TPM2 FTH1 MMP16 LOC427470 PHKG1 CDCA4 PDLIM5 PDGFRA CDC42SE2 BANP TRMT11 HAO1 NFIX C10H15ORF59 MRPL41 WNT11 USP45 VSX2 TRIM59 MYST2 PRDX1 PSME4 MAFF ADAM10 AMPD3 PPP1R8 SPTSSA DNAJB12 LOC431499 SREBF1 GHRHR NKAP TRIP13 STX6 CD72 JMJD4 CASP2 BIRC2 LIMD2 HPS1 MUM1 NGLY1 SUCLG1 UFSP2 COPB1 FAM60A RAB19 ARL2BP FAM3C APOD ACVR1 PDPK1 RABGAP1L NKX2-6 FAP HNMT NDUFB1 PPFIBP1 C15H12ORF65 ATP1B1 RPL37 PRR5 MTTP ARIH1 PIK3CB BMPER CPA6 NAP1L4 SCAF4 HDAC3 CHD2 KCTD14 PIGA SRP72 WDR82 INTS2 LOC396224 NUCB2 GATA3 FAM133B TMEM131 QKI MAP1LC3B MPC1 SYNCRIP ZNF706 TERF1 DNAJC5 SRGAP3 ARHGAP29 GART FAM26E NR5A1 RASSF2 KCND2 CENPH TCEANC2 RAD51D ATM ELMO1 SPI1 LIN9 TMEM175 ZP1 ADCK1 CD74 AIDA KCNJ8 IL1RN ELF1 CCT2 GDPD5 ELMO3 BTF3L4 CORO7 TXLNB PCMT1 LUM LRRC59 CD36 TAF7 CAT RP11-196G11.1 CRCP BTBD9 PRNP TMED8 CHMP2B CHMP5 YARS APCDD1 ACACA B4GALT6 CLINT1 TTC7B RNF111 ABCC6 EXOC3 ARC JAM2 FEM1B SSTR4 CMTM7 PTDSS1 RALGAPB PROM1 PTGR1 GGNBP2 ENSA UGP2 LRRC20 NUDT19 UBE2G1 N6AMT2 SCOC HOOK1 VPS41 RHOA RP11-20I23.1 NARS ZNF767 UBE2V2 TECTA CYB5B TMEM129 TBX6 CLDND1 CNTN1 LOC396380 ZFYVE19 MLF2 CACNB4 ATP6V0E2 UTP15 PRRX2 LOC422090 UBL7 TMX4 MBP CNP SEC31A RNF152 BTK ILK HAGHL LPAR4 HDAC7 TWIST1 VEGFA MYL12A FAM222B PPME1 RARRES2 TBX18 MYH9 CRYL1 COPS4 CACNG3 SULT1B1 CDC27 PIAS2 RAB5B GTF2A1 MCM3 WDR18 ID1 C11H19ORF40 ALDOC SRP9 PSMD1 MRPS18A BCL2 NFKBIZ ADA KRIT1 FK21 FAM114A1 TTPAL RP11-101E3.5 NKX2-5 TMOD3 CLEC3A ACE PDDC1 SH3BP2 TRMU ENPP2 PTGS2 ANAPC10 ALCAM LAP3 ATP6V1E1 RAP2C GATAD2A NR1H3 PEMT TMEM184C CRADD SEC22B FAM65B CTNNBIP1 AAMP CPNE1 GPR174 PSMC3 HOXB1 VCAN NDEL1 PRRG1 USP6NL SNX10 APITD1 SPP1 ENPP4 UBIAD1 SERHL2 NUTF2 MINOS1 PPM1M RS1 CRLF3 RUNDC3B EGR1 MASP1 CBFB ANP32B ADAM9 C11ORF31 RPL32 LRPAP1 SDC2 SLC9A4 TBC1D23 IKZF5 RUVBL1 CLDN1 DPH1 VNN1 CTSS TMEM65 CCDC167 LEPROT MPZL2 ACYP2 RNF7 TEC ATP6V0C DPYSL2 P4HA2 SLC46A3 PEX13 HP1BP3 IMMP1L RBBP7 LBR LEF1 ACAP2 TMPO GPR107 EPM2A CHAC2 MRPS16 TMCO3 EPT1 NCDN STOML1 PAFAH1B1 WBP4 FASN SAP130 SDK1 GNPTG FYTTD1 CSNK1D CIAPIN1 CALD1 PHF13 MON2 UBE2R2 BMP10 RNF220 RRN3 KCTD9 LRRC57 MAPT DYM LOC420419 SLC15A1 GET4 SLC19A1 SLC22A7 MED9 YPEL5 MAP7 PLEKHB1 TNFAIP1 LOC426913 POLR2B THAP5 KLHL13 TMOD4 LOC769139 TMEM26 GABRA1 CDKN1B MMACHC COQ5 DHRS11 DCX CDK1 DKC1 ZFHX3 VMO1 RTN1 P4HA1 GRIA2 NECAP2 AKT1 EIF4A2 CSPG5 HMGN2 GPM6A CYB5A CFLAR LOC770639 SEC13 KCTD2 CCDC61 RNF166 MGAT4C CREBL2 ZMYND19 COX6C SERPINE2 NFE2L1 NONO MINA ATG9A BTD COL14A1 WAPAL DDX42 ACSBG2 ABCA1 PARN METAP1 C1H11ORF54 GJD2 ST7L WIPF1 KRTAP10-4 MTA1 ARHGAP19 CNOT2 FAM46A GPR171 VSX1 PRKCI CHRM3 SSB CDKN2B E2F4 SCLY NKX3-2 CD8B GNE RRP1B TRAF5 ANP32E FABP6 GCHFR ST6GALNAC2 5-Sep TOX3 SGPL1 CTD-2116N17.1 CRYBB3 CSRP1 RNF103 SKIL DDT C1H7ORF60 LOC395991 BAK1 DIO3 CHRNB2 LPP PTPRG OXCT1 RREB1 SLIT2 ST3GAL5 GPR89B WDR44 LYRM9 IHH STX2 C5H15ORF57 FN1 NAE1 PAX6 FZD7 MYL1 CRYAA FGFRL1 EYA4 EIF5A2 TPST2 FAM102A AAR2 PSMG3 MTMR9 NRTN PFN2 ARFGAP1 GIGYF2 RAB8A IGFBP1 LRRK2 RAB28 FSHR KLHDC2 C11H19ORF12 SOCS5 HAVCR1 BPGM JUP KCNAB1 ATP6V0B PTN TMEM17 RPS12 TRAF7 NCAN SGK196 MOXD1 TNNT2 VDAC1 GOLGA7 CCDC50 HNRNPH3 INVS PMS1 ANXA5 ACTG1 BEAN1 STX16 C15H12ORF49 APOC3 AKAP9 RPL35A EIF2AK2 SOCS3 TXNDC12 TNC NBL1 DTD1 NUB1 CANT1 SERPINA4 CISH FBXO9 LYG2 GPR149 GAPVD1 DNAJC6 CCDC28B K123 CASP3 AMPH NDUFS3 LOC395159 DCN RTN4 PWP1 C10H15ORF61 CTNNA2 ZNF330 GALNT18 RGS20 BLOC1S5 TXNRD1 CARD11 PSPH HAGH DCTD IL6ST UBA2 FBXO7 STRA6 RP11-195F19.29 IFRD1 METTL22 CECR1 CD80 EAPP SLC47A2 UCK2 FAM46C SUGP1 MAP2K3 STUB1 CDC25A HMGCS1 C3H6ORF120 CYP1C1 GTDC1 BIVM GPR143 RNF11 SELO GABRB3 ATP6V1D MICALL1 YTHDF3 NFAT5 TOP1 FGF2 CRK LYRM1 LOC770548 NPC2 PHACTR1 IL2RA SLC25A36 SENP8 RASA3 GNAL DAZAP1 USMG5 APC2 PPARA LOC100858504 NUSAP1 LOC431325 ANK3 PPM1B XRCC3 TPT1 PTPRZ1 NCAPH GLI2 ECE1 GLOD4 RBX1 NDUFC2 WSB1 P2RY6 S100Z NCOA1 CDH7 GLCCI1 CHRNA6 LOC424740 LSG1 FOXA2 PNAT10 MYL9 PDCD6IP ITPK1 TMEM170A C4H4ORF29 SF3A1 VPS53 MYO5A DYNLL2 CCNA2 GBX2 MAVS FBXW5 COX4I1 DAPP1 LOC396009 CCNB2 GARS CUEDC2 RBBP4 MST4 TGFBR2 AREG ADIPOR2 RPAP3 KLHL18 CD1B WWP2 PSMD2 ACAD8 SETD3 SYT12 TAGLN ASB8 FHL5 MFAP3 CD200R1L CBLN2 ST3GAL2 NCAM1 FABP5 ALDH1A3 CRH CYP4V2 PLIN2 NPHP1 UBXN2B MALL EGFL6 PAFAH1B2 SNN PDE6H SGOL1 GTF2H4 CRYZ ASB3 B4GALT2 SOX14 NHLH2 RAB1A DHODH MTIF2 COPS5 ZFYVE1 SEPW1 GHRH AGO4 FOXO1 BBS4 CERK CRABP1 BEND7 CELF2 TAF11 GNRHR TBK1 SAR1B FAXDC2 CCDC80 YWHAZ ANKRD40 RAB22A DHFR CST7 NFIA ALG12 GEMIN2 TCEB1 CYP51A1 FUT9 FZD5 COPG1 PRKRIP1 C14ORF2 ENO2 NDP ODC1 MOV10 PLEKHO1 DUSP10 FIBIN STK24 KIAA1143 C10ORF2 MTMR8 KLHL14 TEX264 RIPK1 CNDP2 RBM12B HEP21 PMAIP1 NEURL1 CREB1 SNX16 DESI2 ATP6V1G1 AC025048.1 ADAT1 PPP1R3E MCM6 TSPAN1 PSMA4 DNMT3B LOC100859722 MPPED2 PCDH10 PNO1 SLC7A5 TAL1 ZDHHC8 PARS2 SZRD1 P2RY14 CCNL2 XIAP DUSP4 KLF6 SIGIRR S100B ZCRB1 ATIC MGLL STK17A THPO GOSR1 LOC426385 ETV1 FOXD3 PLEKHB2 KBTBD4 SEMA3E PRTG RAB14 ABTB1 ELL MMP9 ARMC1 TBL1XR1 TWSG1 SBNO1 CAPZA1 BTBD1 FZD6 KLHL20 OPN5L1 YIPF4 TAPT1 MELK HOPX ZBTB34 TBX4 POPDC3 RFXANK LOC428335 TBC1D1 MGAT3 JAK1 TMEM11 NR2C1 POPDC2 RALGAPA1 VDR CCR8 MTMR2 RFC1 ARMC7 PRLH NR3C2 COL8A1 BORCS5 IRF2 ZDHHC21 TCP11L1 SRPR NRG1 BLNK SLC16A3 RRP12 GSKIP USP13 PRKAR1A ZNF622 CARS TWIST2 SSRP1 PITX2 SNRPD3 ACP2 ICMT RLBP1 VPS33B TLDC1 EHD3 CLN8 CDK6 HIC1 EPHA1 PSMD14 LCP1 IPPK LONP2 TRIM71 THYN1 THRSP C1GALT1 CACUL1 PCDHGC3 E2F1 DTWD2 TUBB2B ENOX2 PLA2G2A BRD2 RER1 FBXO32 CD72AG KPNA2 SLC35A3 AARS2 KIF18B IRX4 SH3BP5 SEPT2L RFTN1 BPIFB2 SHISA2 TBL1X LOC422926 SPATS2L ID2 ULK3 MYL4 CCKAR MAP6 ATP1A1 ARID4A CRTAP KCNN2 SOX3 PRKAB1 FBLN1 ZC3H6 RP11-145E5.5 5-Mar DOLPP1 SIKE1 RP11-514O12.4 UBE2H MFAP1 FGF10 AAMDC UQCC RHOT1 SLN SERINC2 DLD MRPS11 LOC408038 PEX11G CENPF DOLK NRP1 CLSPN FAM21A MGAT4A CDPF1 HNF4beta SETD4 NFIL3 |
| gga-miR-3535 | MATR3 DDX4 ZYX ARF1 NCOA7 ANK3 NFAT5 SEMA3D RTN4 CDPF1 VPS41 LYN TICAM1 EDA2R AMIGO2 ENAH EXOC2 PDHX HNRNPH3 T SAT1 TJP2 MYO1C IFNG UBIAD1 AC113404.1 PARP1 A4GALT CLDND1 SLC20A2 STK24 SYT1 SPP2 CACNA2D1 NGEF MAVS LRRC28 DHRS11 DHRS3 IMPG1 IGFBP1 RECQL CDC42 BZW1 CRADD TRAF5 MIB2 ZNF706 LIN52 EIF2AK2 GZMA RPL15 RSL24D1 NADK ANKRD16 RBM24 CLTC FAM222B EPN2 KIAA1671 GLI2 ACSL1 NCOA1 PSEN1 TBL1X ADCK3 MYBPC3 CLDN1 NAP1L4 NUP93 BID EGR1 CD82 FAM46C COL5A1 SDC2 NRXN1 APPBP2 FRZB PFN2 SLC47A1 ARFGAP1 LPGAT1 MST4 N4BP1 FABP6 ELAVL1 PAFAH1B1 ZFAND6 DCTN1 B3GAT1 NT5C2 RNASEH2B SLC1A2 FASN ENTPD1 NFIL3 TRAPPC2 RP11-290H9.2 ST3GAL3 PRC1 CPSF2 RP5-1021I20.4 USP45 WDR1 8-Mar CNTN1 SOCS4 RAB8A DOLPP1 TNC TYRP1 ARHGDIB WAPAL ATM TCF3 GALNT1 CTDSPL2 ACAN IVD RAB9A NDUFB1 HABP2 CAB39L CHD7 PELI1 CDC27 ACACA CSRP3 DNAJC5 LAMA1 SERPINI1 C10H15ORF61 MTMR8 CD1B AHR CNTN2 CDH20 TEAD1 F2RL1 STX7 DPH1 SYCE3 ST3GAL5 P2RX5 MAGI3 EPB41 RNF20 FABP5 CPT1A PDCD6IP GNB5 PLEKHB2 AHSA2 VIT PCMTD1 MAOA MOB4 AANAT SYNGR3 NECAB3 RAB14 RFFL GAPVD1 RALGPS2 HNRPK DDT C2ORF88 CELF1 GPR174 WDR82 CCNE1 RHOG ACVR2A ARIH1 HMGCR TEAD4 RAF1 C1GALT1 SNCG BTBD9 PBRM1 SLC34A2 PRKCI HIPK3 RIPK2 SALL4 KTN1 RB1 VDAC2 GLCCI1 B4GALT2 TARS SEPT2L NCOA4 EPYC SNX3 TBC1D1 SMIM5 ROR1 ASCC3 MYOM1 DCTD IRF1 UBE2V2 RAG2 DNAJC12 CRTAP KATNBL1 ZNF639 TNS1 ACVR1 HMG20A VCAN CMC2 AAR2 GBAS BRSK2 ANXA11 9-Sep CYP2D6 SLC24A1 PDCD5 CHMP4C PLS1 SBNO1 CHD1 NOS2 PEX2 PSMD14 PFKP MBLAC2 PARN F2 ELAVL4 ETFDH STX17 CCR5 ARGLU1 DCAF7 LOC426385 SUB1 MTPN PRNP PTN RNF111 SOD2 STARD4 CD24 VPS50 STK11IP SPINZ TCEB1 TNFAIP1 NEDD1 GDPD5 CBFA2T3 NME5 DRAXIN KCNIP2 ASNSD1 LMBRD2 SCOC KLHL20 SEPSECS HAVCR1 DNAJC6 TTL JAK1 RPL29 RELL1 SGK3 GPR89B SLC2A2 MEAF6 MYOM2 KERA CD151 LIG4 RTKN2 GABRA1 TRABD KIAA1143 LYRM9 RASSF3 TMEM70 DNTT BTC COPB1 HES5 TNS3 NCALD MTMR9 FZR1 ENOX2 NEK7 MCM8 FAM126A SATB1 STIM1 TNFSF10 FSHB RRAD COL8A1 JAM2 ADD1 PHTF2 APELA CNBP MORC3 GATA4 BLOC1S6 ARFIP1 DENR NR1H3 TXNRD3 UBN1 PIGA GLP2R EGFL7 USP15 LRRC59 RREB1 SLC40A1 ELL APITD1 CACNA1D 15-Sep OPN5L1 G3BP1 RAB35 UTS2B DEXI ADIRF RORA CLPX PCM1 JMJD1C CHAF1B RALGAPB GJA5 SGMS2 TMED5 TMEM56 STAU1 LOC418667 CPA6 C4BPA TADA2A TNFAIP8L1 P4HA2 EIF5A2 GALK2 IGSF1 ATG9A SLC6A9 CYP51A1 GTF3C5 ANGPT2 PCMT1 CREB1 MAGI2 ERICH1 GARNL3 WWOX FAR1 PRRG1 MPP5 LYG2 YPEL2 PAIP2 OSTN TACC3 HSPD1 TMEM229B HAS2 FIBIN C26H6ORF89 CYTH1 MAPKAP1 RFTN1 LOC422926 RP11-834C11.12 PRLR ANKRD26 OC3 C1D OSER1 IMMP1L MBNL3 SLC26A5 GLRX ORC3 RBM12B RGS17 GPR149 FBXL21 RAB10 UBE2F C20H20ORF24 LRRN1 WASL PIGR MPPED2 CHRNA8 ANGPTL2 VPS45 RGS9BP DEK SLC16A1 DTWD2 UGT8 TMEM5 NR0B1 XPO1 LOC422426 RGS4 SNX16 CHORDC1 CUL2 RBM22 P4HA1 NR5A1 RAB33B KDM5B PMS1 DMRT1 RABGAP1L POPDC2 SLC9A2 HES4 SERTAD2 LCP1 RASGRP3 HDGFRP2 LZIC USP12-like RCAN3 CCDC93 VIP ST13 ANO5 WBP2 KCTD9 CSNK2A1 ASIP AREG CD200 LOC427470 GPX7 EGF DCUN1D1 CISD1 NUDT5 DAAM1 BRMS1L GLUL CDCA7L GCM1 SCIN LPP C1H7ORF60 TP53I11 COL18A1 JMJD7 IL8L2 NAV3 IL6ST NEK6 GTDC1 TMEM120B MESDC2 FOS BFSP1 AARS2 ESR2 TRAPPC3 SLC8A3 C1GALT1C1 LOC420209 APC2 SMPX ATF7IP ZFP92 SQLE PACSIN2 ENS-1 TRMU SERPINA4 ARHGAP19 POLK CDH6 ADARB1 PIK3CB MRPL38 FUT9 SPPL2B RPS14 KCTD2 PLEKHO1 STRBP MYD88 NR2C1 PDP1 LEF1 SERINC1 YWHAG MTIF2 SLC17A9 MGP SUPT3H SERPINB6 KLHL15 GPR107 PARPBP EXOSC2 FGFBP2 TBC1D15 FAP DPF3 MGLL GHRH RFNG DMTF1 ORAI2 PSPH MVB12B RAD21 STXBP3 CD36 DCN WDFY2 GIGYF2 ABHD13 EDN2 IL16 GFPT1 TLR2A BORCS7 GPBP1L1 TMEM254 FRS2 RALBP1 ELOVL5 POU1F1 ENTPD8 ZFAND5 GLT8D2 JMJD4 COMMD8 C14ORF2 MET MEF2BNB ELOVL1 BPIFB2 DDX42 GREM1 GPR160 PRDX1 TAPBPL CREM OAT GJA1 NFKBIZ BTD MYL9 PRKRIP1 FSTL1 PTGES3 C11H16ORF70 CEP19 GNB1L NUSAP1 RIPK1 LOC420419 PANK4 SPCS1 ECHDC1 SLC25A22 TMEM230 FOXN2 TMED8 C3AR1 GMFB IPPK TNFRSF19 SSR1 HESX1 ABCC1 PSEN2 TMOD3 CTGF DAPP1 LYVE1 TSSC4 ASB12 AMY1A ALDH1A1 FAM20B PIK3R5 FAT3 MRPS17 C4H4ORF29 WIPF1 COL6A2 BRINP1 BCL10 FAM122A PLA2R1 GCHFR CCL20 PUF60 NFKB1 CD200R1L MON2 SLX4IP ALDH3A2 PLIN2 EVL VPS4B PODXL PLEKHM1 LOC418666 PDE6H SNX10 PAK1 NREP MTFR1 SYTL1 IFIH1 SDF4 CMPK1 ST6GAL1 LOC420849 INTS2 ANKRD27 GNB1 PAPD7 MYOZ2 MORN4 FBXL16 MYO5A COL6A3 KCTD7 OVAL CDC42SE2 NFASC MGAT3 FAM213A XIRP1 AKAP2 TCP11L2 CDH17 FBXW11 PDPK1 UBAC1 NRG1 OSBPL2 IGFBP4 RLIM ADAM10 ART4 ACAP2 PPARD CSDE1 EYA4 TOP1MT AGO4 FMOD XIAP ATP6AP1 AZIN1 GPR146 CANX ABCA1 COL22A1 CHP1 FAM105A ACADSB CNGA3 SLC35E3 SLC35A3 HYAL6 C14H17ORF103 SCYL2 RGS7 SLC9A4 ALKBH3 LCLAT1 FBXO34 NINJ1 BASP1 ALDH1A2 ZBTB26 EIF5 STAU2 CDH5 PIK3CD ZDHHC8 E2F5 IKZF5 MXD4 DDX6 TFEB E2F4 RRP7A LOC770548 SRSF5A MAP6 SLC46A3 CPSF6 NCLN RBP KIF5C SH3BP2 PPP4R2 KLHL18 CTDSPL SLC37A3 SNRNP200 CALM1 INVS HPGDS CUEDC2 DNAJC15 C7H2ORF76 LEPROT BIRC2 CSNK1D TASP1 PDS5B MTMR3 SDK1 CCKAR CHTF8 RAP1GAP2 QDPR ALG12 ZNF622 SERPINE2 FYB MBL2 ABHD17C CHN1 ELF1 DOLK TBK1 LOC395159 LUZP2 PKM CFLAR VNN1 CTNNB1 TAPT1 PPP2R5C NUP50 SPG21 MGEA5 KDSR EXOC8 CHRM3 BHLHE40 FAM65B AvBD8 KLHL13 SUV39H2 PRKAA2 NCAN DIO3 FHL5 FHIT STX2 APBB1IP FBXO8 CD80 PHACTR1 TMCO3 PTPRC SYNM TMEM41B SLC38A4 OTUD6B COL9A1 KPNA1 FAM210A RGS6 ZP2 BORA MSRB3 G2E3 NAA50 PRIMPOL C15H12ORF49 SEC22B TMEM30A PHLDA2 NSG1 FAM49A RPRD1A C1H21ORF33 WBP4 TWIST2 MAPK6 CLIP1 CNTN5 SOX3 ORC5 FANCC RBM48 LYSMD2 UGP2 GEMIN2 IRAK4 TPRA1 LIFR CLN8 FPGT RPS6KB1 ZCRB1 ALCAM ENY2 MMACHC CAMK2D PTP4A1 BCL2 NRK RHOA SHOC2 MAPRE2 RHNO1 EXOC6 XPO5 ABHD17B ALAS1 LOC422249 RPL9 NR0B2 PEX13 PBX3 ENSA SUGP1 NEUROD1 BRAP MTHFS SETD3 INO80 VAMP7 TUBA3E BAZ2B COL17A1 DRG2 CD93 MYL12A RABL3 AKT1 RP11-20I23.1 CDH7 CTNNA2 ANKRA2 LGI2 CMKLR1 USP6NL CDH11 BPGM NRG4 RNF185 LOC431324 JUP DUSP12 TBL1XR1 CYP24A1 RNF4 CNDP2 TOX3 LOXL2 TNFRSF1A PAX6 TMX4 UBXN2B TIMM9 KLHL24 WASH1 CTPS2 AP2M1 RASSF5 HDAC4 NFU1 NGF |
| gga-miR-6550-3p | LOC770639 TEAD1 ITGB2 BIRC2 HABP4 PTPRO MSN PRIMPOL RNF14 PPARD RASGRP3 KCNN2 FAM222B ABRACL RBX1 EGR1 SNRNP200 LOC100859039 NRK ANGPTL2 CNRIP1 FZD7 TREM-B1 TRIP13 PAAF1 COX17 GFRA2 PPFIBP1 OTUD6B SCYL2 AAR2 SLC16A3 MORC3 CD200R1L SYNCRIP CST7 ST6GALNAC4 MYH1C RNASEL HIC1 SGK1 RPS6KB1 CBLN2 PTPRU ARRDC1 FDX1 KLHL13 CD93 SLC34A2 CDH20 ANKRD40 LOC416354 DYM GHRH SSR3 FBXW11 SLC2A2 SRF TFEB IRF4 RRAGC SMIM3 DRG2 RPL6 AP2M1 RECQL5 GNOT1 GNLY PRKD1 LOC419112 ACSL5 PPP2R4 SNCA RAB32 ARPC4 VPS4B PHF5A PARD3 DYNLL2 LMBRD2 THAP5 HSPA9 EDA2R LCLAT1 WDSUB1 TMEM173 XPO5 8-Mar GFOD2 PSEN2 FKBP4 GMPS RLIM SNX16 LOC396479 TACC3 LOC395095 APELA RTN4 ERLIN1 G2E3 PCK1 STRBP RBM19 FBXL21 ANKRD27 PPHLN1 FABP6 TCF3 FSHB CRYAA MAOA ANK3 MGAT4A MCL1 NUP50 NIP7 CTSA ID2 UBE2R2 HYAL6 SERINC1 PEX2 NAT8B GRK4 FHIT NHP2 SEPSECS MRPL45 PANK4 FAM76A PRLR CDC42 CASQ2 PXN NTSR1 NPFFR1 LOC420860 IRF1 ZDHHC21 DNASE1 MMP9 C7H2ORF69 METAP1 SEC22A GINS1 RAB8A ARIH1 UFSP2 FOCAD PALM WDR83OS CNBP SLCO4A1 TNNI2 ALDH3A2 COL22A1 RAD9A HNMT NADK YWHAB RAB35 PMP22 ATP5B AKAP2 SLC6A9 GLP2R PLEKHB1 BLCAP APP GDF3 ADAM9 SEPT2L ZCCHC17 ALKBH3 EIF4A2 CRTAP BCL2L1 C8H1ORF52 GABRA1 PCMTD1 OAZ2 SLC40A1 NDUFA10 BORA NPY PRKAR1A RNF111 CDHR1 CDH1 ALAD NAA50 POPDC2 WASH1 SSPO 6-Sep RGS19 LOC100859249 NT5C2 CDC25A ZNF512B TAF7 ENTPD1 LOC770548 PIK3CA SCOC RGS4 TRPV4 TOX3 WDR44 G0S2 CACNA1S NRF1 CBFB PTPN2 COL2A1 EPN2 KIF4A MTMR3 PTPRZ1 YEATS4 RAB33B PAQR8 CTD-2116N17.1 ARL8A RP1-309K20.6 AIDA TMED3 TMEM230 HSD11B1L MVB12B LPL GLI2 ST3GAL2 FAM53A EPHA1 STIM1 PITX2 PCGF2 FAM213A TLDC1 TIMM8A HERC3 PDPK1 DYRK2 PUF60 TSC22D3 CARS RPL9 CHMP2A TMX4 PRTG GOSR1 PDS5B SERPINA4 ARF1 MRPL20 ULK3 GMNN CCNB3 ZNF622 XIRP1 ENTPD6 TNC MAP6 RP11-834C11.12 FBXO34 ST3GAL1 ADCK3 N4BP3 CDH6 DYNC1I2 CEBPG TAF8 CSNK1D SERPINB6 GNG2 COL6A3 KRTAP10-4 HOPX VPS41 XIAP FAM114A1 CHRDL1 MAGI2 HN1 SPPL2B APC2 P2RX1 ATP6V1E1 SGPL1 ERI1 MRPL38 STARD4 PRRG1 CELF1 SRPR EDN2 LEPROT CCDC6 ACTG1 UGDH DEAF1 TARDBP TAGLN FAM105A STAT4 BCL10 LOC419429 CCNE2 IPPK CSRP3 MGAT4C EARS2 COX14 C26H6ORF106 ACOX1 TPM2 MPZL2 LOC100502566 TBC1D23 COL18A1 ST13 TRAF5 SNAP23 ASB7 RP11-87C12.2 MYBPH RABL3 TMEM180 RFXANK CDH5 COPE FYB PTP4A1 CRMP1 KIAA1671 COL8A1 ELOVL5 PAFAH1B2 RAG2 MYEOV2 CBX4 TEX264 JMJD4 PDHX SIX1 APBB1IP NKAP SGTA ORC1 SLC35A3 C1H12ORF23 FOXO1 DCN PHKG1 CEBPB RHCG WDR45B CSRP1 GOT1 PIK3CB CDCA7L UBE2D1 SERPINI1 SDF4 NUDC PPP1R3E AKTIP NBL1 DIO3 IMMP1L RREB1 LCP1 VAMP7 ACSBG2 RELL1 RP11-49K24.9 CPNE1 RASSF5 GTF2H4 TMEM170A C1GALT1 CNTN5 CCDC61 DDT HMHA1 C1D SRSF2 PAX6 LOC396009 TNS1 RASA3 AvBD8 YPEL5 NOS2 GRIA2 PTK7 RANGAP1 ELF1 PARD6B ST6GAL1 SOCS4 TAL1 KRIT1 COL6A1 RAB24 PDCD6IP CHD2 CANT1 RBMS1 DAZAP1 USP45 MPP5 SLC39A13 SYNPR ARPC1A GNG13 EDNRA FAM118B ABCA1 SMIM18 MLX HDGFRP2 QRICH1 PARK7 HPGDS ARHGAP25 MAFF CDK1 TSPAN6 HOXB4 ABI1 AC113404.1 RNF7 RAD54L WDR5 GPATCH2 SFTPA1 TRIM55 KCNJ8 CDC27 SERPINH1 TMEM11 IPO13 BRD2 KDSR PPIB CANX TNFRSF1B PRDX1 NRTN MTERF3 PAFAH1B1 LOC426914 PTDSS1 DNMT3B CASP14 LAMP2 OSBPL2 PEX5 TST SLX4IP RNF185 CDC42SE2 ERBB4 EMB PAIP2 INHBB ACACA ANP32B DDA1 SULT1B1 WDR24 NUDT19 CDC2L1 ODC1 CRYL1 SPATS2L FMOD ALDH1A1 GNB1L RALGPS2 MRPL53 ST6GALNAC2 ACLY LIN9 PLN GTDC1 MRPL15 LZIC CRYBB1 KDELR2 IMPG1 POU2AF1 TMEM175 MTHFD2 RHOC SLC25A22 DMTF1 PRKAA1 SET DLST SLC22A7 QSER1 BUB1 AKT1 GLUL DAAM1 CFAP20 PLK1 BMP15 FASN UCKL1 DFNA5 TAF5 LRRK2 SLC46A3 PARN DEK ZFP64 CMKLR1 KLHL14 MTPN RLBP1 CCDC28B EGFL6 SETD3 MRPS14 DNAJC5 PDGFD MAD2L2 PMM2 CXCR7 HMGA1 ZCRB1 SPP2 SUMO2 PIK3R5 XPA BRSK2 ZSWIM7 GARNL3 L3MBTL2 SYT12 FHL5 GLT8D2 NINJ1 TLR21 PLEKHO1 RGS16 PHTF2 LOC100858444 SLC1A2 C1ORF146 SLC47A1 HRAS RAB3IL1 CDH17 ETV6 STX17 MRPS7 NFASC FZD5 TPRA1 TOB2 UBN1 ABCC1 P2RX5 MYH9 IGF2 SYNGR3 CKB BRINP1 RELA LEPR NECAP2 TAF11 HIBCH AMPD3 APLF AHCYL1 SLC17A9 NFIL3 PBRM1 FBXW5 TEC SCG5 CPT2 BRAP TMC2 PKIG DPH1 INTS10 EYA2 PLCZ1 C1H21ORF33 MAP3K14 SLC16A1 FAM45A MGME1 TTL CHUNK-1 ACAN HHATL TBX22 PRC1 BRE CD82 ELP6 TNR FAM46A SMIM12 C14H17ORF103 NAV3 CD5 CLK2 CBX3 AAMP ACBD3 ARNTL FOS KTN1 NELFA CD200 HNRNPDL TLR1A APTX TMEM17 RGS7 SLC17A5 TMPO CAV3 TRIM59 CASP2 SCIN PHLDA2 PLAG1 TPST2 OCLN IFRD1 MFGE8 GJA1 ACADSB BTC APOV1 THOC3 NKIRAS2 STK11IP DYNLT3 SMAP2 LOC396224 PLEKHB2 DCK ODF2 TM6SF1 CTNNA2 FPGT HDAC7 FOXD2 ADCK1 DNAJC12 ZBTB34 GDPD5 SLC25A15 SDK1 TCEB1 RDH10 CSPG5 PMS1 AMY1A CCNC GATAD2A SERTAD2 TMEM254 HNRNPH3 RMDN1 FZD3 WBP4 PSMD2 ERBB3 DNAJB9 TRPC3 AC025048.1 CSRP2 RP11-5A19.5 B4GALT2 SRP14 PSMD14 GJA5 COL9A2 C11H19ORF12 VTN ADA SLC9A2 FAM126A IL2RG FKBP8 COL4A1 TNFRSF19 CNP RHOA FEN1 UGT8 ENO1 TMEM189 DEPDC6 ARHGAP26 LOC396531 COA7 CCR8 C15H12ORF49 TPM1 PDE6H GLYR1 PPDPF SMPX CLOCK NECAB3 GFRA1 USPL1 YPEL2 KLHL18 LIMK1 PCM1 SLIT1 CD72 TNFAIP1 WIPF1 LGALSL CNGA3 FBXL16 PPP1R21 PSMC6 S100A10 GPX2 TK1 HMG20A TCF21 SLMO2 GPALPP1 FGFRL1 LOC100859427 PHF13 CAPN1 RAD51D TANK CNOT1 CPA6 RFTN1 ABCF2 CYP4V2 IGHMBP2 CECR1 SLC38A4 S100B CEP63 RAC2 TADA2A CNPPD1 FK27 HPCAL1 YWHAH YWHAQ CISD1 SELO VSX2 NOTCH1 AARS2 IGFBP1 C10H15ORF59 NCOA1 CD151 CATH1 SLC26A6 RCSD1 MYLK2 MEAF6 ATF4 UBE2V2 ATP6V0C STK40 FAM20B MAF CKMT2 ELMO3 ZBTB2 PSMD1 GREM1 FAM18B1 GRIA3 NEURL1 TRIB2 NDEL1 DEXI TMEM179 B4GALT6 GALNT1 NEIL1 CITED4 SMIM5 TMEM120B B3GAT1 RBP5 C11H16ORF70 UBE2G2 IGFBP4 MYO1C HIC2 SMIM7 NECAB1 DUSP10 MRPL50 CDKN1B EIF2S1 IRAK4 ZDHHC5 STAT5B FGFR2 ELN HSBP1L1 H1F0 AZIN1 PIAS2 VAC14 B3GNT2 OPN1SW SIX2 PSMC3 RBM12B YWHAG ZFAND6 ANXA11 GAPVD1 RAD21 MINA PPP1R8 YRK PTTG1 PDLIM5 MAPRE2 TIPIN LUZP2 CDCA4 GJA4 FGF13 GLCCI1 CREB1 SARS PTPRG SLC15A1 MAFA ACBD6 MGAT3 PKM NEK6 PRRX2 SPPL2A STAU2 RNF4 C1H7ORF60 REEP5 FAP CDK10 GORASP1 PROKR2 PAPD7 TMOD4 PHF20L1 APOC3 FLT4 CD1C CCKAR ACVR1 RAB9A RHOB MET RALGAPB ZNF800 SNX10 RAD52 TXNDC5 TRIAP1 GCLM EXOC6 ENOX2 STK17A DCBLD2 CUL2 ANKRD16 RAB11A PRPSAP2 NFRKB DAPP1 UBE3B LIFR ACAD8 TMLHE MTFR1 E2F4 MGEA5 NR1H3 NFIX PFN2 RBM48 TYRO3 CLDN1 PDP1 TSKU SLC16A9 SP1 GHSR SGTB ARFGAP2 VMO1 MAP1LC3B CDPF1 VIT BRCA2 ECE1 C20H20ORF24 SRP68 RP11-20I23.1 ARHGAP19 HACD3 MST4 HAS2 TBC1D1 STK10 USP6NL SYK ENTPD2 BBS4 BSG PDGFB TMEM178B TMEM121 MMACHC CRYBB3 BASP1 HPS1 SH3BP2 RAB14 WDFY2 SNCG GARS LGI2 COL5A1 MOV10 NSUN4 PIGA SZT2 FZD1 MAFK CETN1 IL1RN WWP2 PIP4K2A CDCA3 PAQR7 PODXL HIPK3 AMPH ASCL1 YBX3 STX2 ZFHX3 STOML1 CSNK2A2 QARS TERF1 SATB1 MARCO TMEM138 LOC420849 ARFIP1 MYH11 CBLN4 5-Sep GPR107 EIF1AY PRMT7 SUB1 EXOC2 SLC19A1 PDGFRA CCR5 PPP1R2 FHOD1 IVD ADARB1 UTS2B NRN1 LPP ZW10 PDZK1IP1 NOTCH2 MCM3 DNAL4 |
| gga-miR-6555-3p | PELI1 POPDC2 SGK3 CDC27 EHMT1 ELMO1 ADCK1 KPNA1 VAV2 NUP58 GPR146 ALDH3A2 CKAP5 ACADSB BFSP1 CD80 NCALD TNS3 SLC31A2 RP11-77K12.7 FAM213A CRTAP MRAS C11ORF31 FOXM1 XPOT HMHA1 ZFAND6 ALDH4A1 C1H12ORF73 GNB1L STXBP3 CEPT1 RAC2 RP11-834C11.12 SYNPR ZCCHC6 PIK3CA TSPAN6 RAB32 CREB1 LMBRD1 CPSF6 UQCRH DAPP1 BPGM IRAK2 ELOVL4 UBXN2B SRGAP3 NPAS2 NCAN LUZP2 WBP4 IL6ST ACLY CDV3 IBA57 CLU TSSC4 NAP1L4 FMOD SLC35G2 RGS19 QSER1 GFPT1 SEPT2L CCNE1 ACSBG2 NT5C3A CD36 P4HA1 MAL2 JARID2 LDLRAD4 NOTCH1 ANP32B CTGF BORCS5 SQLE CDKN1B VPS4B SEC22B RP11-195F19.29 NKX3-2 PDGFRA TPRA1 ZNF384 FAM214A SCP2 GATA4 IRF5 RHOA FGFR2 CHRM3 TRDMT1 MAD2L1 TRAF7 UBE2V2 SSB NUCKS1 ATAD5 CDK2AP1 GJC1 ST3GAL5 ERGIC2 DCTN1 RUNDC3B SSR2 RAB8A SOX17 TMEM123 KIF3B XPO5 UCP3 ARPC4 RORA GPR89B AAR2 FAM3C SMIM19 SLC15A1 FOXA2 RHOG FADS2 ELF1 CKMT2 TBC1D22A ZBTB2 LIFR GLT1D1 RHOT1 GNB2L1 GET4 PSME3 TP53I11 PPARD NT5C2 CTD-2116N17.1 CSTF3 PRPF3 LIMD2 LOC427470 LOC431499 FANCC RASGRP3 SLC6A9 P4HA2 SNRNP200 FZD2 IPO13 FAR1 VAMP7 RP11-290H9.2 RFNG ARFGAP3 CRELD2 FBXL16 FLOT2 PRL TACC3 FOXO1 STUB1 AMIGO2 PTTG1 COL3A1 YBX3 MRRF RHOB YPEL5 RABL3 PRPSAP2 FLNB MTFR1 CENPH CD5 ORAI2 WBP2 KIAA1191 SERINC1 FAM122A C11H16ORF87 SPPL2B RNF4 PAPD7 RIPK2 SRSF6 TBC1D1 MATR3 MLX SMC5 SRC POLDIP3 BEAN1 GDPD5 YPEL2 MRPL46 FGFR1OP2 APBB1IP AATF TMEM170A ADARB1 LFNG ATF4 TCEB1 SLCO4A1 NAV3 UGDH FBXO34 YWHAQ TRPC4AP TMEM184C DNAJC5 PNPLA6 CNGA3 KATNB1 C4H4ORF29 UBAP1L K123 ELOVL1 TOMM6 CLOCK SNX13 ENO2 ZFHX3 B4GALT2 SCOC 6-Sep ARHGAP26 GID8 LOC420419 SOCS2 NDUFB1 ST6GALNAC1 LOC420849 STK10 ZBTB26 MOB4 RAD51 CLDN1 TRPV4 SCIN DCAF7 PLS3 RLIM LECT1 RDH10 CDH11 C15H12ORF49 GAPVD1 SLC46A3 EDNRA DDB2 GSPT1 AARS2 CCL20 LGI2 SLMO2 SOCS4 ASB9 MSRB3 WDR18 SERPINI1 RBM5 CDA TMEM30A CCT2 NME1 CAB39L DYNLT3 CTDSPL2 FZD3 MCEE PPP1R2 JMJD1C TAF8 UBE2G2 SGK196 HABP2 RASSF2 FEN1 AvBD8 PPME1 PTBP1 AC113404.1 CBFA2T3 FAM46C CST7 ARHGDIA FGF1 KIAA1143 NFIL3 CD44 RGS6 RGS4 PDGFD BRSK2 PLAC8L1 FKBP8 HRAS MYOZ2 FABP6 VDR FXYD6 ZMYND19 PLEKHB1 VSX2 RCHY1 FEM1B P2RX4 ORC3 TBCA MFAP3 RPL29 RAD21 C1H2ORF49 ABCA1 PNAT10 HPS1 CNDP2 METTL2A FDFT1 TSKU YWHAG CD151 SERPINB6 ANKRD40 SERHL2 ATG9A TSTA3 DAP LOC429492 NONO SLC26A5 CTPS2 SLC47A1 TSPO RARS2 RGS16 WDR43 CCR8 TULP1 ZDHHC21 LOC426385 ERCC3 PRLR TMEM70 SDK1 USP6NL TPH1 CRYL1 SYNM RPL6 KCNA4 EEF1A2 SBNO1 EXOC6 LSM8 PKIG TIAM2 MXI1 SLC16A3 HSPA9 C3H6ORF203 IRF4 TMED5 RASSF5 FBXW11 C3H6ORF154 ATG7 S100Z KIAA1671 FGL2 XIAP PUF60 PPP1R3E PACSIN2 LYPLA2 LOC776816 DCUN1D1 LHX9 LOC420209 PPHLN1 GEM AvBD1 GRIA2 VCAN INO80 BMP5 HMGCS1 NADK SLC1A2 CXCR7 GTDC1 TMEM56 PRKRIP1 BID B3GAT2 ADAM10 BRAP CDH8 EPN2 FZR1 SSU72 NCLN GHRH SGTA ELK3 VDAC1 MAPT TUBGCP2 SLC34A2 RBM19 GCHFR WDSUB1 STAT5B RAE1 SGTB DNAJA1 ESR2 TBL1X IRAK4 HIC2 SOX10 BOK ARF1 PIP4K2A RAB14 MYBPC3 DDX47 ATG4A TWSG1 GSKIP WASH1 EHD3 RP4-613B23.5 NDUFS1 PRTG ERBB3 TAPBPL SPATS2L C2ORF88 INTS2 TMLHE CBFB TPST2 OVALX STK11 TWF2 STARD4 SET YWHAE RAB33B MALL SPG7 PKM CDC2L1 SYCE3 DYL1 IL16 IHH ACBD3 NKIRAS2 DCT ELP6 ACAN SPP2 ATF7IP SRGAP1 EIF5A2 SMARCB1 NAPB RAP1GDS1 SLIT1 SNX3 COL18A1 GLP2R HIBCH MYOM2 TBL1XR1 TSPAN3 ACVR2A CCNL2 PDHX SLC47A2 CSNK1D ZBED4 FAM126A COX10 RPL15 UBE2R2 ADIPOQ E2F4 BLOC1S6 TAF5 VTN ALG12 HSPA5 BUB1B NELFA BORA OAZ2 BSG NRG1 COG4 TYRP1 LOC100859039 VNN1 SDF4 AIDA DNAJB12 PHF20L1 MRPS33 USP15 RP11-5A19.5 RP11-514O12.4 PLCXD1 TXNRD1 MON2 MIB2 TANK PDLIM5 EPYC COL22A1 EI24 OSTF1 NOTCH2 P2RY14 RHOC GPR107 UBN1 ZNF302 SEC61B TCOF1 IMMP1L SLC16A1 IGFBP1 CAPN1 GREM1 PIK3CB ANG RELL1 ZDHHC8 CDC25A MCCC1 TOB2 ATP5I DMTF1 RP1-309K20.6 PUS7 PRKCI IRF1 MAP6 PAIP2 BTBD10 C26H6ORF106 DIO3 TNFAIP8L1 CHUNK-1 EXOSC2 SGMS1 TEAD1 VPS45 RPRD1A MSN THRSP THY1 SNCG EMP1 LOC416354 PCMTD1 RIPK1 MPP5 TMEM254 B3GNTL2 CTSS STAU2 GATAD2A ASB12 GNRHR HPCAL1 ENTPD1 MRPL53 NCK2 ARHGAP19 HMGCR FOLR1 VTI1A ESR1 UGT8 SLC37A3 ATP6V0E1 ERLIN1 SLC25A15 NEK7 ATP6V0E2 CDK10 MLNR N4BP2L2 XPA HYAL6 KTN1 OSTN CMKLR1 RFFL MYL12A SRP14 TCP11L2 COL12A1 PLEKHJ1 MTMR8 TEAD4 AKAP2 PLEKHM1 DEXI TM4SF19 NIP7 ARFGAP2 COL6A2 MTMR3 RBMS1 ACOT9 PIGY SPI1 KBP B3GAT1 FOXD3 WASL C14H17ORF103 CD82 GPR149 MORN4 CCK RBBP4 DYNC1I2 SMPX C7H2ORF69 XIRP1 ARIH1 GPR174 SPG21 C1D MRPL28 C26H6ORF89 MOV10 USP45 PTPRO STRA13 UBE2G1 IMPG1 ENSA RBL2 SPATA2 F13A1 SMIM5 YTHDC1 SLC6A6 PLA2R1 CUEDC2 RINT1 ZNF767 TMEM68 USP12-like CBLN4 RAB19 MGEA5 CCKAR MFGE8 PALM CXCL14 RAG2 PRKAR1A KHDRBS1 CNP ST3GAL3 RREB1 DPF3 TMEM180 CPNE1 PTH OCM ACTR5 IPPK COX17 ARL6 NBL1 ALDH1A1 CD200 SRPR TMCO3 LYRM4 PSMD1 ABHD17B CISD1 GJB1 IGFBP4 TFEB MGAT4C SLC39A13 SALL4 ATM CLTB PSEN2 CDC42SE2 WRB FAM49A FAM222B EDEM1 SNAP91 DRAXIN ARIH2 COX4I1 CTNNBIP1 GLCCI1 UQCRFS1 GJA5 COX16 CEBPG CPT1A JUP TCTN3 RPL31 GPRIN2 PPP2R4 NUP50 WDR24 FZD5 YRK TMEM230 TRAPPC4 TMOD3 ENOX2 GFI1B KDSR LYRM9 SGMS2 MED24 FSHB SREBF1 C1H7ORF60 PHTF2 FAM20B IRX4 STRA6 CTD-2410N18.5 EXOC8 BASP1 PTK7 NGF AANAT CNTN2 SNRNP35 MAPKAP1 OXCT1 SLC40A1 LGALS3 PTDSS1 TTL DNAJB9 MGAT3 GABPA APCDD1 FHIT RNASEH2B APELA FBLN1 ANXA11 DHRS3 EMB SYK SLC8A3 LRPAP1 RECQL5 9-Sep RTKN2 TARSL2 GHSR BANP RFTN2 LGALSL TTPAL CSF3R ADD1 KLHL18 C11H19ORF40 RAF1 RNF111 NECAB3 ARID4A ZFYVE19 DPH1 CDH6 CUL2 ACE KPNA4 KLHL20 PDPK1 AZIN1 CACNG3 CDH17 ZP2 CSPG5 CMTM7 SH3BP2 DPM2 TRAF5 ITGB2 CTCF CCDC6 PRKCD MEF2BNB GIP CRIPT ERNI OSTM1 KIF5C NPC2 HSPA2 RAP1GAP2 PDGFA CDH7 IFIH1 GJA1 RRP1B TBX22 TNC PBRM1 MBL2 PTN GMPR NREP RAB3GAP2 SETD3 CPA6 RP5-966M1.6 NEK6 SLC17A9 WFDC1 ZSWIM7 COL17A1 ACBD6 POLK CHST10 TCF7L2 EDN2 DFNA5 TADA2A MPPE1 CUTC ATP6V0A4 FSTL1 MAFF RSFR NRK LEF1 ALCAM NR2C1 RALBP1 ESRP2 NDUFA5 TP53INP1 ANKRD16 JMJD6 ZFAND5 ITGA8 LOC426218 XPO1 PAX6 ORC5 5-Sep PSMD14 FUT9 AKT1 SUB1 SMPDL3B NEURL1 CSNK2A2 CANX AIFM1 TASP1 ADIPOR2 GNG2 PDS5B PDE4B CNTN5 KIAA1467 HAS2 SNX16 GLT8D2 WDR5 ADCK3 LDB3 FAM60A AR TERF1 GNAI1 PTGS2 LCLAT1 PEBP1 PPIB SYNGR3 LUM CBX3 FRS2 RNF152 A4GALT PIGA PMS1 SRSF2 CDKN1A DR1 KCTD2 EDA2R RAB11A KLHL15 GABRA1 ARHGAP8 ADA HDAC4 AMPD3 KDM5B AC005943.2 PPM1M PPDPF NDP USPL1 KRT5 BHLHE40 FIBIN MBNL1 HVCN1 MPPED2 CCR5 PTGES3 NHLH1 NSUN2 ASNSD1 SLC35E3 OAT PHAX TLDC1 EGR1 FLT4 TMEM129 CDCA4 ARHGAP15 USMG5 CASP18 MAGI3 RALGAPB NINJ1 TNFRSF18 QRICH1 ASIP SCFD1 PRRG1 SATB1 SRSF11 CYTH4 TNFRSF19 TRIP13 DMRT1 PAX9 UBIAD1 LMBRD2 RAB9A BTG4 KLHDC2 ABCC1 TAF1B SLC25A26 MAT1A SNCA CACNA2D1 LOC100858447 COPB1 LOC770639 TYRO3 FBXL21 EGFL7 NMU BIN1 FAM172A EXOC2 ST13 DNTT BRINP1 CSRP2 DMD POLE3 PEX2 TOLLIP RASA3 ATP13A4 ZNF609 MYH9 SULT1B ETV6 KLHL24 |
| gga-miR-6696-3p | MYH11 SPPL2B TMEM68 MGAT3 OAT PSMD10 MGLL RPS14 DNAJB6 NUDT5 CDC2L1 CCDC80 THYN1 SLC35G2 STAT4 FAM76A GJA5 SCG5 RNF13 POLR3H STARD4 C2ORF88 TMCO3 ARHGAP8 ACVR2A LYRM1 CCR8 LOC426385 MORC3 CTCF UBIAD1 LPGAT1 PMP22 BHLHE40 F-KER HERC3 RELL1 RNF4 MAP7 TAOK3 ARHGAP21 KLHL15 JDP2 ADAM10 CL2 IPPK HGF HP1BP3 TWIST2 FAM3C BID BEAN1 POU1F1 TSPO SEMA7A CPQ XIRP1 KIAA1671 ZFAND5 AvBD1 ACOX1 PLS1 CTNNBIP1 ZBTB17 KCNMA1 MASP1 TIAM2 SZRD1 FADS2 AKTIP SLC39A13 NGLY1 IL6ST PLEKHF2 FGF1 LCLAT1 MYLIP C11H16ORF70 PDLIM7 DAAM1 FAM45A TAX1BP1 DNAJA1 PDGFRA CDH6 ENY2 NELFA GABPA ENO1 CTTN TRAF7 PTPRG ELOVL4 NAGA GEMIN2 TMOD3 RNF25 RIPK1 CCPG1 FAM172A TTLL5 PLEKHA2 MYOM2 TBX18 IRAK4 IL5RA CACNA1B G3BP1 DCLRE1B BUB1 JAK2 HIC2 HMGA1 JMJD6 SHH ST3GAL3 SEPSECS PCMTD1 RNF185 SGMS2 FAM214A DNAJB9 TAF1B NDUFA5 T CLDN3 ATF4 PTPRO ENSA KIF4A GABRB3 RPL3 TYRP1 PIGK GET4 CD99 TMPO GMFB IGFBP4 RAB35 ZYX TNFRSF19 SH3BP2 SNRNP200 DYNC1I2 VDAC1 MTPN PDGFC AR BET1L CPNE1 PIAS2 ABCC4 EPGN DHCR7 LOC431324 CUEDC2 RP11-403P17.5 RP11-20I23.1 SERINC1 MAP2K2 SETD4 CEPT1 NCOA4 PRIMPOL IL8L2 LSM5 YPEL2 PRKRIP1 IKZF5 CACNA2D1 SNX10 SLC1A2 MRPL53 STK10 CPNE8 CYTH1 SRSF5A DCN MRPS16 TBX22 CKAP5 TTR REEP5 MAFA SETD3 SNX13 HOXB4 RGS16 ABCE1 API5 RBM19 LOC417800 SEC31A ESRP2 HSPA2 NME2 ZNF512B BZW1 FAM177A1 RNF14 B3GNTL2 CHD2 RTN4 ACLY FOXN2 BORCS5 NCF4 SMIM19 HAO1 COL8A1 SIX1 TARS NBL1 SCFD1 SELO MLX SERPINA1 SOUL FBXL16 CALD1 STRA6 C4BPA QDPR TST TOLLIP NUP50 IER3IP1 ELAVL4 MMP16 NACA NAA50 LOC421740 SCNN1A MPC1 MTMR8 GSTA METAP2 AGO4 GTF2H4 CHRNA6 HOXB3 NOC2L RPIA CCND1 C1GALT1 APELA DNMT3B SUMO3 AIDA WDR44 TP53I11 GATAD2A PCM1 S100A10 POLDIP3 XPA SLC16A9 MGAT4A SLC35A3 CDPF1 HNRNPDL MRRF CTD-2370N5.3 CLOCK CD44 DIO2 TMEM180 KDM5B RNF220 ABHD13 LDHA YY1 PELI1 PHTF2 RNF152 PHF20L1 TM4SF19 ASNS RAF1 CD247 TMEM167A FSHR TDRD7 NME5 EFTUD1 PRPF3 AARS DMD TMIGD1 9-Sep TRPM7 APCDD1 ALDH1A2 CD276 UBXN2A MYL4 NR0B2 DCUN1D1 JUP NOS2 FAM49A ATP5I ACSBG2 MCCC1 FEN1 ARMC7 CCDC127 CRIM1 CD151 STX6 IFIH1 C1H2ORF49 UMPS NAP1L4 NGF SFTPA1 VPS41 DCTD DEPDC1B MAP1LC3B FZD10 SAMM50 PRKAA1 SYT13 GTDC1 MYO5A SNRNP35 SPTAN1 IVD LOC100859249 BCL2L1 SRGAP1 DDX4 B3GAT1 G2E3 PLIN2 TAF12 CDKN1A PHF21A DERA VNN1 SLC6A6 FASN COPB1 IRX4 NR1H3 COPE EXT2 TNR TEAD4 PKM ARNTL GFI1B ARRDC1 CDCA4 WDR92 CCDC101 6-Sep ARL14EP IFRD1 RBM5 RABGAP1L ARFGAP2 MGAT4C SGK196 CAPN3 GANC NAIF1 NDUFB2 RHOA HNRPK CHP1 CHN1 SCIN TMEM173 GDI2 ARFGAP3 RAB10 UBN2 SDK1 CD82 ENS-1 FBXL21 ALG6 TAF11 KDSR DYNLL2 MPZL2 SUCLG1 RPS19BP1 STX17 HMGCS1 MGEA5 SGTA GDPD5 TLR4 DKC1 SERTAD2 DRG2 CRTAP MMP13 RAB3GAP2 CIAPIN1 PDE4B FAM222B HYAL6 PIGA STAT5B KERA COL6A3 CSF3 LDB3 FGFR2 PHF14 ATF2 RGS19 TSEN2 GHRH PRMT3 GLI2 PLEKHB2 FOXD1 PPP4R2 AP4B1 CLDND1 MORN4 FBXW5 IPO13 ACTR2 MTX3 MXI1 UCHL5 BLOC1S5 RAG2 P2RX4 ACAN RPSA TLR2A DRAXIN NRF1 RPRD1A TRAF5 CECR1 CLDN1 H3F3B HAS2 RNF141 CD200 TMEM56 C1H11ORF54 SNCA ACBD5 EXOC5 VAV2 ADCK3 SSU72 ZNF384 LPP MEP1A POT1 RALGPS2 ESR2 OTX2 ANAPC10 SRP68 CTDSPL2 OLIG2 HAVCR1 TAF3 GNG2 ZNF706 HSPH1 EIF2B3 NRXN1 DDX47 PARD3 NME3 PALM ESF1 STK17A TXNRD3 PABPC1 GREM1 FBXO34 TNS1 CIR1 ASF1A TRIM27.1 MYL12A MED9 ACADSB TRPV4 PDCD6IP FAM102A MESDC2 RPL35A TPM2 PUS7 H1F0 AMPD3 NEUROG2 KCTD7 GJA1 TNS3 CST3 STOML1 RPL9 LGALS3 CAV3 HNRNPR CELF2 C1H12ORF23 TSPAN12 ERGIC2 PNAT3 GID8 RFNG NR1D2 DNAJC24 JAK1 GTSE1 RFXANK YIPF1 NADK PSKH1 STRBP DNASE1 CMC2 CBFB AREG ARGLU1 C5H11ORF96 PIK3CD CRMP1 NINJ1 RAB8A NCAN FZD3 TRH PTDSS1 GJC1 KLF6 ST6GAL1 ZDHHC21 GCLM OXNAD1 SLC40A1 EXOC3 SERPINA4 SMIM15 PLA2G7 TCEANC2 ARIH1 ABCC1 HINT1 KBP ORC5 B4GALT2 FHL5 C4H4ORF29 ADORA2B CRYAB GOLGA7 RP5-1021I20.4 RTKN2 TARDBP ATM SNAP91 MYH1A OGG1 P4HA1 CEBPB C7H2ORF69 NRP1 ID2 TAL1 CD86 POLK SULT1C3 KBTBD4 CHORDC1 DR1 GALK2 TMEM104 RBM6 KLHL18 CRYAA BASP1 PDPK1 SENP8 TBC1D1 SGMS1 TNFAIP6 PARN KLHL2 LARP4 HDLBP DUSP10 PANK4 BCL10 NT5C3B SNX3 ZFHX3 N6AMT2 SMCO4 RNF111 MYL9 SNN LUZP2 PDLIM3 EEF1A1 TBC1D23 LMX1A DDX42 PIAS1 ZFYVE21 CSNK1D KCTD9 RFTN2 TRIAP1 BMP10 CDC42SE2 A4GALT WFDC1 SLC7A9 FAT3 PFN2 HNRNPD ELOVL1 CDKN1B RAD54L ASB7 C18ORF42 PUM1 ZNF639 MON2 MXD4 EIF1AY CHRDL1 GLOD4 ERLIN1 OC3 TYRO3 MFN1 VTI1A MRPS33 UBE2J1 PSMC5 GFRA2 DCK TNC ANK3 TNFRSF1A SRSF2 TMEM230 HOPX TMEM229B TMEM41B REL TINAG E2F1 AVEN CPA6 PNRC2 CYP2C45 ZNF622 WDFY2 NEUROG1 ZDHHC8 CHAC1 PLAG1 KCNH6 BANP MITF TRIM59 XIAP THY1 CGA UBE2V1 RBMX OSTM1 TARSL2 INTS2 NELFB LGI1 GLUL FURIN SRSF11 MBL2 RARRES1 BIRC2 UBXN10 BRINP1 CPT1A RANGAP1 ABHD17B C26H6ORF89 CKMT2 GLRX PLEKHO1 STAU2 CPSF6 ZNF800 ARIH2 ARPC1B KRT5 C12H3ORF37 MPPED2 PSMC6 RBM12B RP4-613B23.5 UBE3C SLC35E3 TCF7L2 JMJD7 SBNO1 PARK7 ANKH YWHAZ RAP1GDS1 RRAGC BOK RPL31 MYOZ2 PIK3CB CSTB RGS6 GALNT1 PHF5A ENO2 RLIM COL1A2 ATP6V0D2 PYROXD1 LOC415756 CHAF1B FAM98A TLE4 INSIG1 AKT1 COL9A1 GLP2R COG4 FBXO8 RGS20 LIN52 BBS2 FAM114A1 SLC2A2 PREP ZBTB2 SLC34A2 EIF6 SLC25A22 SAR1B ARFIP1 XKR9 RBM12 LRRC28 SYT12 AL158801.1 MAPK6 YPEL5 GJA4 LOC421975 SOX3 WIPI2 XPO5 SLC17A9 C13H5ORF15 OSBPL2 ACVR1 BAZ2B BRSK2 NEURL1 SMAD2 NPAS2 SASS6 LMO4 ELAVL1 SYNM SZT2 LOC431316 SDC2 PCMT1 RDH10 TOP1 ATP6V0E1 GLT8D2 CASP14 MTMR3 GNB1 WASH1 GSTO1 CNOT7 PLEKHJ1 YIPF3 QRICH1 NECAP2 GTF2E2 FOLR1 CYP24A1 HDAC9 MARCKSL1 ACP2 NRK GJB1 GBE ZDHHC17 LYRM9 FAM18B1 EIF5B AGPAT9 PRC1 NPL COL6A1 ANXA7 PRRX2 NFYA DTYMK NEIL2 TANGO2 PLN TBC1D22A ADIPOR2 CA2 TSKU C28H19ORF10 SPTSSB LOC418424 C20H20ORF24 MSRB3 C5H11ORF58 RUVBL1 WASL TIMM9 LOC426913 ABCA1 ST3GAL5 WWOX RPS8 GORASP1 CDH17 PRRG1 GFOD2 F2 STXBP3 PSMD1 LOC416354 ANG FABP4 ARMC1 MET FAM122A TMEM208 UCKL1 RP1-309K20.6 ESD LIN9 GTF3C5 MRPS7 MAPRE1 SOCS2 NRG1 SUGP1 RIPK2 WNT2B AANAT NAT10 APBB1IP CSRP3 NDUFA4 SRPR N6AMT1 TCEB1 EAF2 ANGPTL2 DYNC1LI2 C3H6ORF120 MFAP1 NFIL3 SMIM18 TUBB2B LXN SERINC3 ACTN1 SLX4IP CSNK2A1 PDCD5 PBRM1 C3H6ORF154 SOX10 LOC769139 CSRP2 BCL6 GPR107 CLDN2 LMBRD2 EPB41 ENOX2 PTGS2 RAD52 TSC22D3 PTN ART4 USP48 NTMT1 SSB ELOVL6 PDCL3 GFPT1 FLT4 FLI1 HMGCR CXCL14 ELOVL5 ETFDH UGGT2 RAC2 P2RY14 TSTA3 MRPS26 LOC425362 TPRA1 MAP6 PPAT EPHA1 LYRM2 AZIN1 NT5C2 EXOC6 RB1 PHACTR1 IL16 SNAP23 LEPR PDHA1 GUCA2B INIP ELN RAB5A HMG20A TACC3 TXNDC5 CNTN5 CTNNA2 MAPT DYL1 CDC25A FAP RCHY1 RAB14 LYSMD3 NR3C2 SEC22B DEAF1 TNFSF10 RPL32 ZCCHC17 PDGFD COQ5 GCH1 CTDSPL RP11-145E5.5 SERPINH1 NDP NAT FZD1 ARFGAP1 DAP TMEM5 PEX5 SULT1B SYNPR GSPT1 SRSF1 COL2A1 ARL6IP5 NCOA1 LOC100859427 SMPDL3B TNFAIP8L1 WIPF1 CD200R1L ANO5 UGP2 PAPD7 PPP2R2D HSD3B2 GNG5 LOC426914 INTS7 FAM20B HDAC4 LSG1 ICMT DLX6 HRAS |
